# Comparative analysis and correlation of cancer hotspot proteins and cell markers in tumor-normal adjacent breast and kidney samples using RPPA and LC-MS

**DOI:** 10.1038/s41598-026-48754-2

**Published:** 2026-05-18

**Authors:** Krisztina Paal, Noemi Karnok, Csaba Konrad, David Bui, Fanni Bugyi, Lilla Turiák, Christos Chinopoulos

**Affiliations:** 1 Department of Biochemistry, Semmelweis University, Budapest, 1094 Hungary; 2 Feil Family Brain and Mind Research Institute, Weill Cornell Medicine, New York, NY 10065 USA; 3 MTA-TTK Lendület (Momentum) Glycan Biomarker Research Group, HUN-REN Research Centre for Natural Sciences, Budapest, 1117 Hungary

**Keywords:** Liquid chromatography-mass spectrometry, Oncogene, Proteomics, Reverse phase protein array, Tumor-suppressor gene, Biomarkers, Cancer, Computational biology and bioinformatics, Oncology

## Abstract

Cancer proteomics is crucial for understanding tumor biology, yet consistent quantification of clinically relevant proteins across analytical platforms remains challenging. Here, we compared Reverse Phase Protein Array (RPPA) and Liquid Chromatography-Mass Spectrometry (LC-MS) using identical lysate aliquots prepared from matched tumor and adjacent normal tissues from breast and kidney cancer patients. We focused on proteins encoded by genes represented in the Ion AmpliSeq Cancer Hotspot Panel v2 and included representative cellular markers. RPPA was applied to the larger sample set, whereas data-dependent, data-independent, and targeted parallel reaction monitoring LC-MS modes were performed on a smaller subset of lysates. RPPA identified tumor-normal differences for several proteins and cell markers, while LC-MS quantified a smaller overlapping subset in the matched samples analyzed by both platforms. Cross-platform comparison showed that agreement was partial and strongly protein dependent: some targets displayed concordant tumor/normal fold changes, whereas others exhibited weak or even opposite-direction changes. Targeted PRM modestly improved agreement for selected discordant proteins, but substantial discrepancies remained. These data indicate that RPPA and LC-MS are not interchangeable for quantifying all targets in this panel, calling for a rather confirmatory approach: concordant findings increase confidence in selected candidate biomarkers, whereas discordant findings identify targets that require cautious interpretation.

**Supplementary Information:**

The online version contains supplementary material available at 10.1038/s41598-026-48754-2.

## Introduction

Cancer is driven by complex molecular alterations, and while genome sequencing efforts have catalogued numerous somatic mutations in tumors, the consequences of these alterations at the protein level are not fully captured by genomic data alone. Notably, protein expression and activity can be influenced by epigenetic, post-transcriptional, and post-translational mechanisms, leading to frequent discordances between mRNA levels (or DNA mutations) and actual protein abundances in tumors^1^. This persistent divergence between nucleic‑acid readouts and protein abundance/activity highlights the need for direct proteomic analyses in cancer research to accurately characterize tumor biology and identify clinically relevant biomarkers.

In recent years, advanced proteomic technologies have enabled more comprehensive profiling of the cancer proteome. Mass spectrometry (MS)-based approaches, particularly Liquid Chromatography–Mass Spectrometry (LC-MS), can unbiasedly identify and quantify thousands of proteins in a given sample. Large-scale initiatives such as the Clinical Proteomic Tumor Analysis Consortium (CPTAC) have applied LC-MS to deeply profile tumor proteomes, routinely quantifying on the order of ten thousand proteins per specimen^2^, revealing new molecular subtypes and pathways across diverse cancers^3^. Complementing MS-based discovery, antibody-based platforms like Reverse Phase Protein Array (RPPA) allow high-throughput quantification of predefined protein targets across hundreds or even thousands of specimens. For example, The Cancer Genome Atlas (TCGA) project utilized RPPA to measure ~ 200 key signaling proteins and phosphoproteins in more than 8,000 tumor samples, providing a focused view of cancer-driving pathways at the protein level^4^. Each platform offers distinct advantages: LC-MS affords broad proteome coverage and the ability to detect unexpected or novel proteins, whereas RPPA provides sensitive relative quantitation of specific low-abundance proteins using minimal sample input and typically at substantially lower per-sample operational cost than LC-MS^5^.

RPPA and LC-MS differ fundamentally in their measurement principles. In RPPA, serially diluted lysates are arrayed on nitrocellulose and probed with validated antibodies, enabling sensitive, high-throughput quantification of a predefined target panel with minimal sample input. However, RPPA performance depends on antibody availability, specificity and epitope accessibility, and it provides limited information on unexpected proteins or isoforms. In contrast, LC-MS is antibody-independent and offers broad proteome coverage, but its quantitative precision and depth depend on acquisition mode (e.g., data-dependent acquisition (DDA) vs. data-independent acquisition (DIA)) and can be constrained by dynamic range, missing values and higher per-sample cost and analytical complexity. Targeted LC-MS approaches such as parallel reaction monitoring (PRM) can improve sensitivity and quantitative accuracy for selected proteins, but require prior peptide selection and method optimization.

Thus, despite their successes, both proteomic approaches harbor inherent limitations that hinder a complete understanding of tumor proteomes^6,7^. For example, MS-based analyses may incompletely capture very low-abundance or hydrophobic proteins and often require specialized expertise and instrumentation, while RPPA is restricted to available high-quality antibodies and be confounded by antibody specificity or cross-reactivity. Thus, no single method captures the entire dynamic range and complexity of the cancer proteome^8^. To address this, integrative studies have begun comparing MS and RPPA data, with results indicating that the two platforms often yield complementary insights; for instance, a recent parallel analysis of breast cancer tissues profiled by both LC-MS and RPPA showed limited overlap in differentially expressed proteins yet consistent tumor–normal discrimination and nonredundant biological insights^8^. In parallel, pan‑cancer proteomic compendia derived from deep LC-MS datasets demonstrate that protein-level patterns robustly recover major tumor subtypes across lineages^9^. These findings underscore the value of comparing and integrating antibody-based and MS-based platforms during biomarker discovery, assay development, and early translational validation to obtain a more comprehensive view of oncogenic protein networks than either method alone.

A critical next step is to determine how consistently these platforms quantify specific, clinically significant proteins and to understand any discrepancies between them. Accurate protein quantification is especially important in oncology because many clinical decisions hinge on protein biomarkers, for example, estrogen/progesterone receptor (ER/PR) and HER2 status guide therapy selection in breast cancer^10,11^. Moreover, numerous cancer-driving proteins of interest - such as receptor tyrosine kinases and signaling intermediates - are also drug targets, meaning that reliable measurement of their expression or activation status is directly relevant to patient treatment^12^. In routine clinical practice, however, treatment decisions are expected to rely on analytically validated clinical assays and would not ordinarily require repeated orthogonal confirmation. Orthogonal comparison is most valuable during biomarker discovery, assay development, and early translational validation, where it can increase confidence in candidate biomarkers and reveal false positives, false negatives, or other platform-specific biases inherent to a single technique. To date, however, few studies have rigorously compared RPPA and LC-MS side by side for a targeted panel of cancer-related proteins in patient-derived samples, especially across different tumor types. It also remains unclear how recent advances in MS data acquisition - including data-independent acquisition (DIA^13^ for improved reproducibility and targeted modes like parallel reaction monitoring (PRM^14^ for absolute quantitation - might influence concordance with antibody-based measurements.

In this study, we address these gaps by systematically analyzing and correlating protein expression data obtained from RPPA and LC-MS in human breast and kidney tumors, with matched adjacent normal tissues as controls. We focused on a defined set of proteins encoded by the 50 cancer-related genes included in the Ion AmpliSeq Cancer Hotspot Panel v2 gene list, along with selected cellular “housekeeping” markers used for normalization. Utilizing multiple LC-MS acquisition strategies (label-free data-dependent, data-independent/DIA, and targeted PRM), we quantified the abundance of these cancer-relevant proteins in each sample and directly compared the results to RPPA-based quantifications on the same specimens. This dual-platform approach allowed us to identify significant protein expression alterations between tumor and normal tissue, evaluate the overlap and divergence in proteins detected by each platform, and examine the correlation strength for proteins measured by both methods. Our study provides an in-depth assessment of the concordance and complementarity between RPPA and LC-MS in cancer proteomics. In this setting, cross-platform comparison is intended to inform biomarker discovery, assay development, and early translational evaluation-not to suggest that analytically validated clinical assays require routine orthogonal confirmation in everyday practice. The insights gained here highlight how integrated multi-platform proteomic analyses can strengthen biomarker nomination and analytical validation workflows in oncology research, thereby supporting the development of robust assays for precision medicine.

## Materials and methods

Samples: 586 breast tissue samples (293 matched tumor-adjacent pairs) and 192 kidney tissue samples (96 matched tumor-adjacent pairs) were provided by Voronezh State University and collected in the time interval 2017–2020. Informed consent was obtained from all subjects and/or their legal guardian(s) and documents are kept at the clinics of the Voronezh area where surgical excisions were performed. Normal samples were obtained from proximal, healthy-appearing tissue near the excision site of the tumor from each patient. No sample was embedded in FFPE. There was no macro-or microdissection of tumors prior to analysis. All samples were frozen to – 20 °C within 1 h of excision and shipped in dry ice in bar-coded cryovials. Upon arrival of the samples to the RPPA facility in Budapest they were kept at – 80 °C until further use. All methods were performed in accordance with the relevant guidelines and regulations outlined in the bilateral, transnational project 2017—.3.4-TET-RU-2017-00003. Ethical permission for this project has been obtained by ETT-TUKEB and is on permanent display in https://tinyurl.com/rppahuethicalperm (file number: 35302-5/2017/EKU). Personal data of the patients from whom the samples originated were alphanumerically encrypted using a 128-bit encryption algorithm and stored in a secure server.

RPPA: The workflow for RPPA was the following (detailed protocols can be found in http://rppa.hu/Protocols.html):

(i) solubilization of samples to lysates and protein amount determination: Samples (20–200 mg) were suspended in a buffer (named “Benzonase buffer”), the composition of which was: 20 mM Trizma, 2 mM MgCl_2_, protease inhibitors (EDTA-free, Thermo #A32955), Benzonase (Pierce #88700), 0.2 mM BeSO_4_ and 5 mM NaF, pH 7.2 (HCl). After a few minutes while samples were kept on ice, they were homogenized by bead beating in a Precellys 24 homogenizer (Bertin Technologies SAS) using zirconium oxide 2 mm beads (speed 5000 m/s, 2 cycles of 20 s). Subsequently, the homogenized samples were incubated at 37 °C while shaking @500 rpm. After 5 min, homogenates were spun @ 3500 rpm for 2.5 min. Subsequently, an equal amount of volume of a 2x Laemmli buffer (named “Laemmli buffer”) was added, the composition of which was: 4% SDS, 20% glycerol, 120 mM Trizma, 4 mM dithiothreitol (DTT), 5 mM EDTA, 5 mM EGTA, pH 6.8 (HCl) and the bead-beating protocol was repeated once. Then, tubes were incubated at 97.5 °C for 10 min, while shaking @500 rpm. Lysates were subsequently transferred to new tubes avoiding the top-lipid fraction and any remaining insoluble material, spun at 12,700 rpm @RT for 10 min. Supernatants were collected and diluted 10-fold using a 1-to-1 mixture of Benzonase buffer and Laemmli buffer. Subsequently, protein concentration was determined by IR spectrometry measuring amide bond absorbance using a Direct Detect IR Spectrometer (Merck KGaA). If protein concentration (of the undiluted lysate) was > 21 mg/ml, a calculated amount of buffer was added (to decrease concentration to < 21 mg/ml) and lysate protein concentration was re-estimated. All lysates were set to 1–20 mg/ml, aliquoted in new bar-coded cryovials and stored @ – 80 °C until use.
(ii) Lysates loading to 96-well plates and then to 384-well plates: Lysates were thawn while rocking and then loaded first in 96-well plates and then to 384-well plates using an automated epMotion 5073 liquid handling system (Eppendorf, AG). Dilutions were performed iteratively so that each lysate is greater or equal than 2.05 mg/ml. At the end of each robotic run, each lysate was present in 1x, 2x, 4x, 8x and 16x fold dilutions in 4 replicates each, per 384-well plate. These five dilution points were part of the technical dilution series of the same lysate and were not treated as separate biological replicates. Well plates were then bar-coded, sealed and kept @ – 80 °C until use.
(iii) Spotting of lysates using an Arrayer: Spotting of the lysates occurred by transferring 1 nL of lysate from the 384-well plate (all dilutions and replicates) on Oncyte SuperNOVA nitrocellulose bar-coded slides (Grace Bio-labs) twice per slide using an Aushon 2470 arrayer with 16 pins (Aushon, Billerica, MA, USA). Thus, each sample contributed 40 technical spots per slide (5 dilutions x 4 within-plate replicates x duplicate printing). 13–15 well plates and 50 slides were loaded in the arrayer per run, thus, each slide was spotted by 9660 spots. Each spotting run lasted ~ 29 h. To counter batch effects, samples were randomly assigned into two, partially overlapping runs. Because of the high number of samples the distribution of protein loaded should be the same between the runs. We observed significant batch effects for some antibodies, therefore the runs were normalized to have equal mean values for each antibody between the two runs; supplementary Fig. 1 C (unnormalized) and 1D (normalized) show histograms of RPPA read intensities for Vimentin, which was an example of an antibody with a strong batch effect. Slides were kept in thermo-controlled incubators @15 °C until use.
(iv) Staining of slides: Slides were manually stained in incubation chambers (Grace Bio-labs) using a quadruple amplification protocol that allowed using extremely low titers of antibodies, see supplementary Tables 2 and 3. Specifically: slides were re-hydrated with Tris-buffered saline containing 0.1% Tween-20 for 10 min. Subsequently, they were ‘blocked’ with 5% bovine serum albumin (Sigma, A7906) and 0.1% Tween-20 for 1 h. Slides were subsequently treated with Avidin (immediately after the block step without washing) followed by Biotin, followed by 0.3% H_2_O_2_ and several wash steps in-between and before applying the primary (supplementary Tables 2 and 3) and secondary antibodies (these were either donkey anti-mouse or donkey anti-rabbit, depending on the host organism where the primary antibody was raised; titers of secondary antibodies were 1:10,000 for both). All antibodies were pre-tested by western blotting using the same quadruple amplification protocol described above or pre-validated by the RPPA platform of Institute Curie (IC, https://institut-curie.org/) and see supplementary Tables 2 and 3. Afterwards, slides were treated with an amplification module (Bio-Rad, cat # 1708230) followed by applying streptavidin conjugated with IRDye 800 (1:2,000) and several wash steps in-between. In addition, one slide per RPPA run was stained by FCF Fast Green (Sigma F7258) for total protein normalization of spots during the imaging process (see below). Slides were scanned as described below within a few hours after the last washing step and following a short drying protocol by spinning at 300 rpm for 5 min.
(v) Slide/blot imaging and spot quantification: Slides were loaded (24 slides at a time, using an autoloader) in an Innoscan 710-IR (Innopsys Inc). The FCF-stained slide was scanned at 670 nm, and the IRDye 800-stained slides at 785 nm, using the GAL file generated by the Arrayer. Spot quantification and output to excel was automatically performed using MAPIX software (Innopsys).

Antibody validation: Antibodies were selected according to the following criteria: (i) reactivity to human tissues, (ii) monoclonality (raised in rabbits or mice) or recombinant, (iii) monospecificity (in Western blot), and if applicable, (iv) the epitope should not be located in hotspot area. On the basis of the above, > 400 commercially available antibodies were pre-screened and ~ 100 selected and tested. Antibody monospecificity was verified using standard Western blotting, using 7 random breast- and 7 random kidney samples. Blots were imaged using an Azure 600 Imaging System. 56 antibodies against 48 proteins were deemed as suitable for RPPA. Some were pre-validated by the RPPA platform of Institute Curie (https://institut-curie.org/), see also supplementary Tables 2 and 3. For PIK3CA and SMO proteins, no suitable antibodies were found despite testing several commercially available items. Detailed information regarding all antibodies can be found in db.rppa.hu portal (registration required; contact the corresponding author C.C.). Representative Western blots for selected antibodies/proteins are shown in Supplementary Fig. 12. These blots are included primarily to demonstrate that the antibodies used for RPPA yield predominantly single bands at the expected molecular weight under our assay conditions, thereby supporting their suitability for the dot-based RPPA assay. They are not intended to function as a third quantitative platform for formal correlation analysis. The lysates used for WB, RPPA and LC-MS were from the same aliquots.

Data analysis: Normalization of RPPA data was done by the protein stain (FCF) data using a custom-made python script (https://github.com/csabak/RPPA/tree/main). First, background was subtracted and the exponential function y = A × exp(K × x) + C was fitted to the dilution series of each sample individually, including all replicate spots, where y is the fluorescence value (Fprot), x is the dilution denominator, and A, K, and C are fitted parameters (see also Supplementary Fig. 1B). Background was then subtracted from the antibody values, values below 3000 intensity were marked as low reads and discarded, and each remaining spot read was divided by the FCF value calculated at the respective dilution step from the sample-specific fitted curve; Fprot values with z-scores above 3 or below − 3 were also discarded. Thus, each reported RPPA sample-level value is a derived empirical estimate based on multiple technical spots across five dilutions after filtering, curve fitting, and normalization, rather than a single direct observation. The uncertainty introduced by these sequential processing steps was not formally propagated into a sample-level error term in the present study, and therefore small differences in normalized RPPA values should be interpreted cautiously. Of note, RPPA response curves are typically monotone and often saturating; accordingly, widely used workflows quantify dilution series using logistic/sigmoidal models or monotone smoothers. However, over restricted signal ranges, simpler parametric fits can approximate the dilution-signal relationship. We therefore use an exponential functional form as an empirical approximation within the non-saturated working range considered here. Furthermore, the comparison of multiple RPPA cell-marker normalizers and multiple LC-MS scaling schemes was treated as an exploratory sensitivity analysis of cross-platform concordance rather than as a formal inferential model-selection exercise. Accordingly, we report the full screen in the supplementary datasets and use the resulting rankings descriptively to guide dataset-specific visualization choices. Multiple-testing correction was applied in the formal differential-expression analyses (e.g. the volcano plots in Fig. 3 and the adjusted *p*-values used in the DIA workflow), but not across the exploratory screen of normalization/scaling strategies.

Plots of RPPA were generated by SuperPlotsOfData web app^15^; volcano plots were generated by the VolcaNoseR web app^16^. Slope-colored paired RPPA data were generated in ggplot2 using a custom-made R script deposited at https://github.com/JoachimGoedhart/Slopes-colored. Normalization of RPPA oncoproteins and tumor suppressor proteins to cell markers data was plotted by PlotsOfDifferences^17^.

LC-MS’ (DDA mode): Sample preparation: 10 µg proteins were digested from each lysate. The proteins were precipitated using 90 µL ice-cold ethanol overnight at – 20 °C. Samples were centrifuged at 4 °C, 14,000xg for 20 min. The pellets were washed twice with 200 µL ice-cold ethanol to remove the detergent. The pellets were re-dissolved in 20 µL 8 M urea of 50 mM ammonium bicarbonate. The proteins were reduced using 5 mM dithiothreitol for 30 min at 37 °C, then alkylated using 10 mM iodoacetamide for 30 min at room temperature in the dark. The samples were then diluted tenfold by 50 mM ammonium bicarbonate. Enzymatic digestion was performed first by adding LysC-Trypsin mixture in 1:100 ratio and incubation for 1 h. Subsequently, Trypsin was added in a 1:50 ratio in the second cycle and incubated overnight. After the digestion steps, the digestion was stopped by adding 0.5 µL formic acid. Peptide extracts were then dried and stored at − 20 °C until further usage.

Reversed-phase purification: Thermo Pierce C_18_ spin columns (Kvalitex, Hungary) were used for desalting and clean-up. After the column was conditioned, washed, and equilibrated, the sample was loaded onto the column in 0.1% TFA in water. The elution was performed with 30:70 v/v% water: ACN. After the elution, the samples were dried and stored at − 20 °C until further usage.

NanoUHPLC-MS(MS) analysis: Each proteomic sample was reconstituted in 20 µl 0.1% FA in 2:98 v/v% ACN: H_2_O, out of which 1 µl was injected. Samples were analyzed using a Maxis II QTOF instrument equipped with a CaptiveSpray nanoBooster ion source coupled to a Waters nanoAcquity nanoUHPLC system (Waters, Milford, MA, USA). Peptides were separated on an Acquity M-Class BEH130 C_18_ analytical column (1.7 μm, 75 μm × 250 mm, Waters Hungary) using gradient elution (isocratic hold at 12% B solvent content for 1 min, then elevating B solvent content to 25% in 33 min, and to 45% in 20 min) following 3 min trapping with 15 µL/min on a Symmetry C_18_ trap column (5 μm, 180 μm × 20 mm, Waters, Budapest, Hungary). Solvent A consisted of 0.1% FA in water and Solvent B was 0.1% FA in ACN.

For MS data collection, DDA measurements were performed. The cycle time was set at 2.5 s, with a dynamic MS/MS exclusion of the same precursor ion for 2 min, or if its intensity is at least 3 times larger than before. Preferred charge states were set between + 2 and + 5. MS spectra were acquired at 3 Hz in the 150–2200 m/z range, while MS/MS spectra were at 4–16 Hz depending on the intensity of the precursor. Internal calibration was performed by infusing sodium formate and raw data was recalibrated using the Compass DataAnalysis software 4.3 (Bruker Daltonics, Bremen, Germany).

Data analysis: Protein identification was performed by Byonic v5.0.20^18^ (https://proteinmetrics.com) on Homo sapiens database. Protein quantitation was performed using MaxQuant v2.4.0.0^19^ (https://maxquant.org) on a focused database, made from merging Byonic search results from all proteomic MS/MS analyses separately for breast and kidney tissues. The exact parameters used for all the software used at HUN-REN Research Centre for Natural Sciences are summarized in Supplementary Table Parameters_Byonic_MaxQuant.xlsx. Experimental data measured at HUN-REN Research Centre for Natural Sciences were submitted to the MassIVE data repository with the ID: MSV000095298. The data are available through this link https://doi.org/doi:10.25345/C5Q23RB5V.

The initial DDA screen comprised 32 lysates (8 breast tumor, 8 matched breast adjacent, 8 kidney tumor, and 8 matched kidney adjacent samples). For the downstream DIA and PRM analyses, selection was performed at the level of matched tumor–adjacent patient pairs rather than individual lysates. Specifically, the final DIA/PRM cohort consisted of four matched breast tumor-adjacent pairs and four matched kidney tumor–adjacent pairs (16 lysates total), representing the top 50% of the DDA-screened lysates in terms of protein identifications.

LC-MS (DIA mode): Protein lysates of tissue samples were processed using filter-aided sample preparation (FASP) as described elsewhere^20^ using trypsin as protease (enzyme protein ratio of 1:50; sequencing grade; Promega). The resulting peptides were extracted into LC-MS vial by 2.5% formic acid (FA) in 50% acetonitrile (ACN) and 100% ACN with the addition of polyethylene glycol (final concentration 0.001%)^21^ and concentrated in a SpeedVac concentrator (Thermo Fisher Scientific).

LC-MS/MS analyses of all peptides were done using UltiMate 3000 RSLCnano system (Thermo Fisher Scientific) connected to timsTOF Pro 2 mass spectrometer (Bruker). Before LC separation, tryptic digests were online concentrated and desalted using a trapping column (300 μm × 5 mm, µPrecolumn, 5 μm particles, PepMap Neo Trap Cartridge, Thermo Fisher Scientific). The trap column was then washed with 0.1% trifluoroacetic acid and the peptides were eluted in backflush mode from the trapping column onto an analytical column (Aurora C18, 75 μm ID, 250 mm long, 1.7 μm particles, PN AUR3-25075C18-CSI; Ion Opticks) by linear 2 h gradient program (flow rate 200 nl.min-1, 3–42% of mobile phase B; mobile phase A: 0.1% FA in water; mobile phase B: 0.1% FA in 80% ACN) followed by a system wash using 80% of mobile phase B. Equilibration of the trapping column and the analytical column was done before sample injection to sample loop. The analytical column was installed in the Captive Spray ion source (Bruker; temperatures set to 50 °C) according to the manufacturer’s instructions. Spray voltage was set to 1.5 kV.

MSn data were acquired in data-independent acquisition (DIA) mode with m/z range of 100–1700 and 1/k0 range of 0.6–1.4 V×s×cm-2. Dedicated DIA windows schemes (see diaParameters.xlsx in the supplementary material for more details) were generated using custom script based on peptide identifications coming from the data-dependent acquisition (DDA) analyses tests on pooled breast or kidney tissue samples. diaPateremets.xlsx defined m/z 400–1000 precursor range, 24 windows using two steps for each PASEF scan and cycle time of 100 ms locked to 100% duty cycle.

Four gas phase fractions (GPF) differing in the IM range covered were run on top of the samples solution using pooled sample of all kidney or breast tissue samples. The MS system was calibrated for 0.25 ion mobility range prior the GPF analyses.

Pooled samples for the DDA and GPF data analyses were prepared separately from breast and kidney tissue samples replicates using aliquot of 750ng from individual sample replicates. The same instrumentation was used as for the DIA, DDA and GPF analyses.

DIA data were processed in DIA-NN (version 1.8.1)^22,23^ in library-free mode (separately for the kidney and breast tissue sample analyses) against the modified UniProtKB protein database for Homo sapiens (https://www.uniprot.org/proteomes/UP000005640); version 2024/03, number of protein sequences: 20,598. The original UniProtKB database was supplemented by iRT peptide sequences (Biognosys) and proteins of interest accessions were adjusted with suffix “-target”. No optional, but carbamidomethylation as fixed modification and trypsin/P enzyme with 1 allowed missed cleavage and peptide length 7–30 were set during the library preparation. False discovery rate (FDR) control was set to 1% FDR. MS1 and MS2 accuracies as well as scan window parameters were set based on the initial test searches (median value from all samples ascertained parameter values). MBR was switched on and GPF analyses were searched along the sample analyses. Kidney and breast tissue samples analyses were processed separately in DIA-NN.

Protein MaxLFQ intensities reported in the DIA-NN main report file were further processed (separately for kidney and breast tissue sample analyses) using the software container environment (https://github.com/OmicsWorkflows/KNIME_docker_vnc), version 4.7.7a. Processing workflow is available upon request. Briefly, it covered: (a) removal of low-quality precursors and protein groups, (b) precursor intensities normalization by loessF algorithm, (c) precursor intensities imputation by global quantile (0.001), (d) protein group MaxLFQ intensities calculation using iq R package and log2 transformation, and (e) differential expression analysis using LIMMA statistical test – proteins with adjusted p-value < 0.05 and log2 fold change >|1| were considered as significantly changing. DIA data (breast and kidney, respectively) are available through the PRIDE website using the following account details: Username: reviewer_pxd060845@ebi.ac.uk, Password: vmB54d1jA6A5; and Username: reviewer_pxd060846@ebi.ac.uk, Password: A3IFh7sRn7Qm.

LC-MS (PRM mode): The peptide solutions analysed using DIA approach were run also using targeted mode (PRM – parallel reaction monitoring) using Ultimate 3000 RSLCnano system connected to Orbitrap Exploris 480 mass spectrometer (Thermo Fisher Scientific) with EASY Spray ion source (Thermo Fisher Scientific) installed. Prior to LC separation, tryptic digests were online concentrated and desalted using trapping column (300 μm × 5 mm, µPrecolumn, 5 μm particles, PepMap Neo Trap Cartridge, Thermo Fisher Scientific). After washing of trapping column with 0.1% trifluoroacetic acid, the peptides were eluted (flow rate 200 nl.min^− 1^) from the trapping column onto separation column (Aurora C18, 1.7 μm particles, 75 μm × 250 mm, P/N AUR3-25075C18-TS; IonOpticks) by 90 min long gradient (0 min: 3% B, 90 min: 42% B; mobile phase A: 0.1% FA in water; mobile phase B: 0.1% FA in 80% acetonitrile) followed by a system wash using 80% of mobile phase B. Equilibration of the trapping column and the analytical column was done before sample injection to sample loop. The analytical column was installed in the EASY Spray ion source according to the manufacturer’s instructions. The analytical column temperature was set to 50 °C (Column Heater, IonOpticks). Spray voltage and sweep gas pressure was set to 1.6 kV and 1 Arb, respectively.

MS data were acquired in a product ion scan mode with additional survey scan measurement. The product ion scan mode was set for the selected precursors of the proteins of interest (see PRM_transitions.csv in the supplementary material for more details) monitored using optimized retention time scheduled acquisition mode. Product ion spectra were set as follows: custom AGC target of 3000%, automatic maximal injection time mode, isolation width 0.7 Th, relative fragmentation energy 30%, orbitrap analyser resolution 30,000, first mass m/z 120.

The PRM method was iteratively optimized using DDA and DIA analyses run on the same system utilizing the pooled samples (see above) and based on which the peptides for the proteins of interest were selected (proteotypic peptides, not containing C, M, N or D amino acids if possible). Further adjustment of the monitored peptides list and scheduled method retention times was done in an attempt to increase sensitivity using also dedicated PRM analyses monitoring several target peptides only. PRM data were manually evaluated using Skyline with parallel database searching using in-house Mascot search engine (version 2.6.2). UniProtKB Homo sapiens (see above) as well as the cRAP contamination (https://www.thegpm.org/crap/ downloaded 2018-11-22) databases were used, fragment search tolerance 20ppm. Peptide spectrum matches having Mascot expectation value < 0.05 were considered only and used to build the Skyline library used during the data evaluation. Fragments monitored were selected automatically based on the MS2 spectra with the inclusion of fragments with m/z > precursor. The acquired LC-MS/MS data were manually evaluated using Skyline-daily processing software (version 24_1_1_284). PRM data are available via ProteomeXchange with identifier PXD060847; Username: reviewer_pxd060847@ebi.ac.uk, Password: 3Hr05avTzSrM.

## Results

### Experimental design and parallel workflow of RPPA and LC-MS

**Fig. 1:**
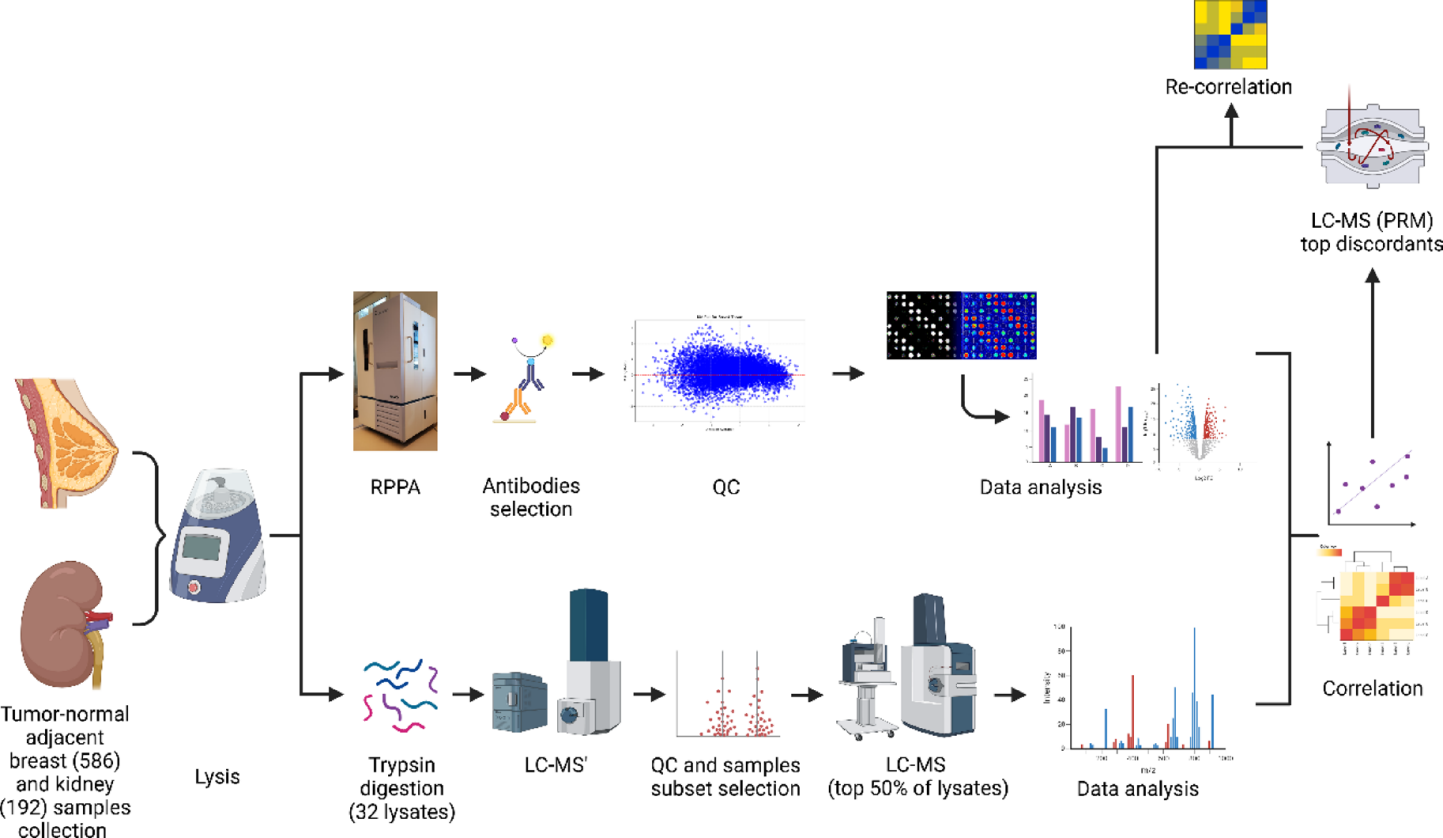
Overview of the experimental workflow. Tumor-normal adjacent breast (586 samples) and kidney (192 samples) tissues were collected and underwent lysis to extract proteins. All lysates were spotted on nitrocellulose slides using RPPA technology as five-point dilution series (1x, 2x, 4x, 8x and 16x), with four within-plate replicates and duplicate printing, yielding 40 technical spots per sample. Antibodies recognizing hotspot proteins and cell markers were selected upon screening for monospecificity. After quality control (QC) of the printing process and staining, RPPA values were calculated from all valid normalized spots of the full dilution series; no single dilution was selected. Thirty-two lysates (8 breast tumor, 8 breast healthy adjacent, 8 kidney tumor and 8 kidney healthy adjacent) underwent an initial DDA-LC-MS screen (denoted as LC-MS’ throughout the text as it took place in a different facility). From these results, the top 50% of lysates (those yielding the highest number of detected proteins) were selected for subsequent deeper DIA-LC-MS analysis. Ultimately, correlation analysis was conducted to evaluate the relationship between protein expression levels measured by RPPA and LC-MS across the 16 selected samples. All LC-MS measurements were performed on aliquots of the same lysates that were profiled by RPPA, enabling direct within-sample comparisons across platforms. Created in BioRender, https://BioRender.com/26ik699, licensed under CC BY 4.0.

The overall workflow is depicted in Fig. 1. Breast tumor and adjacent normal tissue were obtained from 293 patients aged 29–83 years; kidney tumor and adjacent normal tissue were obtained from 96 patients aged 33–84 years. Their age distribution is shown in the violin plots of supplementary Fig. 1A. Each sample was converted to lysate, as described under the section of RPPA in the Materials and Methods, and was represented by 40 technical spots per antibody-stained slide: five dilution points (1x, 2x, 4x, 8x and 16x), four within-plate replicates, and duplicate printing, as shown in supplementary Fig. 1B. These five dilution points were not treated as separate biological replicates and no single dilution was selected for plotting. In supplementary Fig. 1B, the term “0R7Z4MA_002_T_1” is an alphanumerically encrypted tumor sample. All lysates were probed by RPPA, while some were additionally processed for subsequent LC-MS (see below, this section). Regarding the RPPA branch of the workflow, we selected the Ion AmpliSeq Cancer Hotspot Panel v2 as the gene list used to define the protein panel of interest because it is the most commonly employed cancer hotspot panel among datasets, grants, patents, clinical trials, and policy documents, see supplementary Table 1. The panel comprises 50 cancer-related genes, and RPPA was used to measure the abundance of proteins encoded by these genes, subject to antibody availability, rather than mutations per se. We were able to identify RPPA-suitable antibodies for 48 of these 50 proteins [for APC, p16INK4A, VEGFR2, NPM1, RET, SMARCB1 and SRC, two suitable antibodies were found, designated as (AB1) and (AB2)]. No suitable antibody was found for PIK3CA and SMO proteins. Name of proteins, their aliases, abbreviation, short description, Research Resource Identifiers (RRIDs) of antibodies used, epitopes and titers are shown in supplementary Table 2. Arbitrarily, we have divided these 48 proteins in oncoproteins and tumor-suppressor proteins to minimize figure panels clutter, even though several of them exhibit both functions depending on conditions^24–26^. We labeled the following as oncoproteins: AKT1, ALK, BRAF, CTNNB1, EGFR, FGFR1, FLT3, GNA11, GNAQ, HRAS, IDH1, IDH2, JAK3, KIT, KRAS, MET, NRAS, PDGFRA, SRC, TPOR, VEGFR2. Those labelled as tumor suppressor proteins were: ABL1, APC, ATM, CDH1, CSF1R, ERBB2, ERBB4, EZH2, p16INK4A, p14ARF, FBXW7, FGFR2, FGFR3, GNAS, HNF1A, JAK2, MLH1, NOTCH1, NPM1, PTEN, RB1, RET, SHP2, SMAD4, SMARCB1, STK11, TP53, VHL. Finally, we used antibodies for these house-keeping proteins: ACTB, CALR, CAV1, DES, FBL, GOLGA2, IGF2R, KRT19, LSD1, NAKAa1, NUP98, RPS3, TUBA1A, VIM. Supplementary Table 3 lists the cell markers, their abbreviations, antibody RRIDs, and titers. The cell markers were selected to represent a most wide repertoire of cellular functions/compartments as described previously^27^. RPPA tumor-adjacent comparisons were additionally visualized by MA plots (Supplementary Figs. 2–4). Because these plots are based on a small targeted cancer-hotspot/cell-marker panel and on normalized RPPA summary values rather than on an unbiased proteome-wide measurement matrix, conventional MA-plot expectations are of limited interpretability here. We therefore retain these plots only as descriptive supplementary displays and do not use them as stand-alone evidence for global up- or down-regulation or for an underlying systemic technical error. Subsequent analyses therefore focused on fold-change estimation between paired tumor-normal samples and reduction of data jittering by normalization to cell markers. Regarding the LC-MS branch of the workflow, 32 lysates (8 breast tumor, 8 breast healthy adjacent, 8 kidney tumor and 8 kidney healthy adjacent) were tryptically digested and measured by LC-MS using DDA at HUN-REN Research Centre for Natural Sciences, designated as LC-MS’ throughout the text. Selection for the downstream analyses was performed on matched patient pairs, not on individual lysates. Specifically, the DIA/PRM cohort consisted of four matched breast tumor-adjacent pairs and four matched kidney tumor-adjacent pairs (16 lysates total), corresponding to the top 50% of the initial DDA-screened lysates in terms of protein identifications. These samples underwent a second LC-MS analysis using DIA at another facility designated as LC-MS throughout the text. Subsequently, RPPA data (from all samples) and LC-MS data (from these same 16 lysates) were correlated by Spearman coefficient matrix analysis. Finally, top discordants (protein expression levels that exhibited the least correlation between RPPA and LC-MS methods deduced from Spearman correlation analysis) were re-evaluated using LC-MS PRM acquisition strategy, and data were re-correlated.

Importantly, all cross-platform comparisons were performed on identical biological material: each tissue specimen was lysed once, aliquoted, and the same lysate aliquots were used for RPPA and LC-MS (DDA/DIA/PRM). We adopted a tiered LC-MS strategy to balance depth and cost: an initial DDA screen on 32 lysates (LC-MS’) served to assess sample quality and to nominate the 16 lysates with the highest proteome coverage for subsequent deep DIA quantification (LC-MS), followed by targeted PRM re-measurement of the most discordant proteins on the same 16 samples. This design allows direct within-sample RPPA–MS fold-change comparisons while minimizing missingness due to sample quality.

Evaluation of proteins expression represented by the Ion AmpliSeq Cancer Hotspot Panel v2 and cell markers in tumor-normal adjacent human breast and kidney samples by RPPA.

**Fig. 2:**
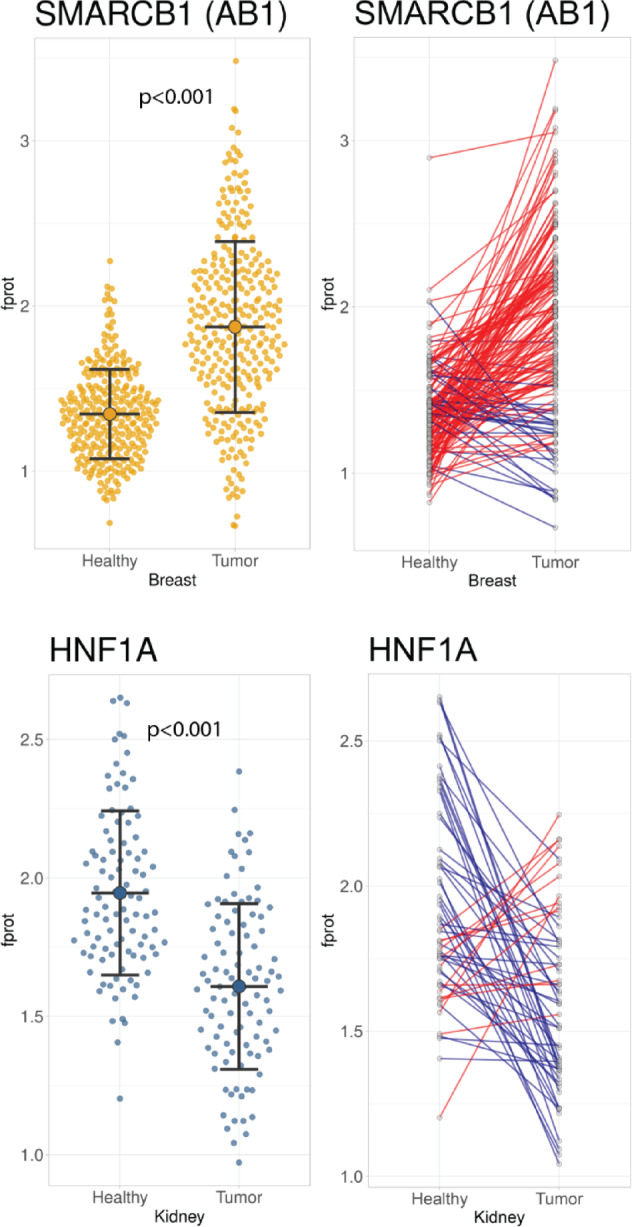
Quasi-random distribution of expression levels (fprot, determined by RPPA) of SMARCB1 (probed by antibody 1 (AB1) in breast tumor-normal adjacent samples) and HNF1A (in kidney tumor-normal adjacent samples). Each point represents one lysate-level fprot value calculated as the mean of all remaining normalized antibody spot values across the full five-point dilution series after matching each antibody spot to the fitted protein-stain (FCF) curve at the same dilution; therefore, no single dilution was plotted, nor do they represent propagated technical uncertainty from the upstream curve-fitting and normalization steps. Black lines indicate mean +/– 1 SD. In the right panels, the tumor-normal breast (top right) or kidney (bottom right) samples matching (connected by a line) from each same patient fprot are depicted; blue vs. red line indicates a decrease vs. an increase in fprot value (healthy as control).

For each sample, all valid normalized antibody spot values from the five-point dilution series were pooled after matching each antibody spot to the fitted FCF value at the same dilution, and the mean of the remaining normalized spots was taken as the sample-level fprot. Thus, the values plotted in Fig. 2 do not represent a single dilution point; they represent the aggregate signal from all valid technical spots of that lysate. Supplementary Fig. 1B shows a representative protein-stain dilution fit used in this normalization procedure. These sample-level fprot values were plotted quasi-randomly as shown on Fig. 2 in the top left panel for breast tumor-normal adjacent samples staining for SMARCB1 (using antibody AB1) and bottom left for kidney tumor-normal adjacent samples staining for HNF1A. Black lines signify mean and +/- 1*SD. Data parity (healthy-tumor samples from the same patient connected by a line) is shown in the corresponding panels to the right; blue vs. red line indicates a decrease vs. an increase in fprot value (healthy as control), respectively. Data for all breast or kidney samples staining all oncoproteins, tumor suppressor proteins and cell markers are shown in the supplementary Figs. 5–10. In some figures, extreme outliers have been arbitrarily removed (> 5 SD) so as to scale down the figure for better visual comparisons; however all data points are present in the corresponding data pair (tumor-normal adjacent from each patient) in the supplementary material. Importantly, all downstream RPPA analyses are based on derived point estimates rather than on fully error-calibrated sample-level quantities, and are interpreted as exploratory summaries of broad paired trends and cross-platform concordance rather than as precise effect-size estimates.

**Fig. 3:**
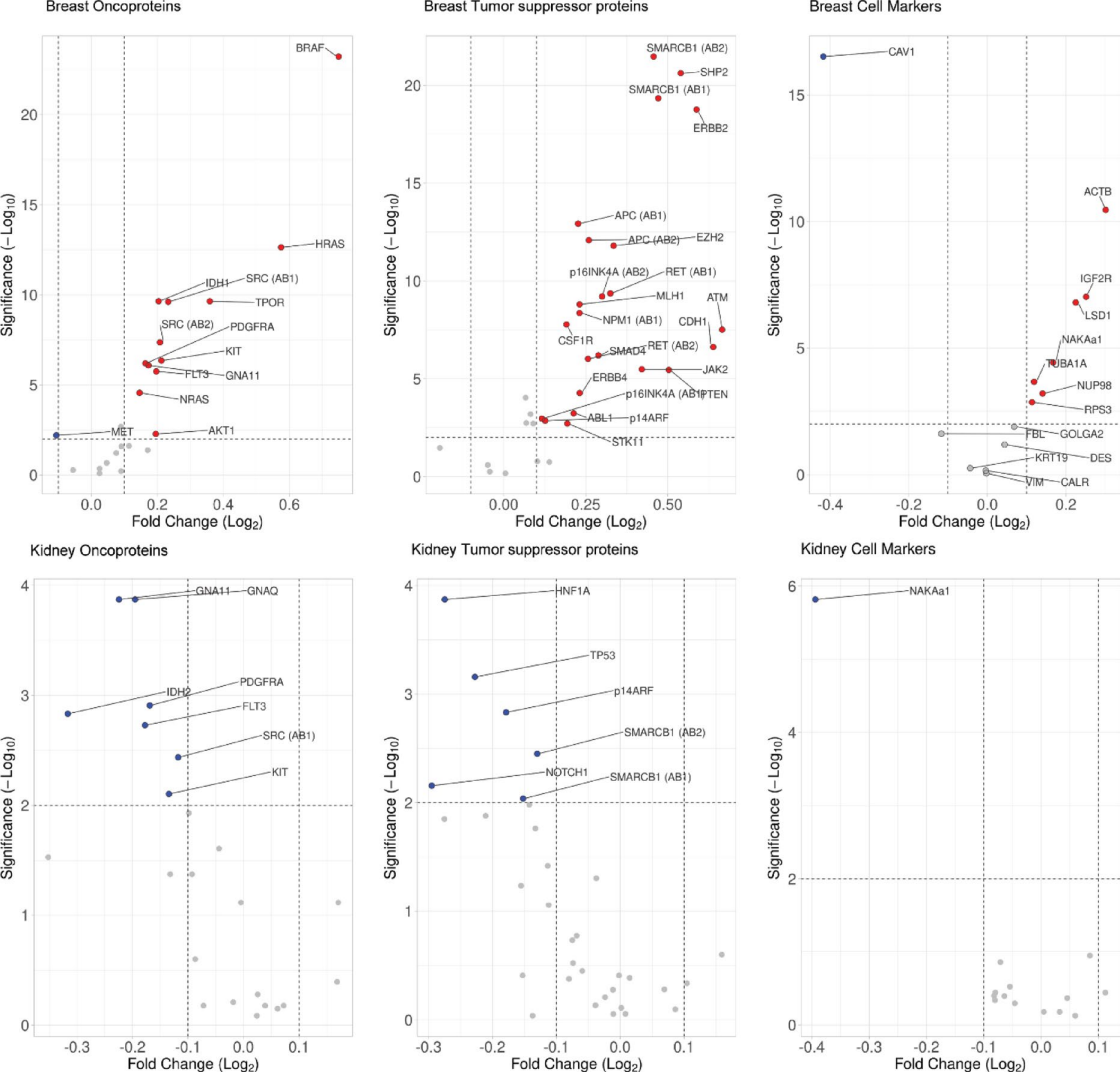
Volcano plots of breast (top row) and kidney (bottom row) oncoproteins, tumor suppressor proteins and cell markers depicting statistical significance versus magnitude of change, comparing matched tumor and adjacent normal tissues. Fold changes and paired t-tests were run on the complete breast and kidney datasets (determined by RPPA), followed by correction for multiple comparisons using the Benjamini-Hochberg method to adjust for false discovery rate. Cutoffs were set to *p*-adjusted < 0.05 and absolute log2 fold change > 0.1 for screening and visualization. These volcano plots are presented as exploratory summaries of direction and approximate magnitude. Because the underlying RPPA values are derived from curve-fitted and normalized measurements without formal propagation of upstream technical uncertainty, the plotted fold changes and adjusted p-values should be interpreted as heuristic ranking statistics within this study rather than as precision-calibrated effect-size estimates. The data was divided into oncoproteins, tumor suppressors, and cell markers for clearer visualization.

Statistical significance of protein expression as a function of fold change is depicted in the volcano plots in Fig. 3: proteins expressed in breast samples are shown in the top row and proteins expressed in kidney samples in the bottom row, subdivided into oncoproteins, tumor suppressor proteins and cell markers as indicated in the panels. Importantly, these plots summarize two separate within-organ comparisons -breast tumor versus matched breast normal-adjacent tissue and kidney tumor versus matched kidney normal-adjacent tissue. They therefore should not be interpreted as a direct breast-versus-kidney comparison or as evidence of a near-universal organ-level pattern of “mostly up in breast” and “mostly down in kidney”. Under Benjamini-Hochberg correction, only a limited subset of the panel reached significance at the cohort level. In breast samples, MET showed the clearest cohort-level decrease among the hotspot-panel proteins. Among the breast cell markers, CAV1 exhibited a marked decrease, whereas FBL, GOLGA2, KRT19, DES, VIM and CALR showed comparatively smaller cohort-level shifts and were therefore retained as candidate normalizers for exploratory analyses in this dataset. In kidney samples, the α subunit of the Na^+^/K^+^ ATPase (NAKAa1) showed the clearest cohort-level decrease, whereas the remaining cell markers showed smaller observed shifts. Thus, the predominant observation in Fig. 3 is not a coordinated organ-wide directional shift, but rather that only a minority of proteins met the multiple-testing threshold and that the observed changes were protein-specific. Several individual cell-marker changes reported in breast cancer -including increased ACTB, IGF2R, LSD1, NAKAa1, TUBA1A, NUP98 and RPS3, and decreased CAV1- are consistent with the directionality reported for some markers in prior studies. Specifically, pan-cancer analysis found significantly higher ACTB expression in breast tumor tissues compared to normal samples^28^. IGF2R is reported to be overexpressed in hormone receptor-negative breast tumors, especially in triple-negative breast cancers^29^. Basal-like and ER-negative breast cancers often exhibit high LSD1 levels^30^. A microarray study of breast cancers found NAKAa1 mRNA significantly overexpressed in tumors^31^. Gene expression profiling showed that TUBA1A is upregulated in breast carcinoma tissues compared to tumor-adjacent normal tissue^32^. Triple-negative breast cancers exhibit elevated NUP98 expression^33^. RPS3 is upregulated in breast tumors, contributing to tumor cell survival^34^. Numerous studies have found significantly lower CAV1 mRNA and protein levels in breast cancers compared to corresponding normal tissue^35^. However, these literature examples should not be extrapolated to a general breast-wide upregulation program in our dataset. Because our panel is intentionally enriched for cancer-relevant proteins and representative cell markers rather than constituting an unbiased proteome sample, and because the analyzed tissues were bulk, non-microdissected specimens, the observed tumor-normal-adjacent differences likely also reflect differences in cellular composition between breast and kidney tissues, including variable stromal contributions^36^. It is also notable that there is good agreement among almost all of the proteins against which two antibodies were used, exemplified by the close proximity of SMARCB1 (AB1) and SMARCB1 (AB2), APC (AB1) and APC (AB2), p16INK4A (AB1) and p16INK4A (AB2), and RET (AB1) and RET (AB2); other proteins against which two antibodies were used are not labeled in the volcano plots because they did not reach statistical significance (grey points). Finally, the observed differences in cell-marker expression between tumor and normal-adjacent tissues are broadly consistent with the macroscopic distinction made at tissue collection, although they do not substitute for histopathological or compositional assessment.

### Effect of cell‑marker normalization on RPPA clustering for cancer‑hotspot panel proteins

**Fig. 4:**
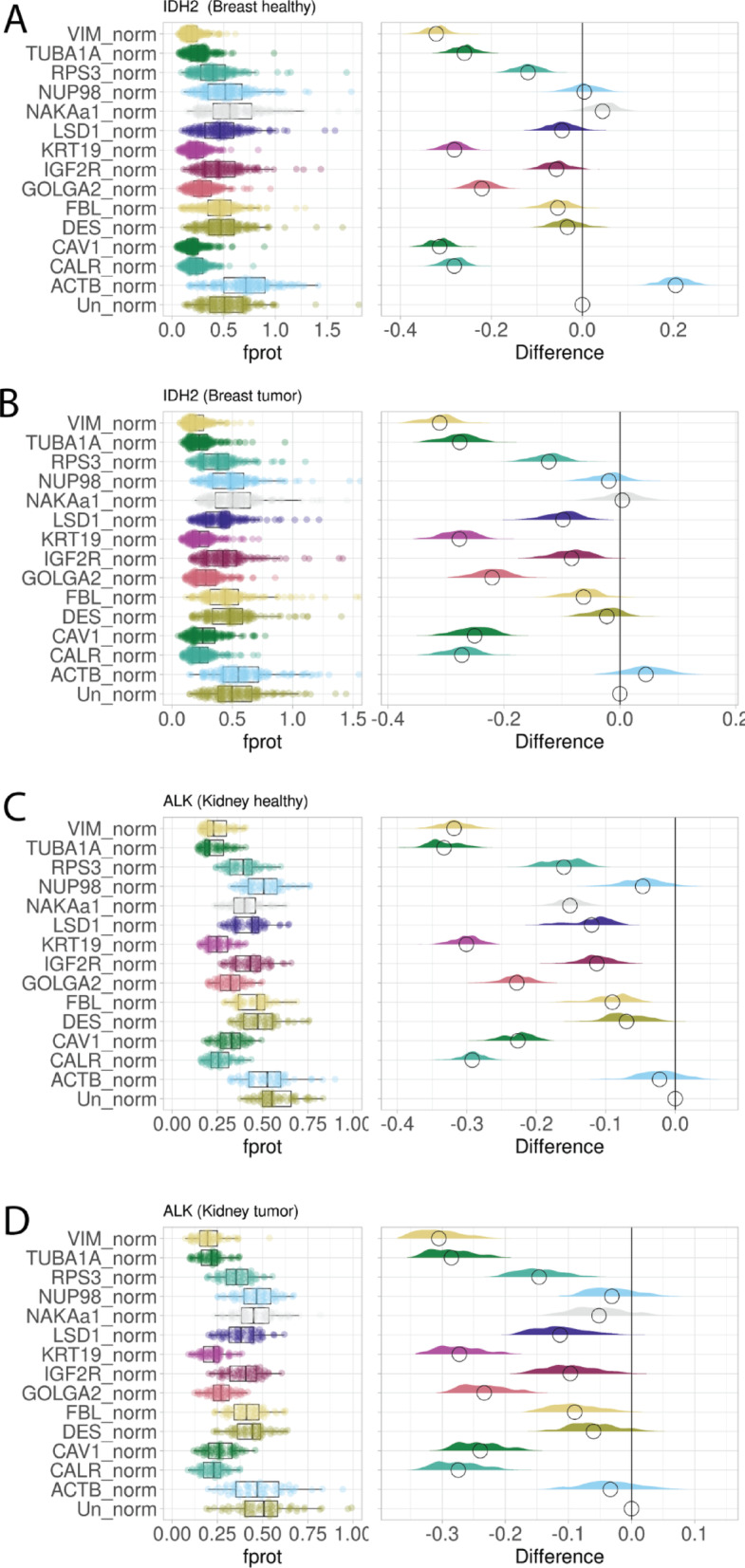
Jittered dot graphs showing RPPA data (fprot) of IDH2 (**A**, **B** breast healthy vs. tumor) or ALK (**C**, **D** kidney healthy vs. tumor, respectively) ratioed to the levels of cell markers expression, in a sample-matched manner. Each fprot value represents the lysate-level mean across all valid normalized spots from the five-point dilution series, summarizing the dispersion of the derived sample-level RPPA estimates after normalization to the indicated cell marker; they do not incorporate upstream uncertainty introduced by RPPA curve fitting, filtering, and protein-stain normalization. Un_norm: un-normalized to a cell marker. The summaries of the data are shown as a boxplot, with the box indicating the interquartile range and vertical line indicating the median. Plots on the right show the effect size, relative to Un_norm, which is the protein indicated on the top of each panel. The bootstrap samples that are used to calculate the 95%CI of the effect size are shown as a distribution.

In order to explore whether sample-matched normalization to cell markers reduced RPPA data spread, we ratioed the levels of cancer hotspot panel proteins to those of each cell marker. As shown in Fig. 4A and B for IDH2 in breast healthy vs. tumor samples, and in Fig. 4C and D for ALK in kidney healthy vs. tumor samples, several cell-marker normalizers produced visually tighter distributions than the un-normalized data. Normalization data for all other cancer hotspot panel proteins are shown in the supplementary dataset “Supplementary dataset RPPA normalizations to cell markers” in the supplementary material that can be downloaded from http://rppa.hu/suppl_mcp.html. In this dataset-specific exploratory screen, VIM most often yielded the greatest tightening of the RPPA distributions in both breast and kidney samples, whereas ACTB performed poorly in breast samples and DES provided little apparent improvement despite not differing significantly between breast healthy and tumor samples in Fig. 3. We emphasize that this screen was not intended as a formal hypothesis-testing exercise, and no multiple-testing correction was applied across the many RPPA normalizer comparisons. Accordingly, VIM is presented here as an empirical working normalizer for exploratory RPPA-LC-MS comparison in this dataset, not as a formally validated universal normalizer. The biological plausibility of relatively stable bulk VIM signal in our paired samples is consistent with the presence of VIM-expressing mesenchymal/stromal cells in both normal breast and kidney tissues^36^. Nevertheless, given the context dependence of VIM biology, our data support only the narrower conclusion that VIM was a practical descriptive choice in this cohort.

### Evaluation of protein expression in tumor-normal adjacent human breast and kidney samples using liquid chromatography–mass spectrometry

**Fig. 5:**
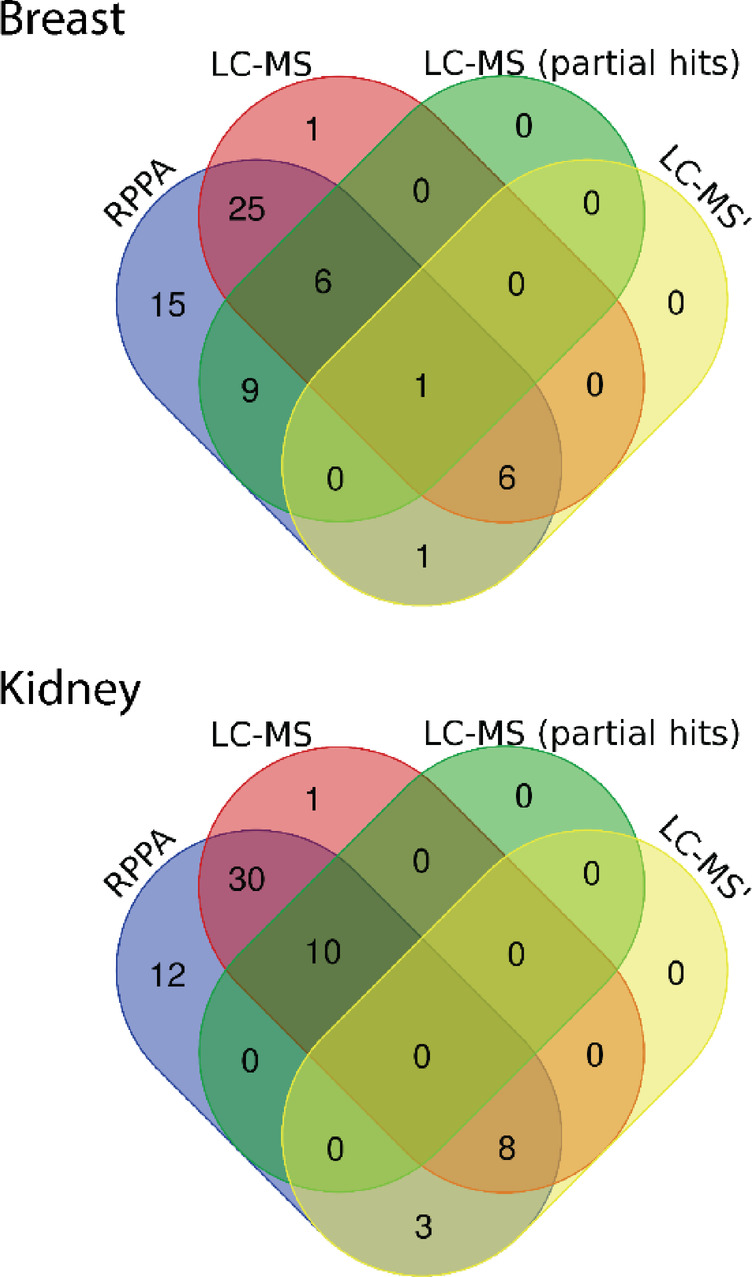
Venn diagrams entailing the number of proteins (from those listed in Supplementary Tables 2 and 3) identified through RPPA or LC-MS in 8 tumor-normal adjacent breast (top) or 8 kidney (bottom) samples. LC-MS (partial hits) is for a subset of proteins that were identified by some of the samples by LC-MS. LC-MS’ indicates the methodology and setup used to identify the top 50% of the lysates (those yielding the highest number of detected proteins) that were used for further, more advanced LC-MS.

To compare RPPA measurements with an independent analytical platform, we analyzed 32 randomly selected lysates (8 breast tumor, 8 adjacent normal breast, 8 kidney tumor, and 8 adjacent normal kidney) by DDA-LC-MS. Of the proteins listed in supplementary Tables 2 and 3, only a subset was identified by this workflow. The number of proteins detected by the different methods is shown in Fig. 5. The DDA screen was then used to select the 16 lysates with the highest proteome coverage for subsequent DIA-LC-MS analysis (4 breast tumor, 4 adjacent normal breast, 4 kidney tumor, and 4 adjacent normal kidney samples, representing 4 breast cancer and 4 kidney cancer patients). This design increased MS coverage for downstream comparison, but it also means that the direct RPPA-LC-MS comparison was limited to a selected subset of samples rather than the full cohort. Notably, this LC-MS workflow identified PIK3CA, for which no suitable antibody was available for RPPA, whereas ACTB could not be reliably distinguished from ACTG1 at the proteotypic peptide level because of their highly similar sequences.

### Comparison of protein expression in tumor-normal adjacent human breast and kidney samples assessed by RPPA vs. LC-MS

**Fig. 6:**
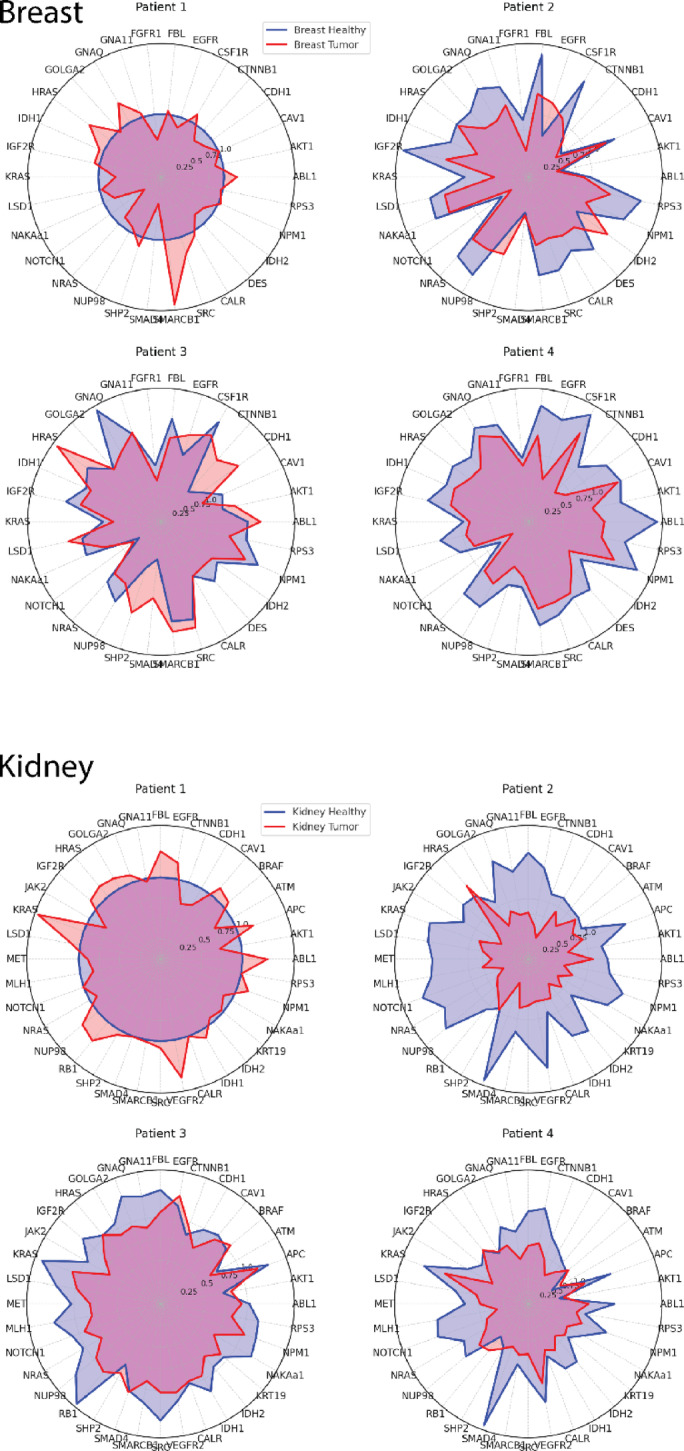
Radar charts for breast (top four) and kidney (bottom four) tumor-normal adjacent samples from 4 patients (patients 1–4 for breast tissues are different than patients 1–4 for kidney tissues). Each chart corresponds to a different patient specified on the top and each axis represents a different protein indicated on the chart, determined by RPPA and normalized to VIM expression in a sample-matched manner. Values are arbitrarily further normalized to healthy sample of patient 1 as “1.0” to facilitate comparison between healthy and tumor samples within each patient/tissue. Radial ticks represent relative expression levels (0.25, 0.5, 0.75, 1.0). Blue vs. red areas represent the expression levels of proteins in healthy vs. tumor samples, respectively.

As shown in the radar charts for breast (top four) and kidney (bottom four) tumor-normal adjacent samples in Fig. 6, the fold change between healthy and corresponding tumor-adjacent sample per protein, using RPPA values normalized to VIM, reveals a diverse proteome landscape per patient. Although only a limited number of proteins reached statistical significance at the cohort level under stringent multiple-testing correction (Fig. 3), the paired tumor/adjacent fold-change profiles across individual patients still provided sufficient variation for exploratory cross-platform comparison. We compared the data obtained from RPPA versus LC-MS in tumor-normal adjacent breast and kidney samples from the same lysates of 4 breast cancer and 4 kidney cancer patients. Because VIM most often reduced RPPA spread in the exploratory screen above, RPPA data are expressed as ratios to VIM unless otherwise indicated; this choice was made to facilitate descriptive comparison within the present dataset and should not be interpreted as proof that VIM is the optimal normalizer in other tissues or cohorts. For LC-MS, we considered four scaling schemes: (i) log2-transformed and ratioed to VIM, (ii) raw, ratioed to VIM, (iii) raw, and (iv) log2-transformed. (By ‘raw’, it is meant the sum of all fragment-ion intensities for all peptides assigned to a protein group, before any normalization or scaling.) Figure panels 7A-E show the example of ABL1, and the full results for all proteins are provided in the supplementary dataset “Supplementary dataset LC-MS and Spearman” that can be downloaded from http://rppa.hu/suppl_mcp.html. These analyses were intended as a sensitivity analysis of how cross-platform agreement depended on preprocessing choice. Accordingly, the Spearman coefficients comparing RPPA with the four LC-MS scaling schemes are presented descriptively, and no claim of a universally optimal cross-platform normalization strategy is made.

**Fig. 7:**
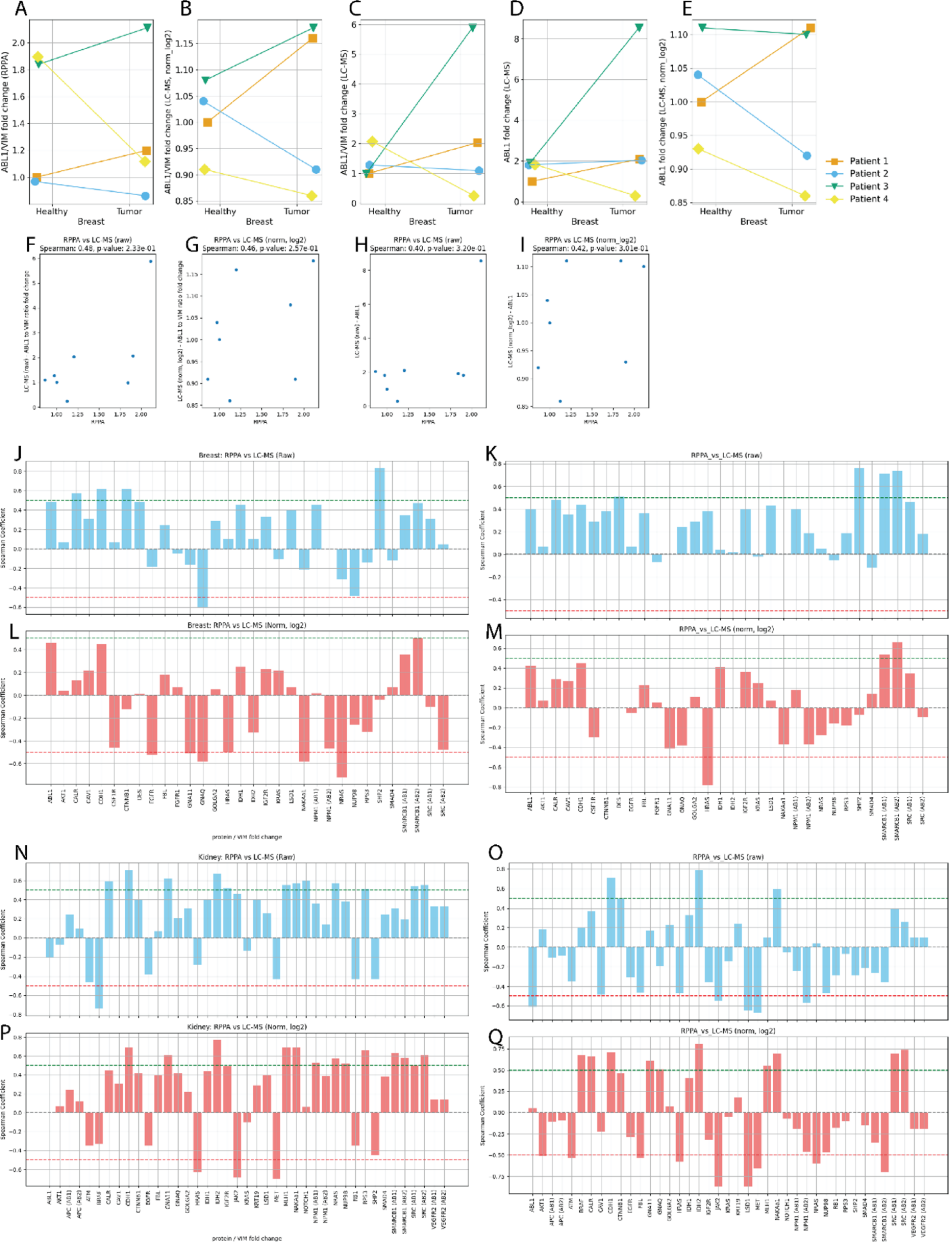
(**A**)–(**E**): Comparison of ABL1 expression between 8 tumor-normal adjacent breast samples from 4 patients estimated by RPPA or LC-MS under various normalization/scaling schemes, as indicated on the y-axis of each panel. (**F**)–(**I**): Spearman correlation coefficients for pairwise comparisons of the above data obtained by RPPA or LC-MS for ABL1 expression. (**J**)–(**Q**): Bar graphs of Spearman correlation coefficients for pairwise comparisons of all proteins estimated by RPPA and LC-MS, indicated on the x-axes. Green and red dashed lines show correlation coefficients of 0.5 and − 0.5, respectively. The normalization/scaling comparisons are shown as an exploratory sensitivity analysis of preprocessing choice and were not treated as a formal multiple-testing-corrected model-selection exercise.

Formal cross-platform correlation analyses in this study were restricted to RPPA and LC-MS datasets (DDA, DIA and PRM) generated from the same lysates. Western blotting was not included in these matrices because its primary role here was antibody validation for RPPA, including verification of predominantly single-band behavior, rather than functioning as a third quantitative platform.

### Correlation of Spearman coefficients in tumor-normal adjacent human breast and kidney samples assessed by RPPA and LC-MS

**Fig. 8:**
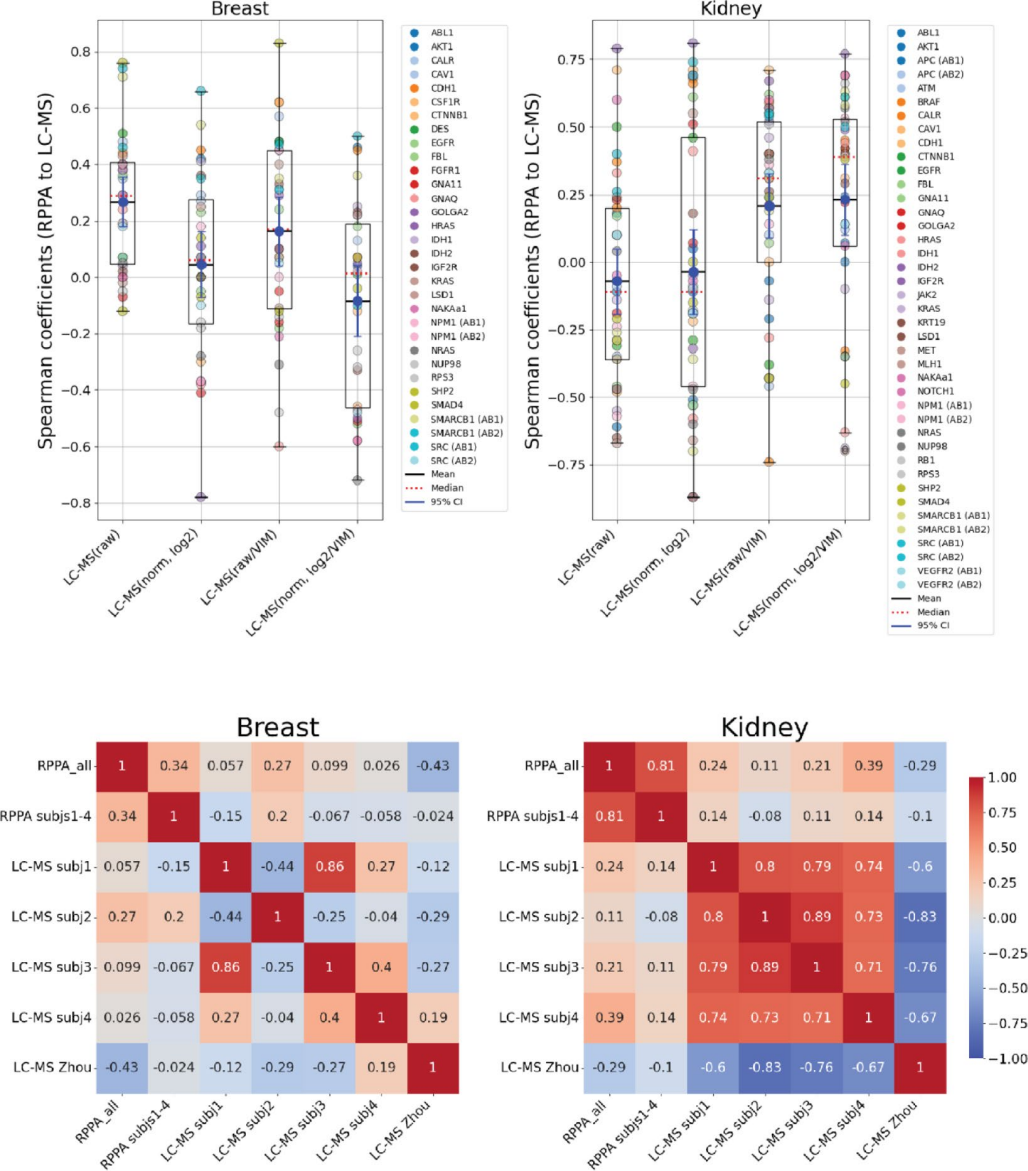
Top row: Correlation of Spearman coefficients between RPPA and LC-MS data analysis methods. Data obtained from breast vs. kidney tissues are shown in the left vs. right panel, respectively. Each colored point corresponds to the specific proteins listed in the legend. The boxes represent the interquartile range of the Spearman correlation coefficients for each method comparison. The mean, median and 95% CI are indicated by a black solid, red dotted and blue solid line, respectively. RPPA data have been normalized to expression of VIM. LC-MS data are expressed as: raw (raw), normalized and log2 transformed (norm, log2), raw and normalized to expression of VIM (raw/VIM) and normalized and log2 transformed and normalized to expression of VIM (norm, log2/VIM). The Spearman coefficients were calculated from the fold change of a healthy- to its tumor-adjacent sample obtained from the 4 patients that were evaluated by both RPPA and LC-MS. These method comparisons are descriptive and were used to illustrate the sensitivity of cross-platform concordance to preprocessing choices; they were not interpreted as a multiple-testing-corrected selection of an optimal normalization strategy. Bottom row: Spearman rank correlation matrix showing the pairwise correlations between RPPA (ratioed to VIM, fold change healthy/tumor, all matched breast or kidney samples as indicated in the panels), LC-MS from this study (per patient indicated as subj1, 2, 3, or 4), and LC-MS data from Zhou et al.^37^, for the subset of proteins quantified in the RPPA and LC-MS datasets being compared. Western blot data were not included in these matrices because the blots were used primarily for antibody validation of RPPA rather than as a third quantitative platform.

As shown in Fig. 8 (top row) for breast (left) and kidney (right), the Spearman coefficients calculated from the fold change (as a function of method and normalization) of a healthy- to its tumor-adjacent sample obtained from the 4 patients that were evaluated by both RPPA and LC-MS for each protein (color-coded in the legend) are plotted. The boxes represent the interquartile range of the coefficients for each method comparison, with the mean, median and 95% CI indicated by a black solid, red dotted and blue solid line, respectively. Within this small exploratory dataset, the mean/median coefficients were highest for RPPA versus LC-MS raw in breast and for RPPA versus LC-MS normalized, log2-transformed, and ratioed to VIM in kidney. We therefore used those pairings for the descriptive correlation matrices shown below. However, because these pairings were identified after screening multiple RPPA/LC-MS normalization combinations in a small sample, and without multiple-testing correction across that screen, they should be interpreted as dataset-specific display choices rather than as definitive optimal settings. As shown in the bottom heatmap panels of Fig. 8, we included not only RPPA data from all samples (RPPA_all) but also nested those from the 4 patients tested by both RPPA and LC-MS, and included data from the study by Zhou et al.^37^, where tumor-normal adjacent human breast and kidney samples were also investigated. For the Zhou et al. data^37^, we obtained the fold change of healthy to tumor-adjacent samples for commonly detected proteins between our study and theirs. These proteins included ABL1, AKT1, ATM, BRAF, CDH1, CDKN2A, CTNNB1, EGFR, ERBB2, GNA11, GNAQ, HRAS, IDH1, IDH2, JAK3, KRAS, MET, NOTCH1, NPM1, NRAS, SMAD4, SMARCB1, SRC, CALR, CAV1, DES, FBL, GOLGA2, IGF2R, KRT19, NUP98, RPS3, and VIM. This matrix highlights the general discord between RPPA and LC-MS data rather than establishing a preferred universal normalization strategy.

### Further evaluation of protein expression using parallel reaction monitoring (PRM) mode of LC-MS and re-correlation to RPPA data

**Fig. 9:**
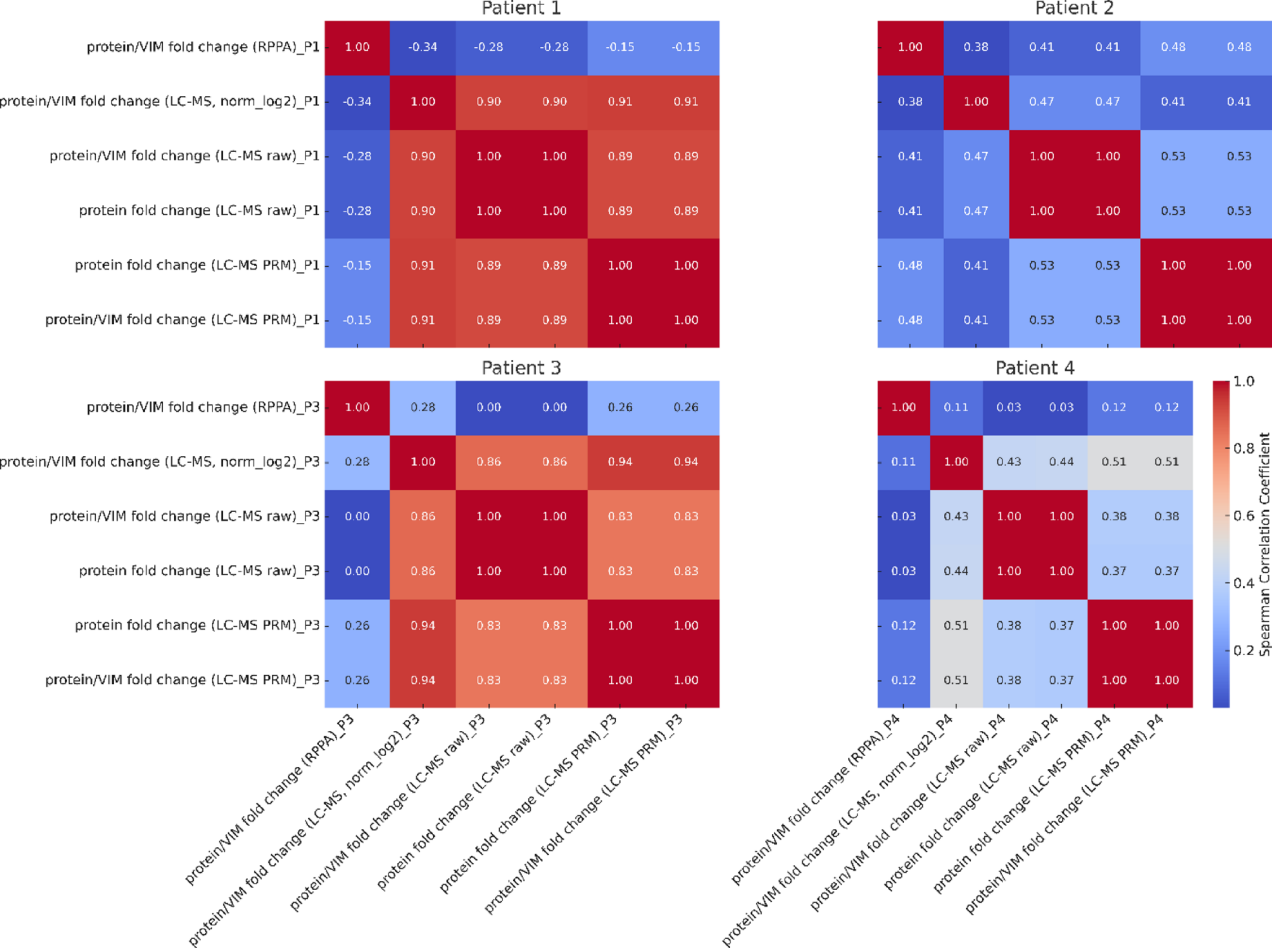
Spearman correlation heatmaps for breast tumor-matched samples. Each panel represents the correlation coefficients between different methods used to measure protein expression levels (tumor/healthy ratios) in breast tissue samples from four patients (P1-4). The color bar on the bottom right heatmap indicates the range of Spearman correlation coefficients, where 1.0 represents a perfect positive correlation and − 1.0 represents a perfect negative correlation.

Because several proteins showed low or negative RPPA-DIA correlations, we re-measured selected targets in the same samples by targeted PRM-LC-MS. The proteins chosen for PRM were those that showed the greatest disagreement between RPPA and DIA-LC-MS, together with the cell markers. The peptide fragments used for PRM are listed in the supplementary files Breast_PRM.xlsx and Kidney_PRM.xlsx. As shown in Fig. 9 for breast samples and Supplementary Fig. 11 for kidney samples, PRM modestly improved cross-platform agreement for some proteins, but discrepancies persisted for others. Thus, targeted MS reduced disagreement in selected cases but did not make the RPPA and LC-MS measurements interchangeable across the panel.

To further document antibody suitability for RPPA and to provide a qualitative check on selected targets, we performed Western blotting for representative proteins using tumor and tumor-adjacent samples from the same lysate aliquots analyzed by RPPA and LC-MS (Supplementary Fig. 12; full antibody-validation blots are available through our online repository). The primary purpose of these blots was to verify that the antibodies used in RPPA yielded predominantly single bands at the expected molecular weight under our assay conditions, thereby supporting their suitability for the dot-based RPPA format, where band resolution is not available. We included examples of proteins showing both closer and weaker RPPA–MS agreement, but the blots were not intended to serve as a third quantitative platform or as formal orthogonal validation of all cross-platform differences. Because Western blotting can accommodate only a limited number of samples per run, these data are presented as qualitative antibody-validation and illustrative support rather than as the basis for quantitative correlation estimates.

### Summary of cross-platform concordance between RPPA and LC-MS

**Fig. 10:**
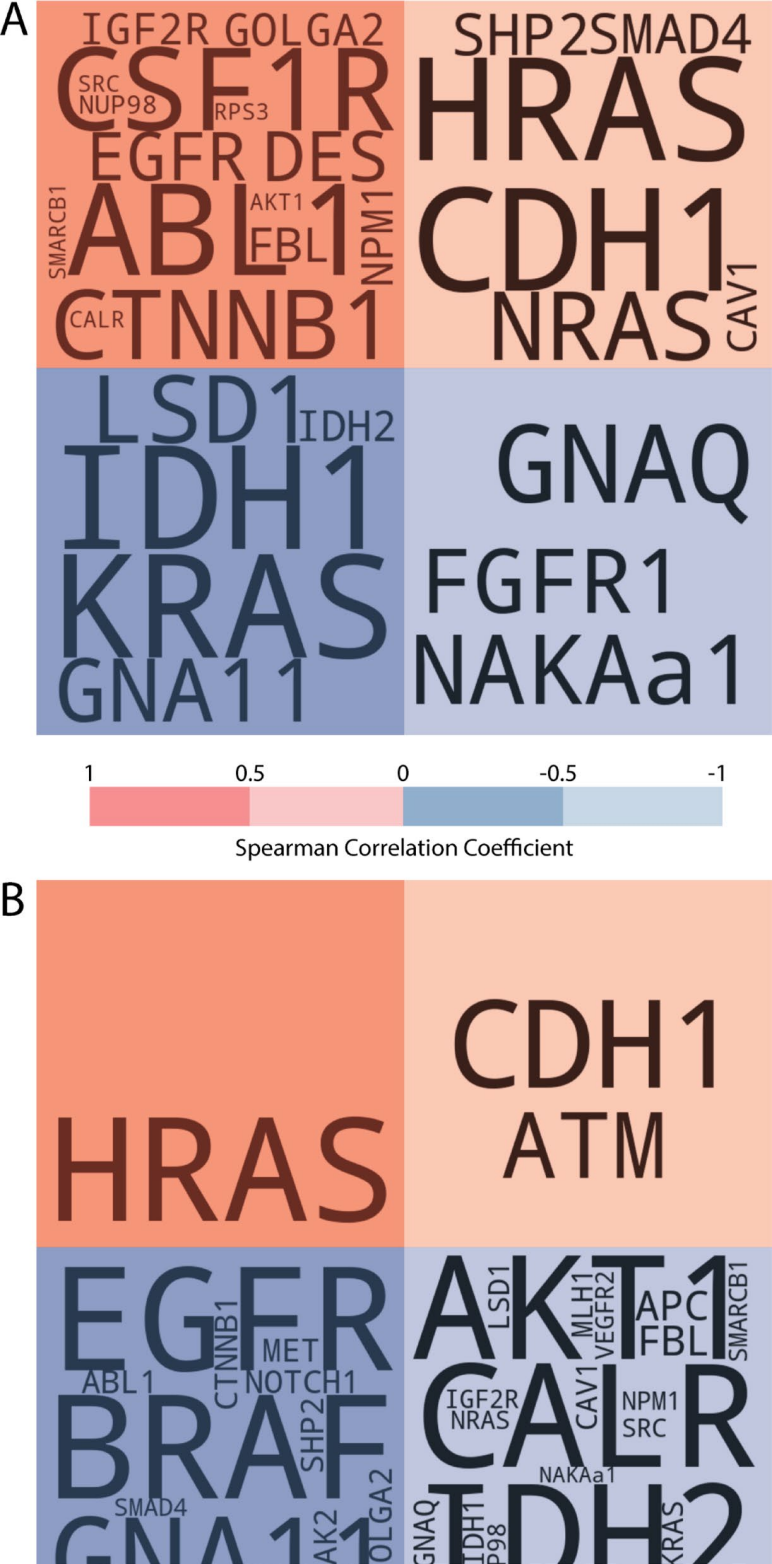
Word clouds of protein expression levels in breast (**A**) and kidney (**B**) tumor to healthy ratio, assessed by RPPA normalized to VIM and LC-MS DIA raw ratioed to VIM, which was used here as a dataset-specific comparison setting for exploratory cross-platform summary. Protein names are scaled by their Spearman correlation coefficients, with font size reflecting the strength of correlation within their own quadrant; quadrant ranges of the coefficients are indicated in the color bar.

After evaluating protein expression by RPPA and LC-MS across multiple LC-MS acquisition modes (DDA, DIA, PRM) and several normalization/scaling options, we do not interpret the present screen as establishing a single best workflow for future studies. Instead, Fig. 10 summarizes concordance patterns using one dataset-specific comparison setting (RPPA normalized to VIM and LC-MS DIA raw ratioed to VIM) chosen for descriptive synthesis across tissues. Because this setting emerged from an exploratory screen conducted on only four matched tumor-normal pairs per tissue type, without multiple-testing correction across the normalization/scaling comparisons, it should be regarded as an empirical analytical choice for this manuscript rather than as a validated universal recommendation. In breast tissues, 15 proteins fell within a Spearman correlation range of 0.5–1, whereas three proteins (GNAQ, FGFR1 and NAKAa1) showed strong negative correlations. Five proteins (IDH1, IDH2, LSD1, KRAS and GNA11) exhibited weak negative correlations, and six proteins (SHP2, SMAD4, HRAS, CDH1, NRAS and CAV1) exhibited weak positive correlations. These patterns highlight analytes for which cross-platform agreement was relatively better or worse in this exploratory comparison, but they should not be used as the sole basis for recommending or excluding specific proteins from future studies. Qualitatively and quantitatively different patterns were also observed in kidney tumor-normal adjacent samples, depicted in Fig. 10B.

## Discussion

Our same-lysate cross-platform comparison revealed that correlations between RPPA and the LC-MS acquisition modes (DDA, DIA, and PRM) were highly variable. Some proteins showed concordant tumor/adjacent-normal fold changes across platforms, but agreement was often only moderate and in several cases absent or inverse. PRM modestly improved alignment for selected targets, yet substantial discrepancies remained. We therefore do not interpret these data as showing that discordance itself yields biological insight. Rather, the main finding is that RPPA and LC-MS provide partial, protein-dependent concordance when applied to the same lysates. This is consistent with the fact that the two methods are not analytically equivalent: RPPA measures an antibody-recognized epitope, whereas LC-MS infers protein abundance from one or more peptides after digestion. The practical implication is that the two platforms should not be treated as interchangeable for targeted quantification of all proteins in this panel. Similar variability in inter-method agreement has been noted by other investigators comparing proteomic platforms^38^.

An important point is that our screen of multiple cell-marker normalizers and LC-MS scaling schemes was exploratory. It was intended to assess how sensitive RPPA-LC-MS concordance was to preprocessing choice, not to perform formal inference over competing normalizers. Accordingly, although VIM often yielded the tightest RPPA distributions in this cohort, and certain LC-MS scalings produced numerically higher correlations in specific tissues, these rankings may partly reflect chance in a small sample and should not be interpreted as universal optimization results. Multiple-testing correction was applied to formal differential-expression analyses, but not across the exploratory normalization/scaling screen. This caution is especially warranted given the known variability in agreement across proteomic platforms^38,39^.

Several factors contribute to the incomplete correlations between RPPA and LC-MS. First, biological heterogeneity can play a role. Cancer tissues are inherently heterogeneous, and even matched tumor and adjacent-normal specimens exhibit patient-specific differences in protein expression. We used identical lysate aliquots for each sample across all platforms (ensuring no differences in the sampled tumor region between methods) and we minimized technical biases by standardized processing and normalization (for example, using IR-based total protein quantitation and normalizing signals to housekeeping proteins). Nevertheless, inter-patient variability and intra-tumor heterogeneity remain unavoidable. A few outlier patients can weaken overall correlations – one method might detect a large change in a given protein for a particular patient while the other method does not—thereby skewing the concordance when data are aggregated.

Beyond biological variation, fundamental technical differences between the two platforms introduce biases that can drive method disagreement. RPPA is an antibody-based assay, thus, its performance is at the mercy of antibody specificity and affinity. If an antibody exhibits off-target cross-reactivity or recognizes multiple isoforms of a protein, the RPPA readout may represent a composite signal. In such cases, RPPA could report an apparent abundance change that LC-MS does not see, or vice versa. In our study we selected antibodies with as much monospecificity as possible, yet even a well-validated antibody may have subtle cross-reactivity or be affected by differences in epitope availability. By contrast, LC-MS is not limited by antibodies and in theory can detect any peptide from any protein; however, MS-based quantification has its own constraints. A known limitation is sensitivity and ionization bias—peptides vary widely in their ionization efficiency, meaning a low-abundance protein might go undetected or unquantified by MS even if it is present, especially in DDA mode. RPPA, which benefits from signal amplification through antibody binding and highly sensitive detection, might successfully measure such a low-abundance protein, yielding a discrepancy between the methods. Conversely, for very abundant proteins, an MS detector can become saturated, whereas RPPA signals from lysates sampled in many replicates and dilutions (as it is the case in the present study) remain in the measuring range.

Another important consideration is the detection of protein variants, such as tumor-specific mutations. A mutated protein can be essentially invisible to a conventional MS analysis if the mutation creates a peptide sequence that is not present in the reference database—standard proteomic search pipelines will simply not identify a novel peptide arising from that mutation. Unless a proteogenomic approach is used to include patient-specific variant sequences in the MS database, the LC-MS data will reflect only the wild-type protein. An RPPA antibody, however, will still bind to the protein as long as the mutation is outside of the antibody’s epitope. Consequently, a mutant protein might be quantified by RPPA but entirely missed by MS, causing a major discrepancy in cases where a tumor harbors an abundant mutant form of a protein. Conversely, if a mutation occurs within the antibody’s binding epitope (or induces a conformational change that masks that site), the RPPA could under-report or fail to detect the protein, whereas MS might still detect and quantify peptides from other parts of the protein sequence. We did not specifically test for such mutations in our samples; this is a limitation of our study, since without DNA sequencing data or mutant-specific antibodies we cannot determine whether some of the RPPA–MS discordances were due to unrecognized mutant proteins in the tumors.

Post-translational modifications (PTMs) add another layer of complexity. RPPA in our study was designed to measure total protein levels, so it would not depict changes in protein activation state if the total amount of the protein remained unchanged. For instance, considering a signaling protein that is activated in tumors by phosphorylation, MS might detect an increase in a specific phosphopeptide even if the total protein quantity is constant, leading MS to indicate an increase while RPPA (tracking total protein) shows no change. Alternatively, MS may not be able to reliably detect a heavily modified protein by glycosylation/acetylation/sumoylation/ubiquitination/multi-site phosphorylation through altered proteolytic cleavage or ionization efficiency. Indeed, this was the case for the six proteins that exhibited the most negative correlation between RPPA and LC-MS (FGFR1, GNAQ, NAKAa1, AKT1, CALR, IDH2), as all harbor a high number of ubiquitinated lysines and phosphorylation sites. Specifically, FGFR1 is heavily glycosylated (8 N-glycosylation sites) and contains > 10 phosphorylation sites and 5 ubiquitinated lysines; GNAQ has > 10 ubiquitinated lysines and 2 phosphorylation sites; NAKAa1 possesses > 20 ubiquitinated lysines and > 30 phosphorylation sites; AKT1 includes 10 ubiquitinated lysines, > 10 phosphorylation sites, 4 O-linked glycosylation sites, 2 acetyllysines, and 5 sumoylated lysines; CALR features > 20 ubiquitinated lysines, 15 acetyllysines, 5 phosphorylation sites, and 1 N-glycosylation site; and IDH2 carries > 20 ubiquitinated lysines, 4 phosphorylation sites, and 6 acetyllysines. An antibody that recognizes an unmodified epitope might not bind the modified form (if the modification occurs at or near the epitope), meaning that RPPA could selectively measure either the modified or the unmodified pool, depending on the antibody’s specificity. In our panel, most antibodies targeted total protein rather than specific PTMs, so a protein that was differentially modified in tumors might show no change by RPPA but a change by MS (if MS detected a modified peptide), or vice versa. Additionally, MS data – particularly in DIA mode—suffers from spectral interference (chimeric spectra), where multiple co-eluting peptides fragment simultaneously and complicate quantification. This interference causes MS to over- or underestimate a protein’s abundance, an issue not encountered with RPPA. Such DIA-specific artifacts may have contributed to some of the variance we observed between DIA and RPPA results; for example, if two peptides from different proteins share fragment ions, the DIA analysis might erroneously inflate the signal for one protein, producing an outlier that does not align with the RPPA data. In our analysis, we employed modern data-processing tools to mitigate interference, but it remains a known limitation of DIA. In light of these factors, discordance between methods should not be interpreted as a direct biological signal. Rather, it is compatible with the distinct analytical biases and molecular surrogates of RPPA and LC-MS. In the present study, such discordance therefore serves primarily as a cautionary indicator that additional targeted follow-up is required before a protein can be advanced as a robust biomarker. Indeed, comparable inconsistencies between different proteomic technologies have been documented in other studies^39^, indicating that this is a general challenge.

On the basis of our results, the most defensible value of orthogonal analysis in this setting is confirmatory rather than additive. When RPPA and LC-MS converge on the direction of change for a given protein in the same lysates, confidence in that candidate increases. When they diverge, the present data do not justify assigning biological meaning to the disagreement itself; instead, such proteins should be considered unresolved and prioritized for further targeted validation, ideally in larger and clinically better-annotated cohorts. In this sense, cross-platform comparison helps distinguish comparatively robust signals from method-sensitive ones, which is directly relevant to biomarker triage.

Finally, we acknowledge several methodological limitations in this study and in the platforms employed, which also point to areas for improvement. First, our LC-MS analyses did not identify every protein that was on the RPPA panel – incomplete proteome coverage is an inherent limitation of shotgun MS. Some proteins were not detected by MS in DDA mode at all (likely because their abundance was below the MS detection threshold), and even DIA, with its greater depth of coverage, can miss proteins that have very few tryptic peptides or that ionize poorly. Even when a protein was detected by MS, the accuracy of its quantification could be affected by the issues discussed earlier (such as peptide ionization differences and interference from co-eluting species). In particular, DIA data can suffer from chimeric spectra that complicate accurate quantitation. We employed computational tools to reduce interference in the DIA data, but this issue cannot be entirely eliminated, especially when analyzing complex tissue lysates.

On the RPPA side, a major limitation is the dependence on antibody quality and availability. We could only measure proteins for which a high-specificity, validated antibody was available. For instance, the oncogenic protein PIK3CA was not included in our RPPA analysis because we were unable to obtain a suitable antibody, even though PIK3CA was readily detected by MS. Moreover, RPPA generally provides no information about protein isoforms or specific mutations unless one uses specialized antibodies. As mentioned above, without mutant-specific antibodies, RPPA cannot distinguish a mutant protein from its wild-type counterpart if both are present in the sample – it will report a combined signal. In our study, we attempted to choose antibody target epitopes that were in regions of the protein less likely to harbor common mutations (so as to reduce the chance that a prevalent mutation would disrupt antibody binding), but this was not always feasible. We also note that our MS pipeline did not include a custom search for mutant peptides, so in this regard both platforms share a limitation: a mutated protein could manifest as a discrepancy between RPPA and MS if, for example, the mutation abrogated the antibody binding (making RPPA underestimate the protein) while the corresponding novel peptide escaped identification in the MS data.

Another practical limitation involves throughput and sample selection. RPPA is a high-throughput technique that can rapidly process hundreds of samples at relatively low cost, whereas comprehensive LC-MS profiling is slower, resource-intensive, and much more expensive per sample. Indicatively, inquiries into state-of-the-art commercial platforms for synthesizing heavy isotope-labeled peptides for subsequent use in LC-MS PRM mode—the most reliable and sensitive method—revealed a cost of eighty-two thousand euros for sixteen samples, the number utilized in this study. Such pricing could not be incorporated into the standard of care for cancer patients. Due to these constraints, we analyzed only a subset of the total sample set by LC-MS. We selected samples with sufficient high-quality lysate for MS analysis, which means the MS cohort may have been biased towards better-quality or higher-yield specimens. While this approach was necessary for practical reasons, it could potentially skew the comparison if, for example, higher-quality samples also tended to have certain proteomic characteristics. The cost and throughput differences between RPPA and MS partly explain why large clinical studies typically choose one platform or the other, and why direct cross-platform comparisons (like the one we undertook) are relatively uncommon. One consequence is that platform-specific biases often go unrecognized in single-platform studies, underscoring the usefulness of direct cross-platform benchmarking when prioritizing candidate biomarkers.

The insights from our study suggest several directions for methodological improvement. On the RPPA front, a clear need is the development of more specific and diverse antibodies. For example, mutant-specific antibodies (that recognize only the oncogenic mutant form of a protein and not the wild-type) would allow direct measurement of cancer-driving protein variants, removing ambiguity about their presence. Likewise, isoform-specific antibodies that target unique regions of a particular isoform could enable researchers to distinguish between closely related proteins (such as different RAS isoforms) in tumor samples. Additionally, expanding the repertoire of high-quality antibodies—especially for proteins that are currently difficult to probe – is important. Some proteins remain challenging to measure via RPPA because no high-affinity, highly specific antibody exists for them. Investments in antibody technology (for instance, generating recombinant monoclonal antibodies or using phage-display libraries to discover binders for traditionally “undruggable” targets) and improved signal amplification strategies on the array could together enhance RPPA’s sensitivity. These advances would make it possible to reliably quantify even lower-abundance proteins and to detect specific PTM states, thus narrowing the gap between what RPPA and MS can measure.

For MS-based proteomics, improving sensitivity and throughput is key to bridging the divide with antibody-based assays. One promising avenue is the emergence of new mass spectrometry platforms with faster scanning speeds and higher ion utilization efficiency. Instruments that can acquire spectra at a high frequency or accumulate ions in parallel (for example, using parallel reaction monitoring or trapped ion mobility separation) enable the use of narrower isolation windows in DIA, which reduces co-fragmentation, while still collecting enough data points across each chromatographic peak for accurate quantification. Techniques like ion mobility-integrated DIA (e.g., the DIA-PASEF method) add an additional dimension of separation, allowing many more precursors to be analyzed concurrently without sacrificing signal quality. At the same time, ongoing improvements in computational analysis – including machine learning-assisted algorithms for deconvoluting complex DIA spectra—are helping to mitigate interference and extract more accurate quantitative information from MS data. Implementing these technological advances should make untargeted MS quantification approach the fidelity of targeted assays over time. On the targeted MS side (PRM/SRM), while these methods already offer very accurate quantification for dozens of targets, their multiplexing capacity is limited. An area of active development is increasing the number of proteins that can be quantified in a single run without loss of sensitivity. If, for instance, PRM could be scaled to monitor hundreds of peptides with high precision in one experiment, it would become feasible to routinely validate large panels of candidate biomarkers across extensive patient cohorts as a follow-up to discovery studies.

Finally, future studies may benefit from a sequential cross-validation workflow rather than from assuming that the union of discordant measurements necessarily yields a more accurate biological answer, in line with the field which is moving toward more integrative proteomic approaches^40^. Untargeted LC-MS can nominate candidate proteins or pathways, whereas RPPA or targeted MS can test whether those candidates reproduce across an independent analytical principle and across larger cohorts. Our data support this more conservative framework because only a subset of proteins behaved consistently across platforms. Accordingly, combining platforms is most useful here as a strategy for confirmation and prioritization, not as evidence that discordant measurements are intrinsically informative.

In summary, benchmarking RPPA against LC-MS on same tissue lysates showed partial, protein-dependent concordance and substantial discordance for other targets. The platforms are therefore not interchangeable for quantifying all proteins in this panel, and opposite results across methods should not be over-interpreted as biological insight. The practical value of orthogonal proteomic analysis in our dataset is instead to confirm a subset of candidate biomarkers across independent measurement principles and to flag others for cautious interpretation and further validation.

## Limitations of the study

An important limitation of the study is that the samples that we used have not been probed for mutations of the respective genes. An additional limitation is that we have no patient history, no tumor grading or subtyping of any kind, nor tumor percentage in the ‘tumor samples’. Furthermore, considering that RPPA is an antibody-based assay and we applied that to proteins that possibly harbor a number of mutations, it is a risk that some of these mutations would exert unknown effects on immunogenicity (and thus RPPA output signals). To this end, we have included in our antibody selection criteria the use of epitopes away from high mutagenic areas, although this was not always possible. Finally, we acknowledge that our LC-MS validation was performed on a relatively small subset of samples, which imposes important limitations on the study. Specifically, only half of the sample lysates (the top 50%) were selected for advanced DIA/PRM LC-MS analysis, thus, this was done in only four matched tumor-normal pairs per tissue type. As a result, any cross-platform comparisons between RPPA and LC-MS (such as Spearman correlation coefficients estimated here) are based on a very limited sample size, making these correlations statistically tenuous and highly susceptible to outlier effects. Consequently, the resulting correlation heatmaps and protein scoring should be interpreted with caution given the reduced confidence at this small n. In addition, the comparison of multiple RPPA cell-marker normalizers and LC-MS scaling schemes was exploratory and no multiple-testing correction was applied across that screen. Therefore, the ranking of VIM and of the LC-MS scaling options may partly reflect chance, and these settings should be viewed as dataset-specific analytical choices rather than validated optimal normalizers. This limited LC-MS validation cohort was primarily due to practical constraints, as comprehensive LC-MS proteomic analysis (especially with advanced DIA/PRM workflows) is substantially more expensive and resource-intensive per sample than the high-throughput RPPA platform, which inherently restricted the number of samples we could feasibly analyze by LC-MS.

An additional analytical limitation concerns uncertainty propagation in the RPPA workflow. The RPPA sample-level values used throughout this study are not direct single-spot measurements but derived quantities obtained from multiple technical spots across five dilutions after background correction, curve fitting to the FCF series, exclusion of low-intensity and outlier spots, and normalization, with additional normalization to VIM in some analyses. Although this strategy reduces single-spot noise, it also compounds uncertainty across sequential processing steps. Because this uncertainty was not formally propagated into sample-level error estimates in the present study, small fold changes and marginal differences should not be over-interpreted. Future studies should incorporate formal propagation-of-error or resampling-based uncertainty estimation for these derived RPPA quantities.

## Supplementary Information

Below is the link to the electronic supplementary material.

Supplementary Material 1

Supplementary Material 2

Supplementary Material 3

Supplementary Material 4

Supplementary Material 5

Supplementary Material 6

Supplementary Material 7

Supplementary Material 8

Supplementary Material 9

Supplementary Material 10

Supplementary Material 11

Supplementary Material 12

Supplementary Material 13

Supplementary Material 14

Supplementary Material 15

Supplementary Material 16

Supplementary Material 17

Supplementary Material 18

Supplementary Material 19

Supplementary Material 20

Supplementary Material 21

Supplementary Material 22

Supplementary Material 23

## Data Availability

Unless provided in the supplementary material or accessible through web links specified in the text, the datasets used and/or analysed during the current study are available from the corresponding author on reasonable request.
