# Supplementary figures and images for: Comparative analysis and correlation of cancer hotspot proteins and cell markers in tumor-normal adjacent breast and kidney samples using RPPA and LC-MS (part 1 of 3)
Source: Sci Rep. 2026 May 18;16:22442. doi: 10.1038/s41598-026-48754-2 (PMC13377106; doi:10.1038/s41598-026-48754-2)

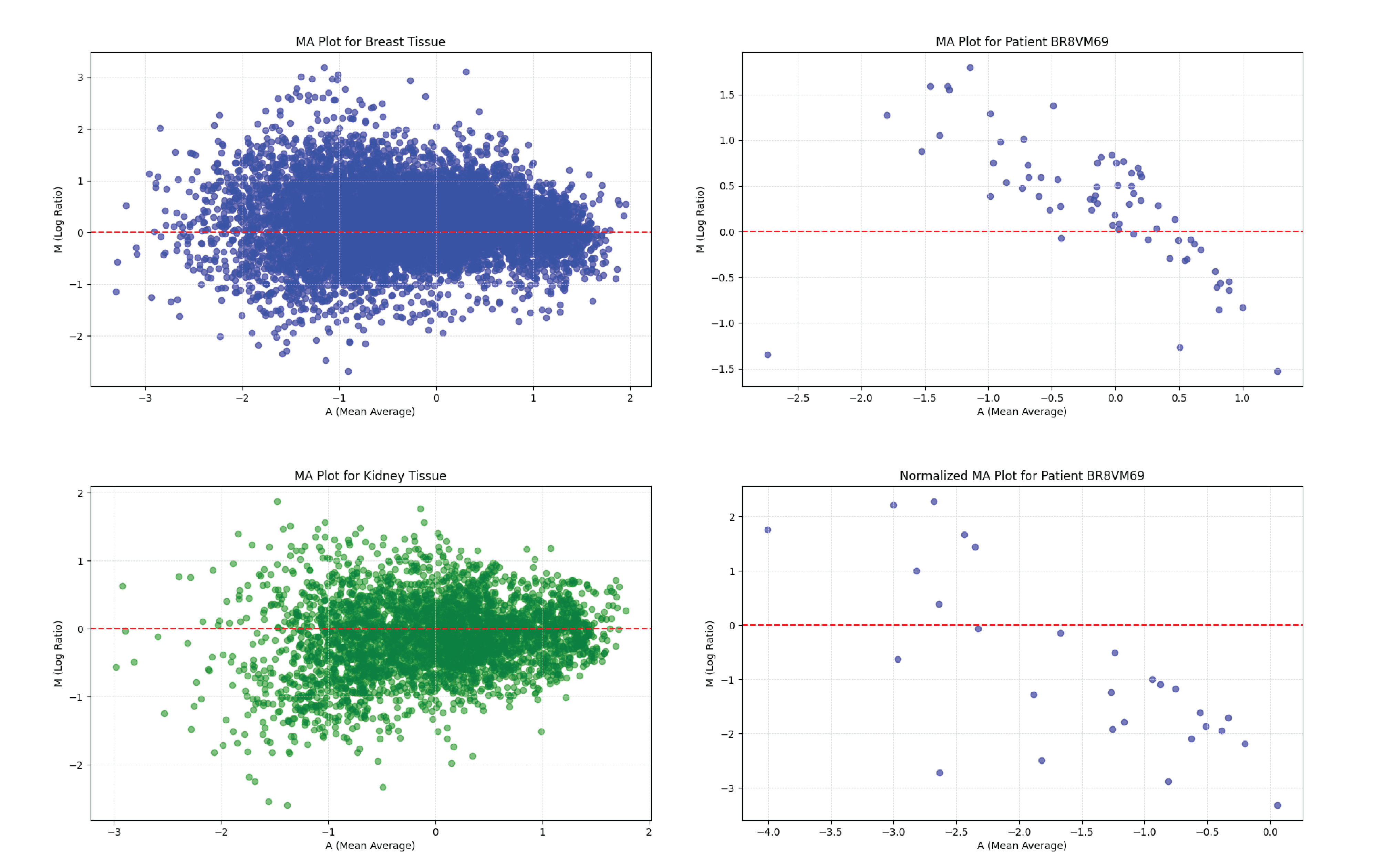

Supplement: Supplementary file 5 — Supplementary Material 5 [file 41598_2026_48754_MOESM5_ESM.png]

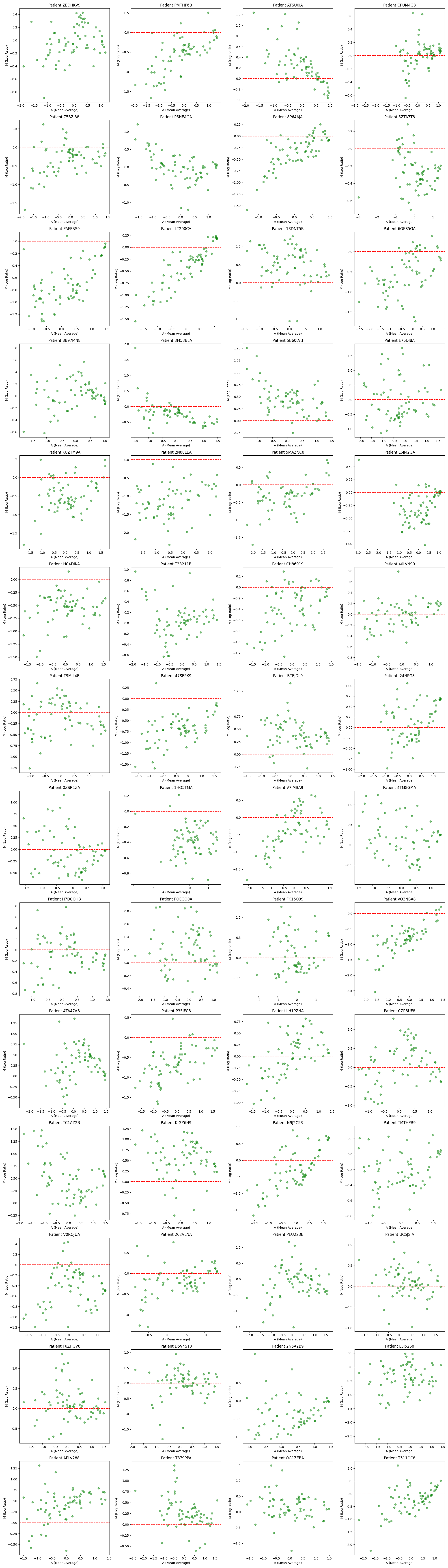

Supplement: Supplementary file 6 — Supplementary Material 6 [file 41598_2026_48754_MOESM6_ESM.png]

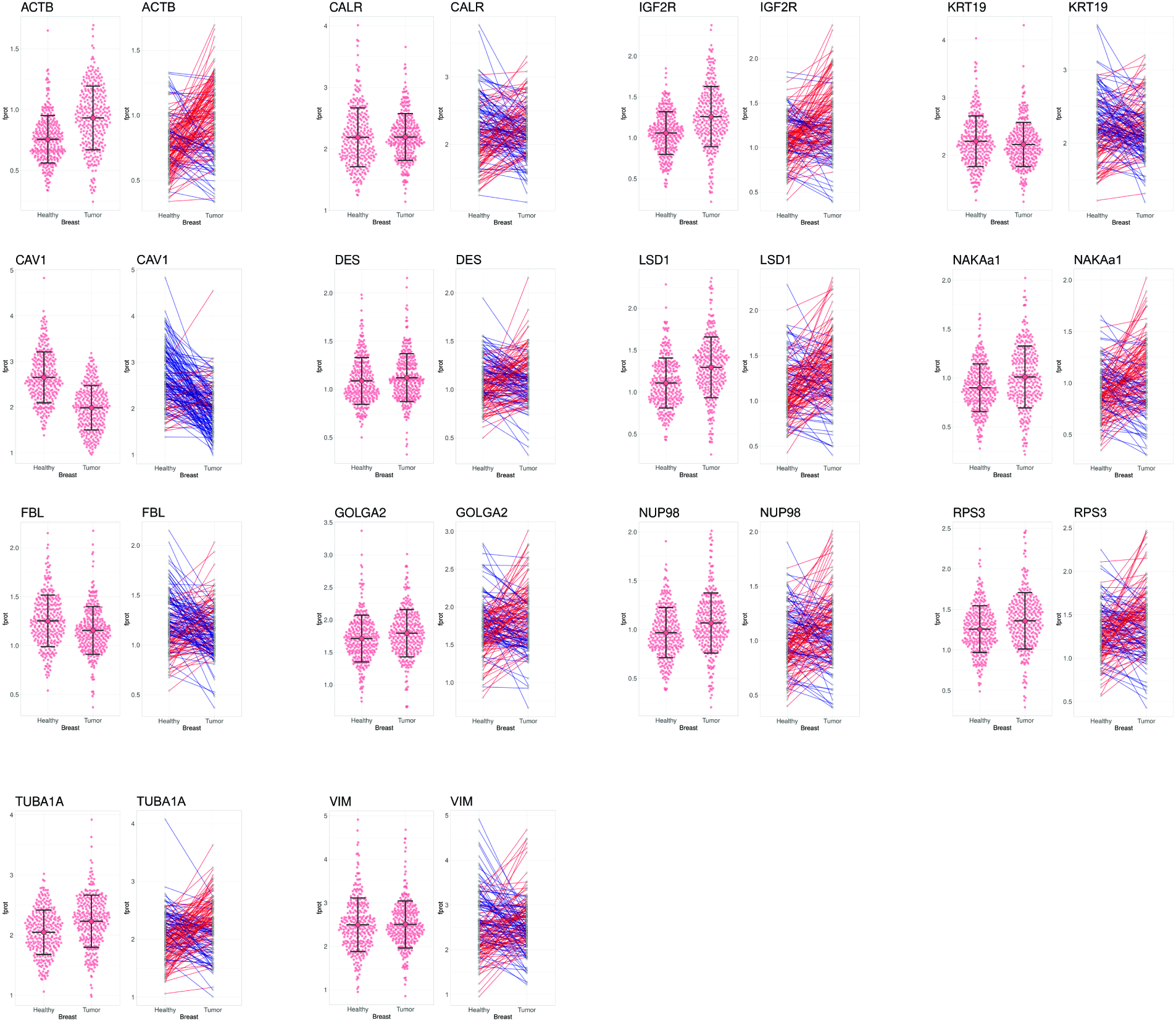

Supplement: Supplementary file 8 — Supplementary Material 8 [file 41598_2026_48754_MOESM8_ESM.tif]

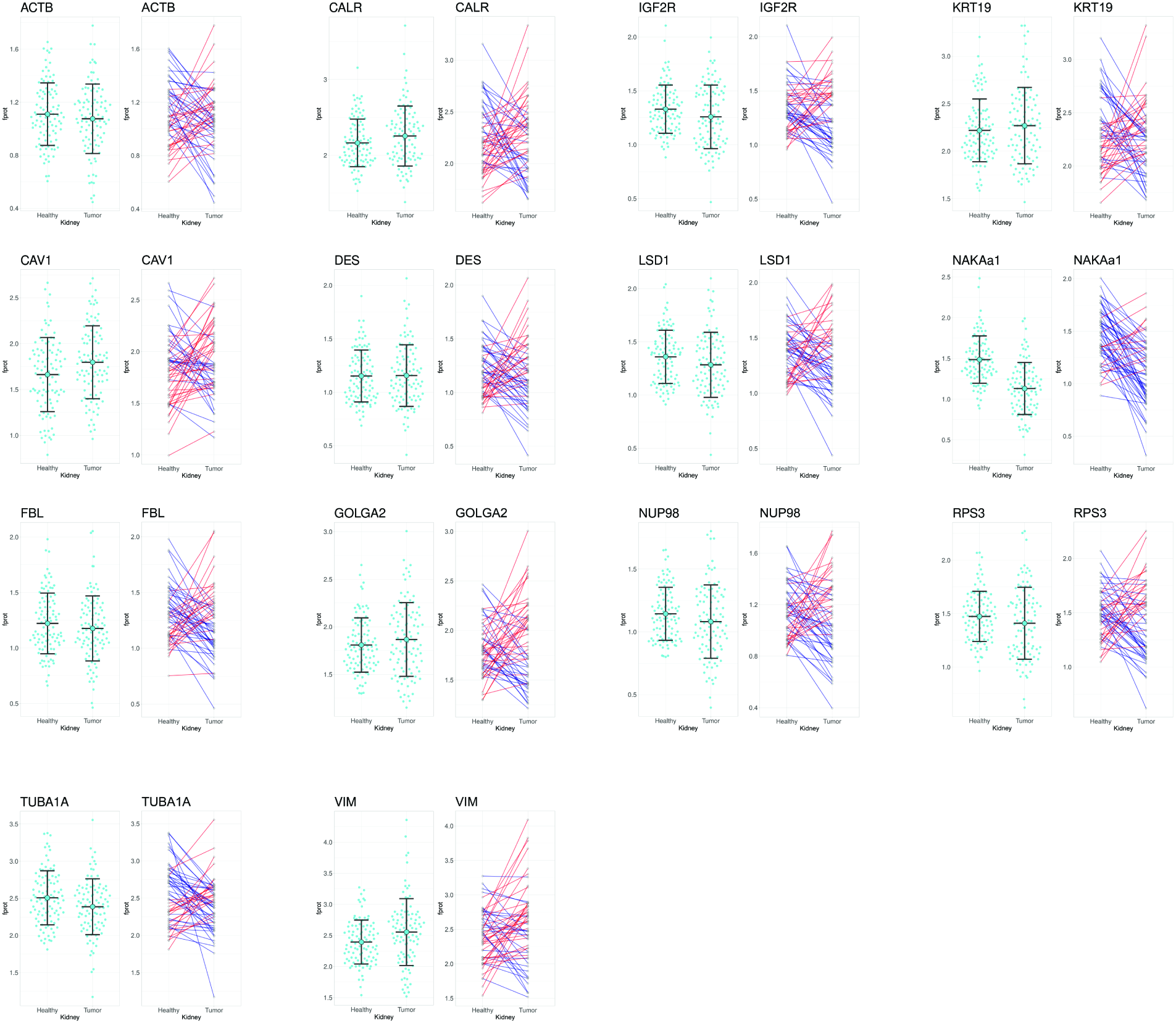

Supplement: Supplementary file 10 — Supplementary Material 10 [file 41598_2026_48754_MOESM10_ESM.tif]

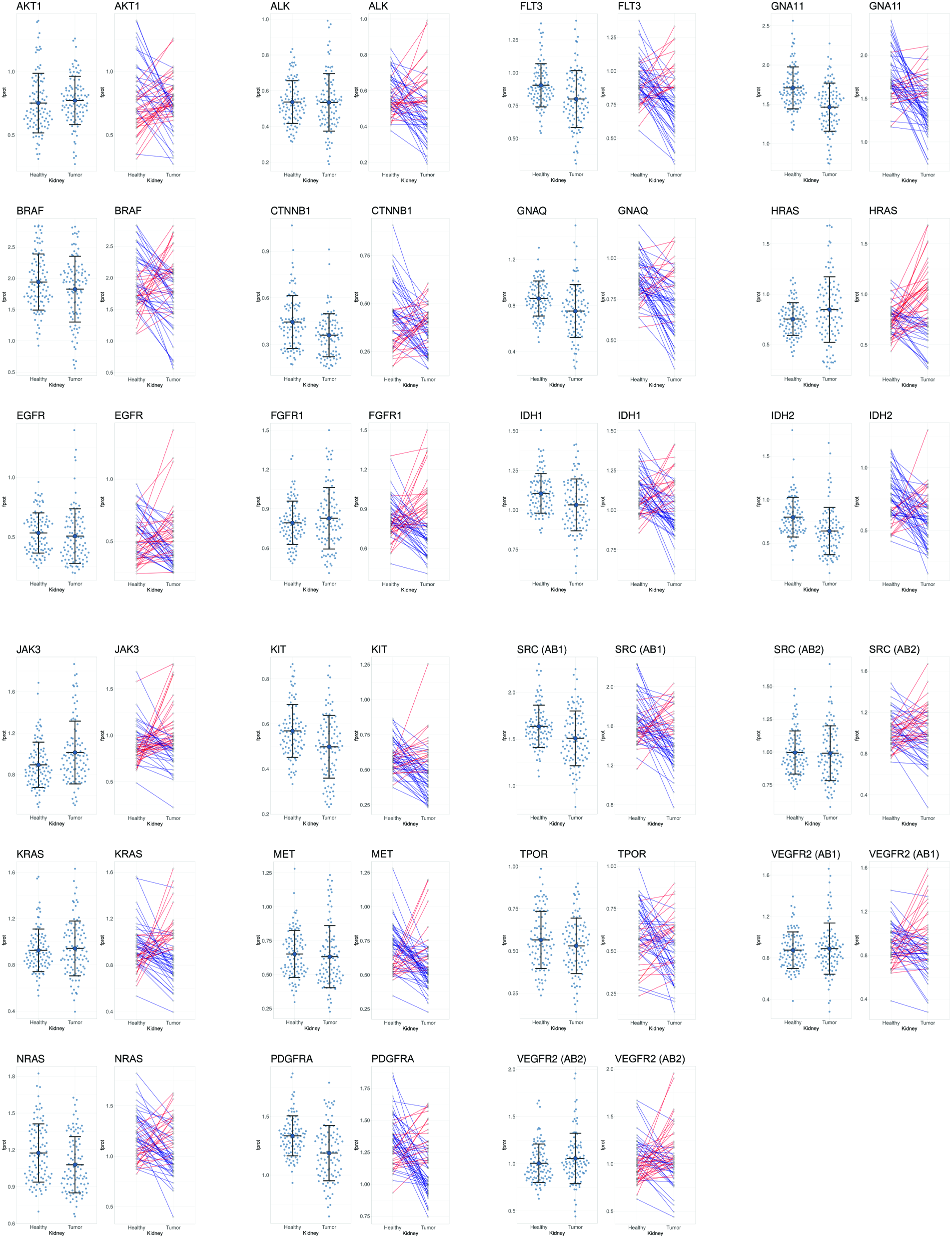

Supplement: Supplementary file 12 — Supplementary Material 12 [file 41598_2026_48754_MOESM12_ESM.tif]

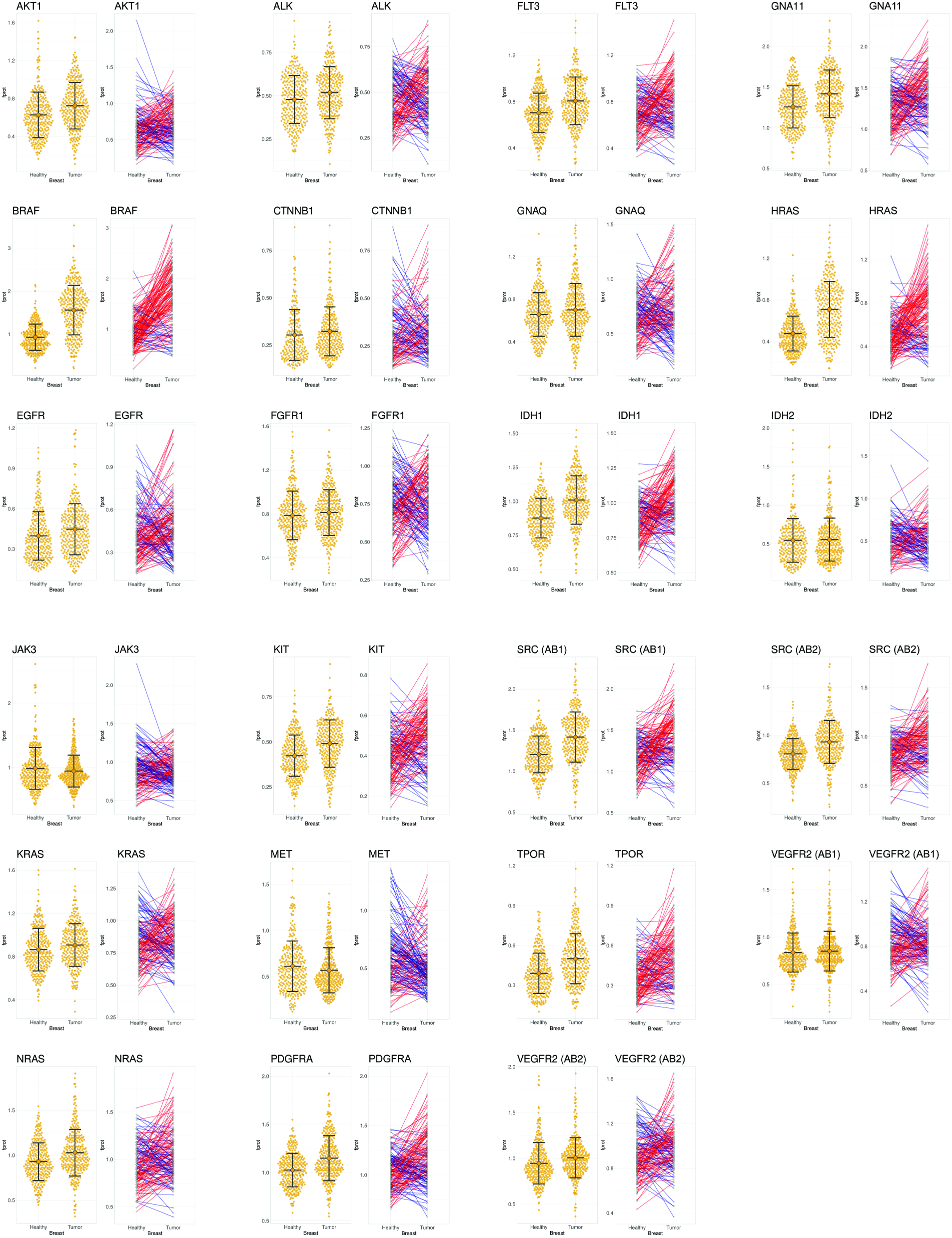

Supplement: Supplementary file 13 — Supplementary Material 13 [file 41598_2026_48754_MOESM13_ESM.tif]

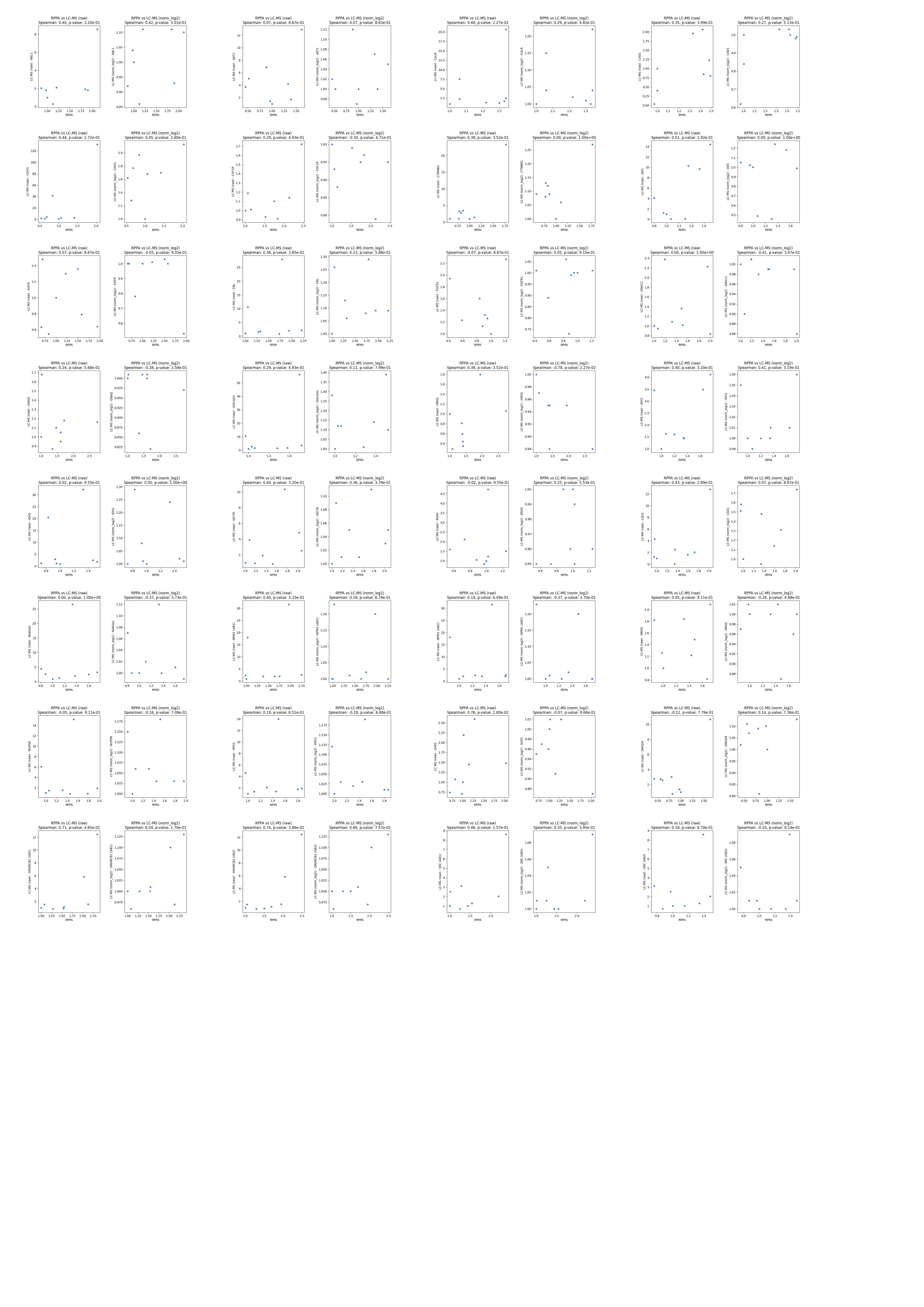

Supplement: Supplementary file 14 — Supplementary Material 14 [file 41598_2026_48754_MOESM14_ESM.zip › graph_H_I_Spearman_Breast_not_div_VIM.tif]

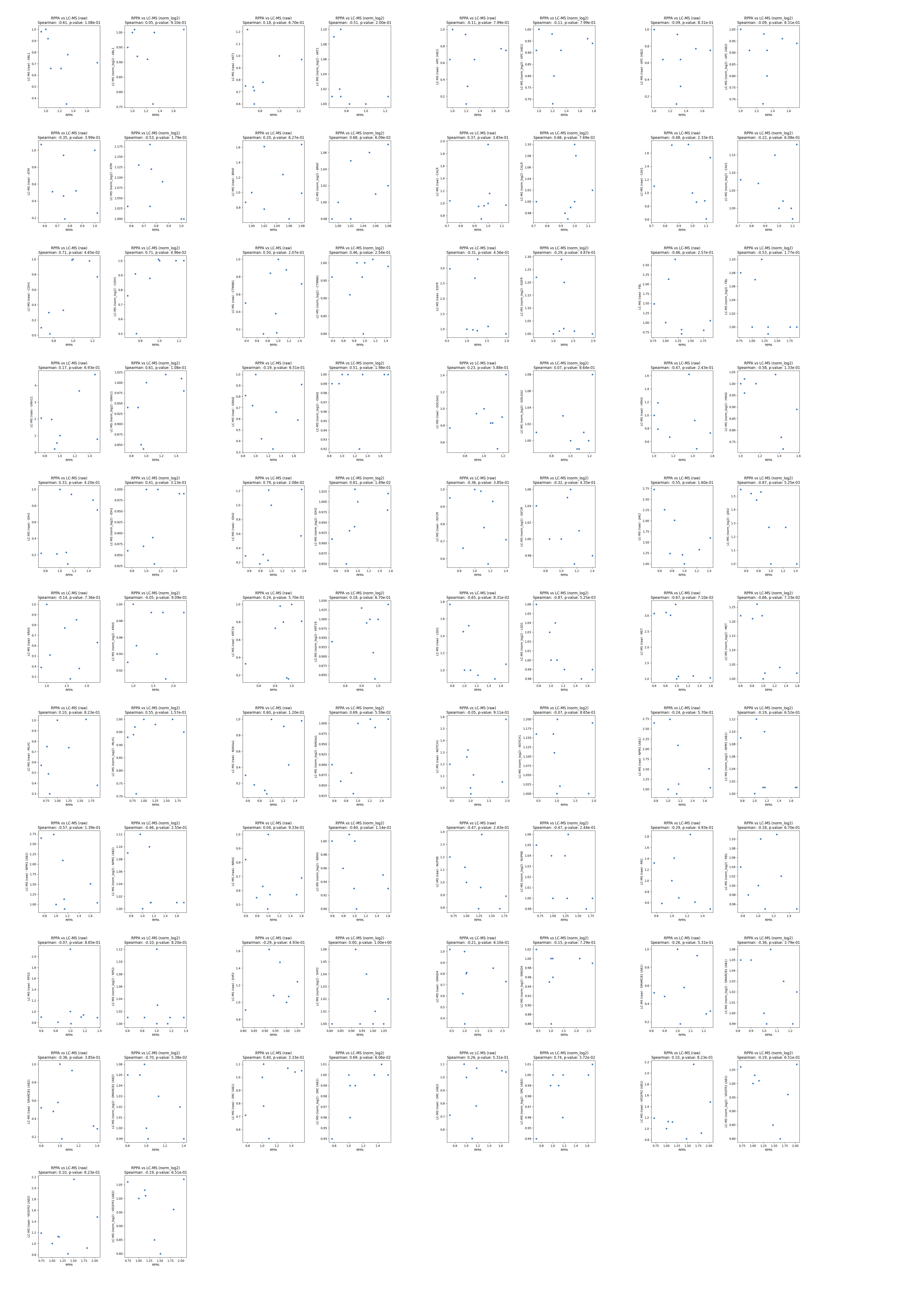

Supplement: Supplementary file 14 — Supplementary Material 14 [file 41598_2026_48754_MOESM14_ESM.zip › graph_H_I_Spearman_Kidney_not_div_VIM.tif]

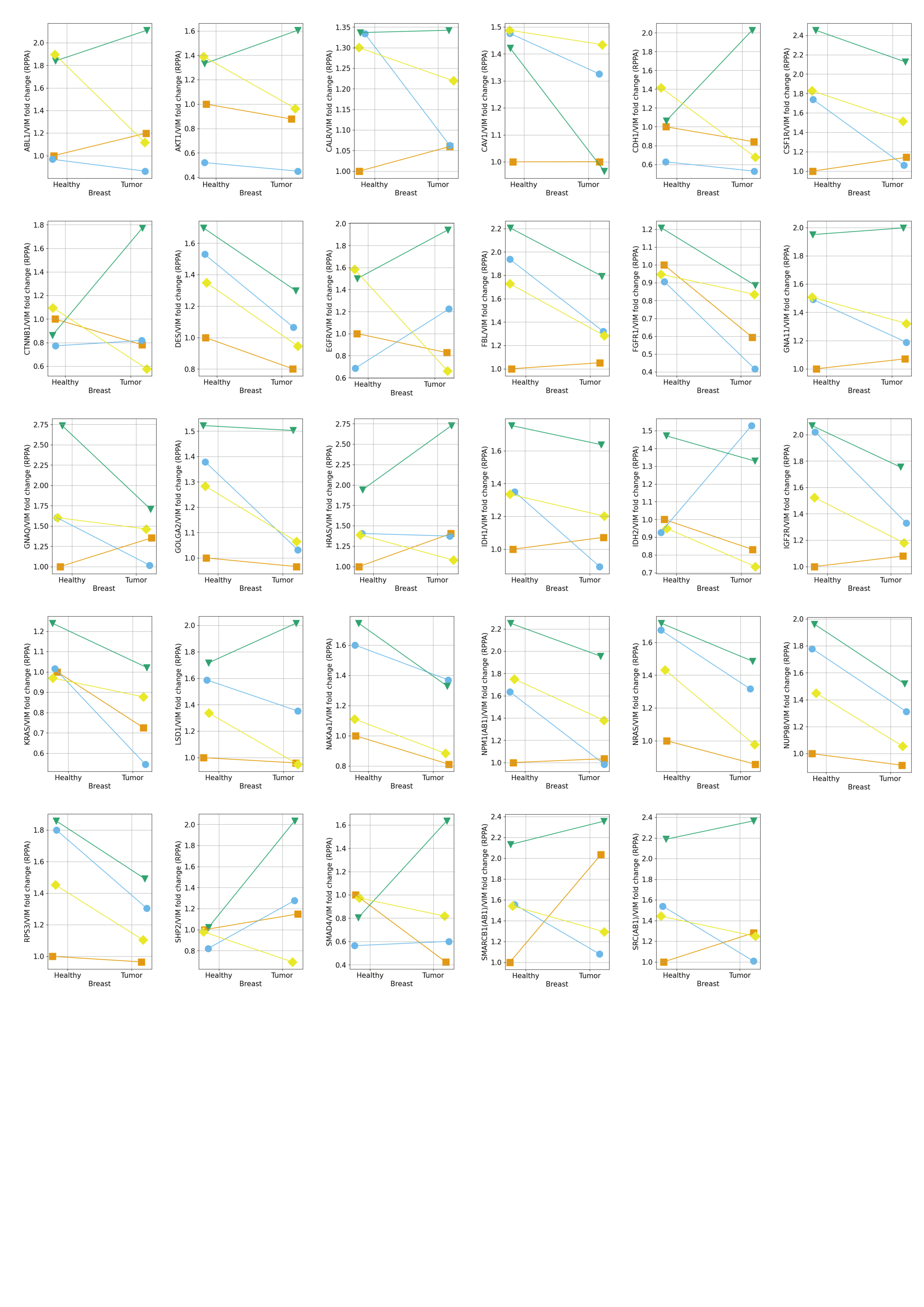

Supplement: Supplementary file 14 — Supplementary Material 14 [file 41598_2026_48754_MOESM14_ESM.zip › graph_A_Breast_RPPA_div_VIM.tif]

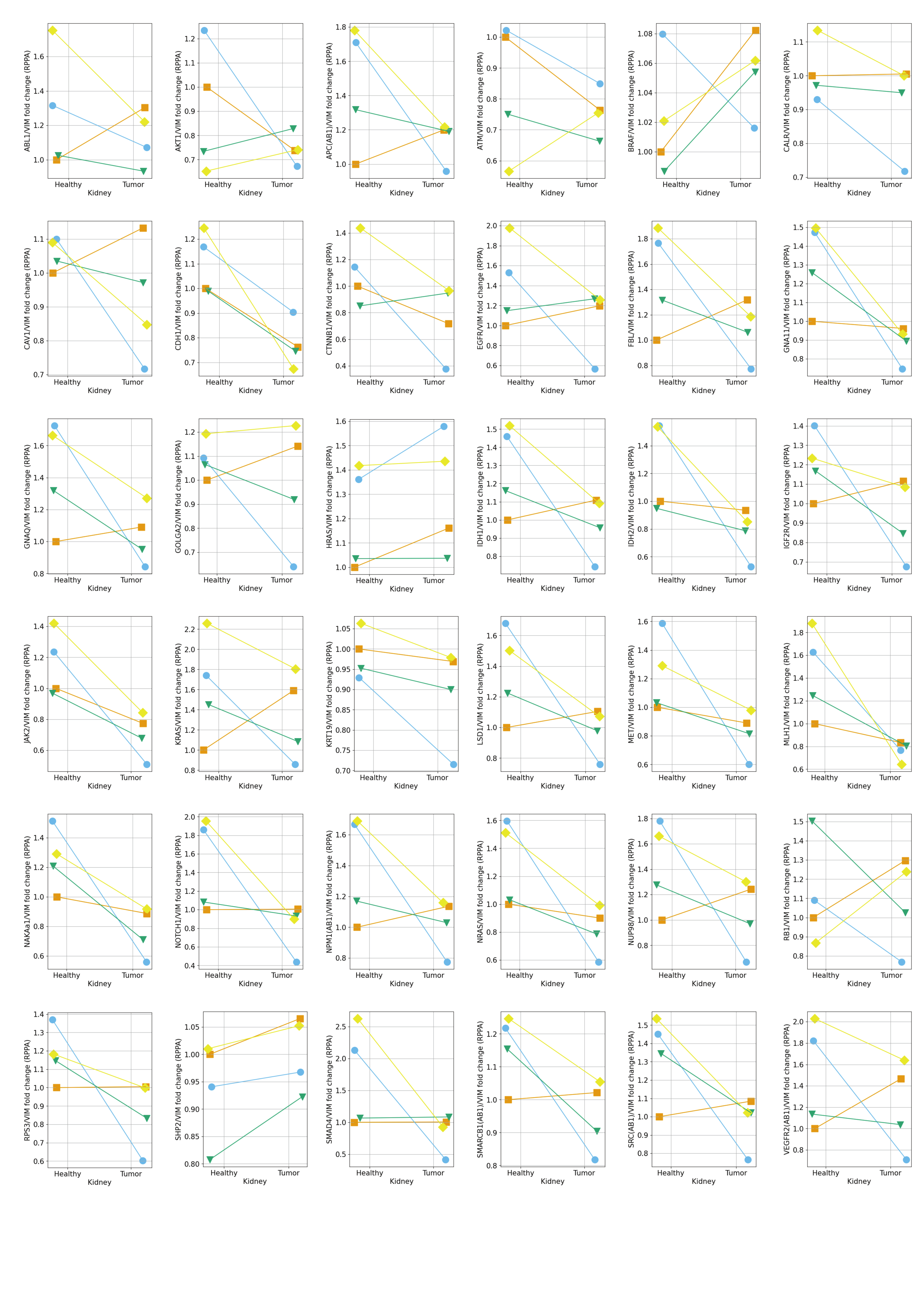

Supplement: Supplementary file 14 — Supplementary Material 14 [file 41598_2026_48754_MOESM14_ESM.zip › graph_A_Kidney_RPPA_div_VIM.tif]

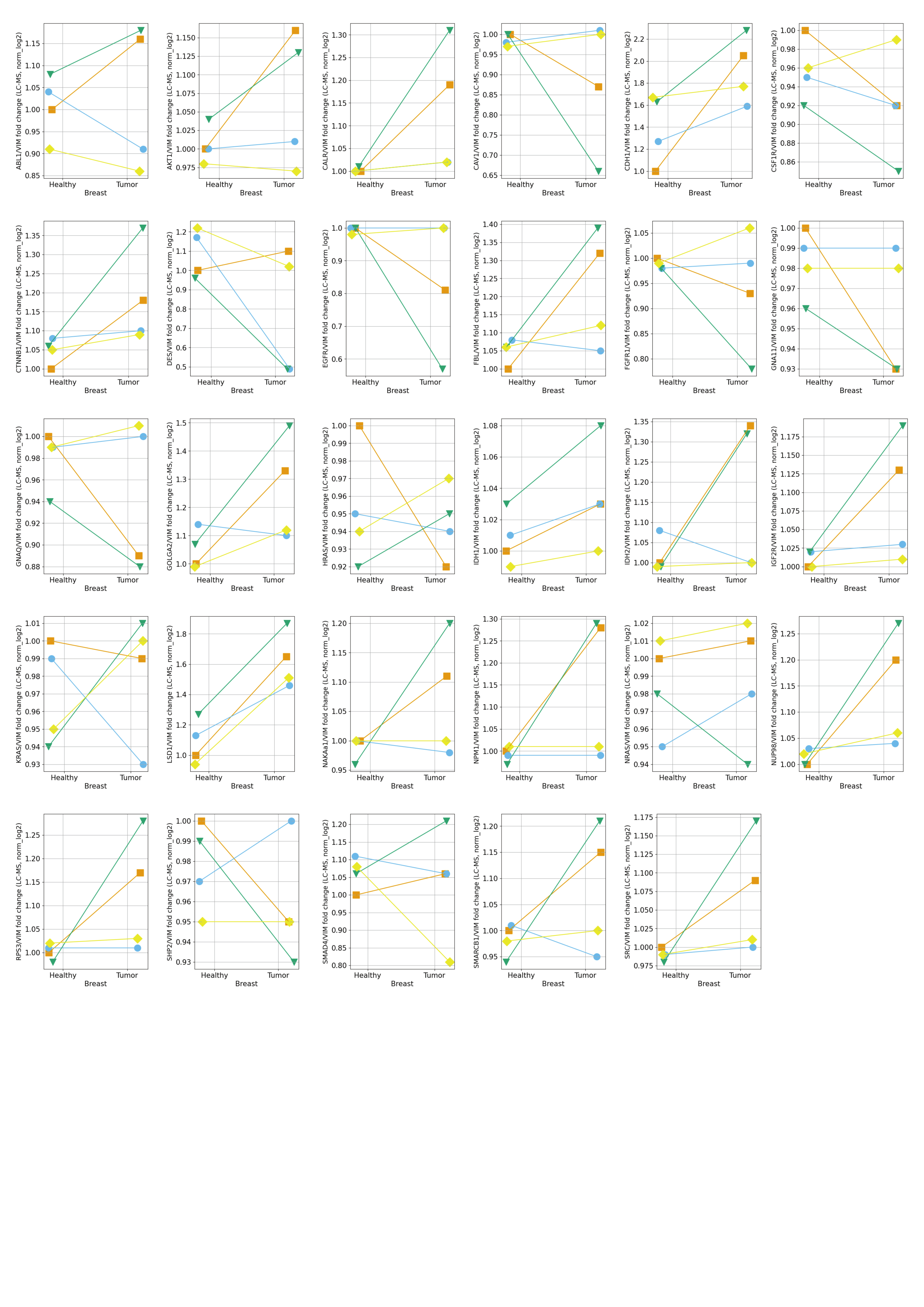

Supplement: Supplementary file 14 — Supplementary Material 14 [file 41598_2026_48754_MOESM14_ESM.zip › graph_B_Breast_LC_MS_norm_div_VIM.tif]

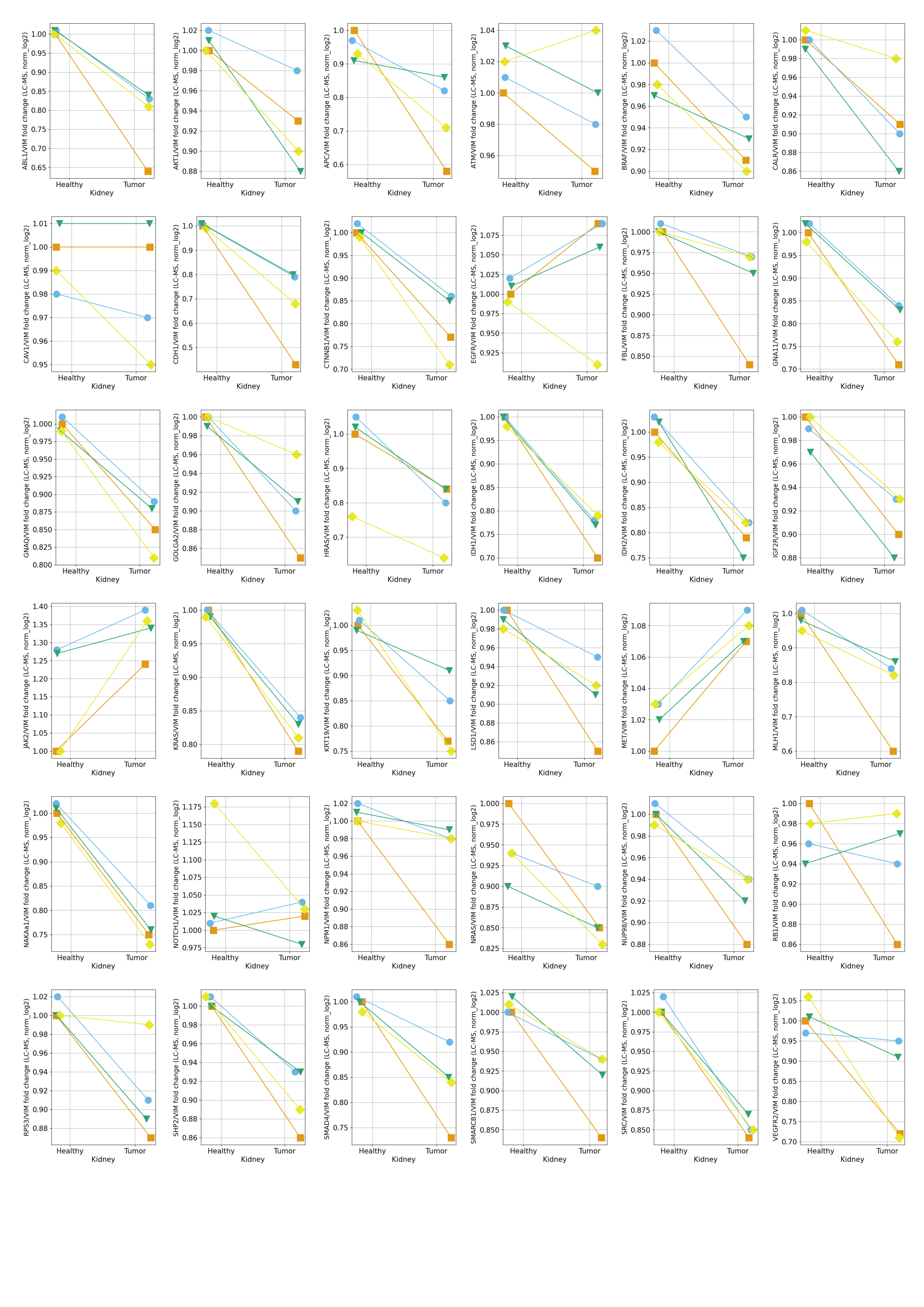

Supplement: Supplementary file 14 — Supplementary Material 14 [file 41598_2026_48754_MOESM14_ESM.zip › graph_B_Kidney_LC_MS_norm_div_VIM.tif]

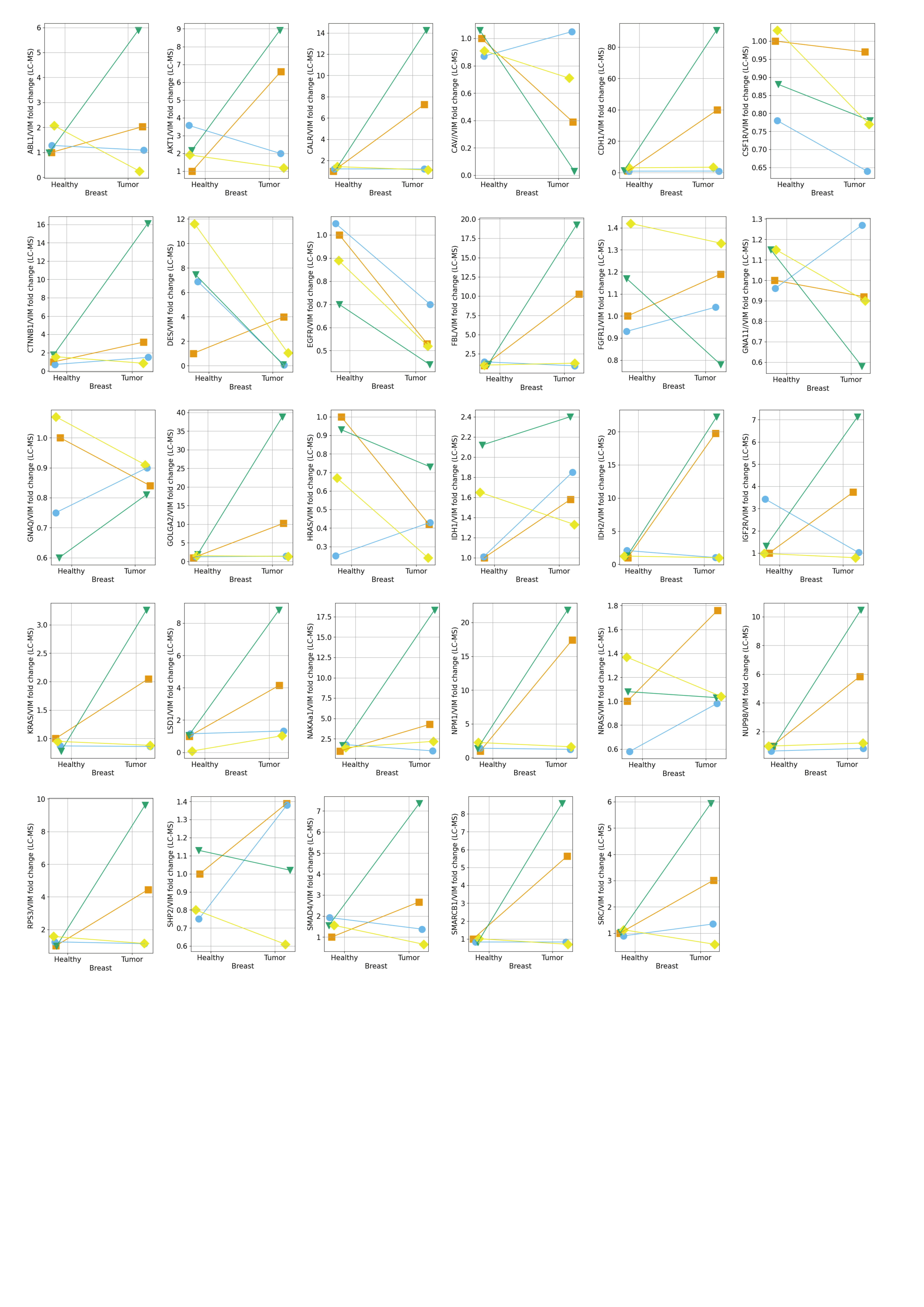

Supplement: Supplementary file 14 — Supplementary Material 14 [file 41598_2026_48754_MOESM14_ESM.zip › graph_C_Breast_LC_MS_raw_div_VIM.tif]

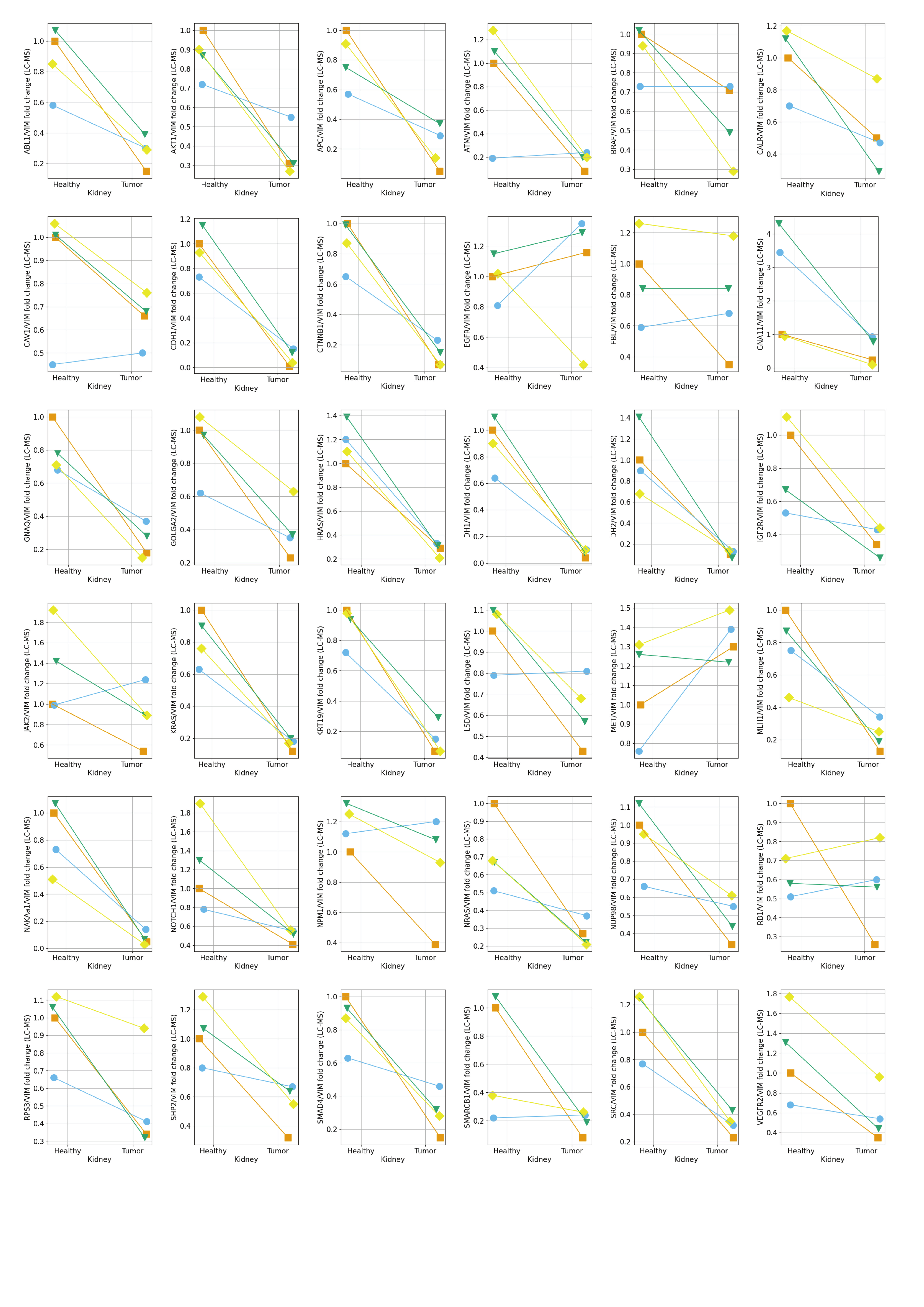

Supplement: Supplementary file 14 — Supplementary Material 14 [file 41598_2026_48754_MOESM14_ESM.zip › graph_C_Kidney_LC_MS_raw_div_VIM.tif]

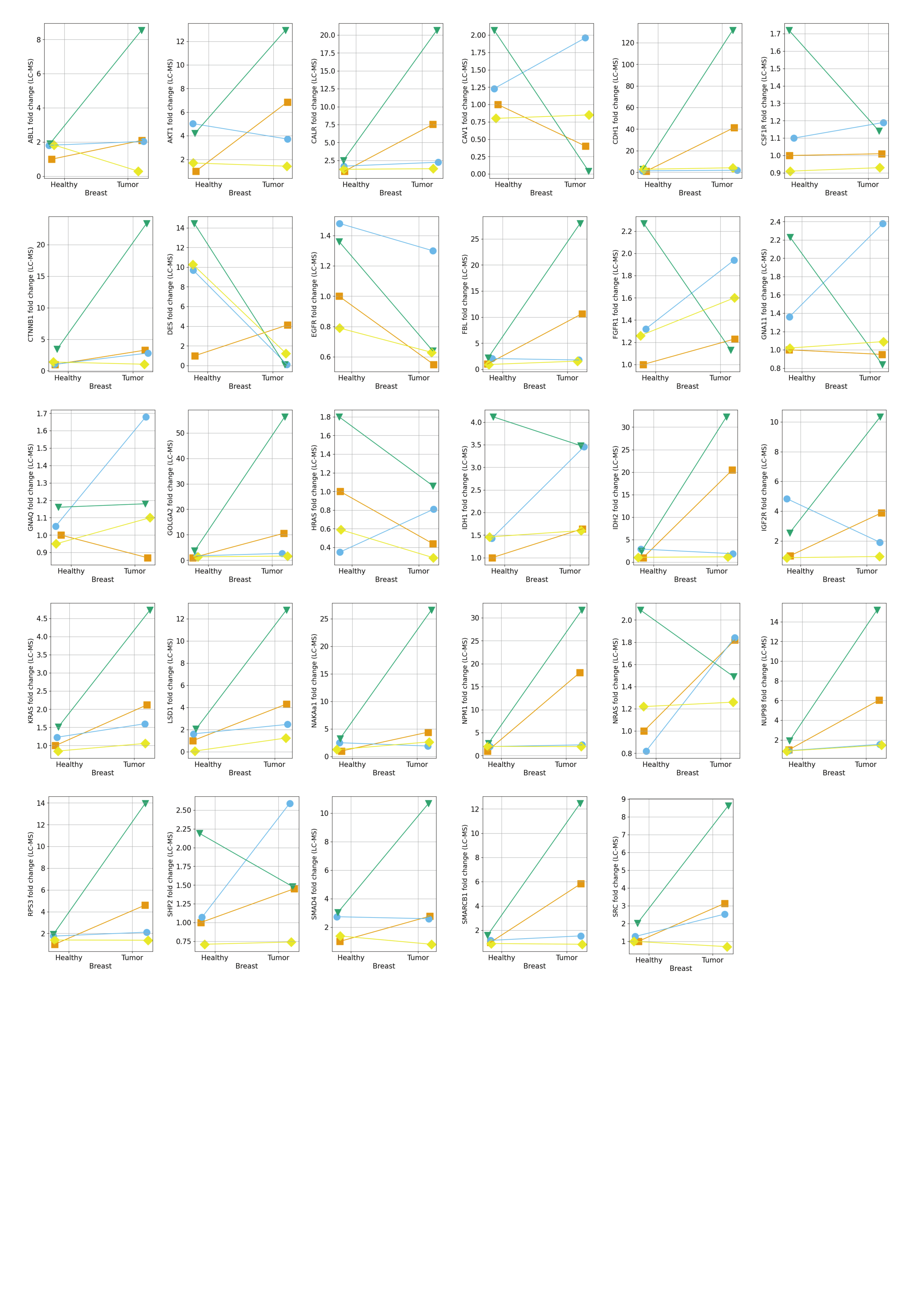

Supplement: Supplementary file 14 — Supplementary Material 14 [file 41598_2026_48754_MOESM14_ESM.zip › graph_D_Breast_LC_MS_raw_not_div_VIM.tif]

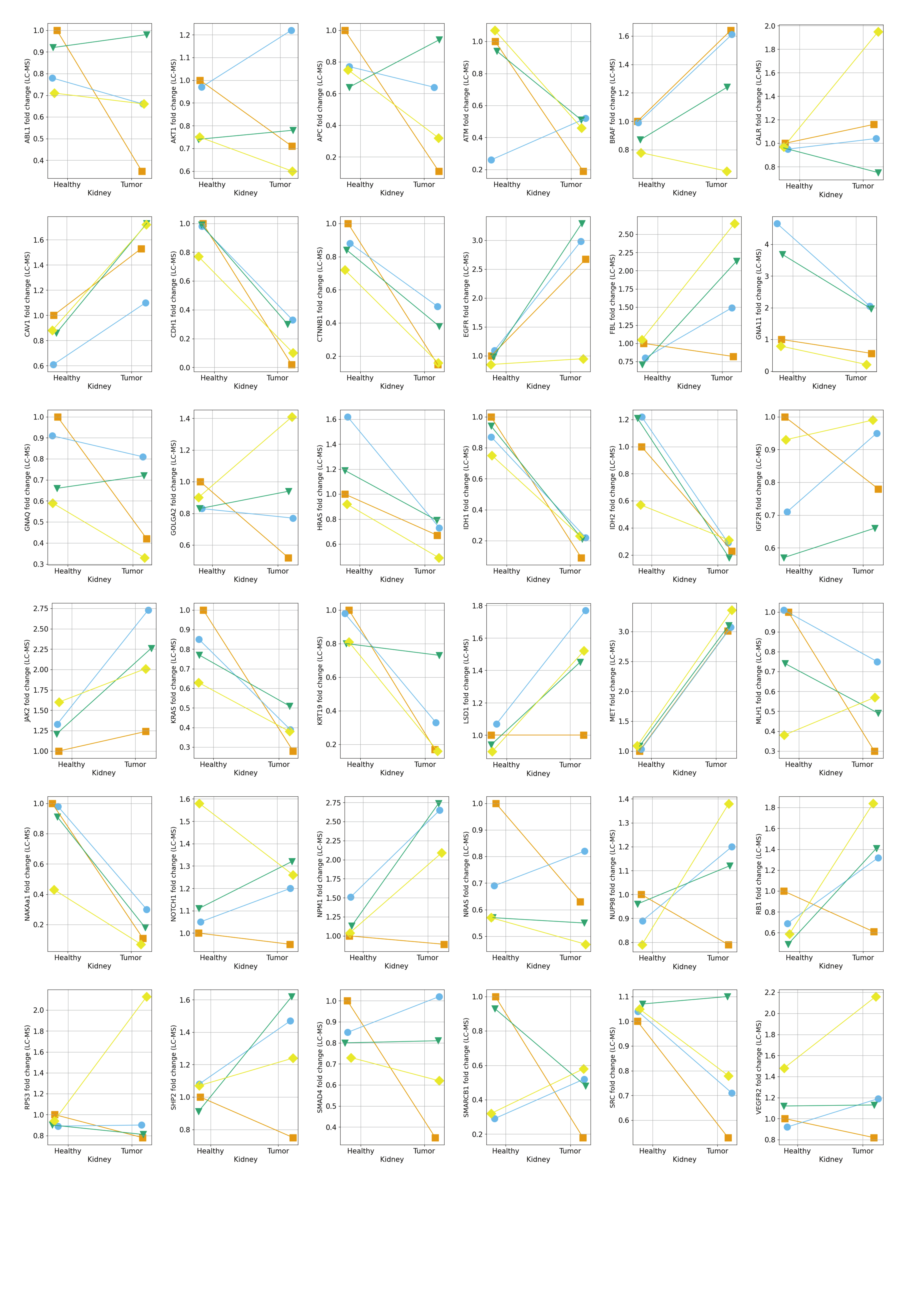

Supplement: Supplementary file 14 — Supplementary Material 14 [file 41598_2026_48754_MOESM14_ESM.zip › graph_D_Kidney_LC_MS_raw_not_div_VIM.tif]

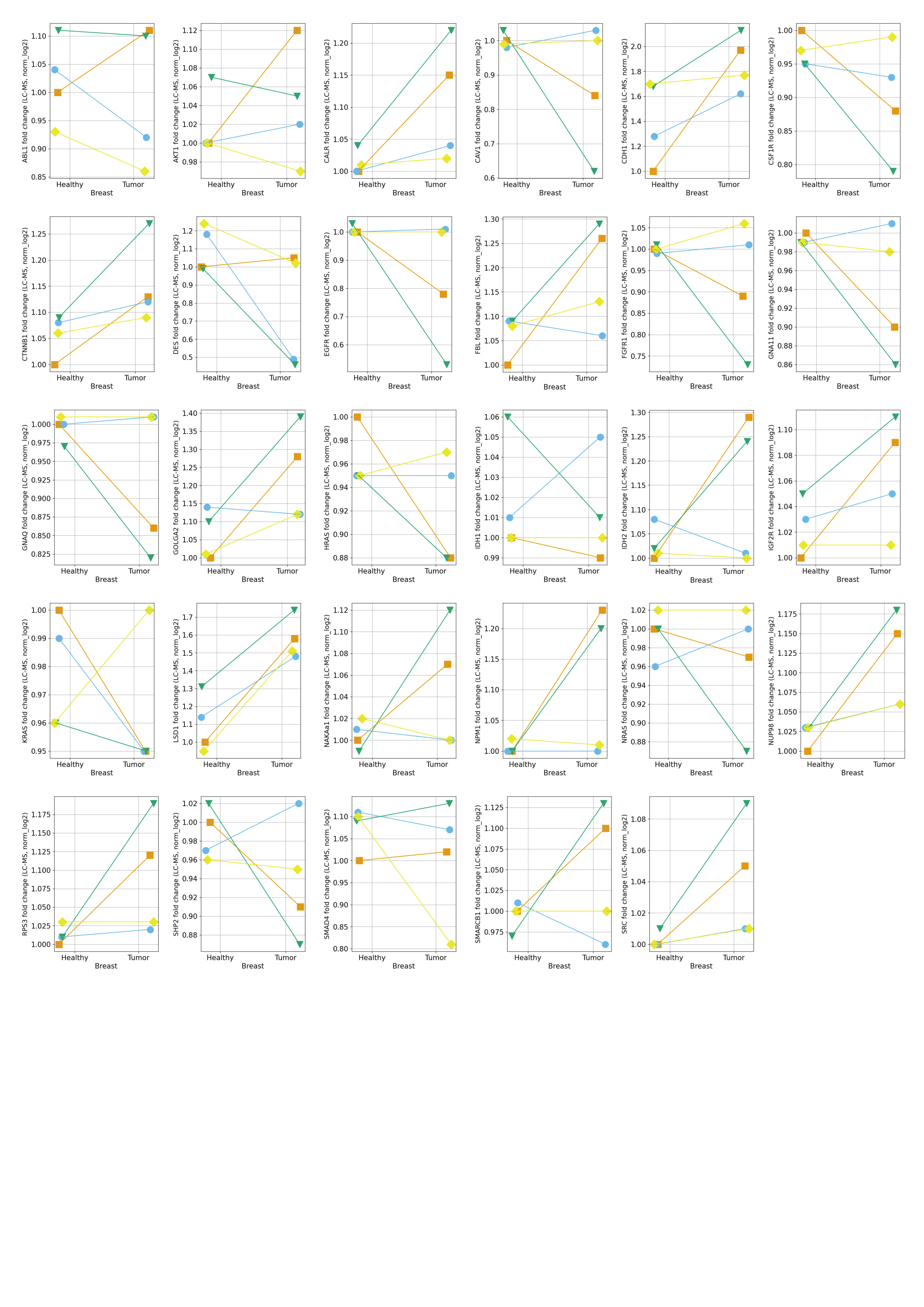

Supplement: Supplementary file 14 — Supplementary Material 14 [file 41598_2026_48754_MOESM14_ESM.zip › graph_E_Breast_LC_MS_norm_not_div_VIM.tif]

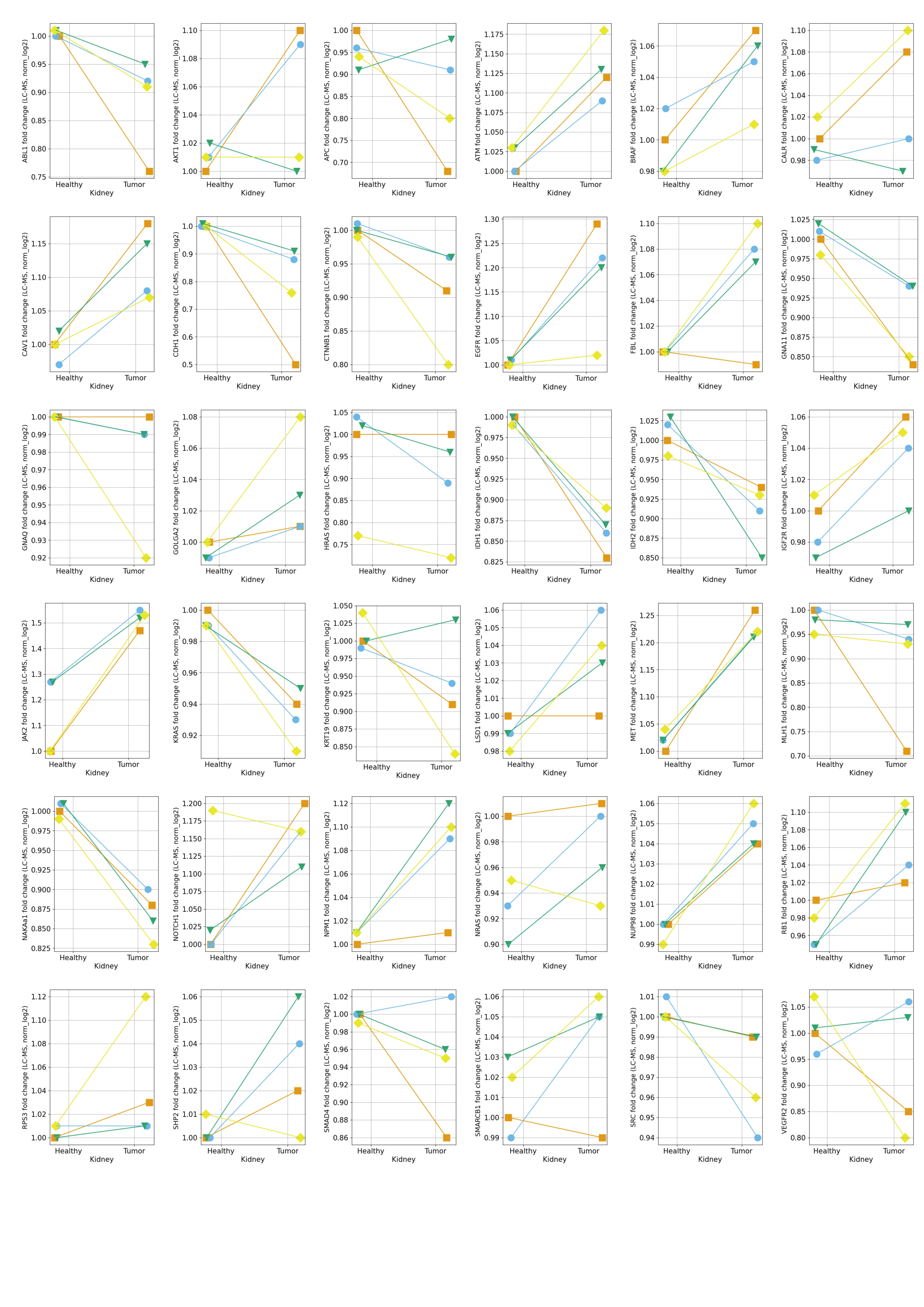

Supplement: Supplementary file 14 — Supplementary Material 14 [file 41598_2026_48754_MOESM14_ESM.zip › graph_E_Kidney_LC_MS_norm_not_div_VIM.tif]

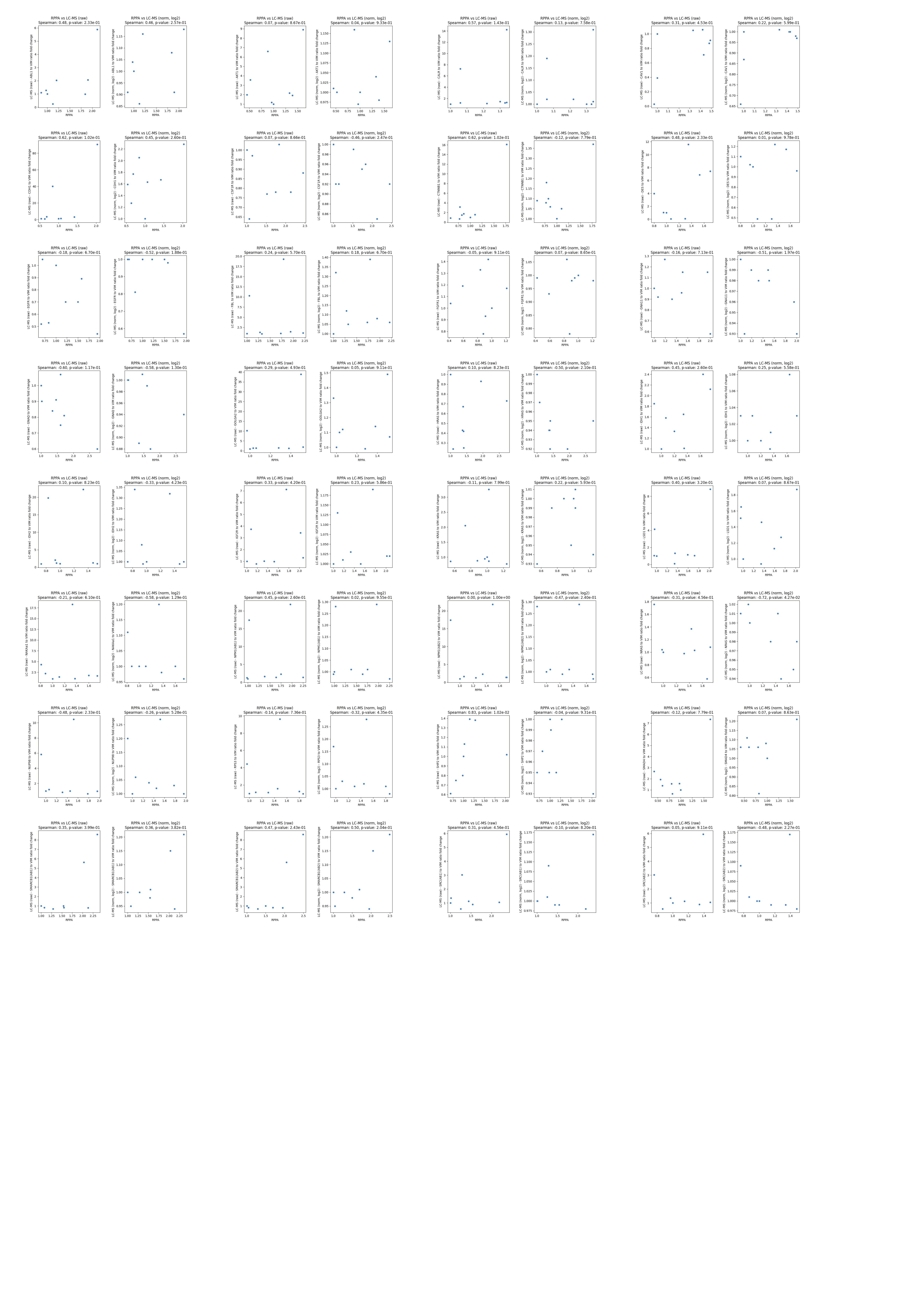

Supplement: Supplementary file 14 — Supplementary Material 14 [file 41598_2026_48754_MOESM14_ESM.zip › graph_F_G_Spearman_Breast_div_VIM.tif]

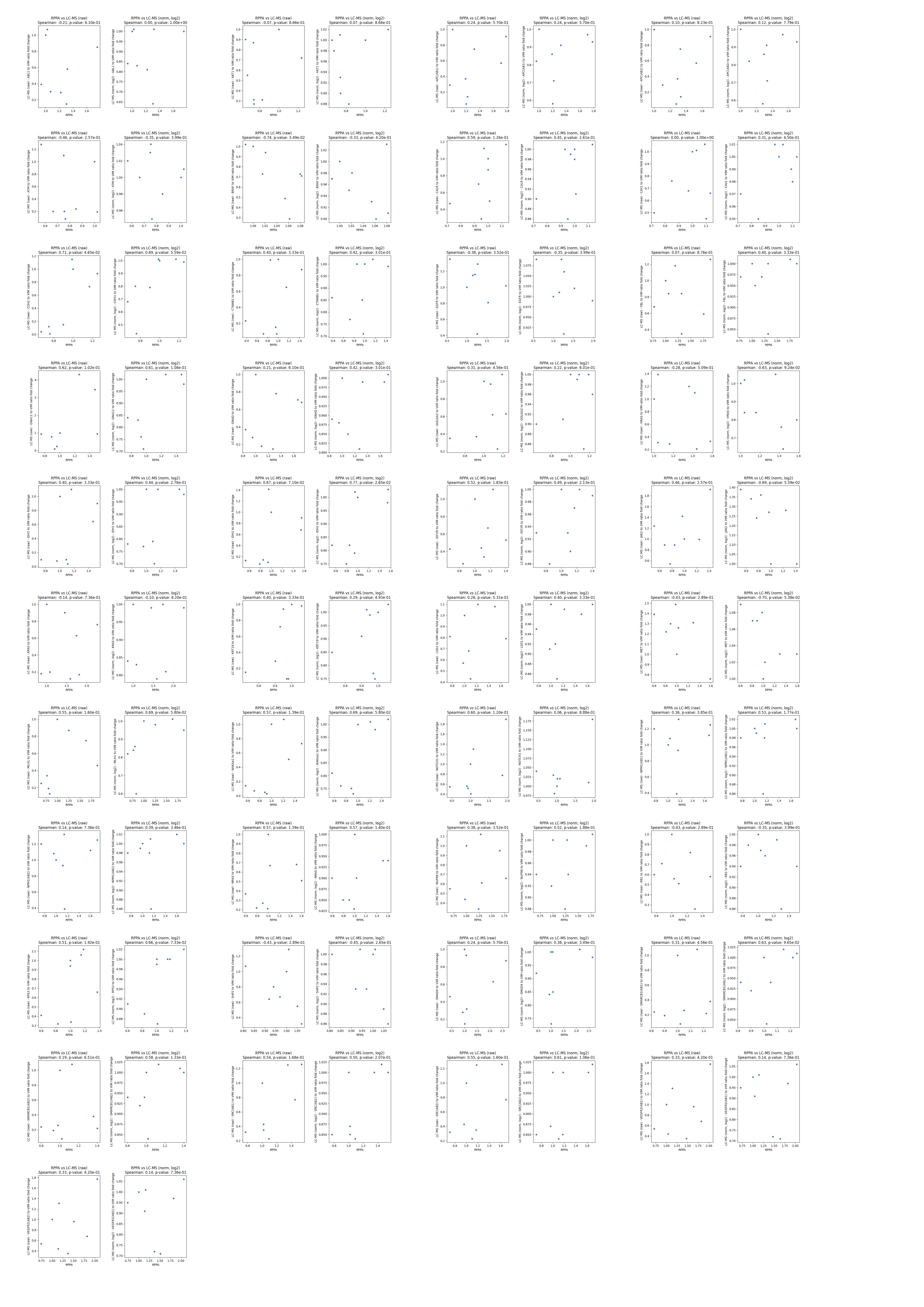

Supplement: Supplementary file 14 — Supplementary Material 14 [file 41598_2026_48754_MOESM14_ESM.zip › graph_F_G_Spearman_Kidney_div_VIM.tif]

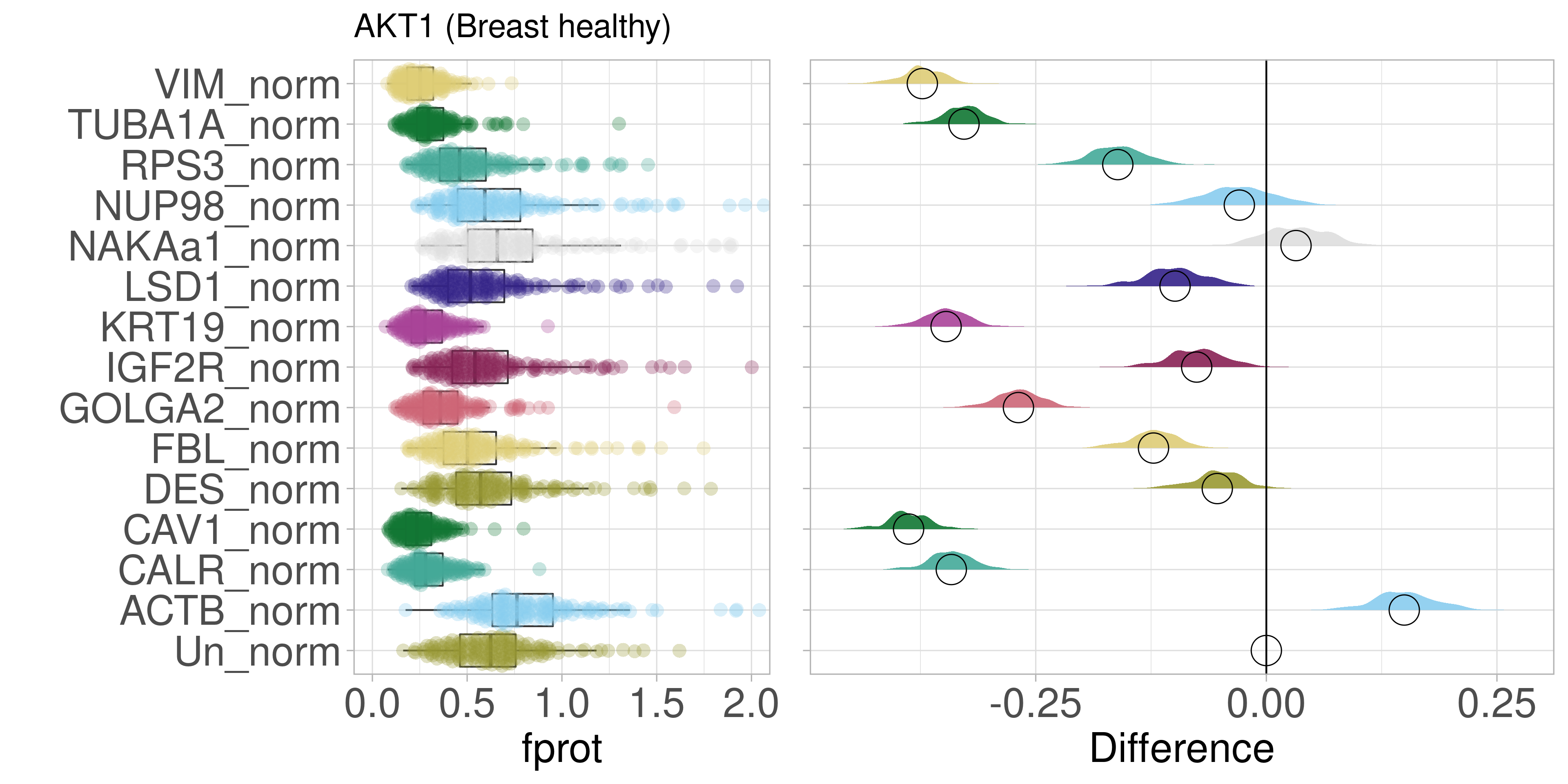

Supplement: Supplementary file 17 — Supplementary Material 17 [file 41598_2026_48754_MOESM17_ESM.zip › RPPA normalizations to cell markers/Breast_Plots/Oncoproteins_breast/AKT1_Breast_H.png]

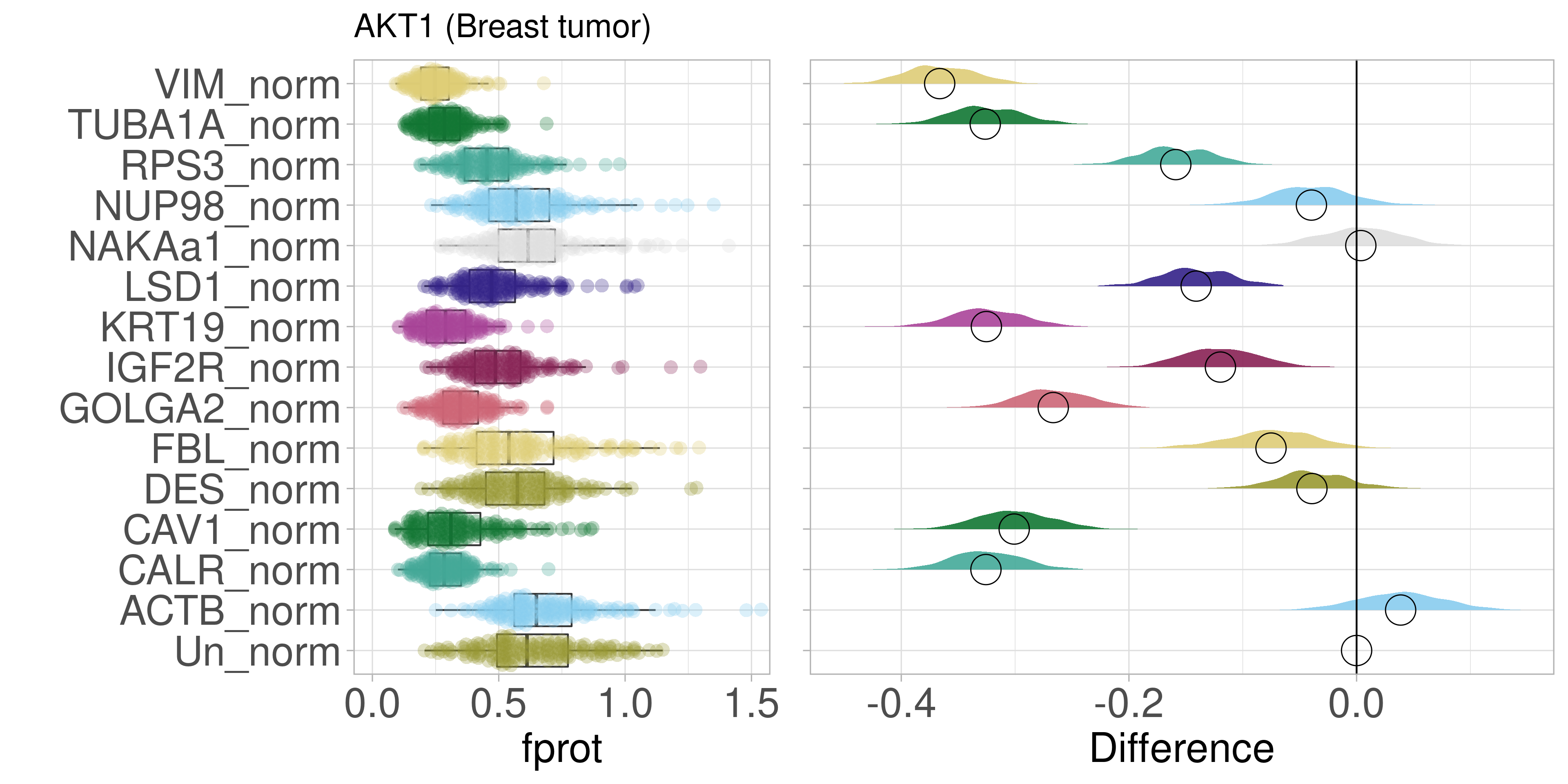

Supplement: Supplementary file 17 — Supplementary Material 17 [file 41598_2026_48754_MOESM17_ESM.zip › RPPA normalizations to cell markers/Breast_Plots/Oncoproteins_breast/AKT1_Breast_T.png]

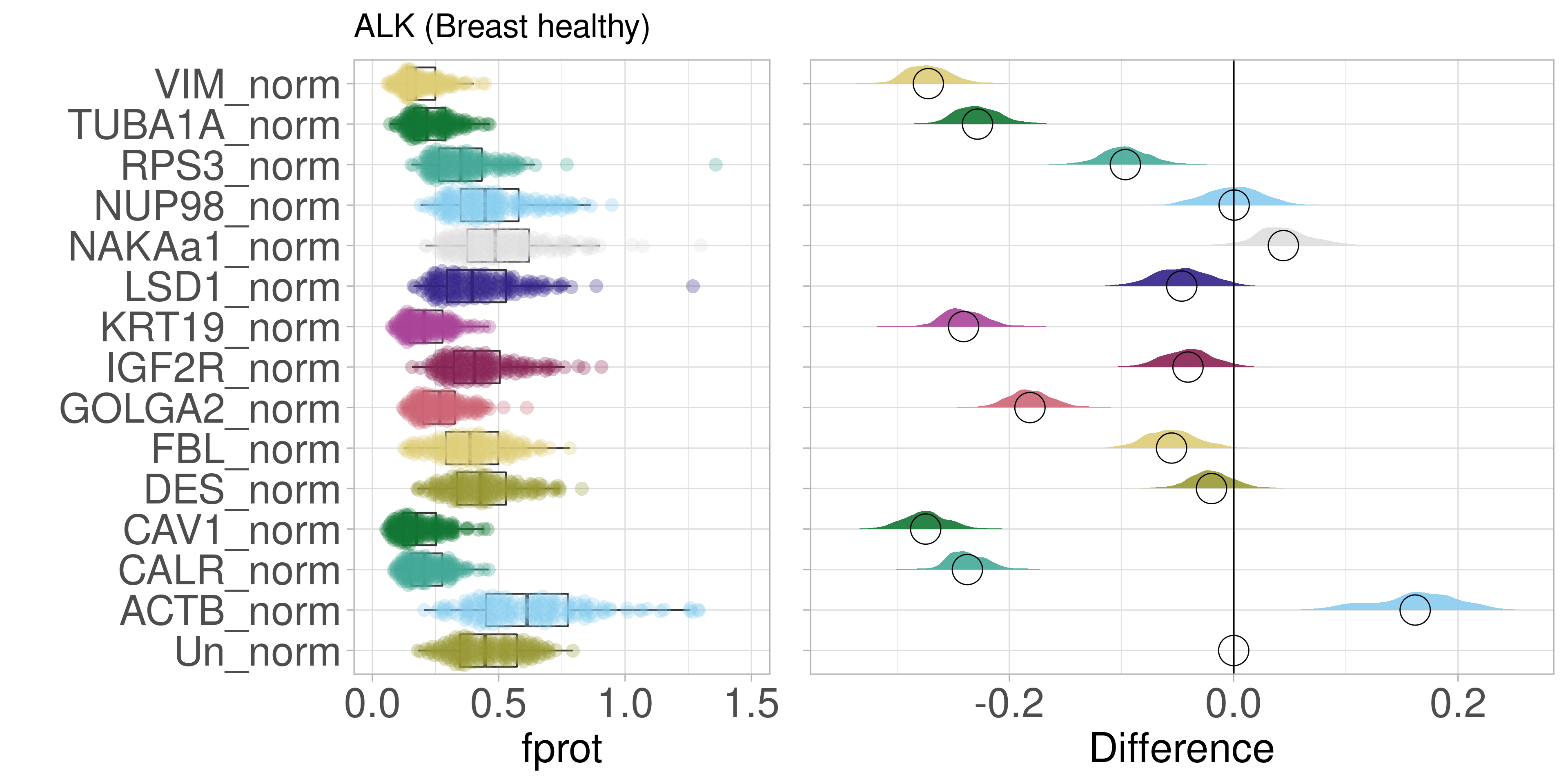

Supplement: Supplementary file 17 — Supplementary Material 17 [file 41598_2026_48754_MOESM17_ESM.zip › RPPA normalizations to cell markers/Breast_Plots/Oncoproteins_breast/ALK_Breast_H.png]

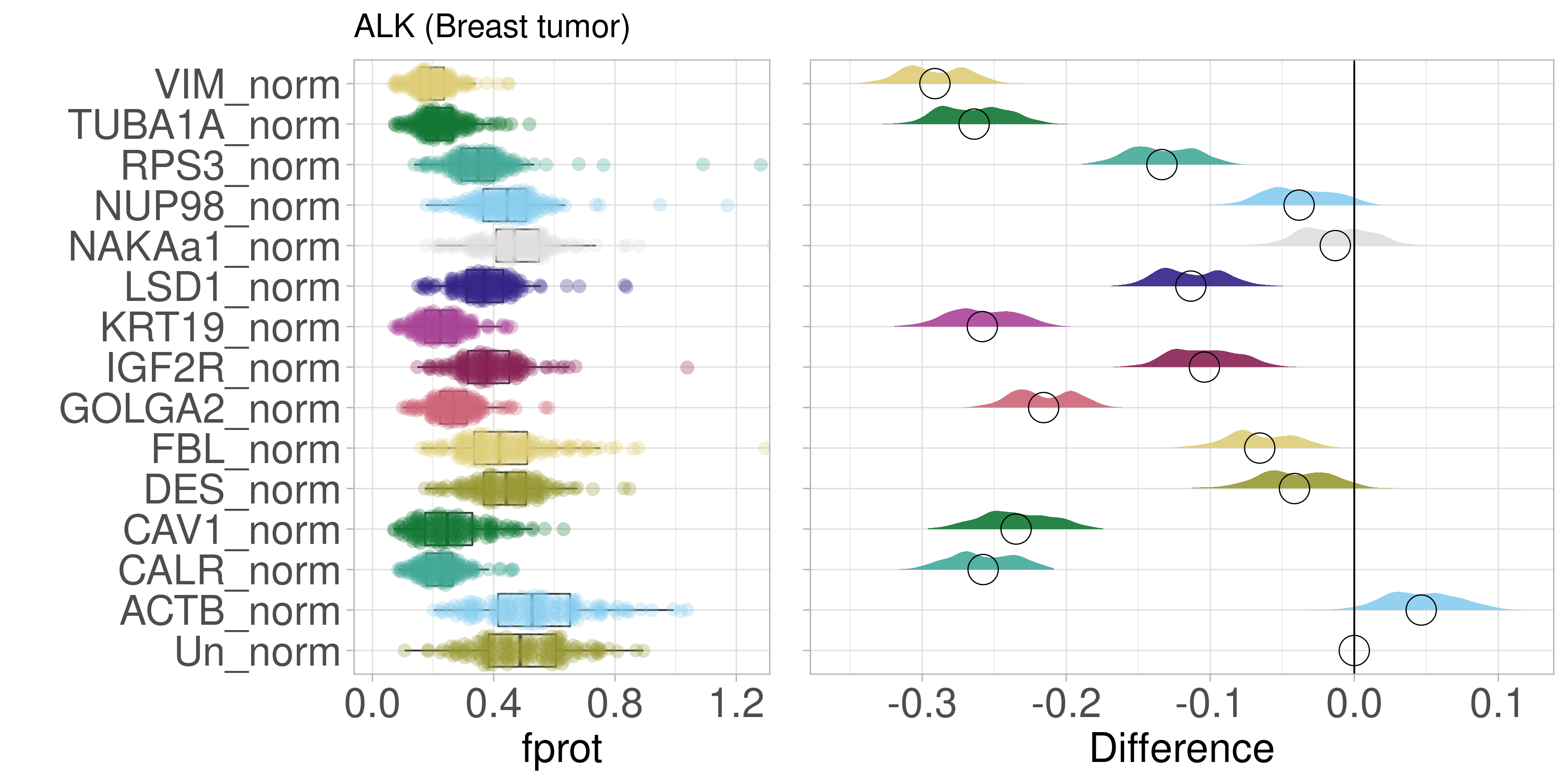

Supplement: Supplementary file 17 — Supplementary Material 17 [file 41598_2026_48754_MOESM17_ESM.zip › RPPA normalizations to cell markers/Breast_Plots/Oncoproteins_breast/ALK_Breast_T.png]

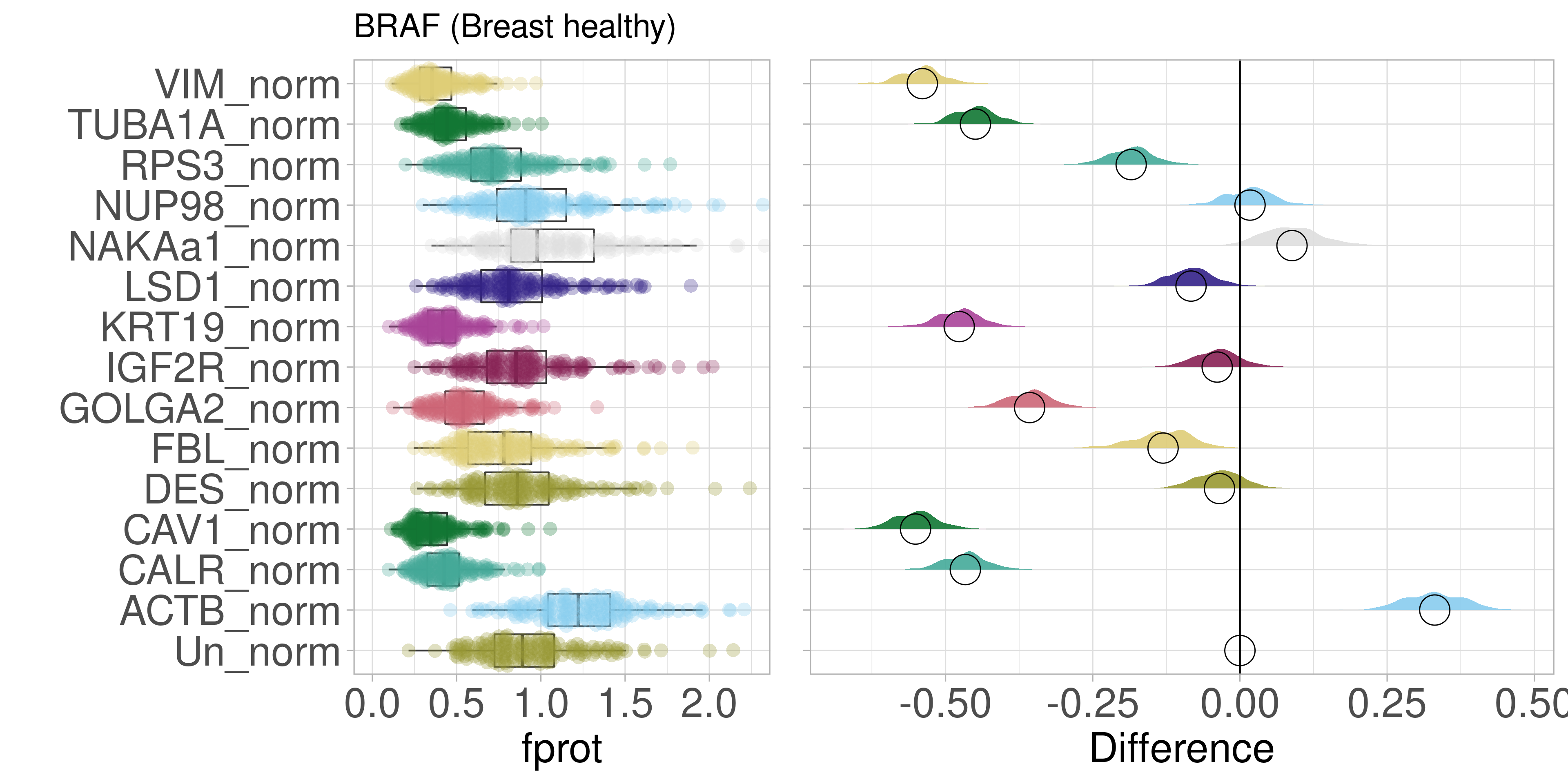

Supplement: Supplementary file 17 — Supplementary Material 17 [file 41598_2026_48754_MOESM17_ESM.zip › RPPA normalizations to cell markers/Breast_Plots/Oncoproteins_breast/BRAF_Breast_H.png]

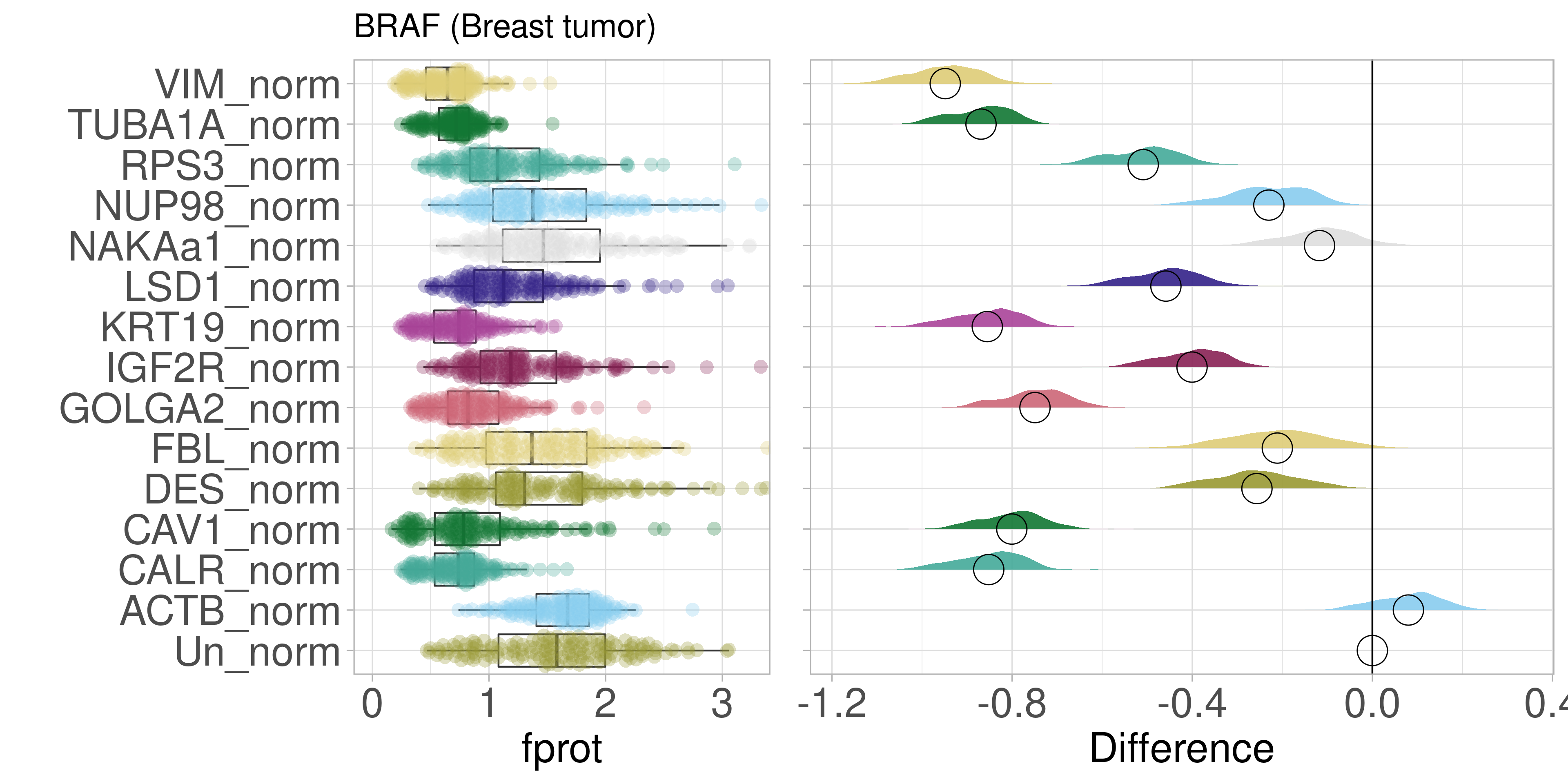

Supplement: Supplementary file 17 — Supplementary Material 17 [file 41598_2026_48754_MOESM17_ESM.zip › RPPA normalizations to cell markers/Breast_Plots/Oncoproteins_breast/BRAF_Breast_T.png]

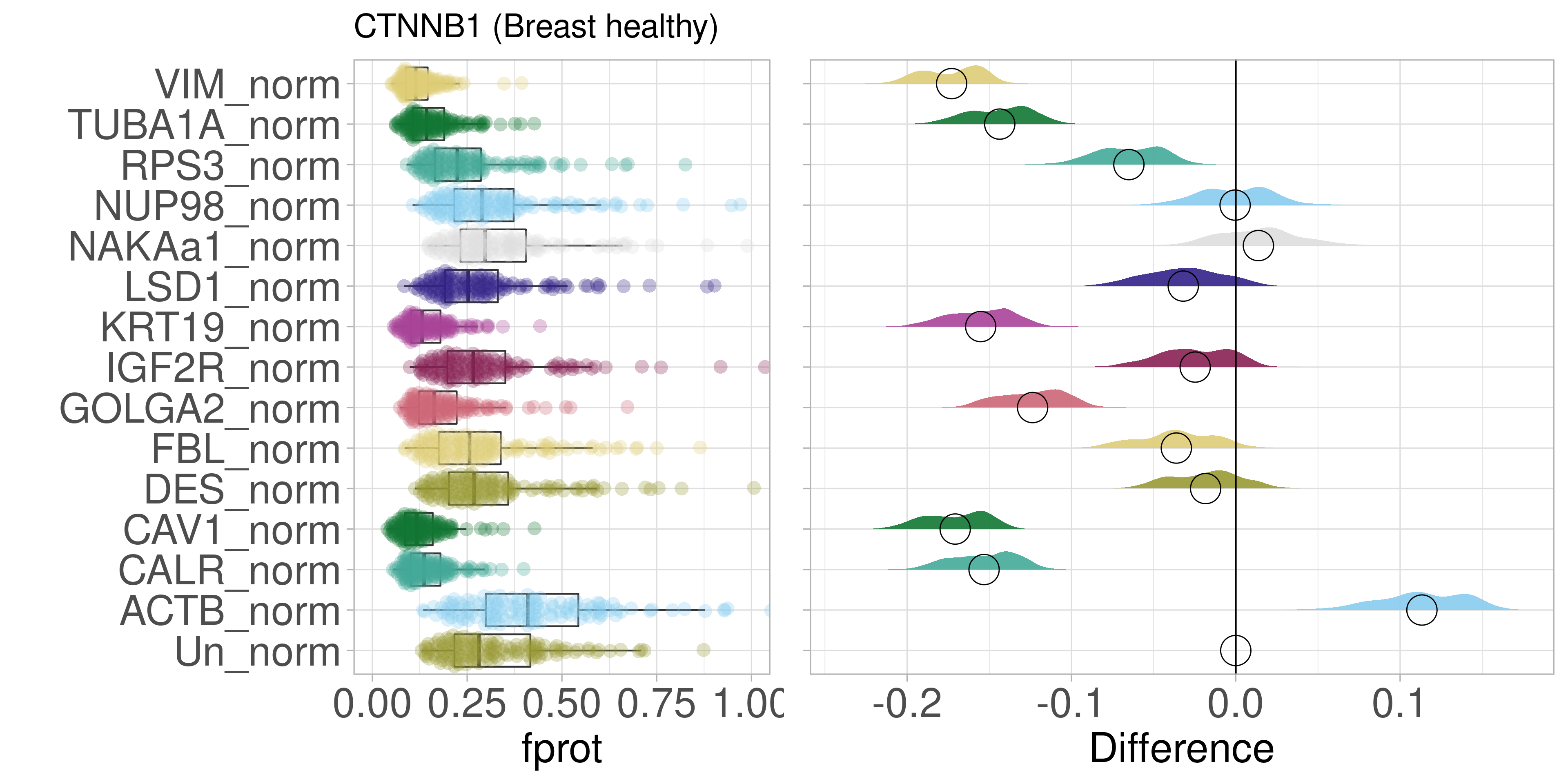

Supplement: Supplementary file 17 — Supplementary Material 17 [file 41598_2026_48754_MOESM17_ESM.zip › RPPA normalizations to cell markers/Breast_Plots/Oncoproteins_breast/CTNNB1_Breast_H.png]

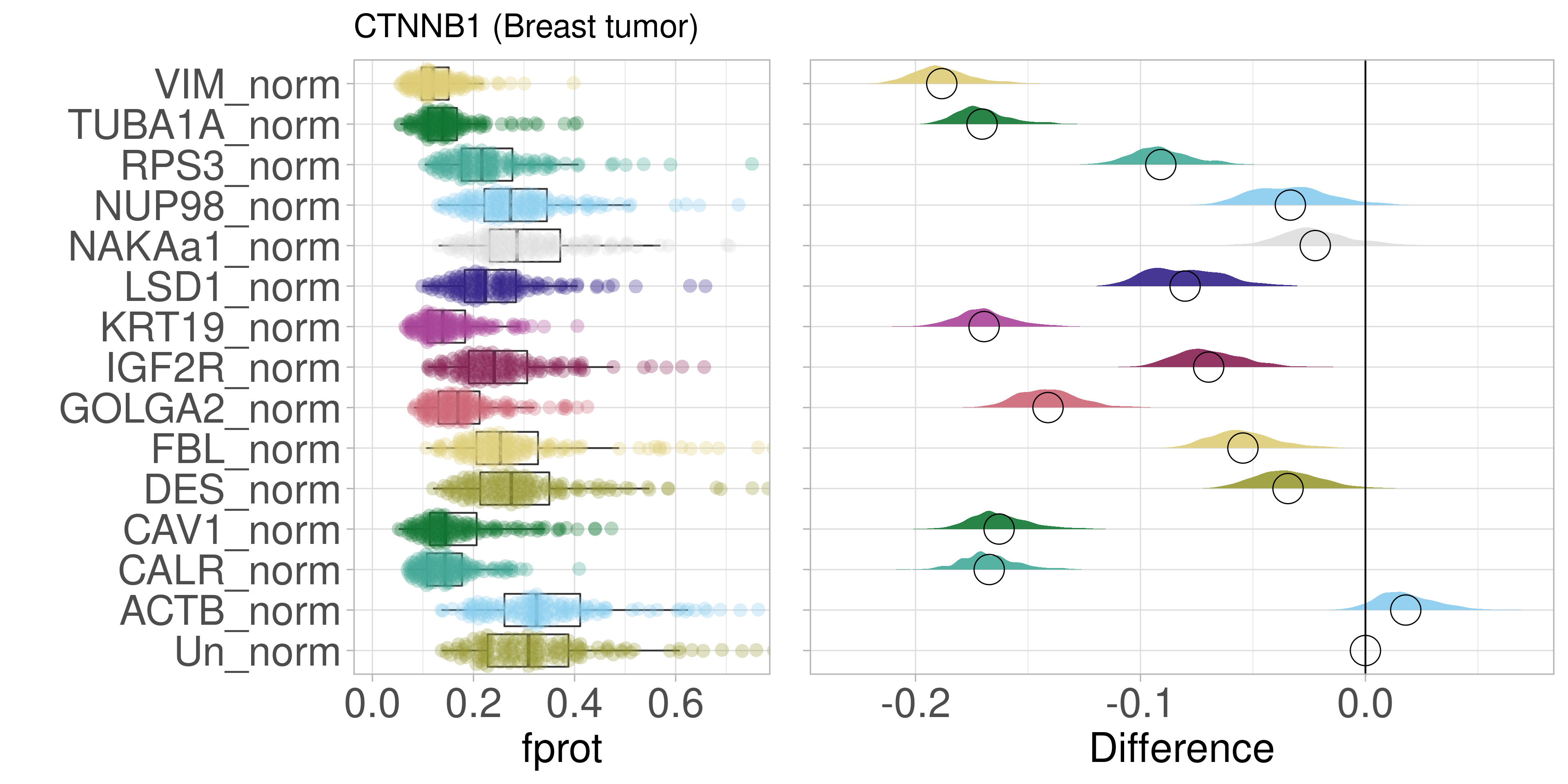

Supplement: Supplementary file 17 — Supplementary Material 17 [file 41598_2026_48754_MOESM17_ESM.zip › RPPA normalizations to cell markers/Breast_Plots/Oncoproteins_breast/CTNNB1_Breast_T.png]

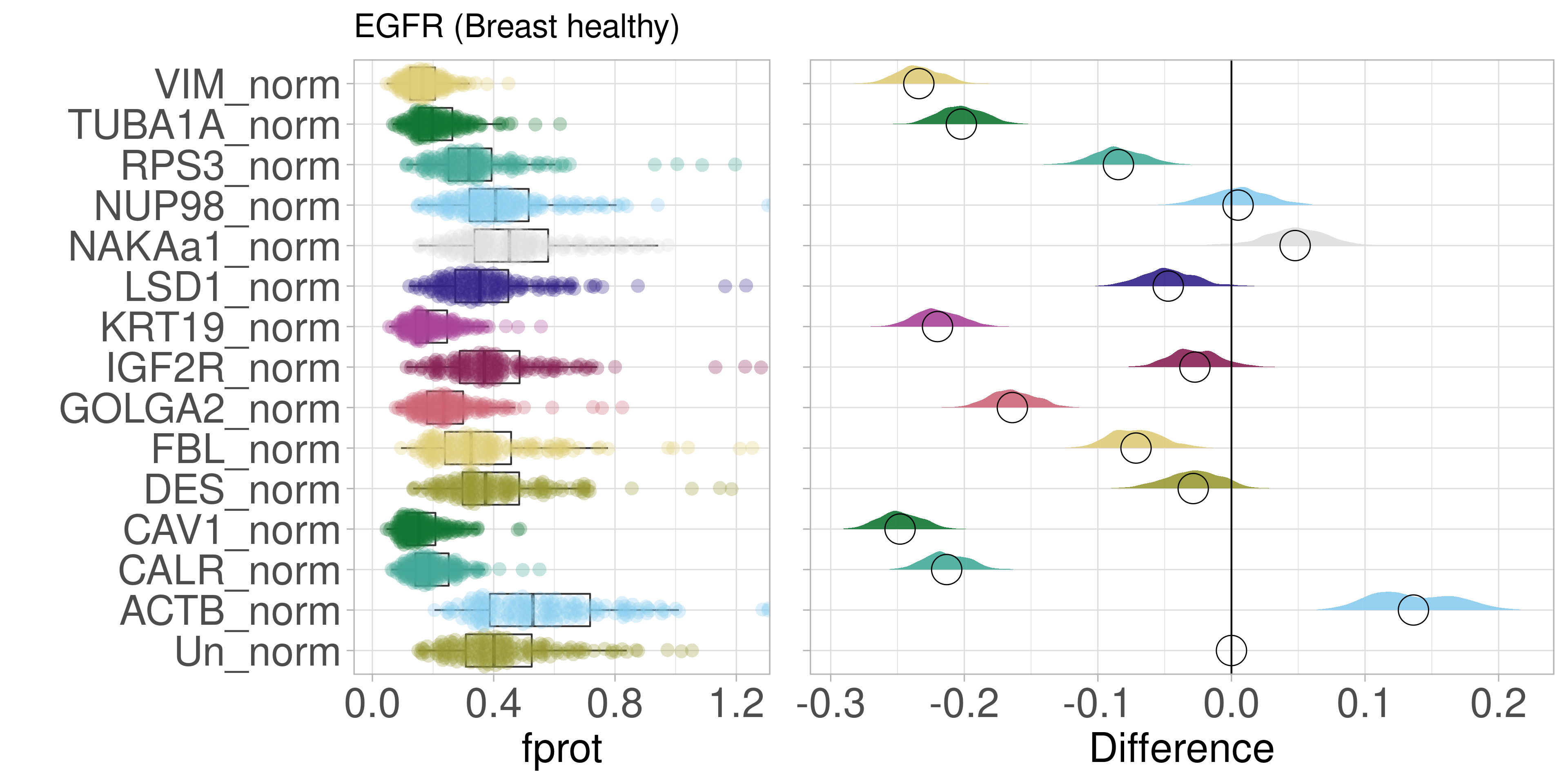

Supplement: Supplementary file 17 — Supplementary Material 17 [file 41598_2026_48754_MOESM17_ESM.zip › RPPA normalizations to cell markers/Breast_Plots/Oncoproteins_breast/EGFR_Breast_H.png]

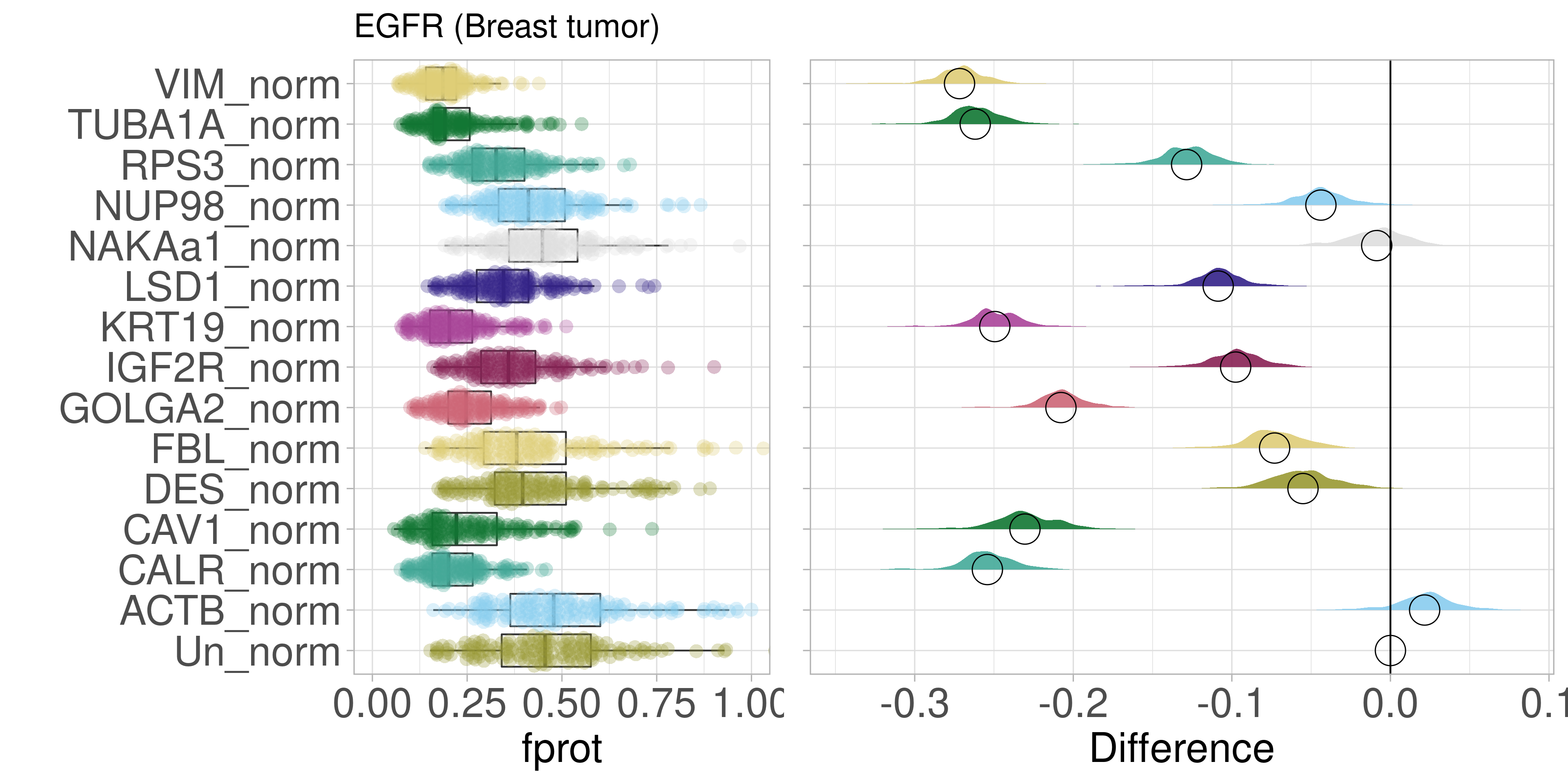

Supplement: Supplementary file 17 — Supplementary Material 17 [file 41598_2026_48754_MOESM17_ESM.zip › RPPA normalizations to cell markers/Breast_Plots/Oncoproteins_breast/EGFR_Breast_T.png]

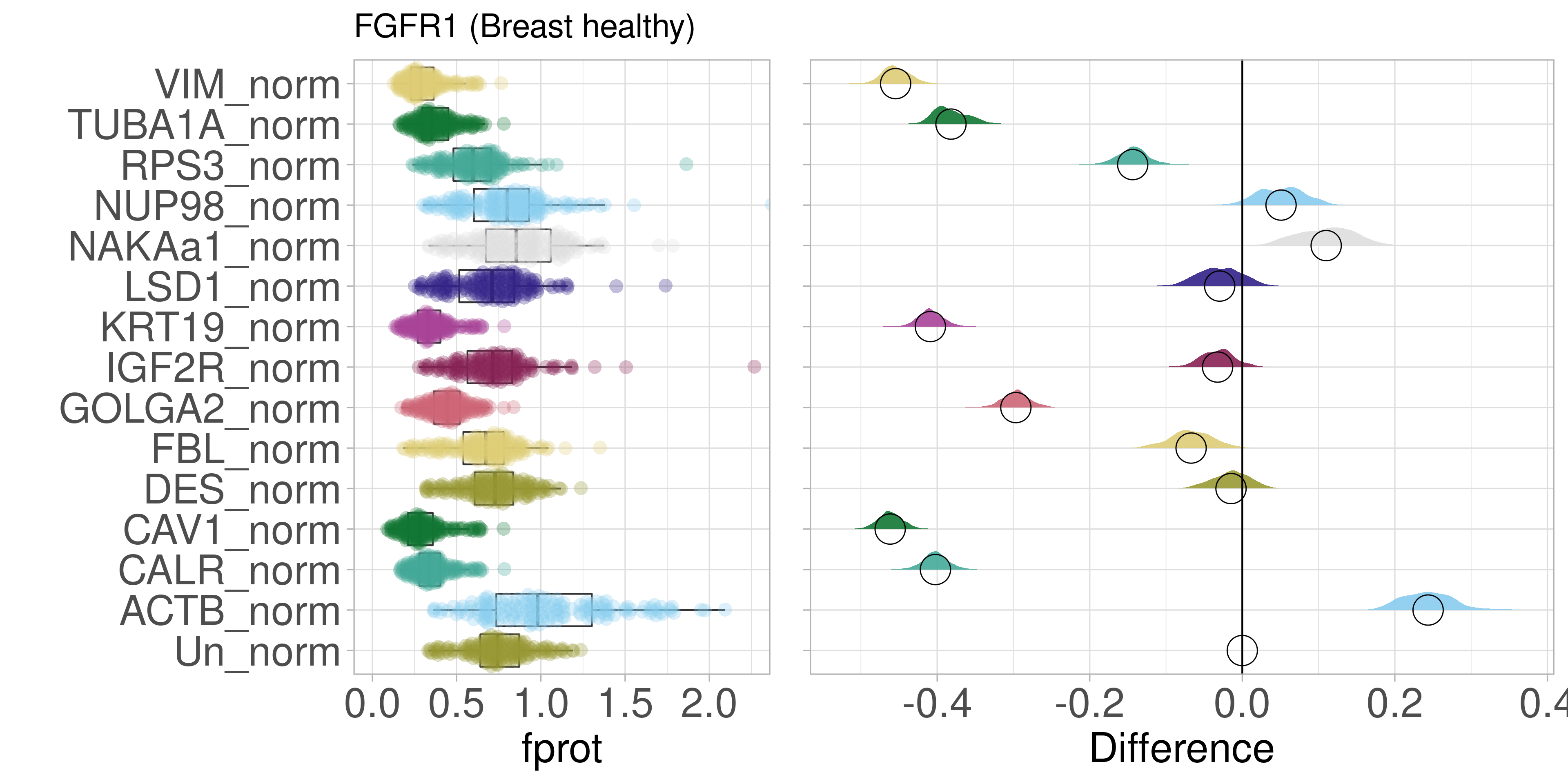

Supplement: Supplementary file 17 — Supplementary Material 17 [file 41598_2026_48754_MOESM17_ESM.zip › RPPA normalizations to cell markers/Breast_Plots/Oncoproteins_breast/FGFR1_Breast_H.png]

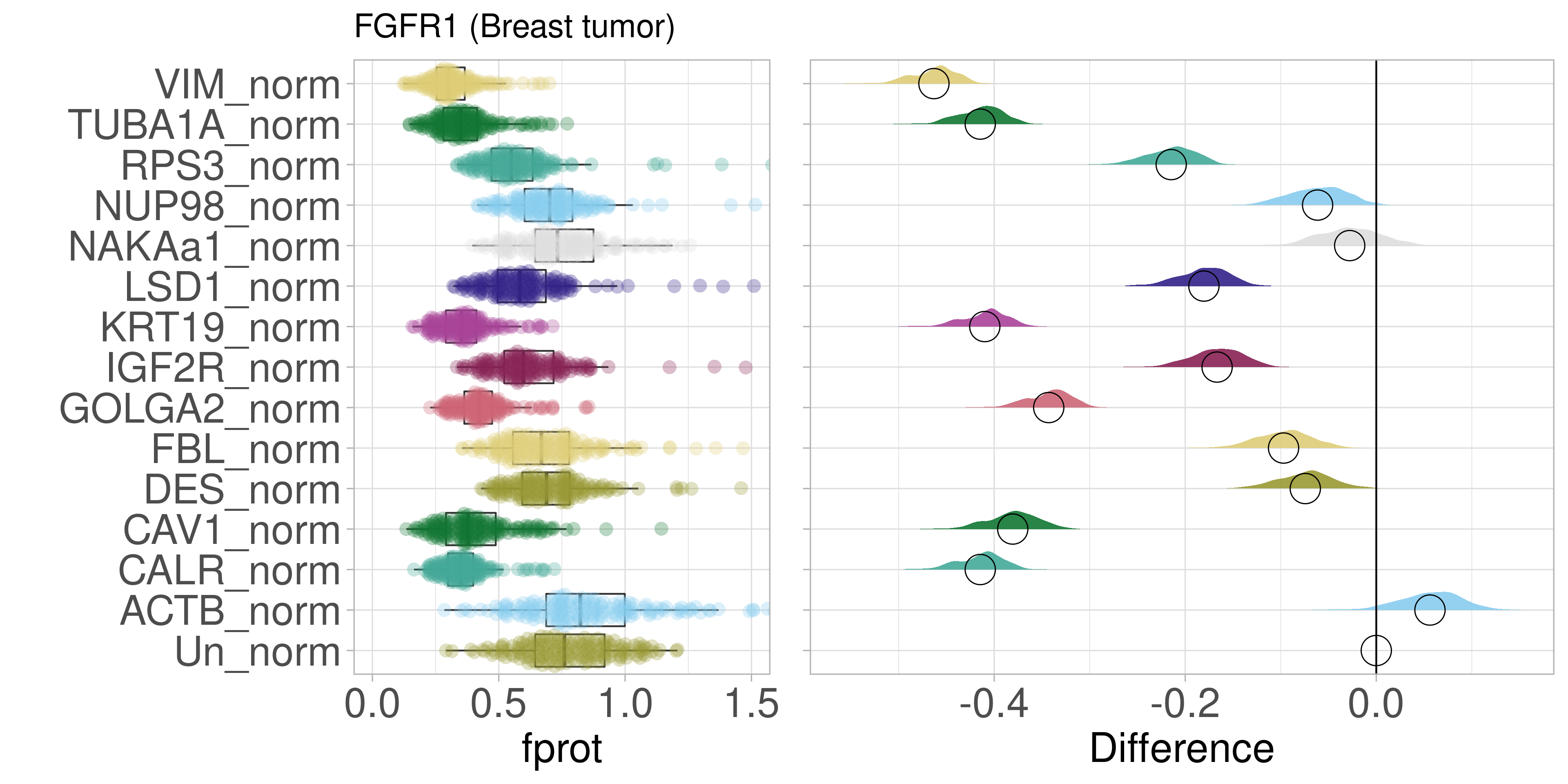

Supplement: Supplementary file 17 — Supplementary Material 17 [file 41598_2026_48754_MOESM17_ESM.zip › RPPA normalizations to cell markers/Breast_Plots/Oncoproteins_breast/FGFR1_Breast_T.png]

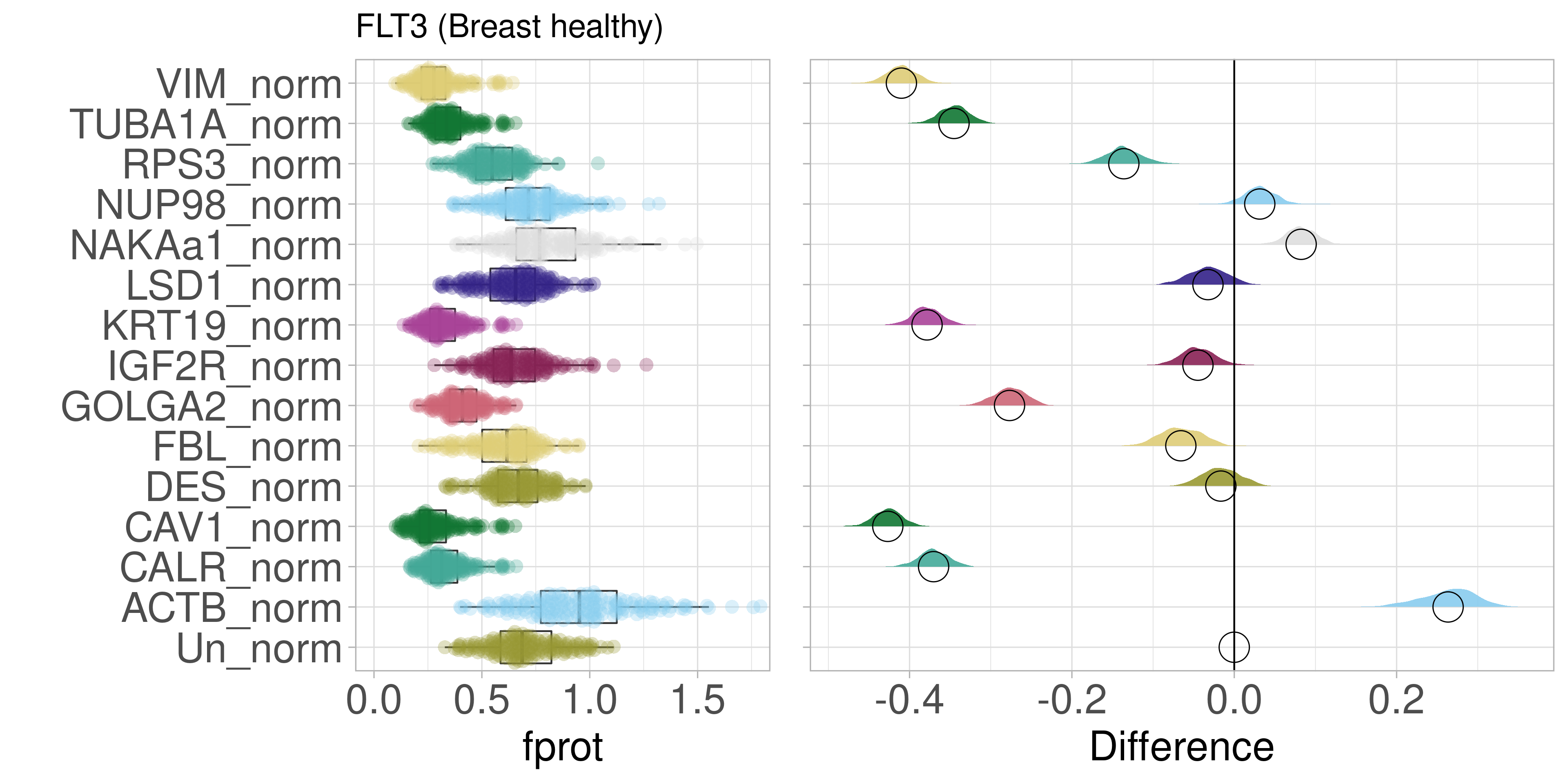

Supplement: Supplementary file 17 — Supplementary Material 17 [file 41598_2026_48754_MOESM17_ESM.zip › RPPA normalizations to cell markers/Breast_Plots/Oncoproteins_breast/FLT3_Breast_H.png]

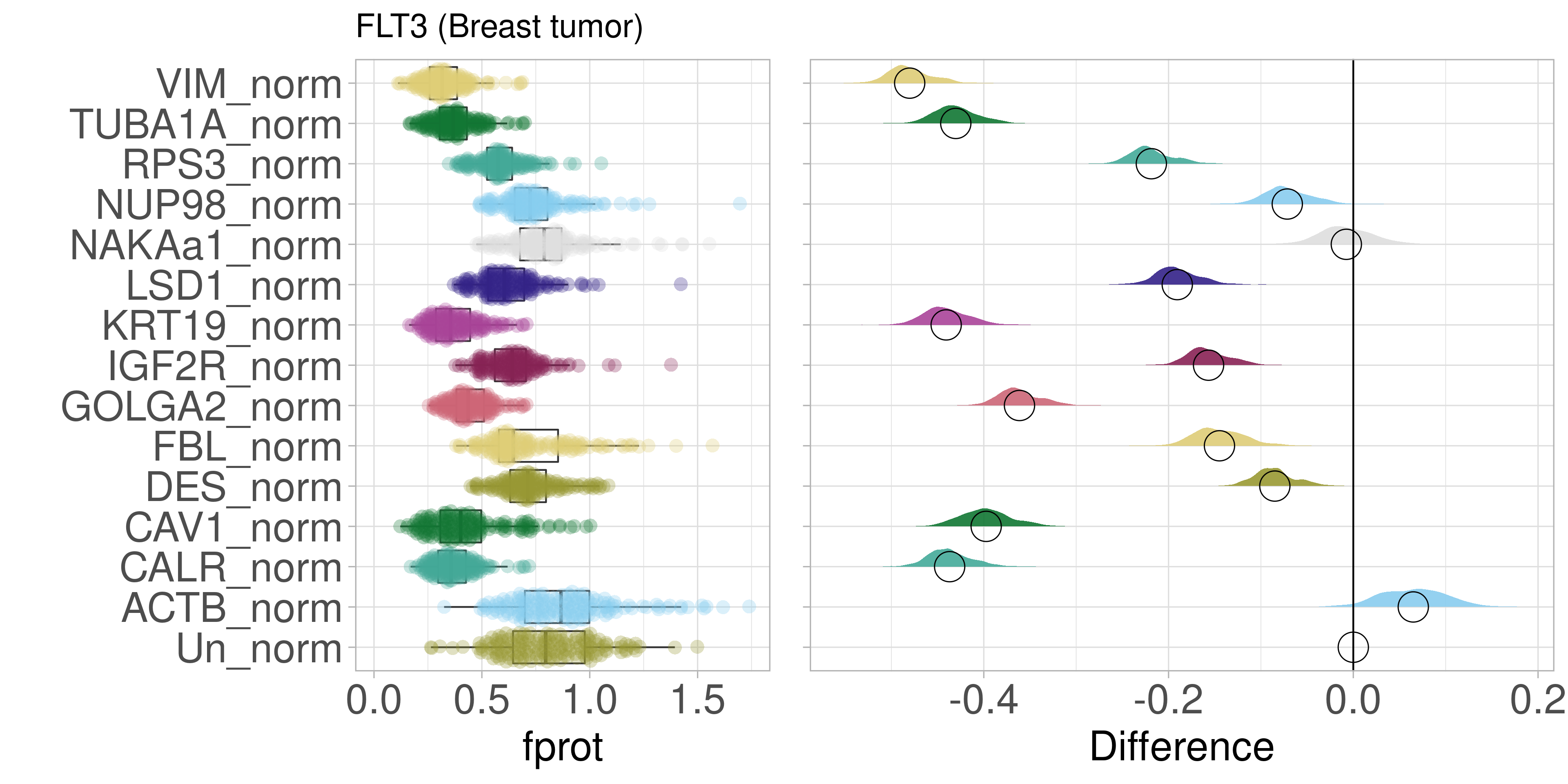

Supplement: Supplementary file 17 — Supplementary Material 17 [file 41598_2026_48754_MOESM17_ESM.zip › RPPA normalizations to cell markers/Breast_Plots/Oncoproteins_breast/FLT3_Breast_T.png]

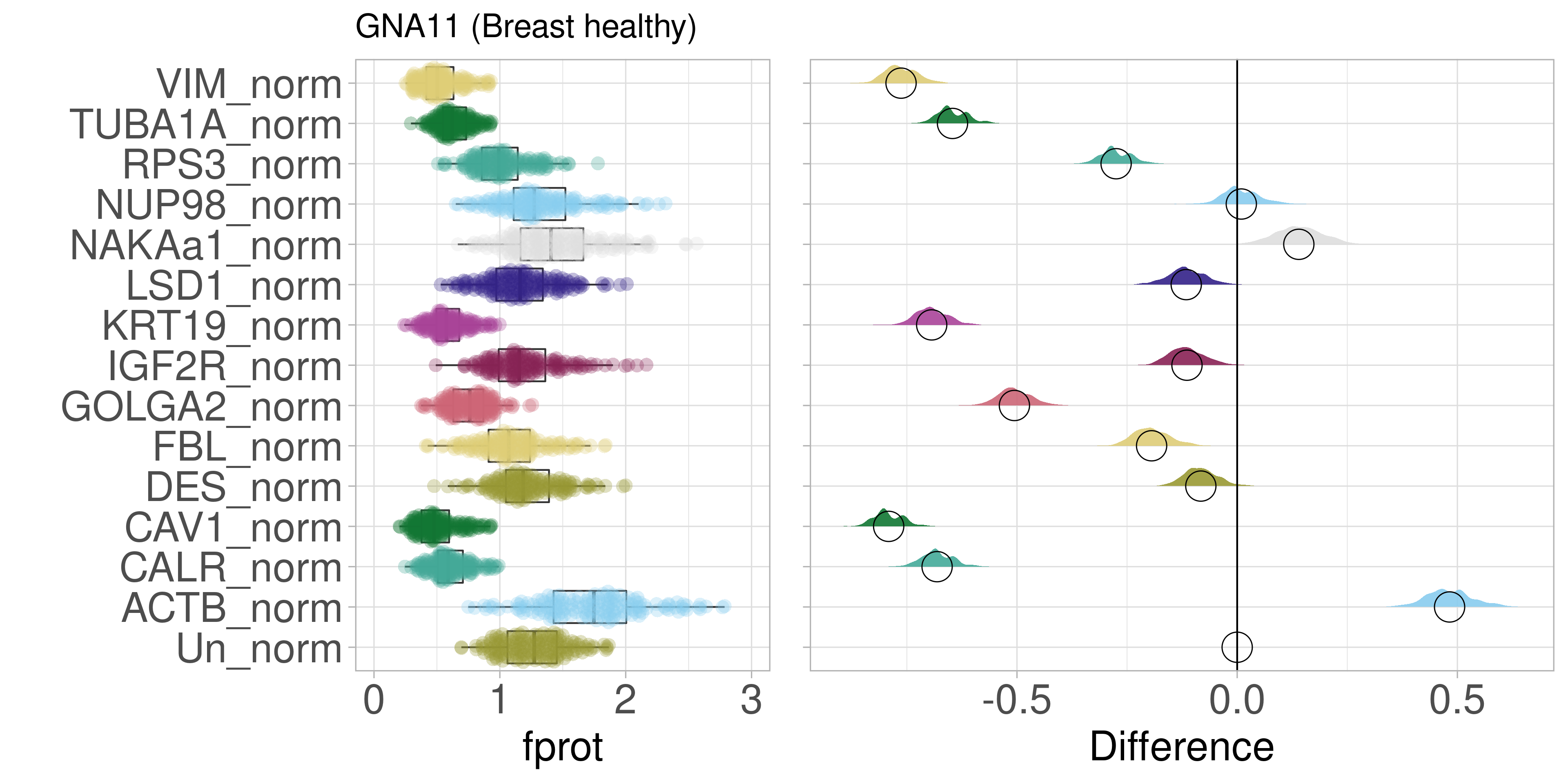

Supplement: Supplementary file 17 — Supplementary Material 17 [file 41598_2026_48754_MOESM17_ESM.zip › RPPA normalizations to cell markers/Breast_Plots/Oncoproteins_breast/GNA11_Breast_H.png]

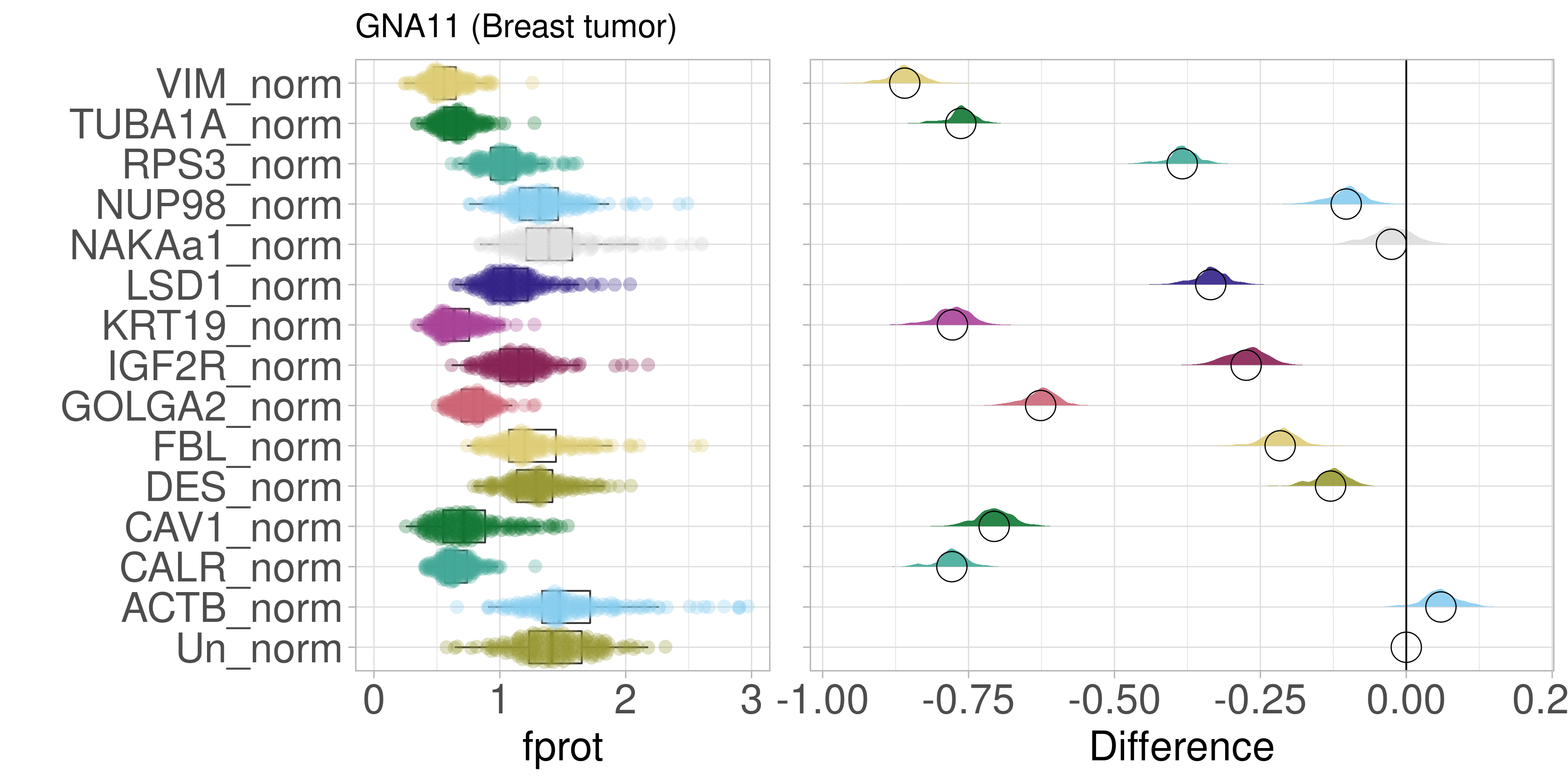

Supplement: Supplementary file 17 — Supplementary Material 17 [file 41598_2026_48754_MOESM17_ESM.zip › RPPA normalizations to cell markers/Breast_Plots/Oncoproteins_breast/GNA11_Breast_T.png]

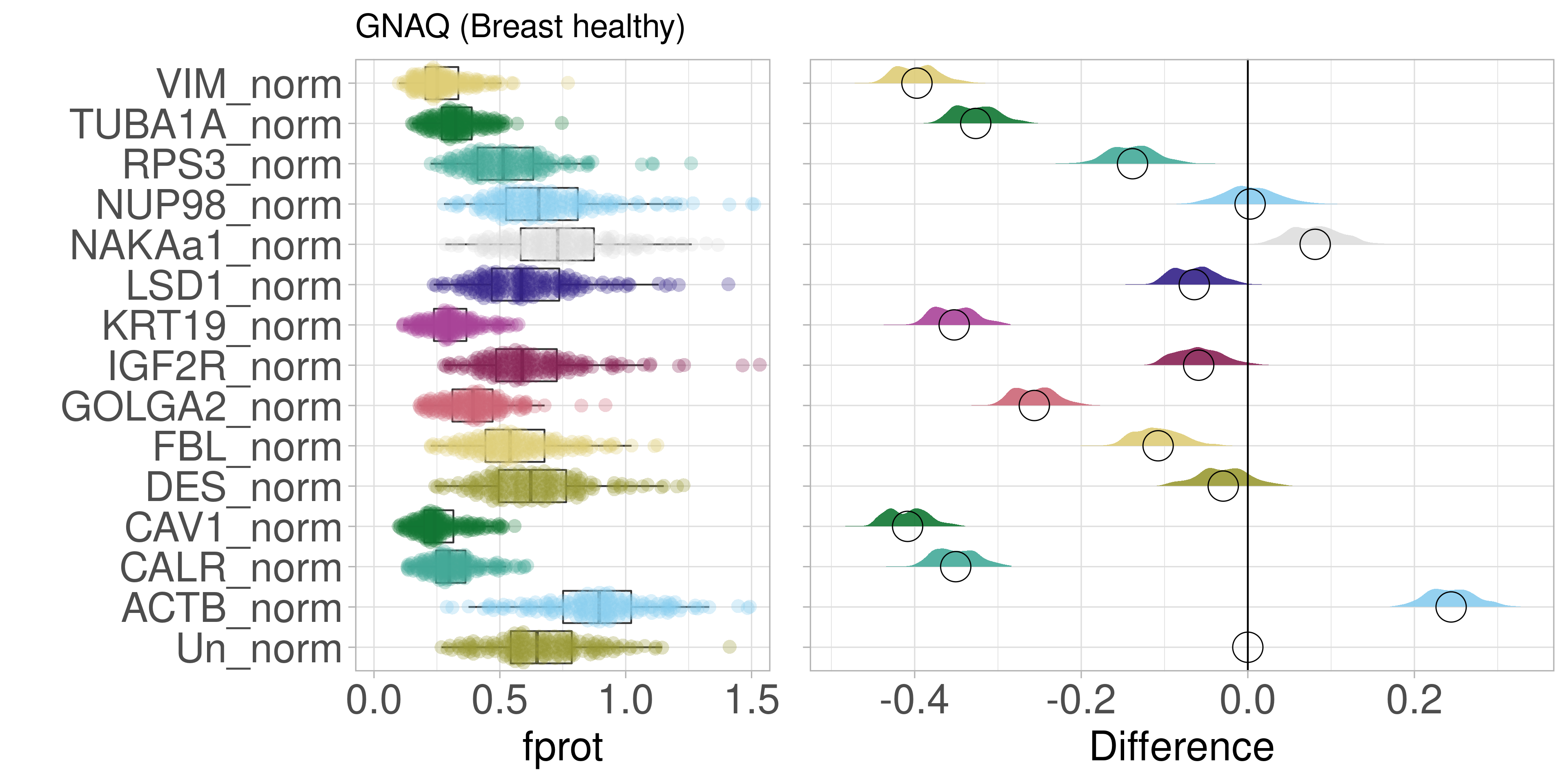

Supplement: Supplementary file 17 — Supplementary Material 17 [file 41598_2026_48754_MOESM17_ESM.zip › RPPA normalizations to cell markers/Breast_Plots/Oncoproteins_breast/GNAQ_Breast_H.png]

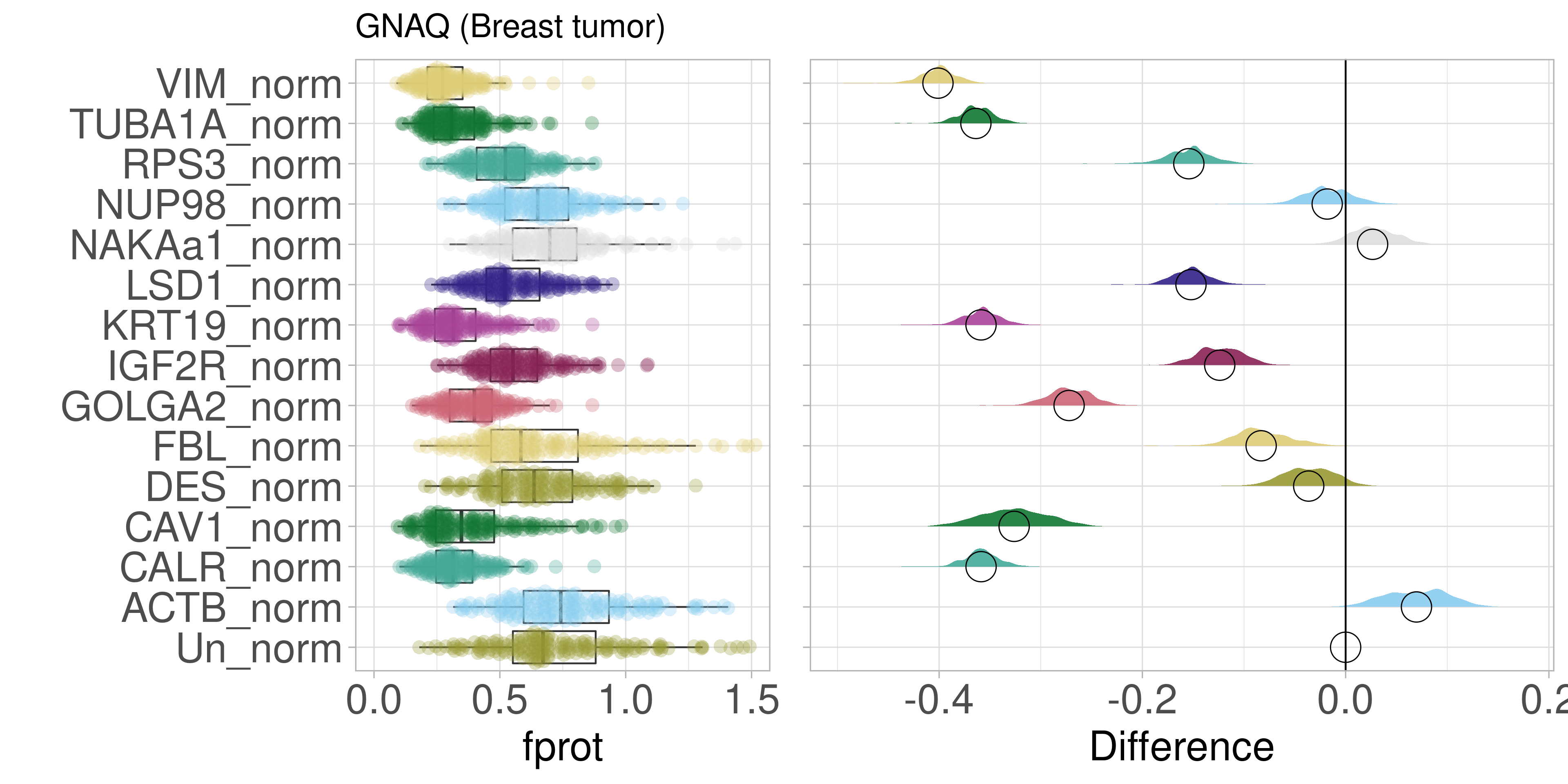

Supplement: Supplementary file 17 — Supplementary Material 17 [file 41598_2026_48754_MOESM17_ESM.zip › RPPA normalizations to cell markers/Breast_Plots/Oncoproteins_breast/GNAQ_Breast_T.png]

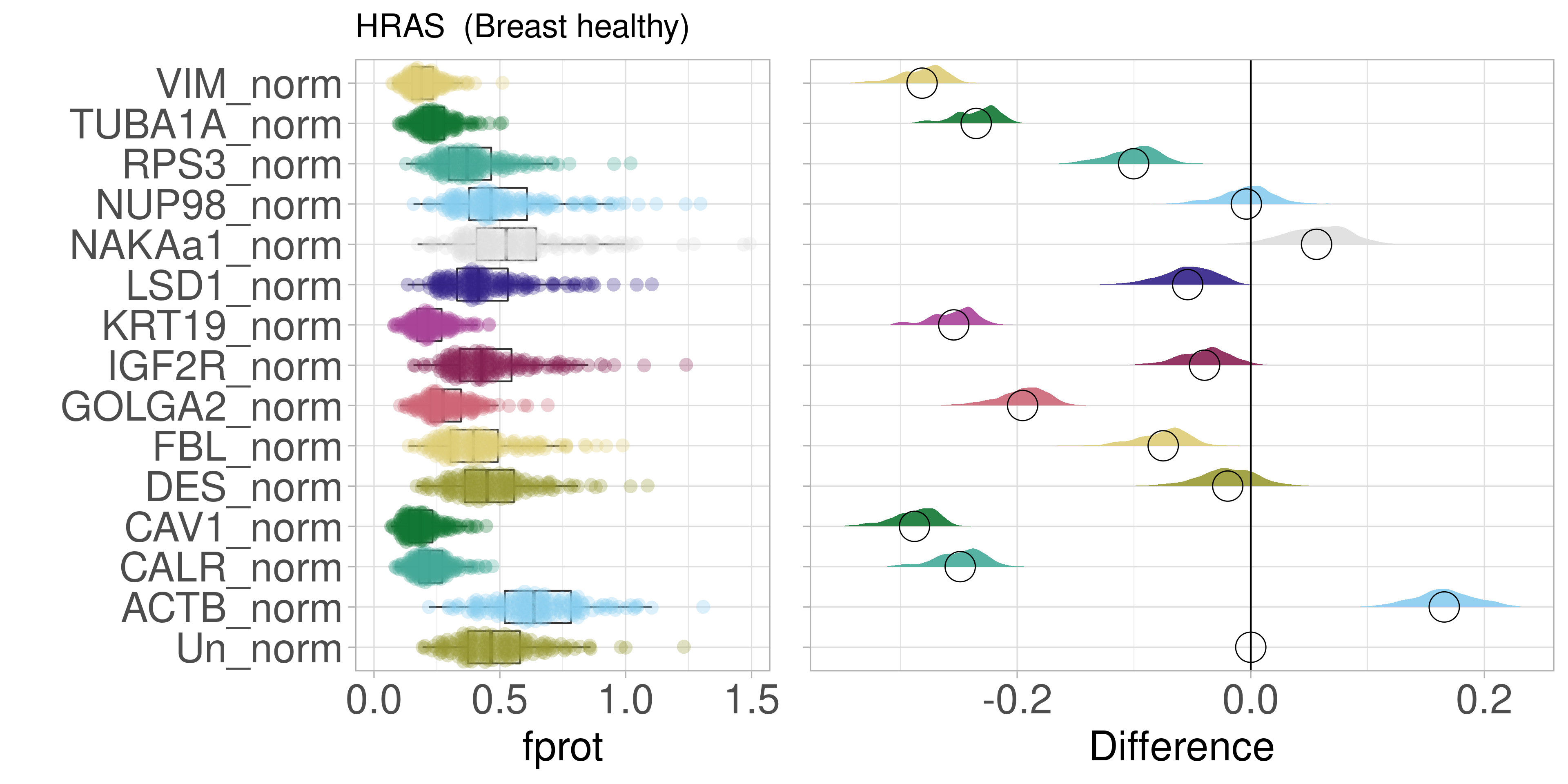

Supplement: Supplementary file 17 — Supplementary Material 17 [file 41598_2026_48754_MOESM17_ESM.zip › RPPA normalizations to cell markers/Breast_Plots/Oncoproteins_breast/HRAS_Breast_H.png]

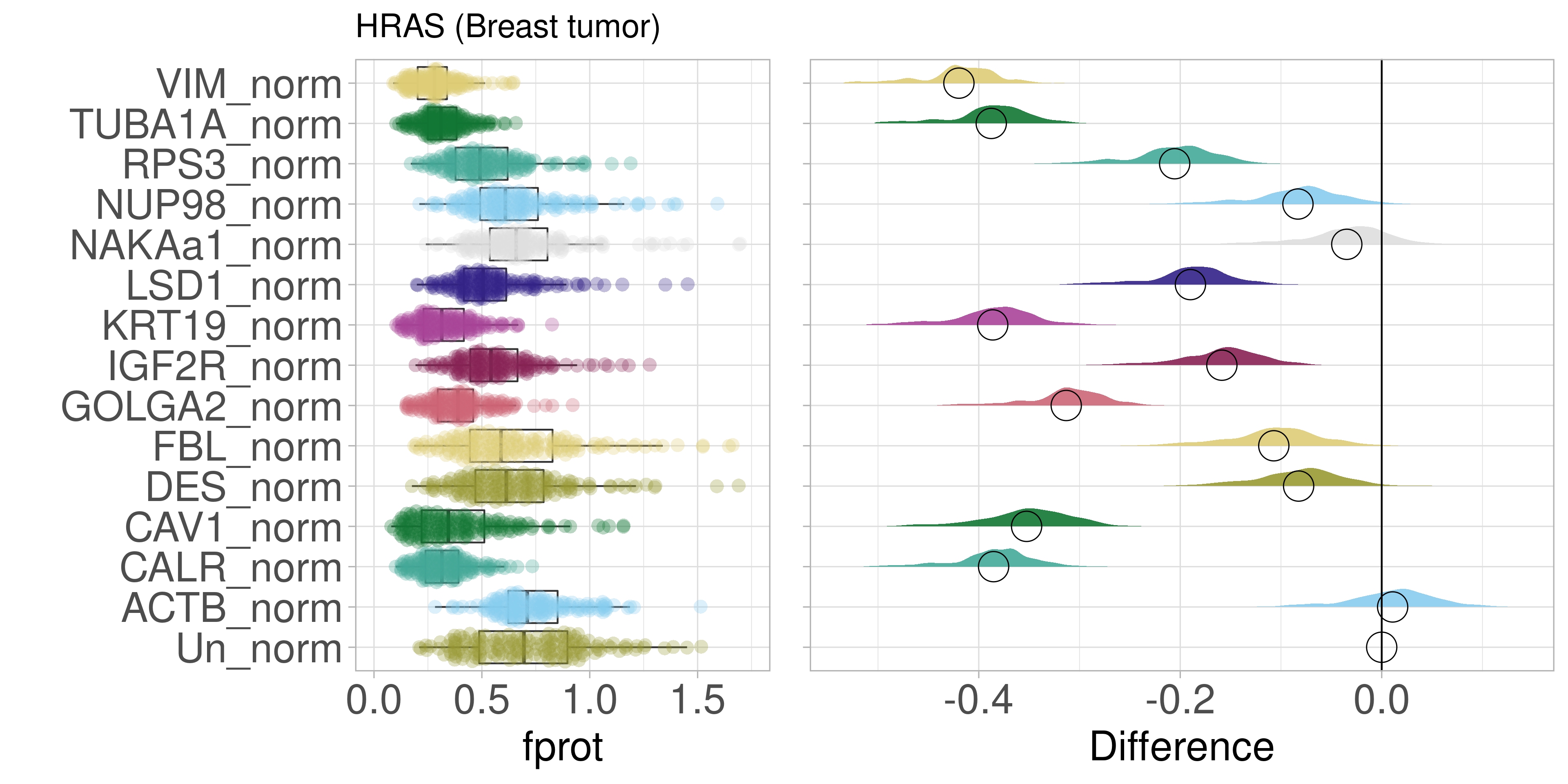

Supplement: Supplementary file 17 — Supplementary Material 17 [file 41598_2026_48754_MOESM17_ESM.zip › RPPA normalizations to cell markers/Breast_Plots/Oncoproteins_breast/HRAS_Breast_T.png]

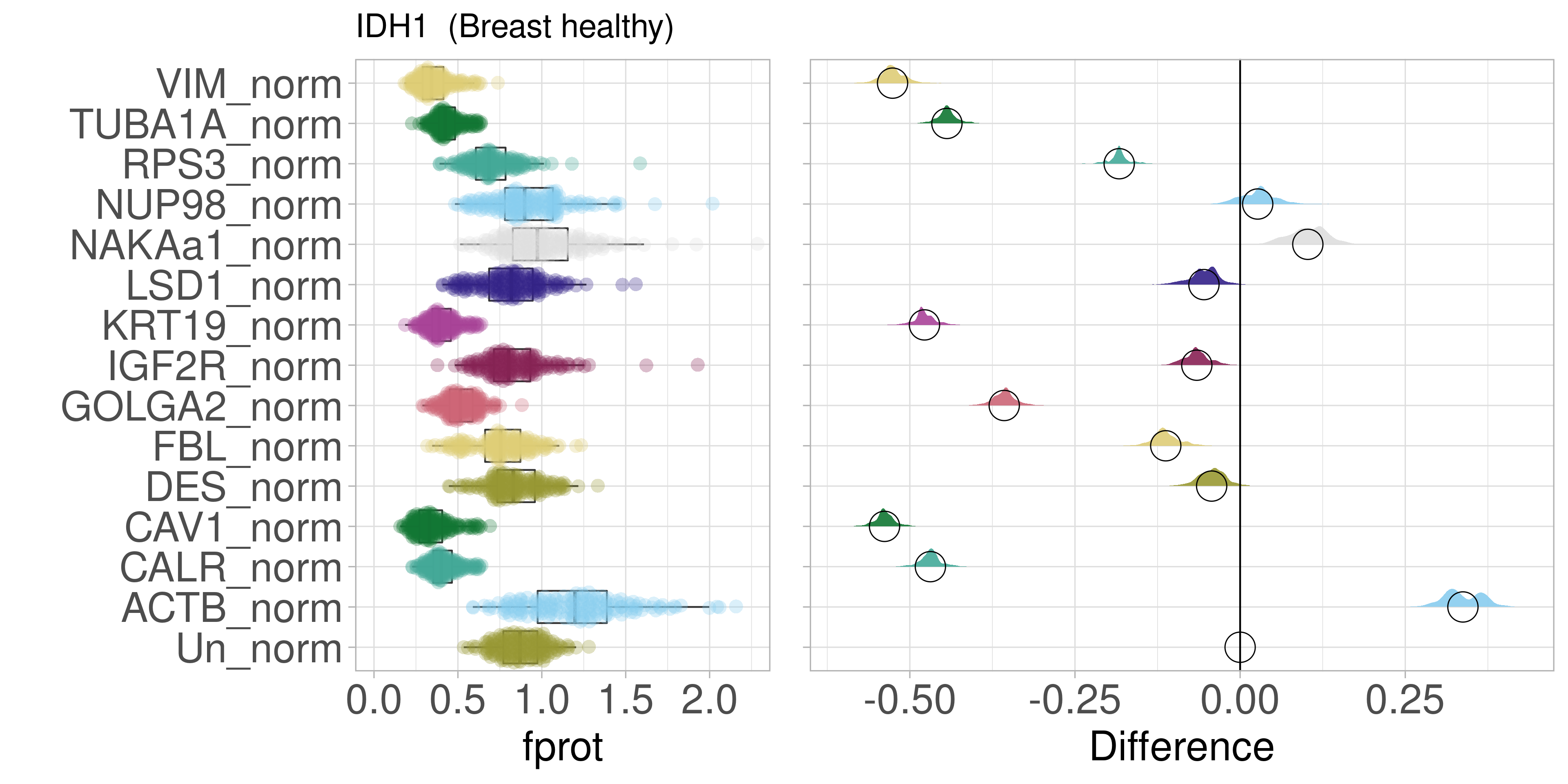

Supplement: Supplementary file 17 — Supplementary Material 17 [file 41598_2026_48754_MOESM17_ESM.zip › RPPA normalizations to cell markers/Breast_Plots/Oncoproteins_breast/IDH1_Breast_H.png]

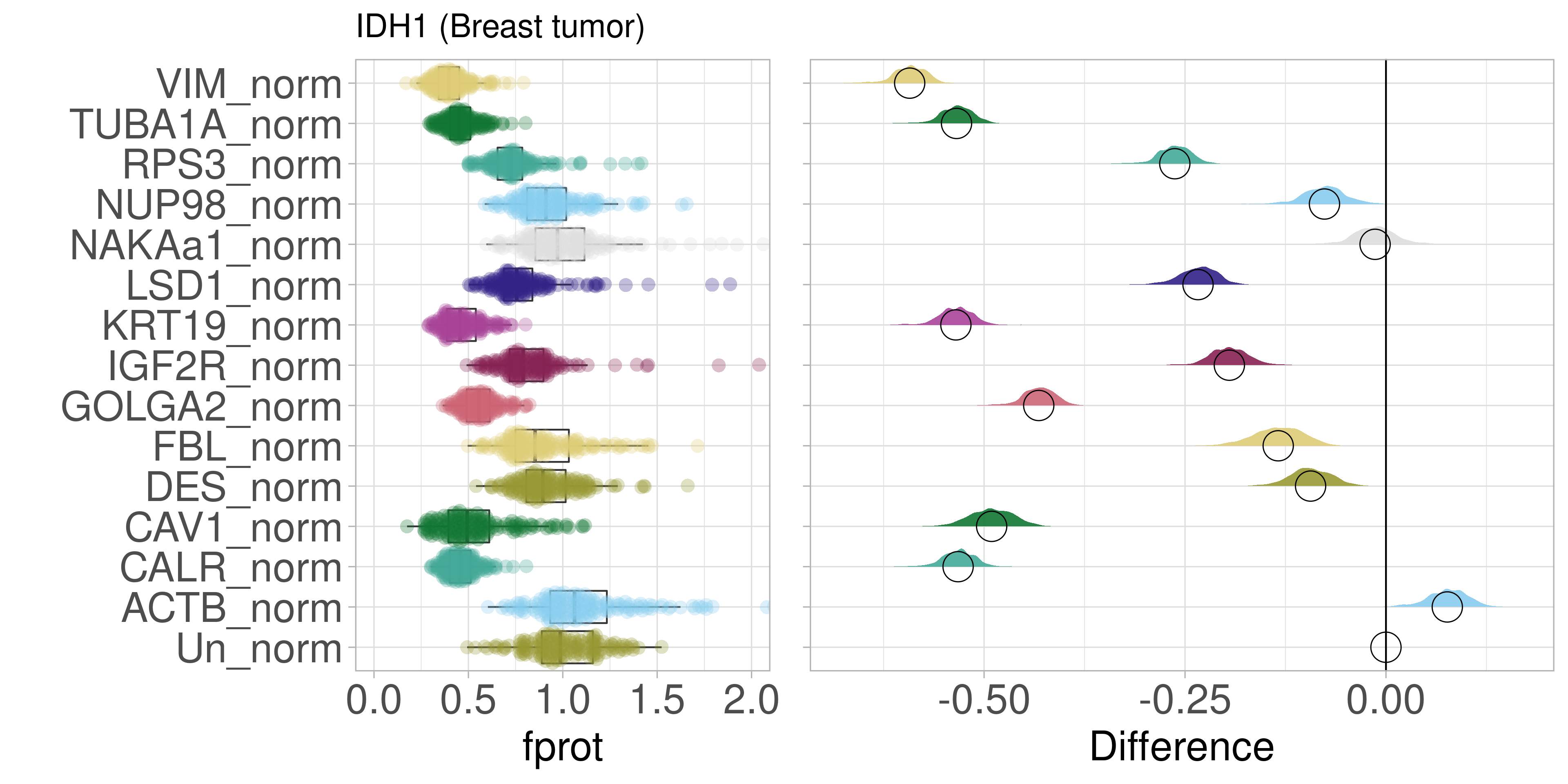

Supplement: Supplementary file 17 — Supplementary Material 17 [file 41598_2026_48754_MOESM17_ESM.zip › RPPA normalizations to cell markers/Breast_Plots/Oncoproteins_breast/IDH1_Breast_T.png]

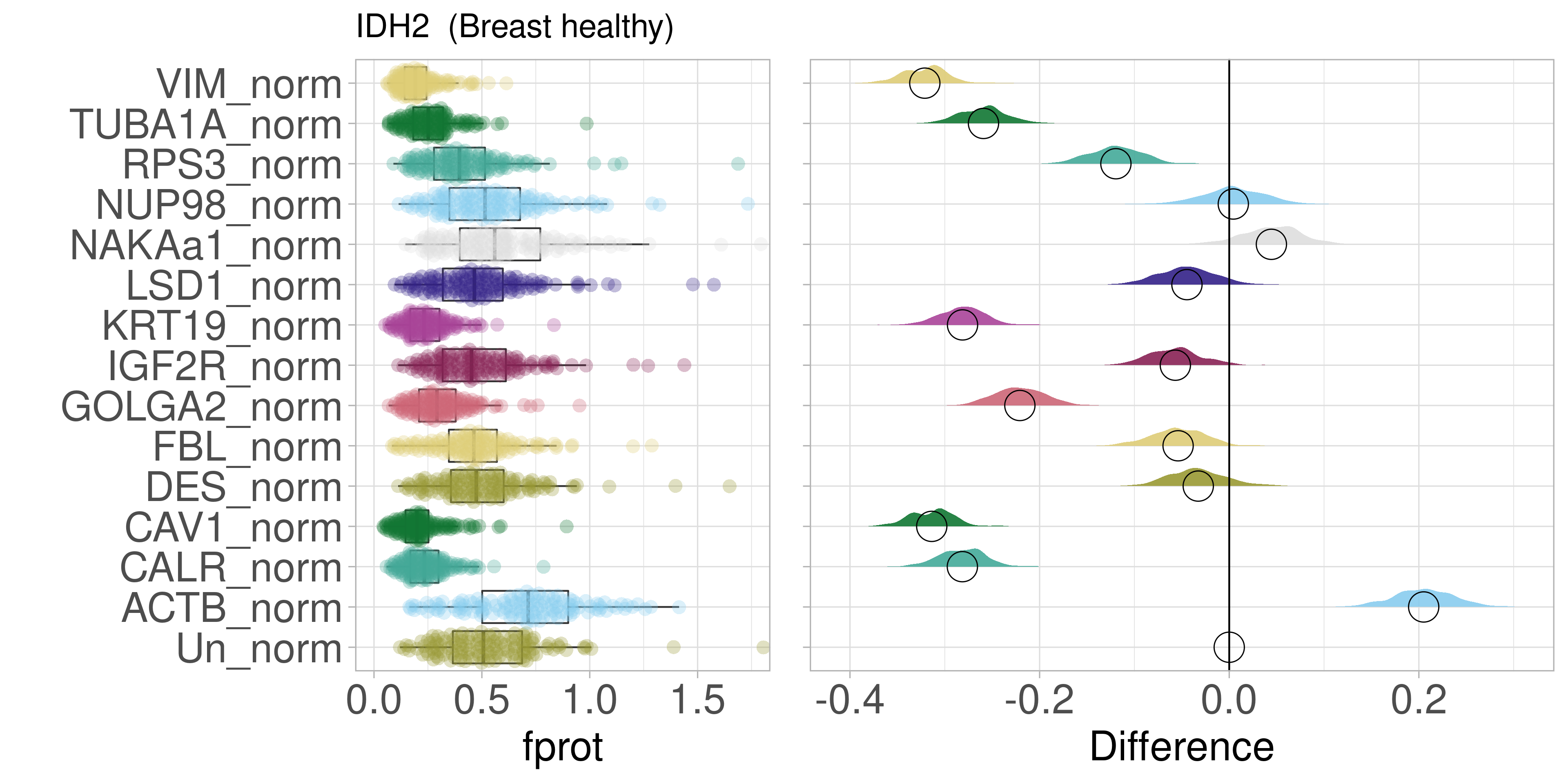

Supplement: Supplementary file 17 — Supplementary Material 17 [file 41598_2026_48754_MOESM17_ESM.zip › RPPA normalizations to cell markers/Breast_Plots/Oncoproteins_breast/IDH2_Breast_H.png]

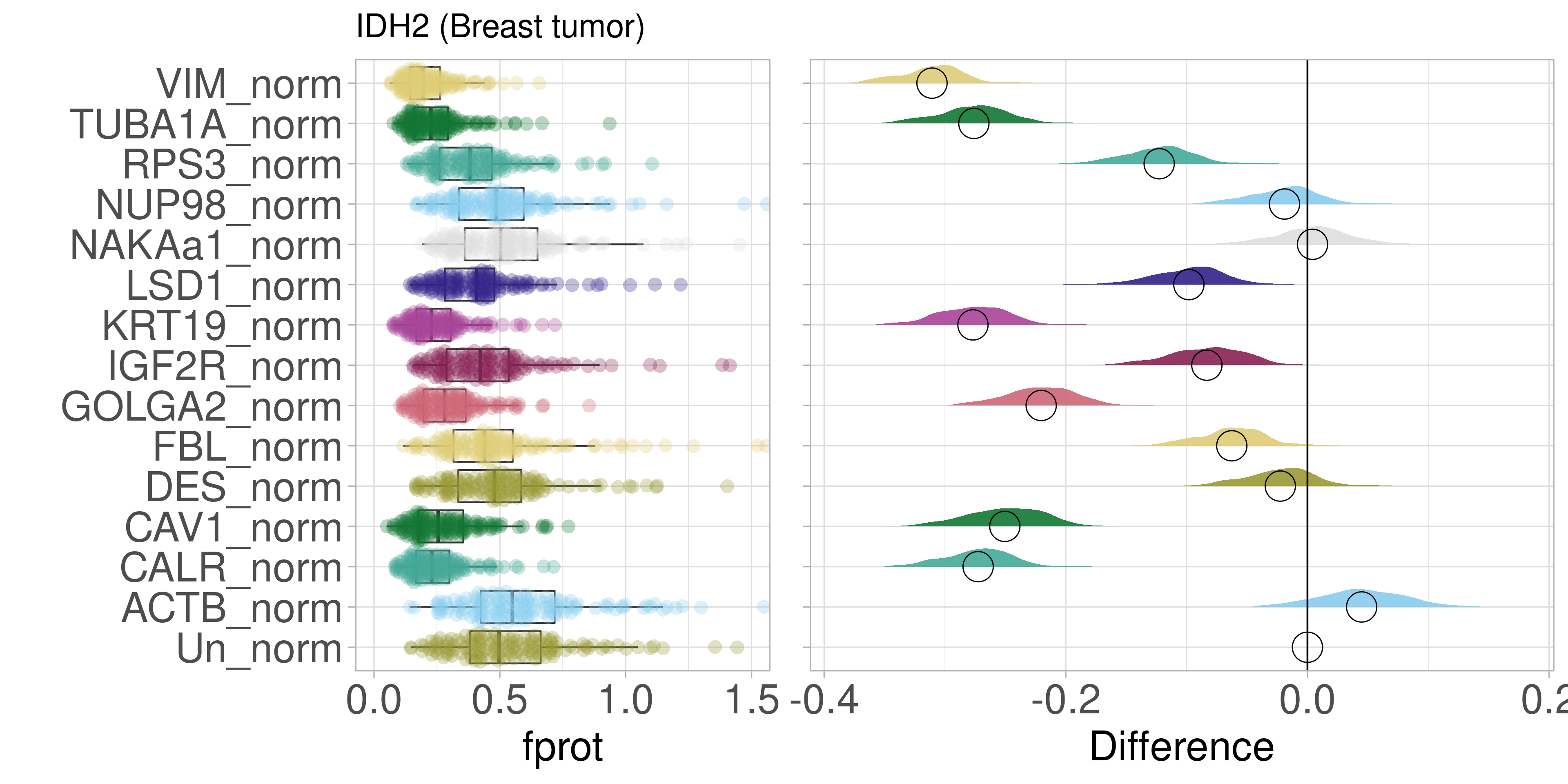

Supplement: Supplementary file 17 — Supplementary Material 17 [file 41598_2026_48754_MOESM17_ESM.zip › RPPA normalizations to cell markers/Breast_Plots/Oncoproteins_breast/IDH2_Breast_T.png]

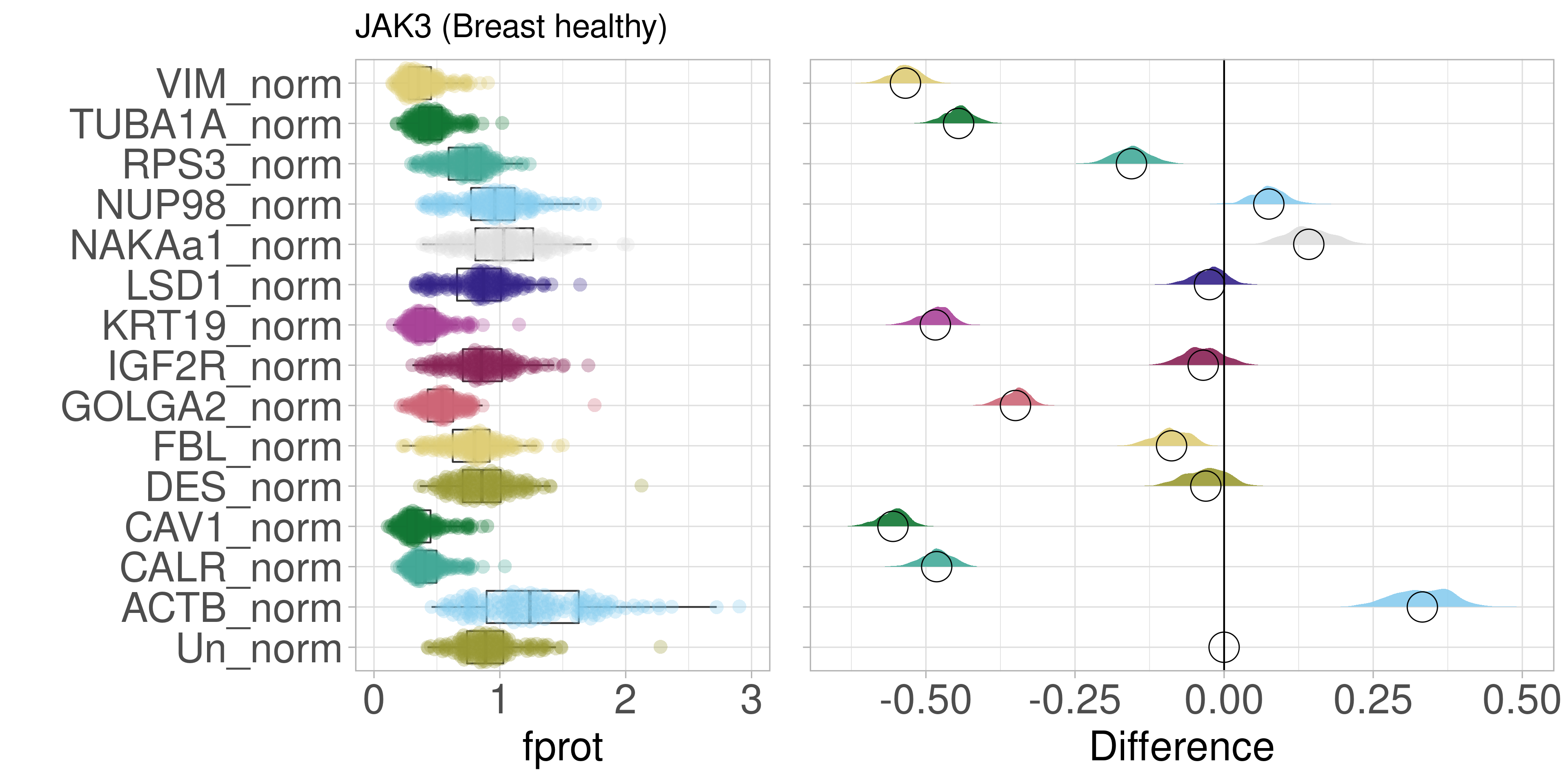

Supplement: Supplementary file 17 — Supplementary Material 17 [file 41598_2026_48754_MOESM17_ESM.zip › RPPA normalizations to cell markers/Breast_Plots/Oncoproteins_breast/JAK3_Breast_H.png]

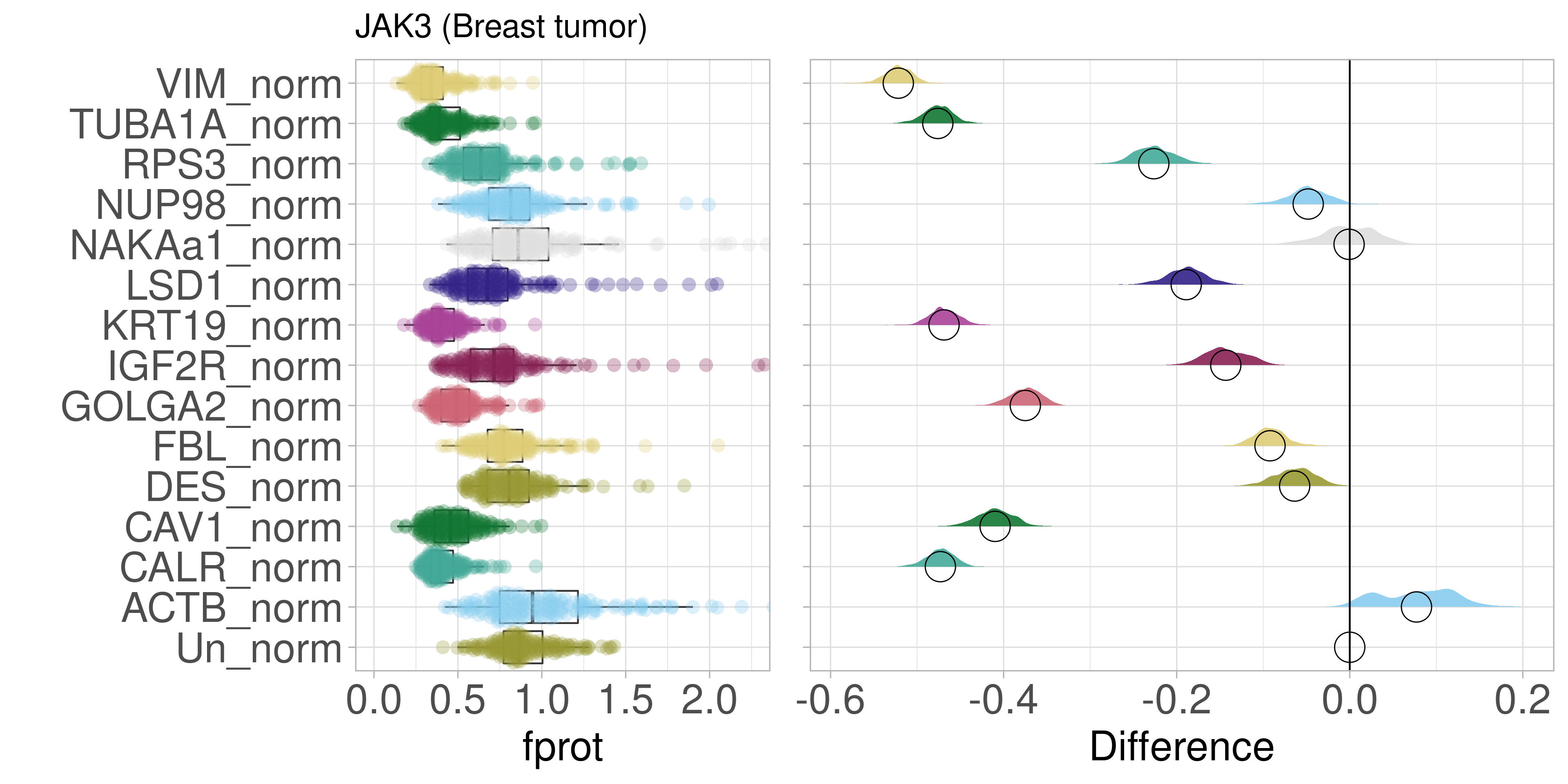

Supplement: Supplementary file 17 — Supplementary Material 17 [file 41598_2026_48754_MOESM17_ESM.zip › RPPA normalizations to cell markers/Breast_Plots/Oncoproteins_breast/JAK3_Breast_T.png]

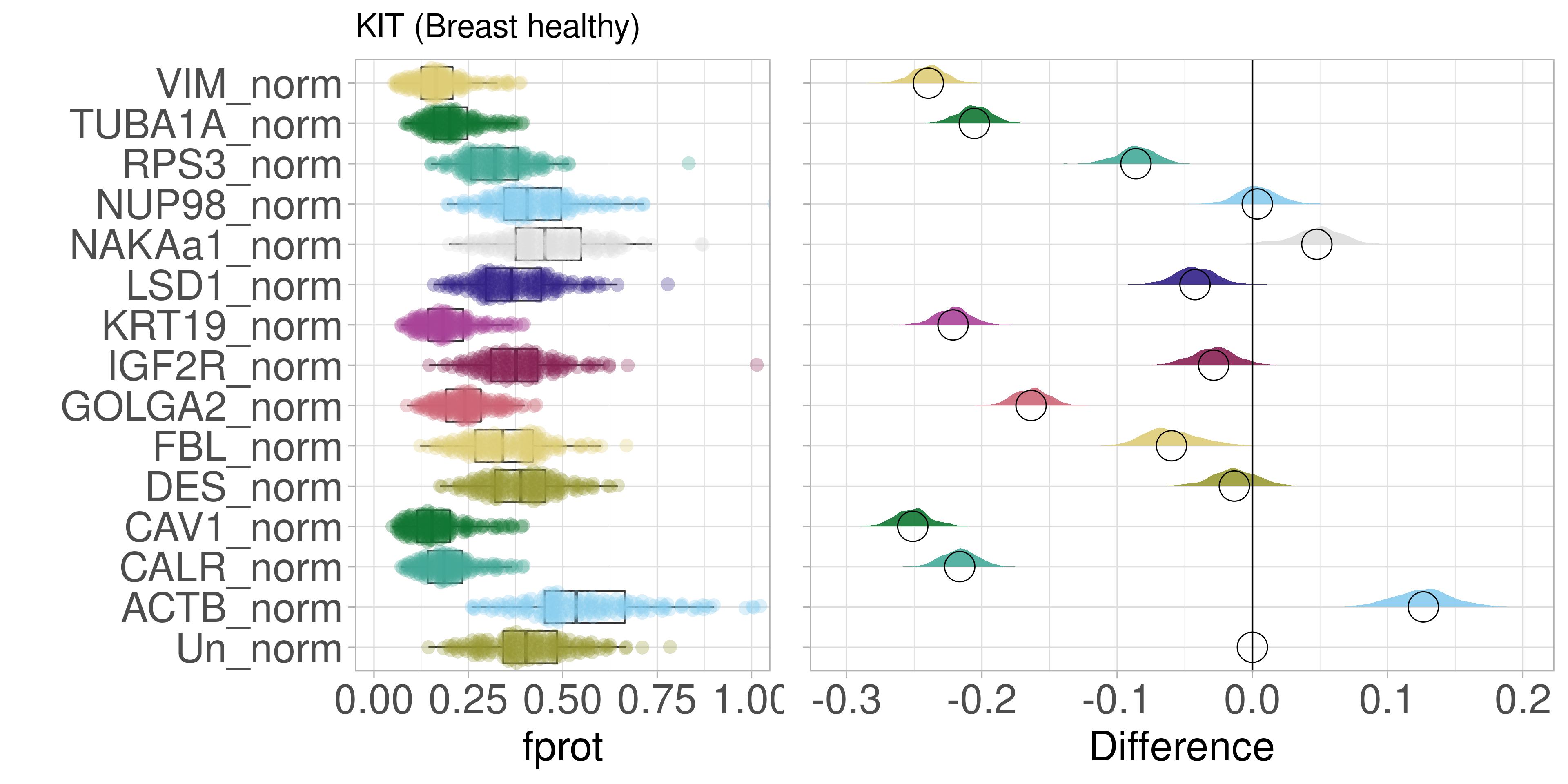

Supplement: Supplementary file 17 — Supplementary Material 17 [file 41598_2026_48754_MOESM17_ESM.zip › RPPA normalizations to cell markers/Breast_Plots/Oncoproteins_breast/KIT_Breast_H.png]

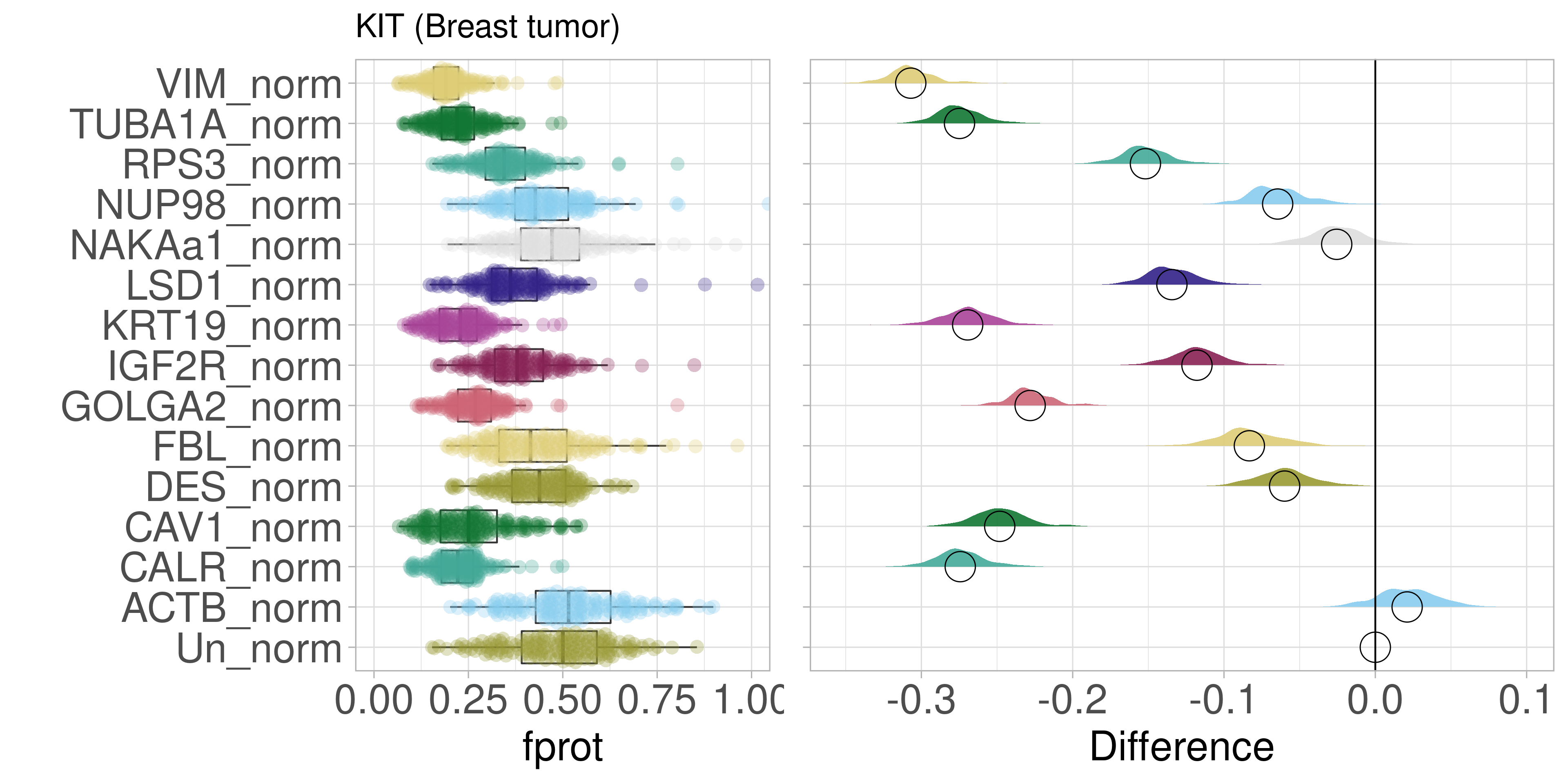

Supplement: Supplementary file 17 — Supplementary Material 17 [file 41598_2026_48754_MOESM17_ESM.zip › RPPA normalizations to cell markers/Breast_Plots/Oncoproteins_breast/KIT_Breast_T.png]

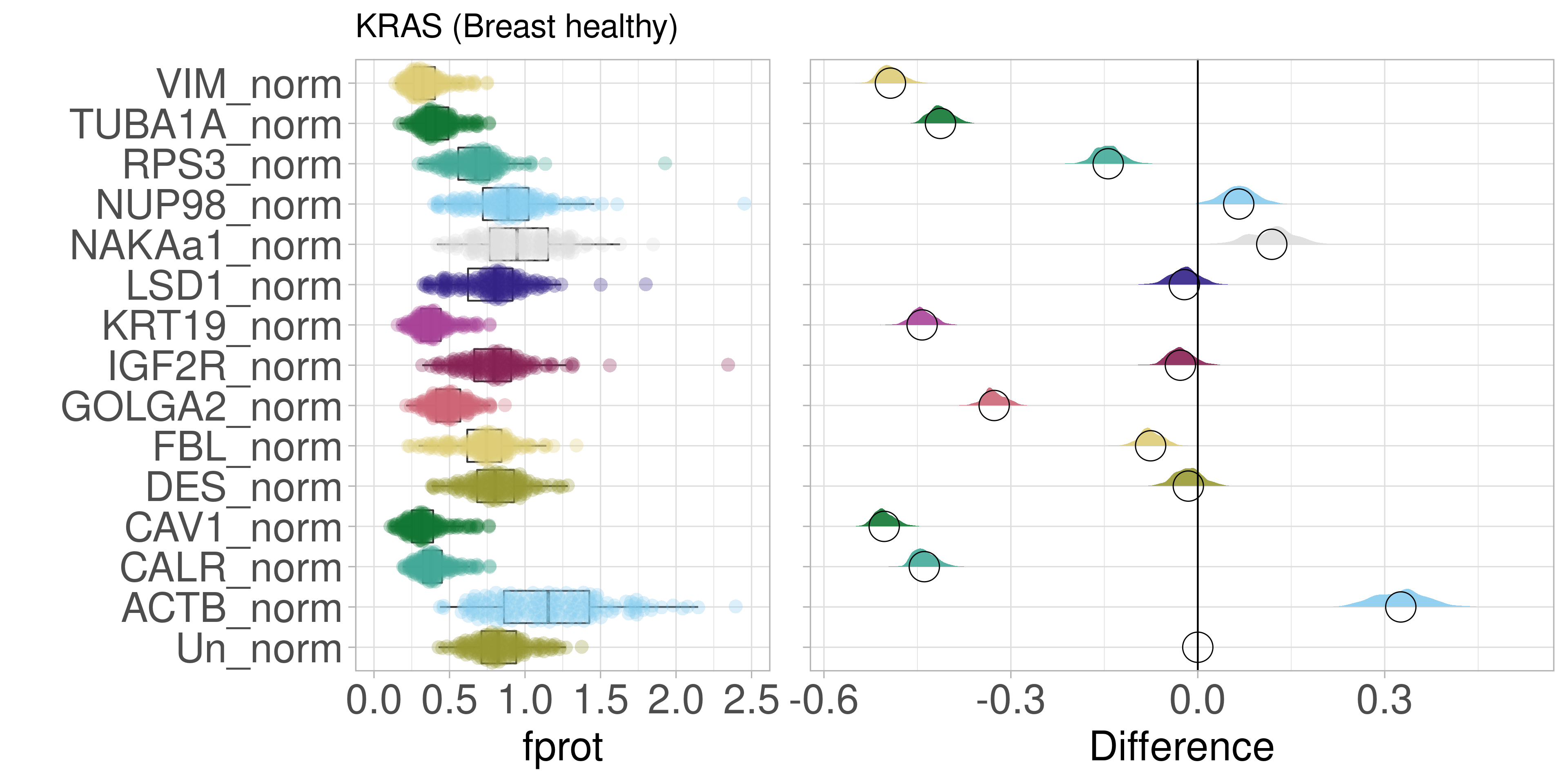

Supplement: Supplementary file 17 — Supplementary Material 17 [file 41598_2026_48754_MOESM17_ESM.zip › RPPA normalizations to cell markers/Breast_Plots/Oncoproteins_breast/KRAS_Breast_H.png]

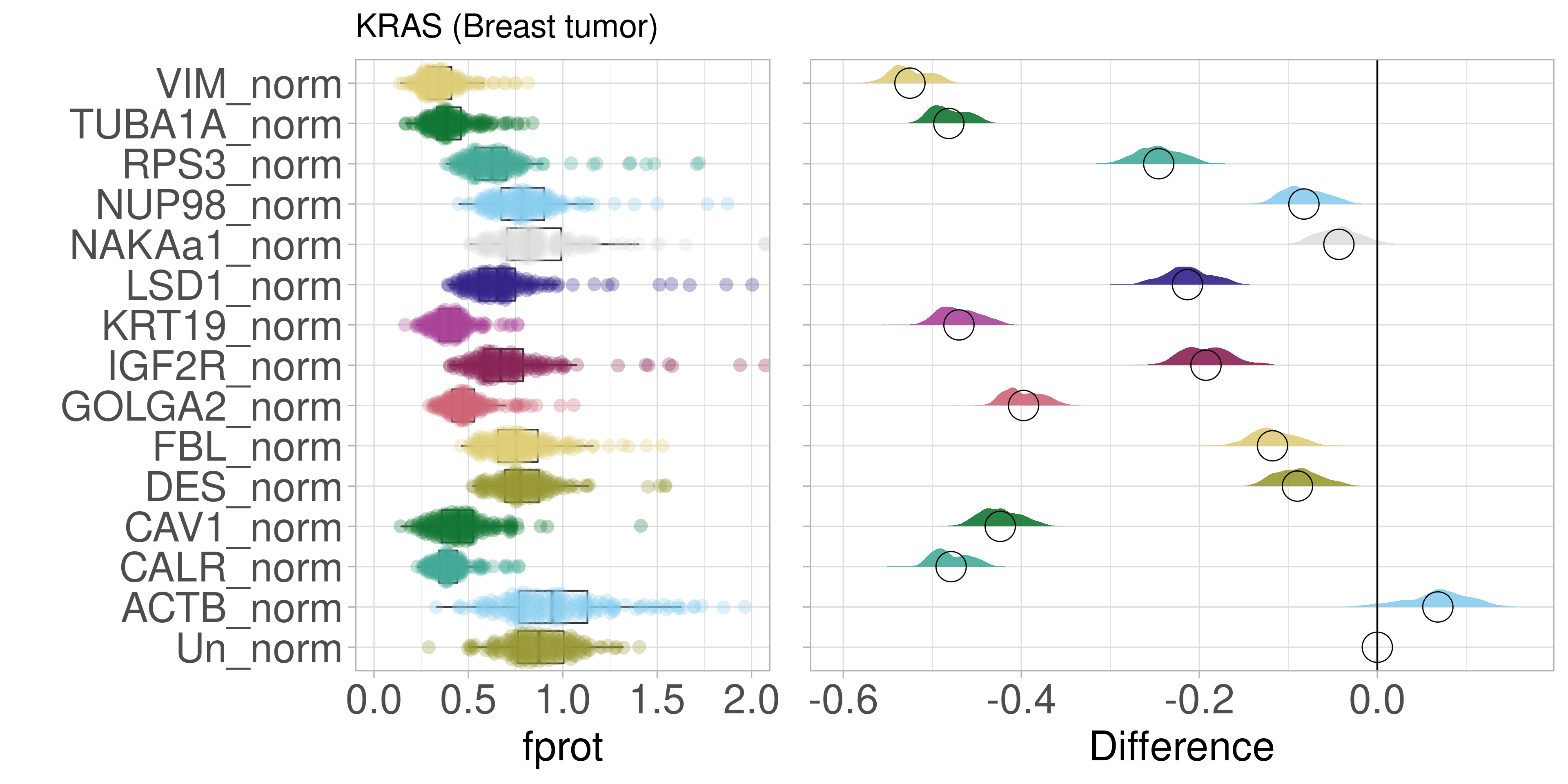

Supplement: Supplementary file 17 — Supplementary Material 17 [file 41598_2026_48754_MOESM17_ESM.zip › RPPA normalizations to cell markers/Breast_Plots/Oncoproteins_breast/KRAS_Breast_T.png]

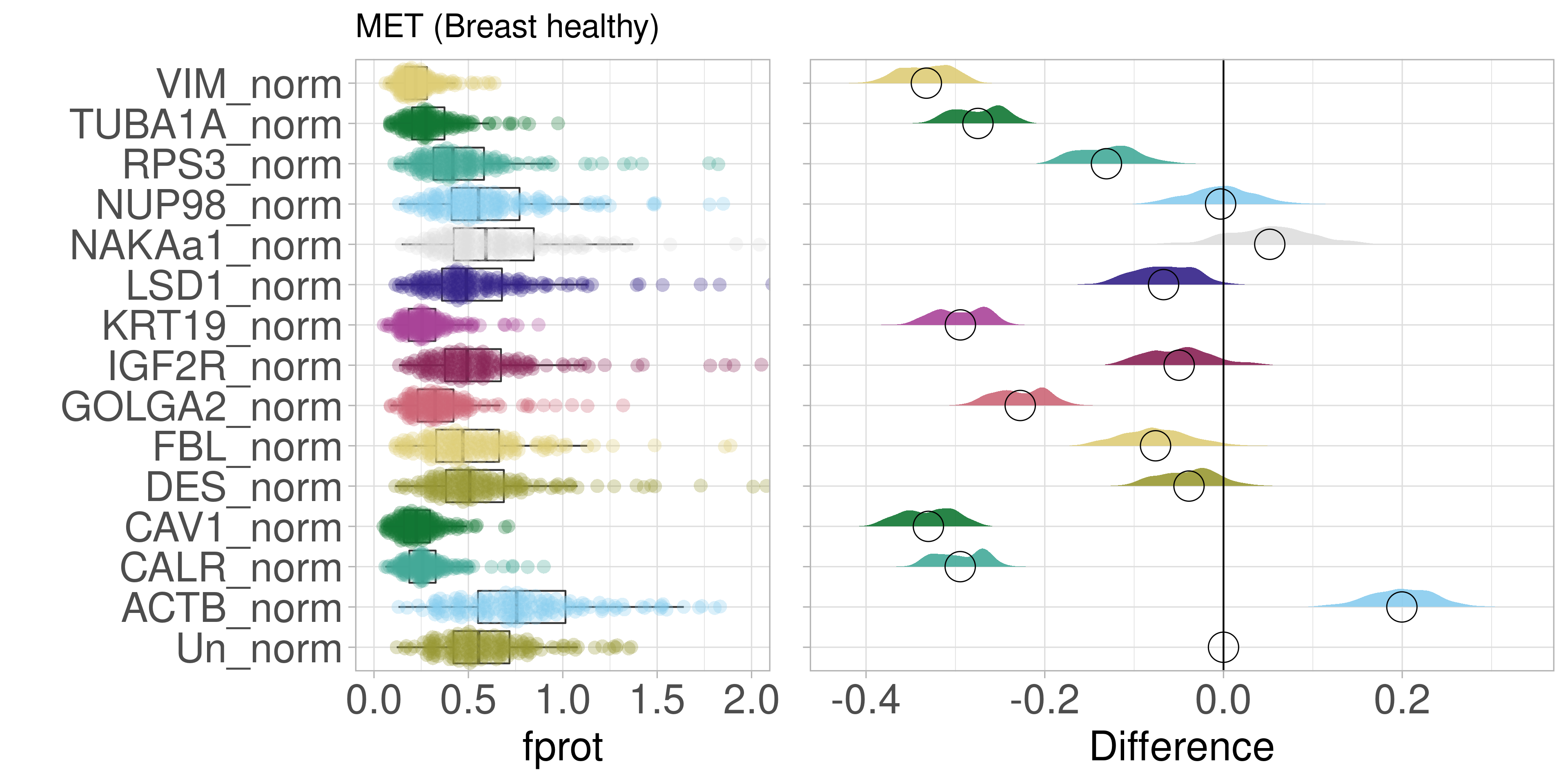

Supplement: Supplementary file 17 — Supplementary Material 17 [file 41598_2026_48754_MOESM17_ESM.zip › RPPA normalizations to cell markers/Breast_Plots/Oncoproteins_breast/MET_Breast_H.png]

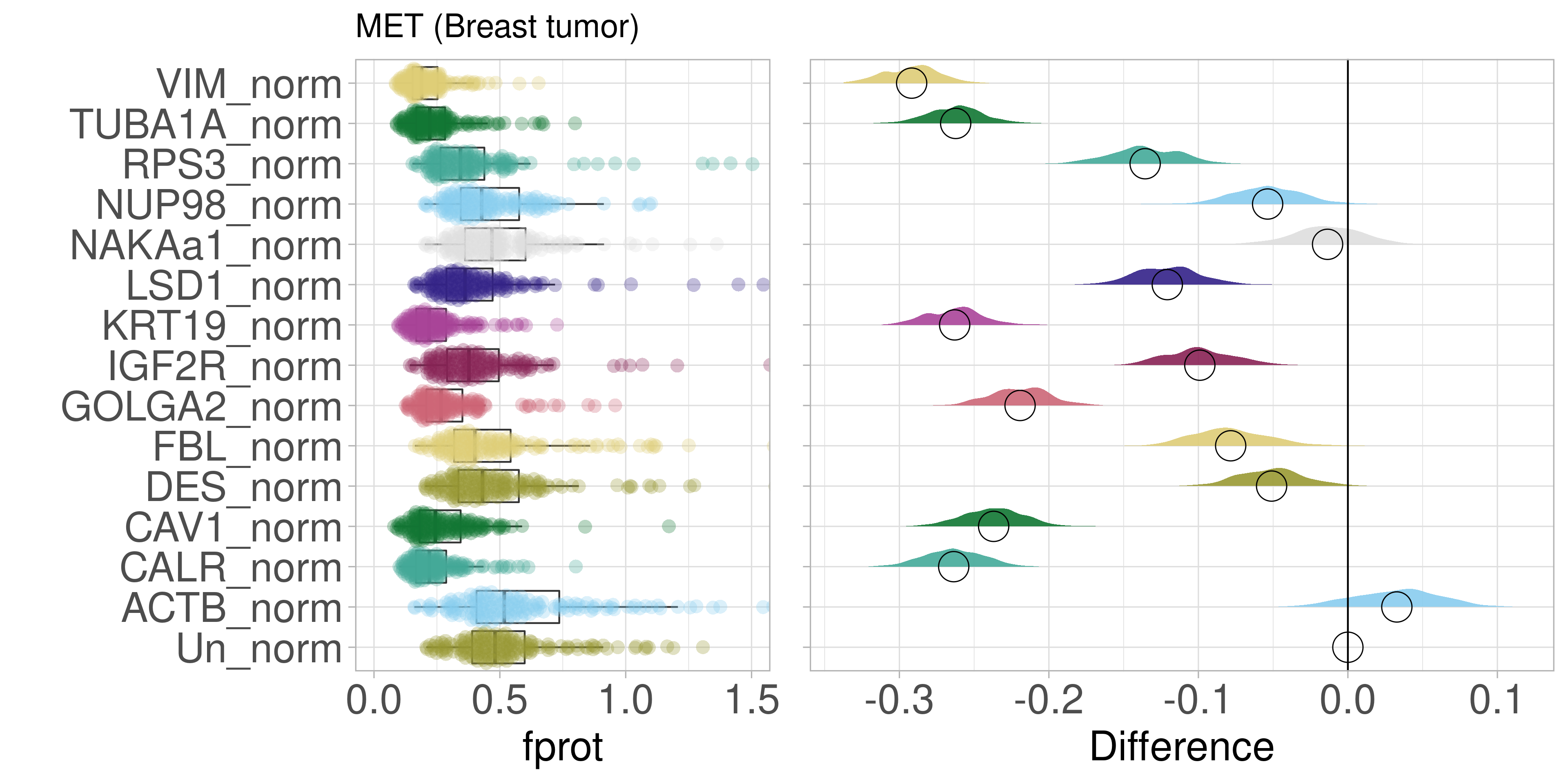

Supplement: Supplementary file 17 — Supplementary Material 17 [file 41598_2026_48754_MOESM17_ESM.zip › RPPA normalizations to cell markers/Breast_Plots/Oncoproteins_breast/MET_Breast_T.png]

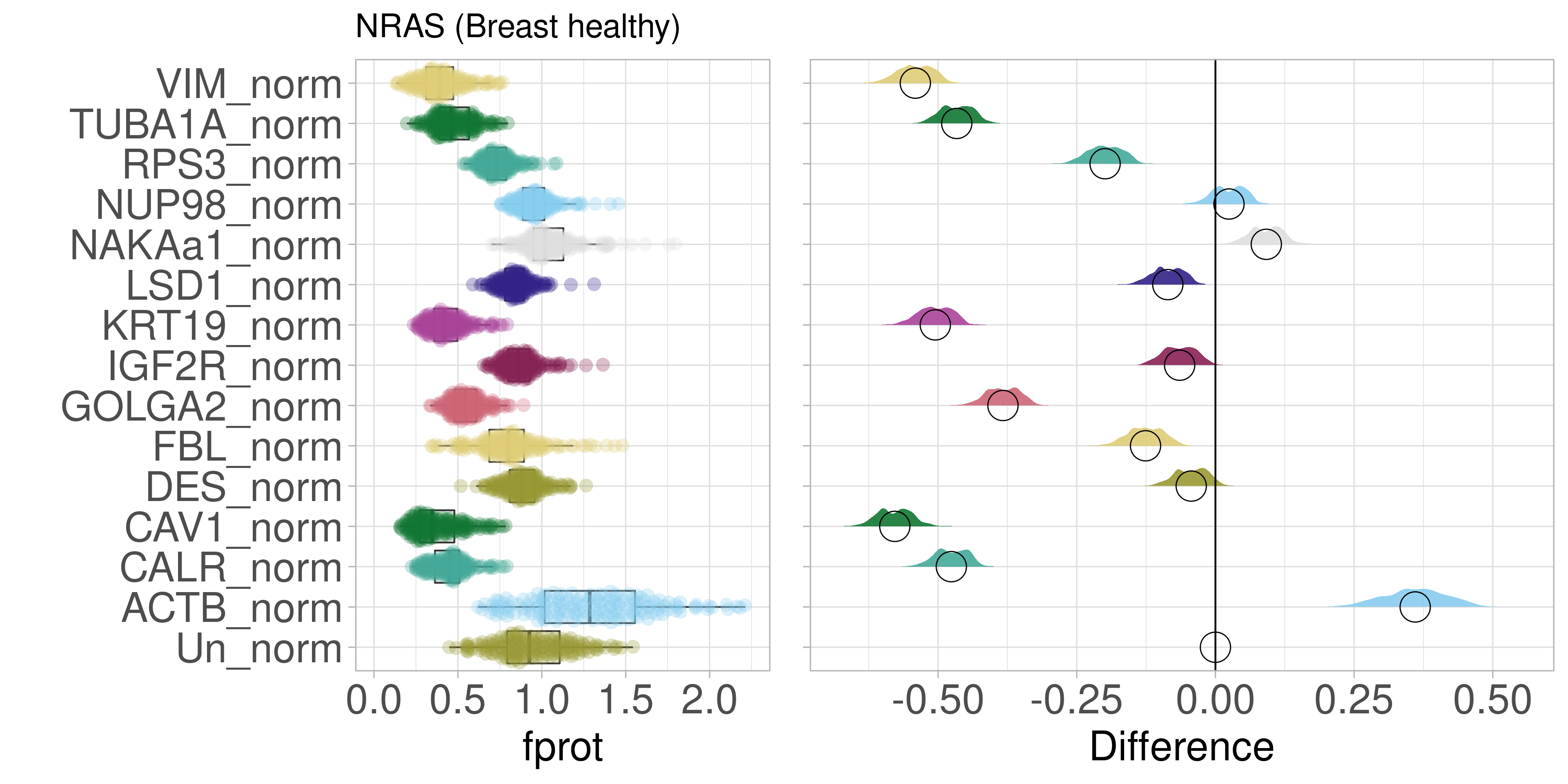

Supplement: Supplementary file 17 — Supplementary Material 17 [file 41598_2026_48754_MOESM17_ESM.zip › RPPA normalizations to cell markers/Breast_Plots/Oncoproteins_breast/NRAS_Breast_H.png]

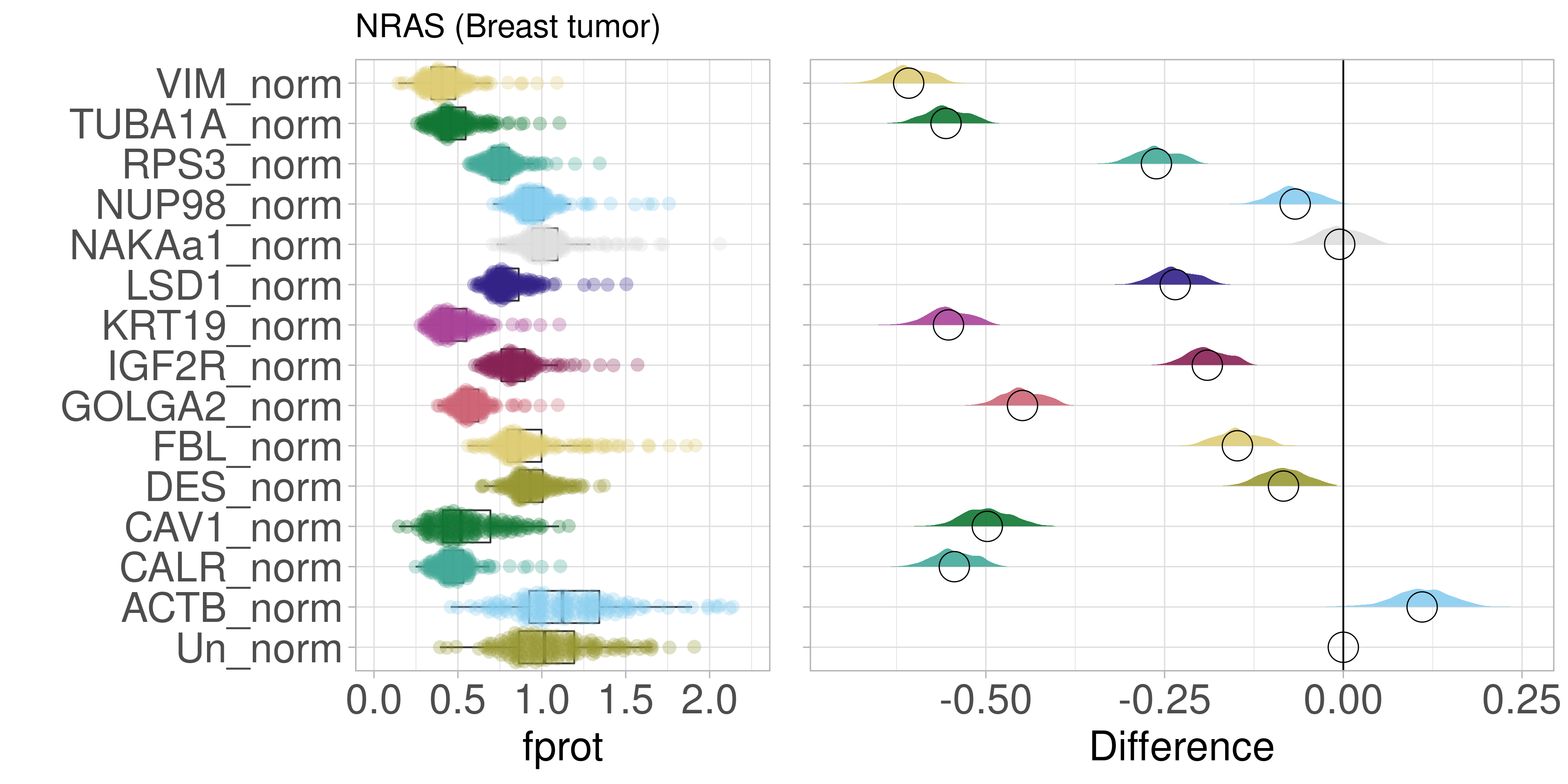

Supplement: Supplementary file 17 — Supplementary Material 17 [file 41598_2026_48754_MOESM17_ESM.zip › RPPA normalizations to cell markers/Breast_Plots/Oncoproteins_breast/NRAS_Breast_T.png]

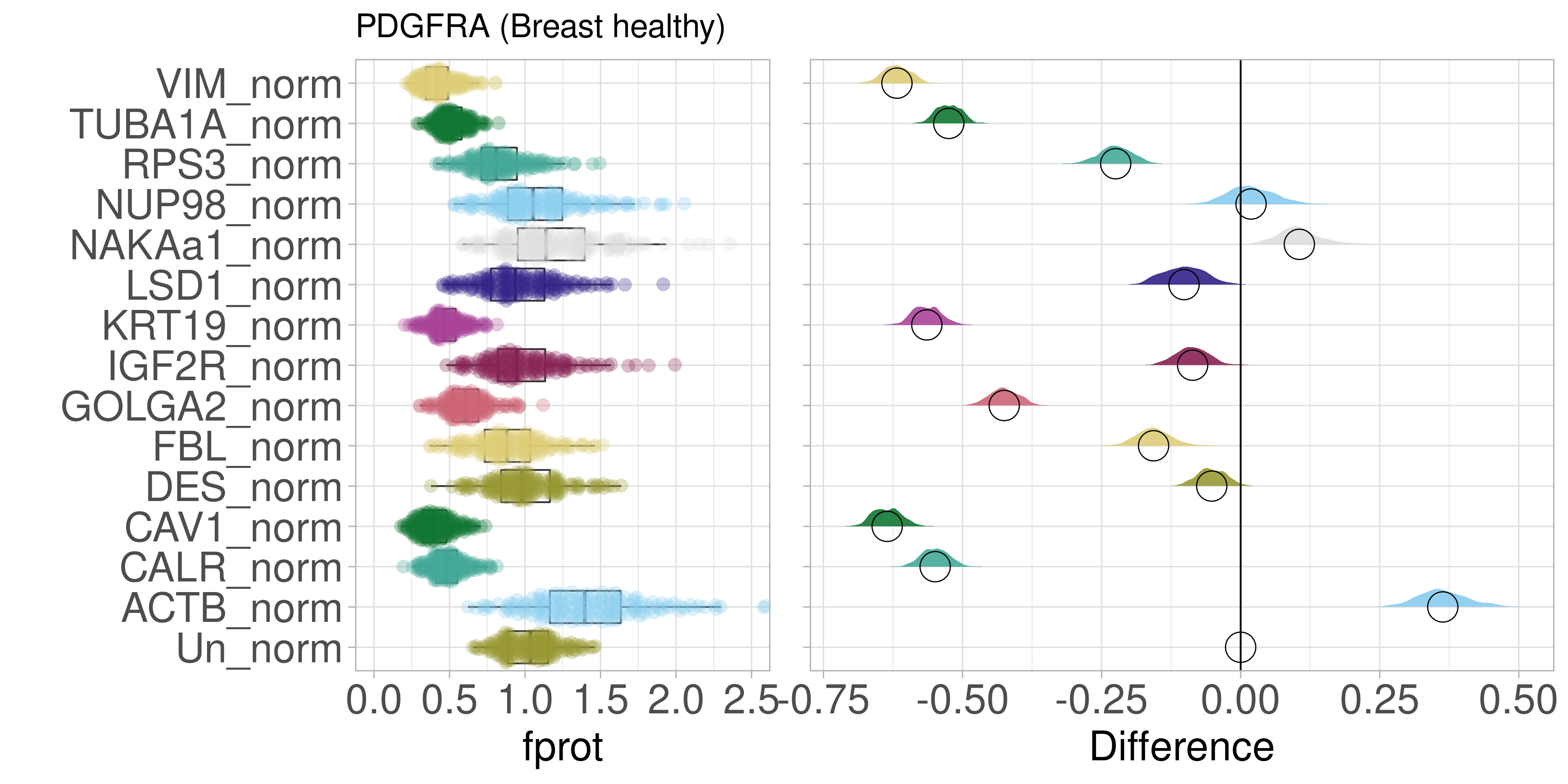

Supplement: Supplementary file 17 — Supplementary Material 17 [file 41598_2026_48754_MOESM17_ESM.zip › RPPA normalizations to cell markers/Breast_Plots/Oncoproteins_breast/PDGFRA_Breast_H.png]

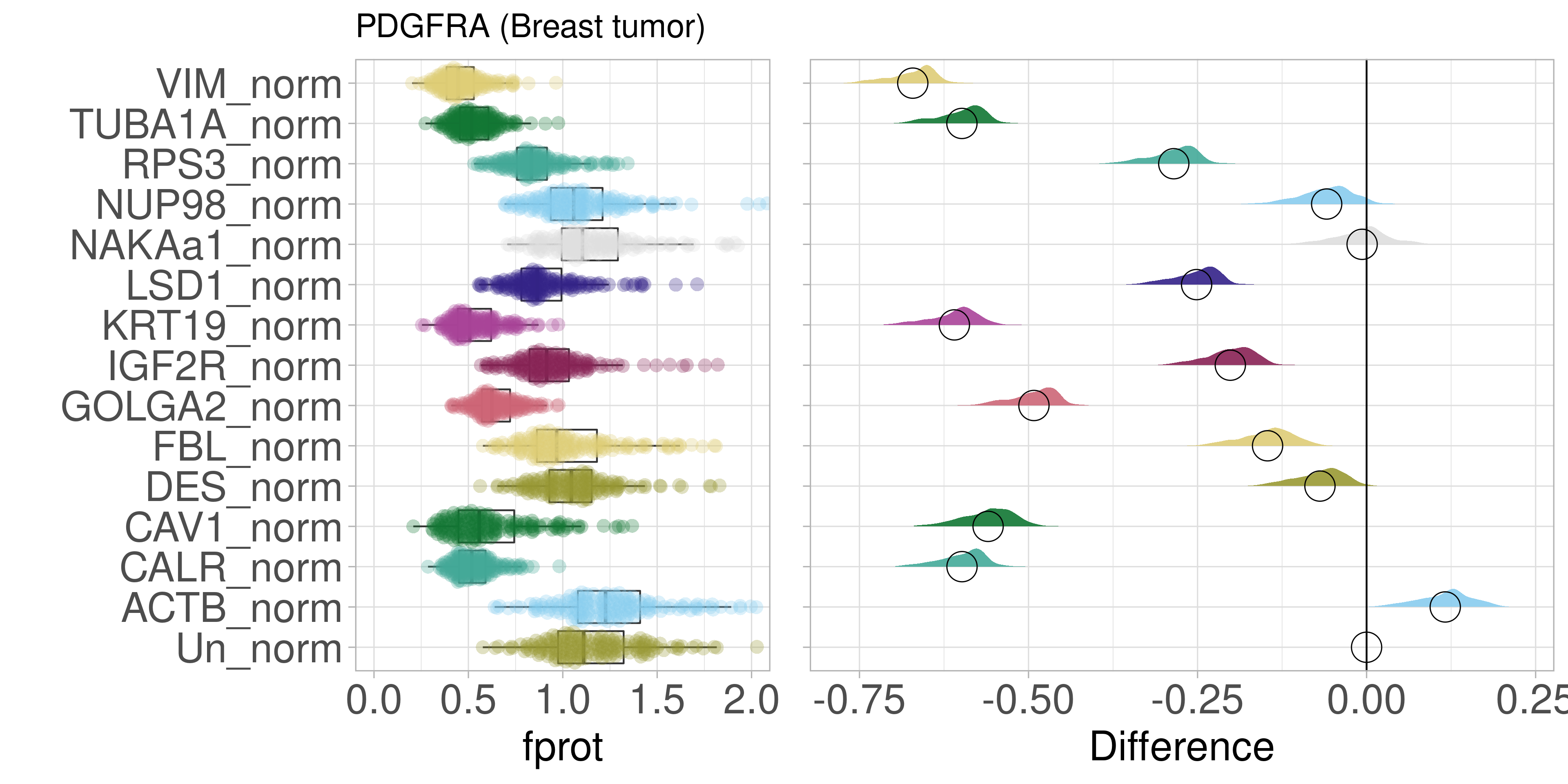

Supplement: Supplementary file 17 — Supplementary Material 17 [file 41598_2026_48754_MOESM17_ESM.zip › RPPA normalizations to cell markers/Breast_Plots/Oncoproteins_breast/PDGFRA_Breast_T.png]

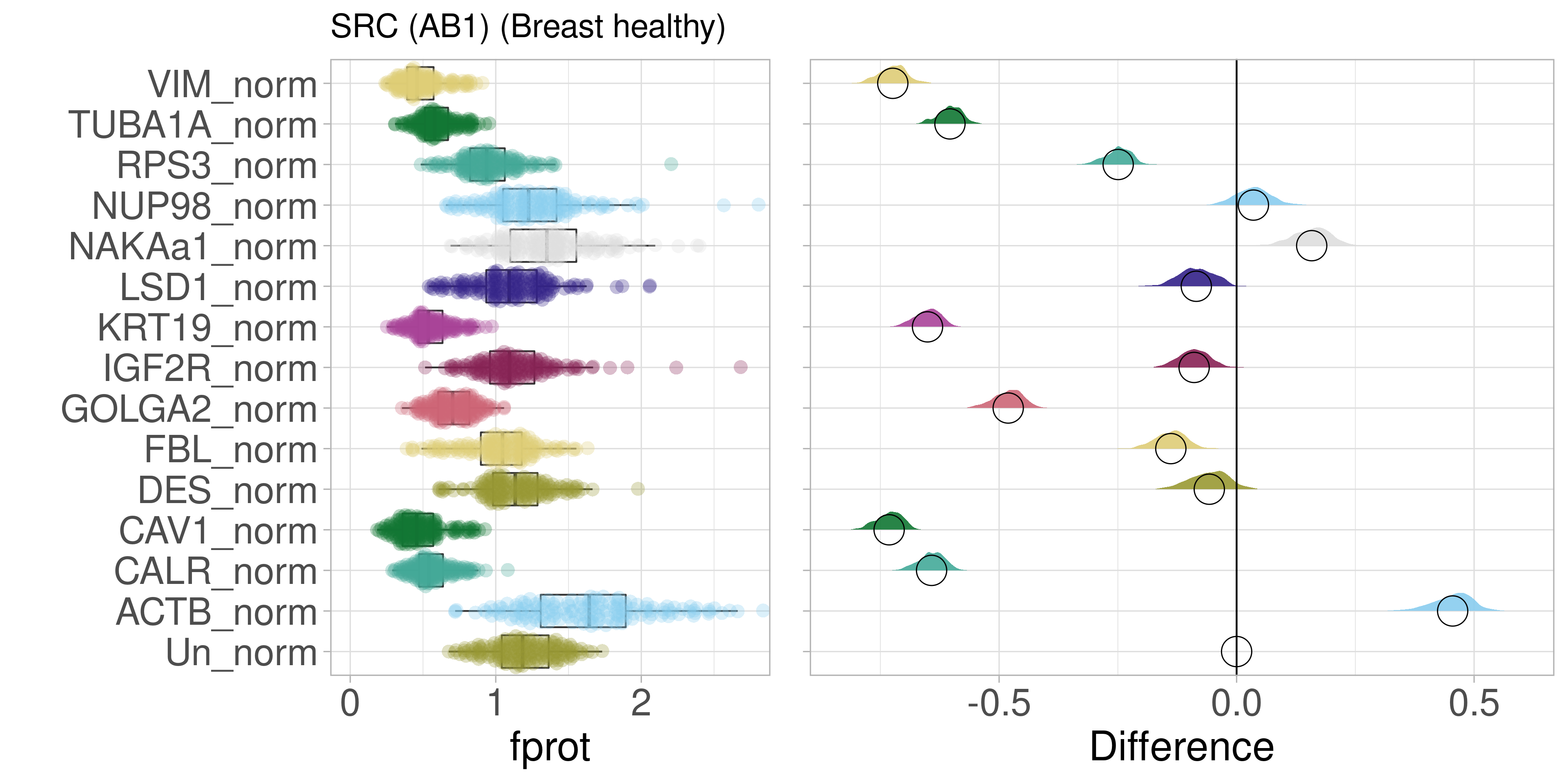

Supplement: Supplementary file 17 — Supplementary Material 17 [file 41598_2026_48754_MOESM17_ESM.zip › RPPA normalizations to cell markers/Breast_Plots/Oncoproteins_breast/SRC(AB1)_Breast_H.png]

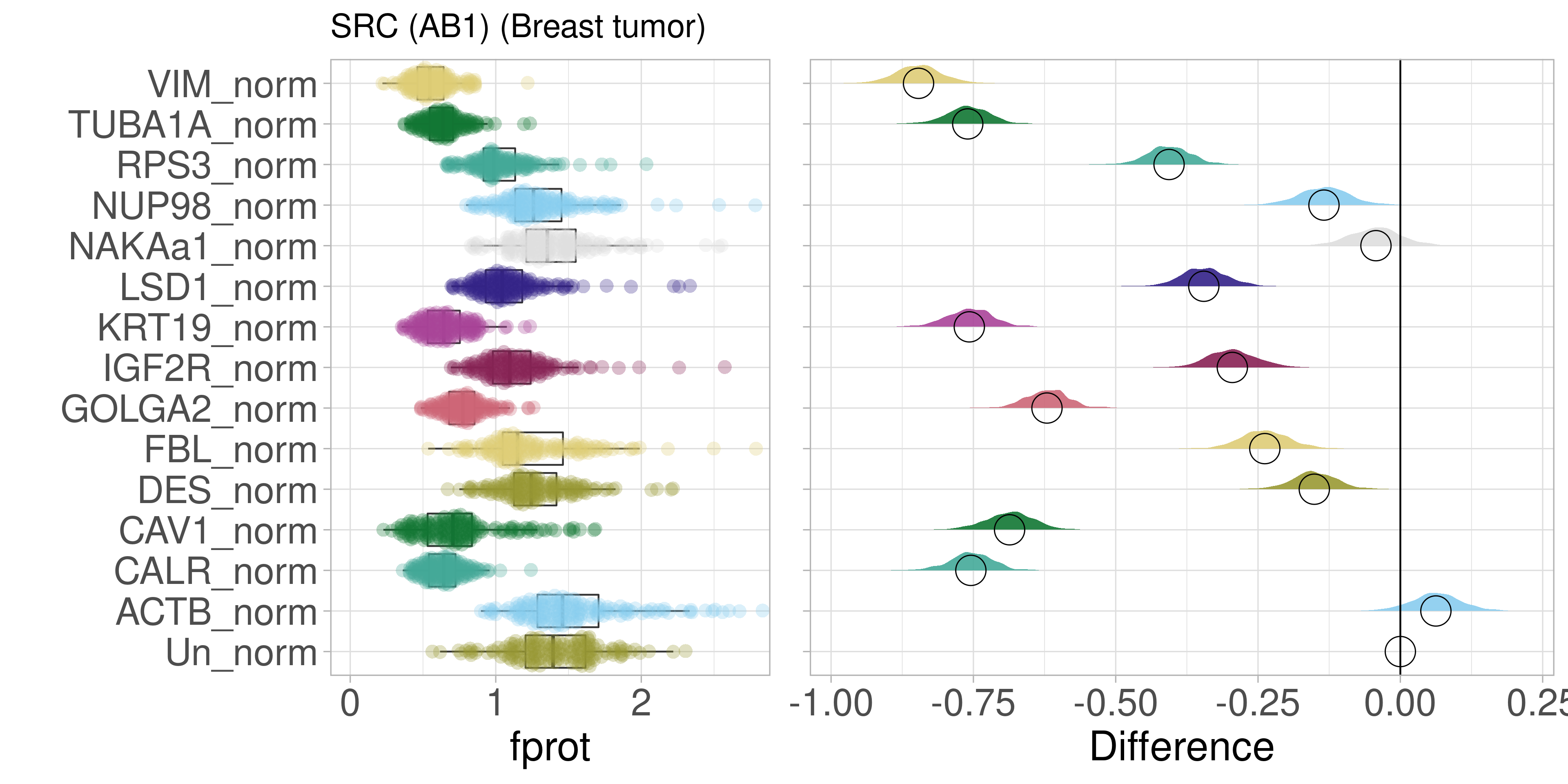

Supplement: Supplementary file 17 — Supplementary Material 17 [file 41598_2026_48754_MOESM17_ESM.zip › RPPA normalizations to cell markers/Breast_Plots/Oncoproteins_breast/SRC(AB1)_Breast_T.png]

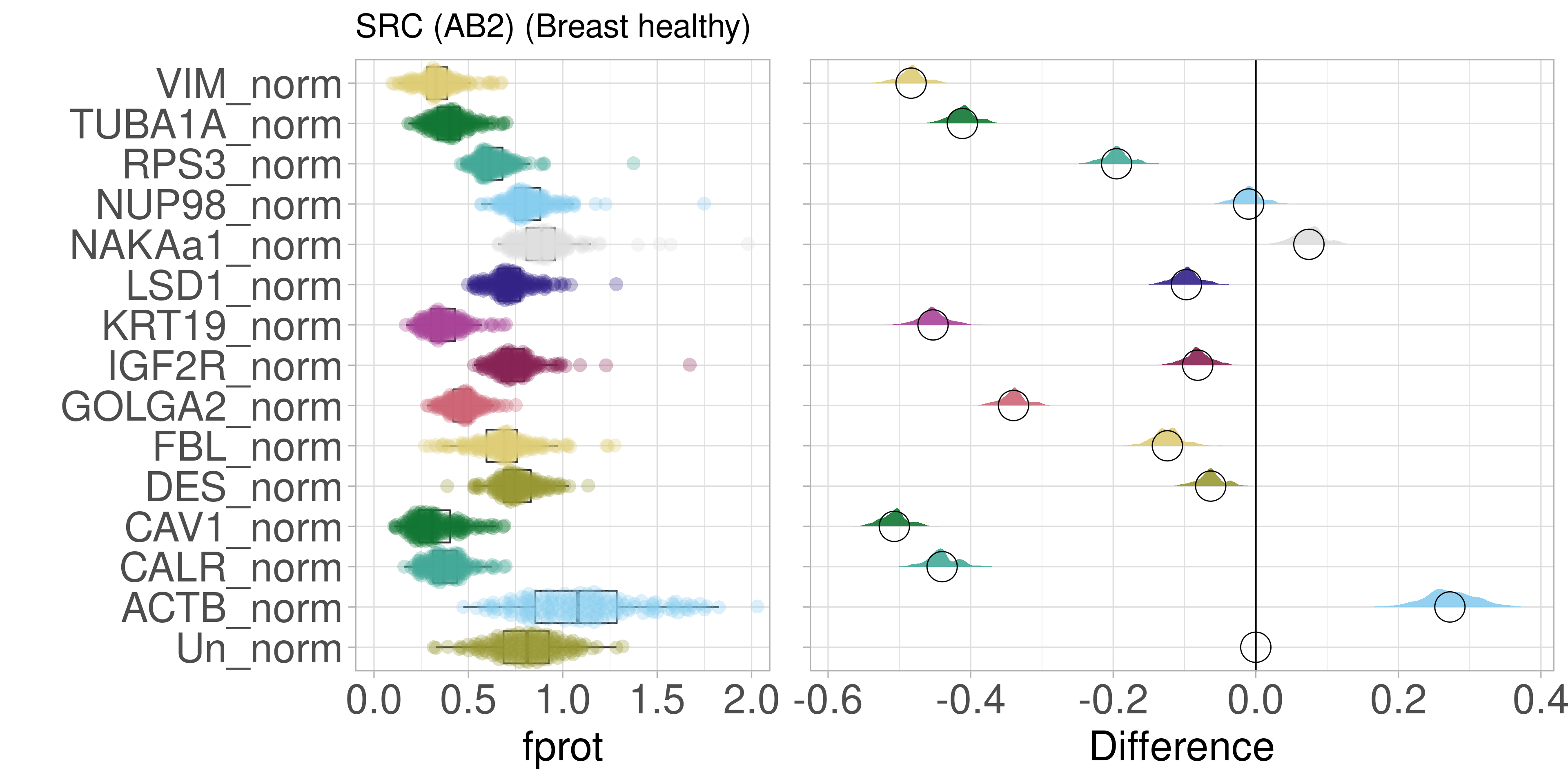

Supplement: Supplementary file 17 — Supplementary Material 17 [file 41598_2026_48754_MOESM17_ESM.zip › RPPA normalizations to cell markers/Breast_Plots/Oncoproteins_breast/SRC(AB2)_Breast_H.png]

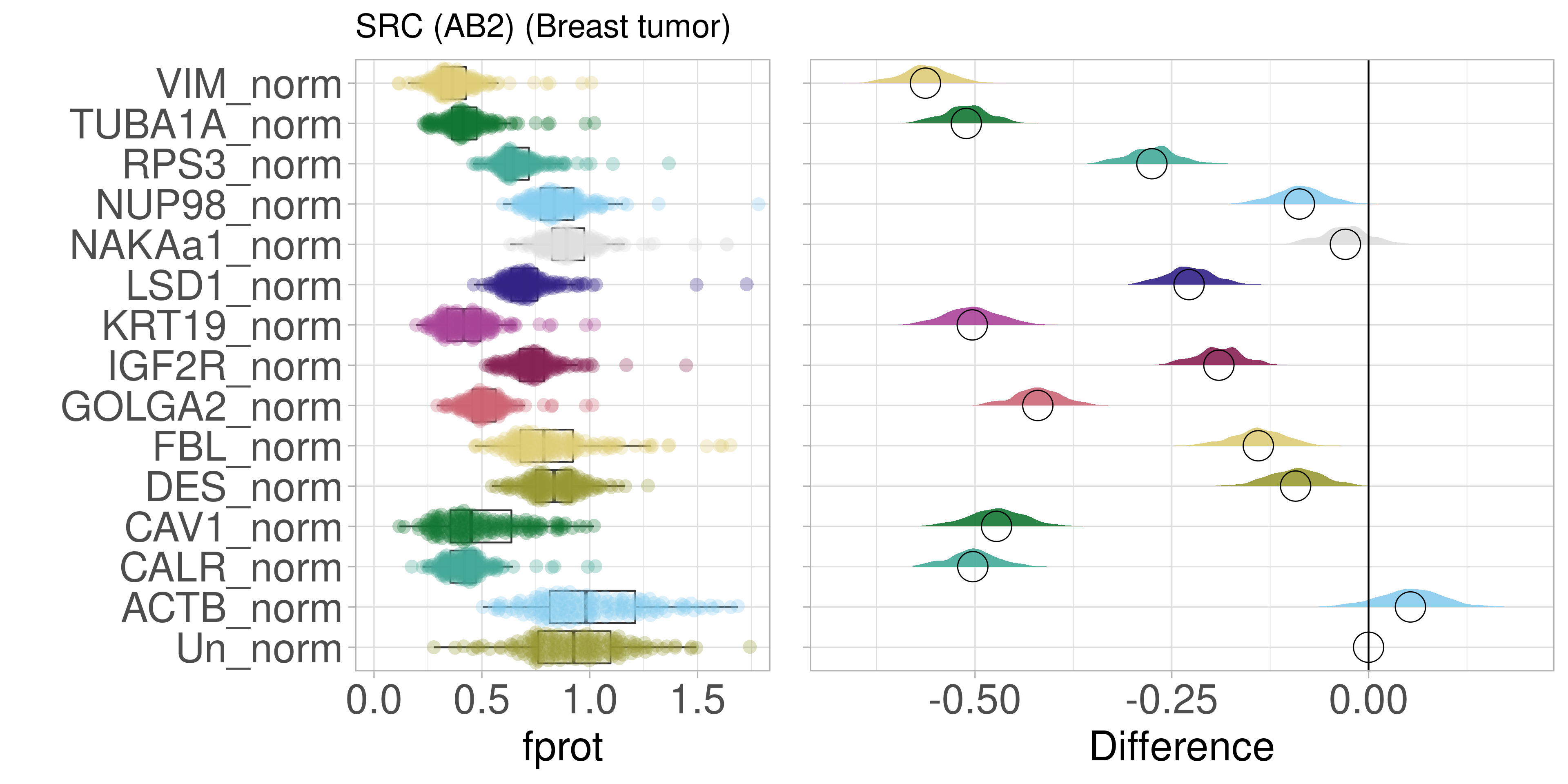

Supplement: Supplementary file 17 — Supplementary Material 17 [file 41598_2026_48754_MOESM17_ESM.zip › RPPA normalizations to cell markers/Breast_Plots/Oncoproteins_breast/SRC(AB2)_Breast_T.png]

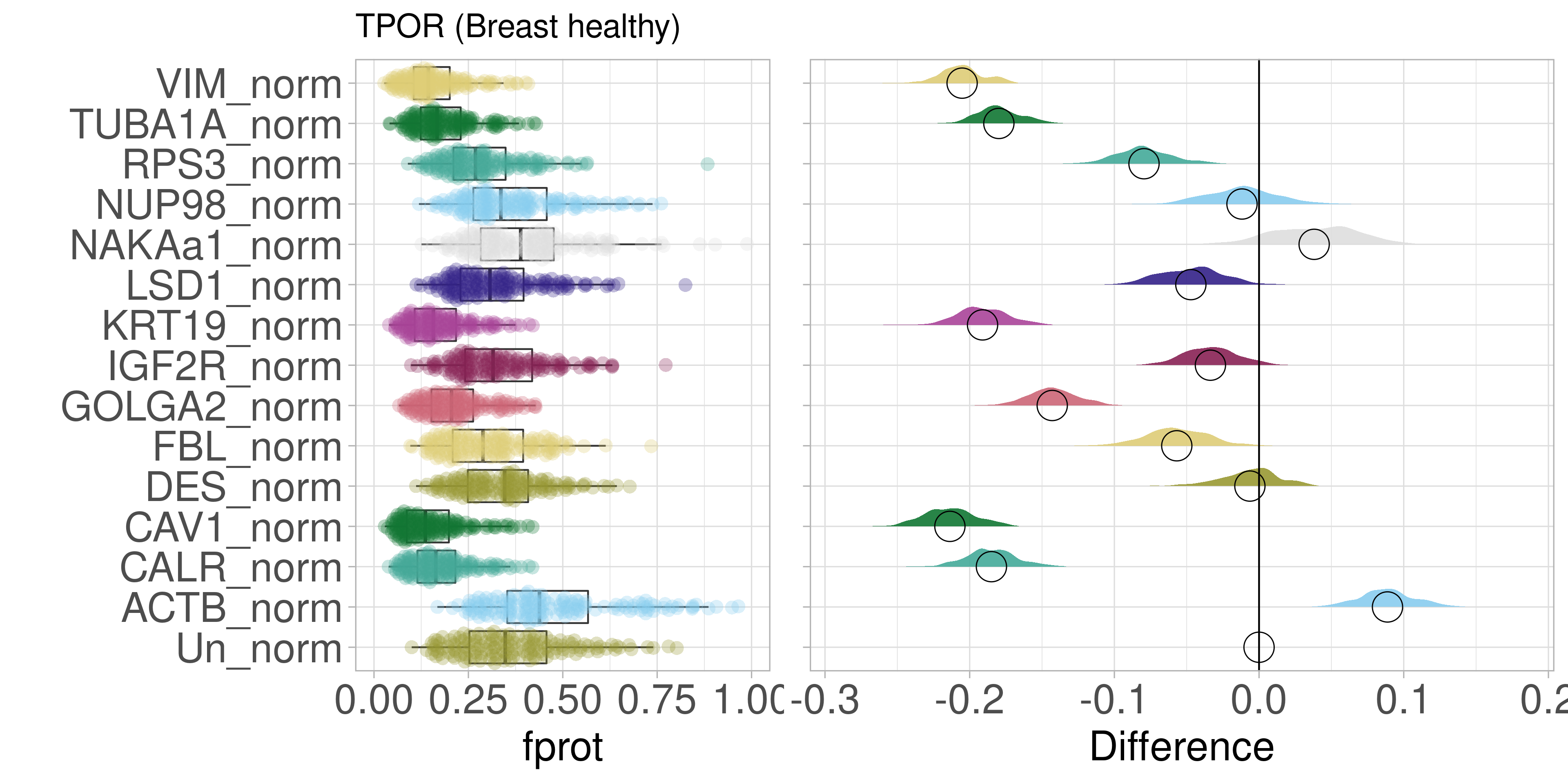

Supplement: Supplementary file 17 — Supplementary Material 17 [file 41598_2026_48754_MOESM17_ESM.zip › RPPA normalizations to cell markers/Breast_Plots/Oncoproteins_breast/TPOR_Breast_H.png]

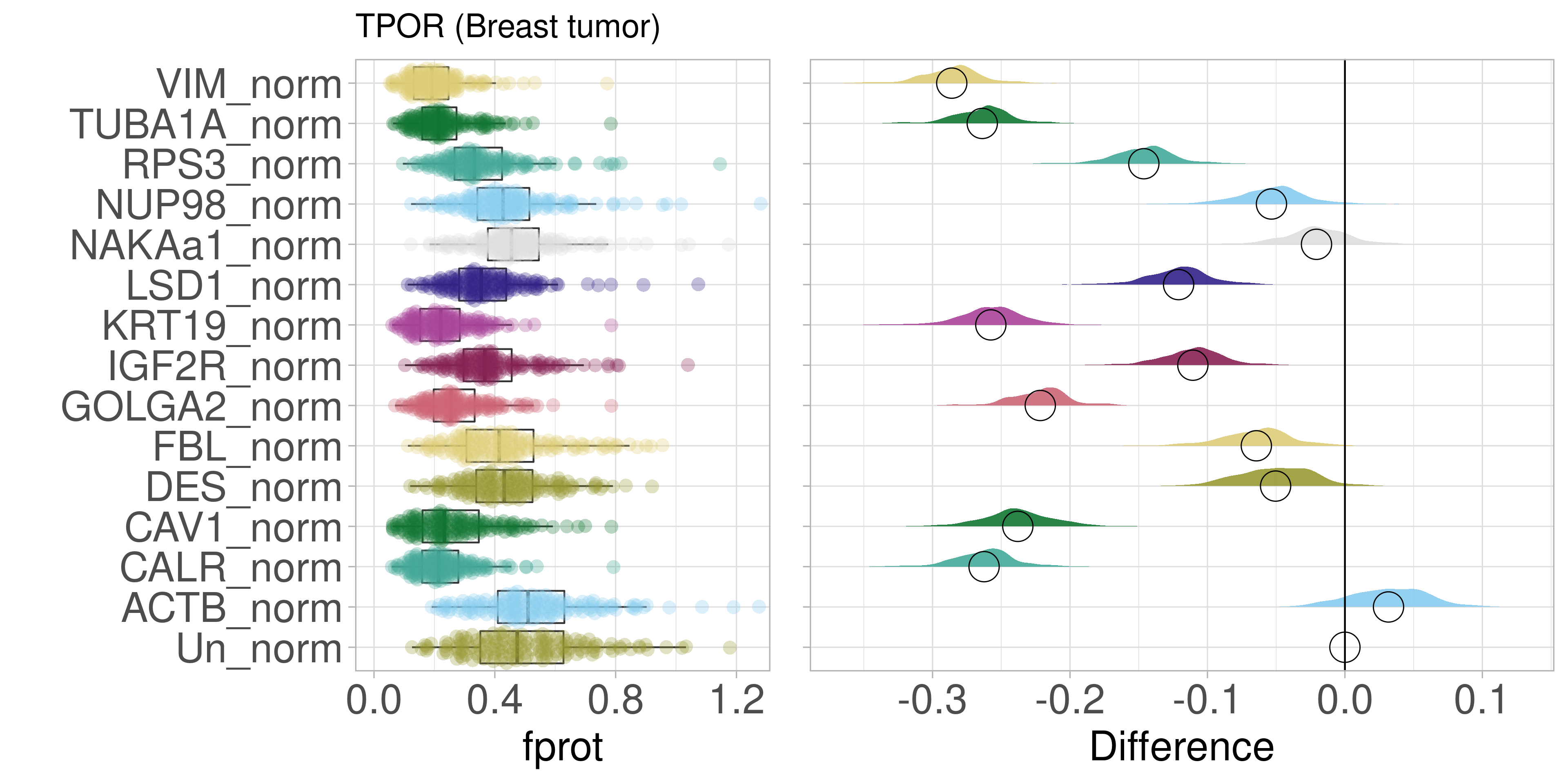

Supplement: Supplementary file 17 — Supplementary Material 17 [file 41598_2026_48754_MOESM17_ESM.zip › RPPA normalizations to cell markers/Breast_Plots/Oncoproteins_breast/TPOR_Breast_T.png]

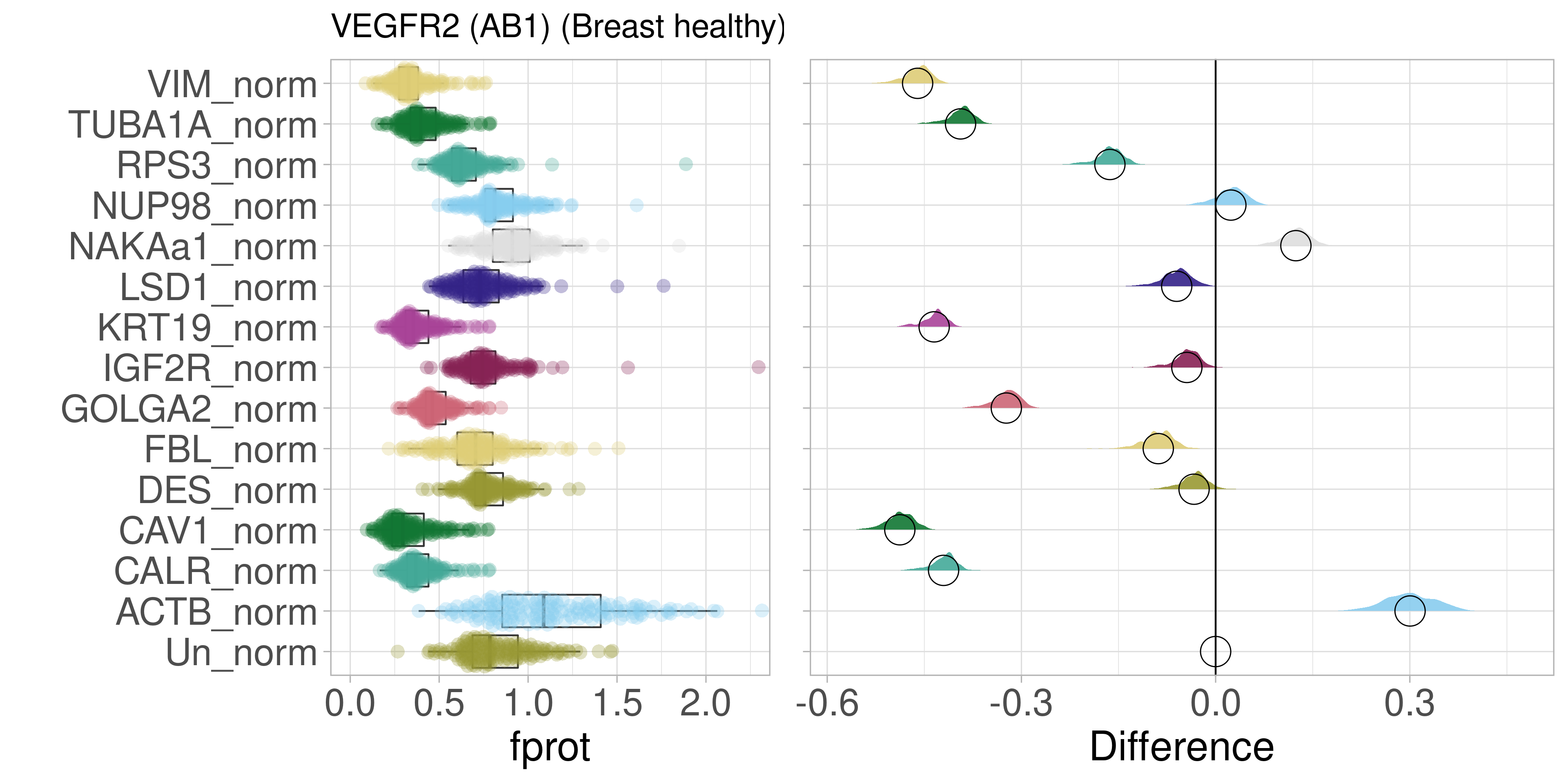

Supplement: Supplementary file 17 — Supplementary Material 17 [file 41598_2026_48754_MOESM17_ESM.zip › RPPA normalizations to cell markers/Breast_Plots/Oncoproteins_breast/VEGFR2(AB1)_Breast_H.png]

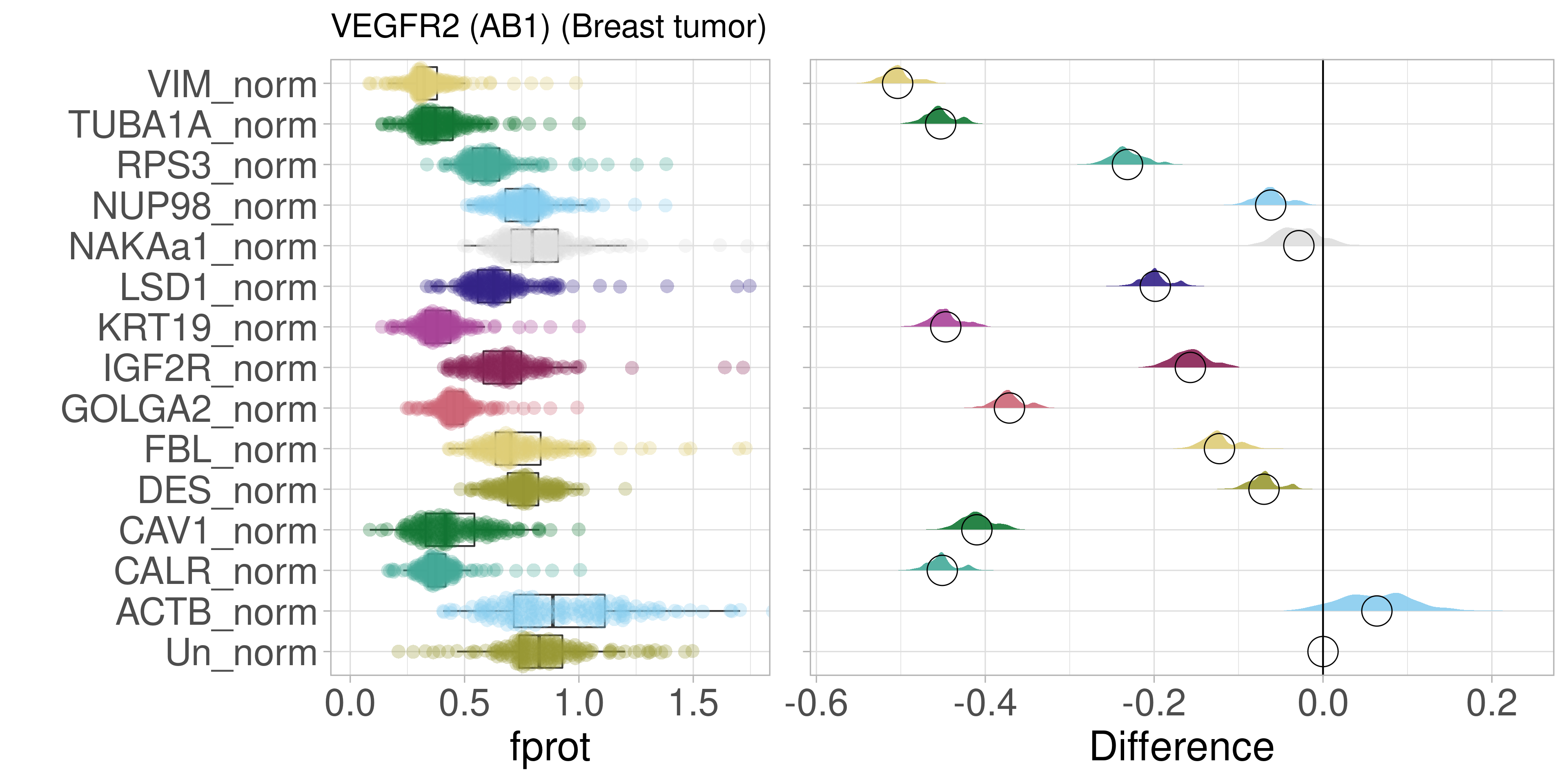

Supplement: Supplementary file 17 — Supplementary Material 17 [file 41598_2026_48754_MOESM17_ESM.zip › RPPA normalizations to cell markers/Breast_Plots/Oncoproteins_breast/VEGFR2(AB1)_Breast_T.png]

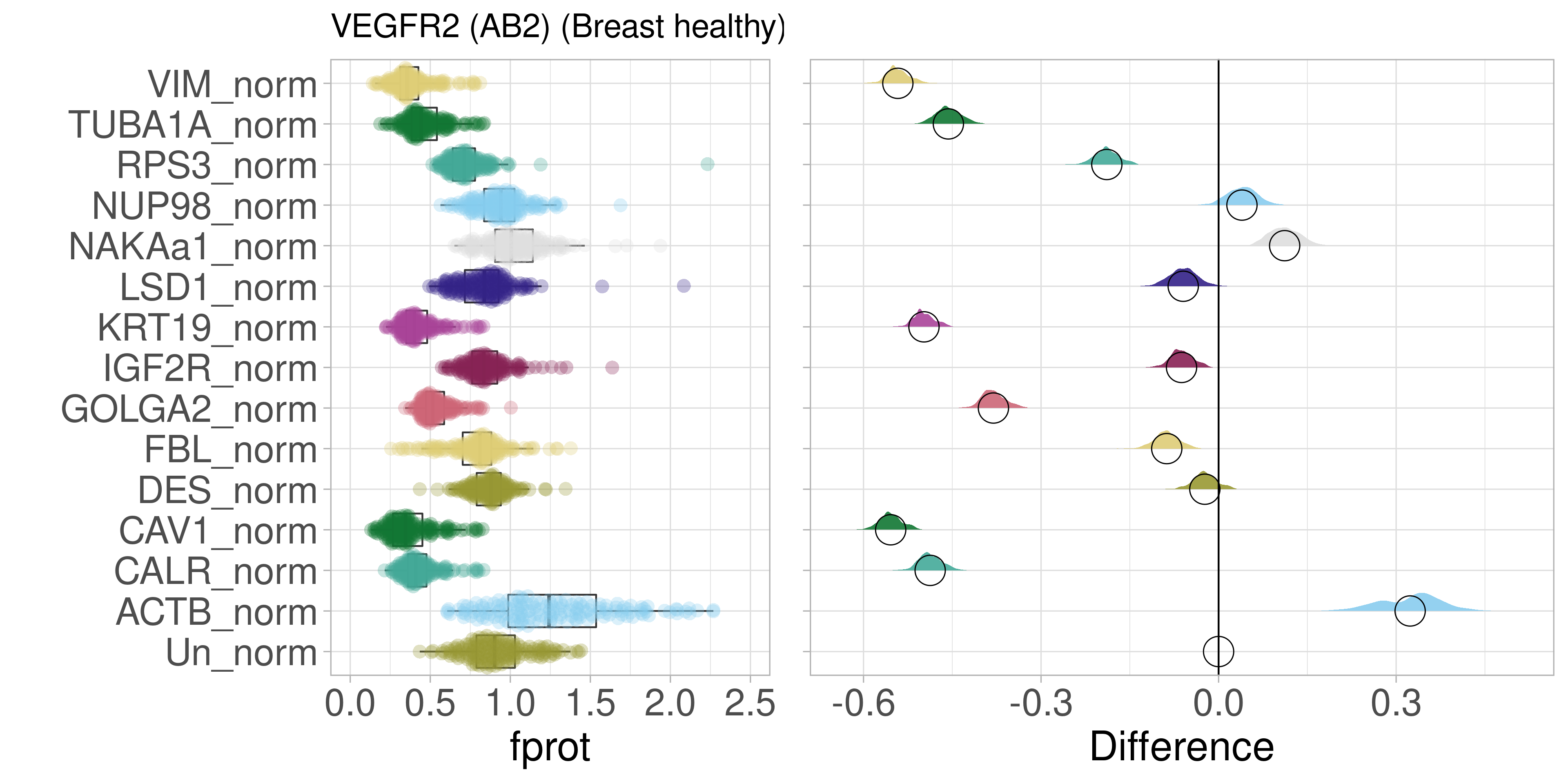

Supplement: Supplementary file 17 — Supplementary Material 17 [file 41598_2026_48754_MOESM17_ESM.zip › RPPA normalizations to cell markers/Breast_Plots/Oncoproteins_breast/VEGFR2(AB2)_Breast_H.png]

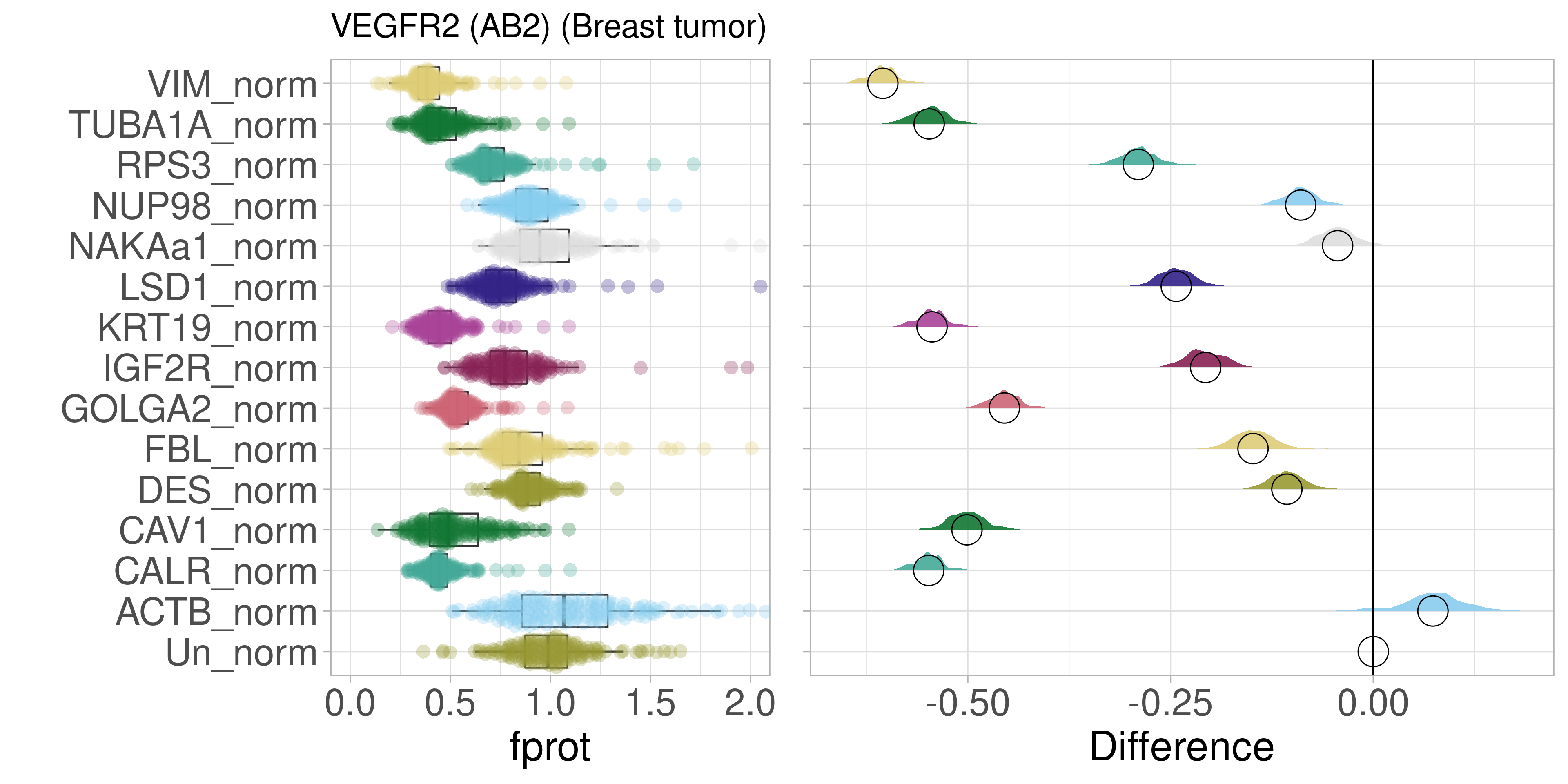

Supplement: Supplementary file 17 — Supplementary Material 17 [file 41598_2026_48754_MOESM17_ESM.zip › RPPA normalizations to cell markers/Breast_Plots/Oncoproteins_breast/VEGFR2(AB2)_Breast_T.png]

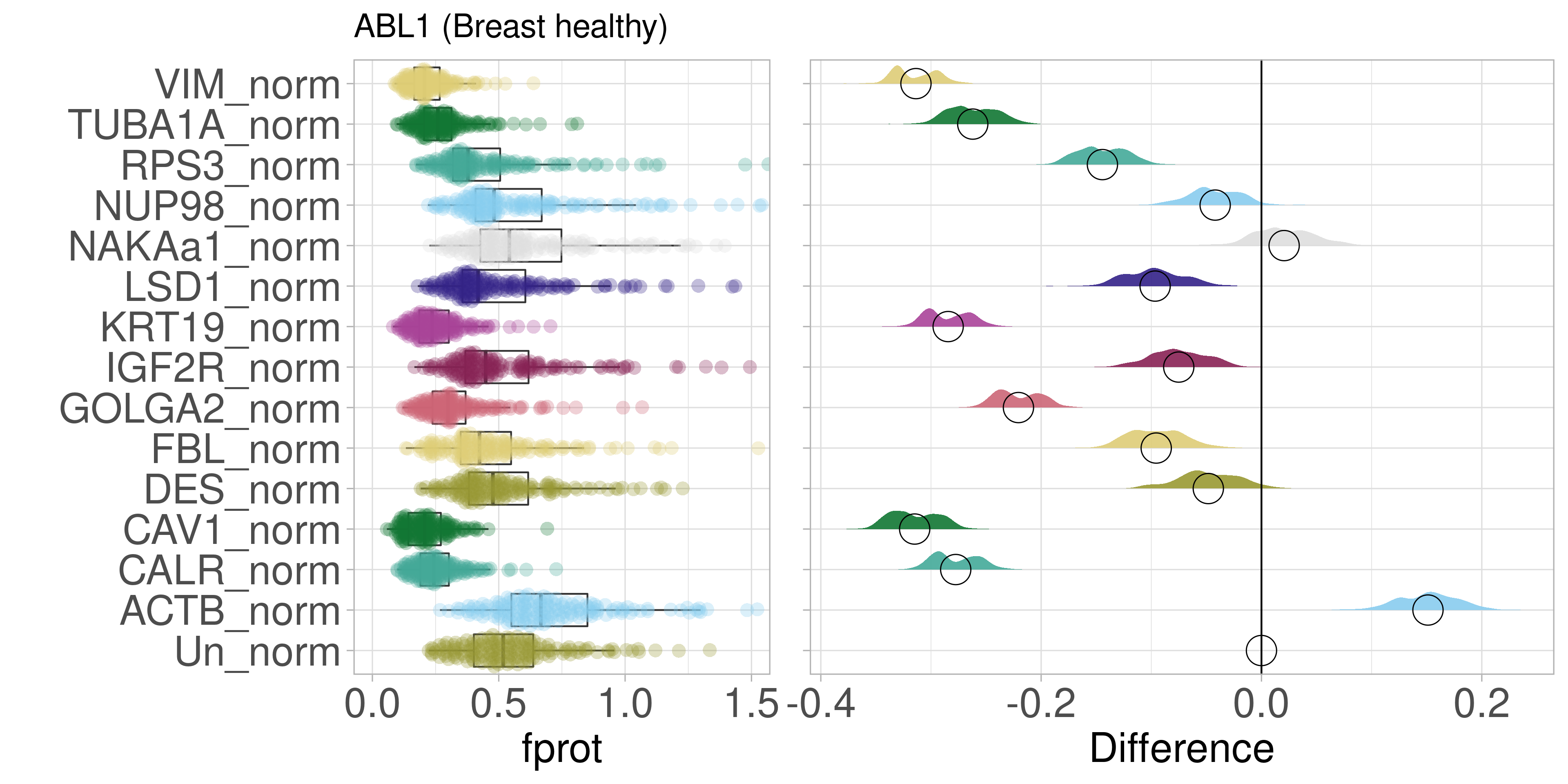

Supplement: Supplementary file 17 — Supplementary Material 17 [file 41598_2026_48754_MOESM17_ESM.zip › RPPA normalizations to cell markers/Breast_Plots/Tumor_suppr_Breast/ABL1_Breast_H.png]

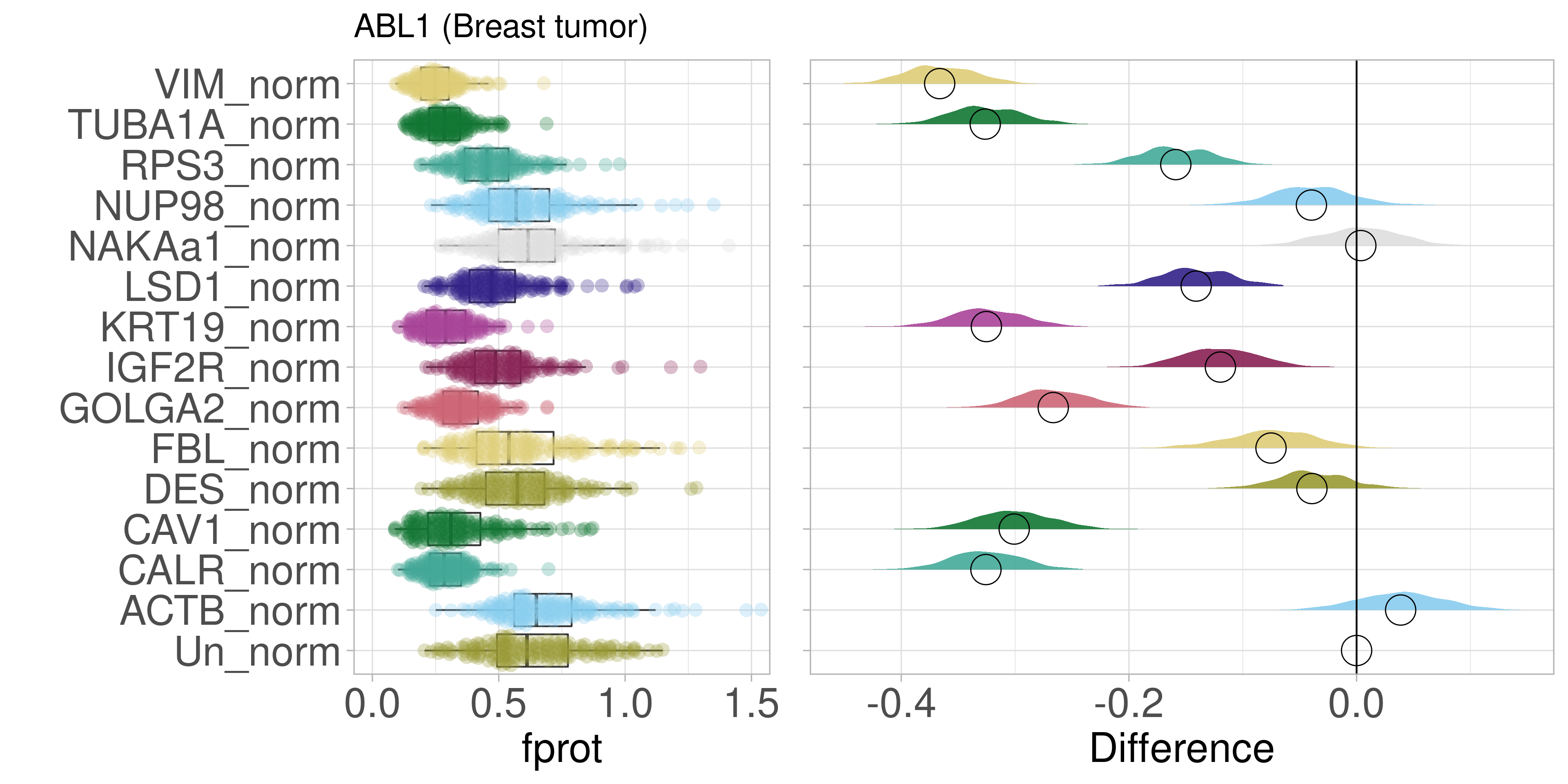

Supplement: Supplementary file 17 — Supplementary Material 17 [file 41598_2026_48754_MOESM17_ESM.zip › RPPA normalizations to cell markers/Breast_Plots/Tumor_suppr_Breast/ABL1_Breast_T.png]

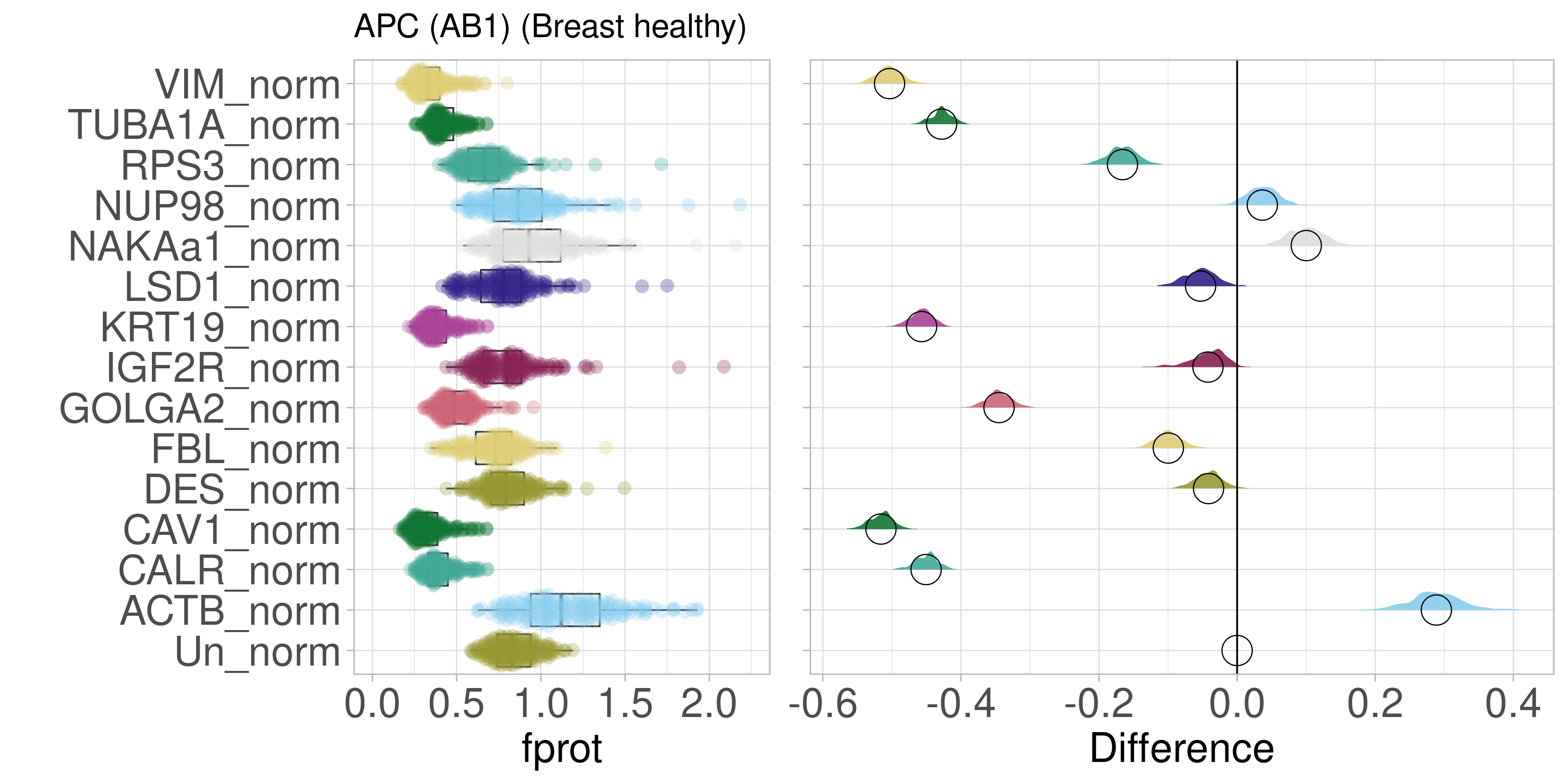

Supplement: Supplementary file 17 — Supplementary Material 17 [file 41598_2026_48754_MOESM17_ESM.zip › RPPA normalizations to cell markers/Breast_Plots/Tumor_suppr_Breast/APC(AB1)_Breast_H.png]

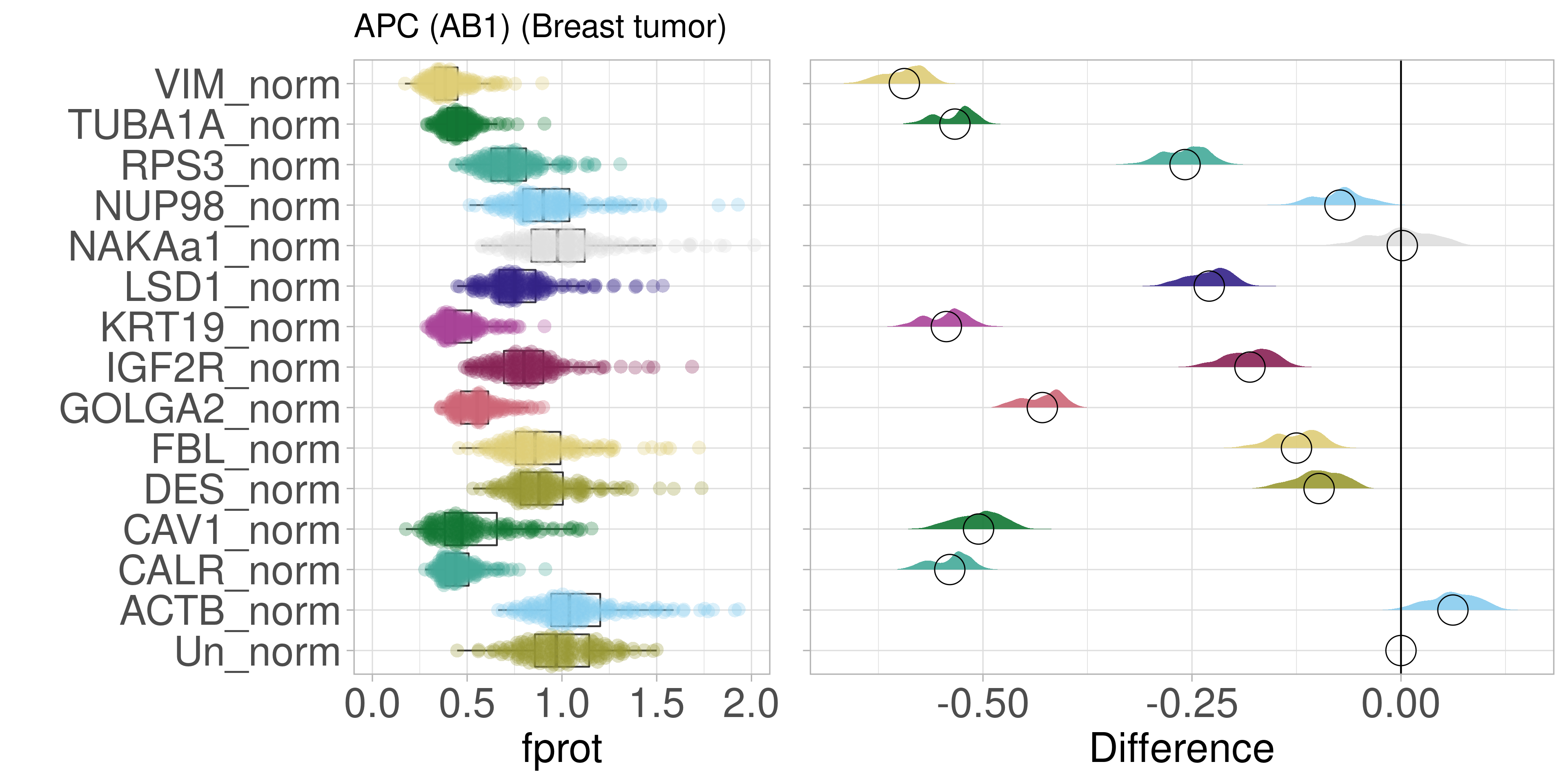

Supplement: Supplementary file 17 — Supplementary Material 17 [file 41598_2026_48754_MOESM17_ESM.zip › RPPA normalizations to cell markers/Breast_Plots/Tumor_suppr_Breast/APC(AB1)_Breast_T.png]

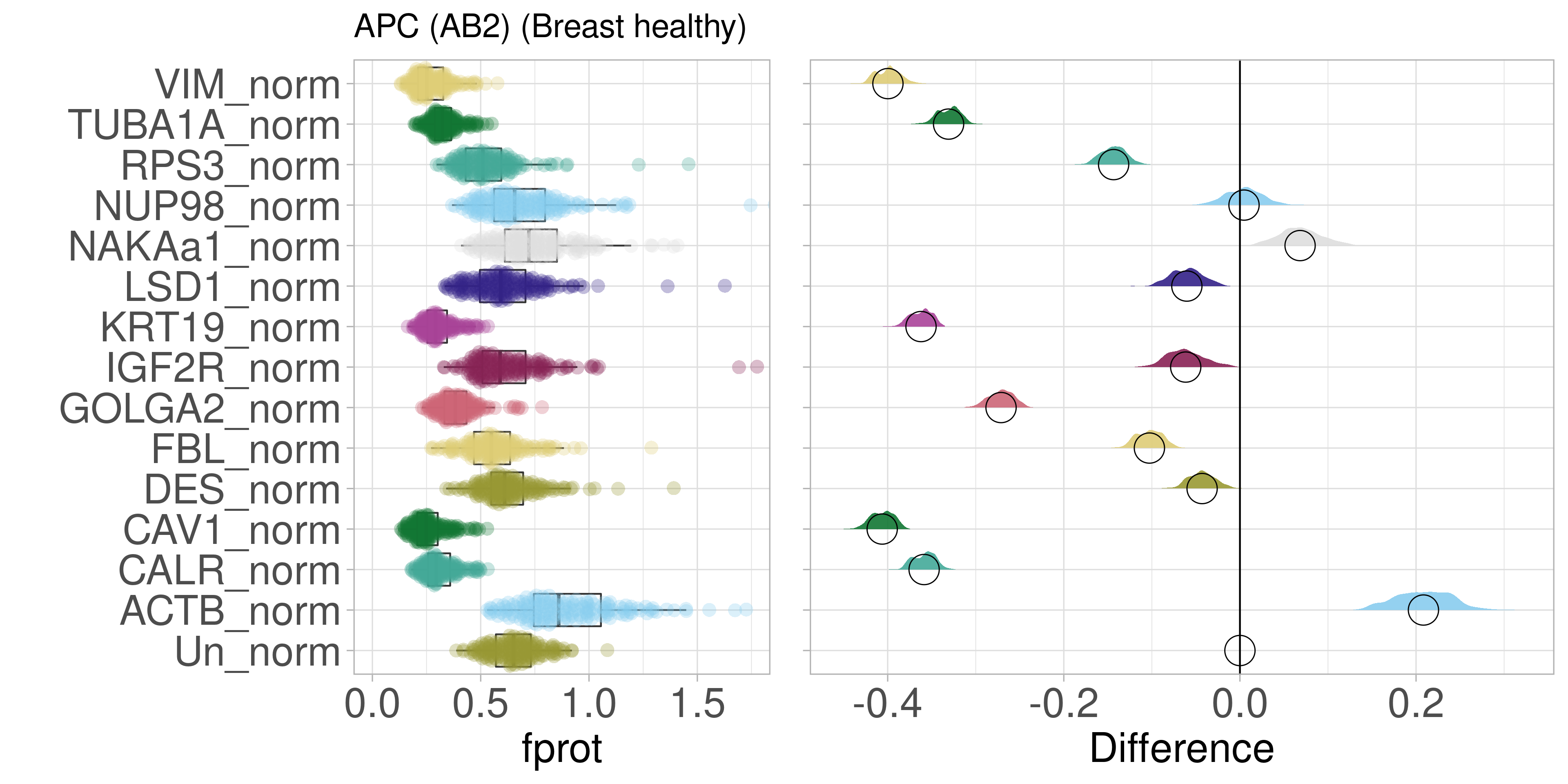

Supplement: Supplementary file 17 — Supplementary Material 17 [file 41598_2026_48754_MOESM17_ESM.zip › RPPA normalizations to cell markers/Breast_Plots/Tumor_suppr_Breast/APC(AB2)_Breast_H.png]

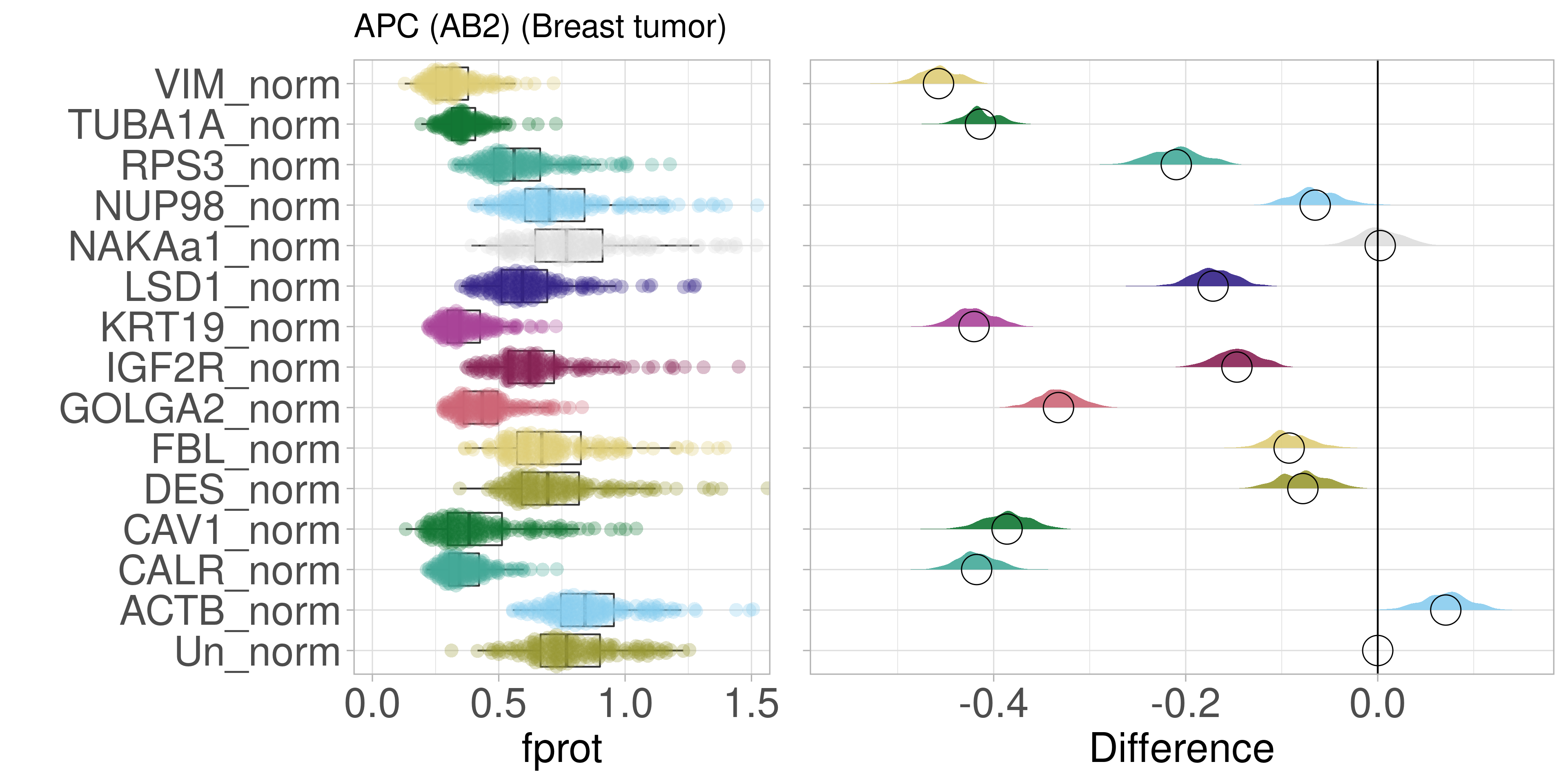

Supplement: Supplementary file 17 — Supplementary Material 17 [file 41598_2026_48754_MOESM17_ESM.zip › RPPA normalizations to cell markers/Breast_Plots/Tumor_suppr_Breast/APC(AB2)_Breast_T.png]

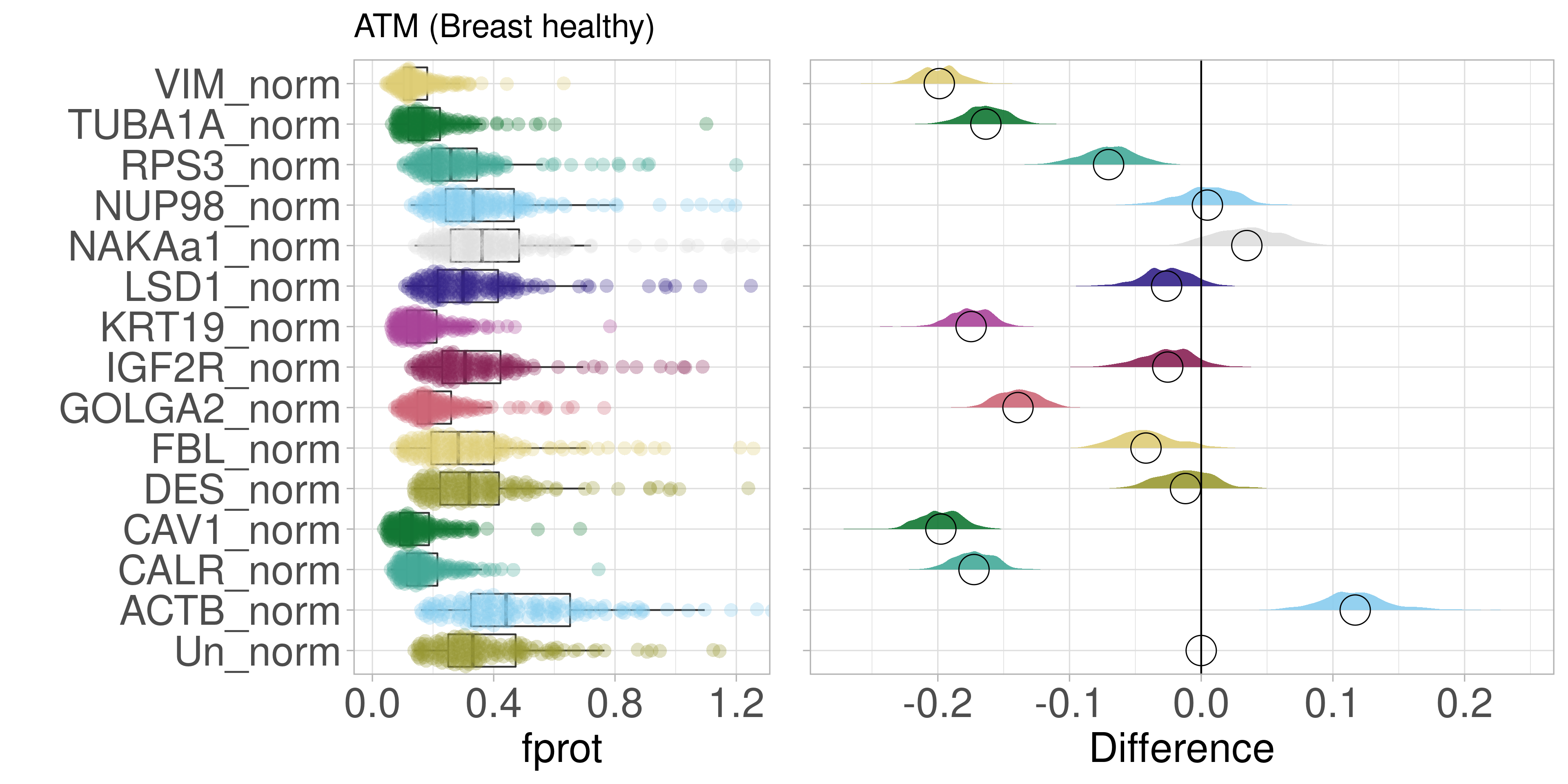

Supplement: Supplementary file 17 — Supplementary Material 17 [file 41598_2026_48754_MOESM17_ESM.zip › RPPA normalizations to cell markers/Breast_Plots/Tumor_suppr_Breast/ATM_Breast_H.png]

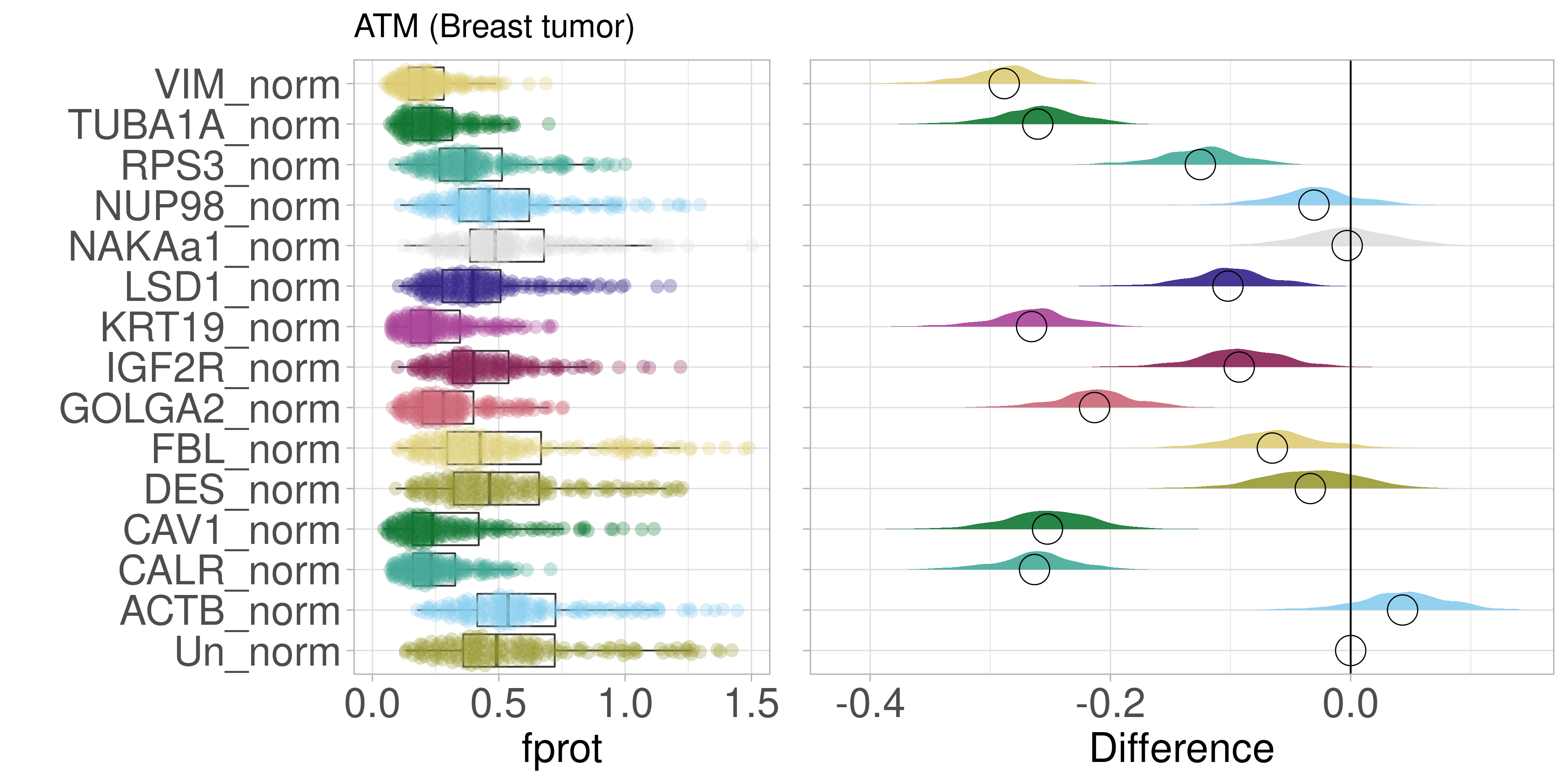

Supplement: Supplementary file 17 — Supplementary Material 17 [file 41598_2026_48754_MOESM17_ESM.zip › RPPA normalizations to cell markers/Breast_Plots/Tumor_suppr_Breast/ATM_Breast_T.png]

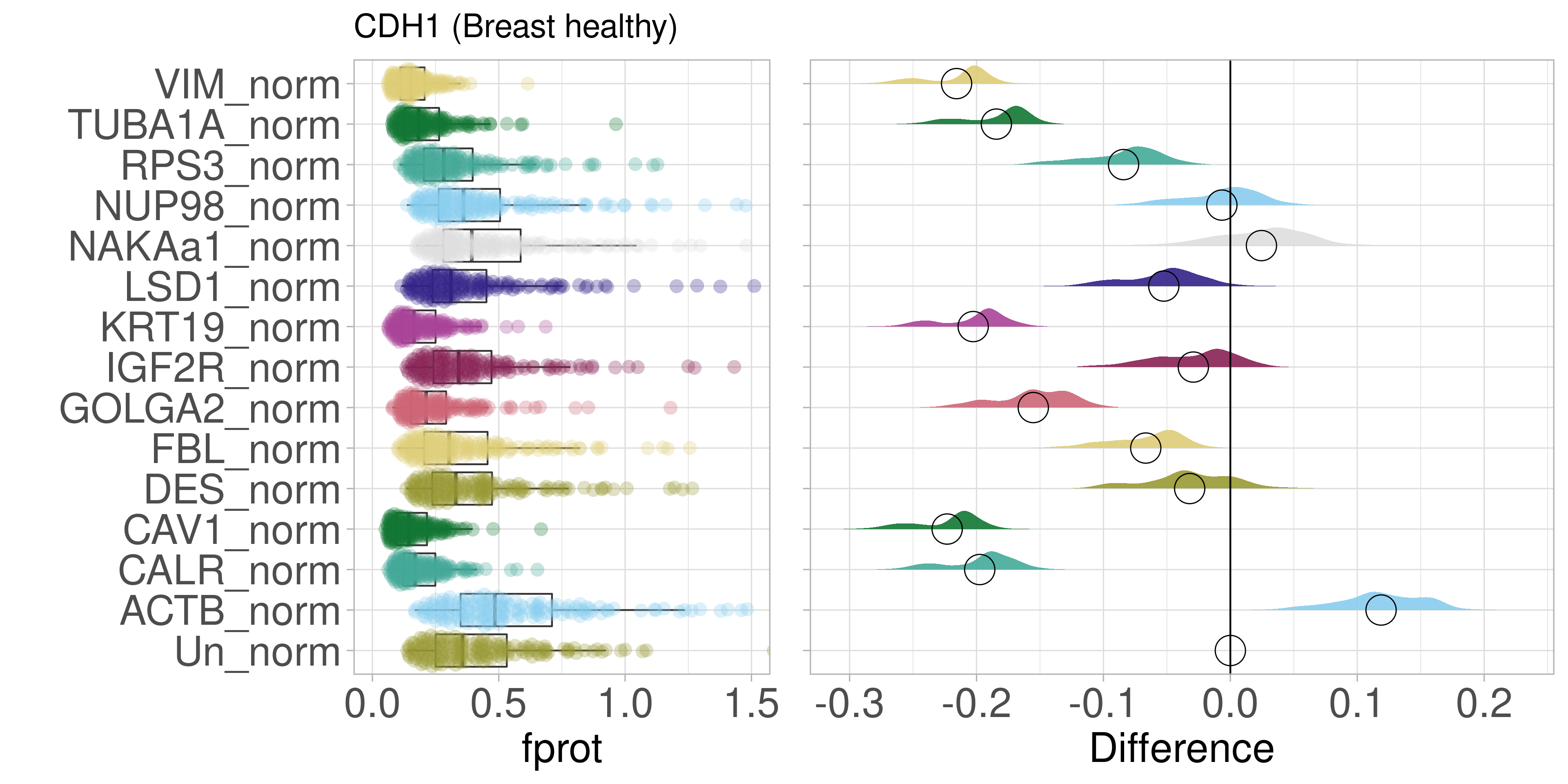

Supplement: Supplementary file 17 — Supplementary Material 17 [file 41598_2026_48754_MOESM17_ESM.zip › RPPA normalizations to cell markers/Breast_Plots/Tumor_suppr_Breast/CDH1_Breast_H.png]

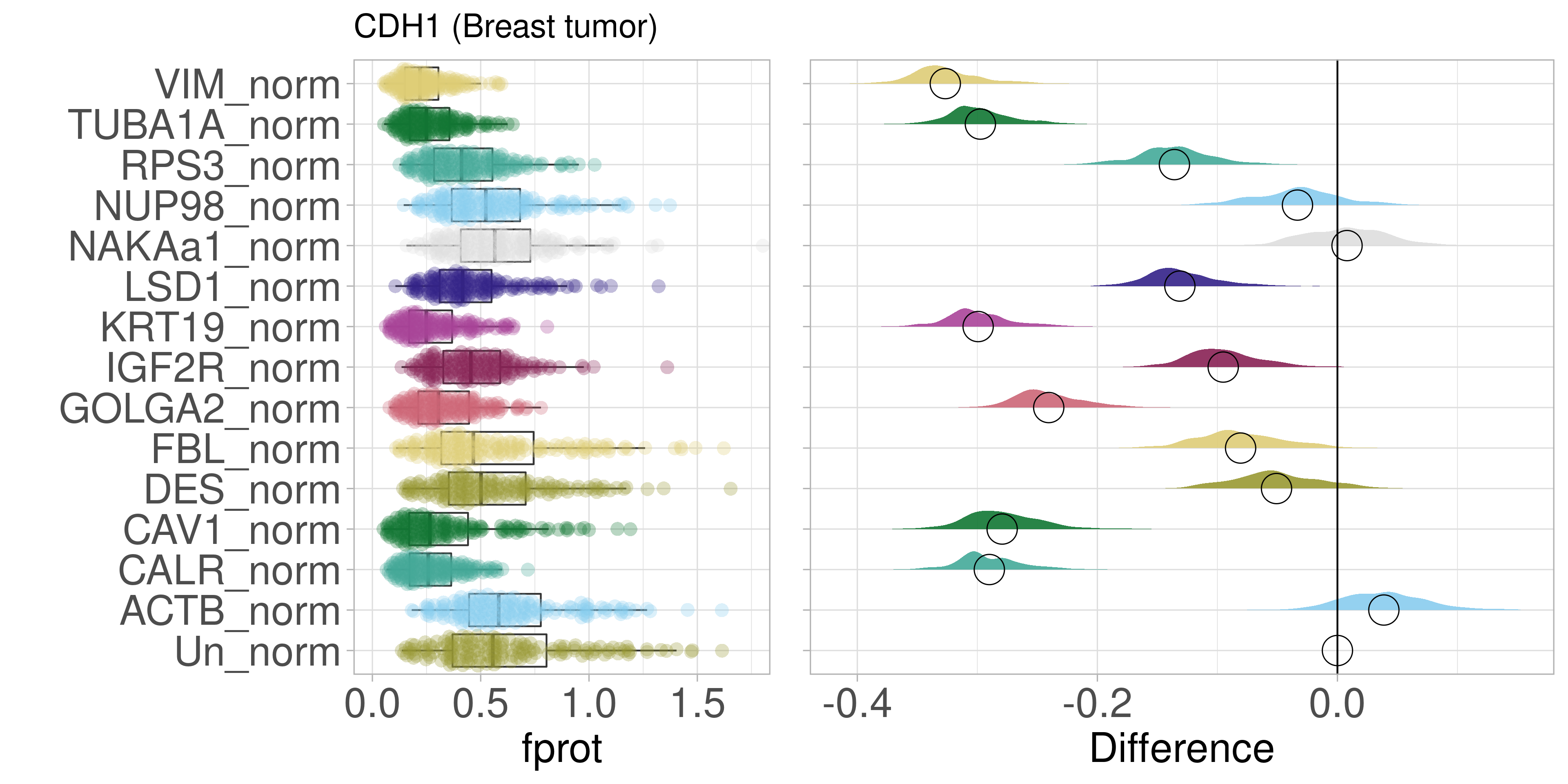

Supplement: Supplementary file 17 — Supplementary Material 17 [file 41598_2026_48754_MOESM17_ESM.zip › RPPA normalizations to cell markers/Breast_Plots/Tumor_suppr_Breast/CDH1_Breast_T.png]

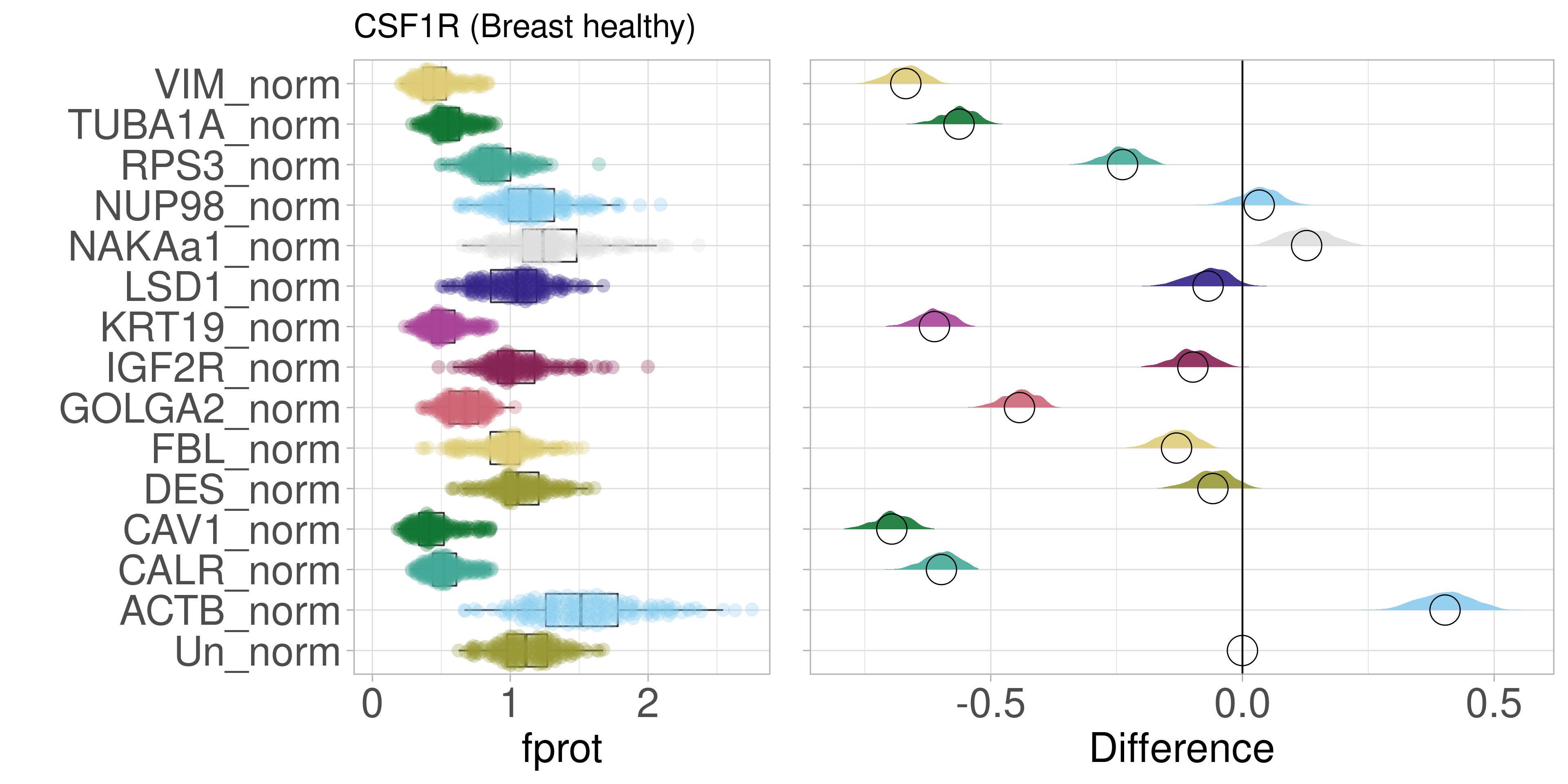

Supplement: Supplementary file 17 — Supplementary Material 17 [file 41598_2026_48754_MOESM17_ESM.zip › RPPA normalizations to cell markers/Breast_Plots/Tumor_suppr_Breast/CSF1R_Breast_H.png]

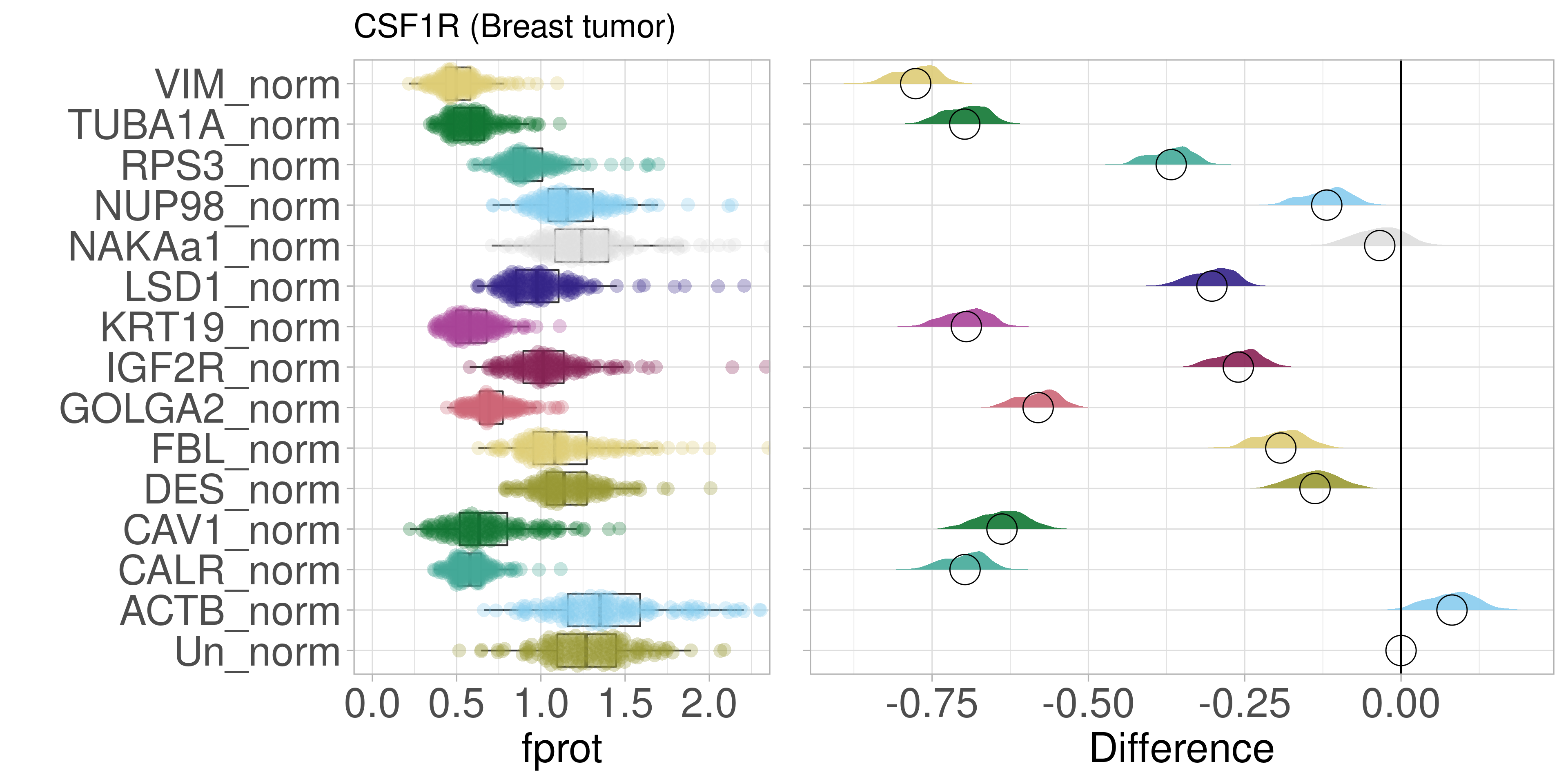

Supplement: Supplementary file 17 — Supplementary Material 17 [file 41598_2026_48754_MOESM17_ESM.zip › RPPA normalizations to cell markers/Breast_Plots/Tumor_suppr_Breast/CSF1R_Breast_T.png]

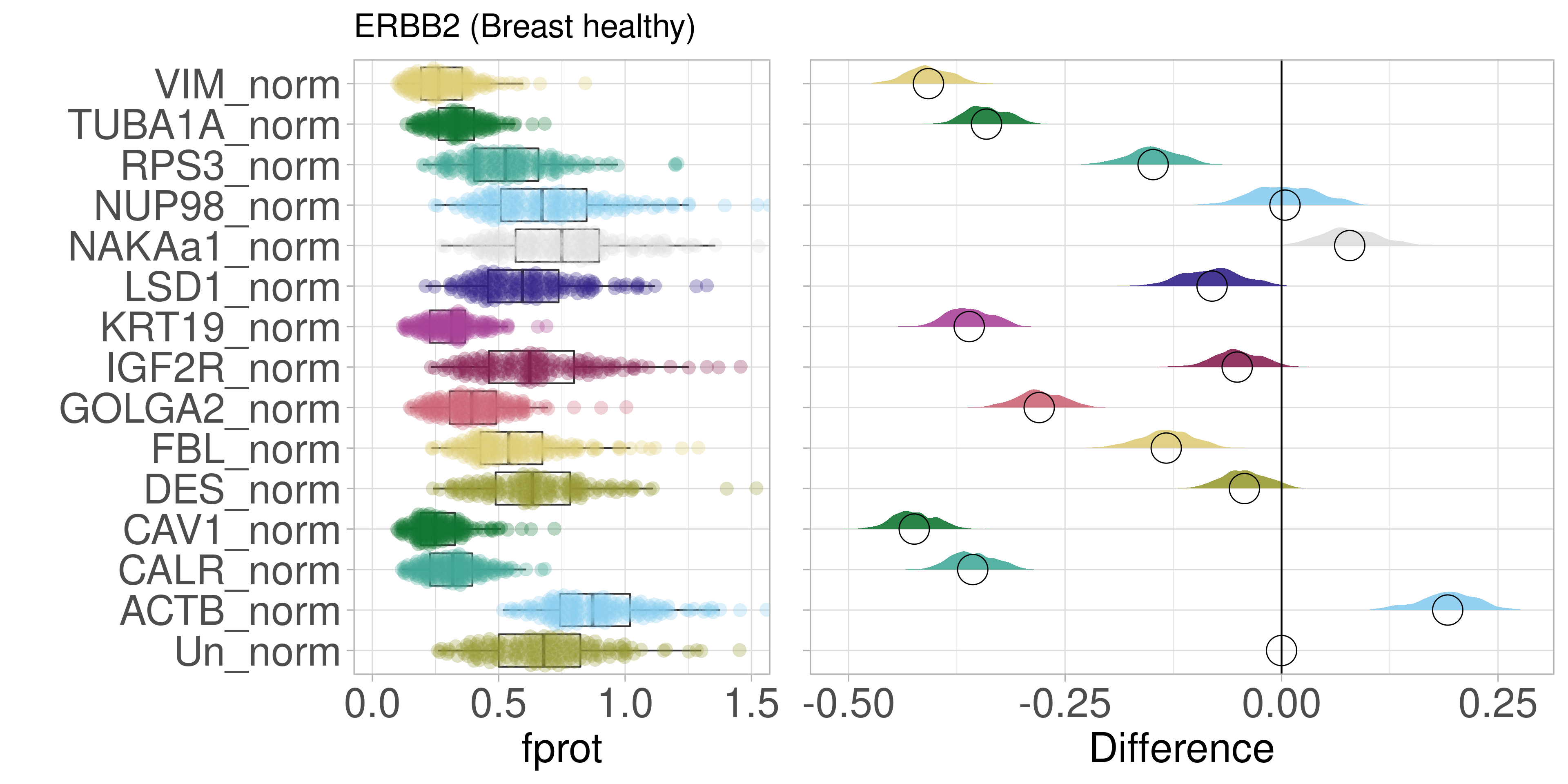

Supplement: Supplementary file 17 — Supplementary Material 17 [file 41598_2026_48754_MOESM17_ESM.zip › RPPA normalizations to cell markers/Breast_Plots/Tumor_suppr_Breast/ERBB2_Breast_H.png]

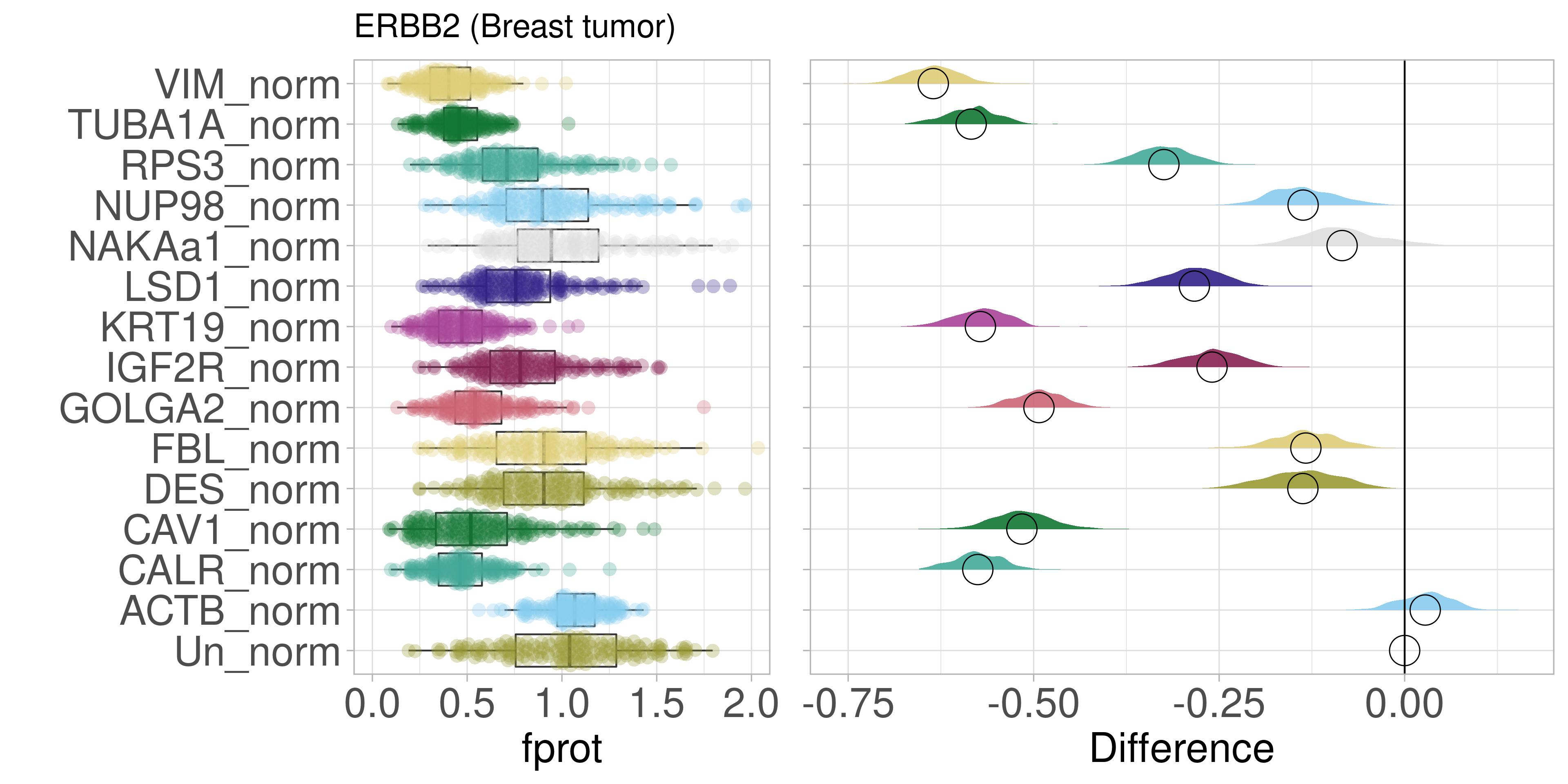

Supplement: Supplementary file 17 — Supplementary Material 17 [file 41598_2026_48754_MOESM17_ESM.zip › RPPA normalizations to cell markers/Breast_Plots/Tumor_suppr_Breast/ERBB2_Breast_T.png]

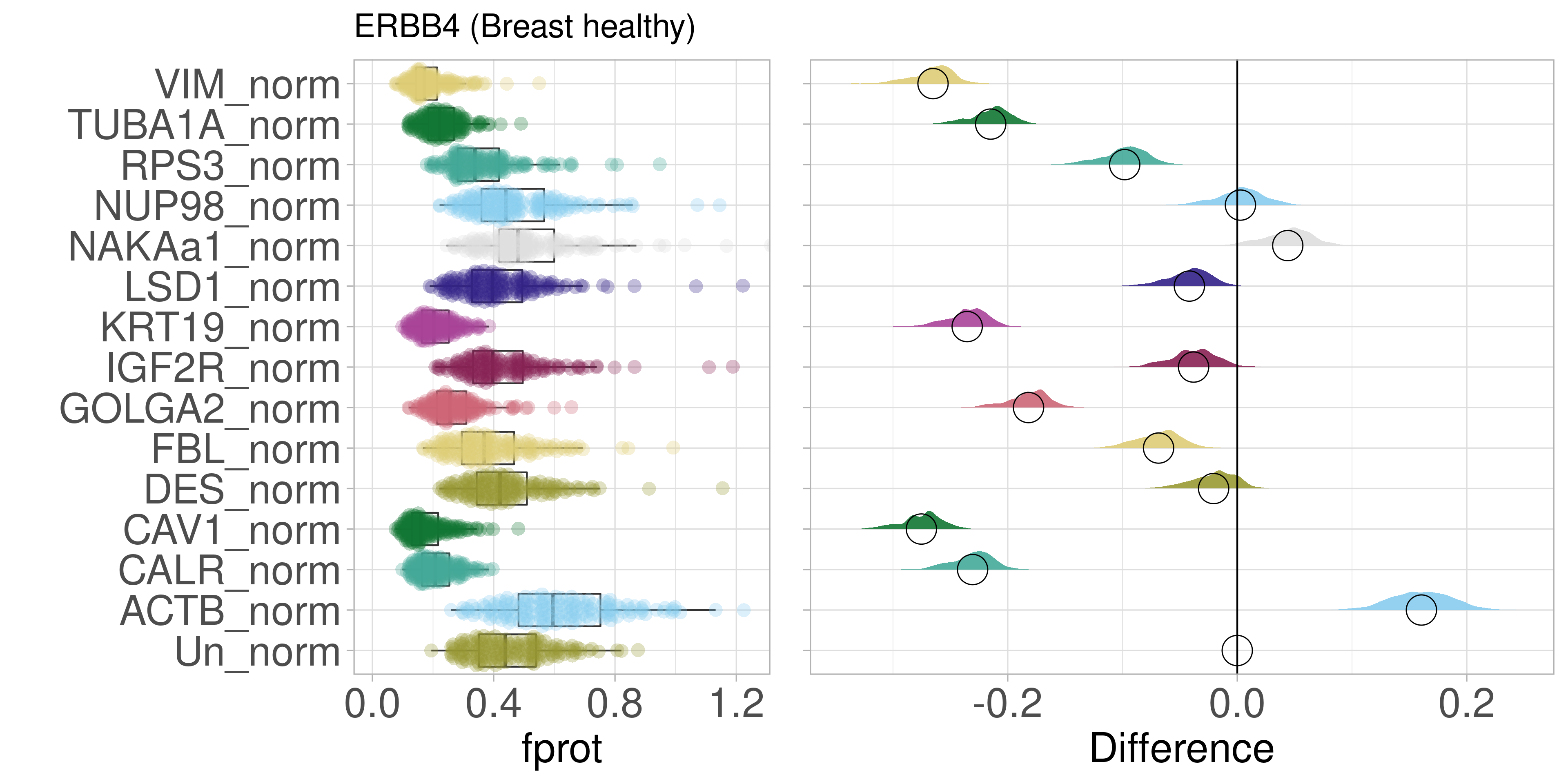

Supplement: Supplementary file 17 — Supplementary Material 17 [file 41598_2026_48754_MOESM17_ESM.zip › RPPA normalizations to cell markers/Breast_Plots/Tumor_suppr_Breast/ERBB4_Breast_H.png]

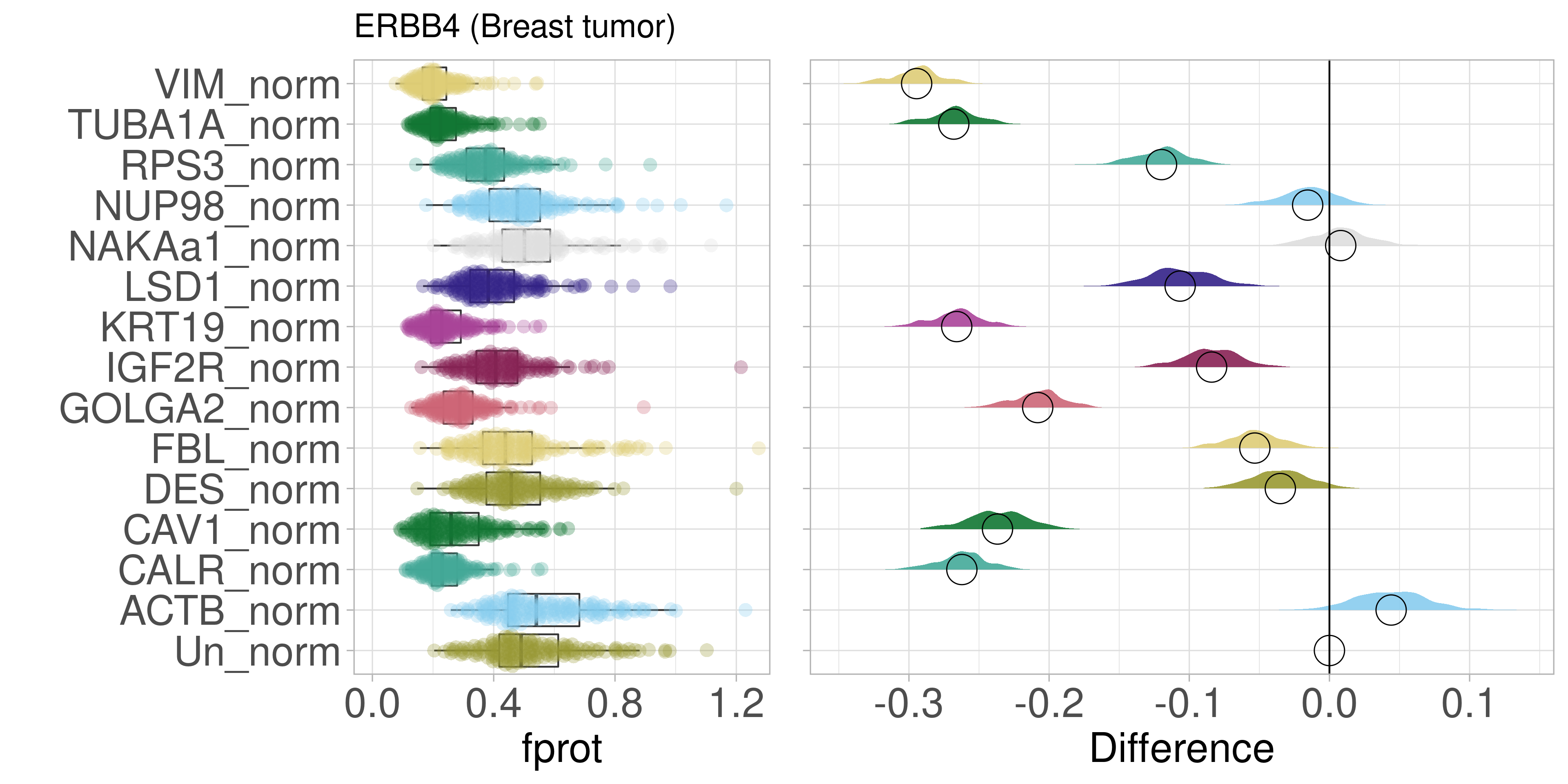

Supplement: Supplementary file 17 — Supplementary Material 17 [file 41598_2026_48754_MOESM17_ESM.zip › RPPA normalizations to cell markers/Breast_Plots/Tumor_suppr_Breast/ERBB4_Breast_T.png]

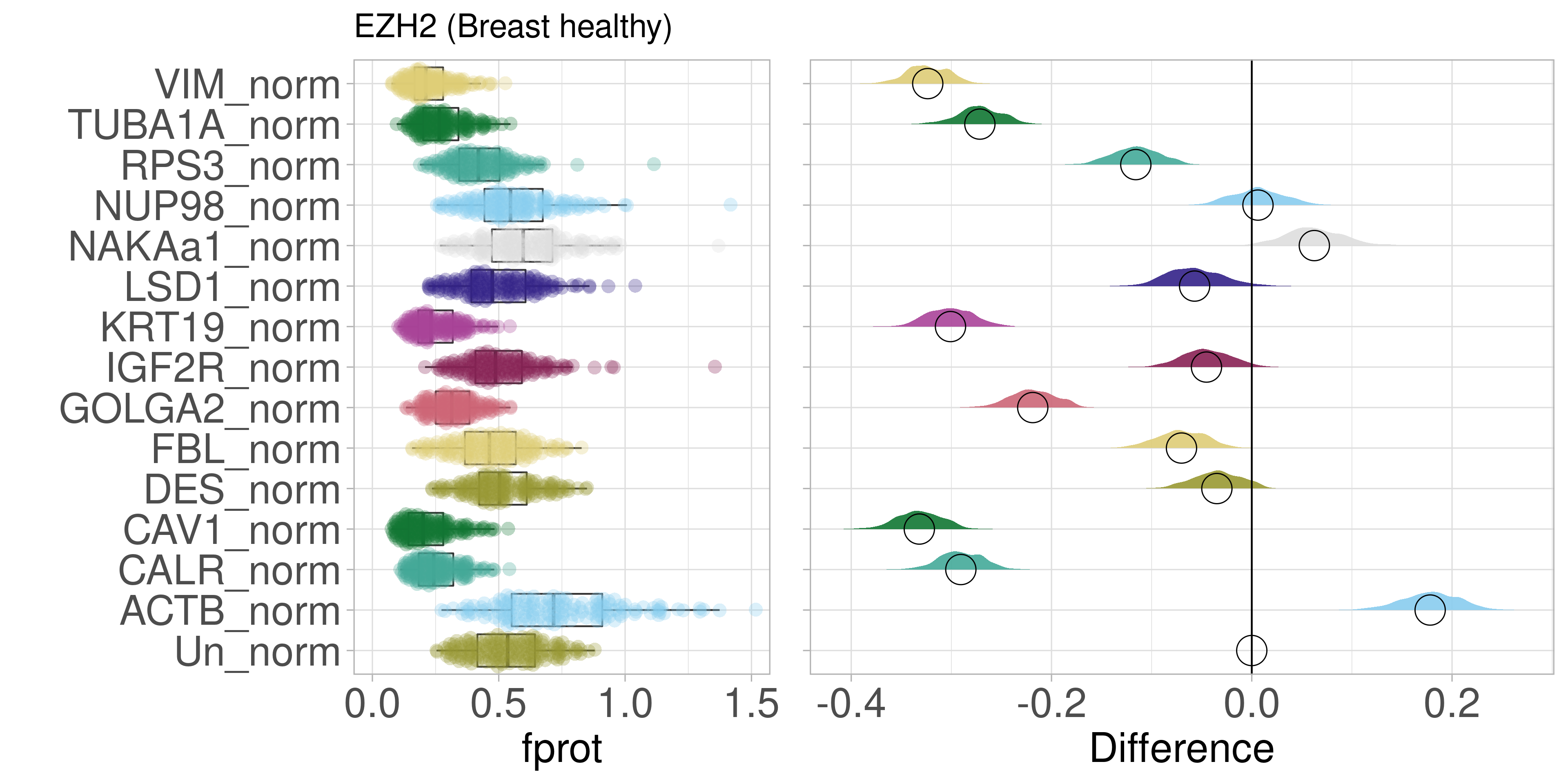

Supplement: Supplementary file 17 — Supplementary Material 17 [file 41598_2026_48754_MOESM17_ESM.zip › RPPA normalizations to cell markers/Breast_Plots/Tumor_suppr_Breast/EZH2_Breast_H.png]

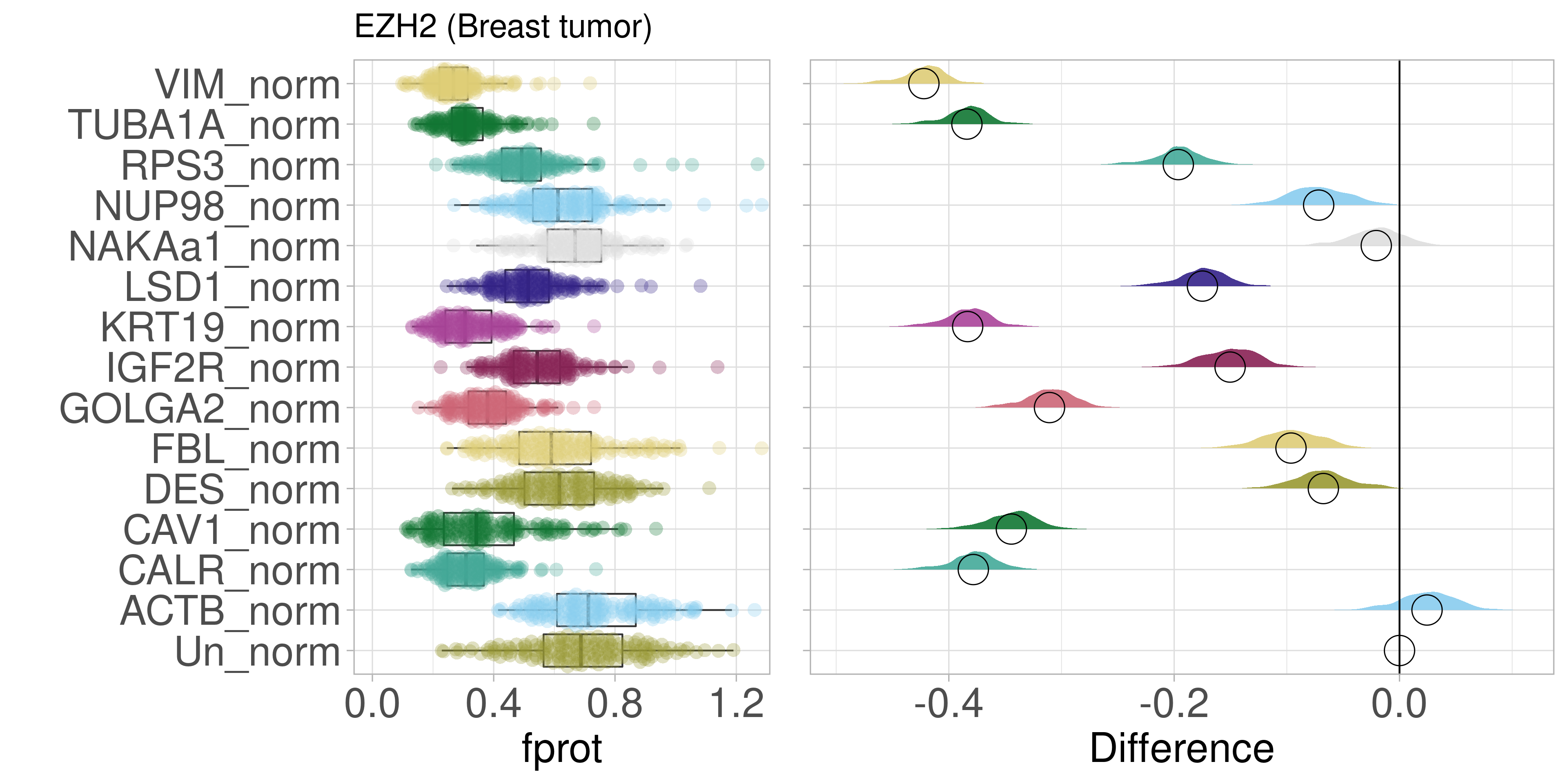

Supplement: Supplementary file 17 — Supplementary Material 17 [file 41598_2026_48754_MOESM17_ESM.zip › RPPA normalizations to cell markers/Breast_Plots/Tumor_suppr_Breast/EZH2_Breast_T.png]

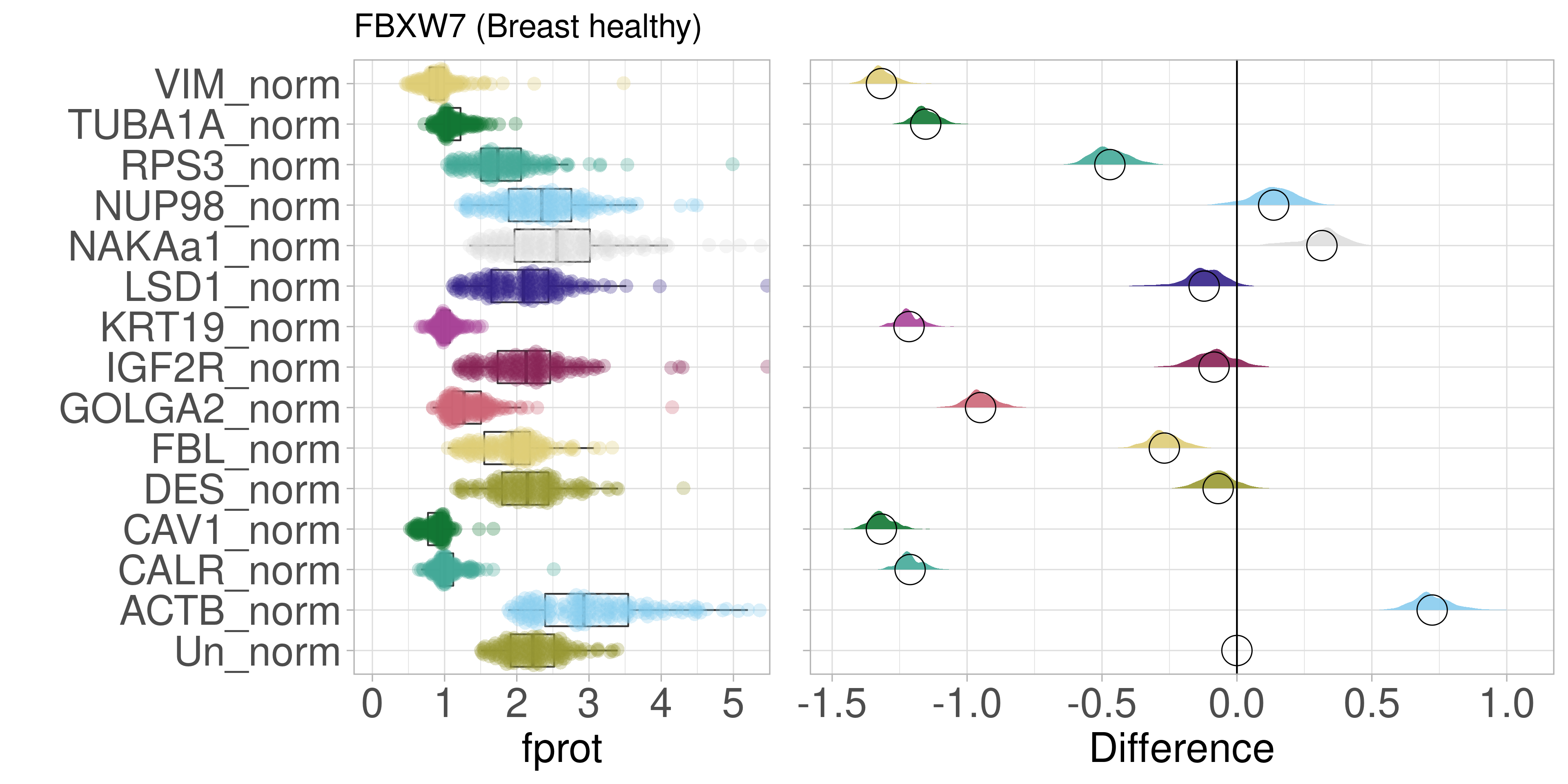

Supplement: Supplementary file 17 — Supplementary Material 17 [file 41598_2026_48754_MOESM17_ESM.zip › RPPA normalizations to cell markers/Breast_Plots/Tumor_suppr_Breast/FBXW7_Breast_H.png]

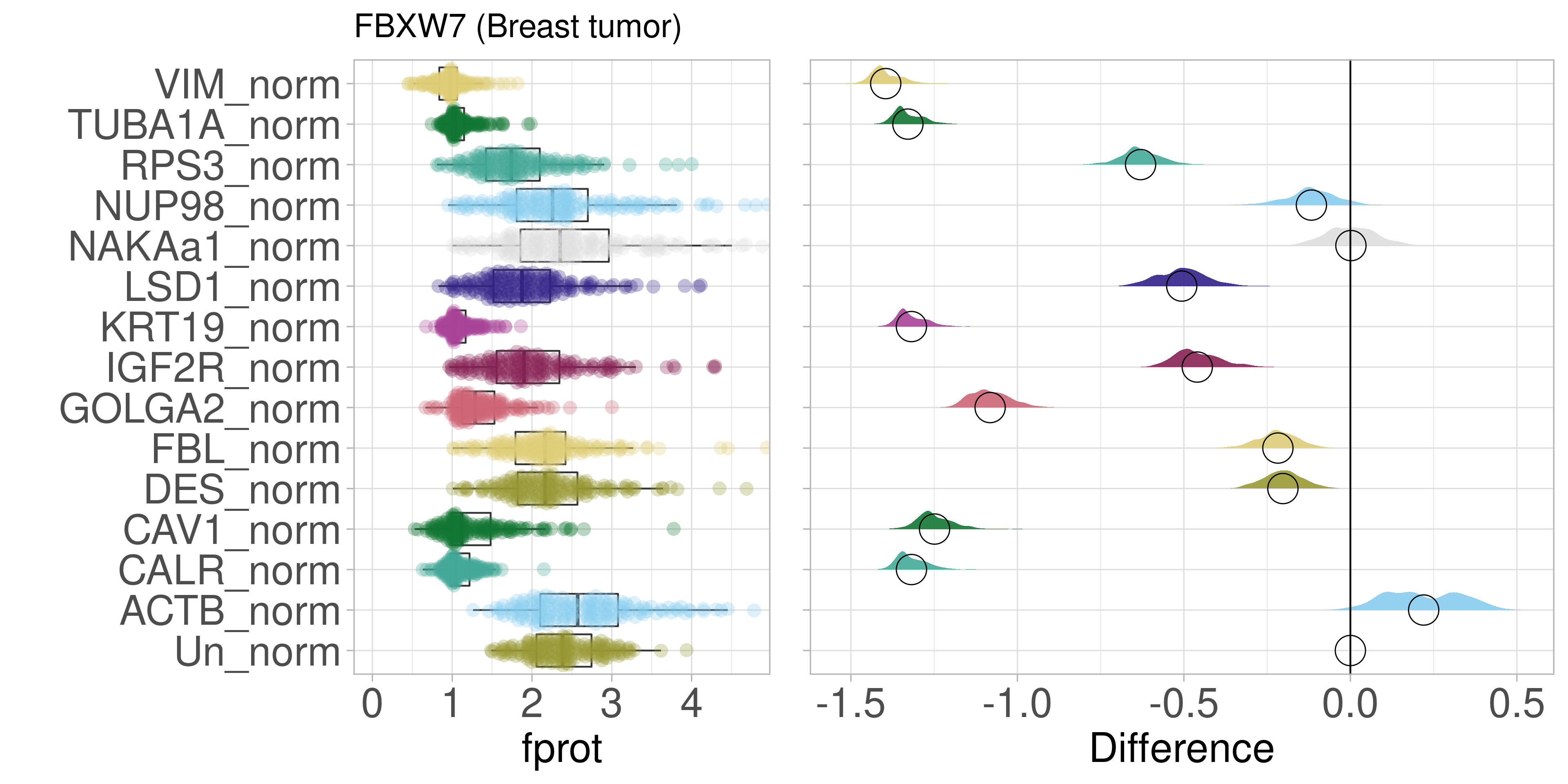

Supplement: Supplementary file 17 — Supplementary Material 17 [file 41598_2026_48754_MOESM17_ESM.zip › RPPA normalizations to cell markers/Breast_Plots/Tumor_suppr_Breast/FBXW7_Breast_T.png]

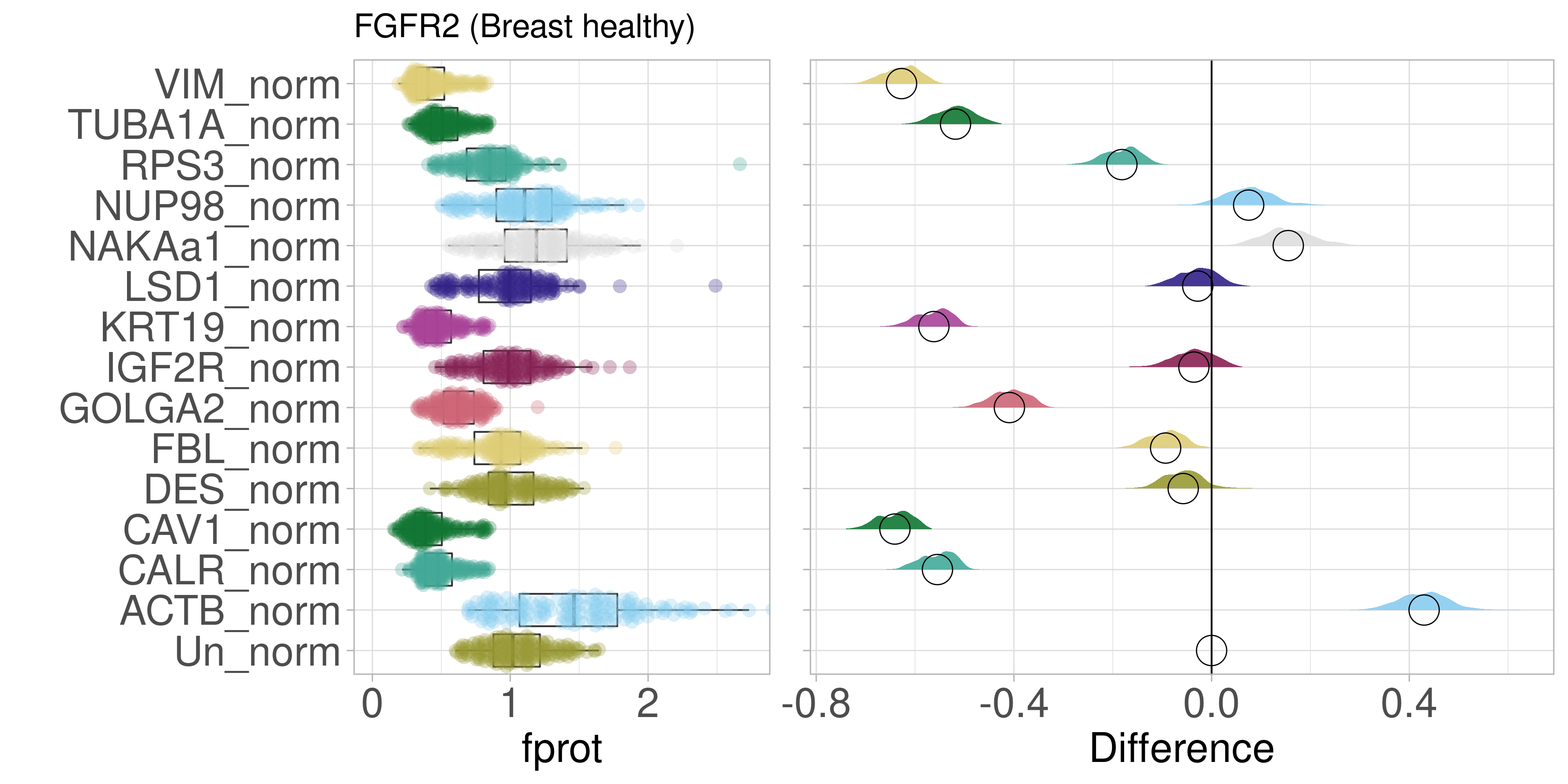

Supplement: Supplementary file 17 — Supplementary Material 17 [file 41598_2026_48754_MOESM17_ESM.zip › RPPA normalizations to cell markers/Breast_Plots/Tumor_suppr_Breast/FGFR2_Breast_H.png]

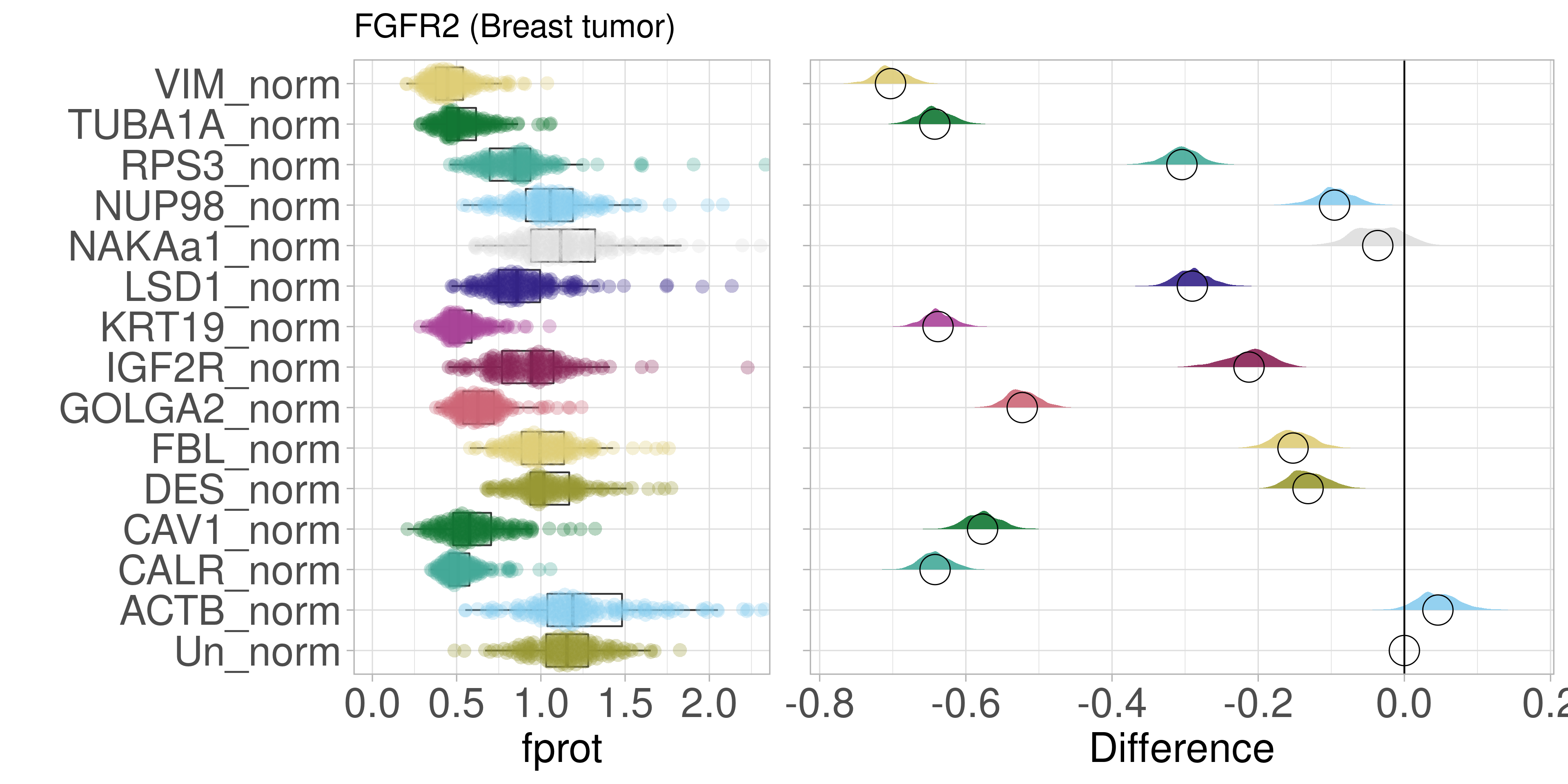

Supplement: Supplementary file 17 — Supplementary Material 17 [file 41598_2026_48754_MOESM17_ESM.zip › RPPA normalizations to cell markers/Breast_Plots/Tumor_suppr_Breast/FGFR2_Breast_T.png]

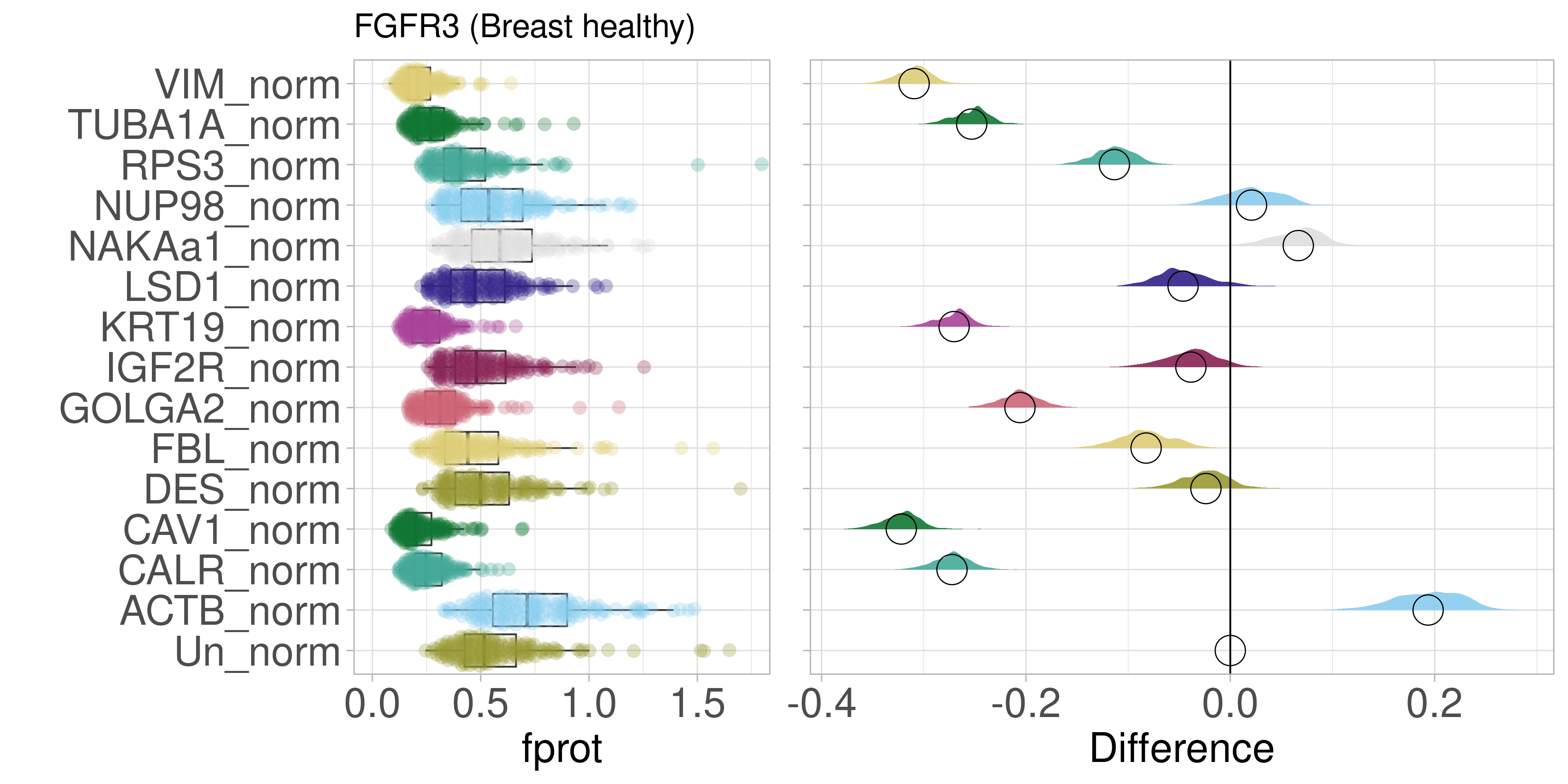

Supplement: Supplementary file 17 — Supplementary Material 17 [file 41598_2026_48754_MOESM17_ESM.zip › RPPA normalizations to cell markers/Breast_Plots/Tumor_suppr_Breast/FGFR3_Breast_H.png]

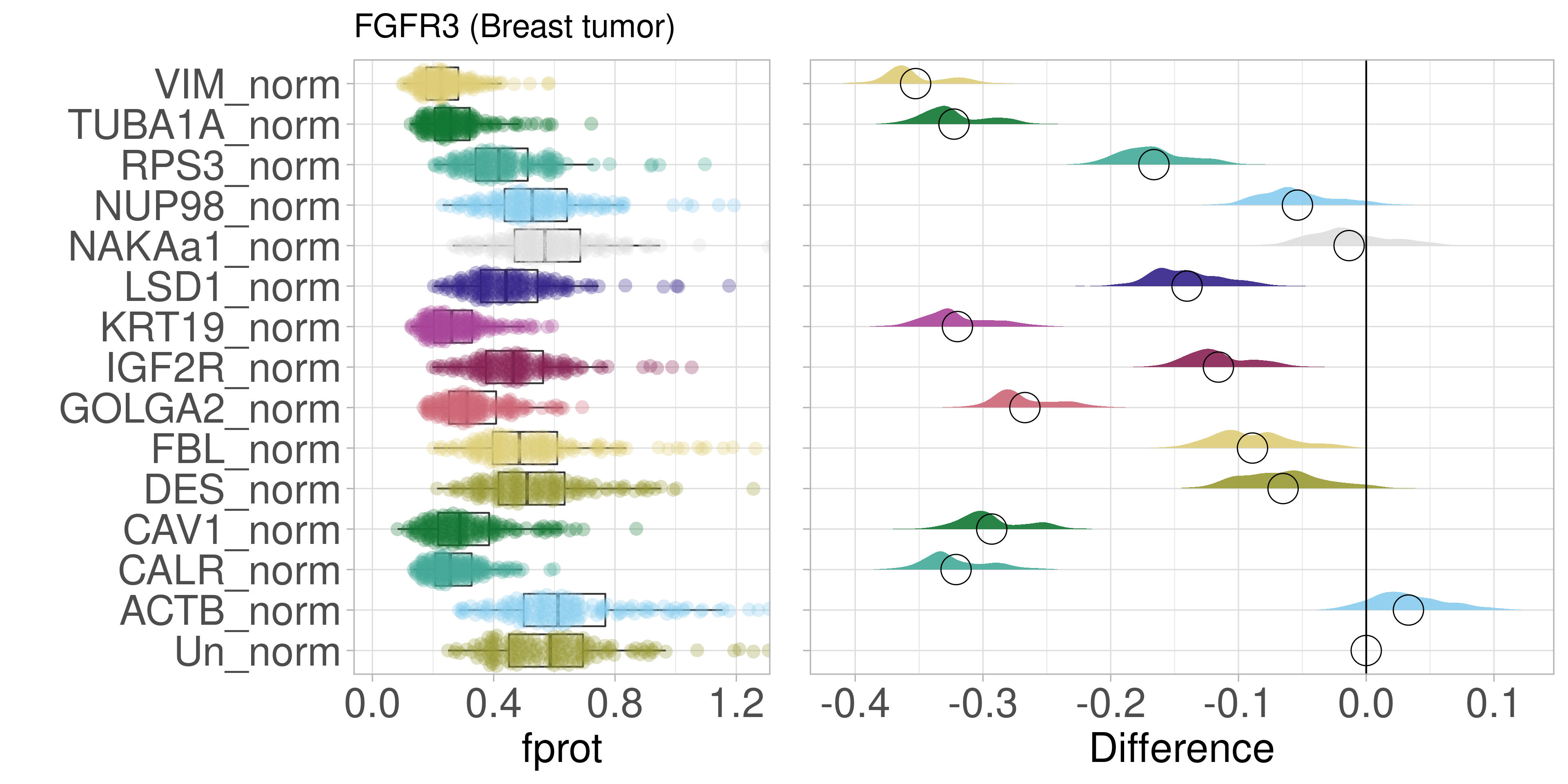

Supplement: Supplementary file 17 — Supplementary Material 17 [file 41598_2026_48754_MOESM17_ESM.zip › RPPA normalizations to cell markers/Breast_Plots/Tumor_suppr_Breast/FGFR3_Breast_T.png]

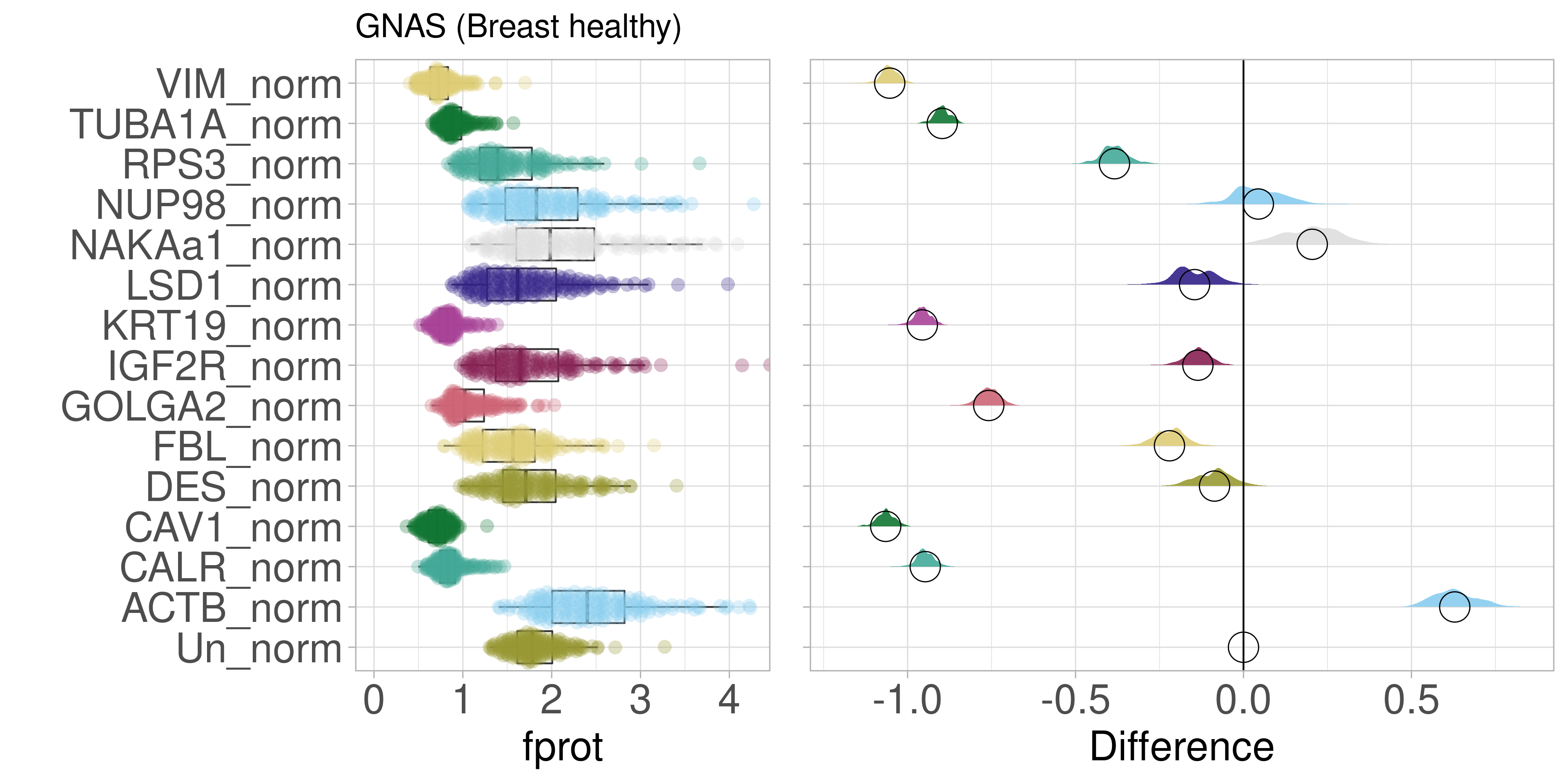

Supplement: Supplementary file 17 — Supplementary Material 17 [file 41598_2026_48754_MOESM17_ESM.zip › RPPA normalizations to cell markers/Breast_Plots/Tumor_suppr_Breast/GNAS_Breast_H.png]

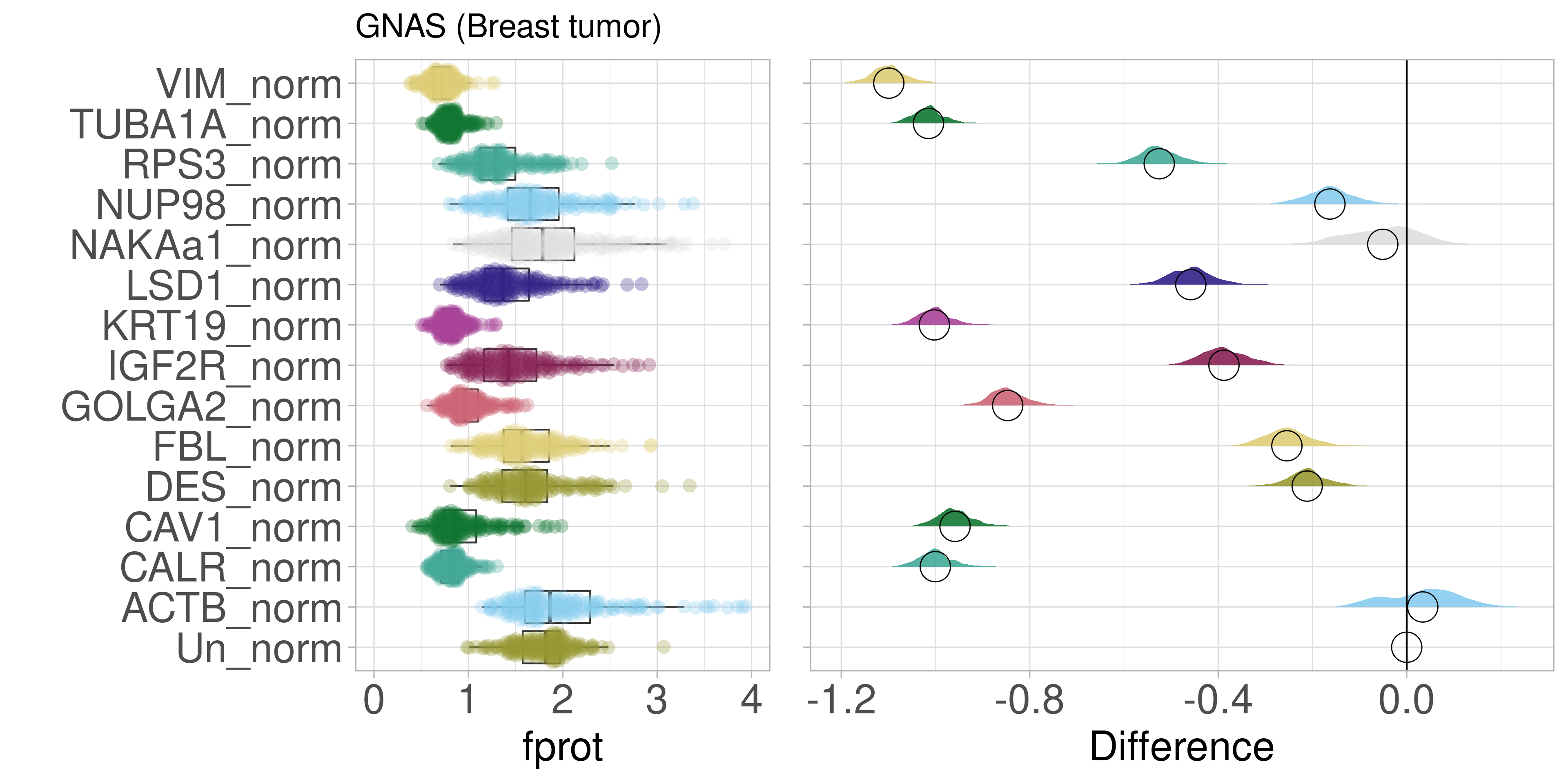

Supplement: Supplementary file 17 — Supplementary Material 17 [file 41598_2026_48754_MOESM17_ESM.zip › RPPA normalizations to cell markers/Breast_Plots/Tumor_suppr_Breast/GNAS_Breast_T.png]

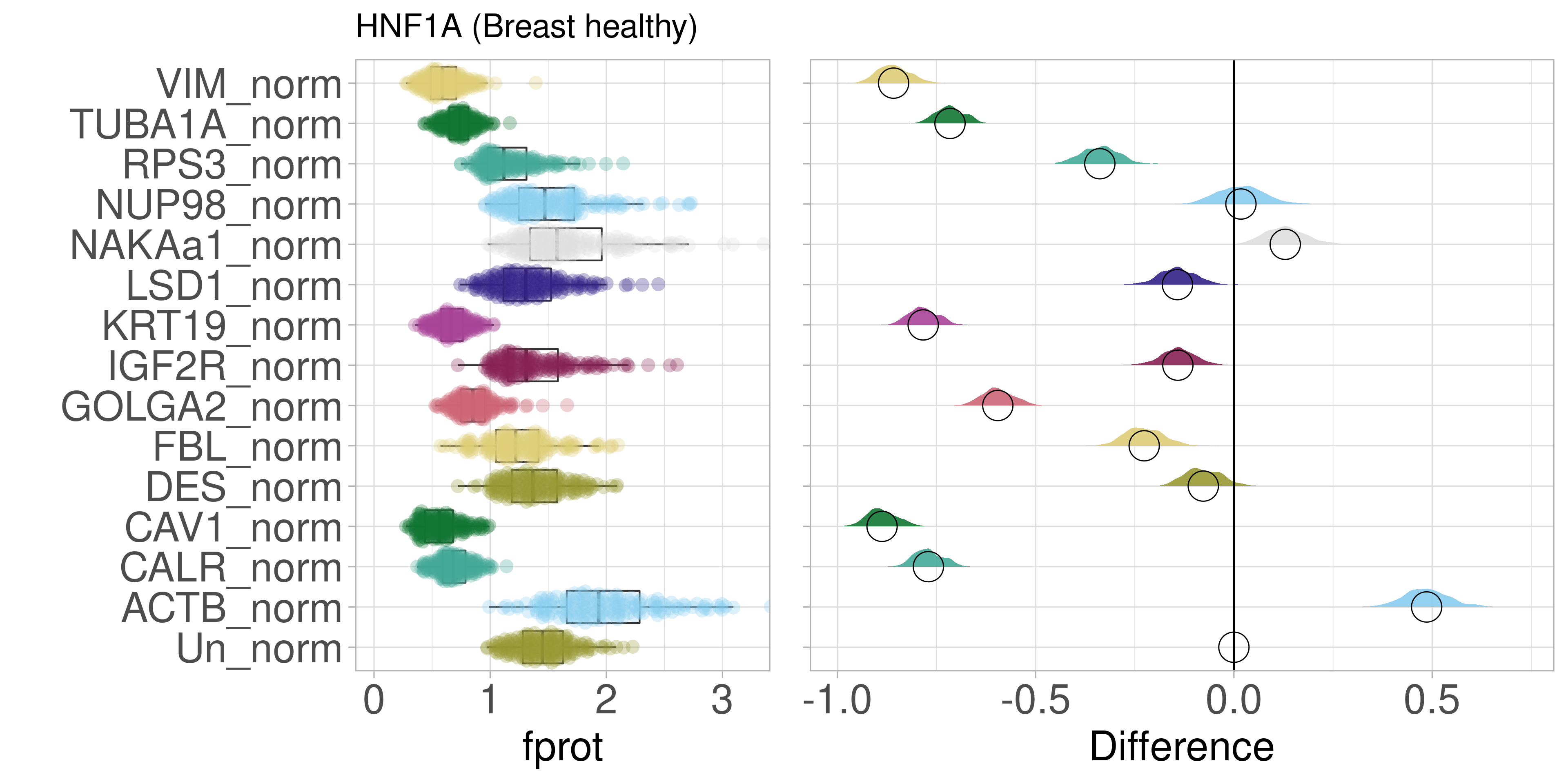

Supplement: Supplementary file 17 — Supplementary Material 17 [file 41598_2026_48754_MOESM17_ESM.zip › RPPA normalizations to cell markers/Breast_Plots/Tumor_suppr_Breast/HNF1A_Breast_H.png]

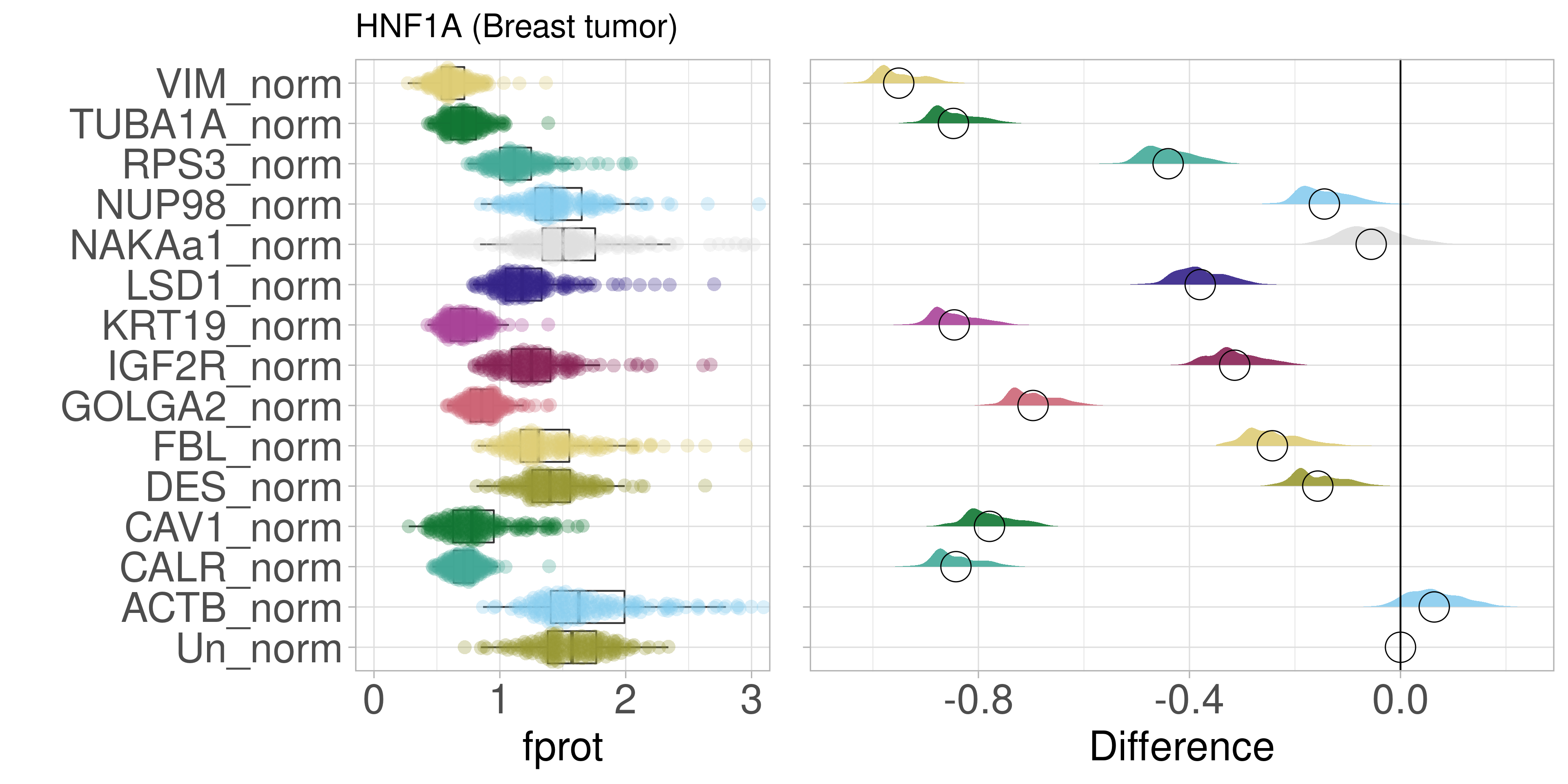

Supplement: Supplementary file 17 — Supplementary Material 17 [file 41598_2026_48754_MOESM17_ESM.zip › RPPA normalizations to cell markers/Breast_Plots/Tumor_suppr_Breast/HNF1A_Breast_T.png]

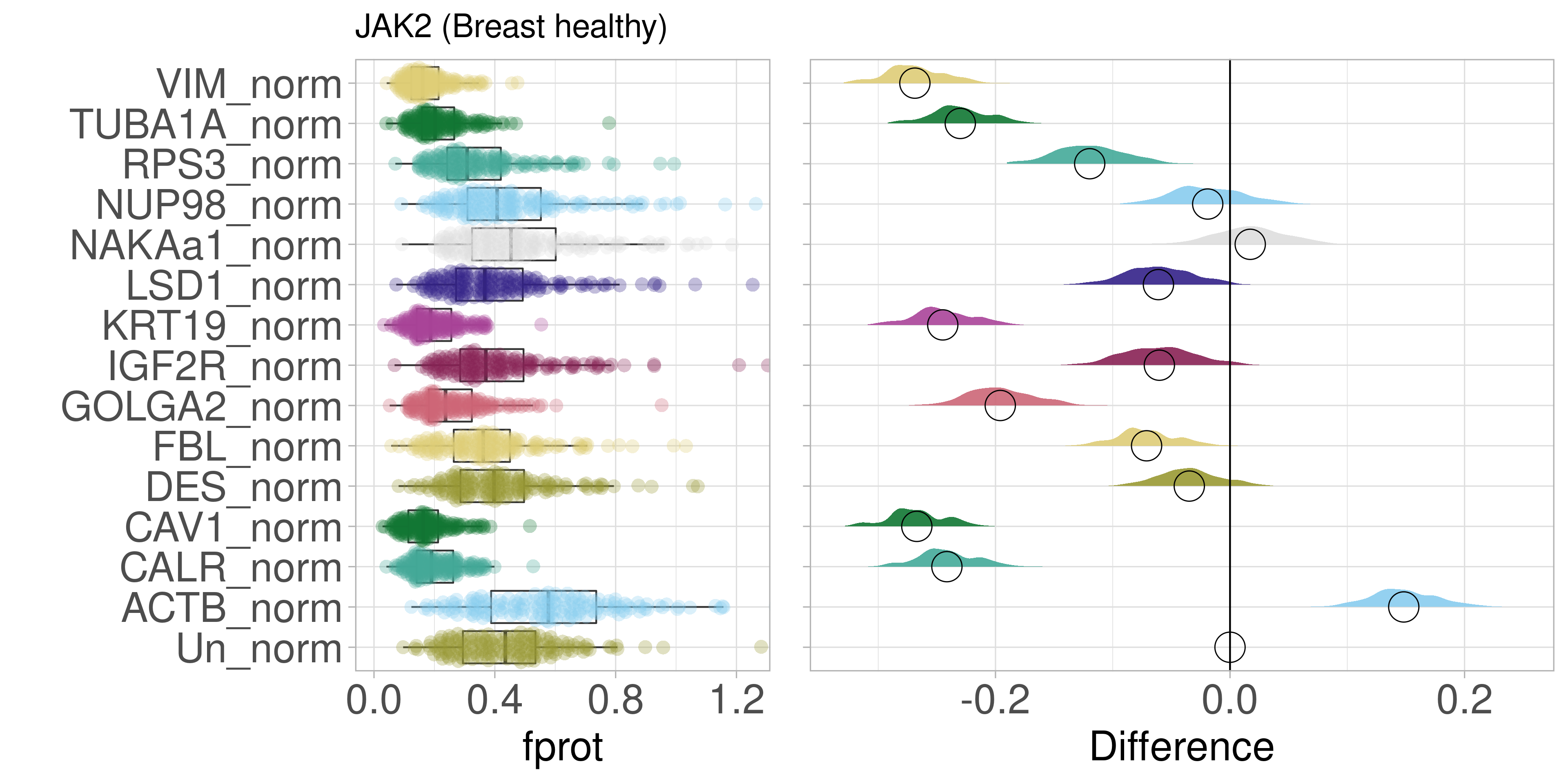

Supplement: Supplementary file 17 — Supplementary Material 17 [file 41598_2026_48754_MOESM17_ESM.zip › RPPA normalizations to cell markers/Breast_Plots/Tumor_suppr_Breast/JAK2_Breast_H.png]

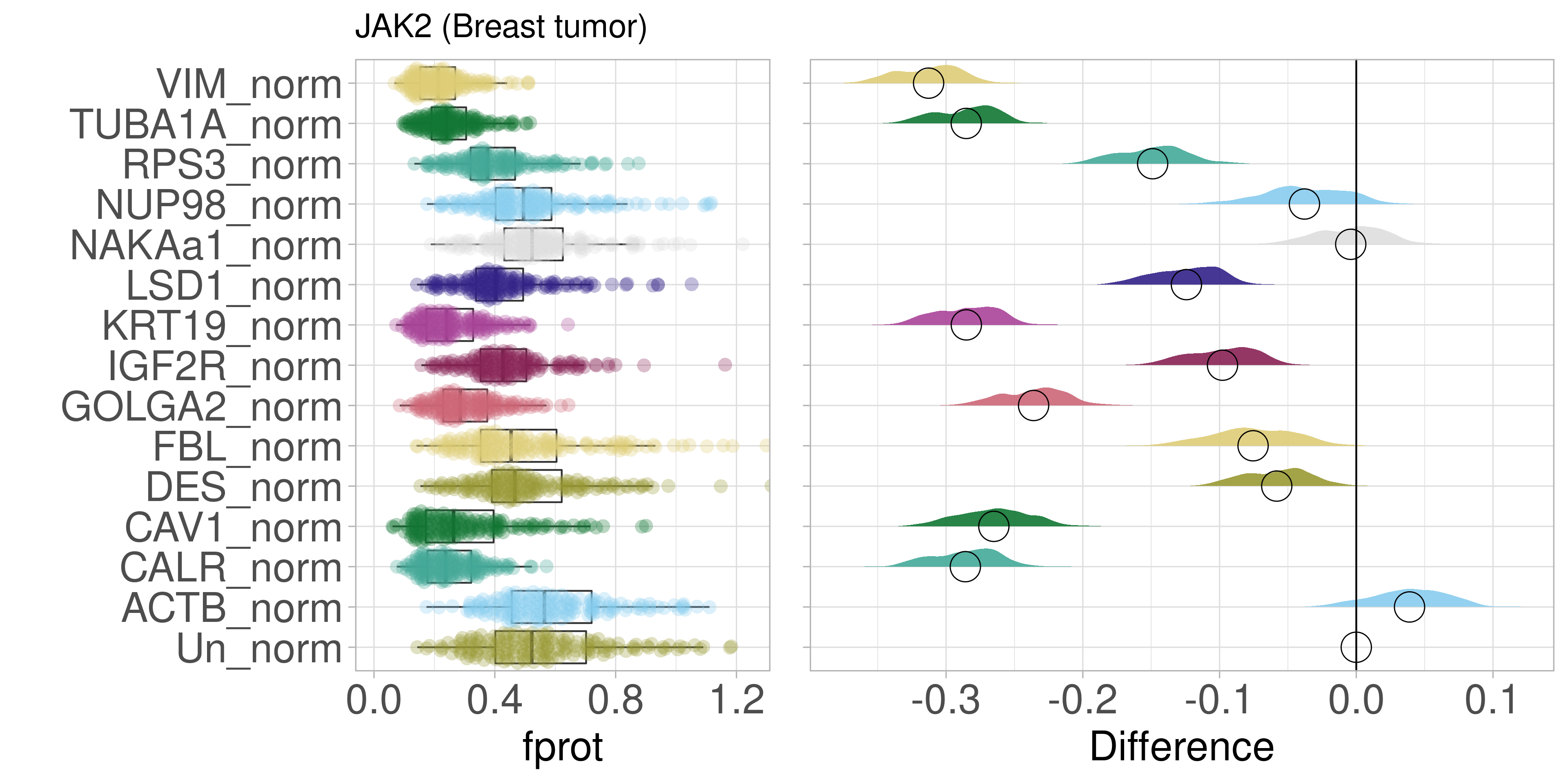

Supplement: Supplementary file 17 — Supplementary Material 17 [file 41598_2026_48754_MOESM17_ESM.zip › RPPA normalizations to cell markers/Breast_Plots/Tumor_suppr_Breast/JAK2_Breast_T.png]

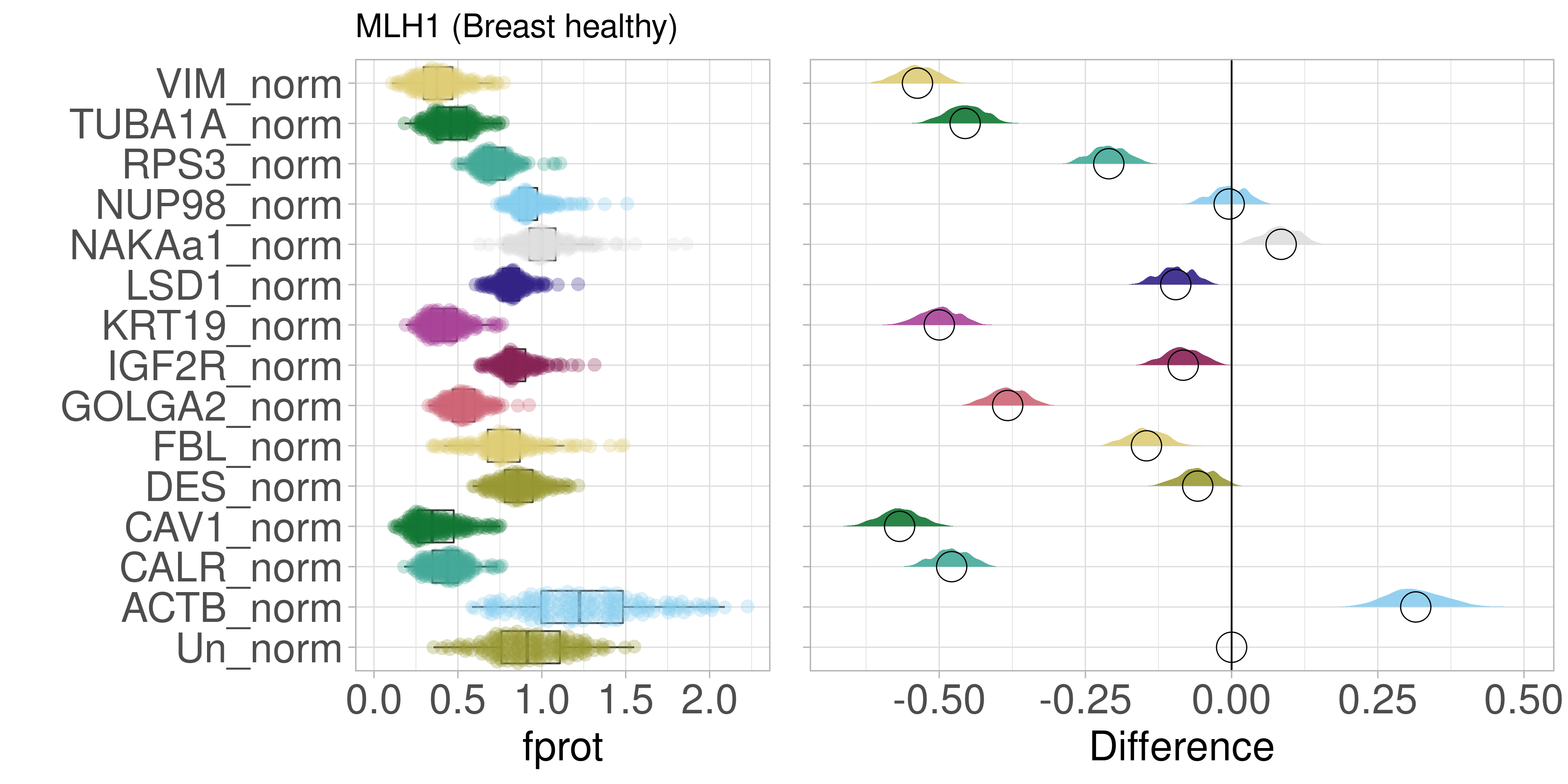

Supplement: Supplementary file 17 — Supplementary Material 17 [file 41598_2026_48754_MOESM17_ESM.zip › RPPA normalizations to cell markers/Breast_Plots/Tumor_suppr_Breast/MLH1_Breast_H.png]

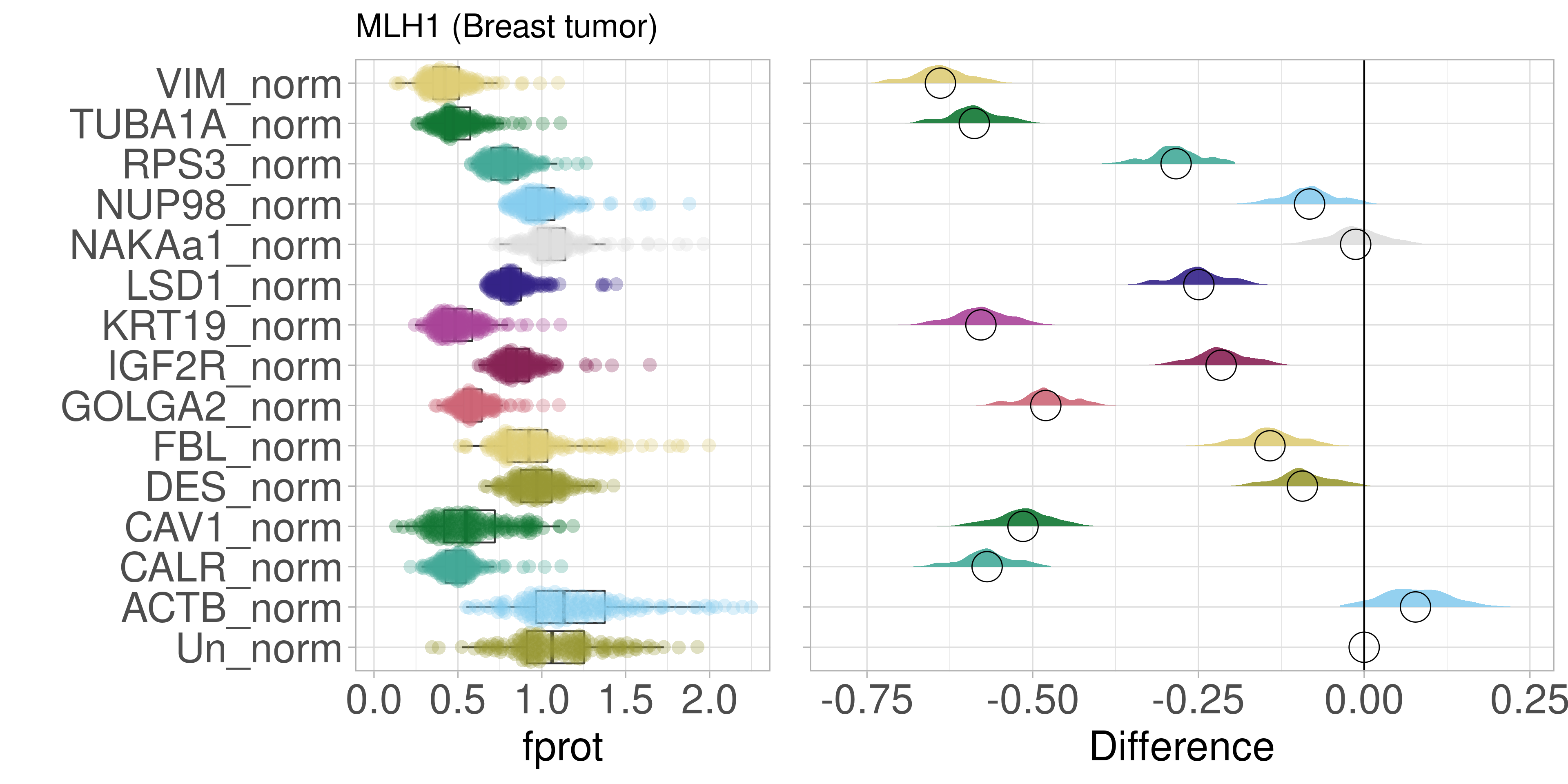

Supplement: Supplementary file 17 — Supplementary Material 17 [file 41598_2026_48754_MOESM17_ESM.zip › RPPA normalizations to cell markers/Breast_Plots/Tumor_suppr_Breast/MLH1_Breast_T.png]

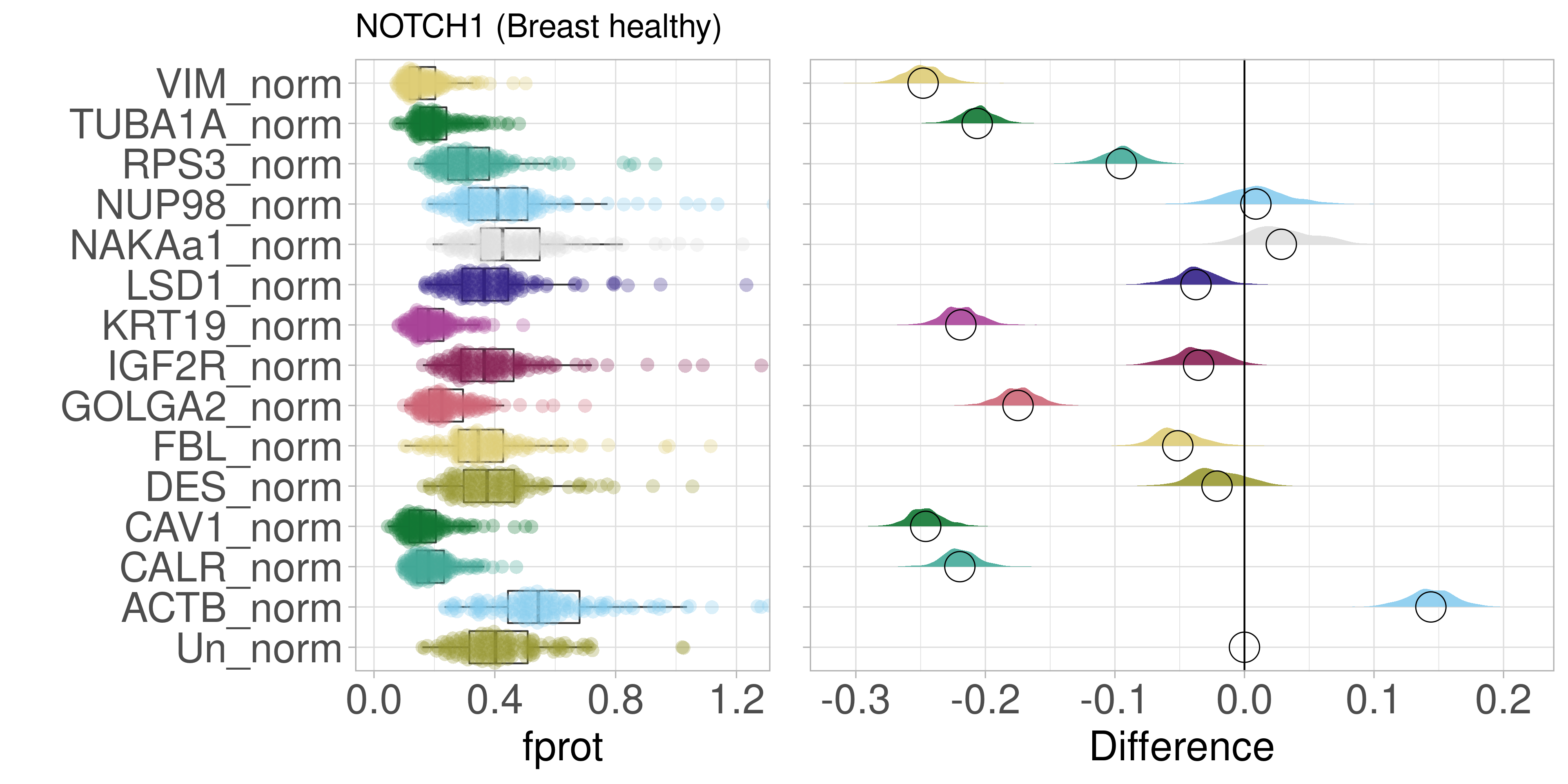

Supplement: Supplementary file 17 — Supplementary Material 17 [file 41598_2026_48754_MOESM17_ESM.zip › RPPA normalizations to cell markers/Breast_Plots/Tumor_suppr_Breast/NOTCH1_Breast_H.png]

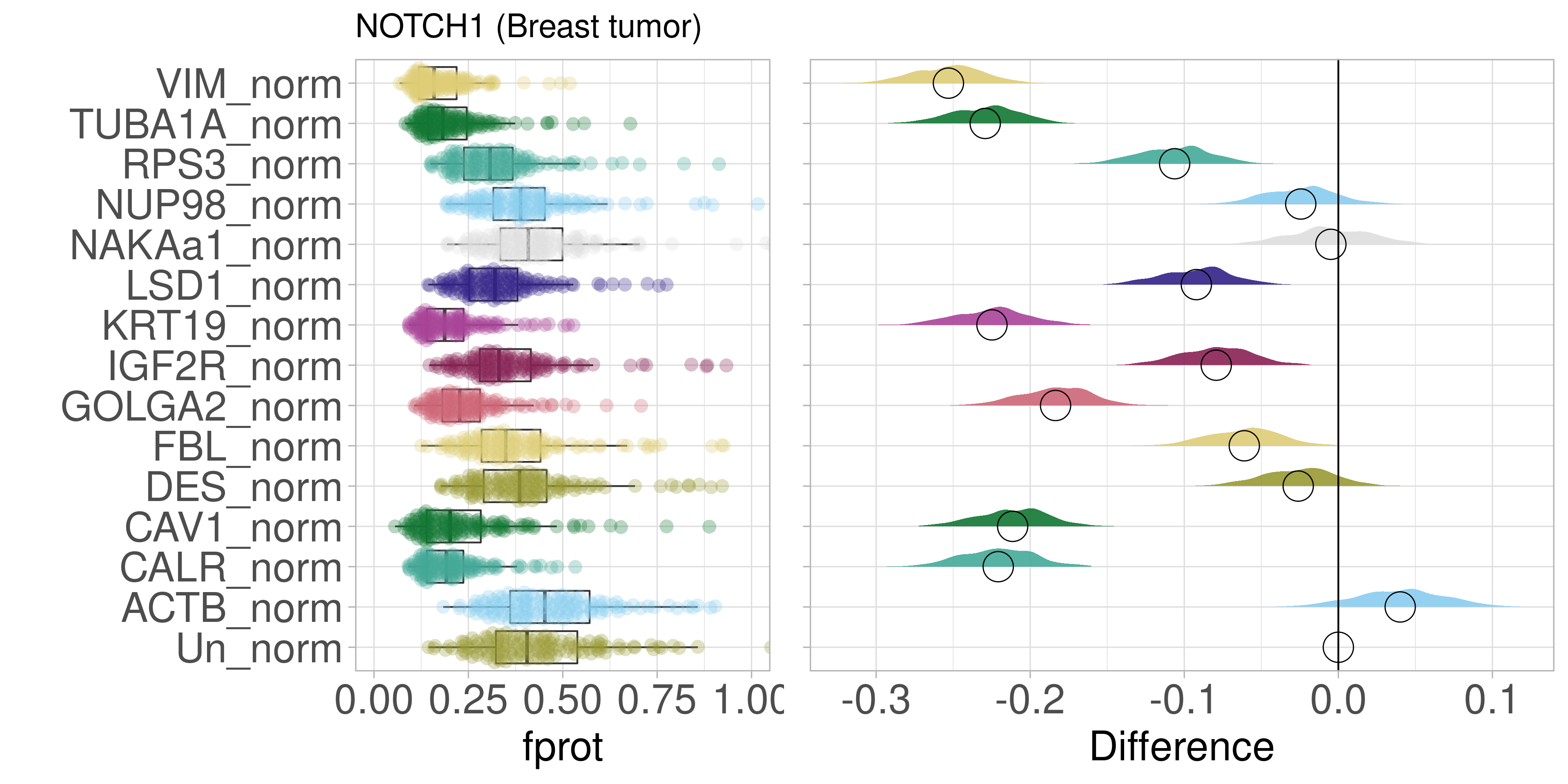

Supplement: Supplementary file 17 — Supplementary Material 17 [file 41598_2026_48754_MOESM17_ESM.zip › RPPA normalizations to cell markers/Breast_Plots/Tumor_suppr_Breast/NOTCH1_Breast_T.png]
