# Supplementary figures and images for: Comparative analysis and correlation of cancer hotspot proteins and cell markers in tumor-normal adjacent breast and kidney samples using RPPA and LC-MS (part 3 of 3)
Source: Sci Rep. 2026 May 18;16:22442. doi: 10.1038/s41598-026-48754-2 (PMC13377106; doi:10.1038/s41598-026-48754-2)

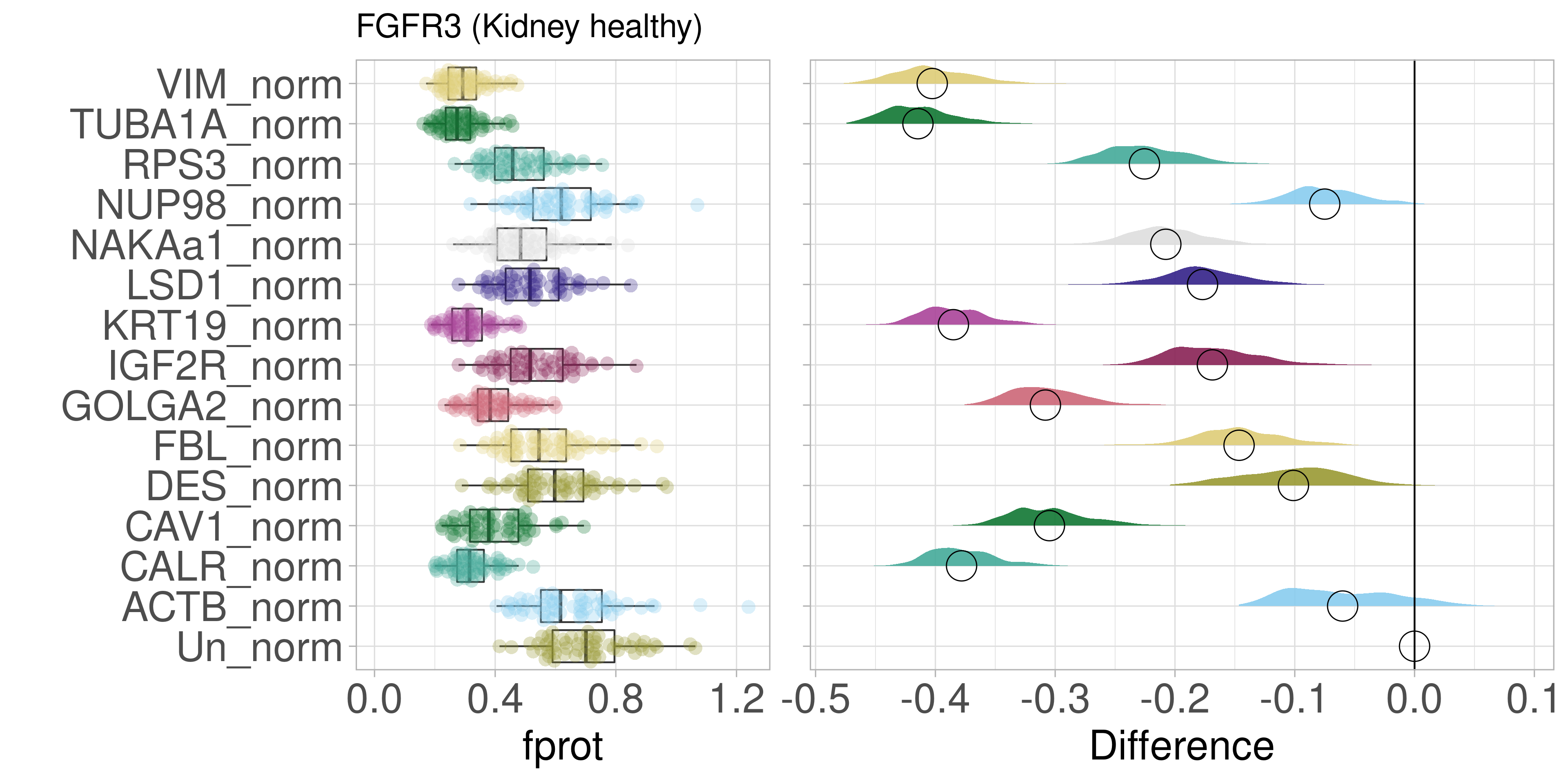

Supplement: Supplementary file 17 — Supplementary Material 17 [file 41598_2026_48754_MOESM17_ESM.zip › RPPA normalizations to cell markers/Kidney_plots/Tumor_suppr_Kidney/FGFR3_Kidney_H.png]

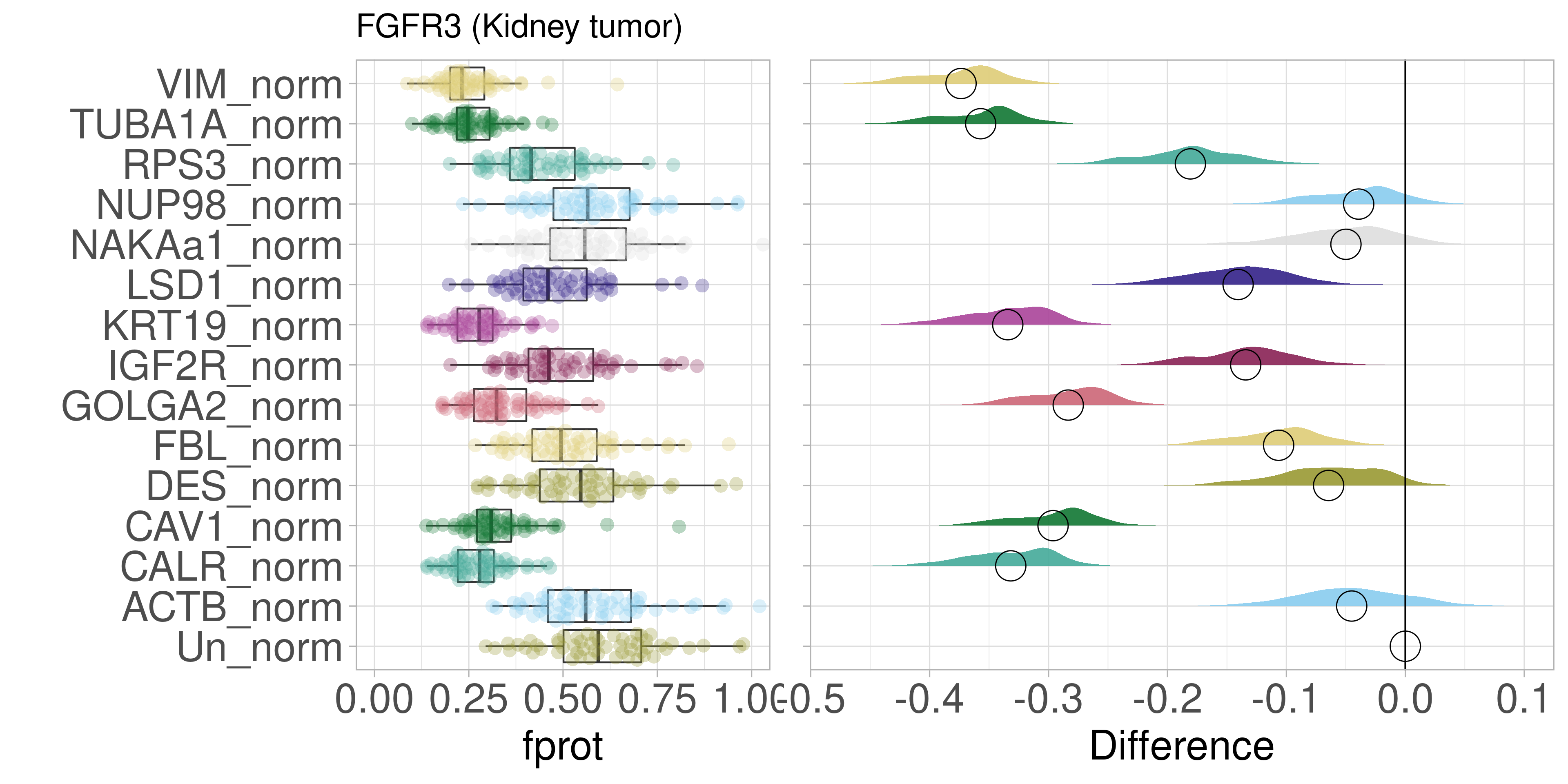

Supplement: Supplementary file 17 — Supplementary Material 17 [file 41598_2026_48754_MOESM17_ESM.zip › RPPA normalizations to cell markers/Kidney_plots/Tumor_suppr_Kidney/FGFR3_Kidney_T.png]

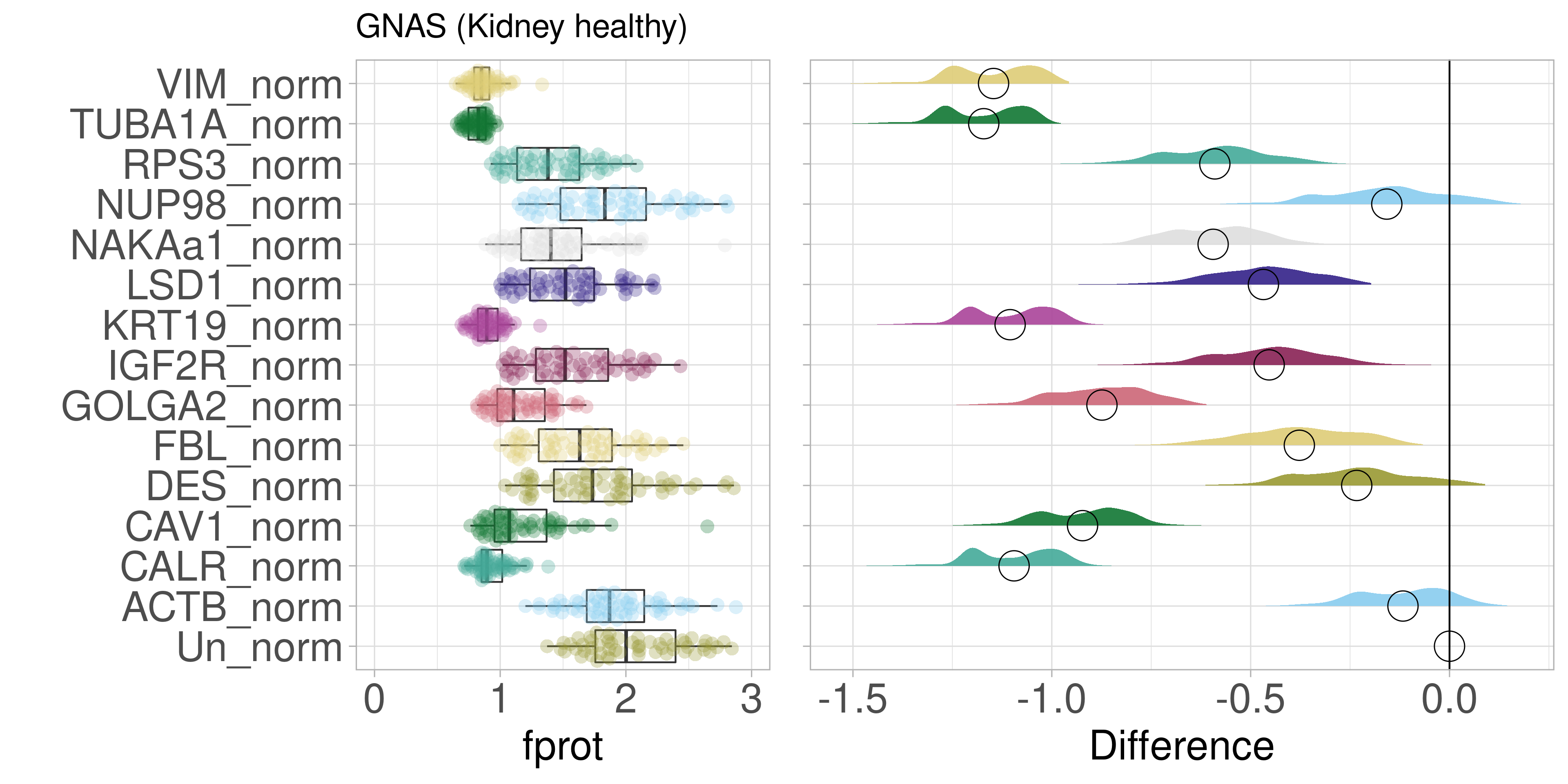

Supplement: Supplementary file 17 — Supplementary Material 17 [file 41598_2026_48754_MOESM17_ESM.zip › RPPA normalizations to cell markers/Kidney_plots/Tumor_suppr_Kidney/GNAS_Kidney_H.png]

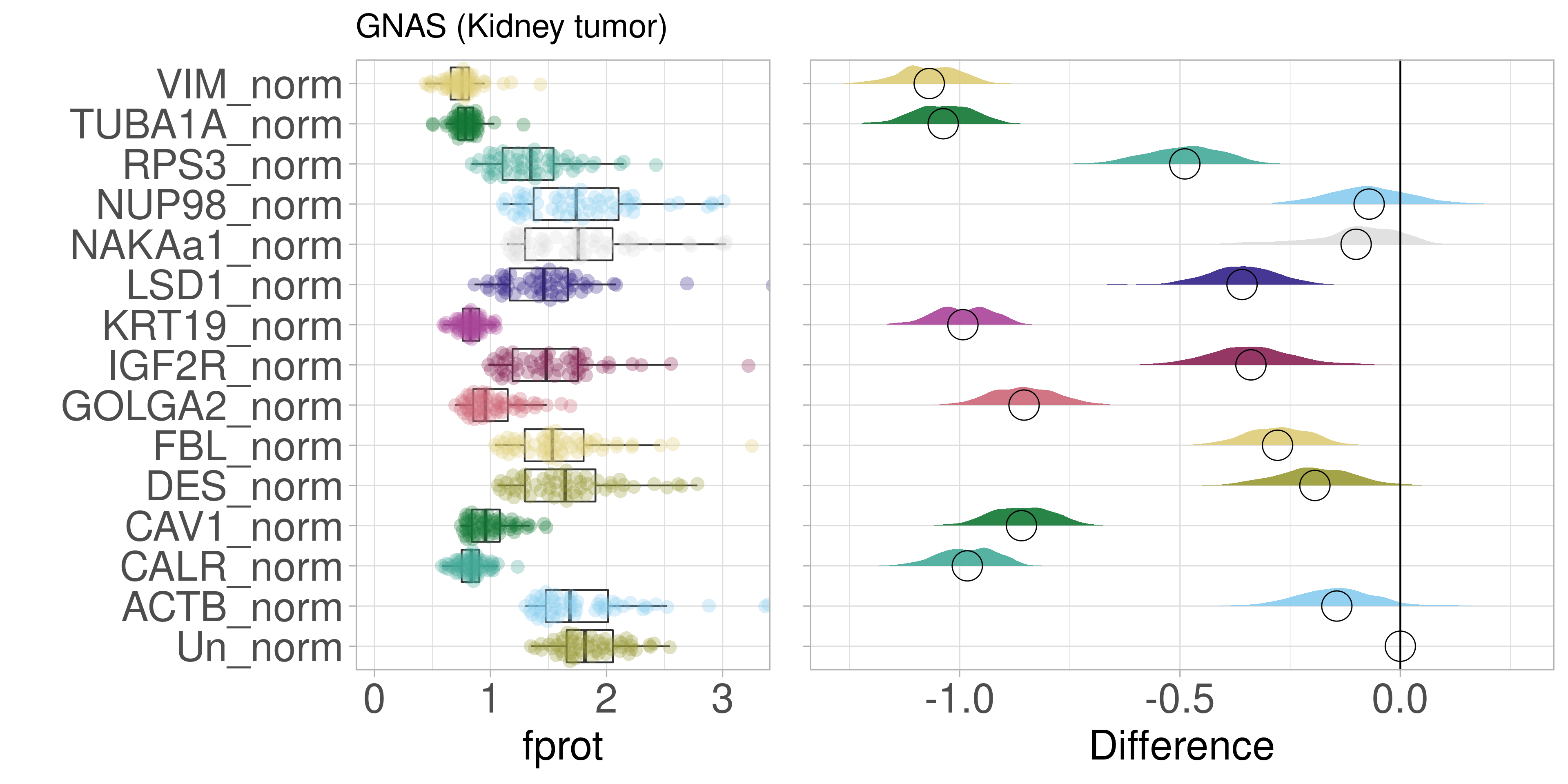

Supplement: Supplementary file 17 — Supplementary Material 17 [file 41598_2026_48754_MOESM17_ESM.zip › RPPA normalizations to cell markers/Kidney_plots/Tumor_suppr_Kidney/GNAS_Kidney_T.png]

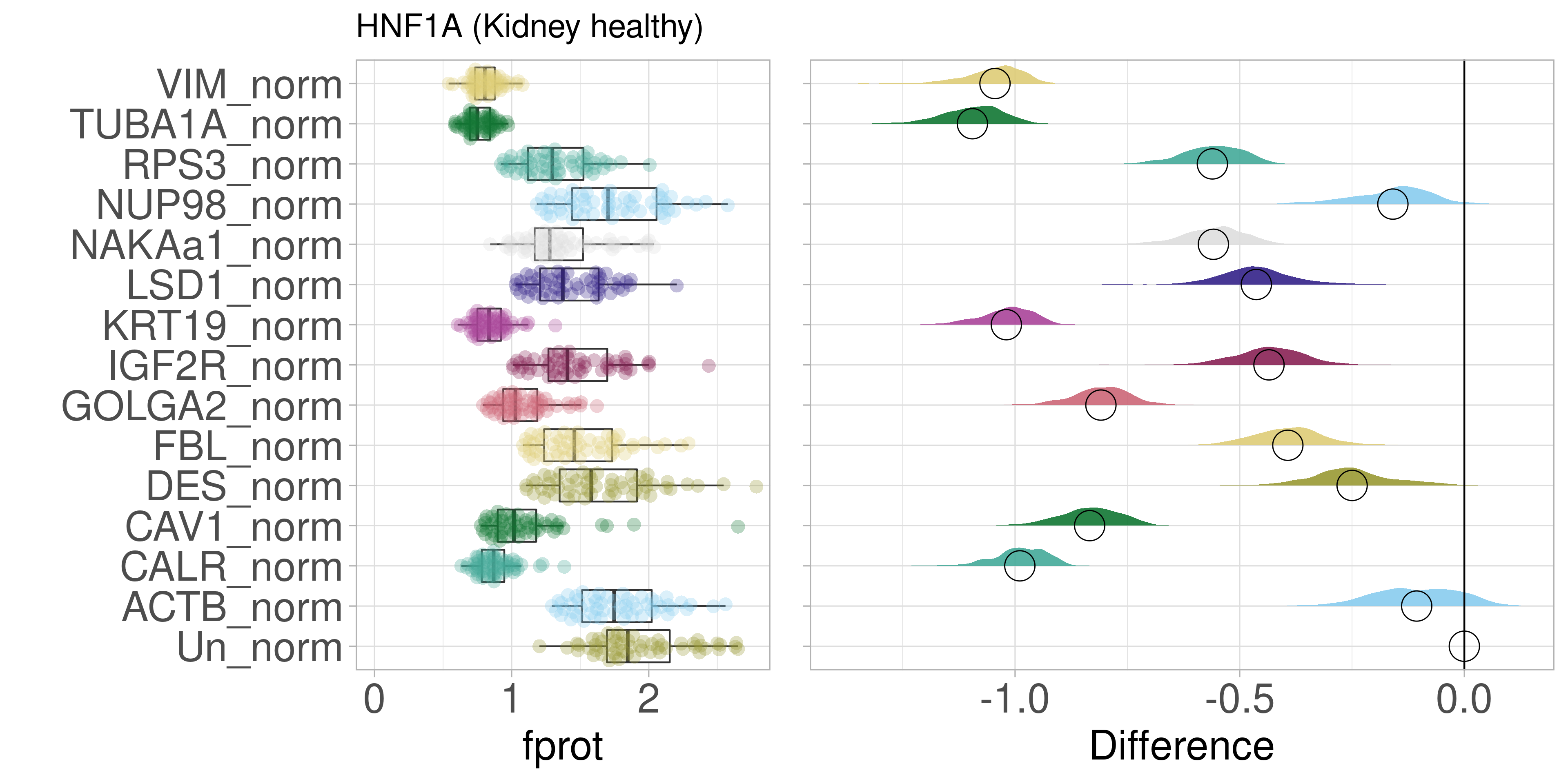

Supplement: Supplementary file 17 — Supplementary Material 17 [file 41598_2026_48754_MOESM17_ESM.zip › RPPA normalizations to cell markers/Kidney_plots/Tumor_suppr_Kidney/HNF1A_Kidney_H.png]

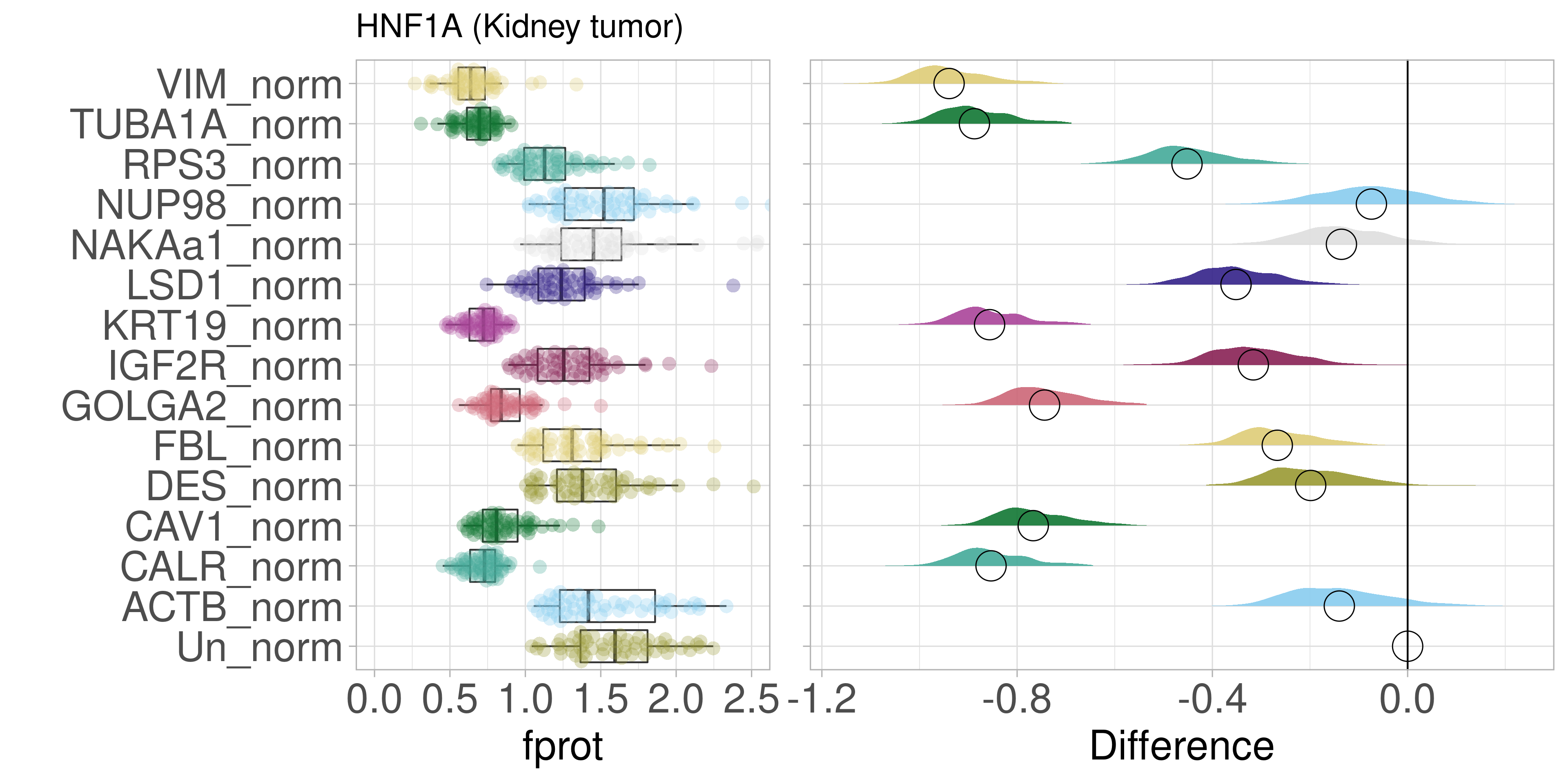

Supplement: Supplementary file 17 — Supplementary Material 17 [file 41598_2026_48754_MOESM17_ESM.zip › RPPA normalizations to cell markers/Kidney_plots/Tumor_suppr_Kidney/HNF1A_Kidney_T.png]

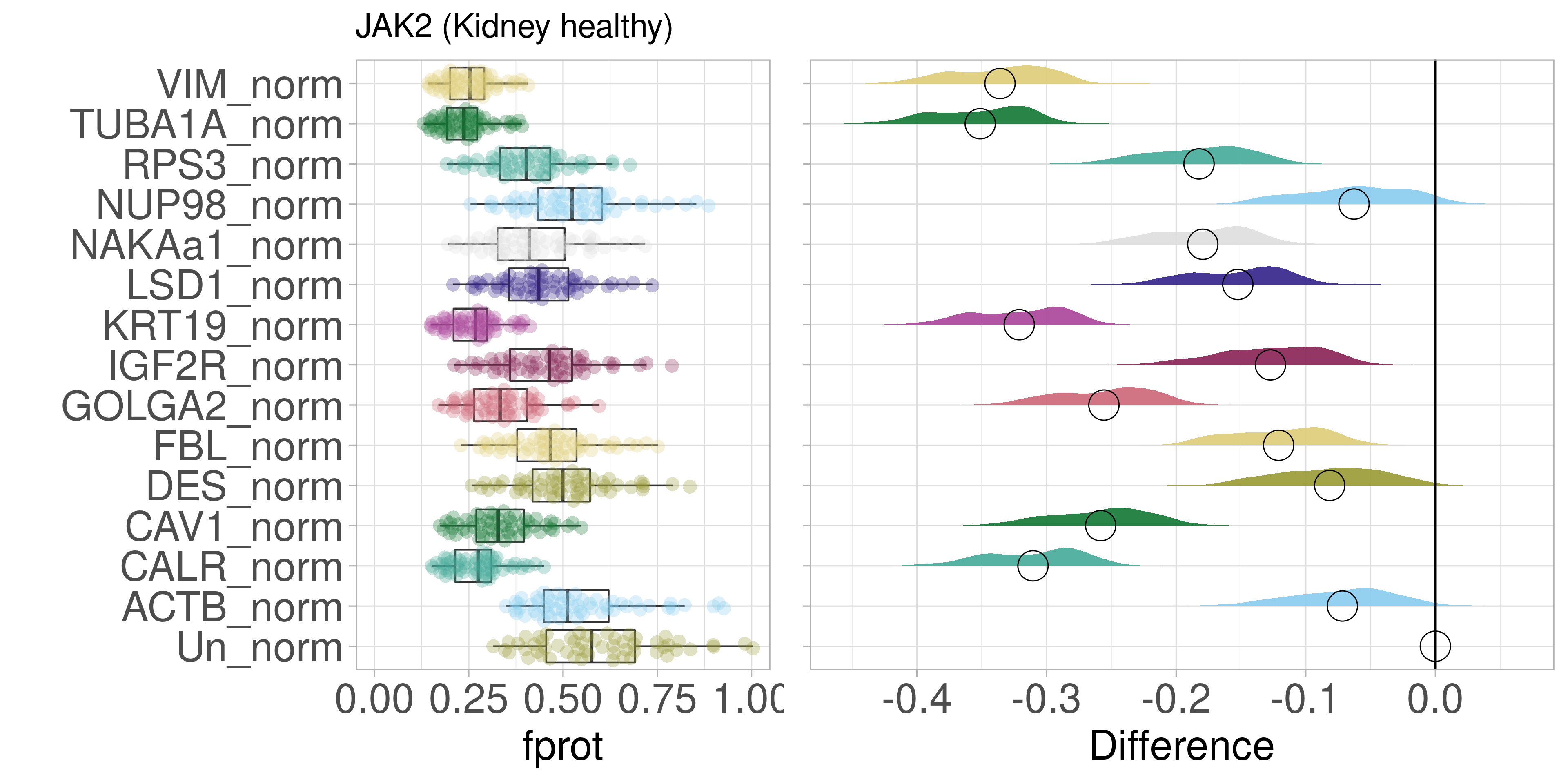

Supplement: Supplementary file 17 — Supplementary Material 17 [file 41598_2026_48754_MOESM17_ESM.zip › RPPA normalizations to cell markers/Kidney_plots/Tumor_suppr_Kidney/JAK2_Kidney_H.png]

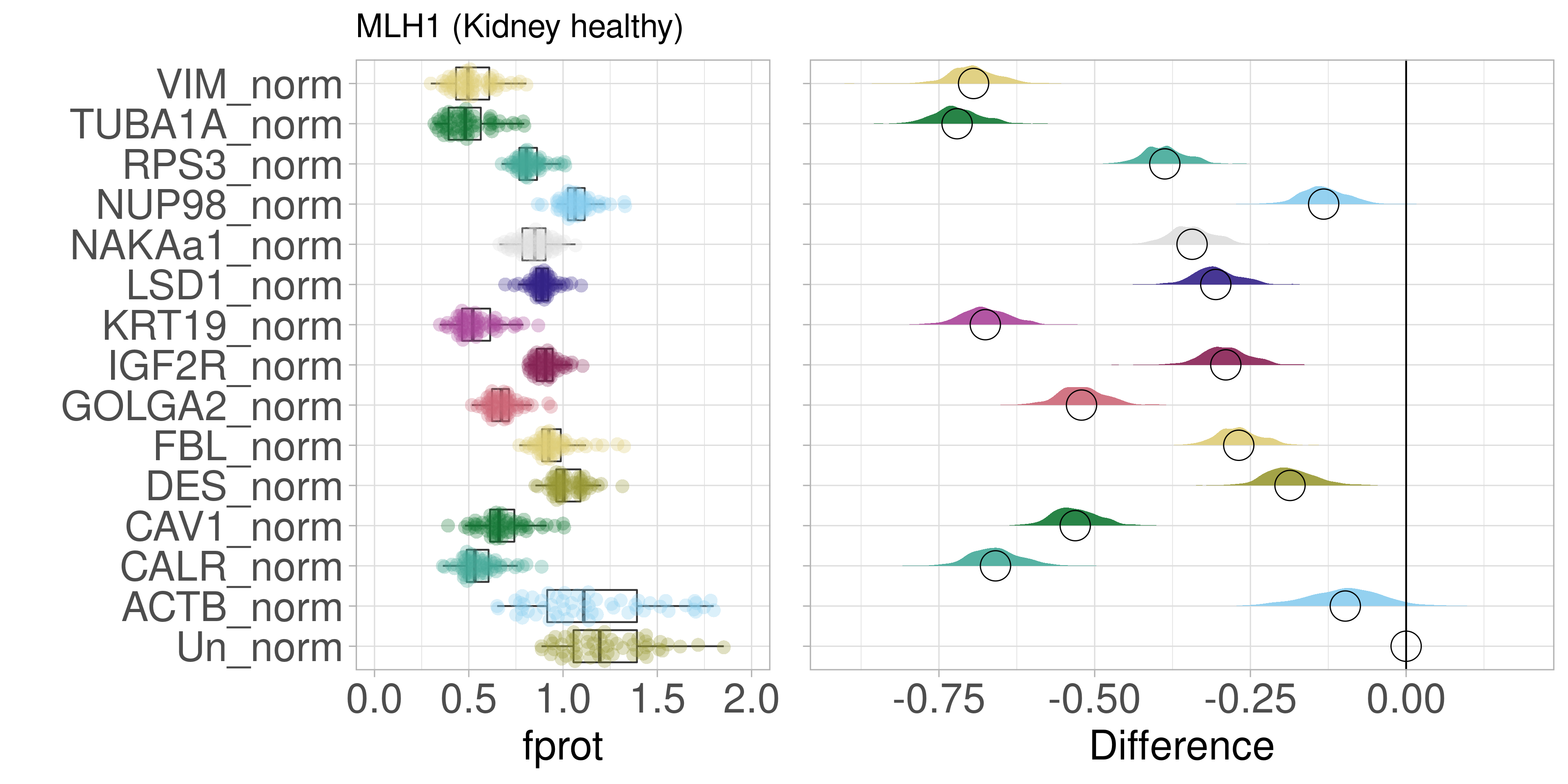

Supplement: Supplementary file 17 — Supplementary Material 17 [file 41598_2026_48754_MOESM17_ESM.zip › RPPA normalizations to cell markers/Kidney_plots/Tumor_suppr_Kidney/MLH1_Kidney_H.png]

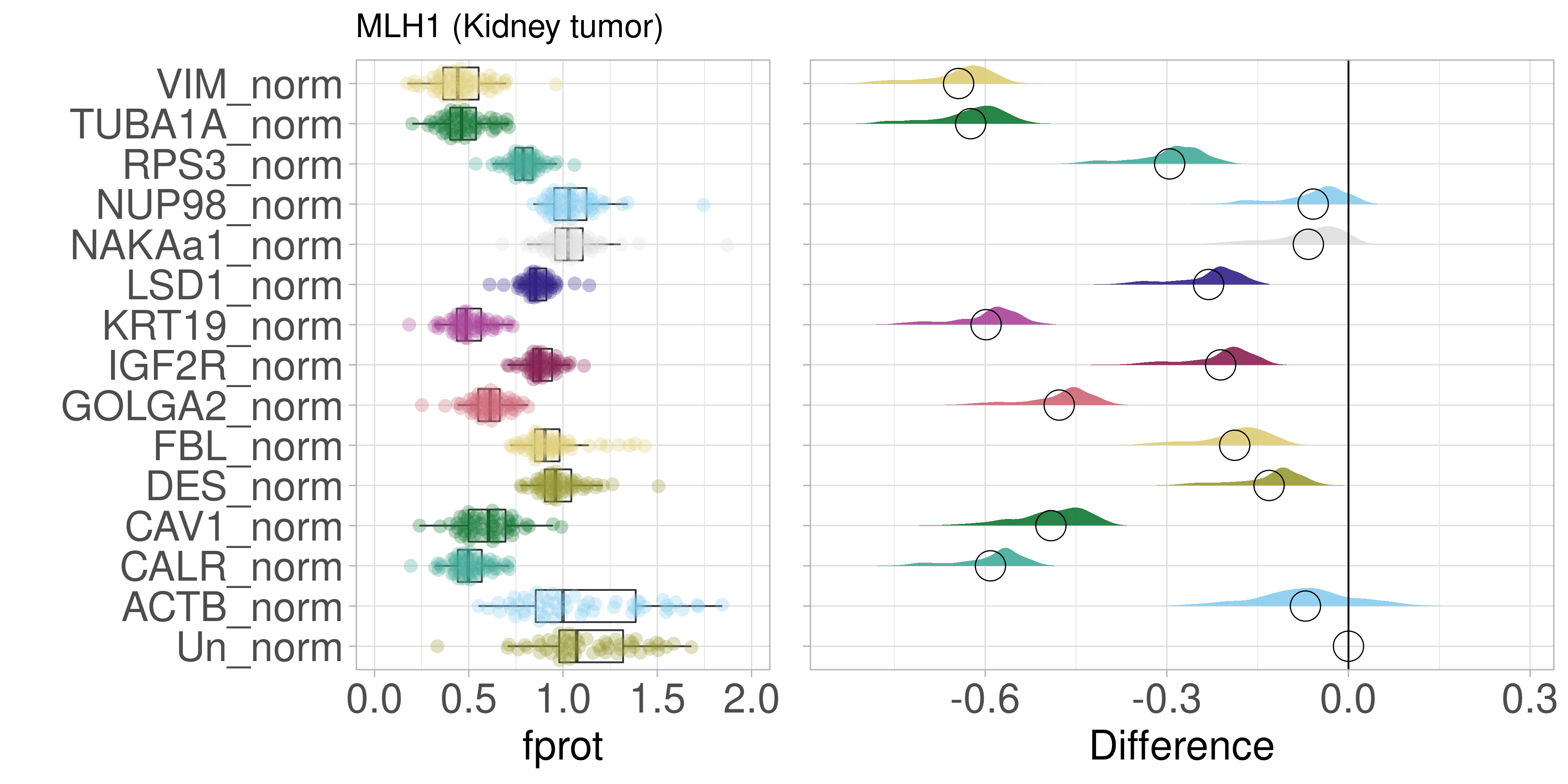

Supplement: Supplementary file 17 — Supplementary Material 17 [file 41598_2026_48754_MOESM17_ESM.zip › RPPA normalizations to cell markers/Kidney_plots/Tumor_suppr_Kidney/MLH1_Kidney_T.png]

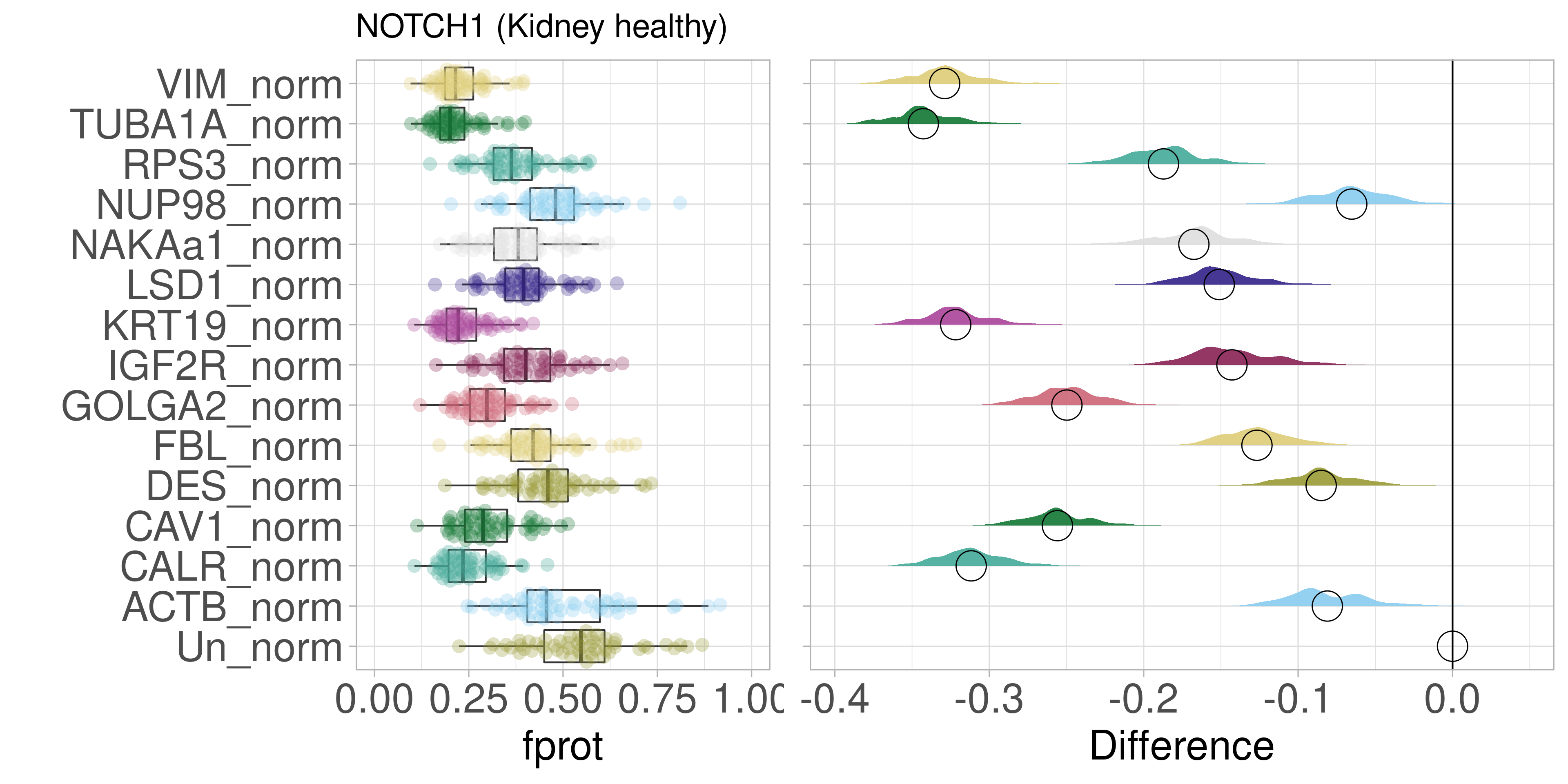

Supplement: Supplementary file 17 — Supplementary Material 17 [file 41598_2026_48754_MOESM17_ESM.zip › RPPA normalizations to cell markers/Kidney_plots/Tumor_suppr_Kidney/NOTCH1_Kidney_H.png]

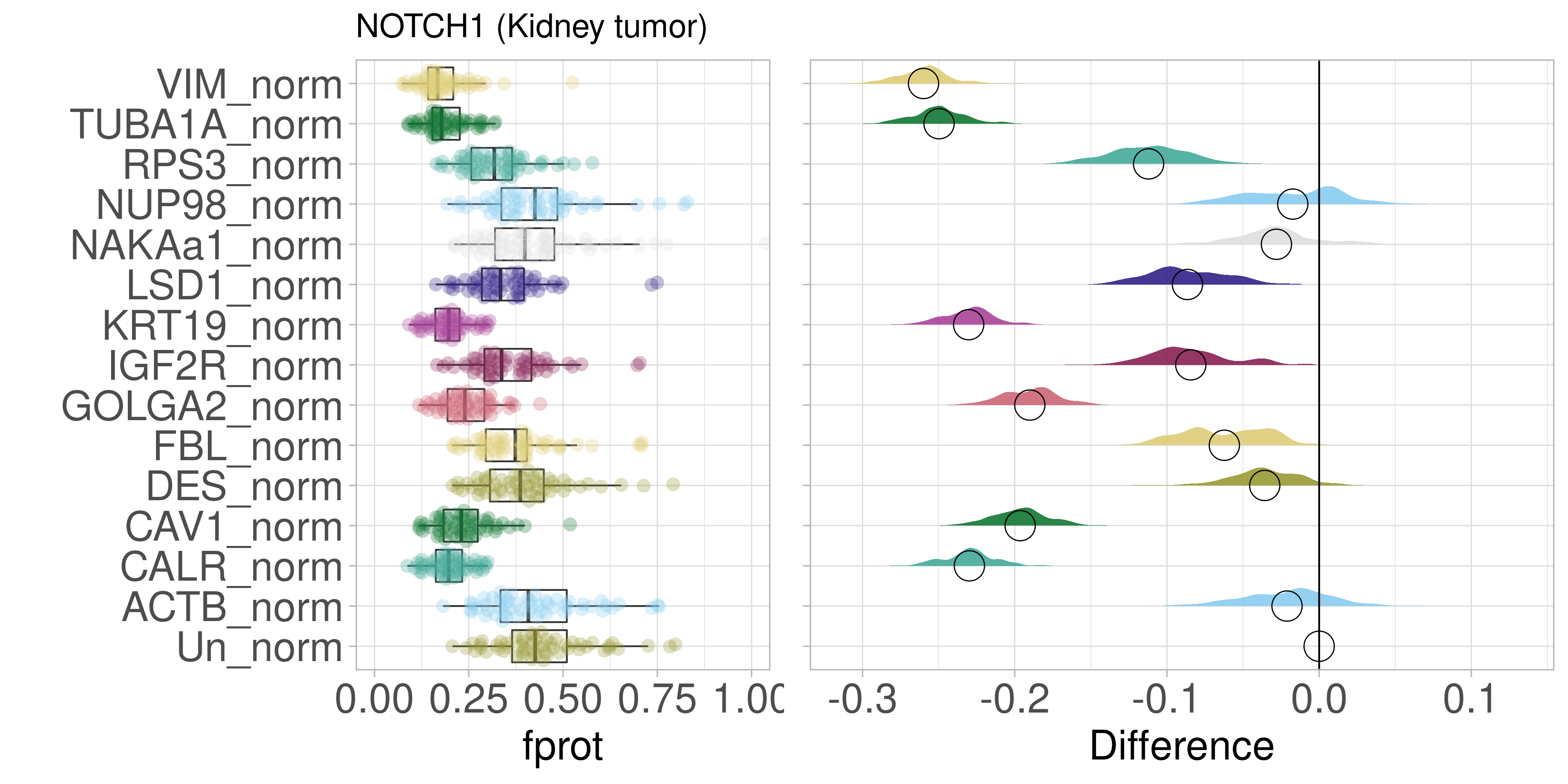

Supplement: Supplementary file 17 — Supplementary Material 17 [file 41598_2026_48754_MOESM17_ESM.zip › RPPA normalizations to cell markers/Kidney_plots/Tumor_suppr_Kidney/NOTCH1_Kidney_T.png]

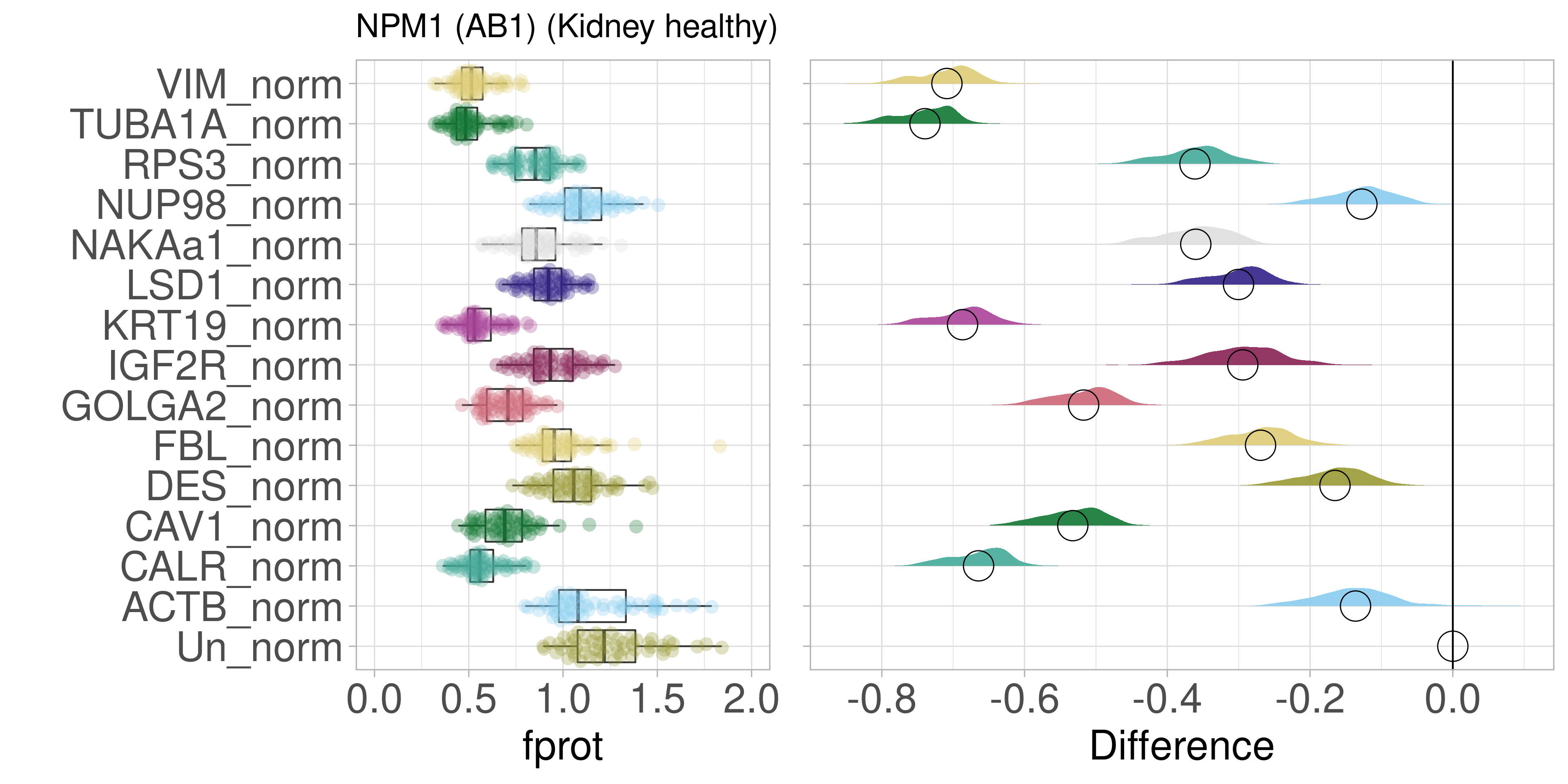

Supplement: Supplementary file 17 — Supplementary Material 17 [file 41598_2026_48754_MOESM17_ESM.zip › RPPA normalizations to cell markers/Kidney_plots/Tumor_suppr_Kidney/NPM1(AB1)_Kidney_H.png]

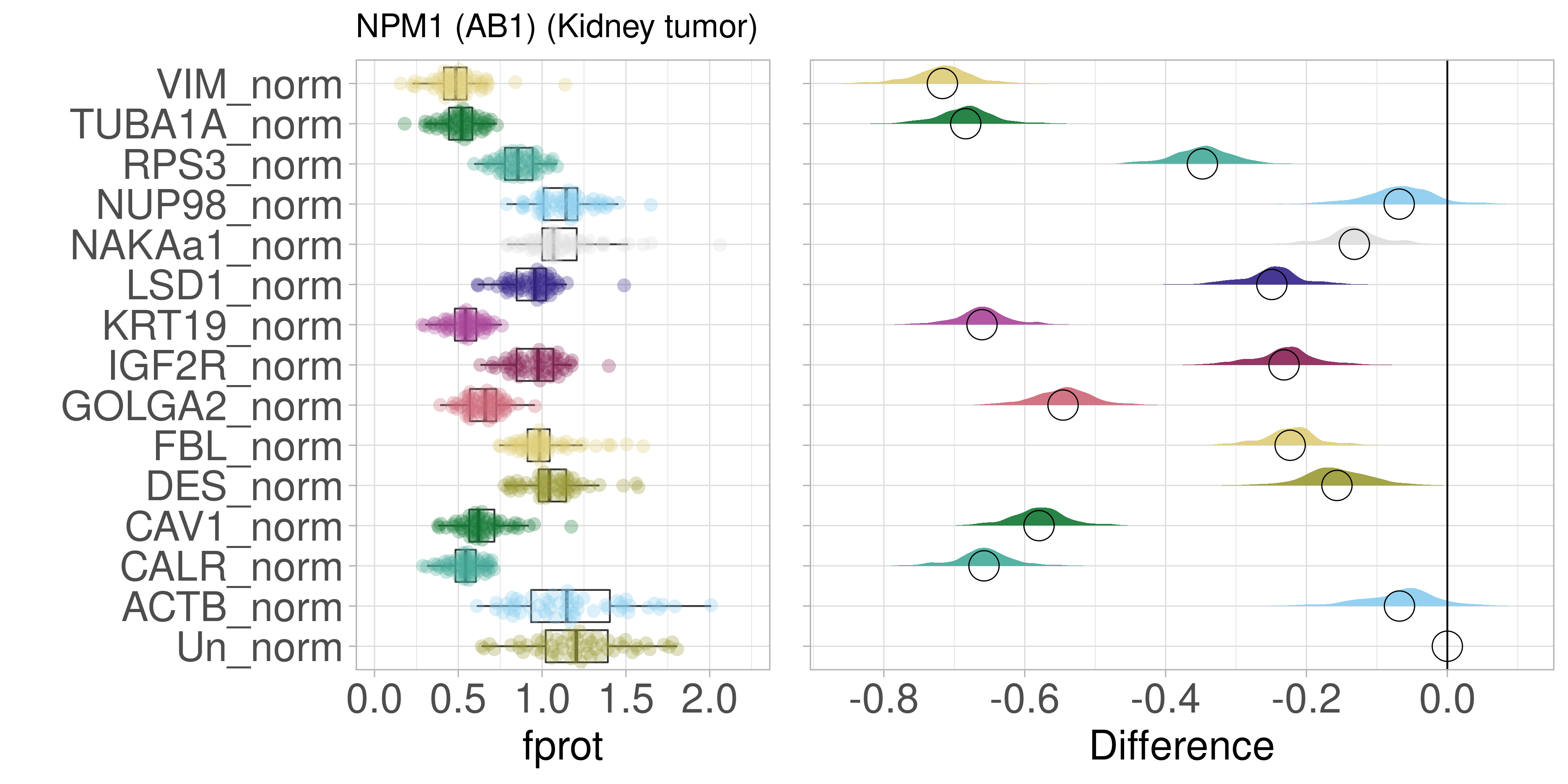

Supplement: Supplementary file 17 — Supplementary Material 17 [file 41598_2026_48754_MOESM17_ESM.zip › RPPA normalizations to cell markers/Kidney_plots/Tumor_suppr_Kidney/NPM1(AB1)_Kidney_T.png]

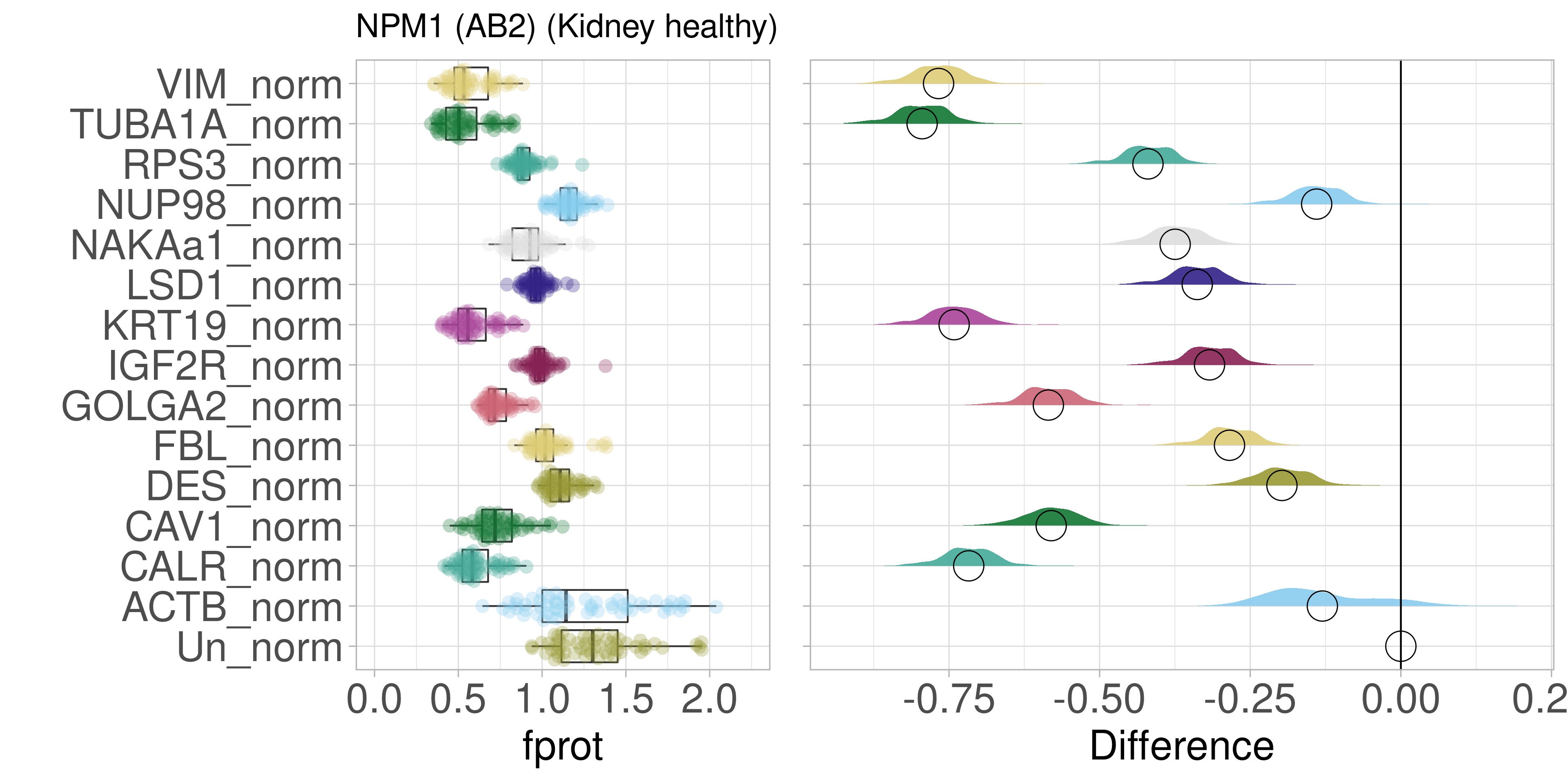

Supplement: Supplementary file 17 — Supplementary Material 17 [file 41598_2026_48754_MOESM17_ESM.zip › RPPA normalizations to cell markers/Kidney_plots/Tumor_suppr_Kidney/NPM1(AB2)_Kidney_H.png]

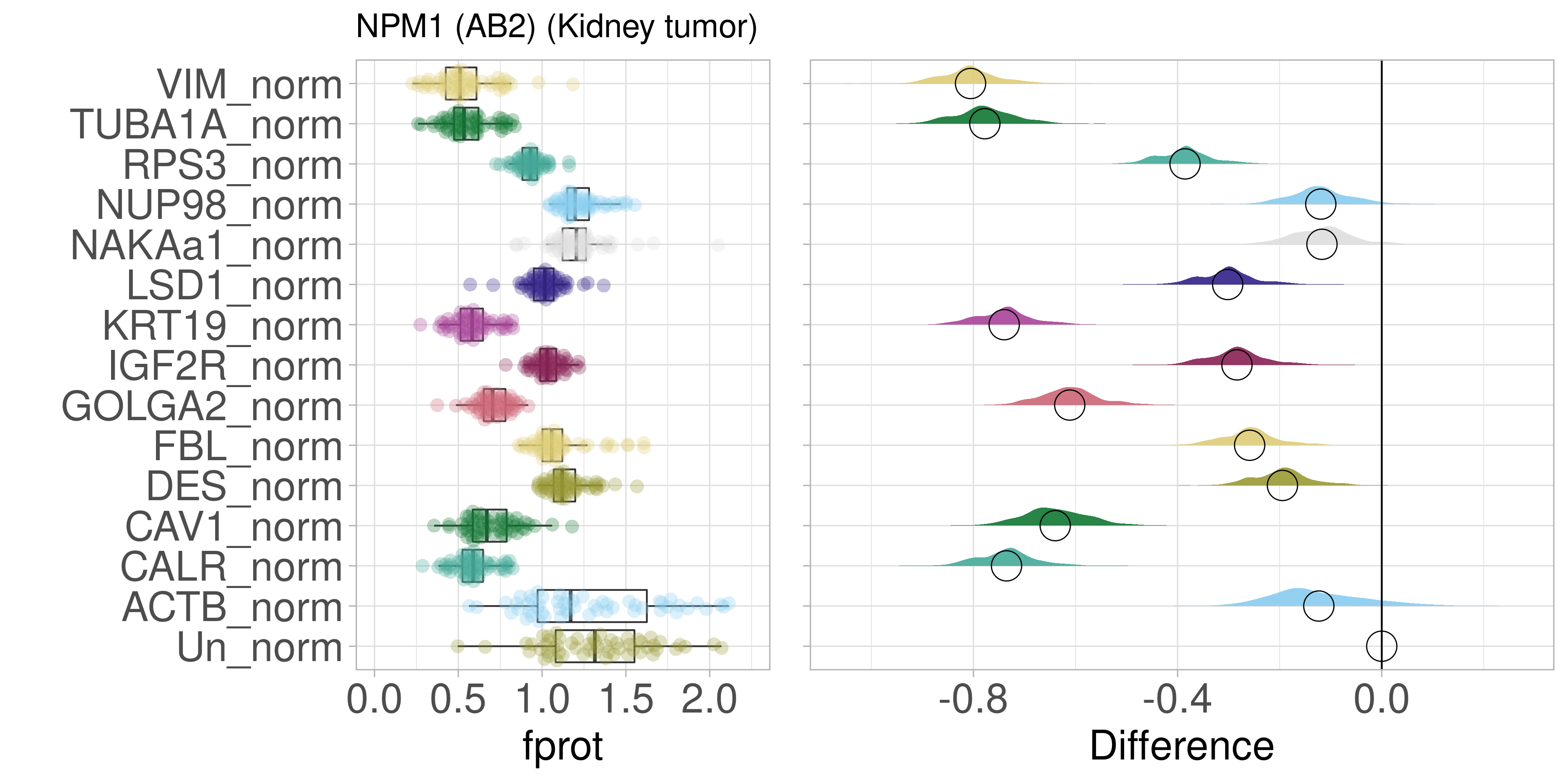

Supplement: Supplementary file 17 — Supplementary Material 17 [file 41598_2026_48754_MOESM17_ESM.zip › RPPA normalizations to cell markers/Kidney_plots/Tumor_suppr_Kidney/NPM1(AB2)_Kidney_T.png]

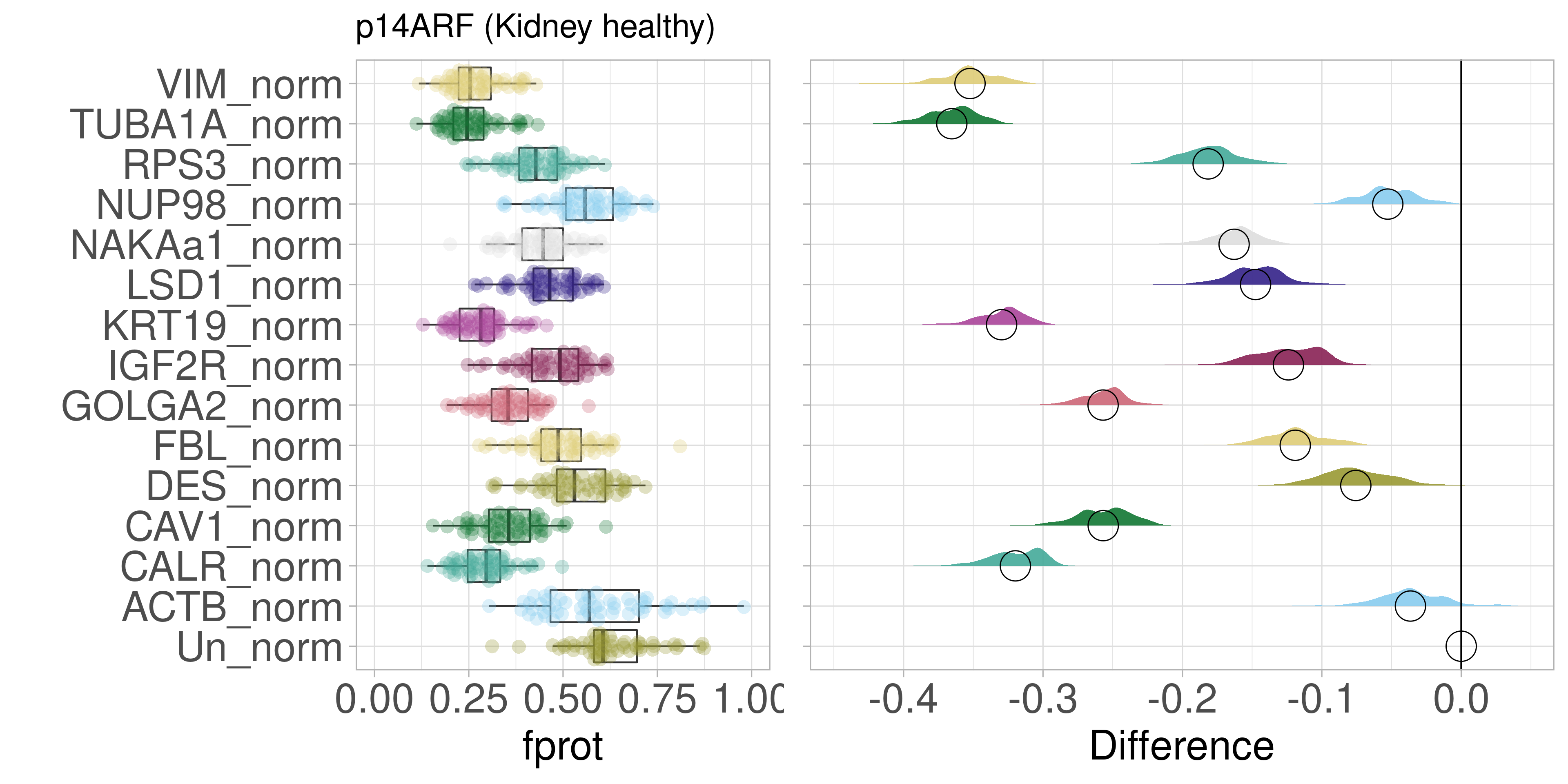

Supplement: Supplementary file 17 — Supplementary Material 17 [file 41598_2026_48754_MOESM17_ESM.zip › RPPA normalizations to cell markers/Kidney_plots/Tumor_suppr_Kidney/p14ARF_Kidney_H.png]

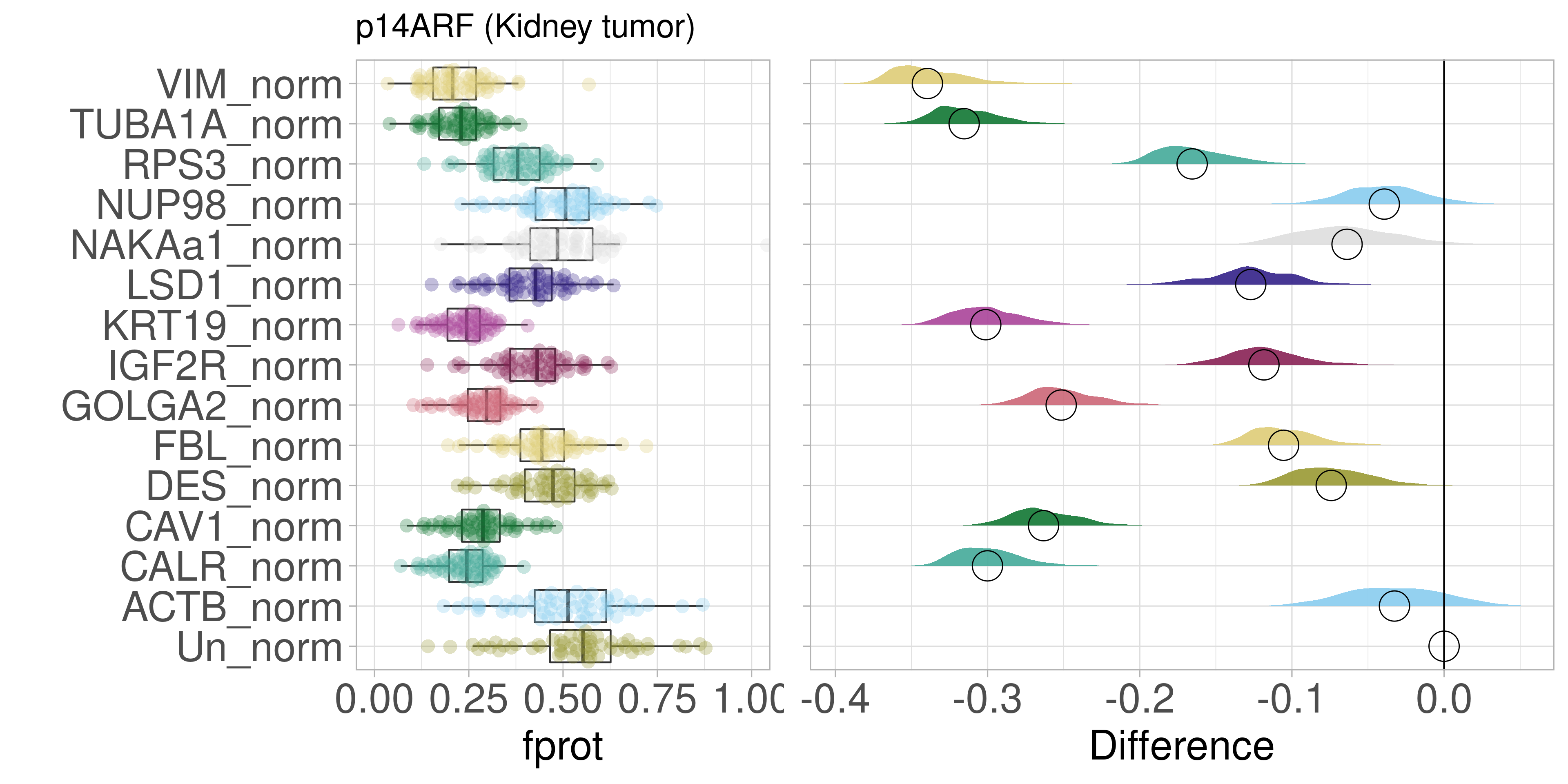

Supplement: Supplementary file 17 — Supplementary Material 17 [file 41598_2026_48754_MOESM17_ESM.zip › RPPA normalizations to cell markers/Kidney_plots/Tumor_suppr_Kidney/p14ARF_Kidney_T.png]

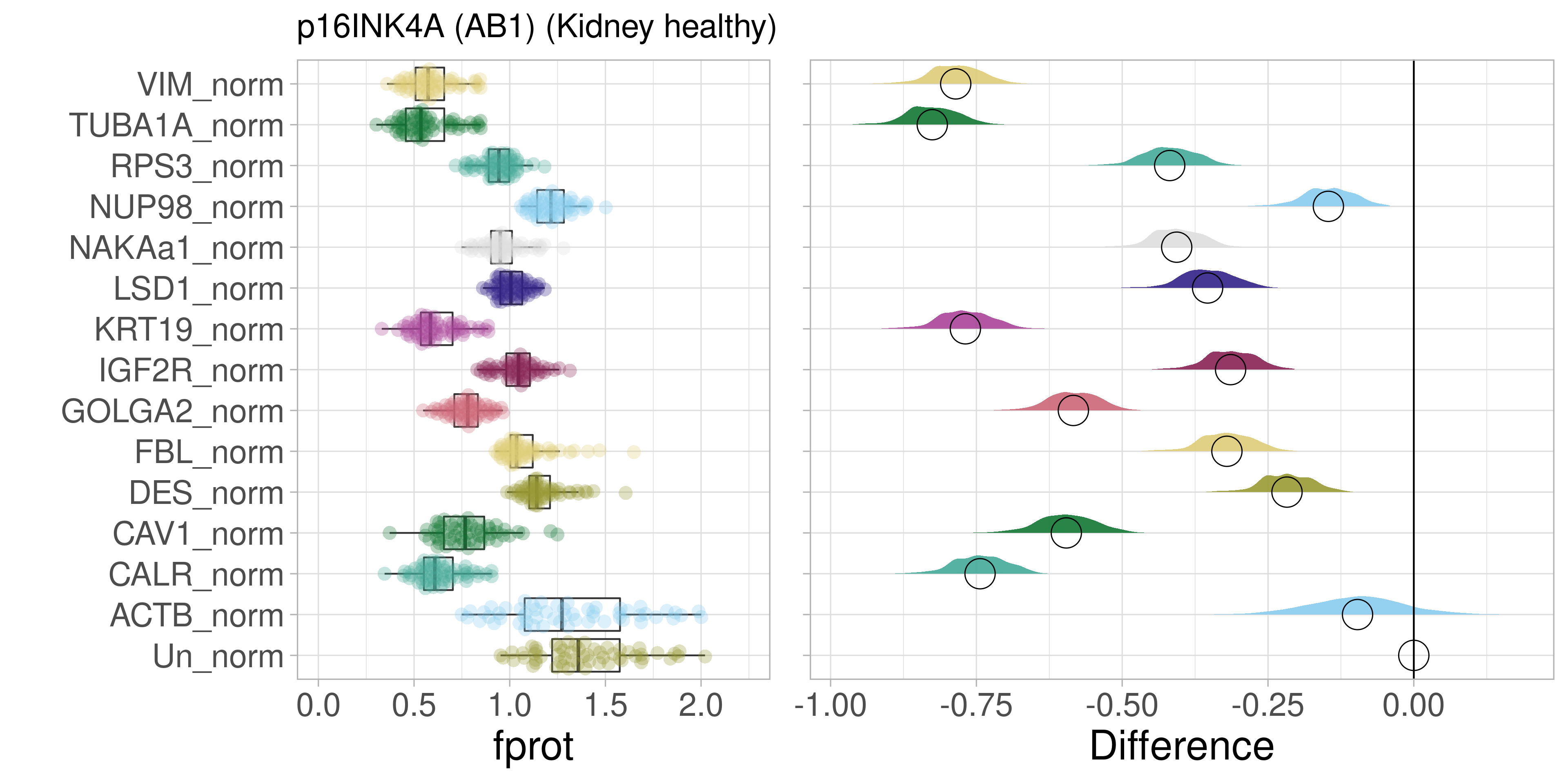

Supplement: Supplementary file 17 — Supplementary Material 17 [file 41598_2026_48754_MOESM17_ESM.zip › RPPA normalizations to cell markers/Kidney_plots/Tumor_suppr_Kidney/p16INK4A(AB1)_Kidney_H.png]

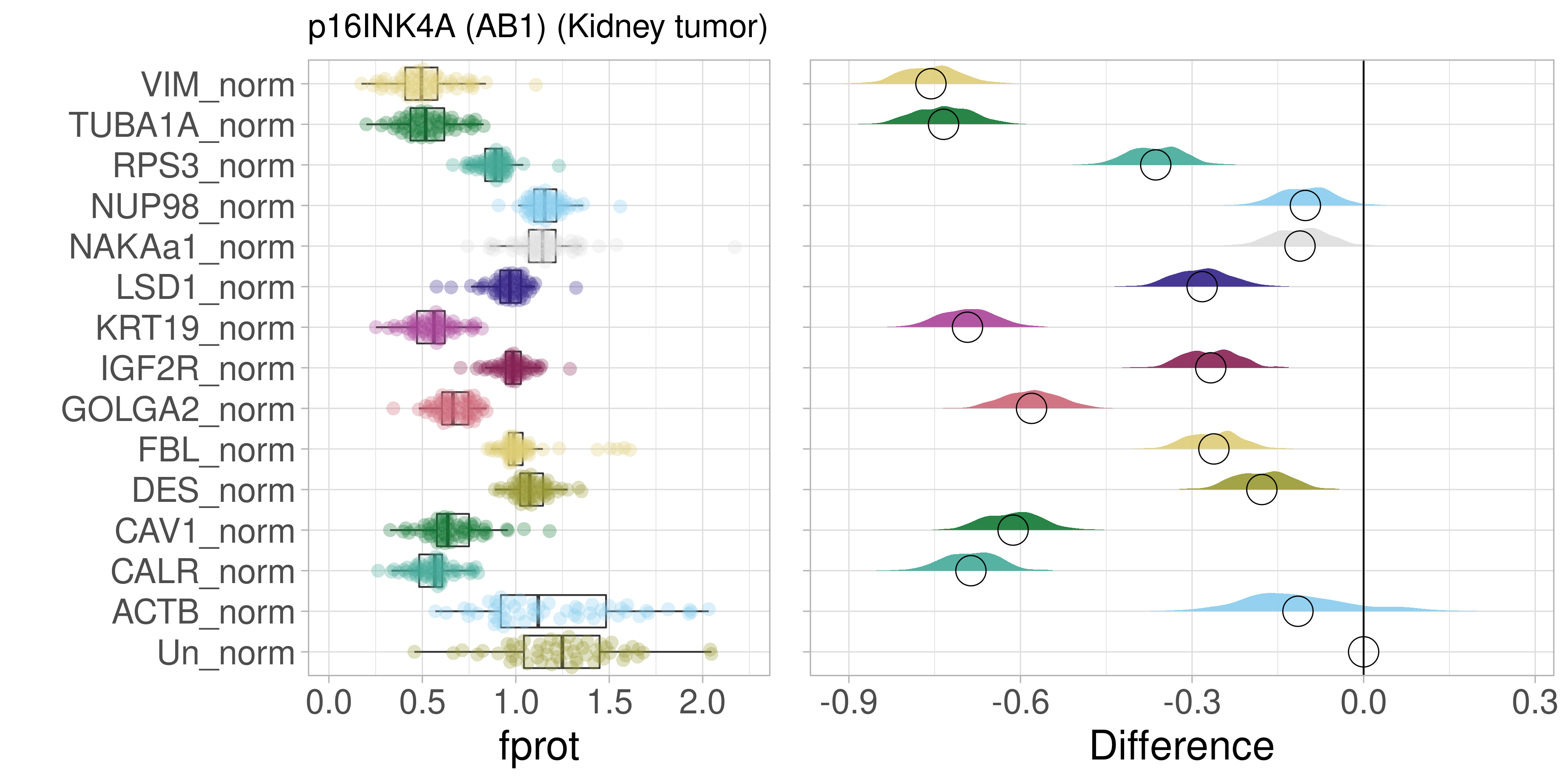

Supplement: Supplementary file 17 — Supplementary Material 17 [file 41598_2026_48754_MOESM17_ESM.zip › RPPA normalizations to cell markers/Kidney_plots/Tumor_suppr_Kidney/p16INK4A(AB1)_Kidney_T.png]

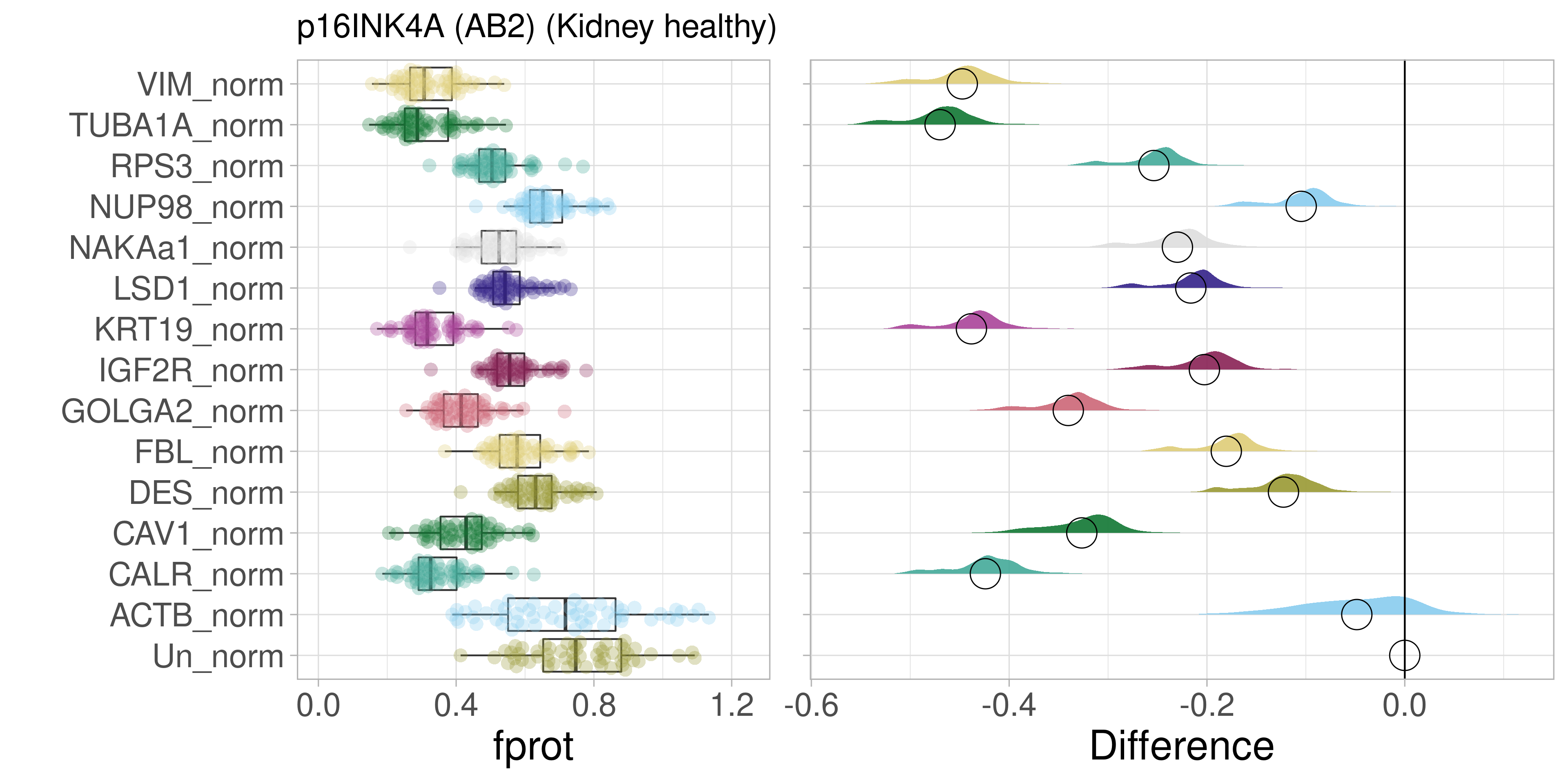

Supplement: Supplementary file 17 — Supplementary Material 17 [file 41598_2026_48754_MOESM17_ESM.zip › RPPA normalizations to cell markers/Kidney_plots/Tumor_suppr_Kidney/p16INK4A(AB2)_Kidney_H.png]

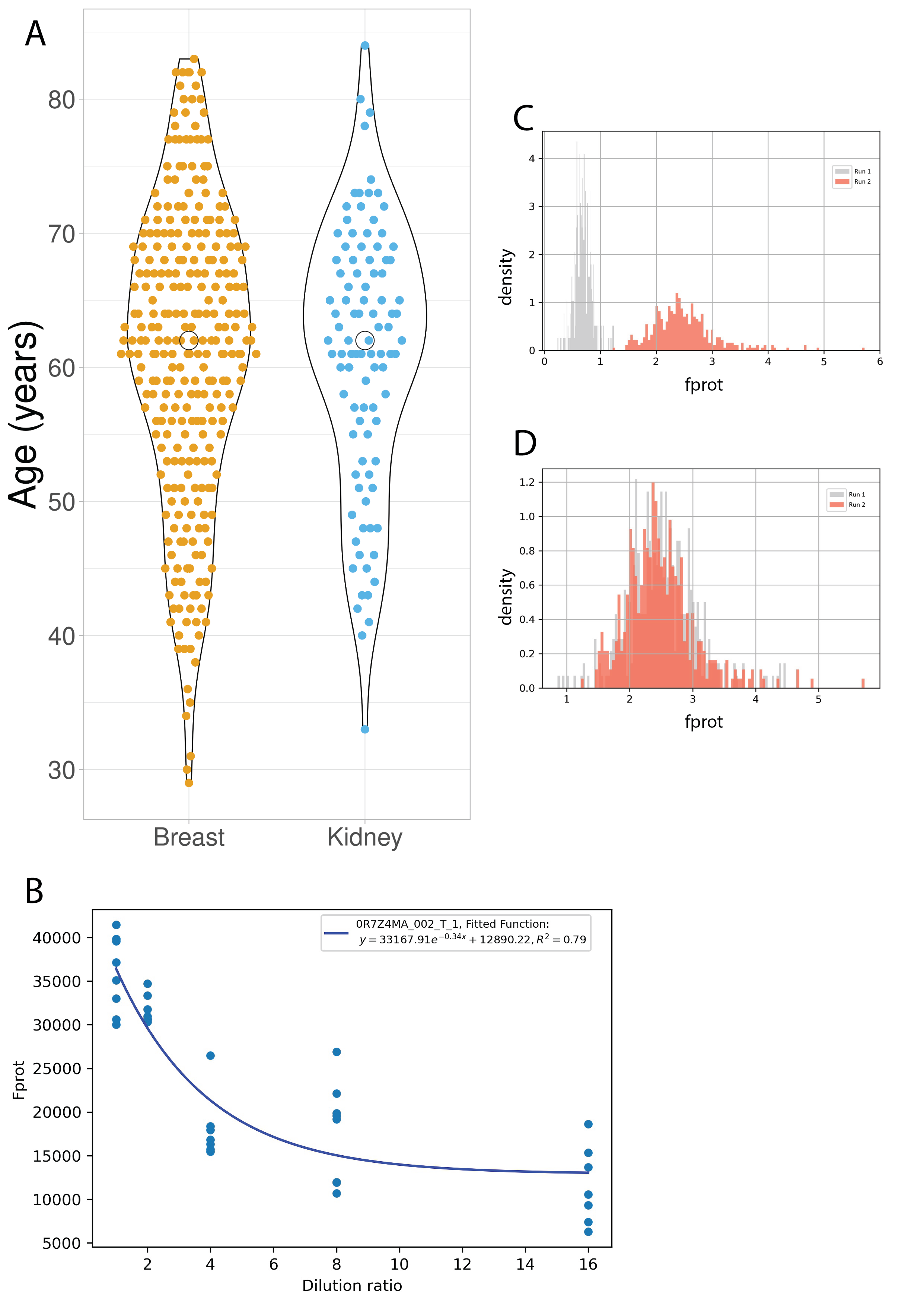

Supplement: Supplementary file 20 — Supplementary Material 20 [file 41598_2026_48754_MOESM20_ESM.png]

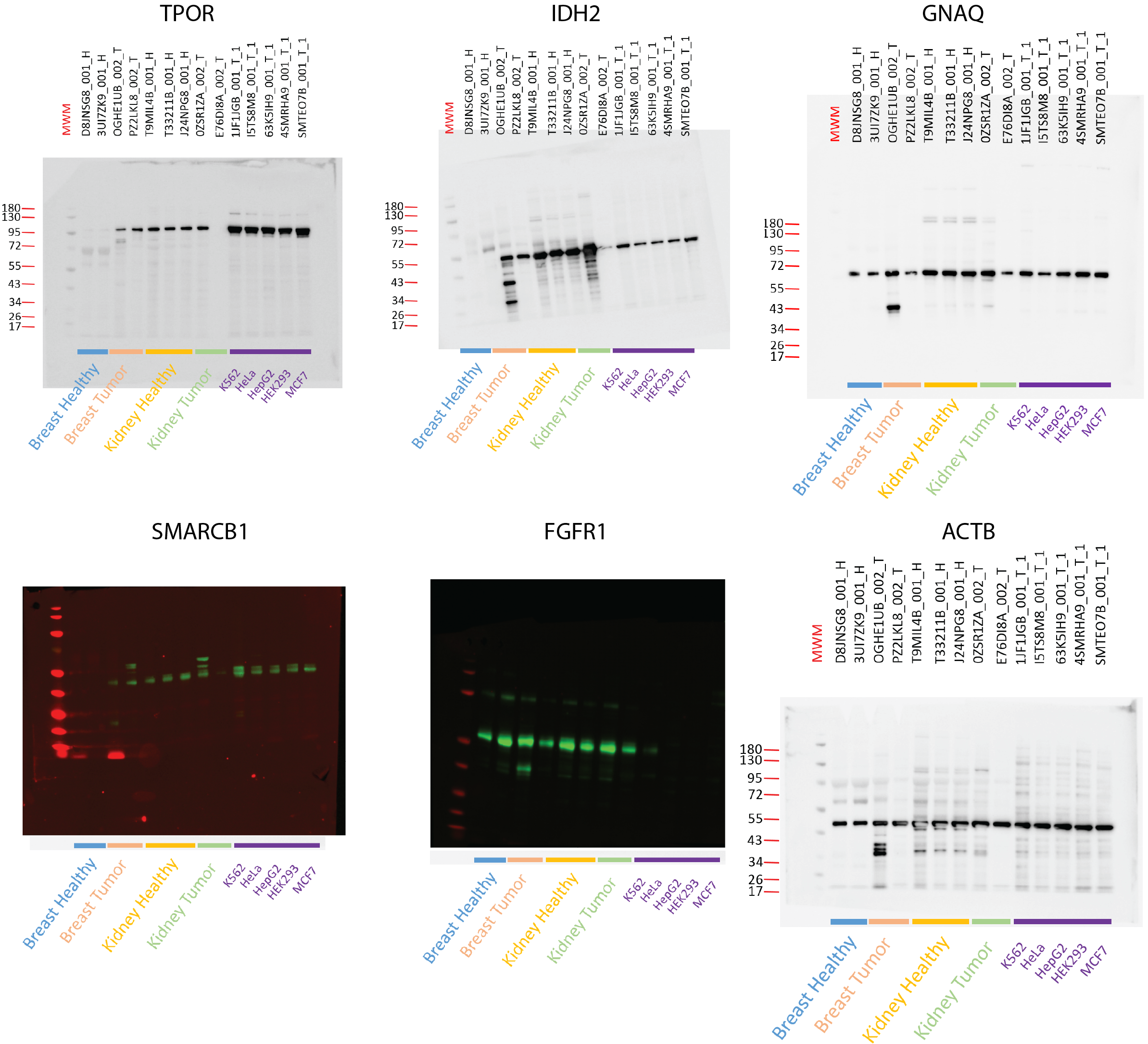

Supplement: Supplementary file 23 — Supplementary Material 23 [file 41598_2026_48754_MOESM23_ESM.png]
