# Supplementary figures and images for: Comparative analysis and correlation of cancer hotspot proteins and cell markers in tumor-normal adjacent breast and kidney samples using RPPA and LC-MS (part 2 of 3)
Source: Sci Rep. 2026 May 18;16:22442. doi: 10.1038/s41598-026-48754-2 (PMC13377106; doi:10.1038/s41598-026-48754-2)

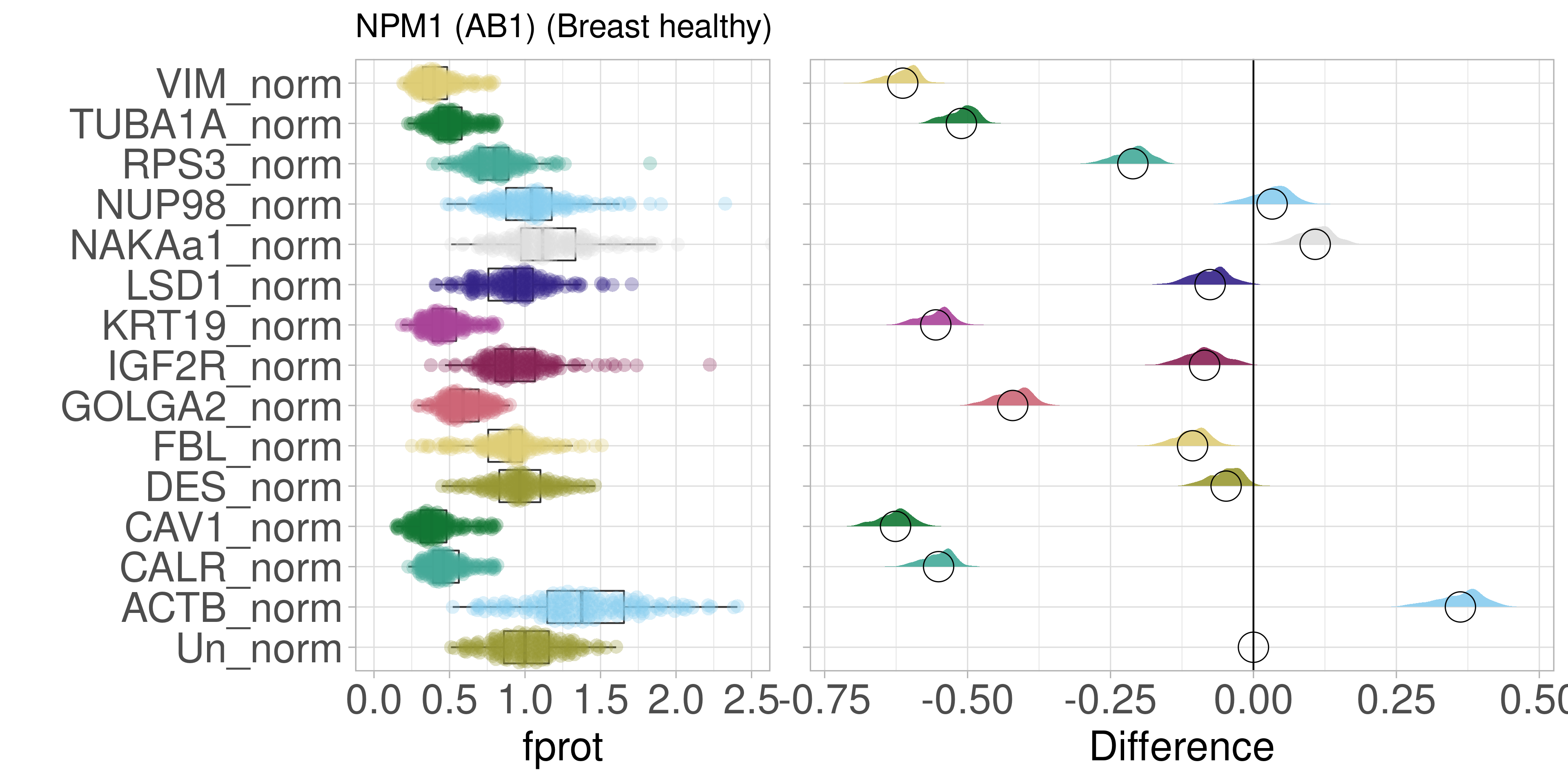

Supplement: Supplementary file 17 — Supplementary Material 17 [file 41598_2026_48754_MOESM17_ESM.zip › RPPA normalizations to cell markers/Breast_Plots/Tumor_suppr_Breast/NPM1(AB1)_Breast_H.png]

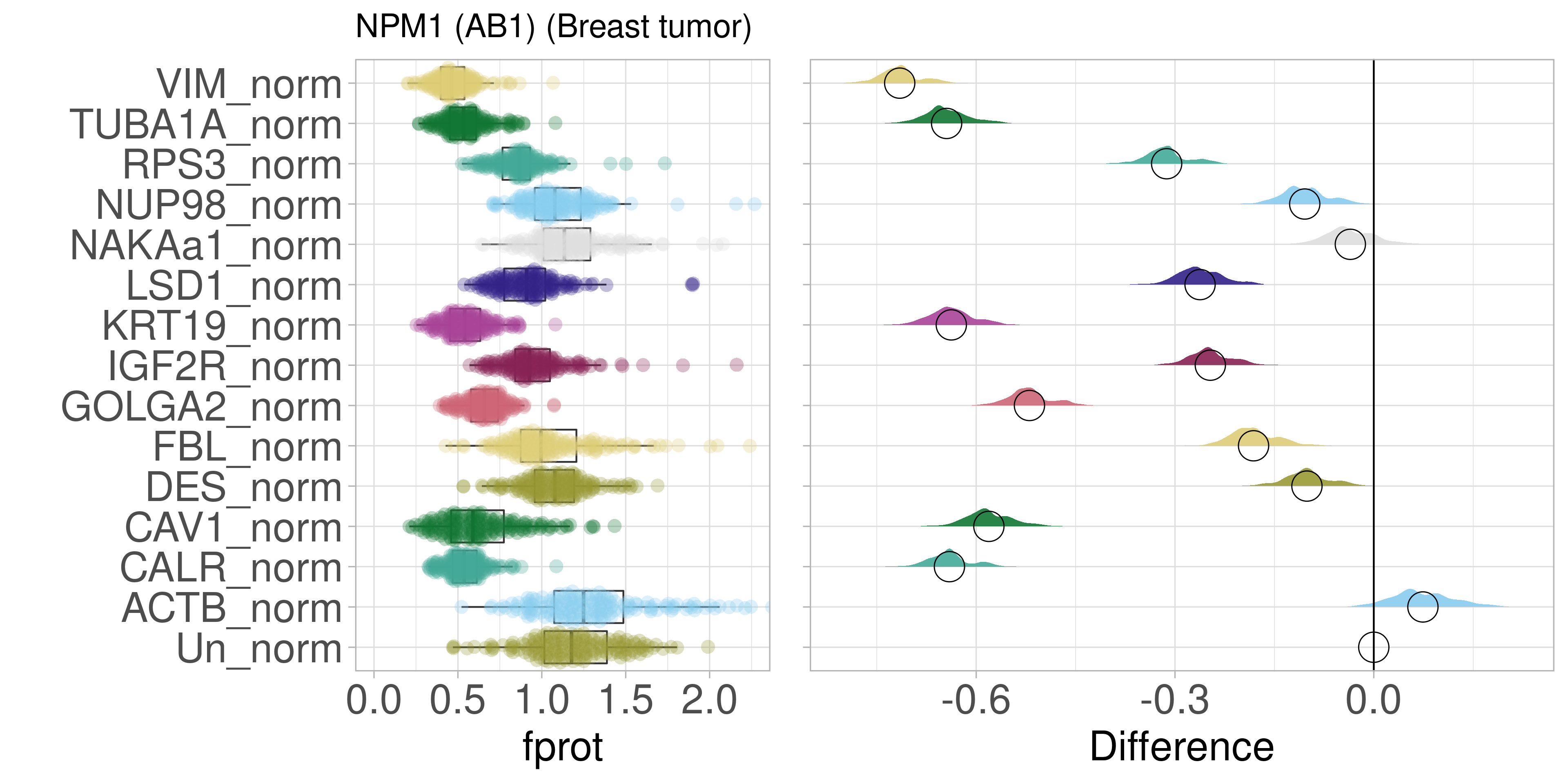

Supplement: Supplementary file 17 — Supplementary Material 17 [file 41598_2026_48754_MOESM17_ESM.zip › RPPA normalizations to cell markers/Breast_Plots/Tumor_suppr_Breast/NPM1(AB1)_Breast_T.png]

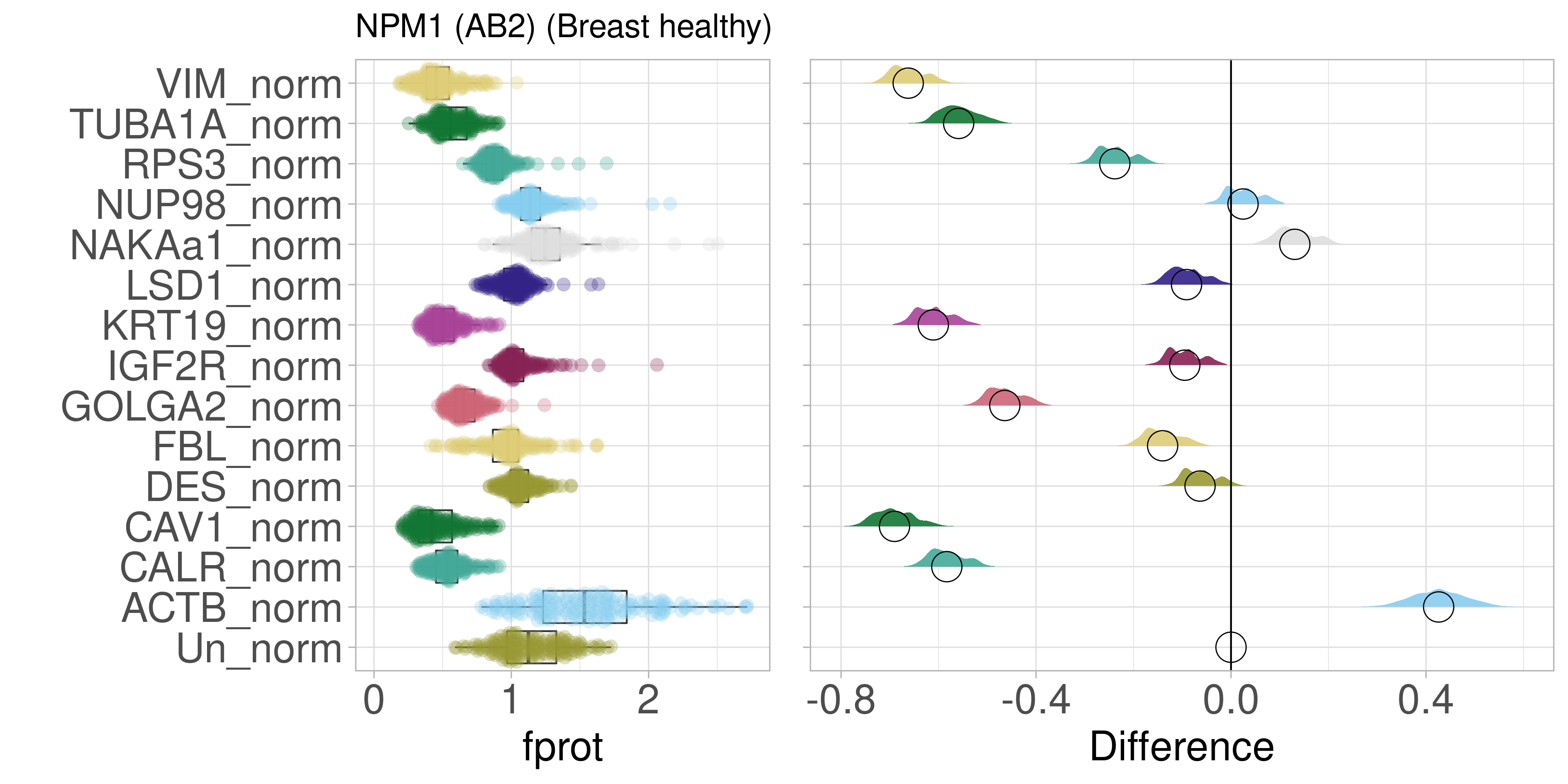

Supplement: Supplementary file 17 — Supplementary Material 17 [file 41598_2026_48754_MOESM17_ESM.zip › RPPA normalizations to cell markers/Breast_Plots/Tumor_suppr_Breast/NPM1(AB2)_Breast_H.png]

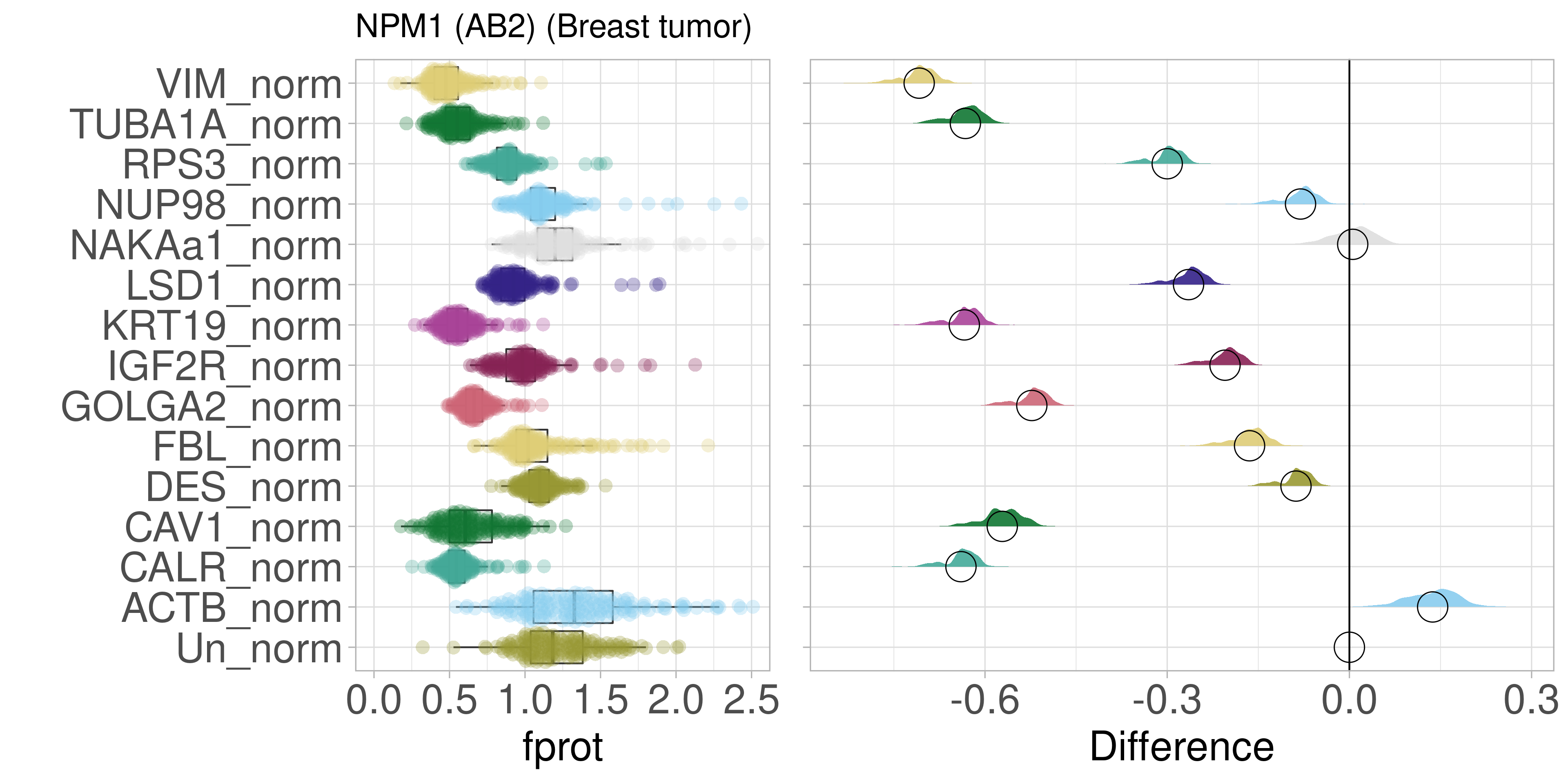

Supplement: Supplementary file 17 — Supplementary Material 17 [file 41598_2026_48754_MOESM17_ESM.zip › RPPA normalizations to cell markers/Breast_Plots/Tumor_suppr_Breast/NPM1(AB2)_Breast_T.png]

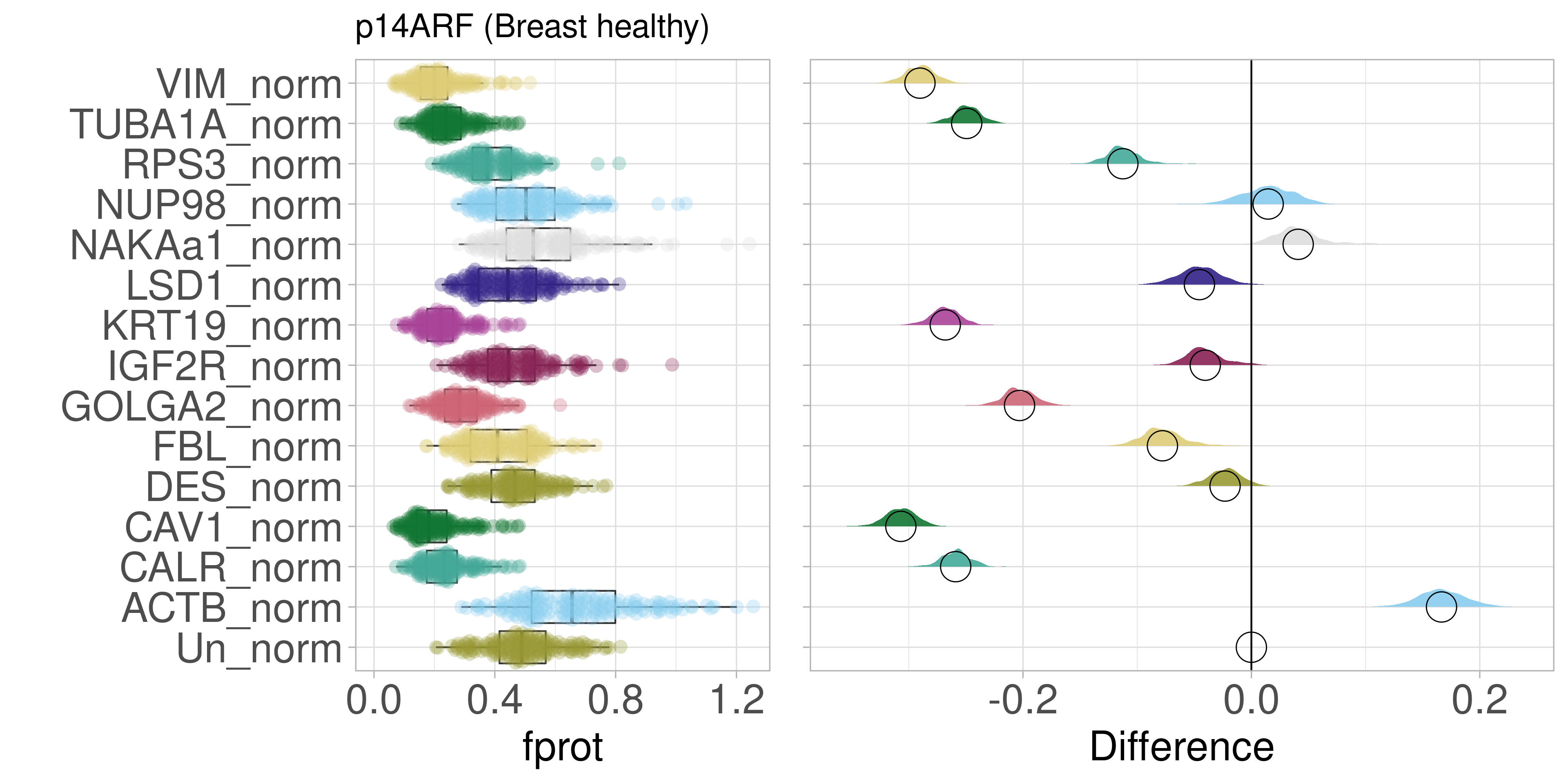

Supplement: Supplementary file 17 — Supplementary Material 17 [file 41598_2026_48754_MOESM17_ESM.zip › RPPA normalizations to cell markers/Breast_Plots/Tumor_suppr_Breast/p14ARF_Breast_H.png]

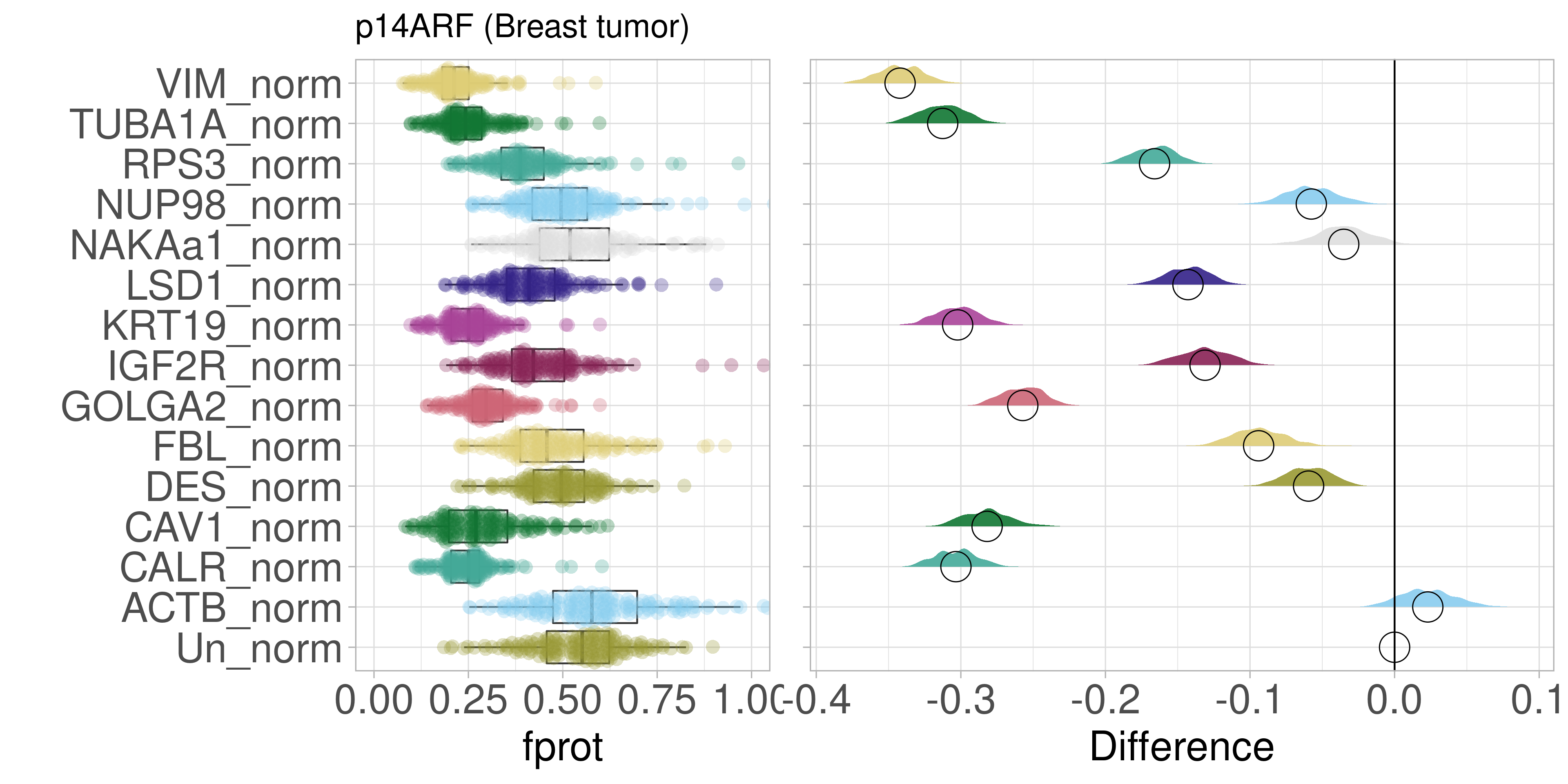

Supplement: Supplementary file 17 — Supplementary Material 17 [file 41598_2026_48754_MOESM17_ESM.zip › RPPA normalizations to cell markers/Breast_Plots/Tumor_suppr_Breast/p14ARF_Breast_T.png]

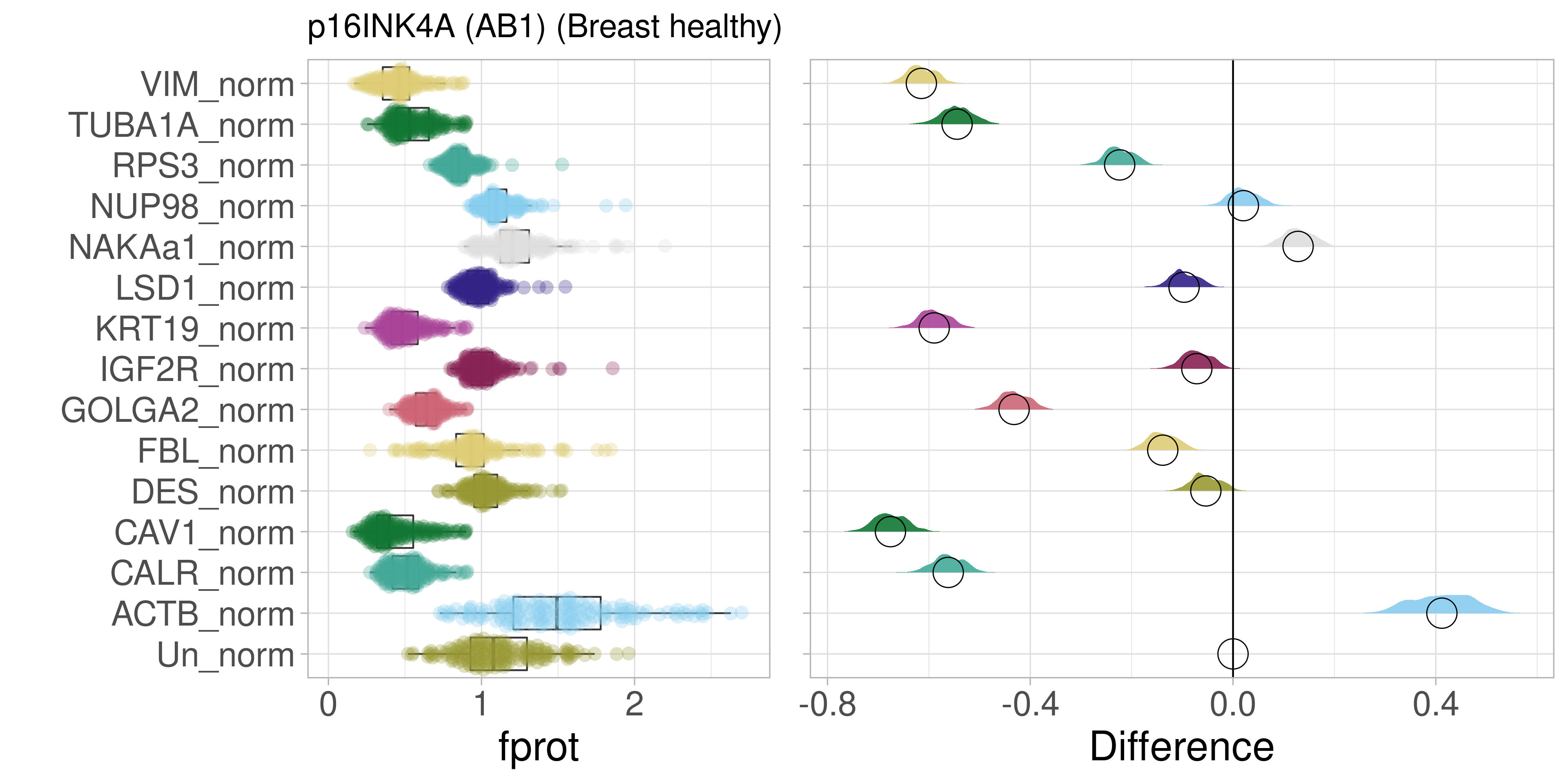

Supplement: Supplementary file 17 — Supplementary Material 17 [file 41598_2026_48754_MOESM17_ESM.zip › RPPA normalizations to cell markers/Breast_Plots/Tumor_suppr_Breast/p16INK4A(AB1)_Breast_H.png]

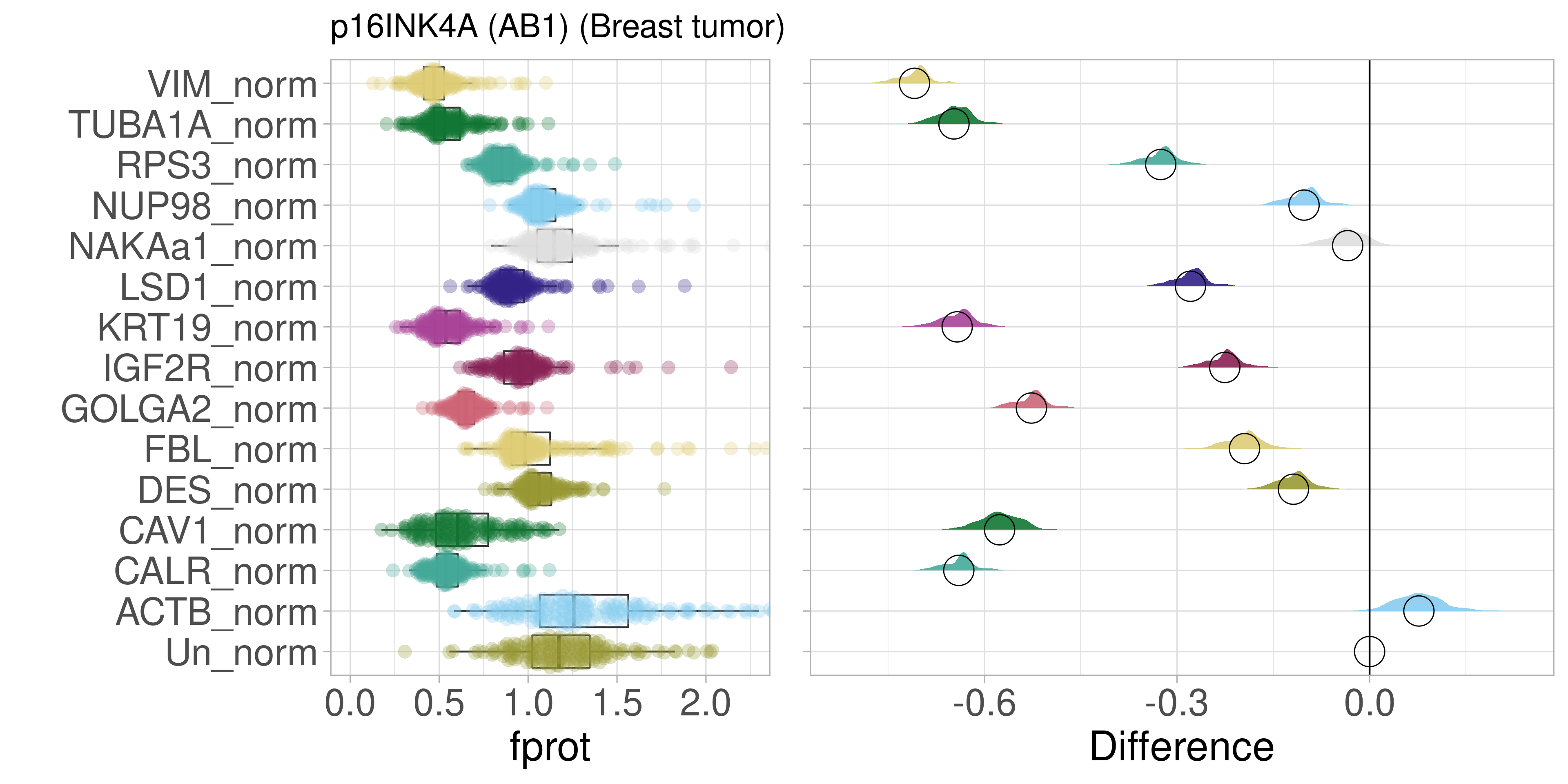

Supplement: Supplementary file 17 — Supplementary Material 17 [file 41598_2026_48754_MOESM17_ESM.zip › RPPA normalizations to cell markers/Breast_Plots/Tumor_suppr_Breast/p16INK4A(AB1)_Breast_T.png]

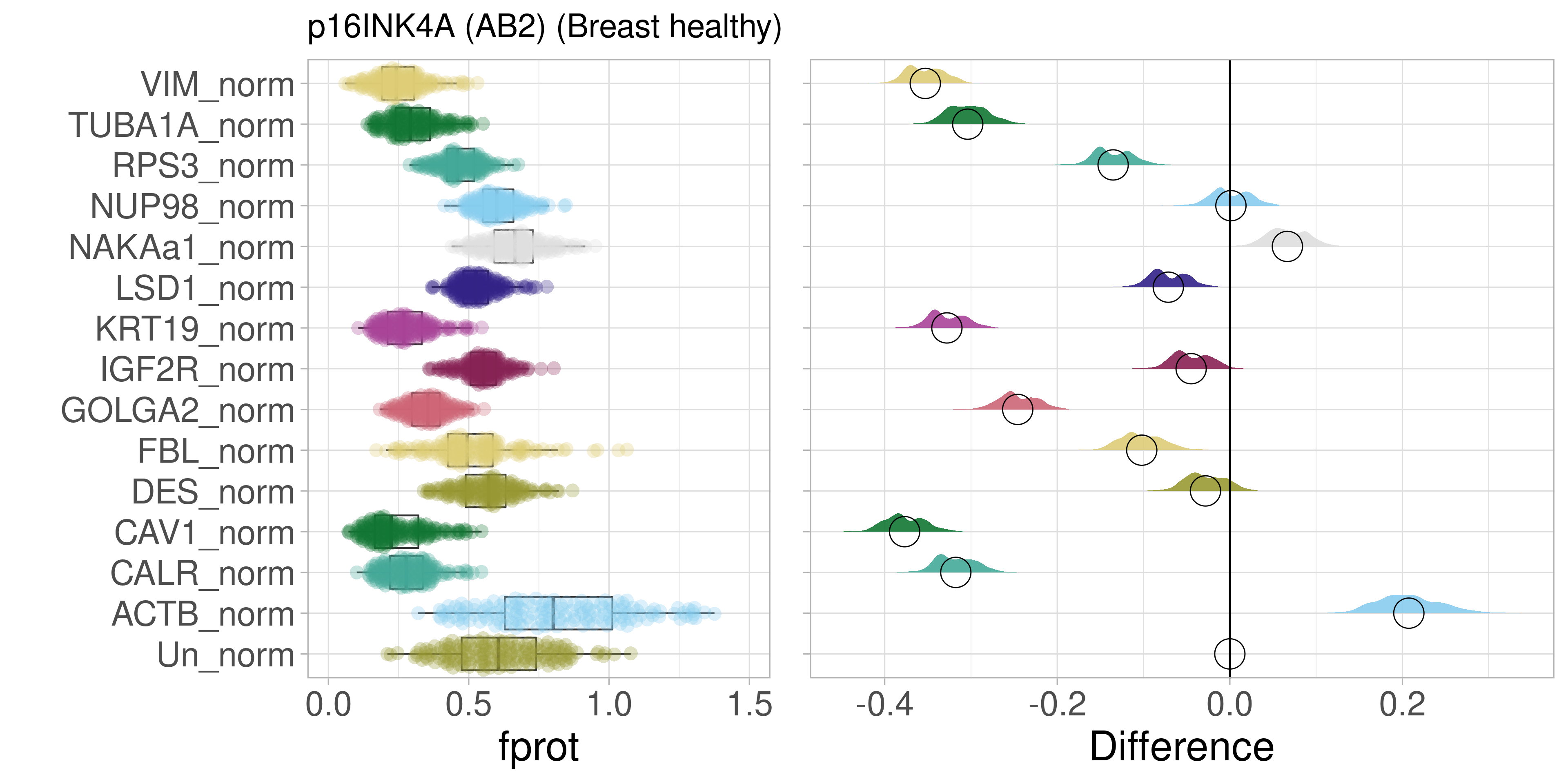

Supplement: Supplementary file 17 — Supplementary Material 17 [file 41598_2026_48754_MOESM17_ESM.zip › RPPA normalizations to cell markers/Breast_Plots/Tumor_suppr_Breast/p16INK4A(AB2)_Breast_H.png]

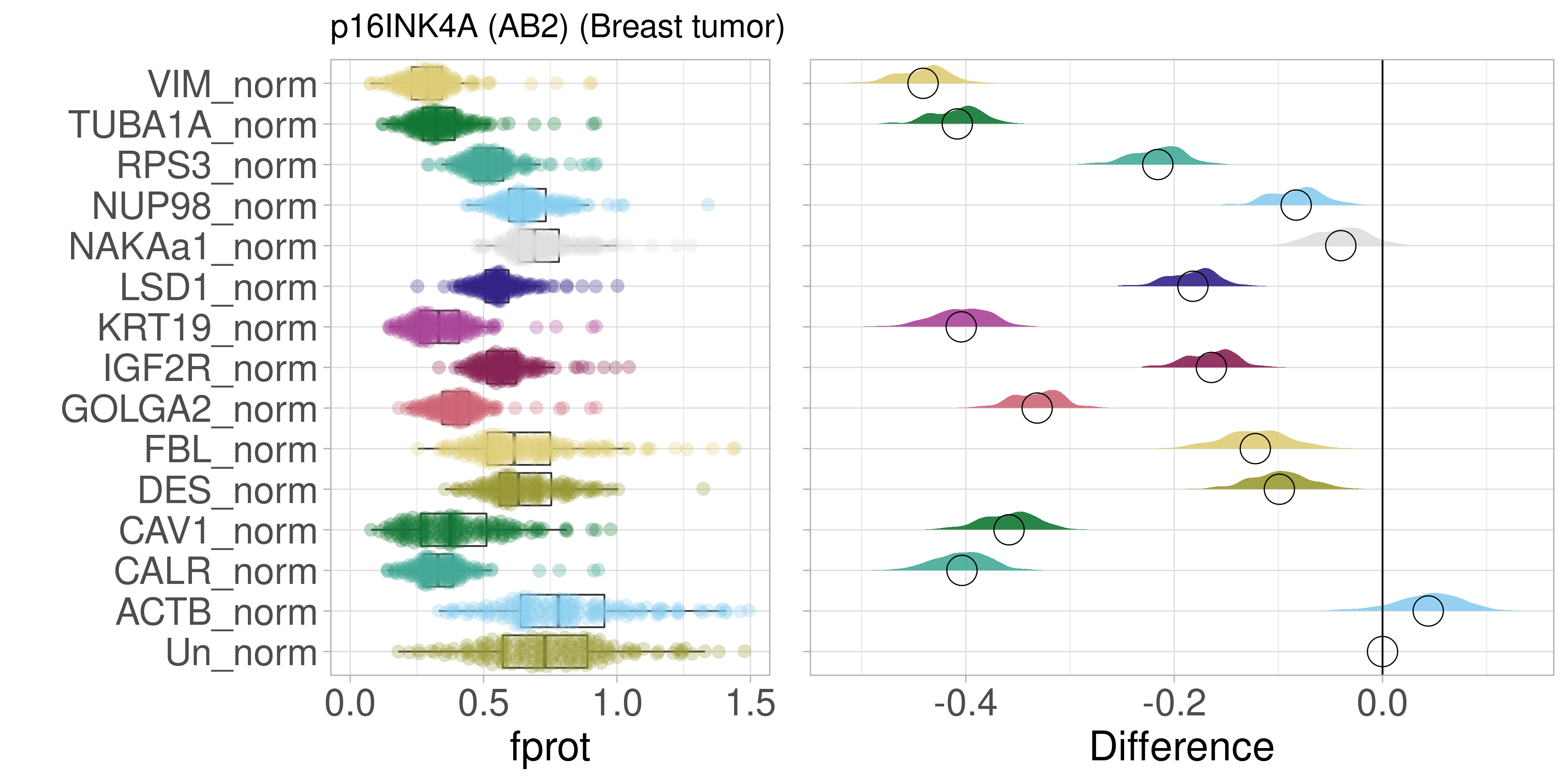

Supplement: Supplementary file 17 — Supplementary Material 17 [file 41598_2026_48754_MOESM17_ESM.zip › RPPA normalizations to cell markers/Breast_Plots/Tumor_suppr_Breast/p16INK4A(AB2)_Breast_T.png]

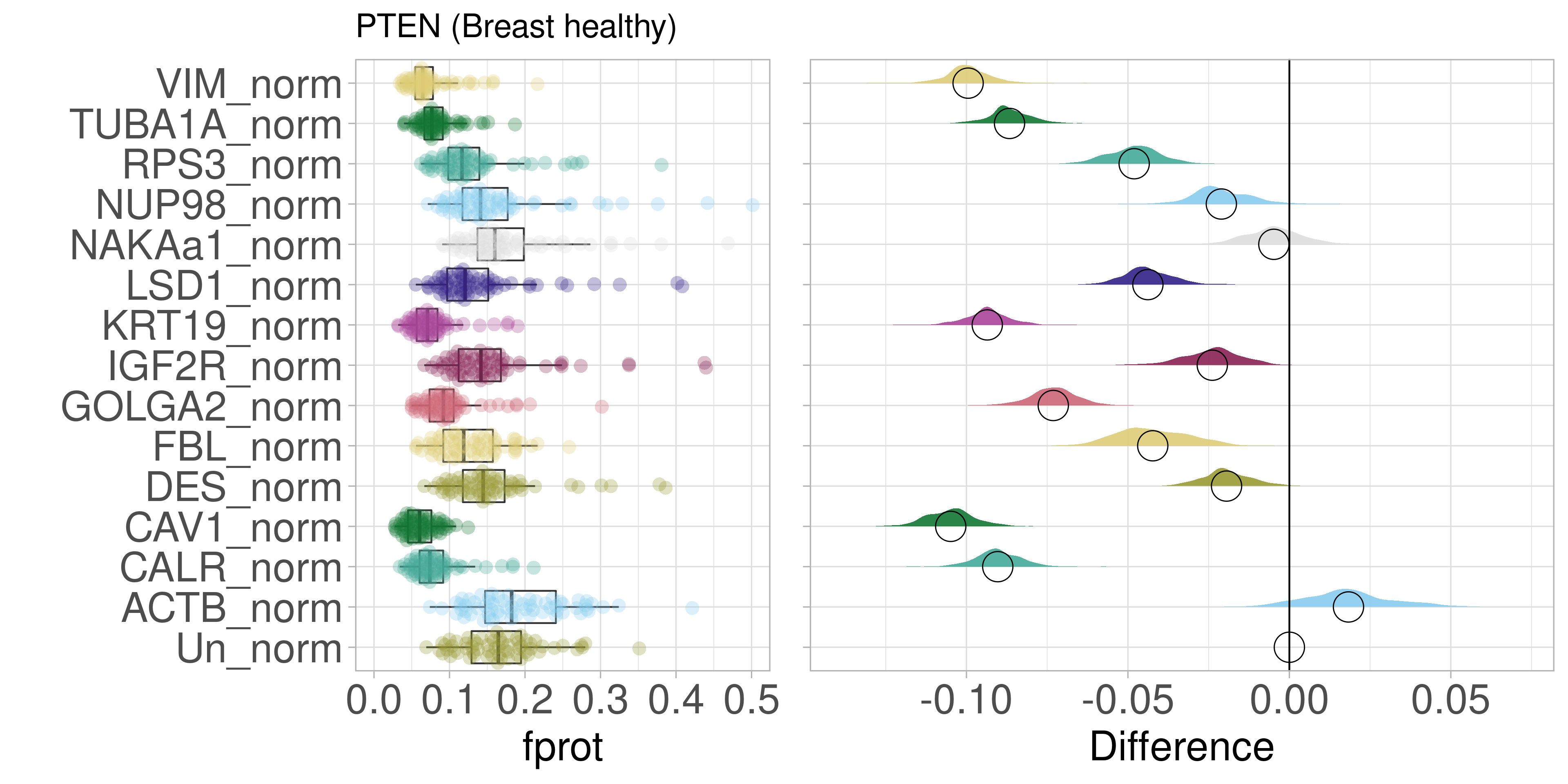

Supplement: Supplementary file 17 — Supplementary Material 17 [file 41598_2026_48754_MOESM17_ESM.zip › RPPA normalizations to cell markers/Breast_Plots/Tumor_suppr_Breast/PTEN_Breast_H.png]

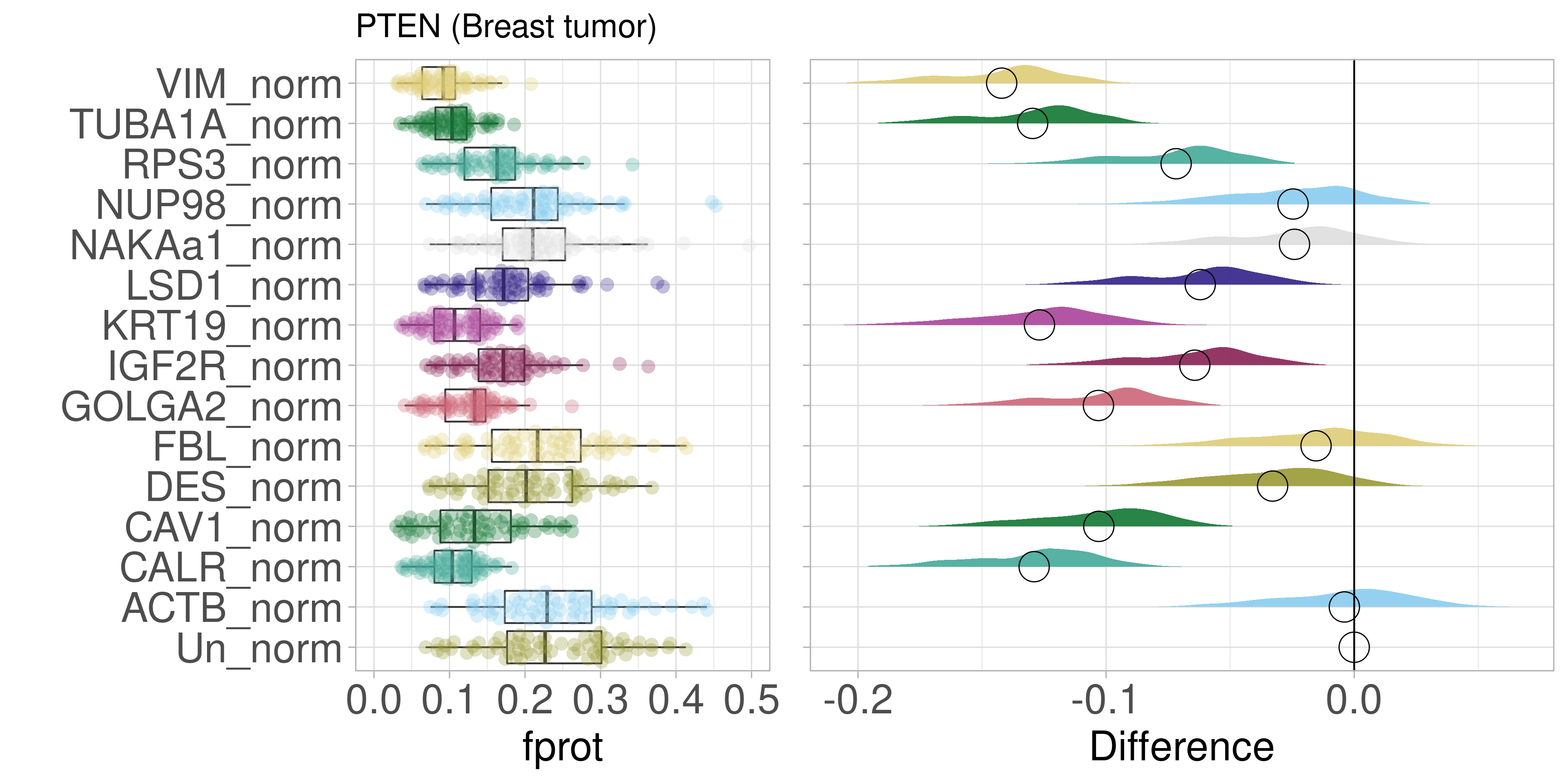

Supplement: Supplementary file 17 — Supplementary Material 17 [file 41598_2026_48754_MOESM17_ESM.zip › RPPA normalizations to cell markers/Breast_Plots/Tumor_suppr_Breast/PTEN_Breast_T.png]

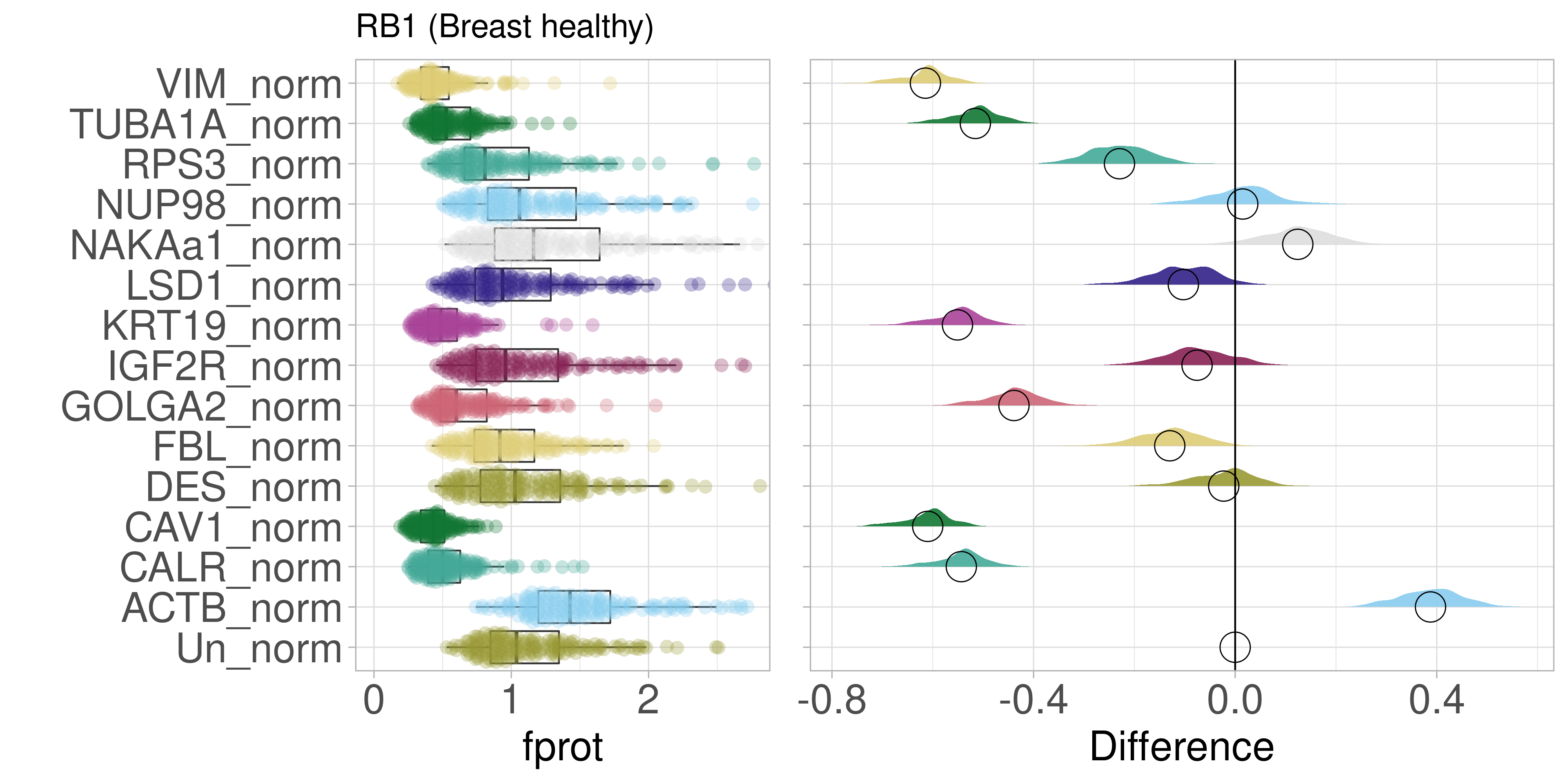

Supplement: Supplementary file 17 — Supplementary Material 17 [file 41598_2026_48754_MOESM17_ESM.zip › RPPA normalizations to cell markers/Breast_Plots/Tumor_suppr_Breast/RB1_Breast_H.png]

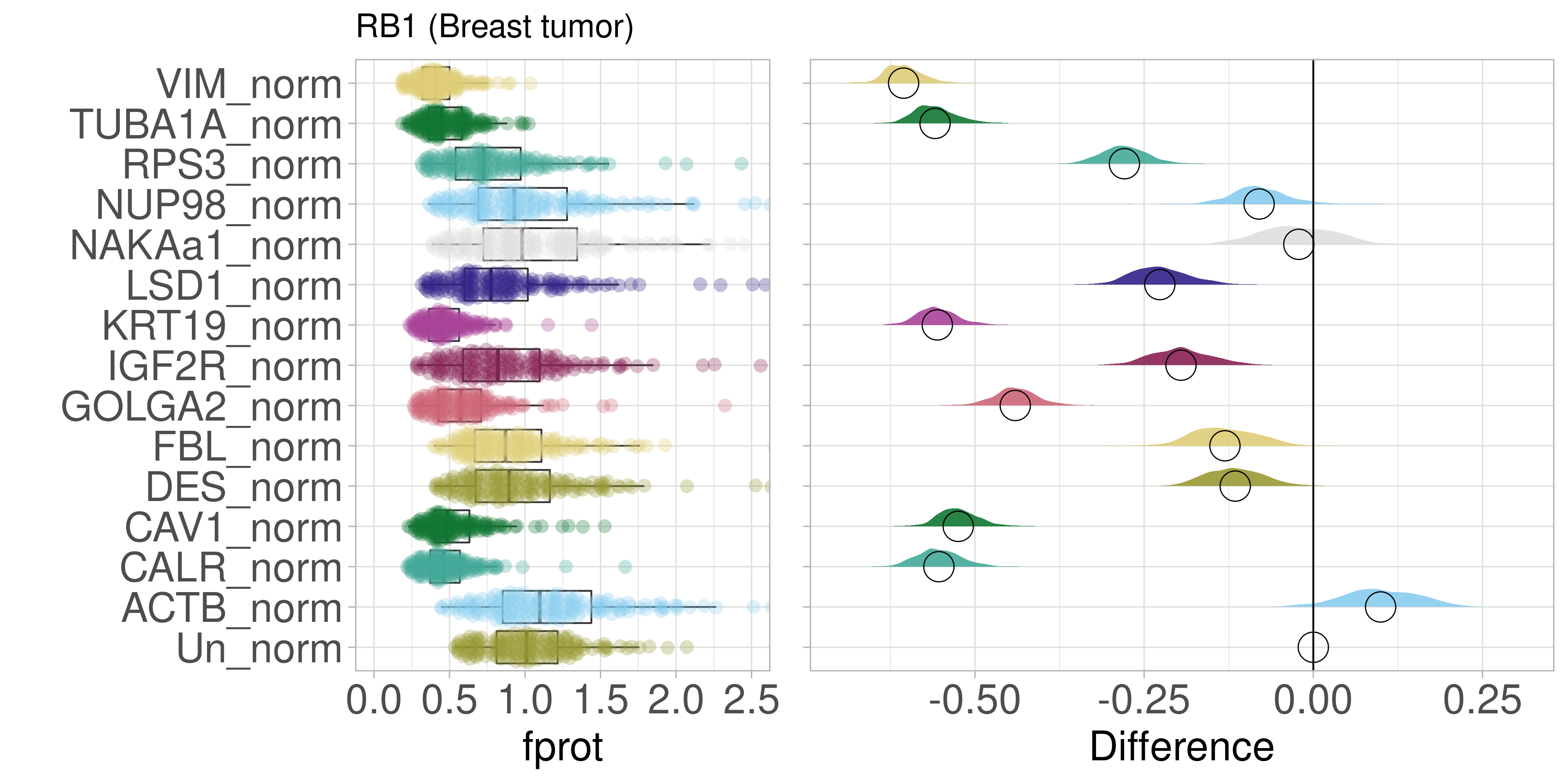

Supplement: Supplementary file 17 — Supplementary Material 17 [file 41598_2026_48754_MOESM17_ESM.zip › RPPA normalizations to cell markers/Breast_Plots/Tumor_suppr_Breast/RB1_Breast_T.png]

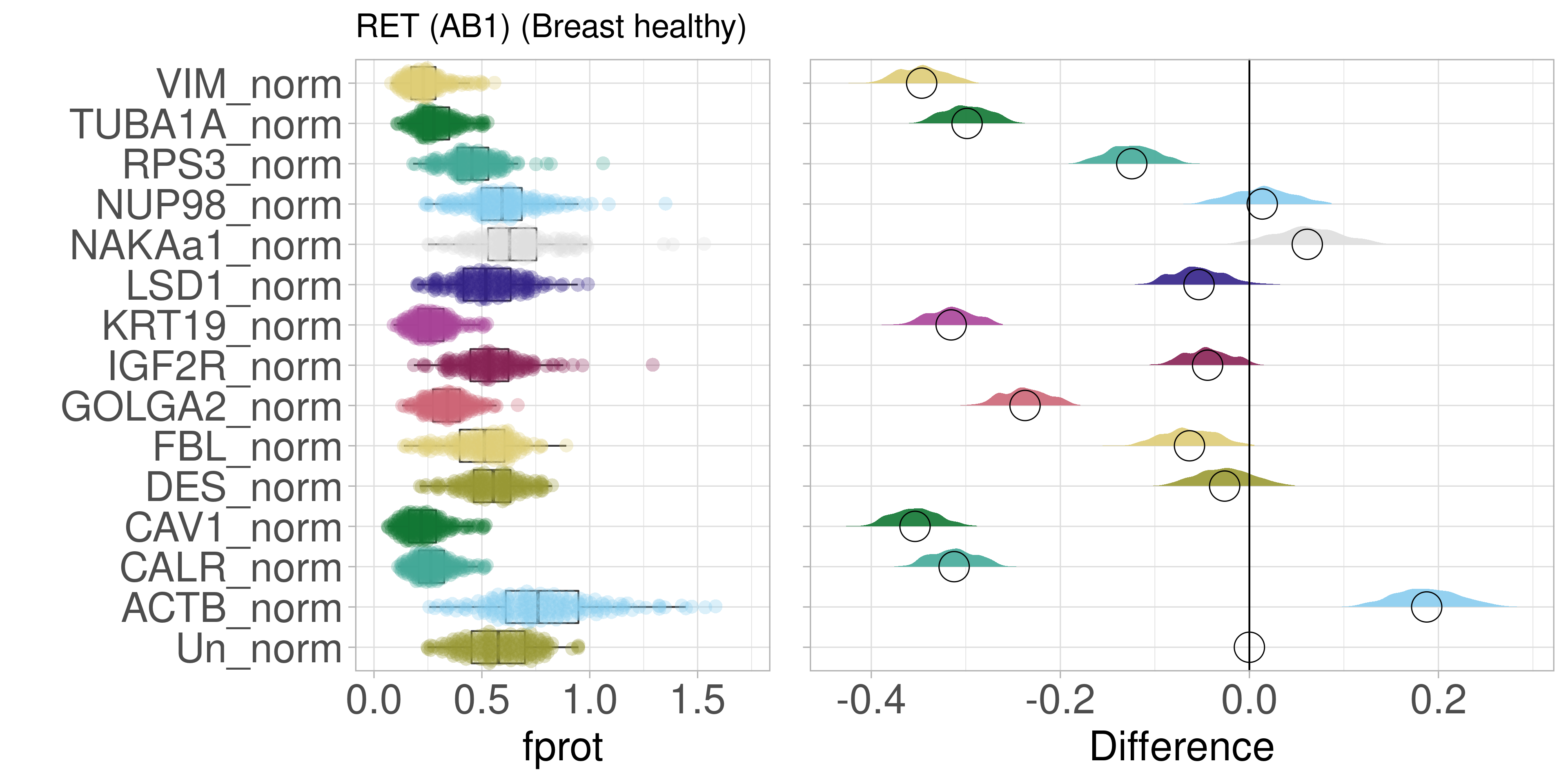

Supplement: Supplementary file 17 — Supplementary Material 17 [file 41598_2026_48754_MOESM17_ESM.zip › RPPA normalizations to cell markers/Breast_Plots/Tumor_suppr_Breast/RET(AB1)_Breast_H.png]

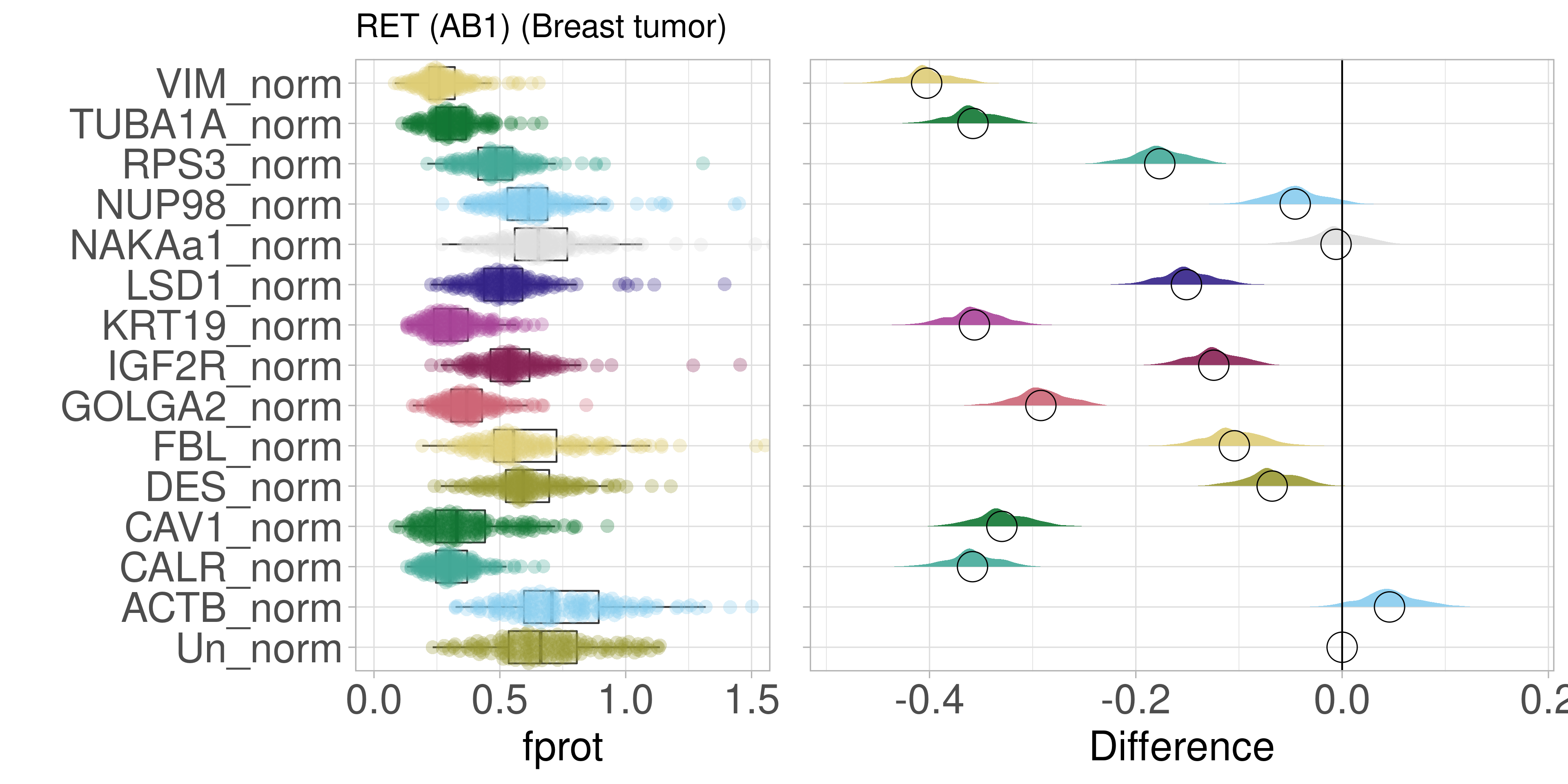

Supplement: Supplementary file 17 — Supplementary Material 17 [file 41598_2026_48754_MOESM17_ESM.zip › RPPA normalizations to cell markers/Breast_Plots/Tumor_suppr_Breast/RET(AB1)_Breast_T.png]

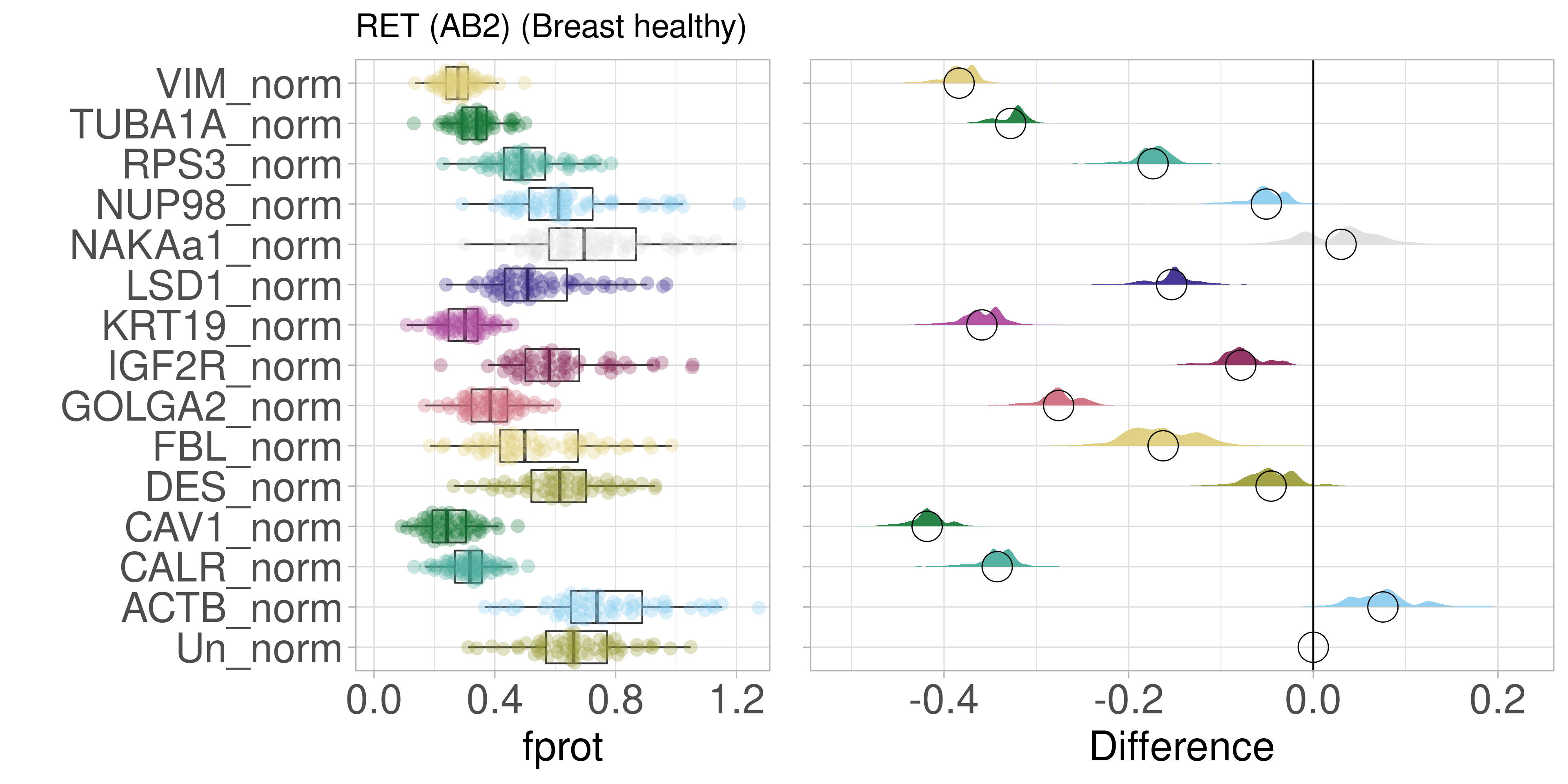

Supplement: Supplementary file 17 — Supplementary Material 17 [file 41598_2026_48754_MOESM17_ESM.zip › RPPA normalizations to cell markers/Breast_Plots/Tumor_suppr_Breast/RET(AB2)_Breast_H.png]

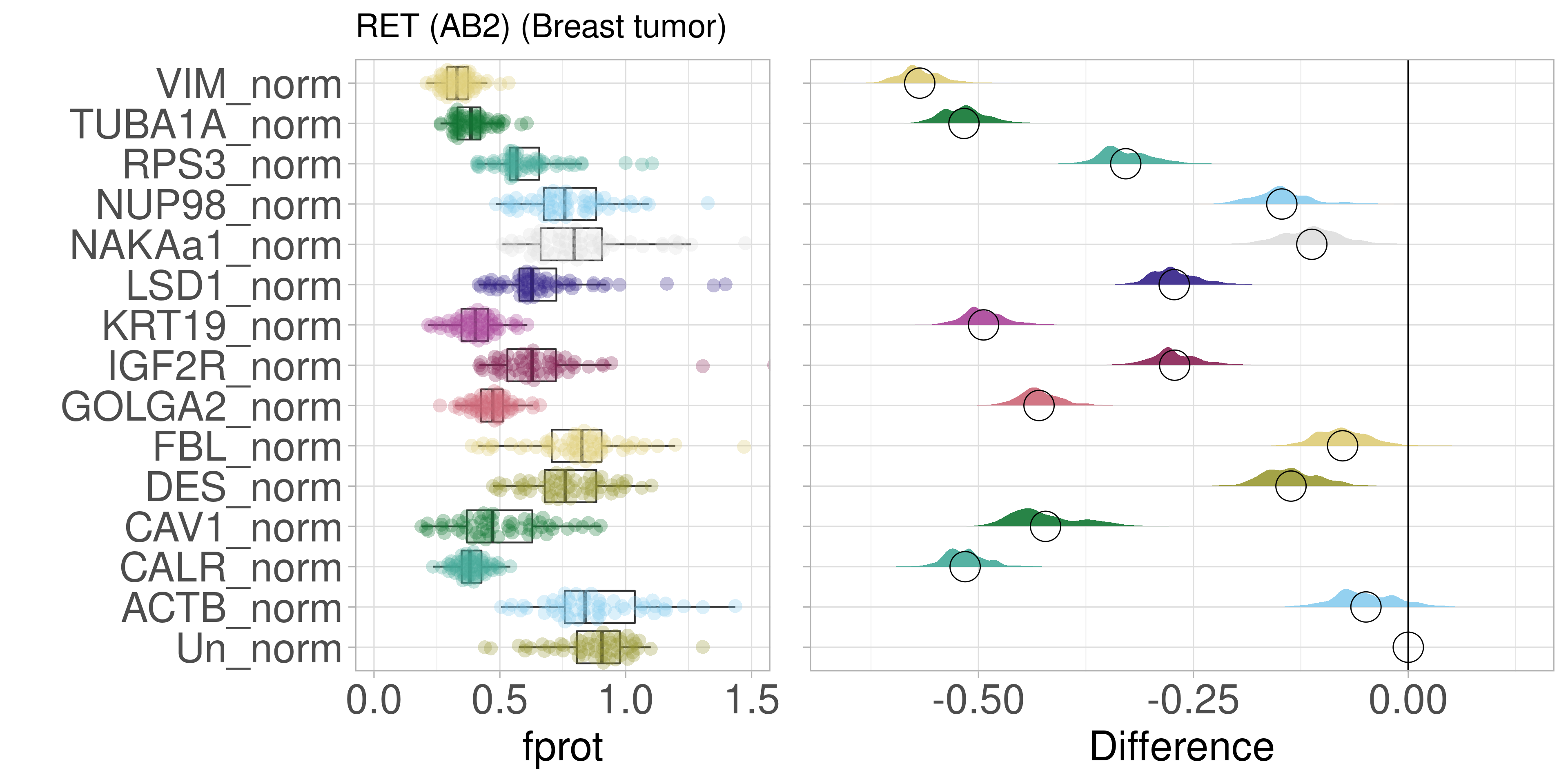

Supplement: Supplementary file 17 — Supplementary Material 17 [file 41598_2026_48754_MOESM17_ESM.zip › RPPA normalizations to cell markers/Breast_Plots/Tumor_suppr_Breast/RET(AB2)_Breast_T.png]

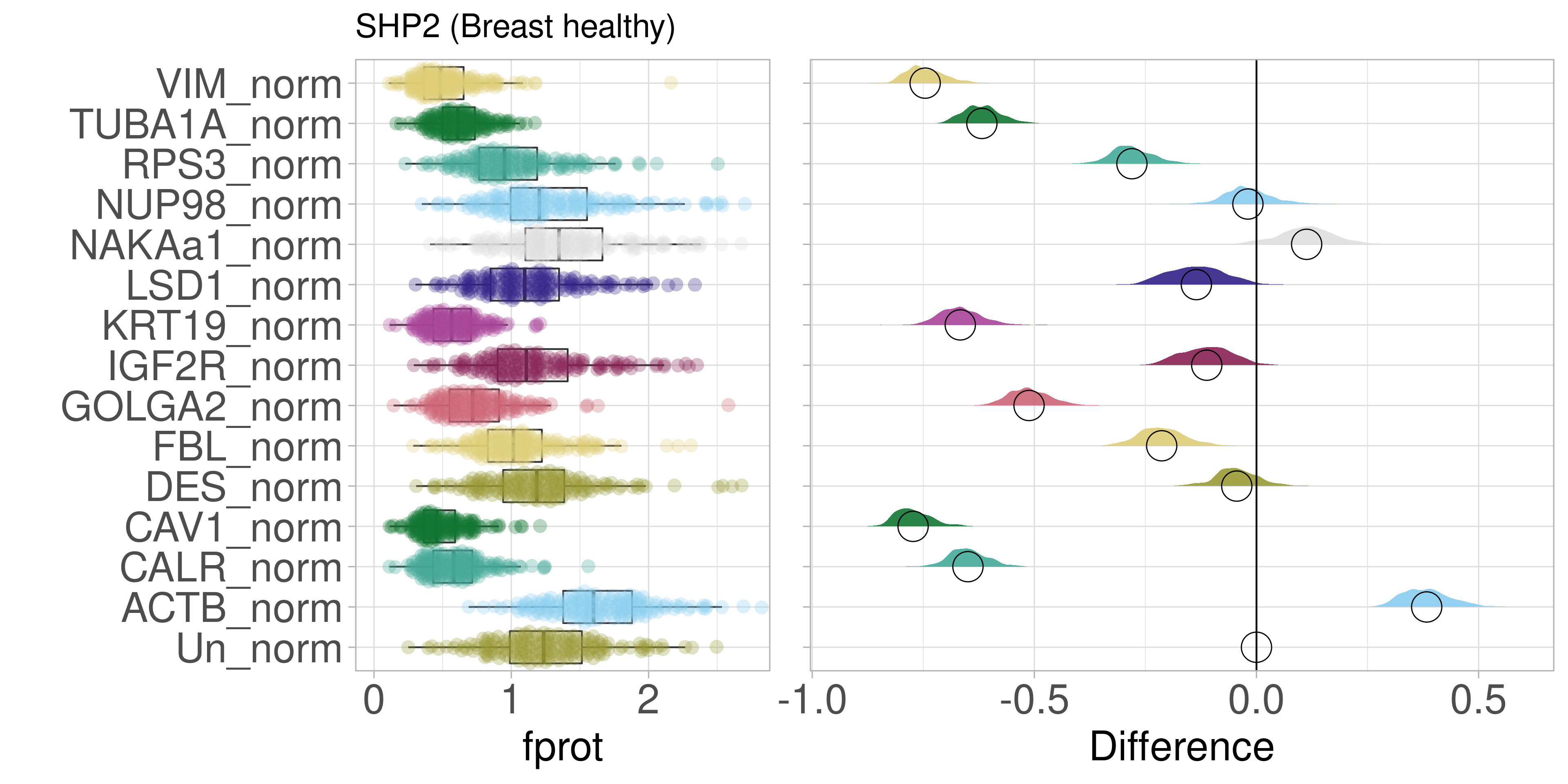

Supplement: Supplementary file 17 — Supplementary Material 17 [file 41598_2026_48754_MOESM17_ESM.zip › RPPA normalizations to cell markers/Breast_Plots/Tumor_suppr_Breast/SHP2_Breast_H.png]

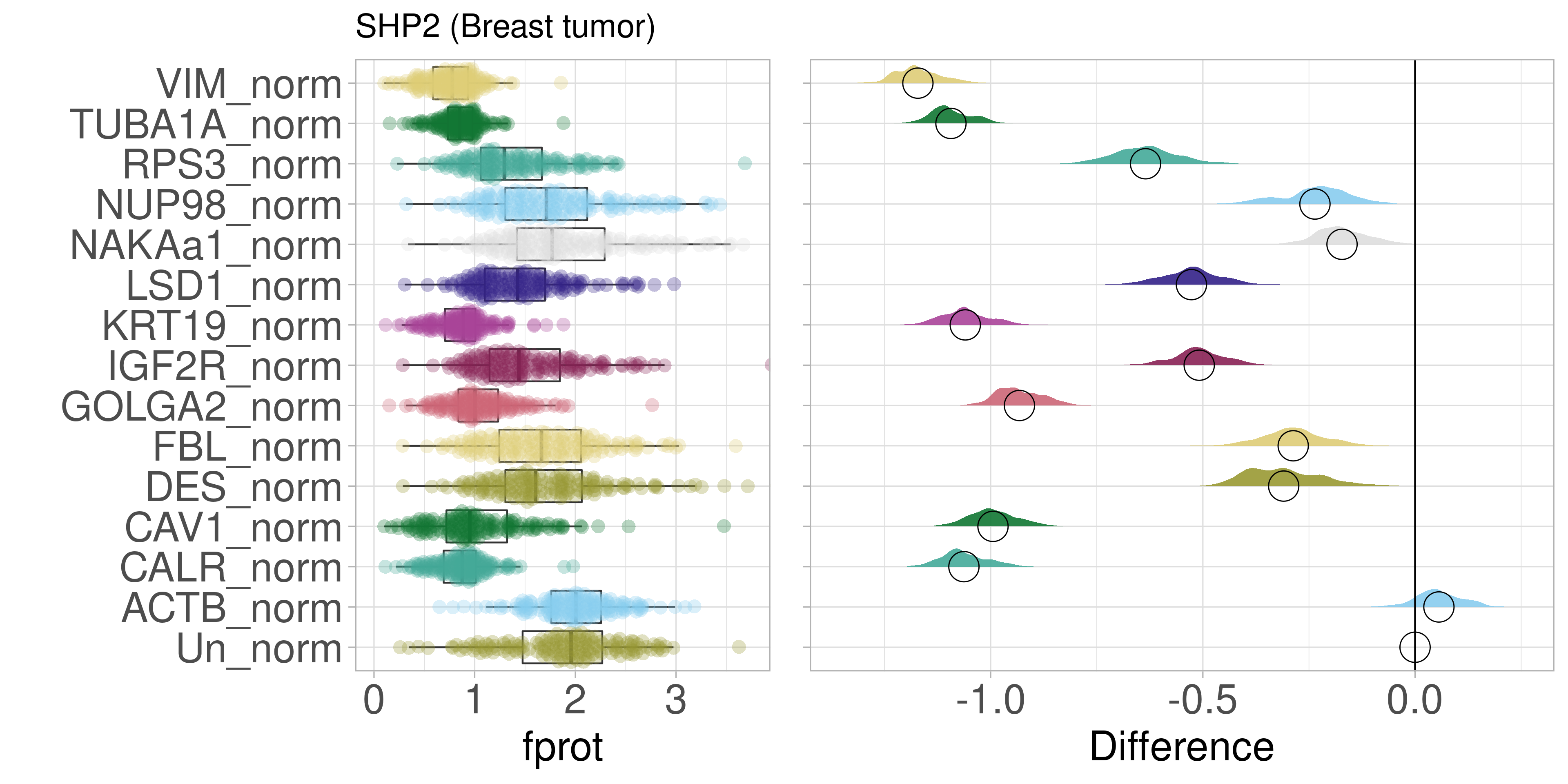

Supplement: Supplementary file 17 — Supplementary Material 17 [file 41598_2026_48754_MOESM17_ESM.zip › RPPA normalizations to cell markers/Breast_Plots/Tumor_suppr_Breast/SHP2_Breast_T.png]

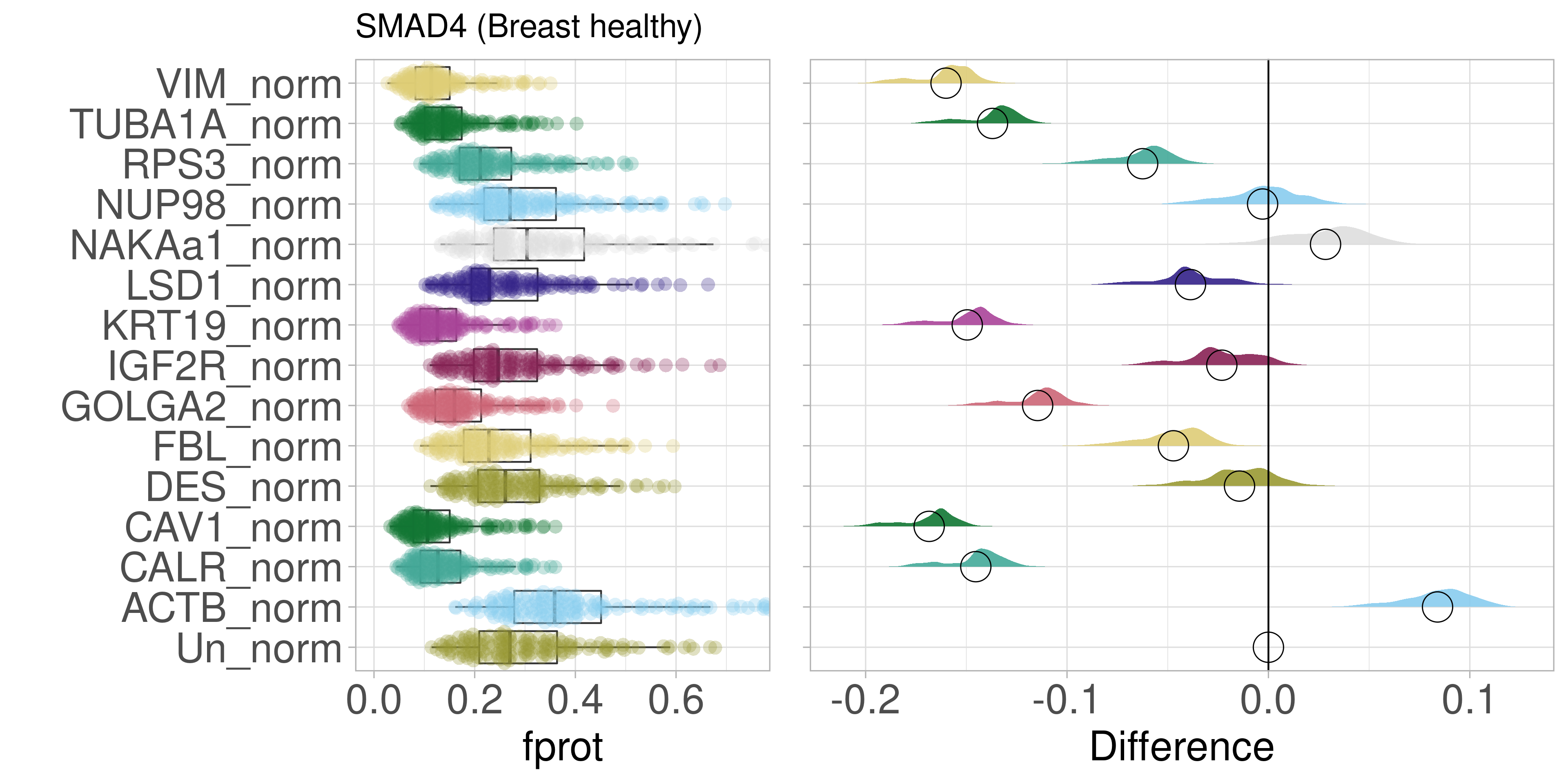

Supplement: Supplementary file 17 — Supplementary Material 17 [file 41598_2026_48754_MOESM17_ESM.zip › RPPA normalizations to cell markers/Breast_Plots/Tumor_suppr_Breast/SMAD4_Breast_H.png]

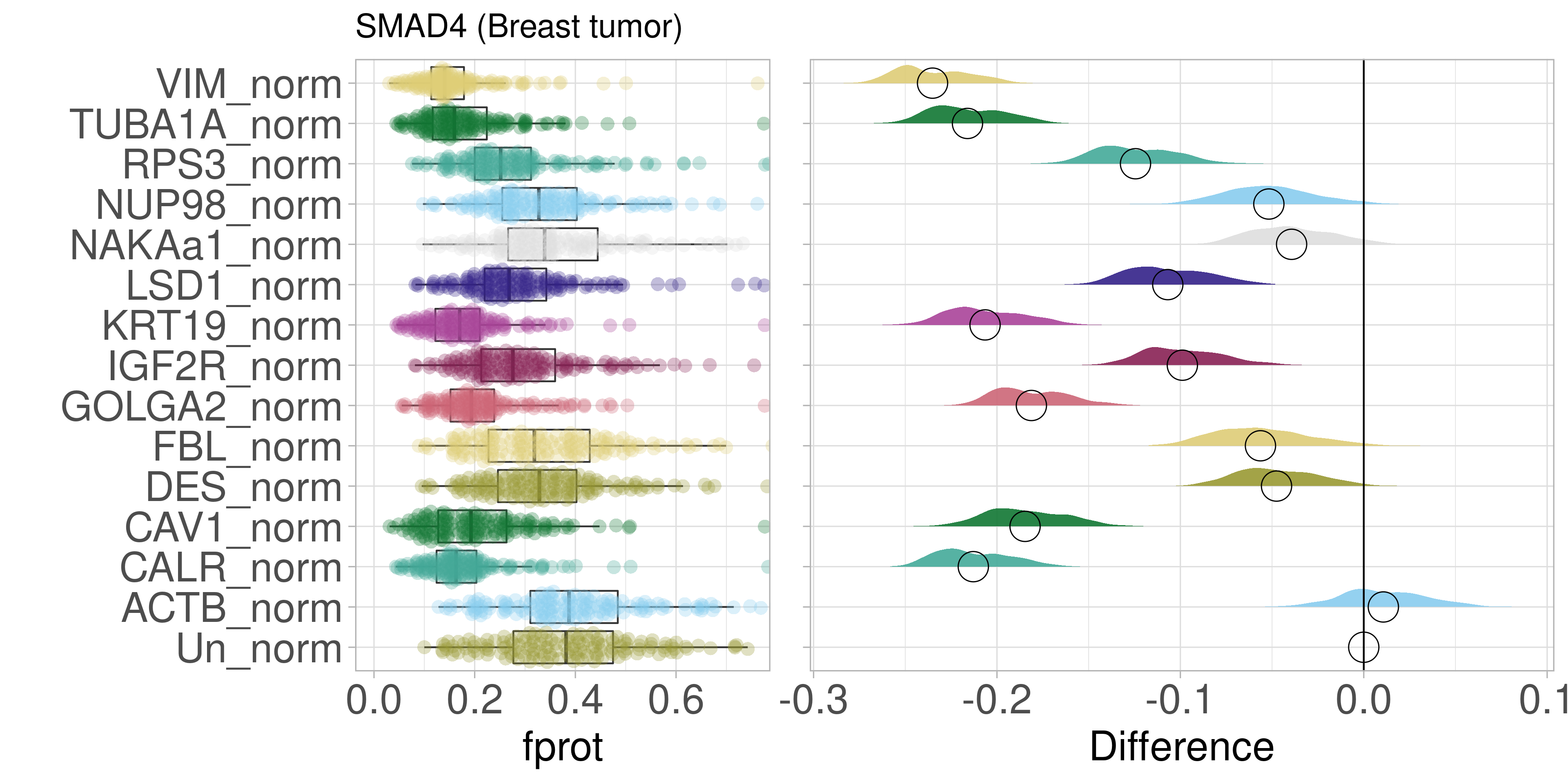

Supplement: Supplementary file 17 — Supplementary Material 17 [file 41598_2026_48754_MOESM17_ESM.zip › RPPA normalizations to cell markers/Breast_Plots/Tumor_suppr_Breast/SMAD4_Breast_T.png]

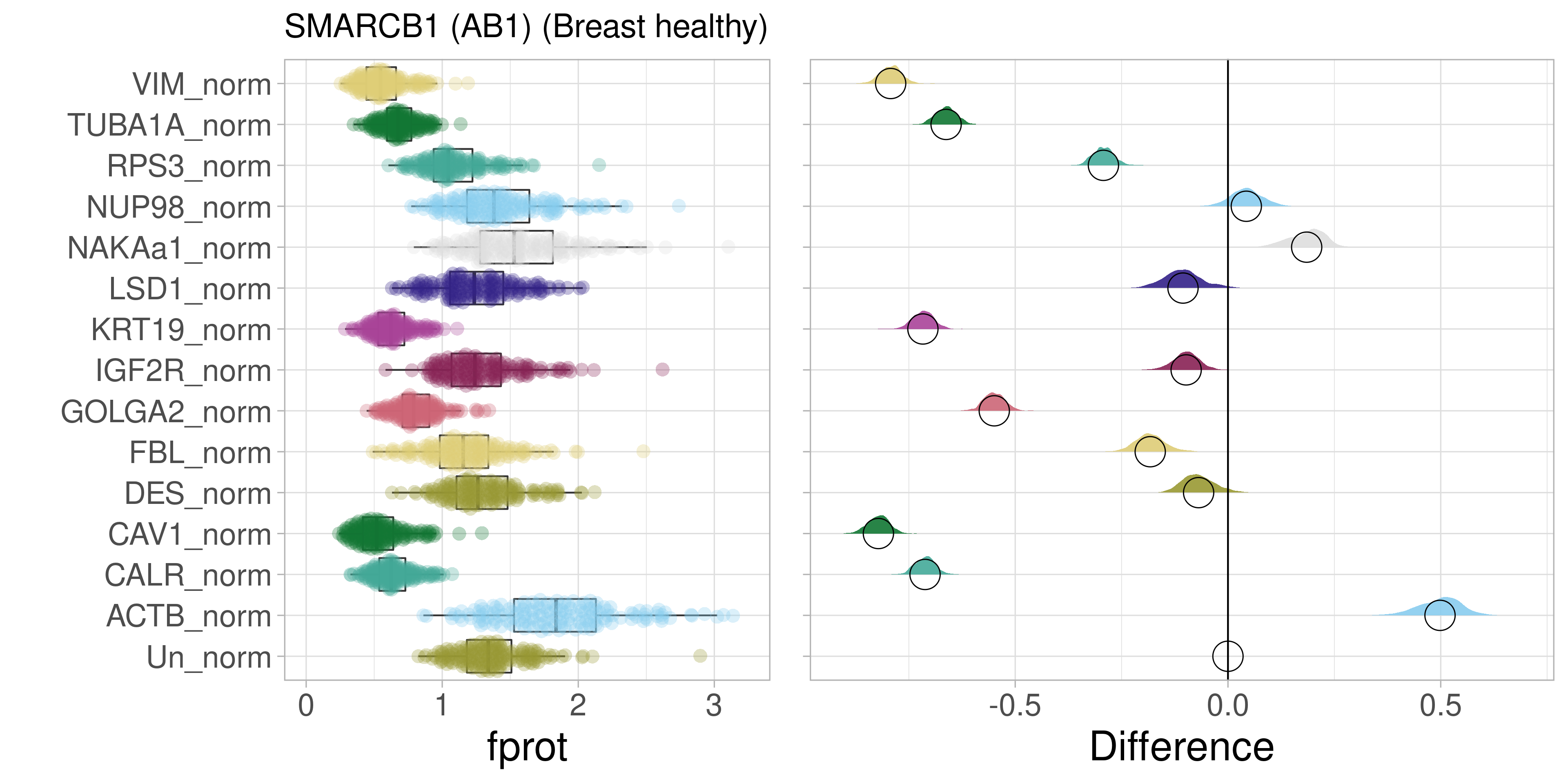

Supplement: Supplementary file 17 — Supplementary Material 17 [file 41598_2026_48754_MOESM17_ESM.zip › RPPA normalizations to cell markers/Breast_Plots/Tumor_suppr_Breast/SMARCB1(AB1)_Breast_H.png]

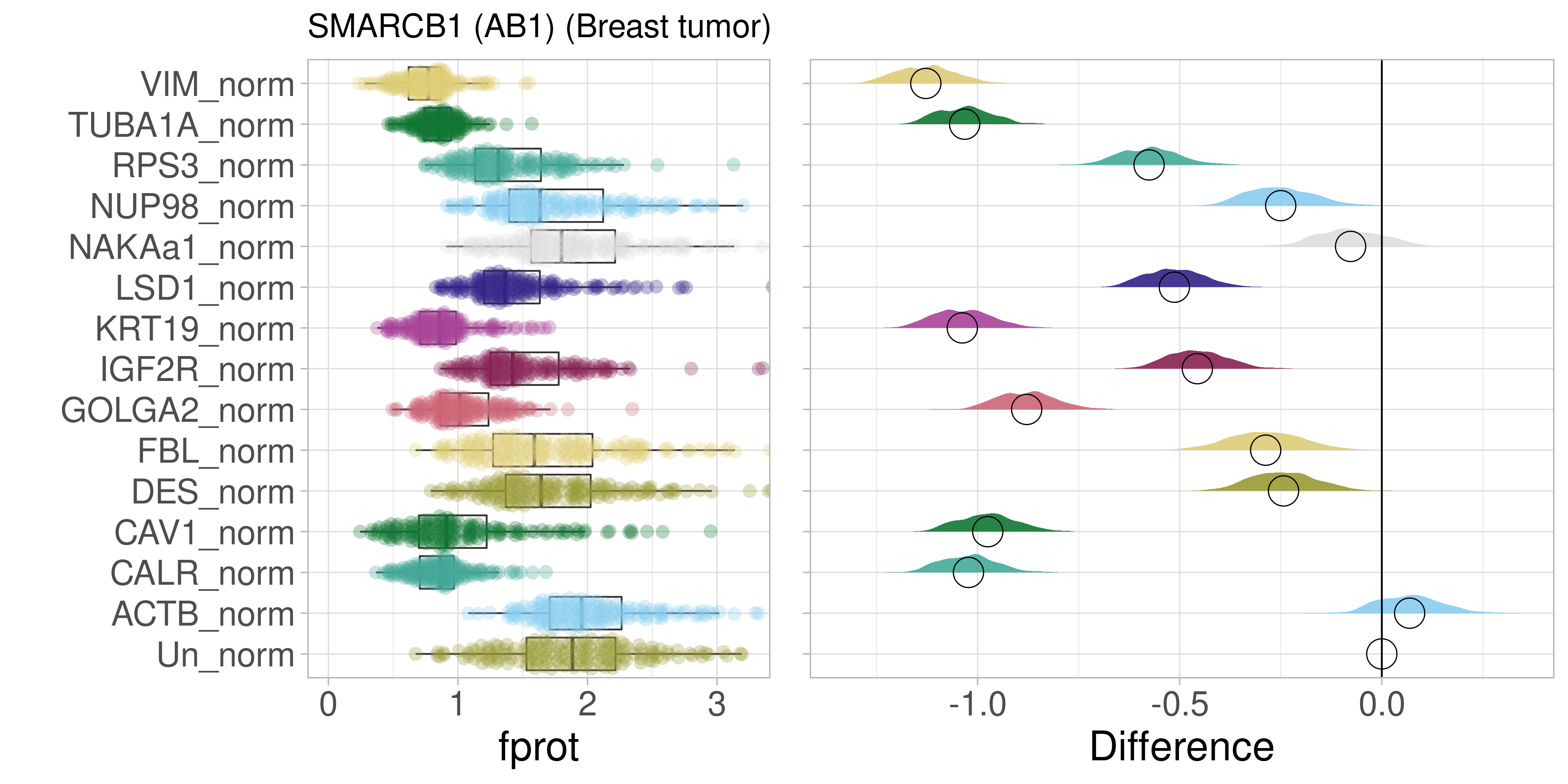

Supplement: Supplementary file 17 — Supplementary Material 17 [file 41598_2026_48754_MOESM17_ESM.zip › RPPA normalizations to cell markers/Breast_Plots/Tumor_suppr_Breast/SMARCB1(AB1)_Breast_T.png]

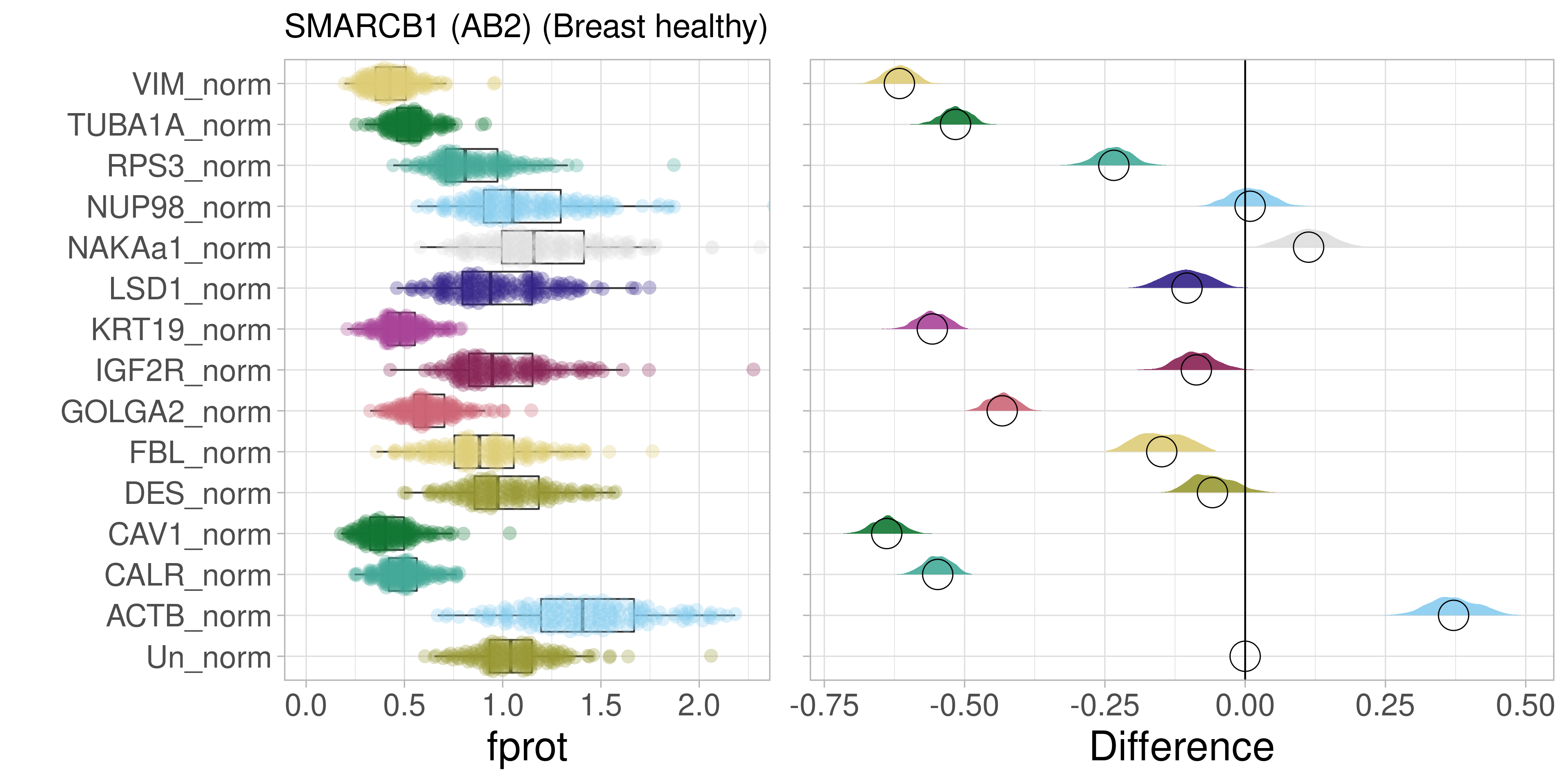

Supplement: Supplementary file 17 — Supplementary Material 17 [file 41598_2026_48754_MOESM17_ESM.zip › RPPA normalizations to cell markers/Breast_Plots/Tumor_suppr_Breast/SMARCB1(AB2)_Breast_H.png]

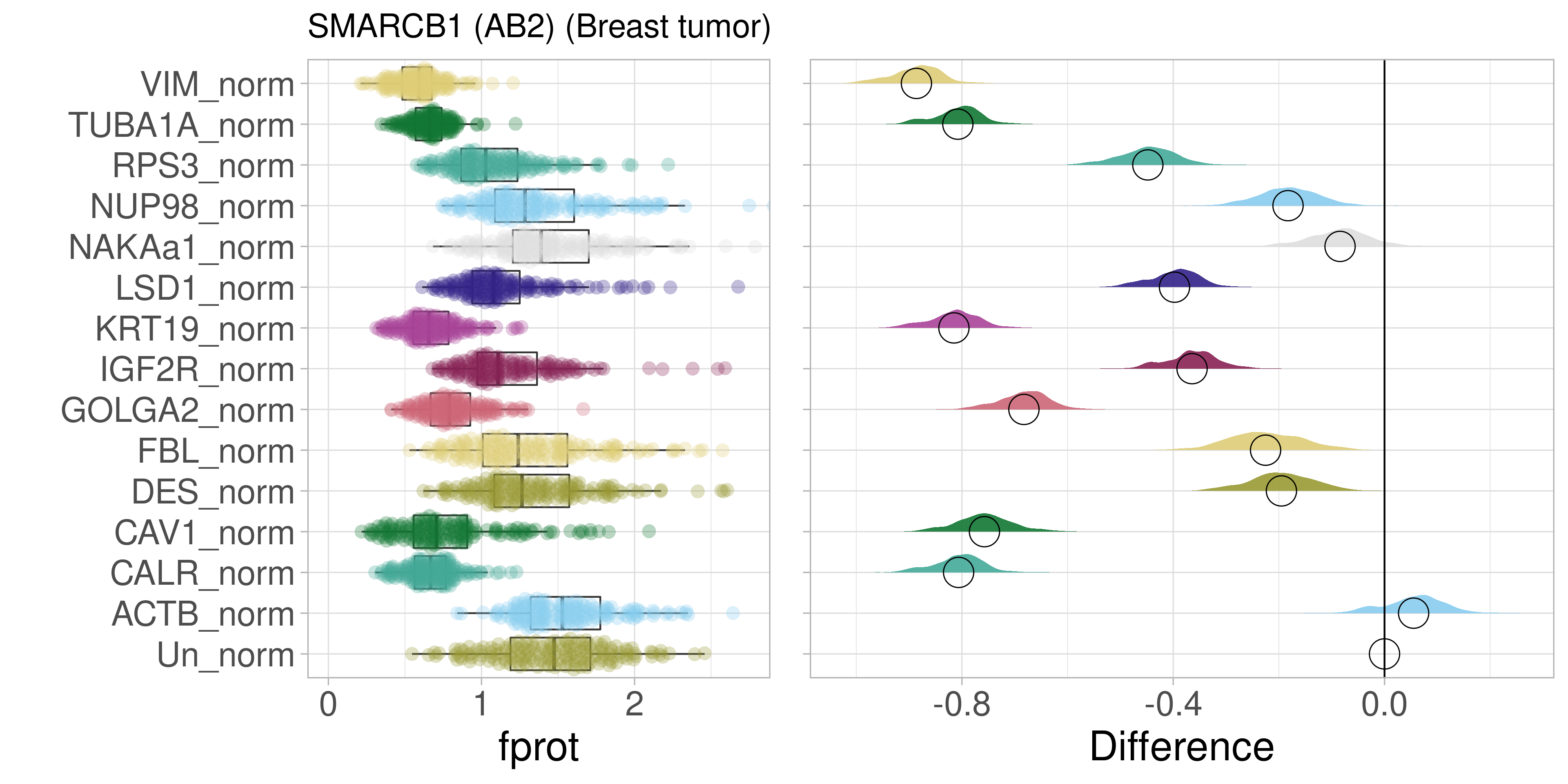

Supplement: Supplementary file 17 — Supplementary Material 17 [file 41598_2026_48754_MOESM17_ESM.zip › RPPA normalizations to cell markers/Breast_Plots/Tumor_suppr_Breast/SMARCB1(AB2)_Breast_T.png]

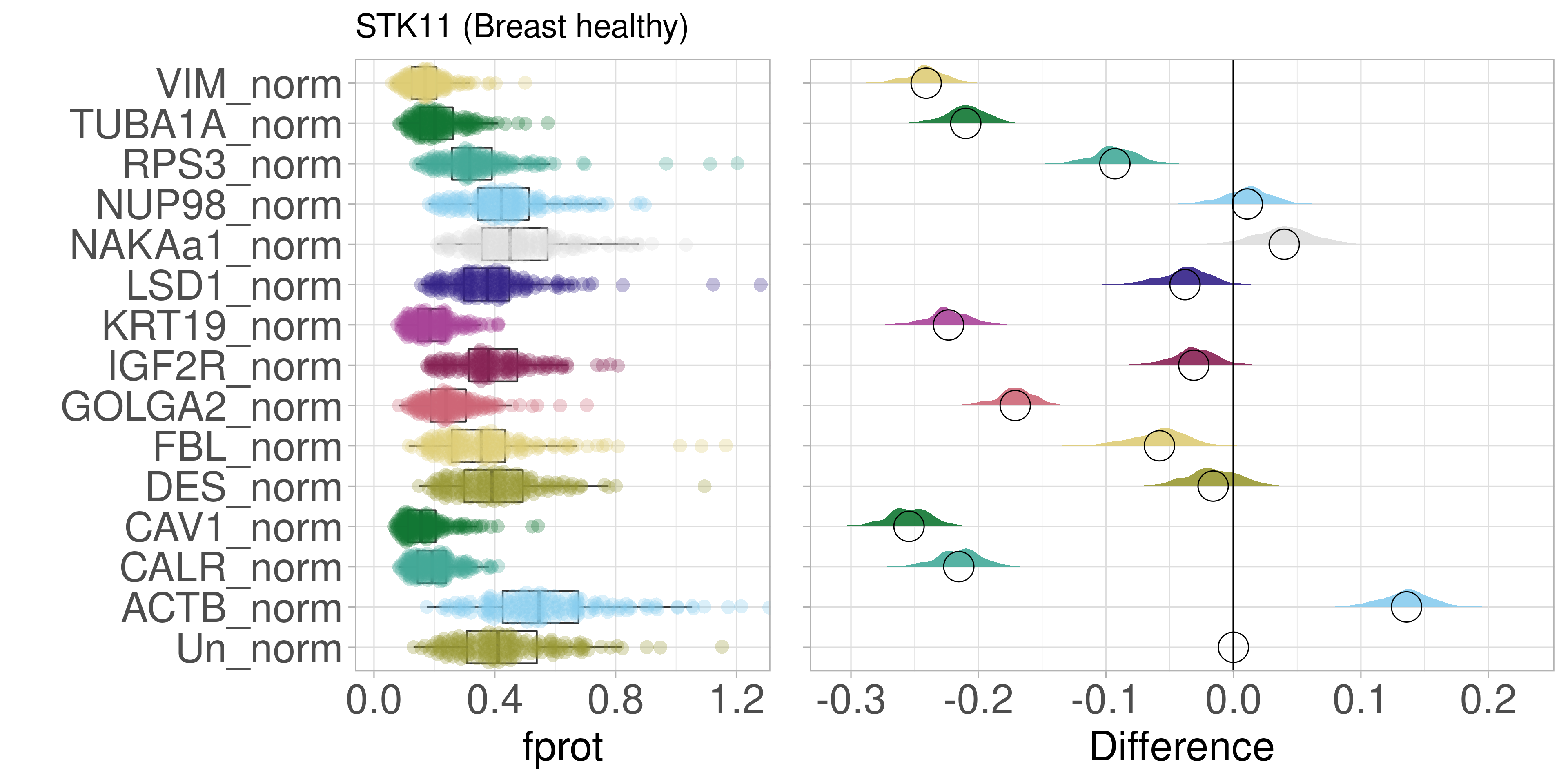

Supplement: Supplementary file 17 — Supplementary Material 17 [file 41598_2026_48754_MOESM17_ESM.zip › RPPA normalizations to cell markers/Breast_Plots/Tumor_suppr_Breast/STK11_Breast_H.png]

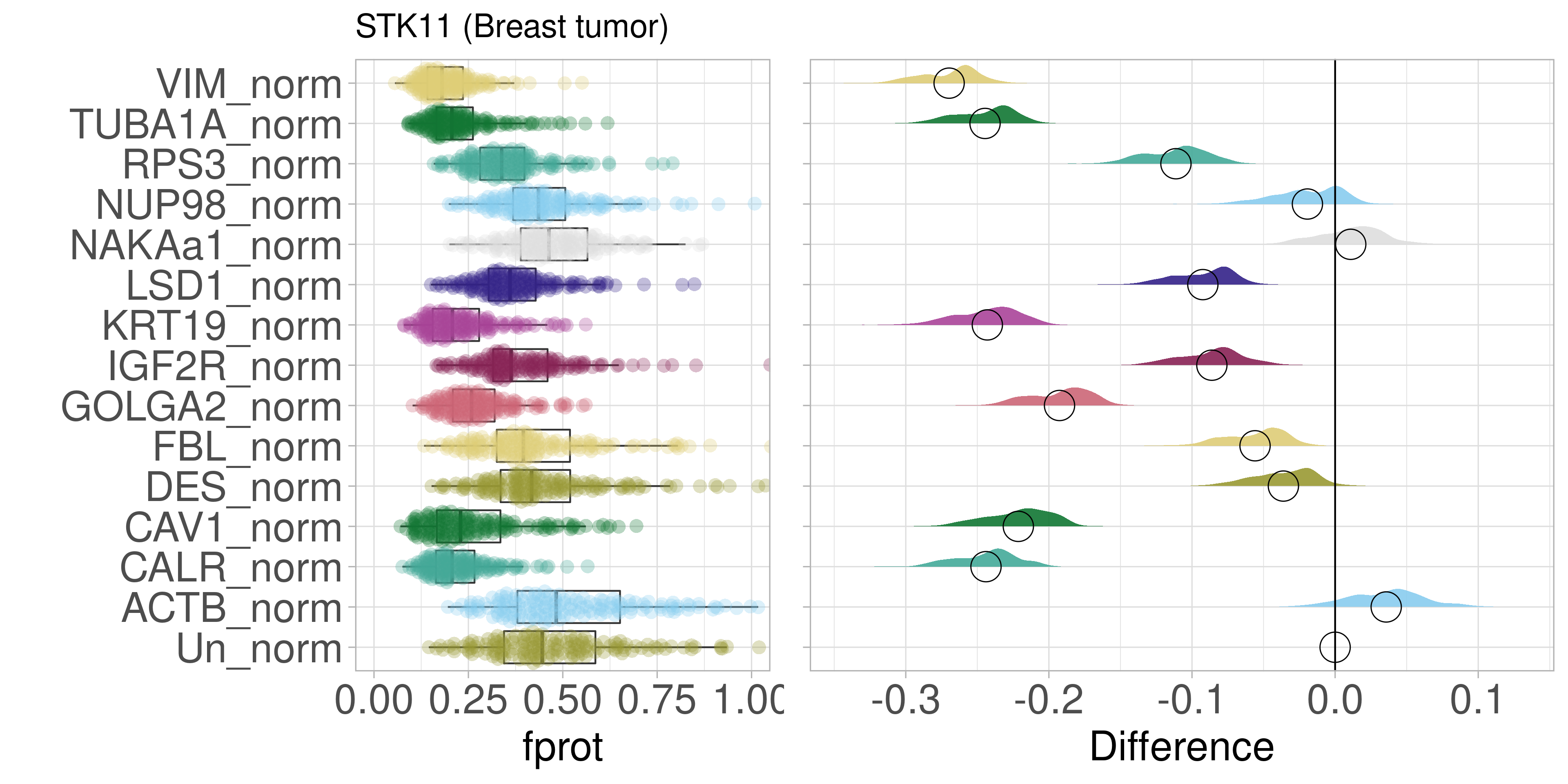

Supplement: Supplementary file 17 — Supplementary Material 17 [file 41598_2026_48754_MOESM17_ESM.zip › RPPA normalizations to cell markers/Breast_Plots/Tumor_suppr_Breast/STK11_Breast_T.png]

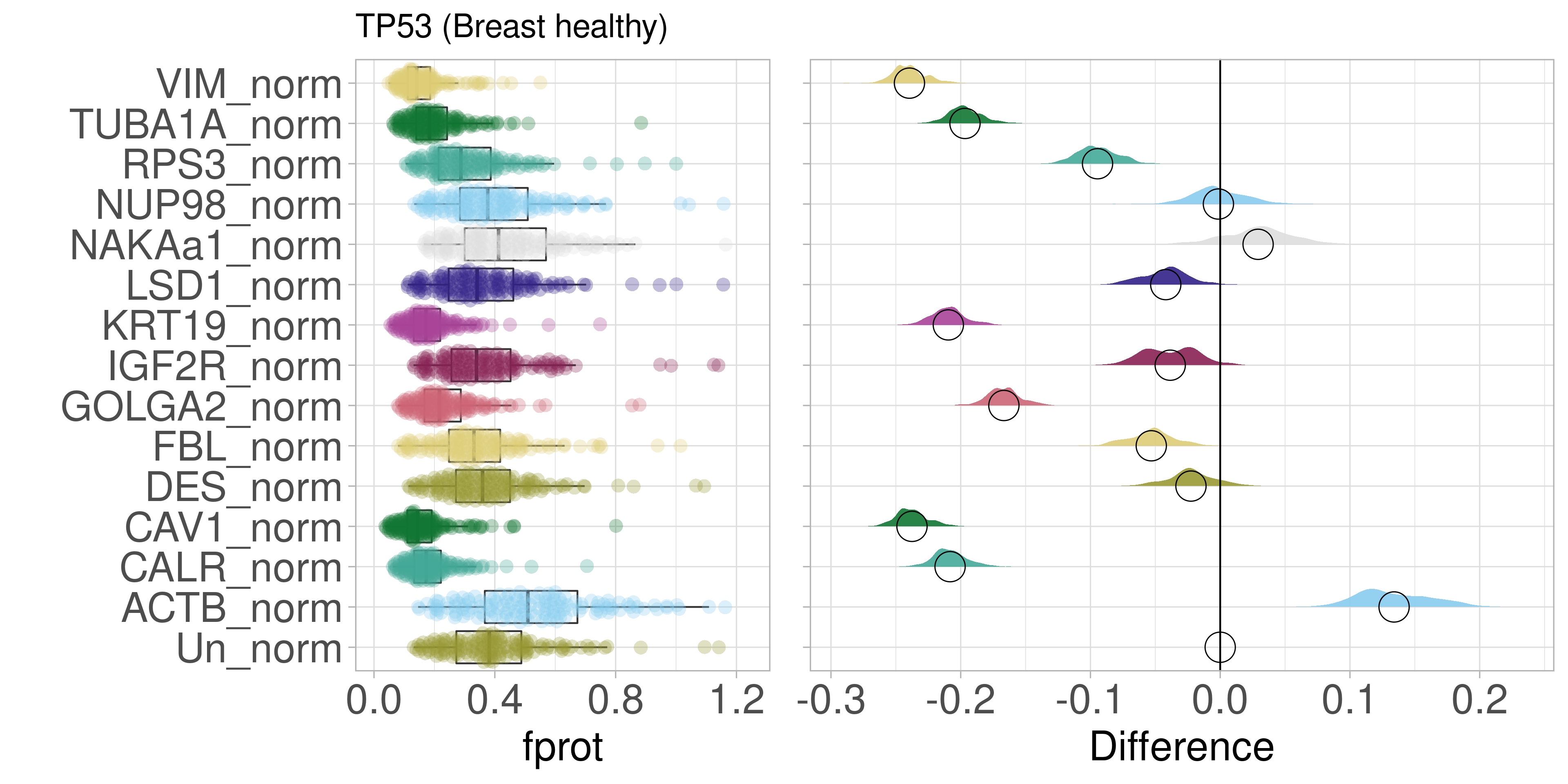

Supplement: Supplementary file 17 — Supplementary Material 17 [file 41598_2026_48754_MOESM17_ESM.zip › RPPA normalizations to cell markers/Breast_Plots/Tumor_suppr_Breast/TP53_Breast_H.png]

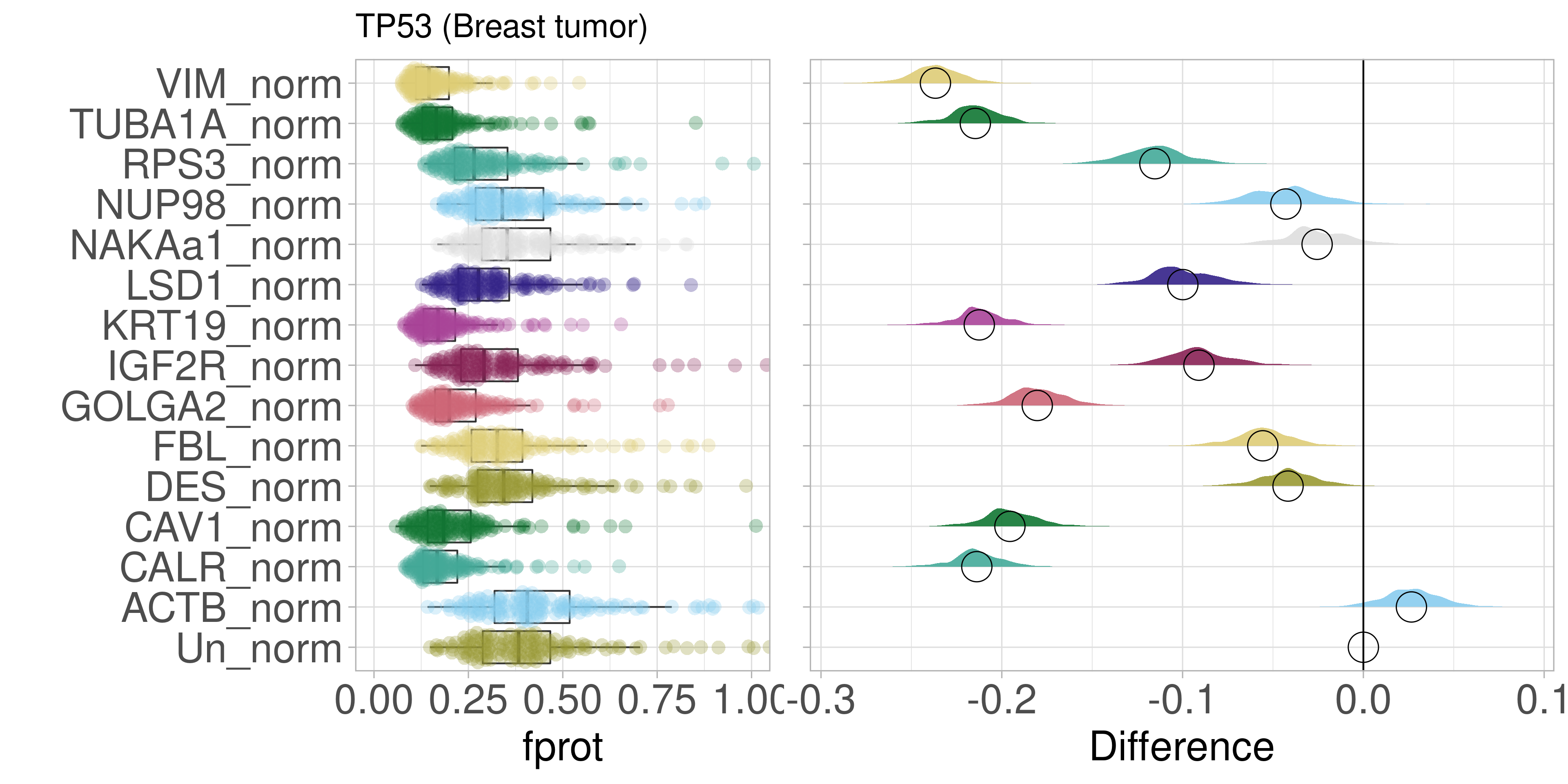

Supplement: Supplementary file 17 — Supplementary Material 17 [file 41598_2026_48754_MOESM17_ESM.zip › RPPA normalizations to cell markers/Breast_Plots/Tumor_suppr_Breast/TP53_Breast_T.png]

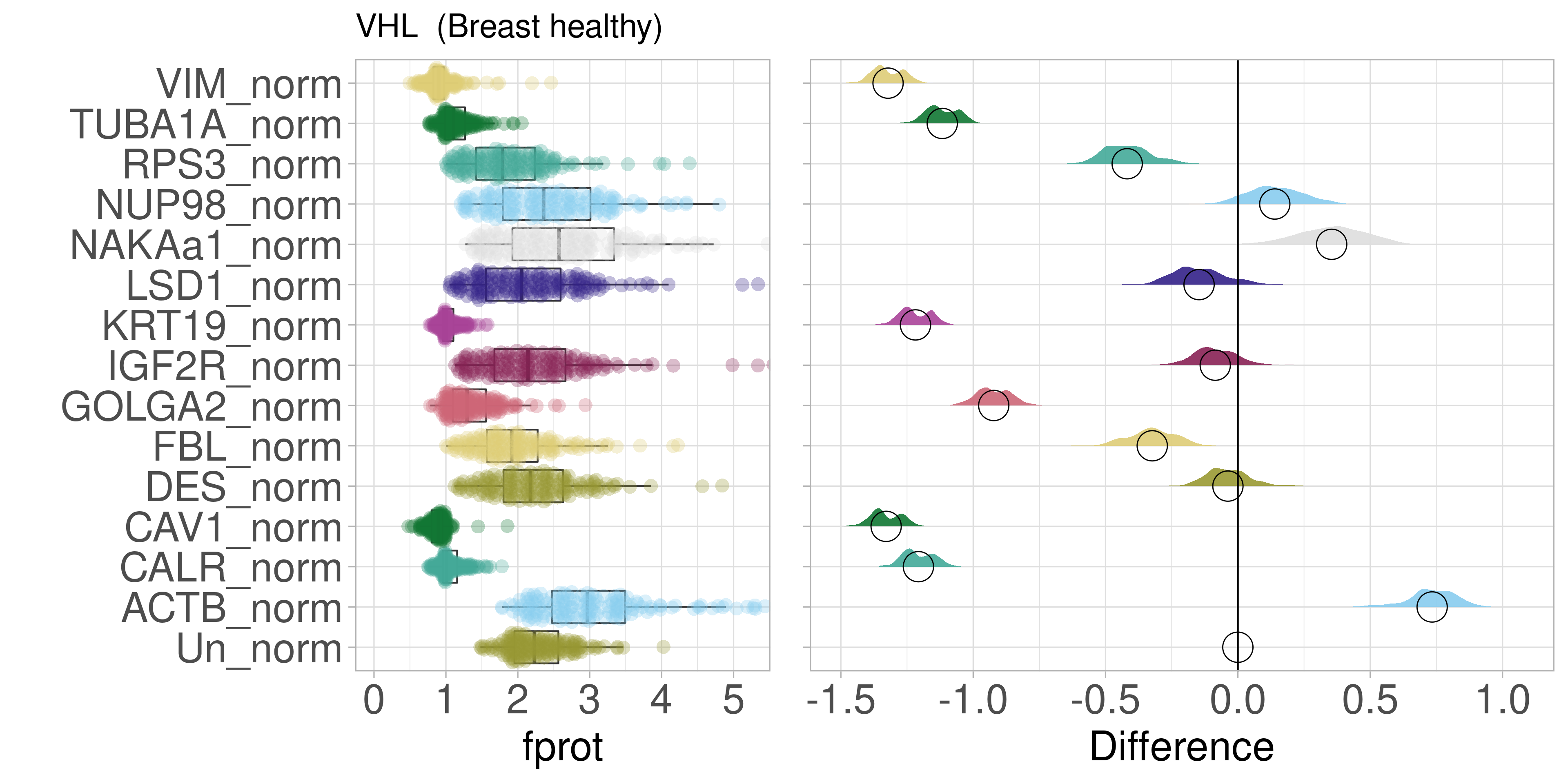

Supplement: Supplementary file 17 — Supplementary Material 17 [file 41598_2026_48754_MOESM17_ESM.zip › RPPA normalizations to cell markers/Breast_Plots/Tumor_suppr_Breast/VHL_Breast_H.png]

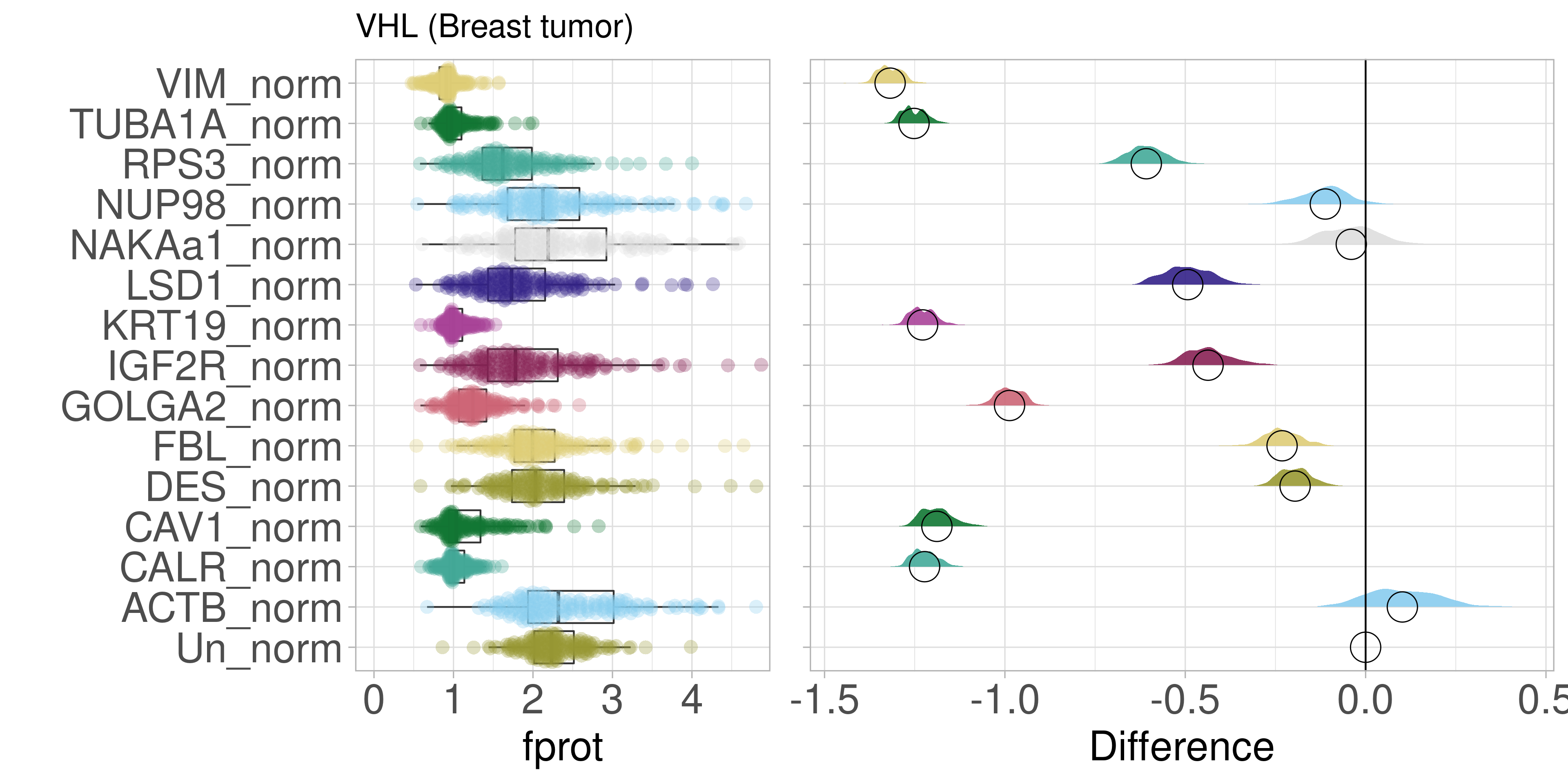

Supplement: Supplementary file 17 — Supplementary Material 17 [file 41598_2026_48754_MOESM17_ESM.zip › RPPA normalizations to cell markers/Breast_Plots/Tumor_suppr_Breast/VHL_Breast_T.png]

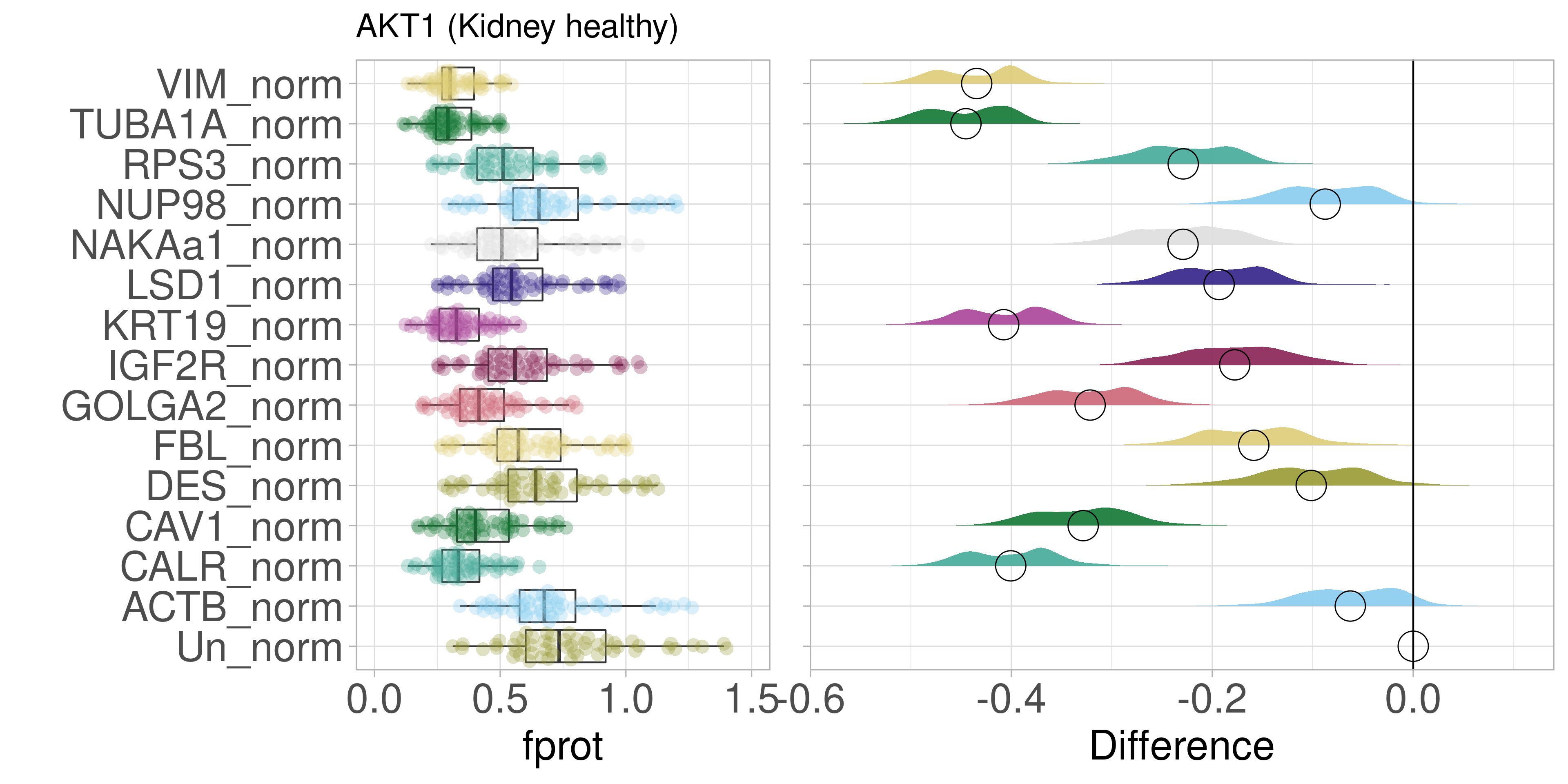

Supplement: Supplementary file 17 — Supplementary Material 17 [file 41598_2026_48754_MOESM17_ESM.zip › RPPA normalizations to cell markers/Kidney_plots/Oncoprotein_Kidney/AKT1_Kidney_H.png]

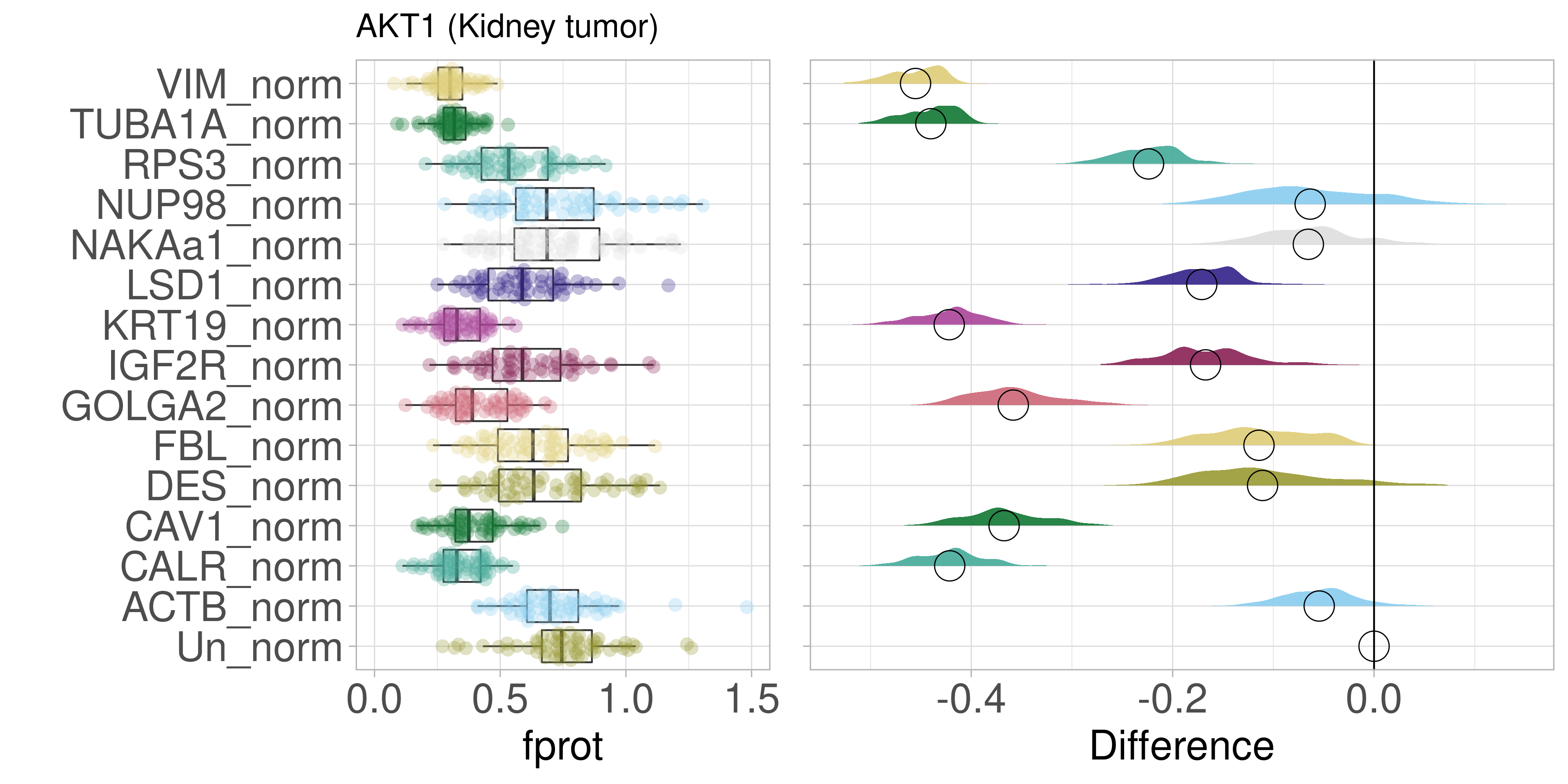

Supplement: Supplementary file 17 — Supplementary Material 17 [file 41598_2026_48754_MOESM17_ESM.zip › RPPA normalizations to cell markers/Kidney_plots/Oncoprotein_Kidney/AKT1_Kidney_T.png]

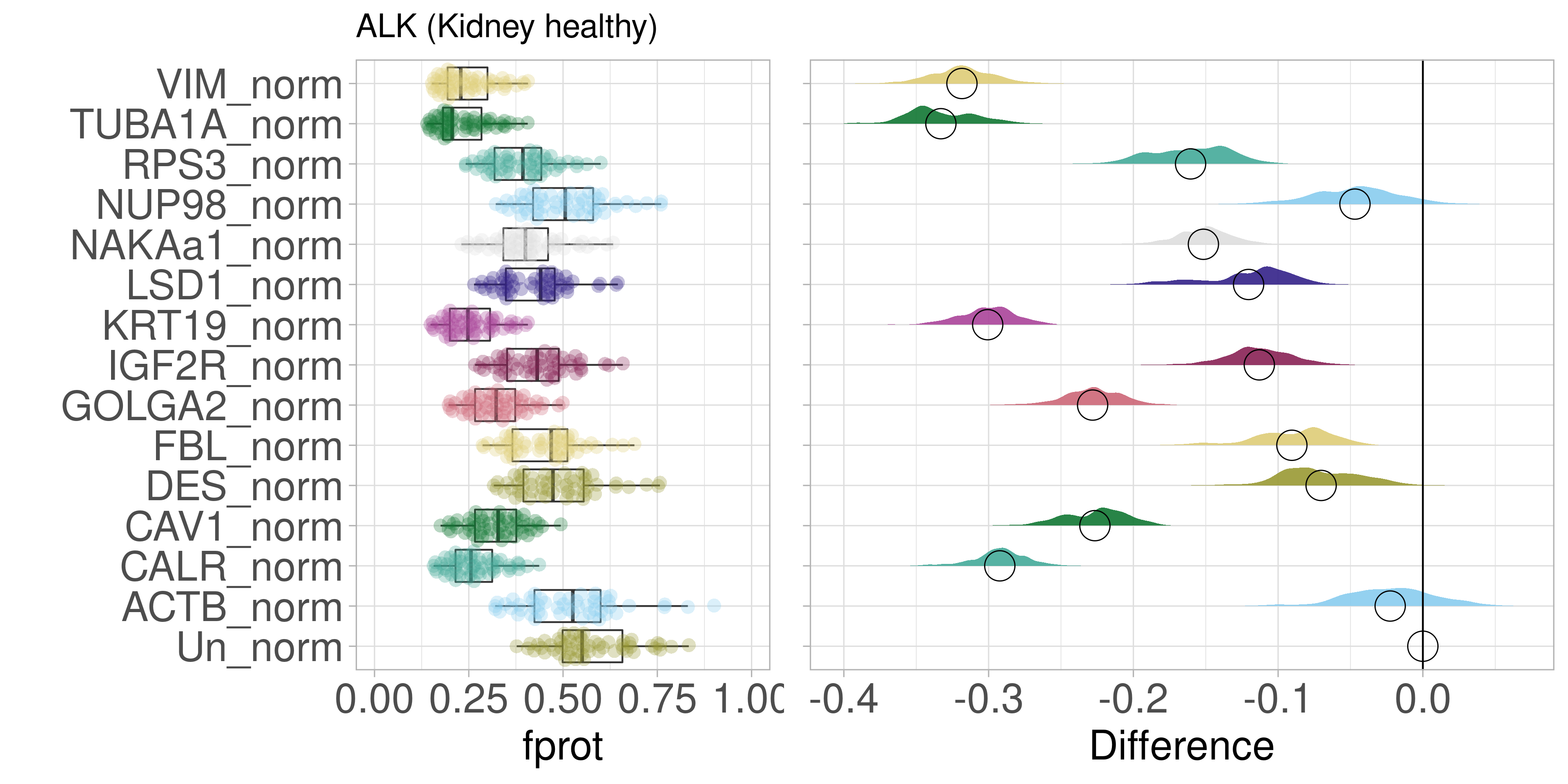

Supplement: Supplementary file 17 — Supplementary Material 17 [file 41598_2026_48754_MOESM17_ESM.zip › RPPA normalizations to cell markers/Kidney_plots/Oncoprotein_Kidney/ALK_Kidney_H.png]

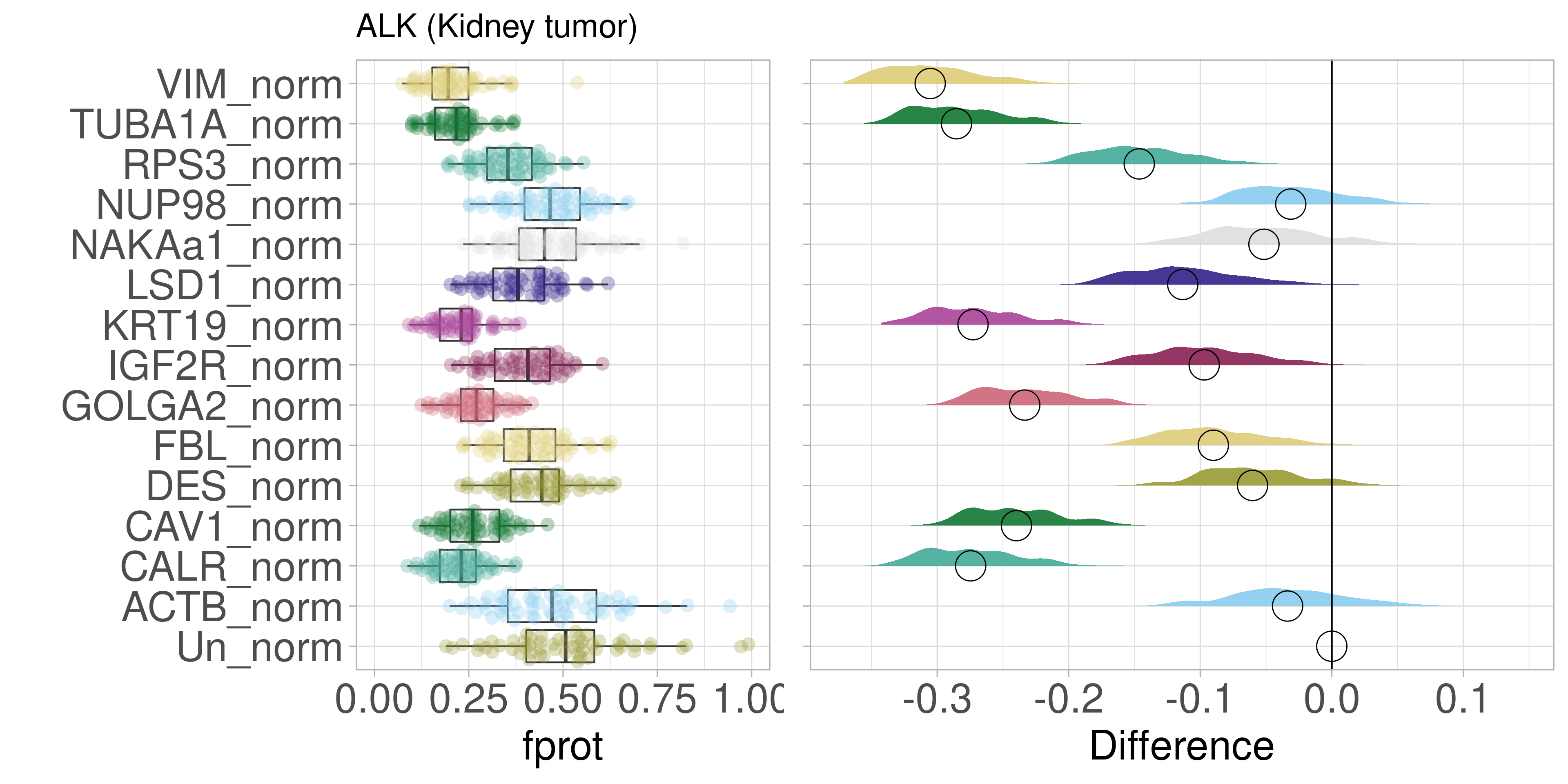

Supplement: Supplementary file 17 — Supplementary Material 17 [file 41598_2026_48754_MOESM17_ESM.zip › RPPA normalizations to cell markers/Kidney_plots/Oncoprotein_Kidney/ALK_Kidney_T.png]

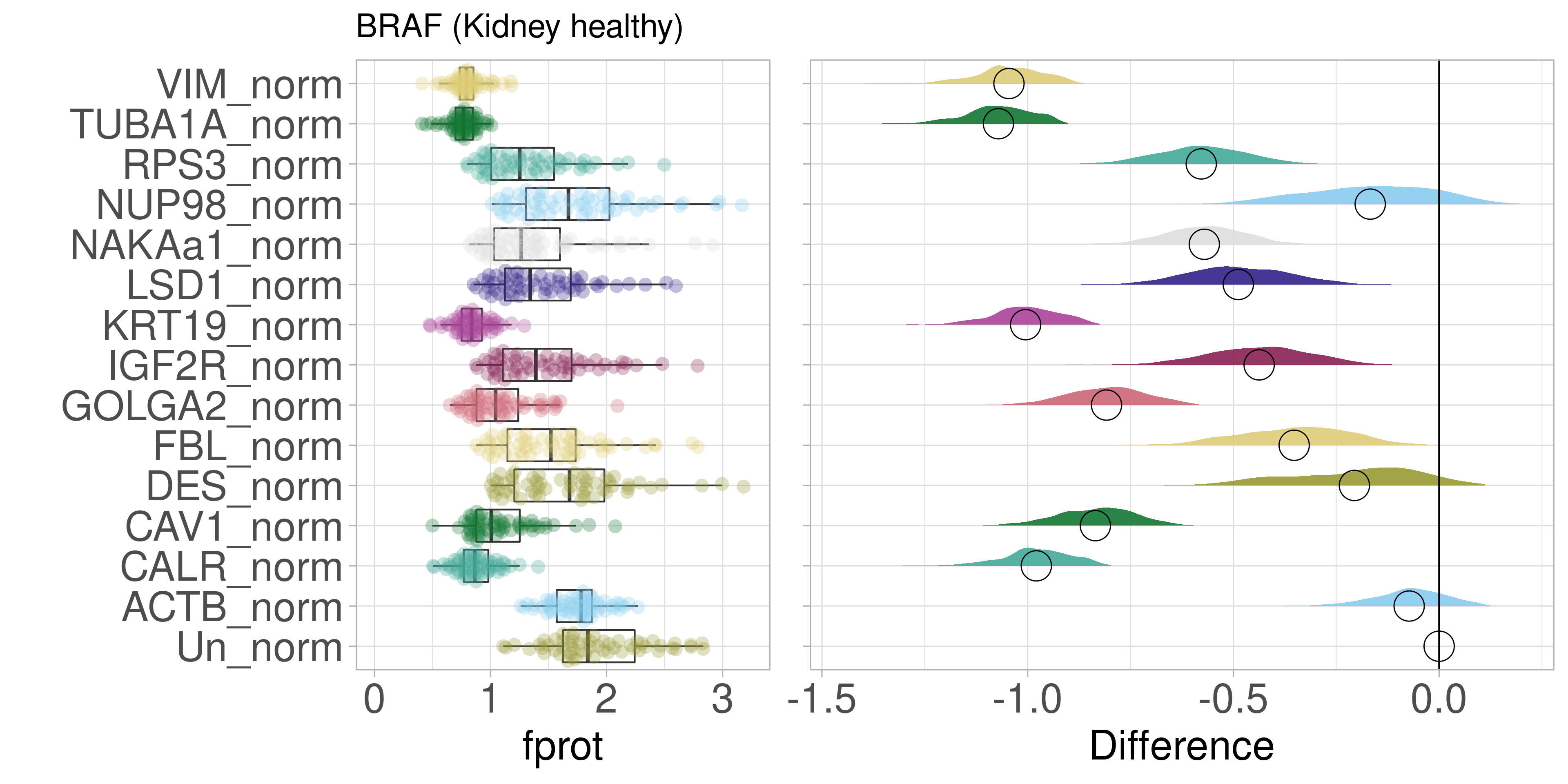

Supplement: Supplementary file 17 — Supplementary Material 17 [file 41598_2026_48754_MOESM17_ESM.zip › RPPA normalizations to cell markers/Kidney_plots/Oncoprotein_Kidney/BRAF_Kidney_H.png]

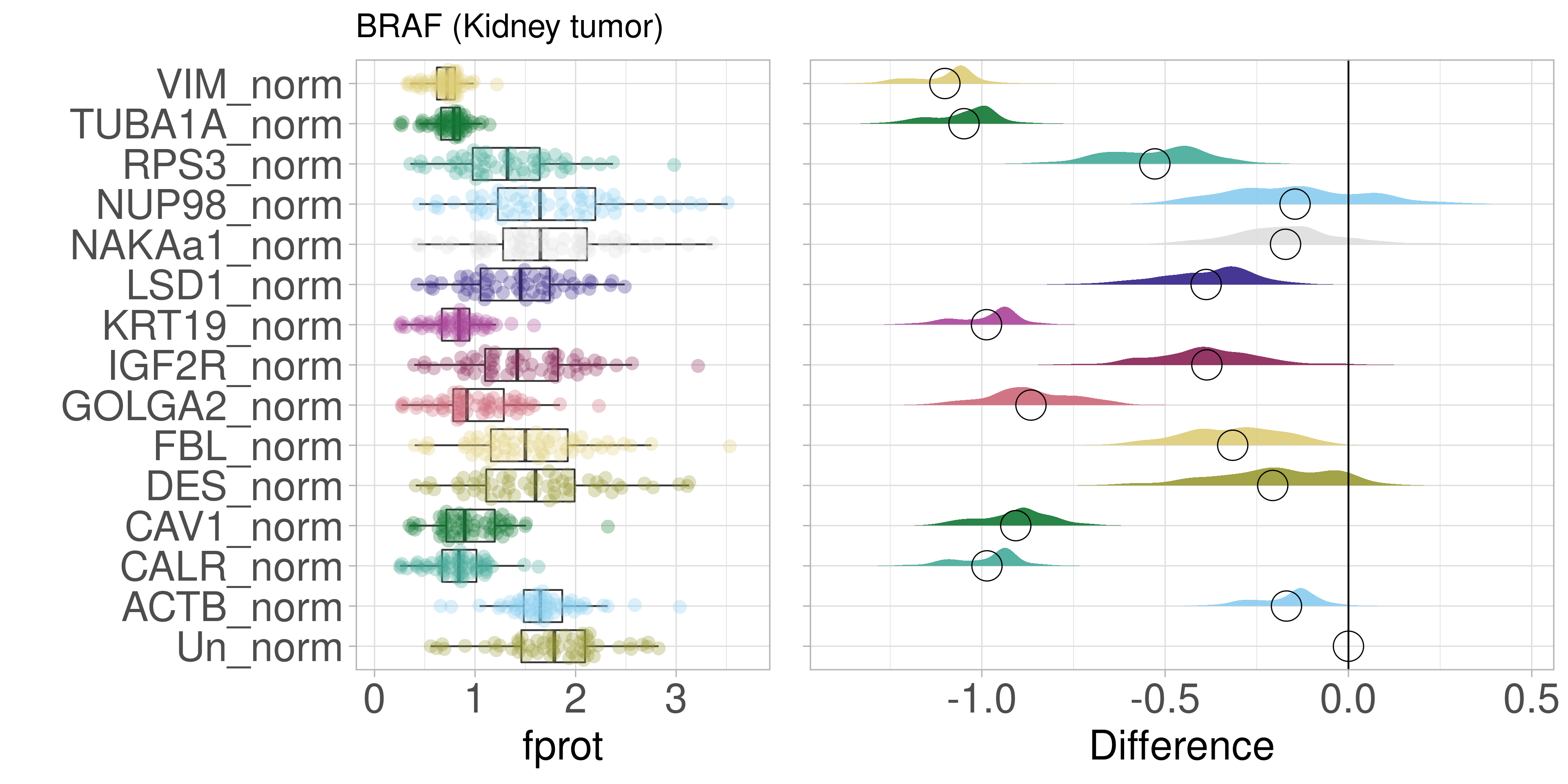

Supplement: Supplementary file 17 — Supplementary Material 17 [file 41598_2026_48754_MOESM17_ESM.zip › RPPA normalizations to cell markers/Kidney_plots/Oncoprotein_Kidney/BRAF_Kidney_T.png]

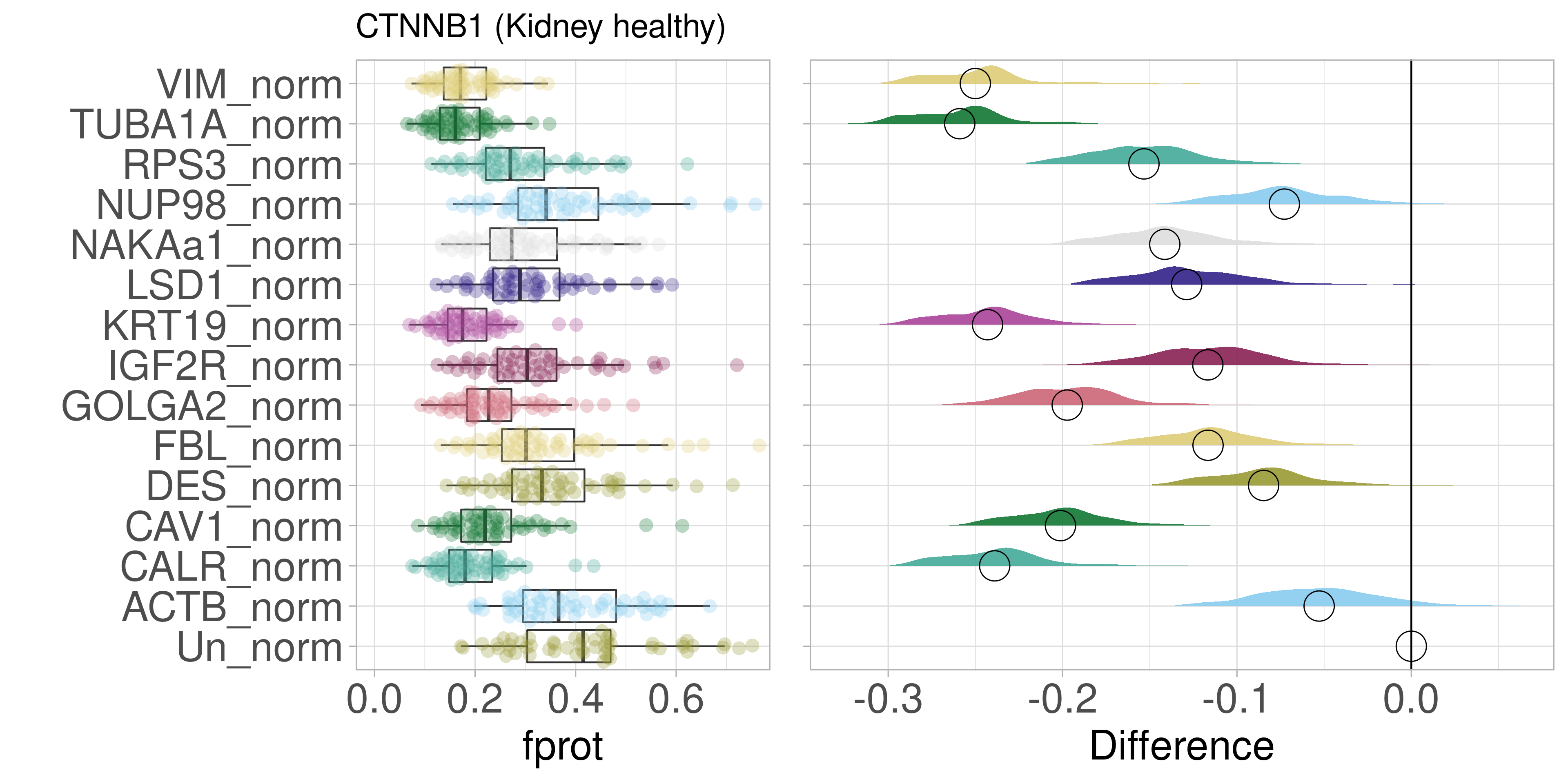

Supplement: Supplementary file 17 — Supplementary Material 17 [file 41598_2026_48754_MOESM17_ESM.zip › RPPA normalizations to cell markers/Kidney_plots/Oncoprotein_Kidney/CTNNB1_Kidney_H.png]

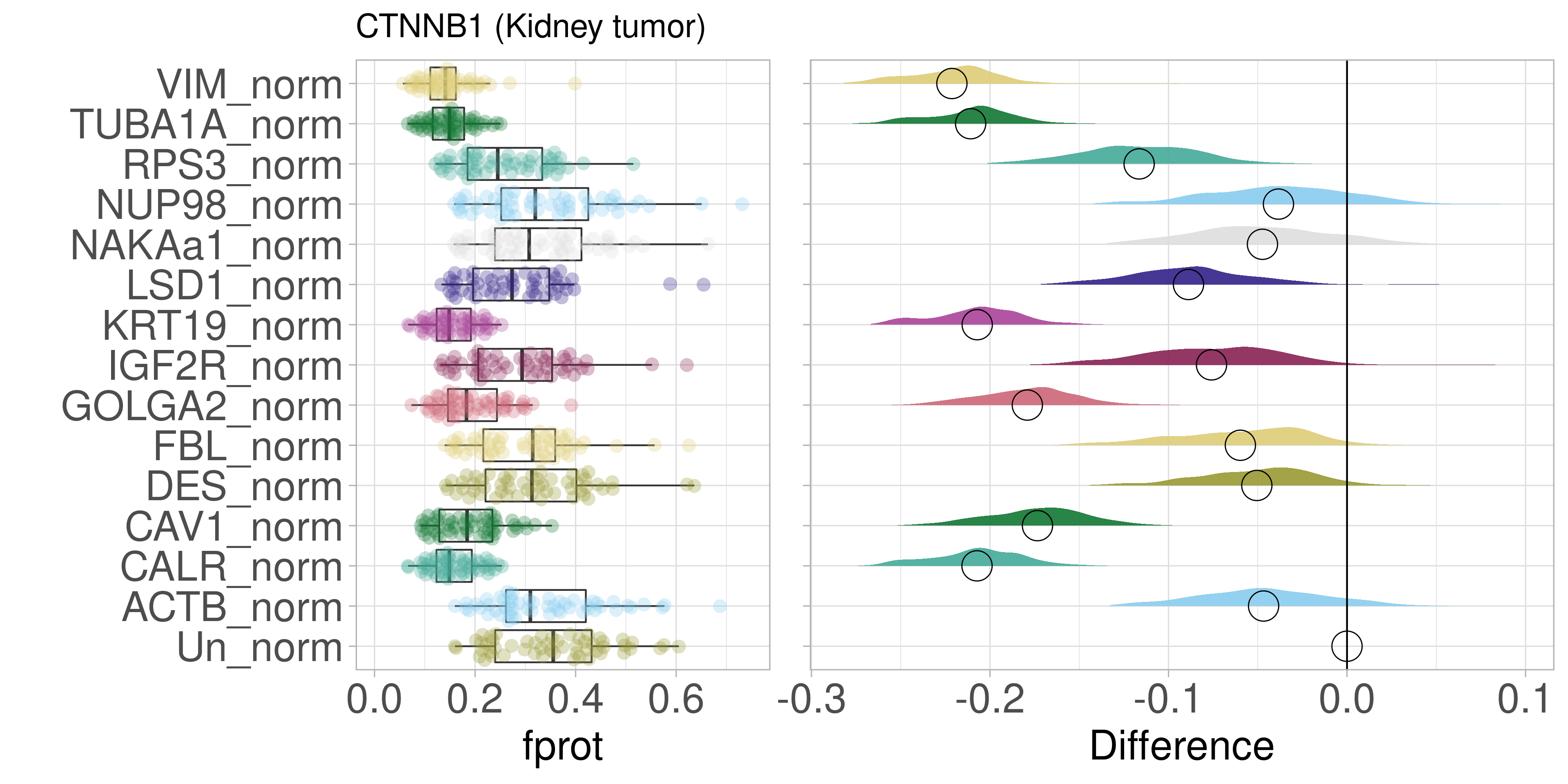

Supplement: Supplementary file 17 — Supplementary Material 17 [file 41598_2026_48754_MOESM17_ESM.zip › RPPA normalizations to cell markers/Kidney_plots/Oncoprotein_Kidney/CTNNB1_Kidney_T.png]

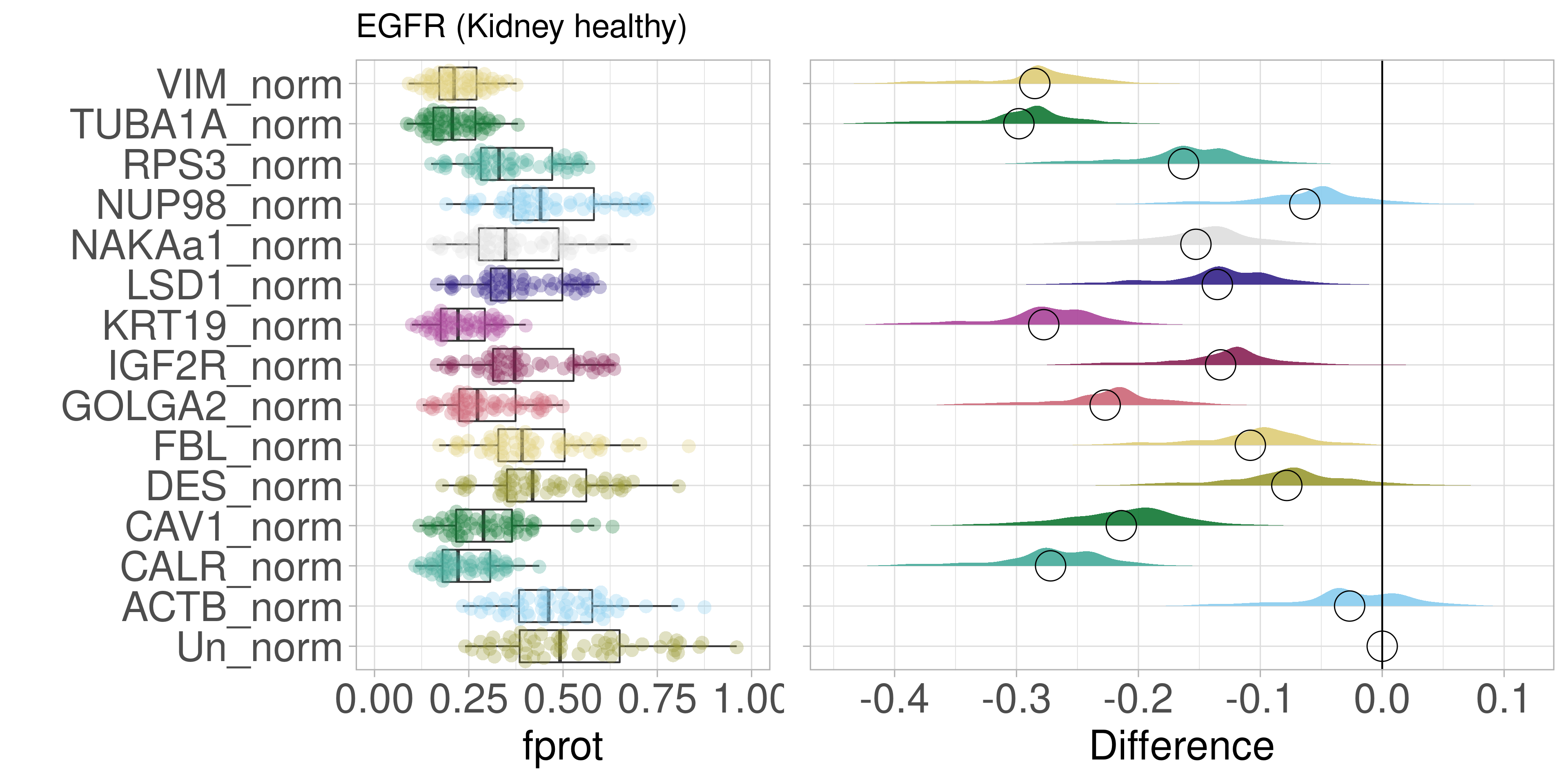

Supplement: Supplementary file 17 — Supplementary Material 17 [file 41598_2026_48754_MOESM17_ESM.zip › RPPA normalizations to cell markers/Kidney_plots/Oncoprotein_Kidney/EGFR_Kidney_H.png]

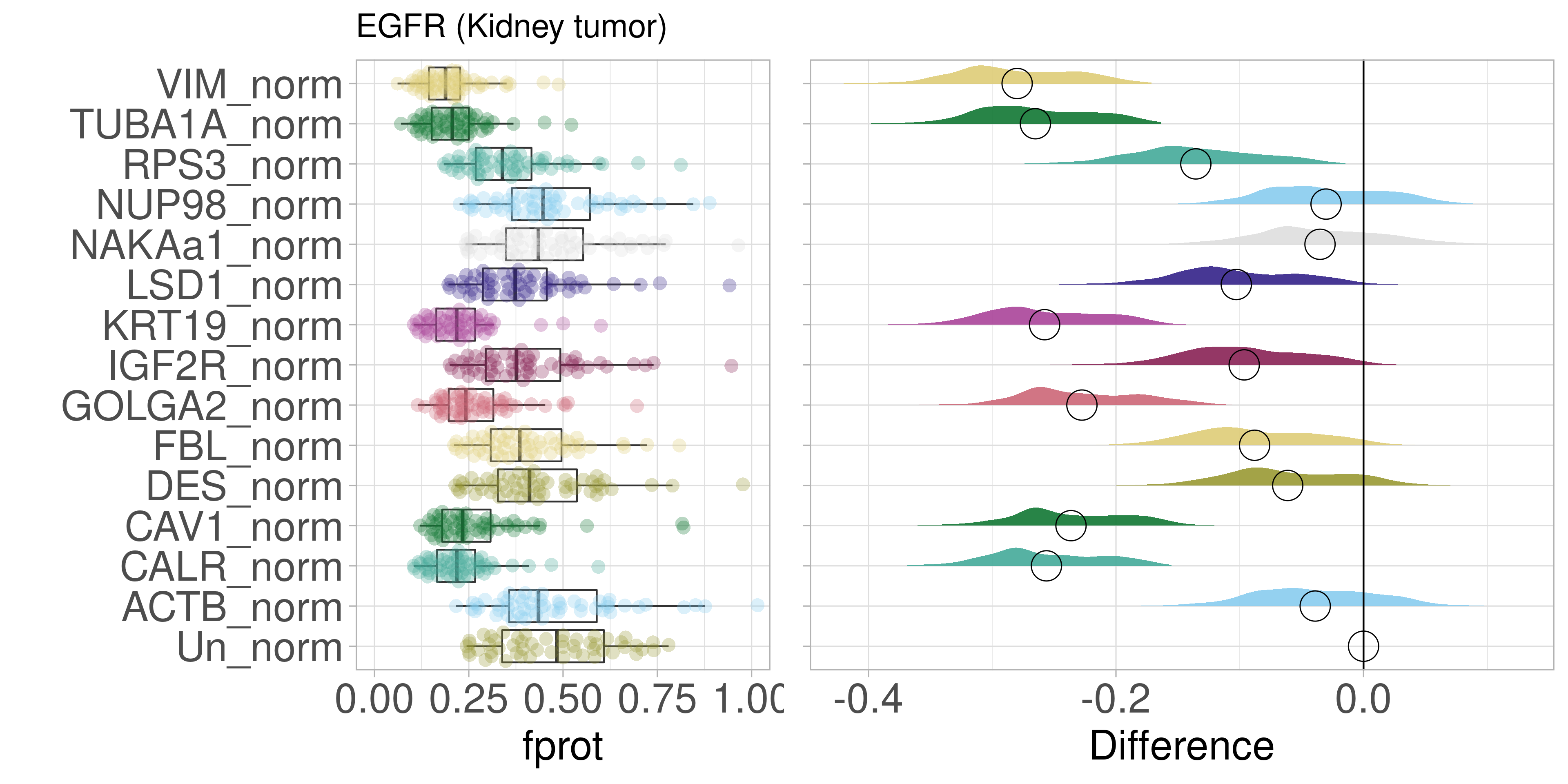

Supplement: Supplementary file 17 — Supplementary Material 17 [file 41598_2026_48754_MOESM17_ESM.zip › RPPA normalizations to cell markers/Kidney_plots/Oncoprotein_Kidney/EGFR_Kidney_T.png]

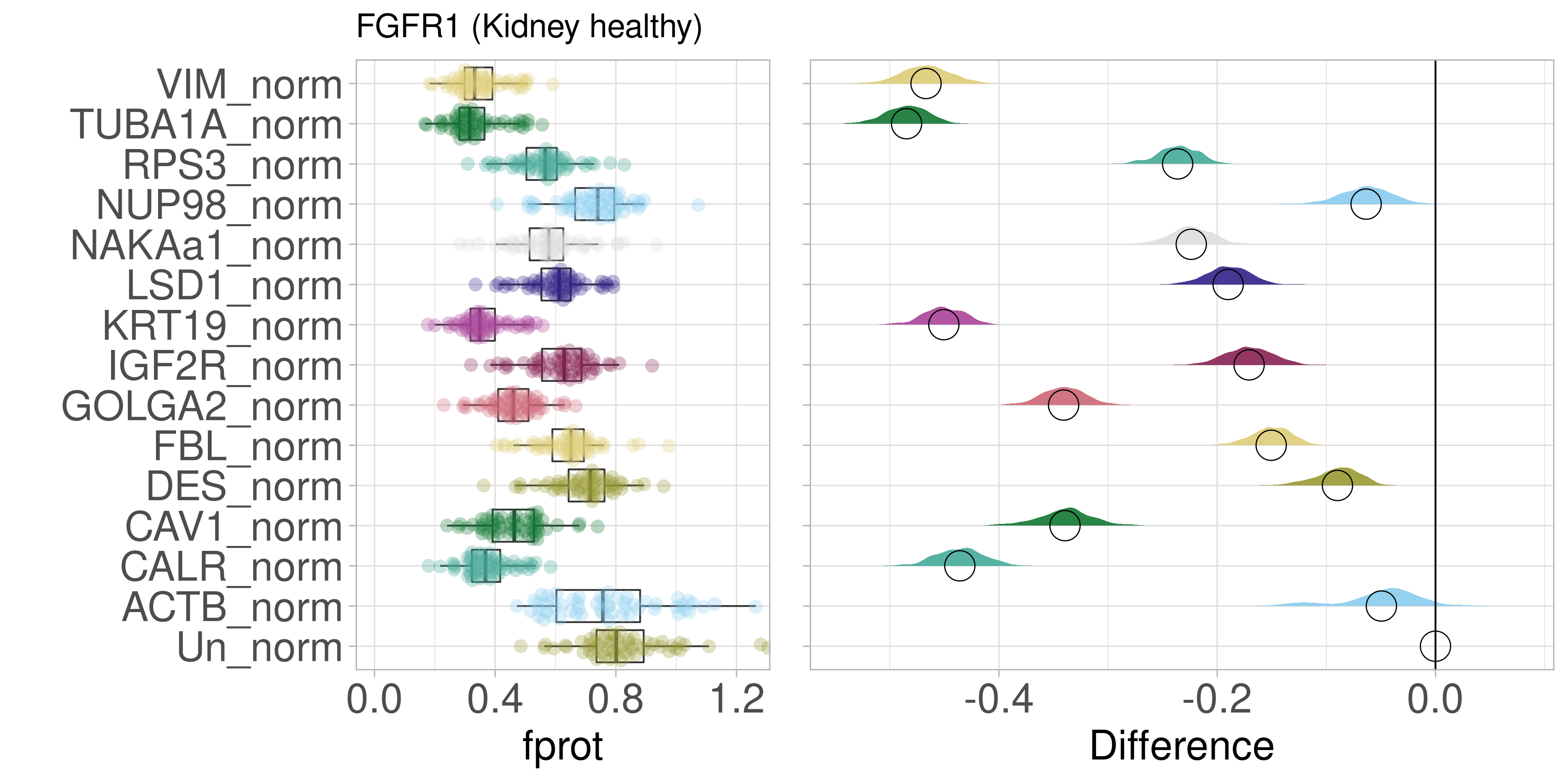

Supplement: Supplementary file 17 — Supplementary Material 17 [file 41598_2026_48754_MOESM17_ESM.zip › RPPA normalizations to cell markers/Kidney_plots/Oncoprotein_Kidney/FGFR1_Kidney_H.png]

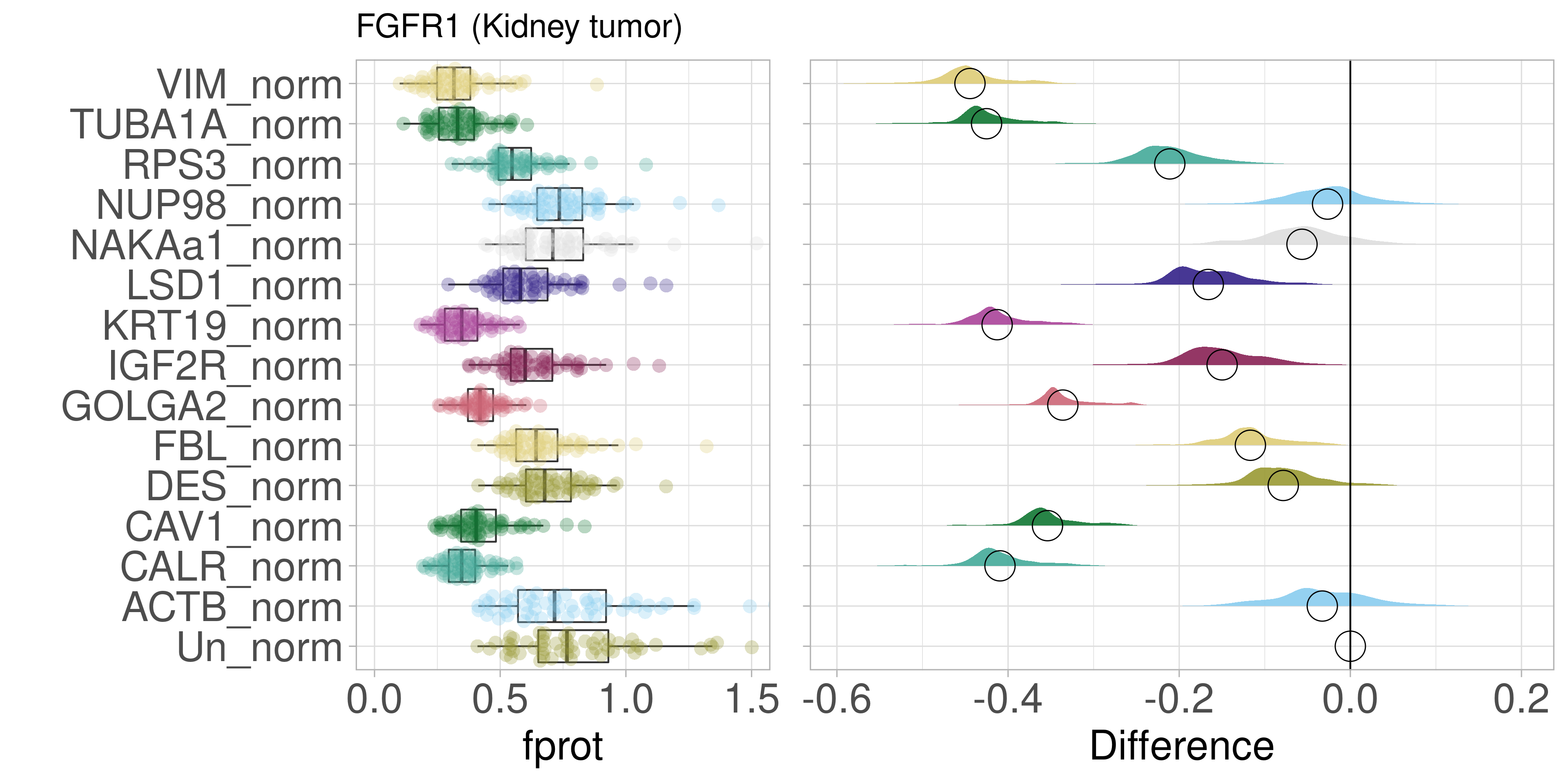

Supplement: Supplementary file 17 — Supplementary Material 17 [file 41598_2026_48754_MOESM17_ESM.zip › RPPA normalizations to cell markers/Kidney_plots/Oncoprotein_Kidney/FGFR1_Kidney_T.png]

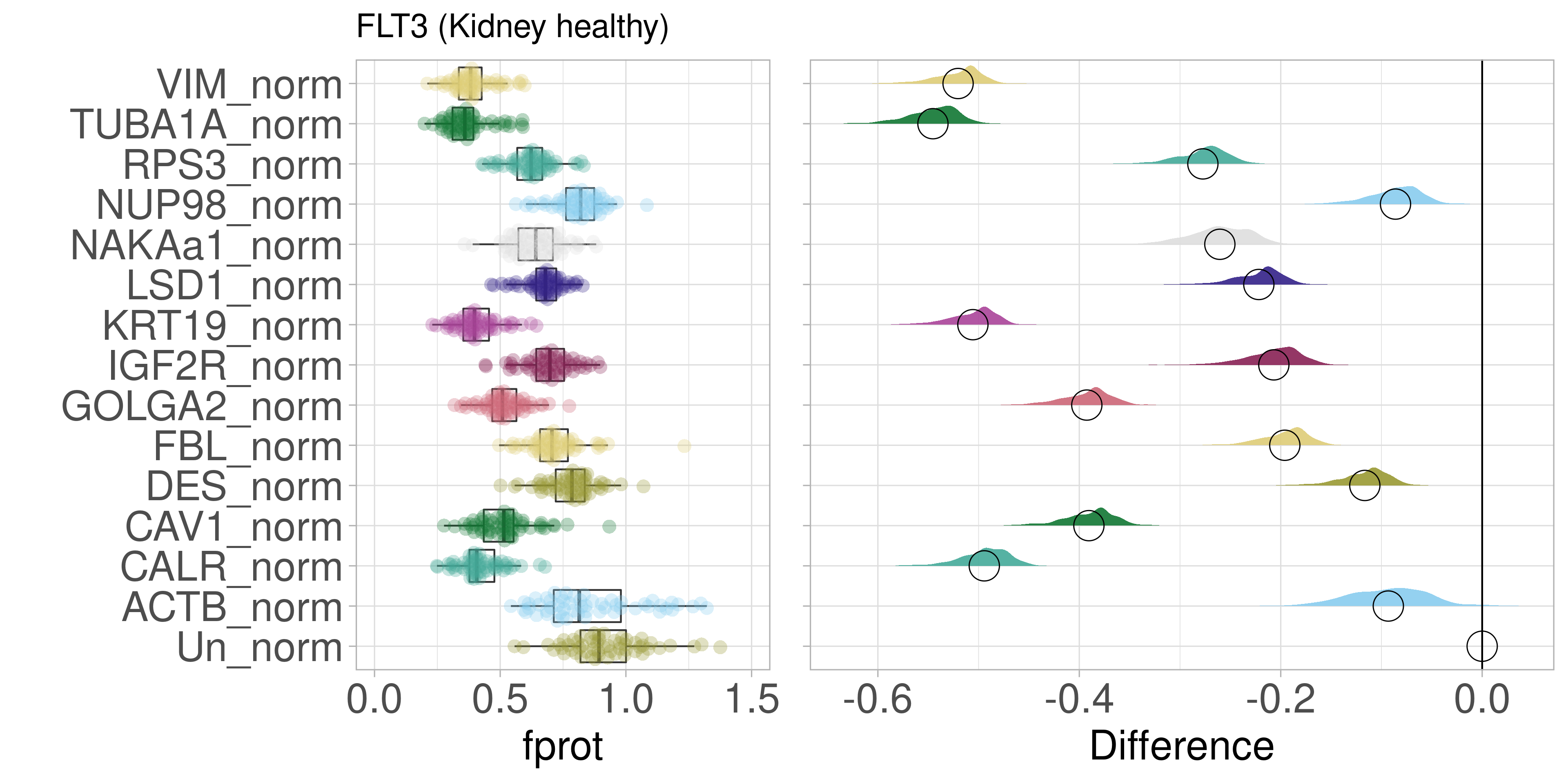

Supplement: Supplementary file 17 — Supplementary Material 17 [file 41598_2026_48754_MOESM17_ESM.zip › RPPA normalizations to cell markers/Kidney_plots/Oncoprotein_Kidney/FLT3_Kidney_H.png]

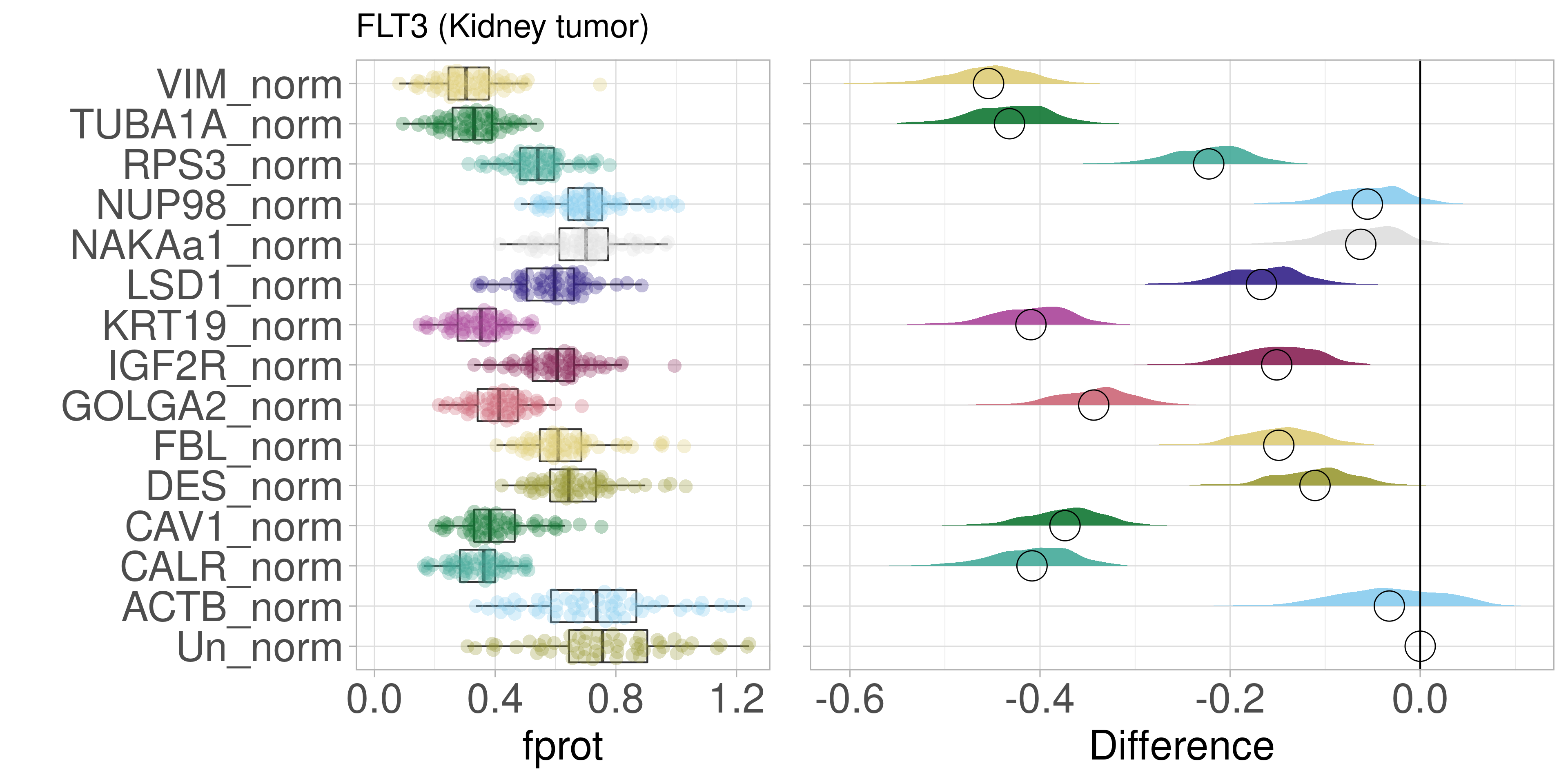

Supplement: Supplementary file 17 — Supplementary Material 17 [file 41598_2026_48754_MOESM17_ESM.zip › RPPA normalizations to cell markers/Kidney_plots/Oncoprotein_Kidney/FLT3_Kidney_T.png]

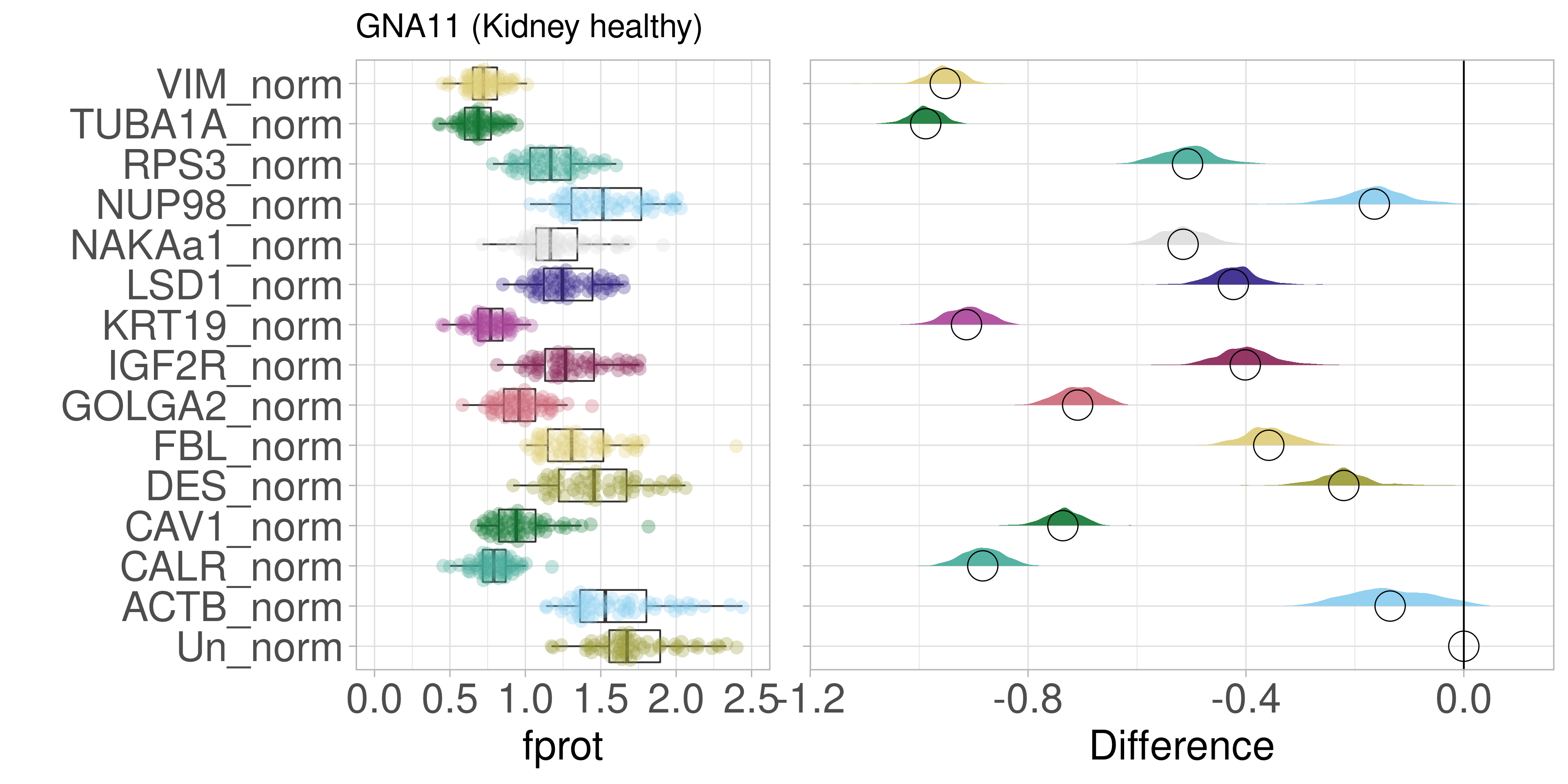

Supplement: Supplementary file 17 — Supplementary Material 17 [file 41598_2026_48754_MOESM17_ESM.zip › RPPA normalizations to cell markers/Kidney_plots/Oncoprotein_Kidney/GNA11_Kidney_H.png]

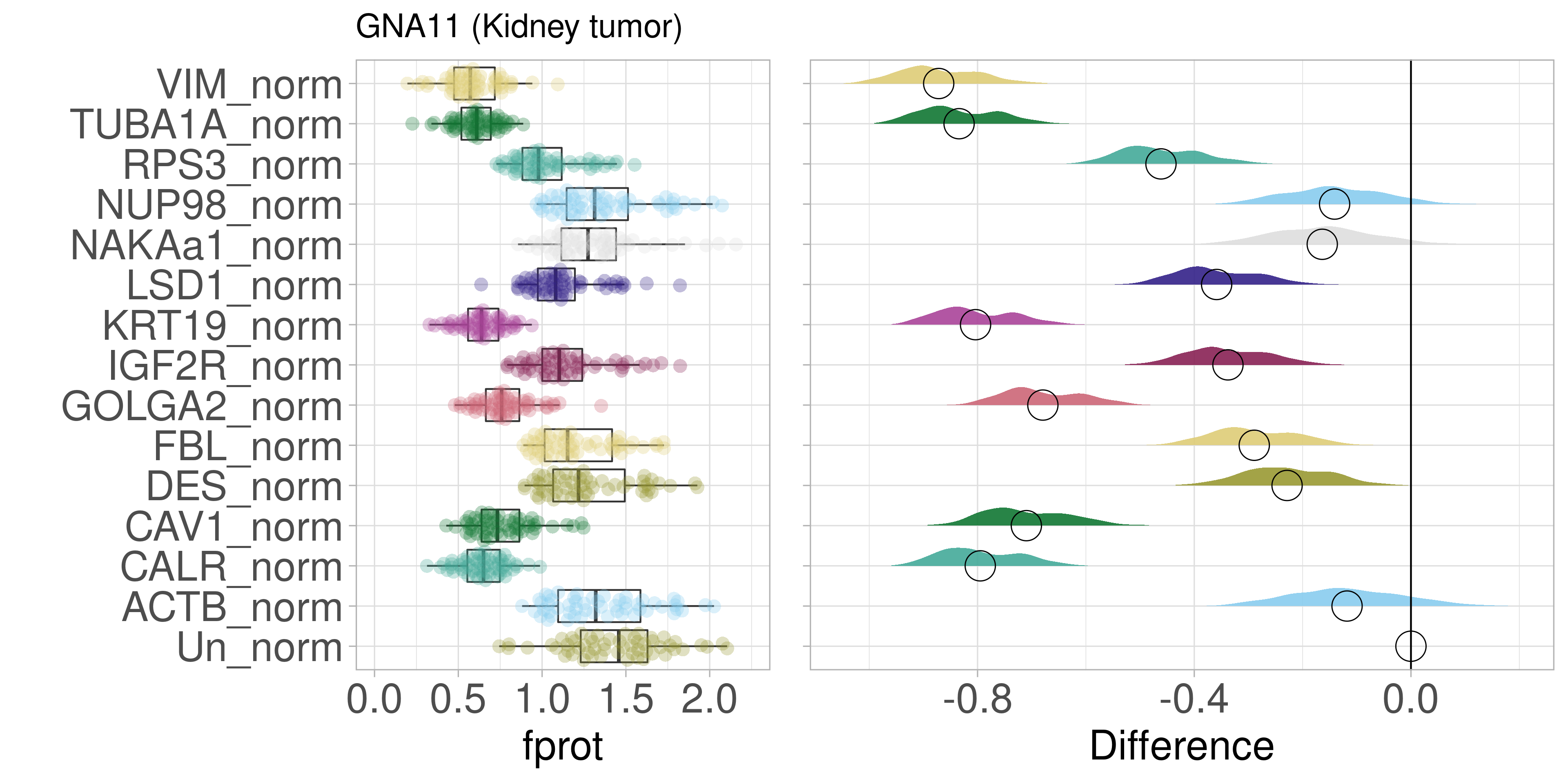

Supplement: Supplementary file 17 — Supplementary Material 17 [file 41598_2026_48754_MOESM17_ESM.zip › RPPA normalizations to cell markers/Kidney_plots/Oncoprotein_Kidney/GNA11_Kidney_T.png]

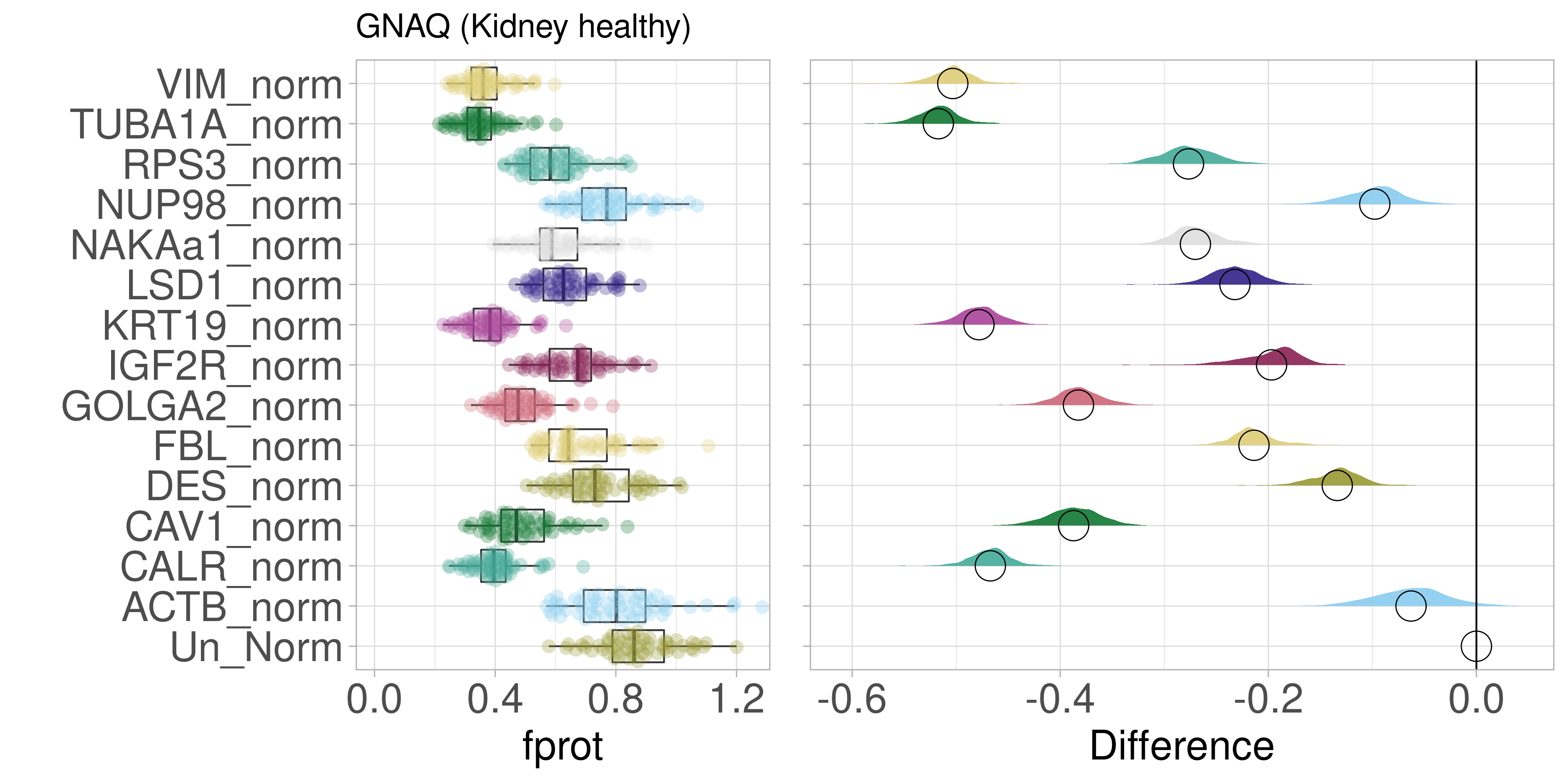

Supplement: Supplementary file 17 — Supplementary Material 17 [file 41598_2026_48754_MOESM17_ESM.zip › RPPA normalizations to cell markers/Kidney_plots/Oncoprotein_Kidney/GNAQ_Kidney_H.png]

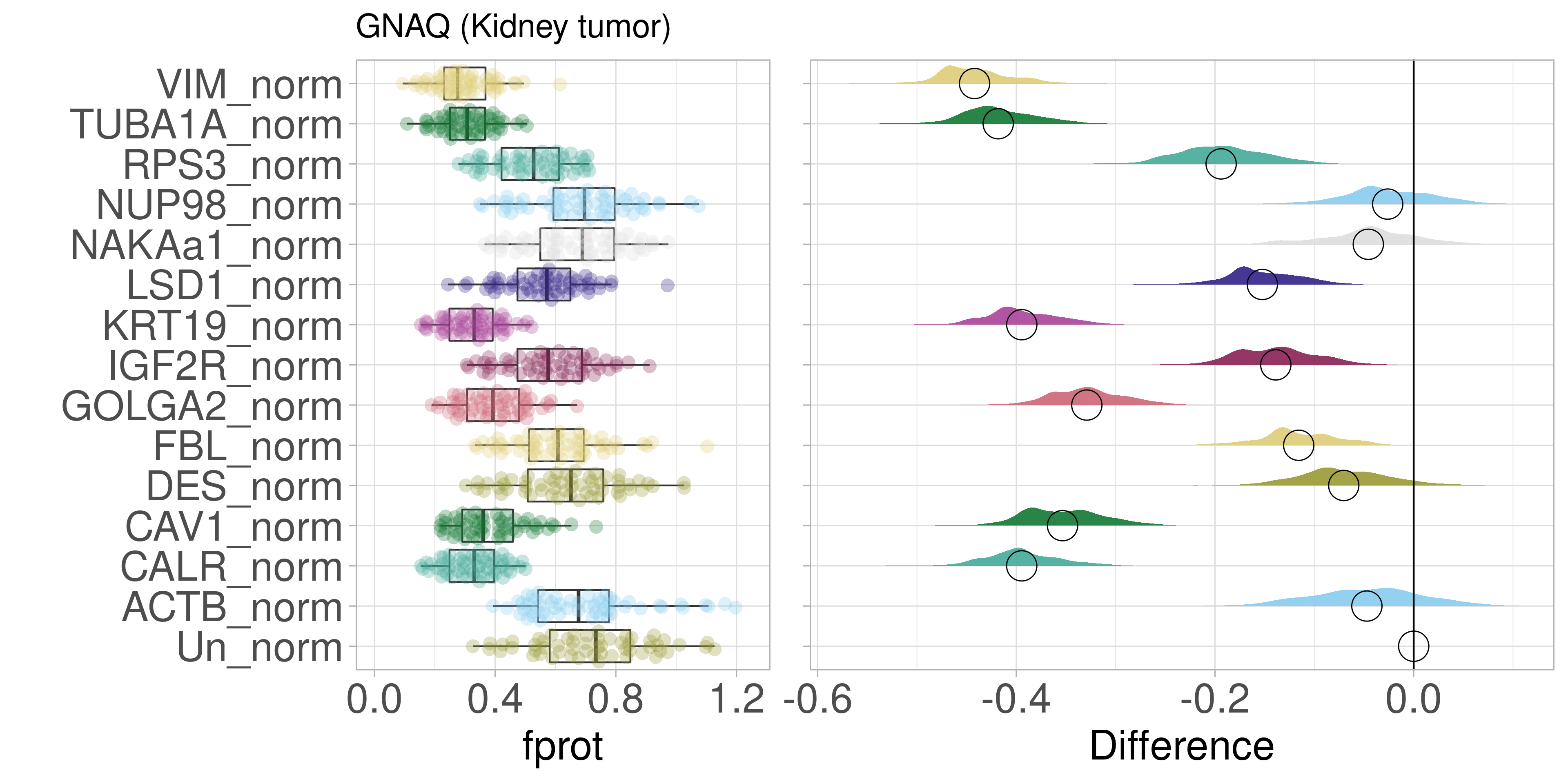

Supplement: Supplementary file 17 — Supplementary Material 17 [file 41598_2026_48754_MOESM17_ESM.zip › RPPA normalizations to cell markers/Kidney_plots/Oncoprotein_Kidney/GNAQ_Kidney_T.png]

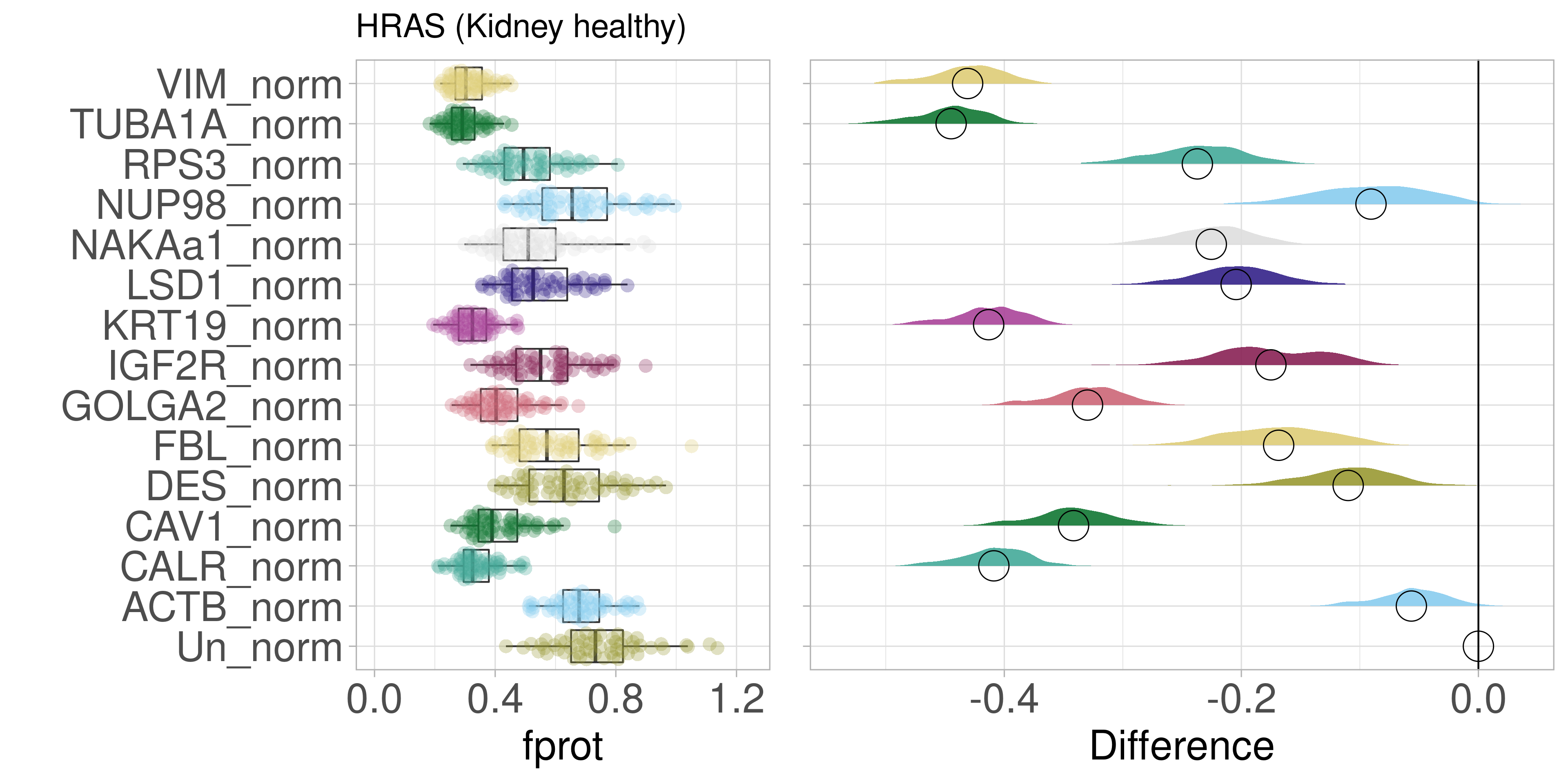

Supplement: Supplementary file 17 — Supplementary Material 17 [file 41598_2026_48754_MOESM17_ESM.zip › RPPA normalizations to cell markers/Kidney_plots/Oncoprotein_Kidney/HRAS_Kidney_H.png]

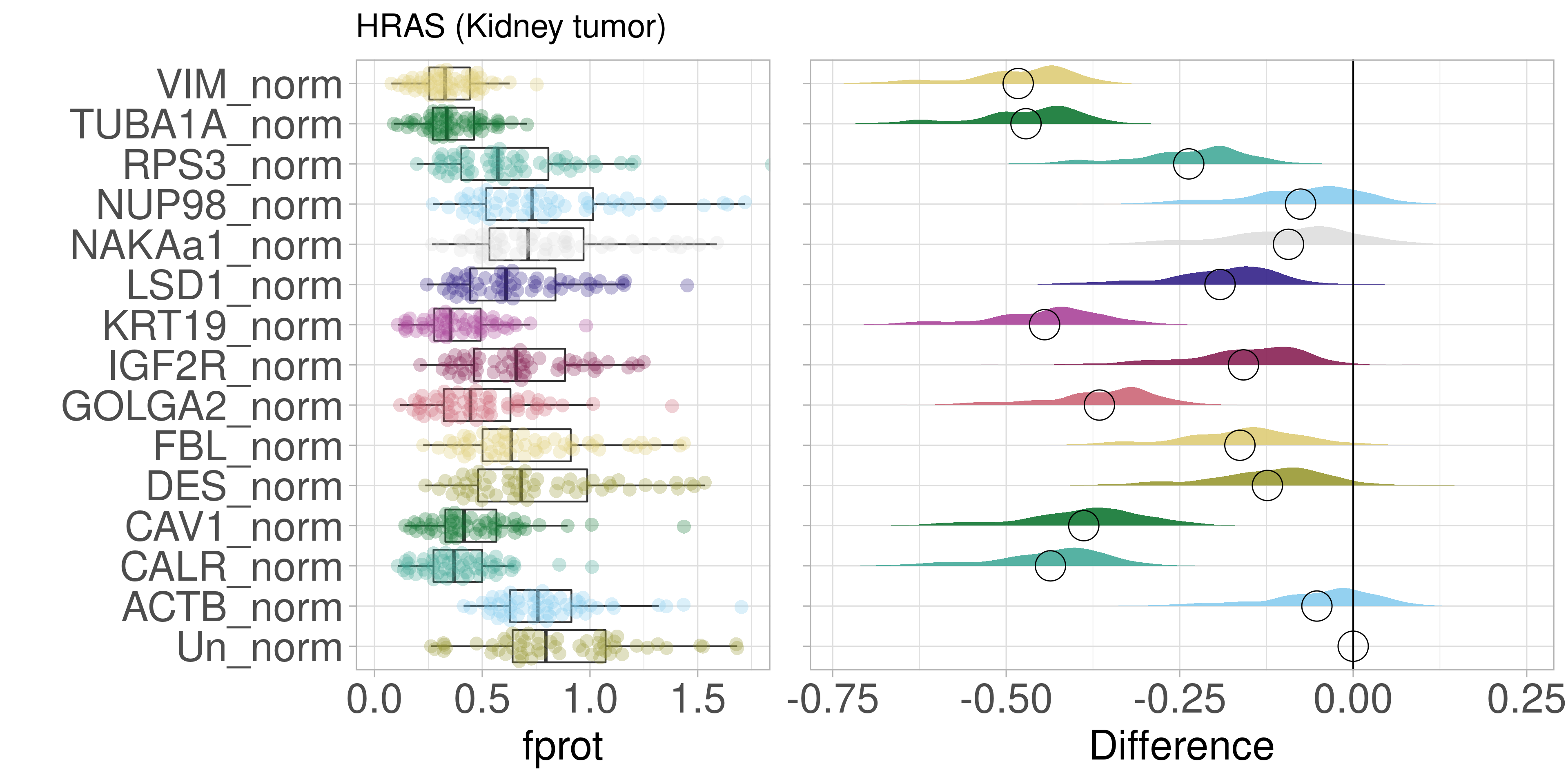

Supplement: Supplementary file 17 — Supplementary Material 17 [file 41598_2026_48754_MOESM17_ESM.zip › RPPA normalizations to cell markers/Kidney_plots/Oncoprotein_Kidney/HRAS_Kidney_T.png]

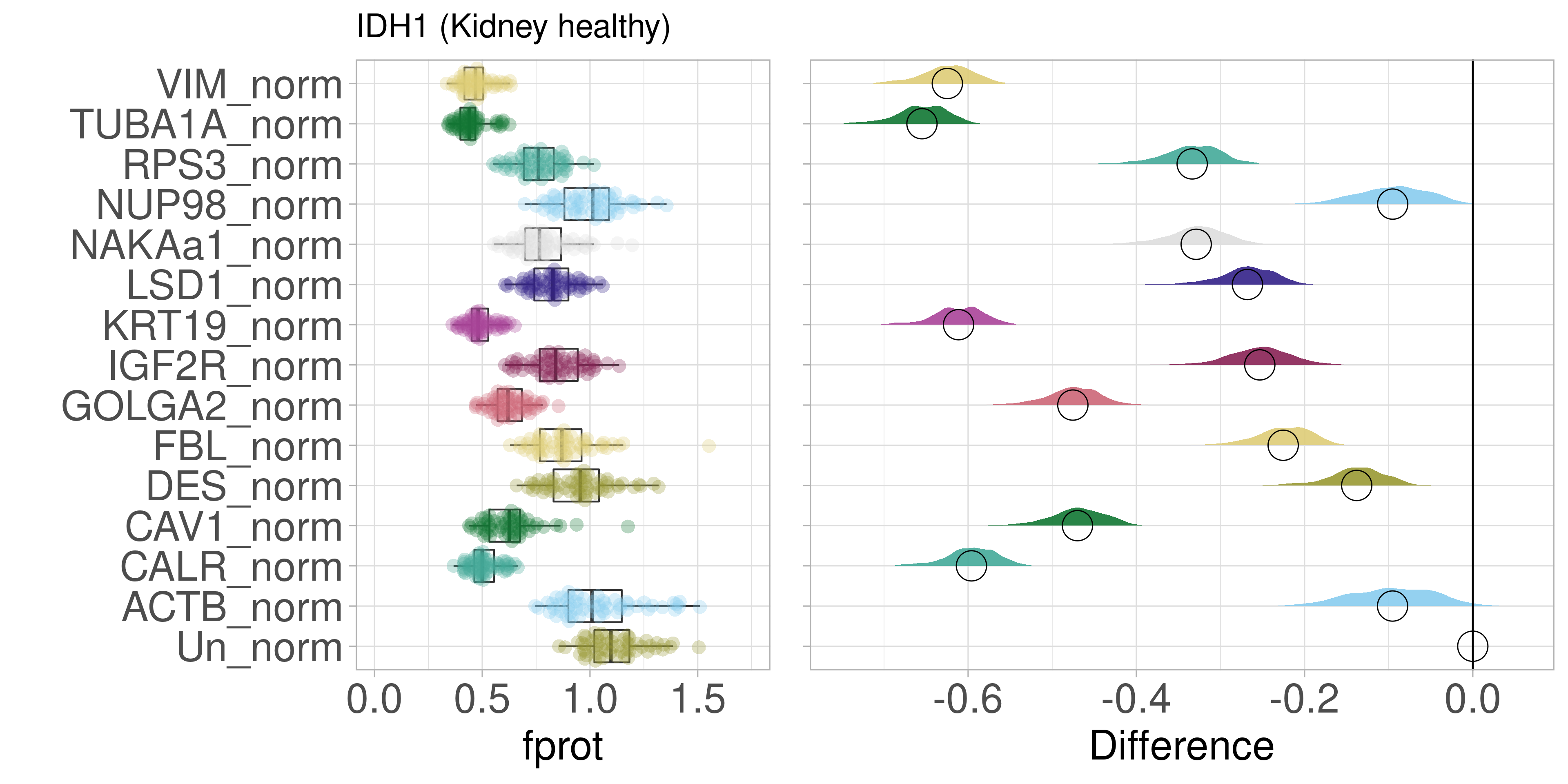

Supplement: Supplementary file 17 — Supplementary Material 17 [file 41598_2026_48754_MOESM17_ESM.zip › RPPA normalizations to cell markers/Kidney_plots/Oncoprotein_Kidney/IDH1_Kidney_H.png]

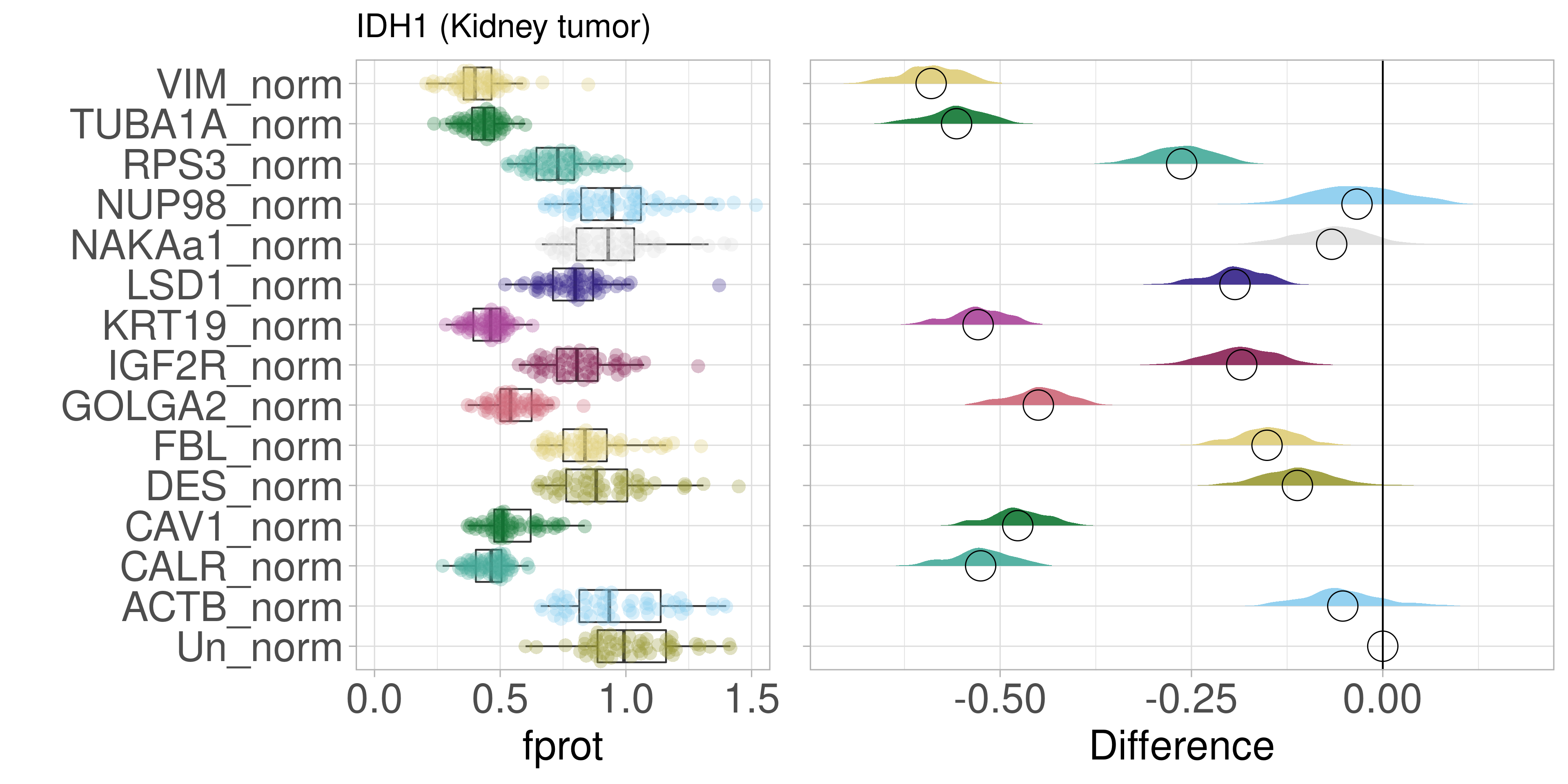

Supplement: Supplementary file 17 — Supplementary Material 17 [file 41598_2026_48754_MOESM17_ESM.zip › RPPA normalizations to cell markers/Kidney_plots/Oncoprotein_Kidney/IDH1_Kidney_T.png]

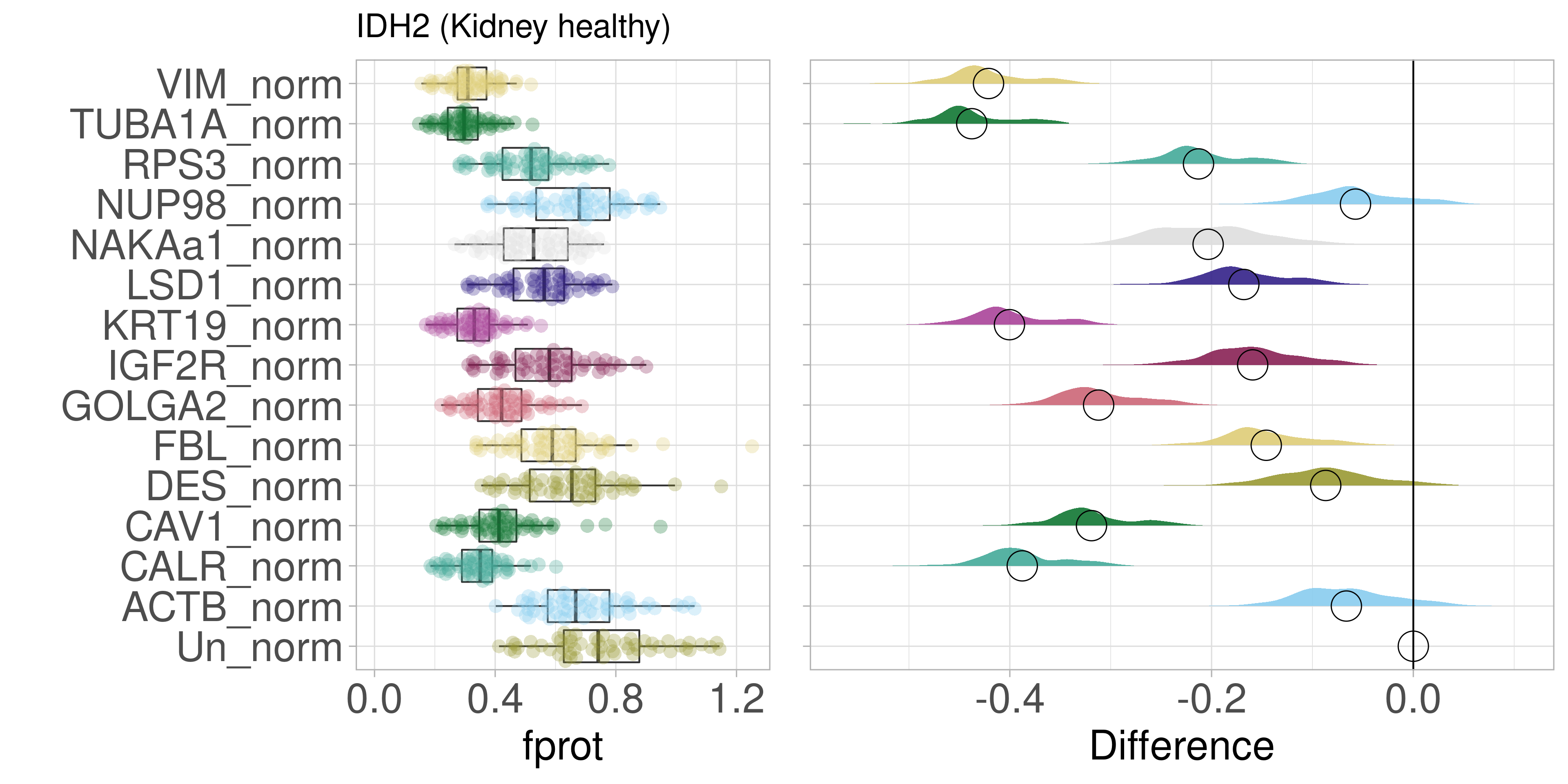

Supplement: Supplementary file 17 — Supplementary Material 17 [file 41598_2026_48754_MOESM17_ESM.zip › RPPA normalizations to cell markers/Kidney_plots/Oncoprotein_Kidney/IDH2_Kidney_H.png]

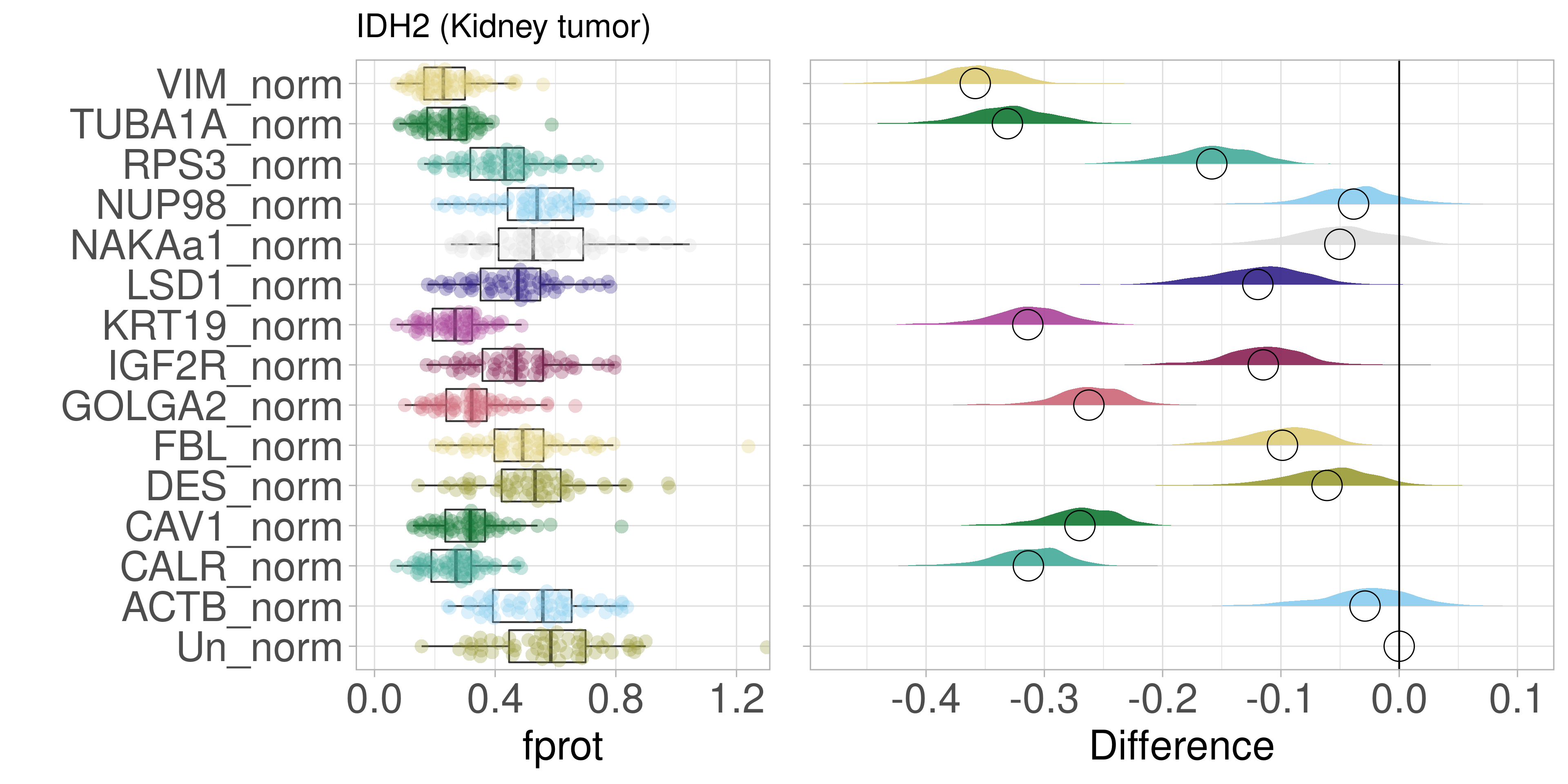

Supplement: Supplementary file 17 — Supplementary Material 17 [file 41598_2026_48754_MOESM17_ESM.zip › RPPA normalizations to cell markers/Kidney_plots/Oncoprotein_Kidney/IDH2_Kidney_T.png]

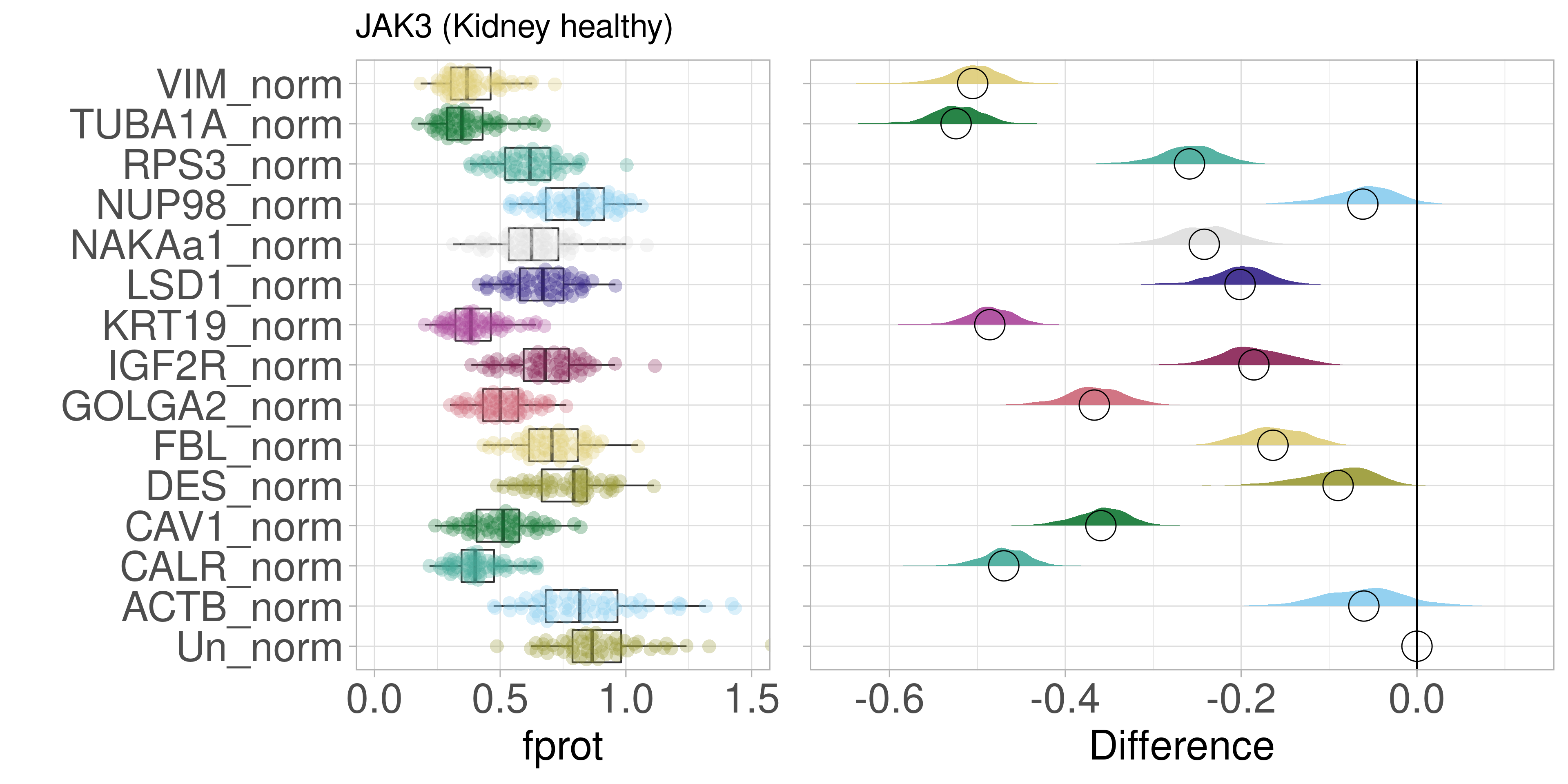

Supplement: Supplementary file 17 — Supplementary Material 17 [file 41598_2026_48754_MOESM17_ESM.zip › RPPA normalizations to cell markers/Kidney_plots/Oncoprotein_Kidney/JAK3_Kidney_H.png]

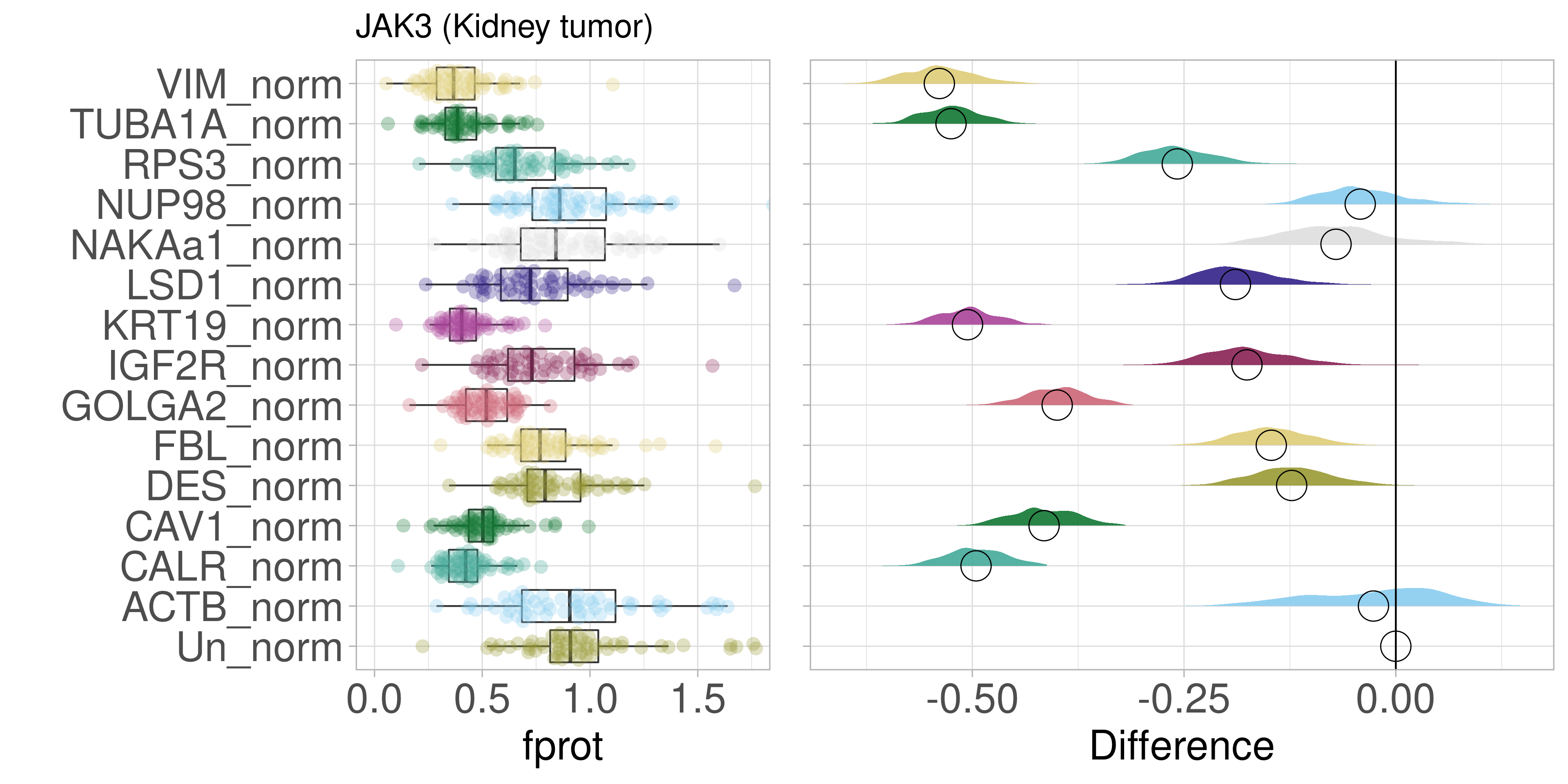

Supplement: Supplementary file 17 — Supplementary Material 17 [file 41598_2026_48754_MOESM17_ESM.zip › RPPA normalizations to cell markers/Kidney_plots/Oncoprotein_Kidney/JAK3_Kidney_T.png]

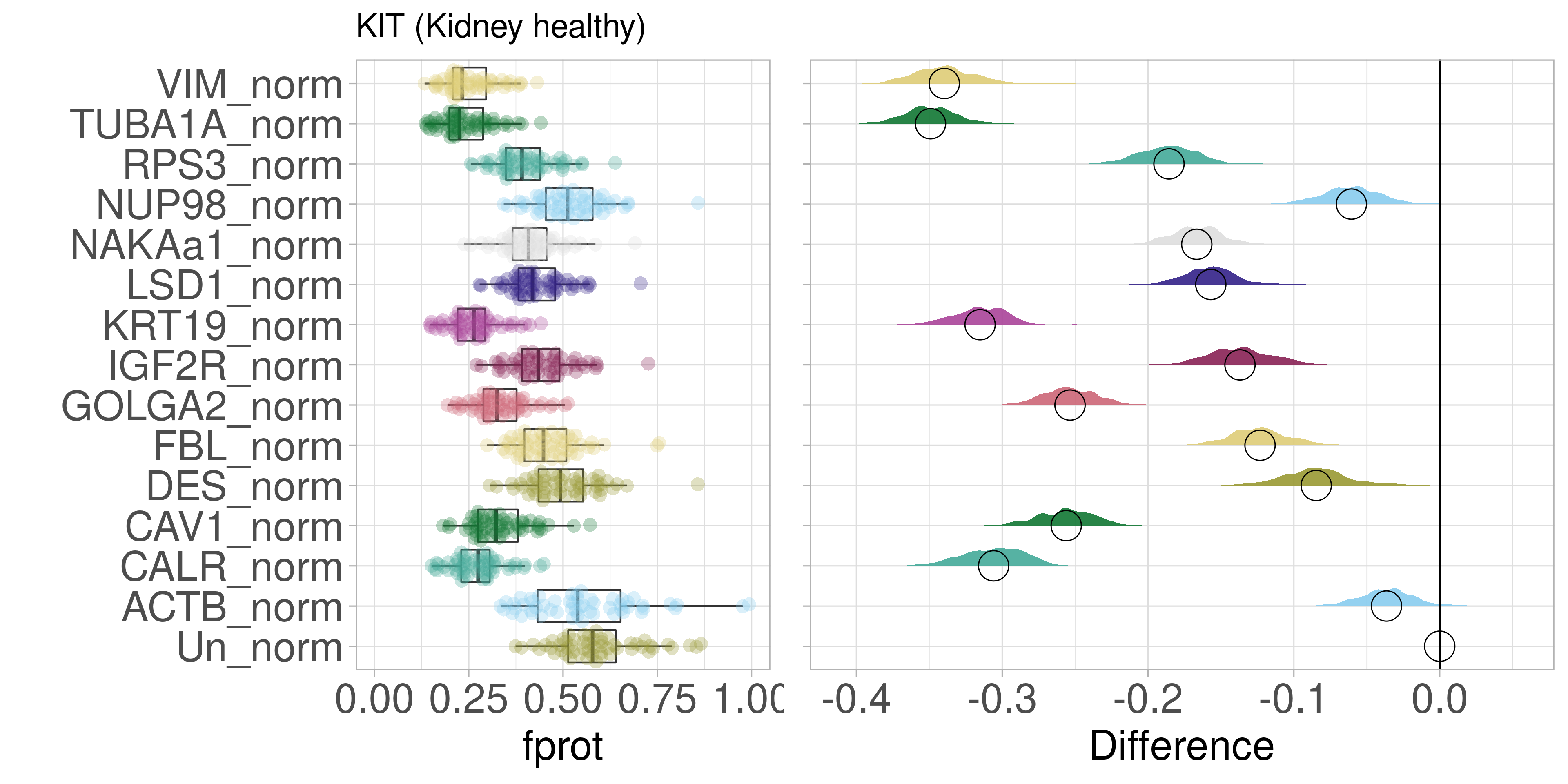

Supplement: Supplementary file 17 — Supplementary Material 17 [file 41598_2026_48754_MOESM17_ESM.zip › RPPA normalizations to cell markers/Kidney_plots/Oncoprotein_Kidney/KIT_Kidney_H.png]

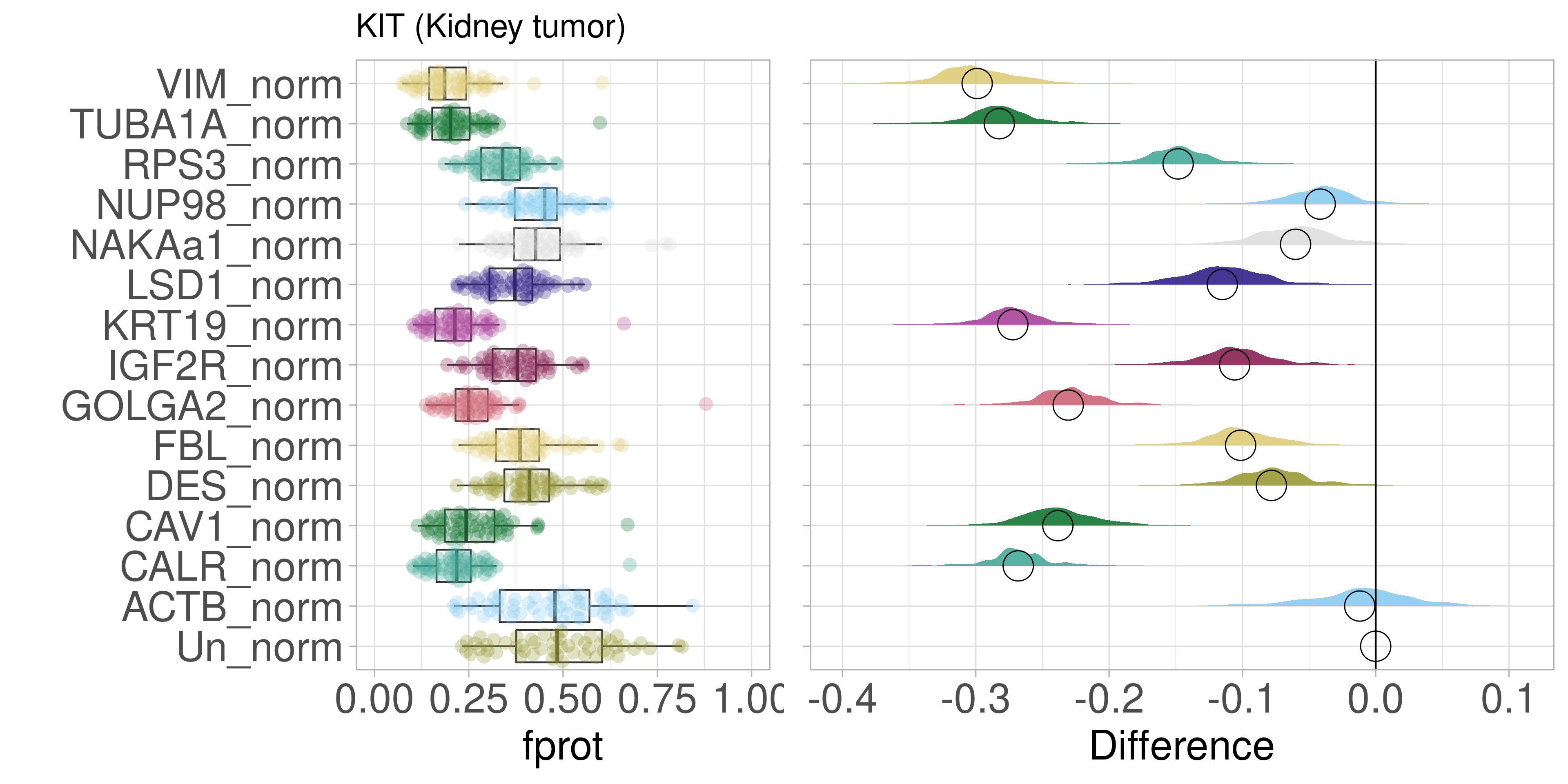

Supplement: Supplementary file 17 — Supplementary Material 17 [file 41598_2026_48754_MOESM17_ESM.zip › RPPA normalizations to cell markers/Kidney_plots/Oncoprotein_Kidney/KIT_Kidney_T.png]

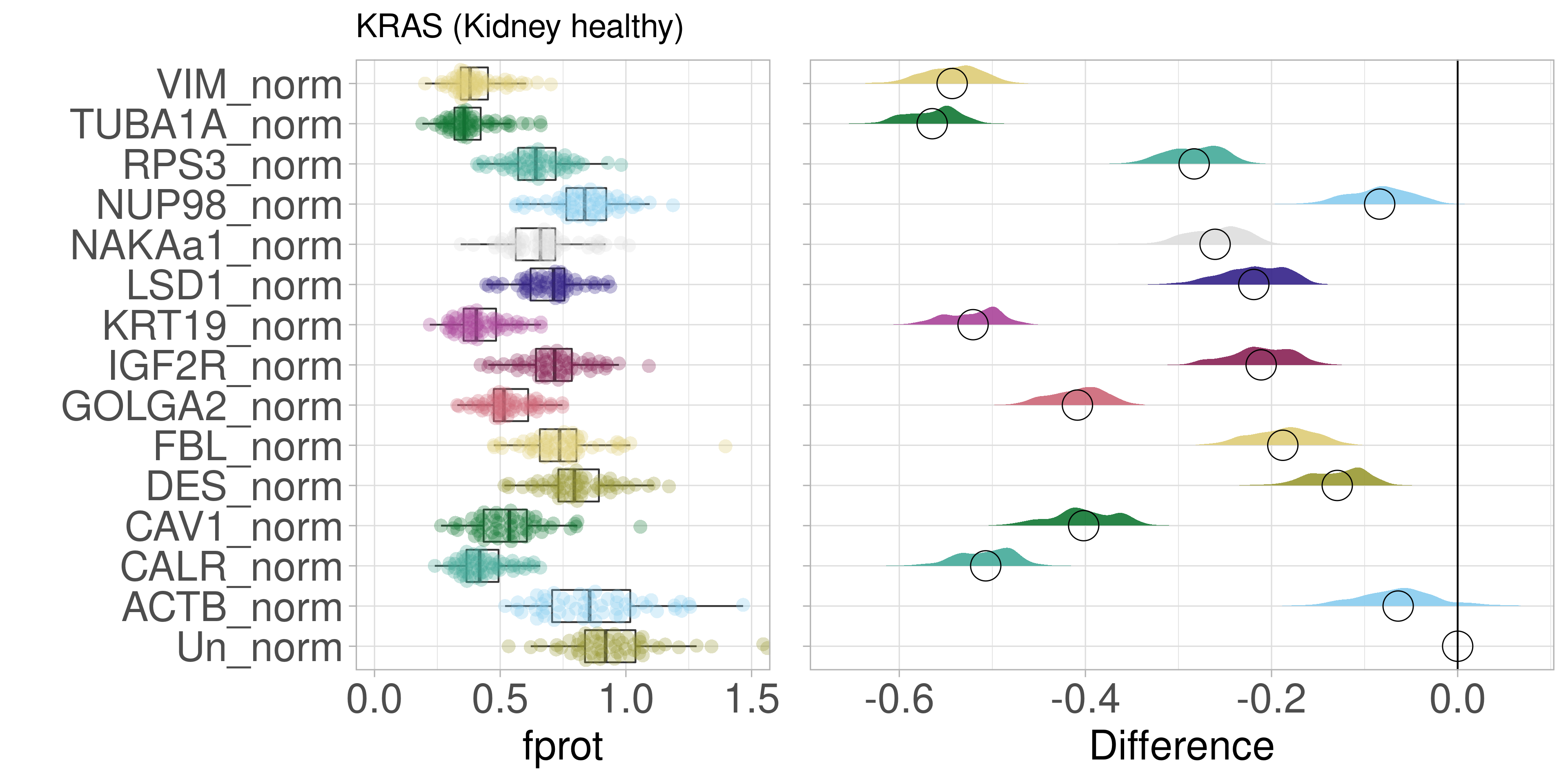

Supplement: Supplementary file 17 — Supplementary Material 17 [file 41598_2026_48754_MOESM17_ESM.zip › RPPA normalizations to cell markers/Kidney_plots/Oncoprotein_Kidney/KRAS_Kidney_H.png]

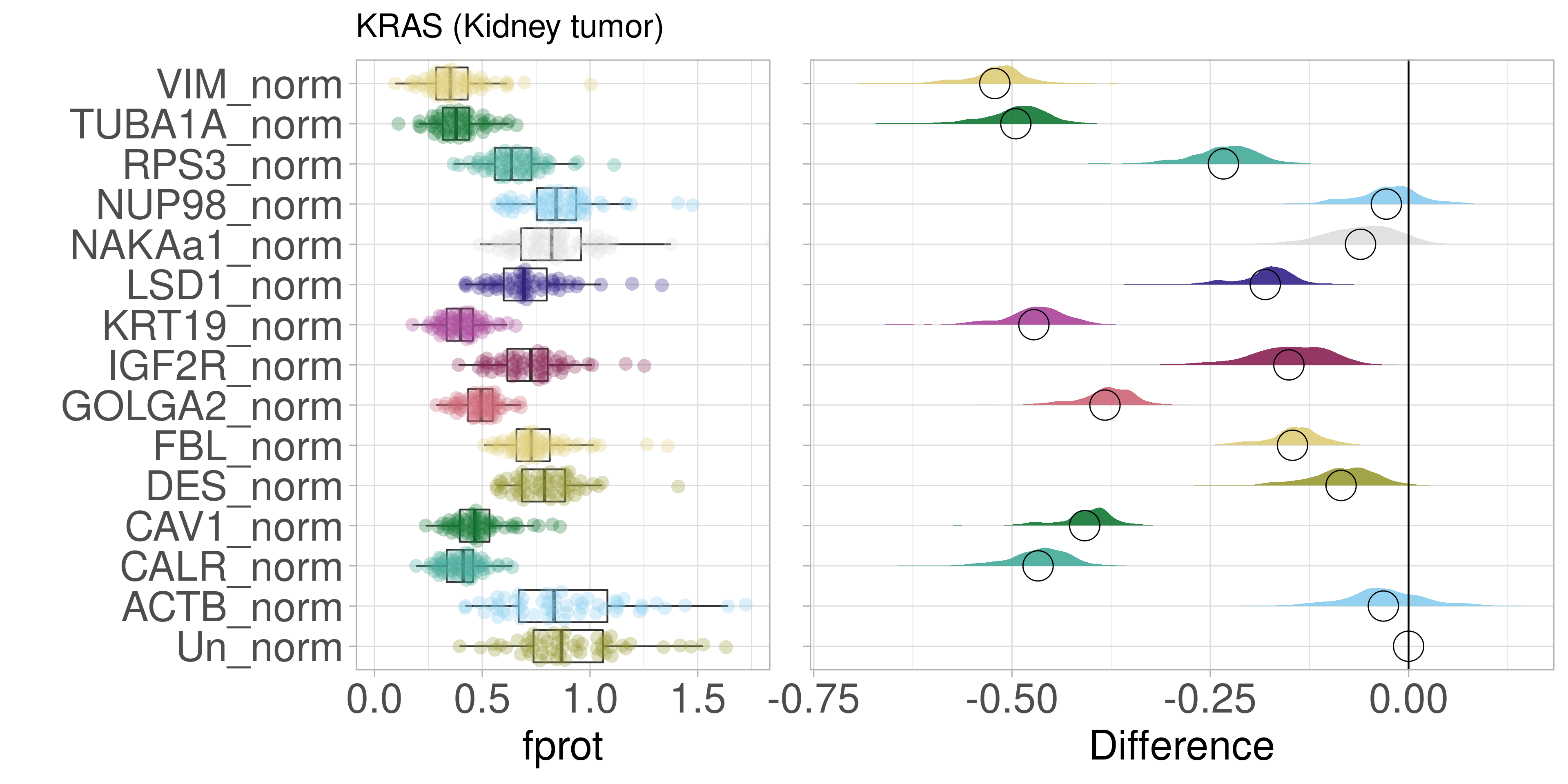

Supplement: Supplementary file 17 — Supplementary Material 17 [file 41598_2026_48754_MOESM17_ESM.zip › RPPA normalizations to cell markers/Kidney_plots/Oncoprotein_Kidney/KRAS_Kidney_T.png]

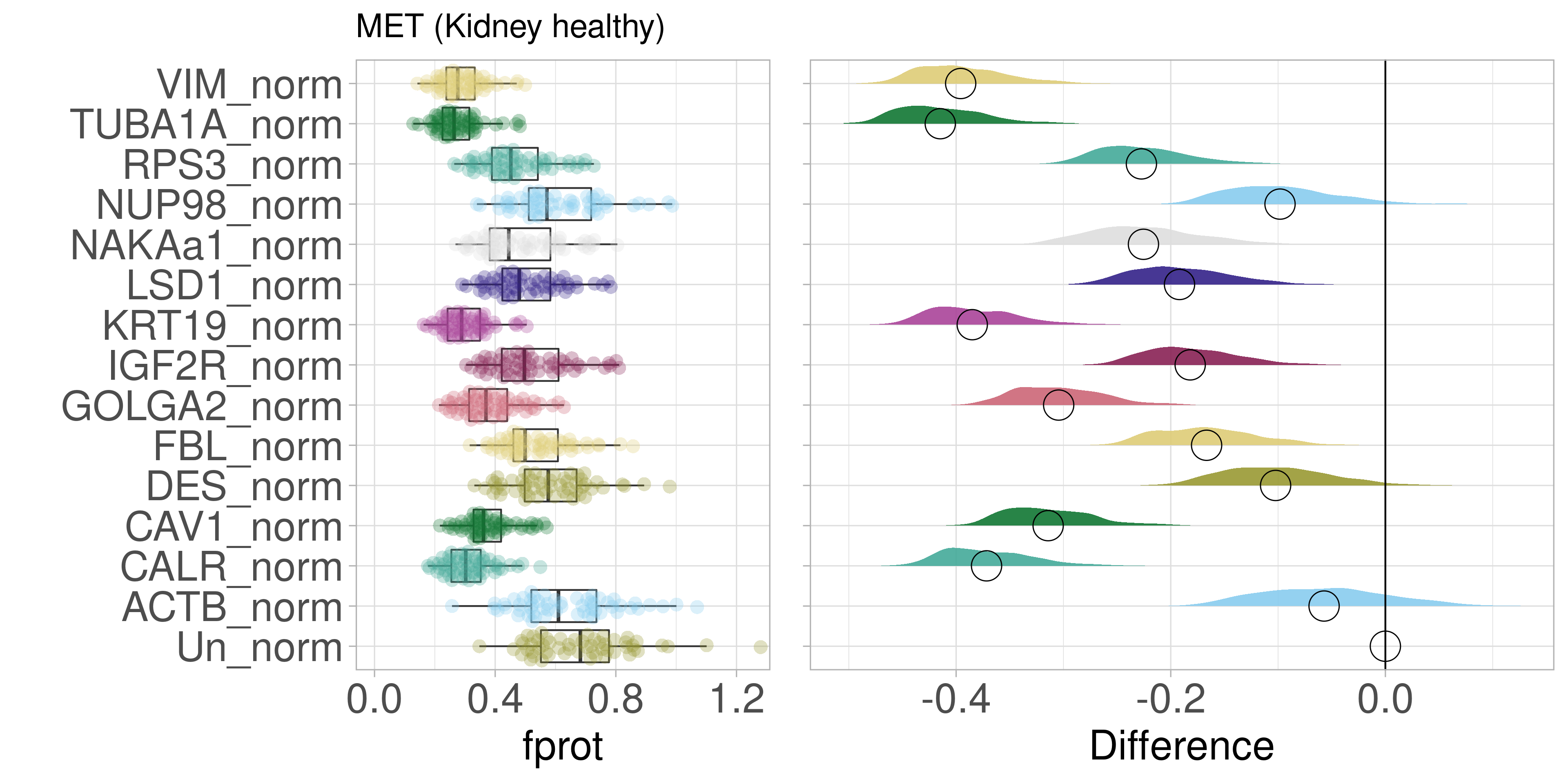

Supplement: Supplementary file 17 — Supplementary Material 17 [file 41598_2026_48754_MOESM17_ESM.zip › RPPA normalizations to cell markers/Kidney_plots/Oncoprotein_Kidney/MET_Kidney_H.png]

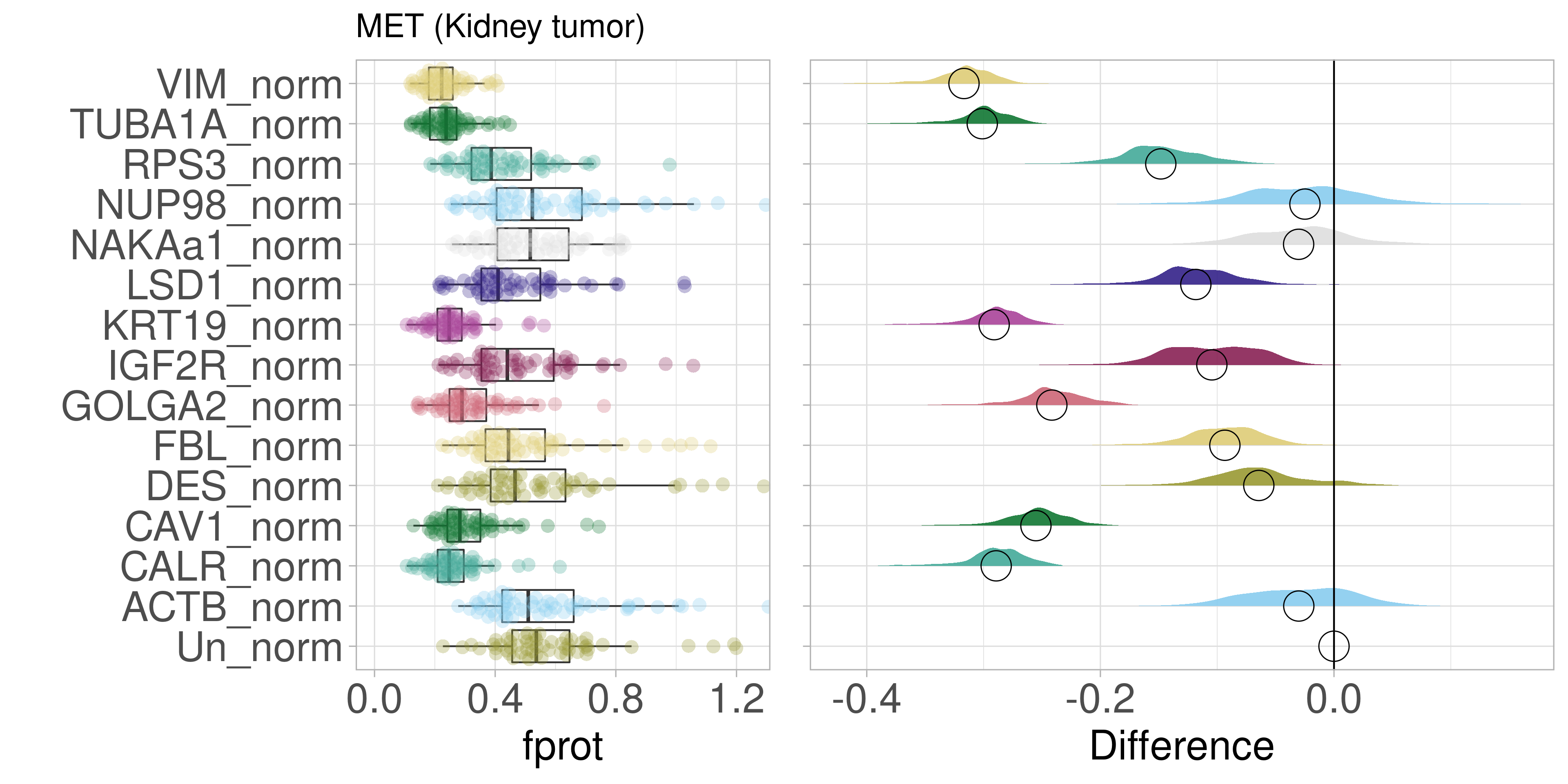

Supplement: Supplementary file 17 — Supplementary Material 17 [file 41598_2026_48754_MOESM17_ESM.zip › RPPA normalizations to cell markers/Kidney_plots/Oncoprotein_Kidney/MET_Kidney_T.png]

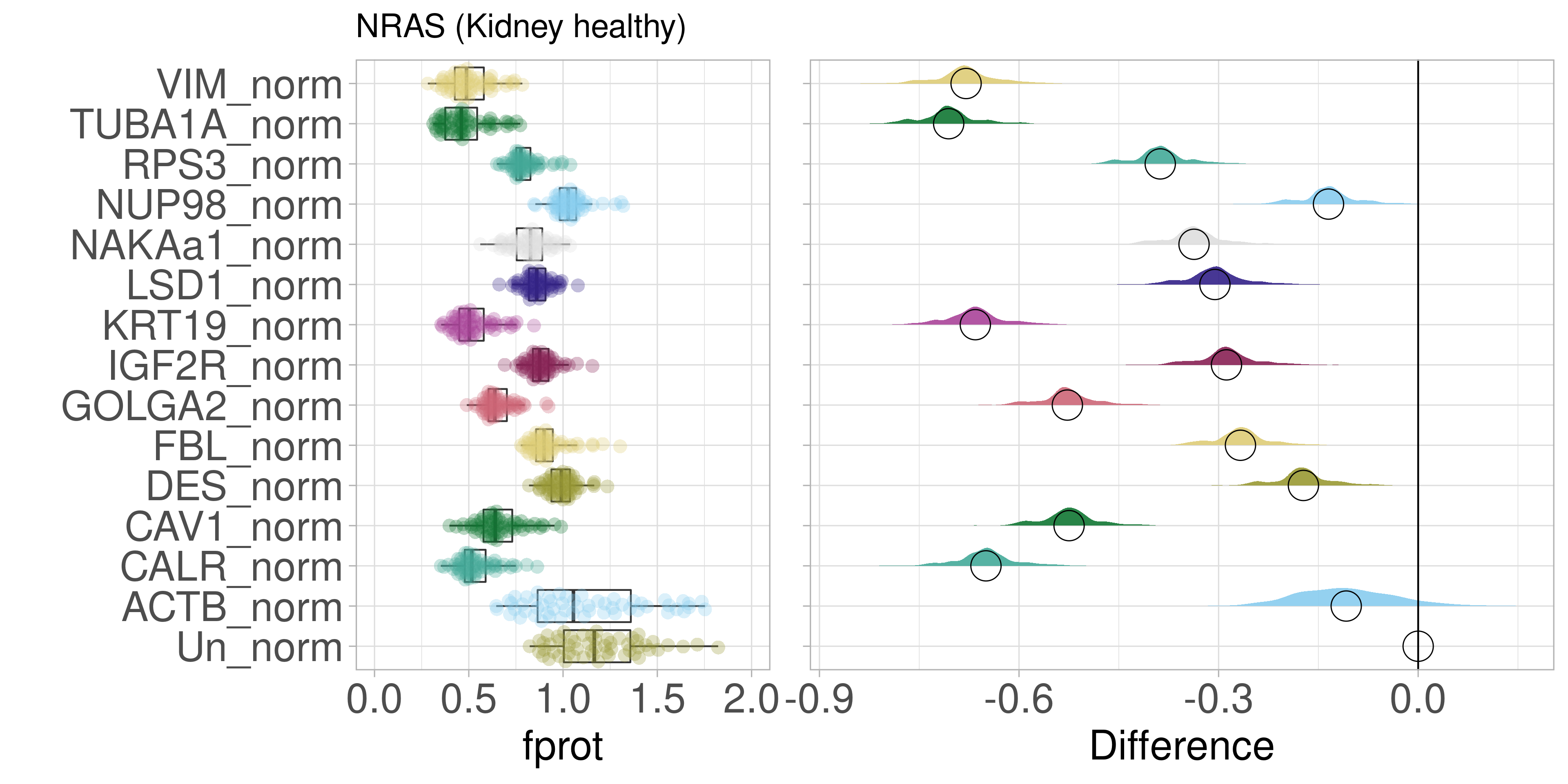

Supplement: Supplementary file 17 — Supplementary Material 17 [file 41598_2026_48754_MOESM17_ESM.zip › RPPA normalizations to cell markers/Kidney_plots/Oncoprotein_Kidney/NRAS_Kidney_H.png]

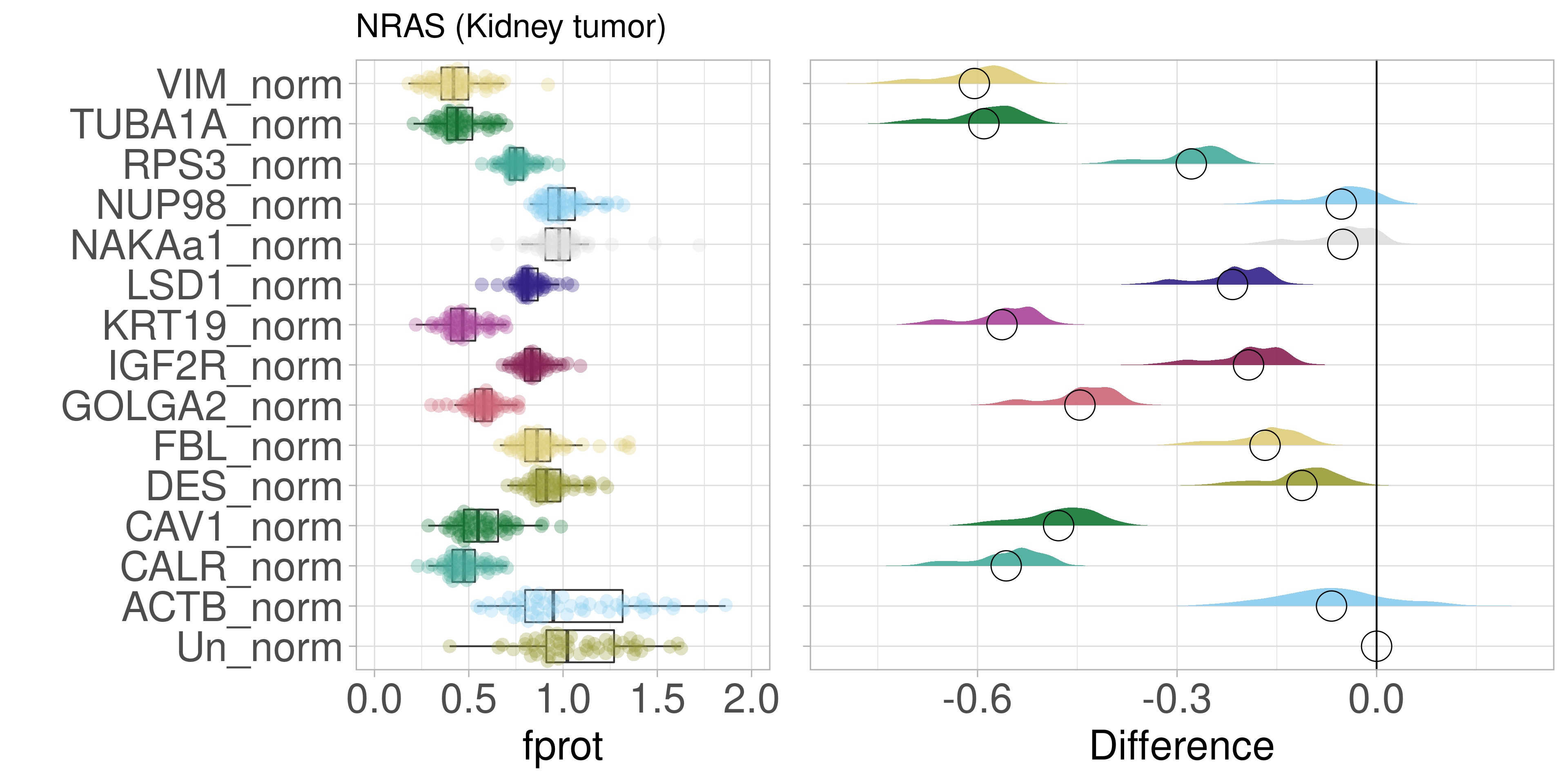

Supplement: Supplementary file 17 — Supplementary Material 17 [file 41598_2026_48754_MOESM17_ESM.zip › RPPA normalizations to cell markers/Kidney_plots/Oncoprotein_Kidney/NRAS_Kidney_T.png]

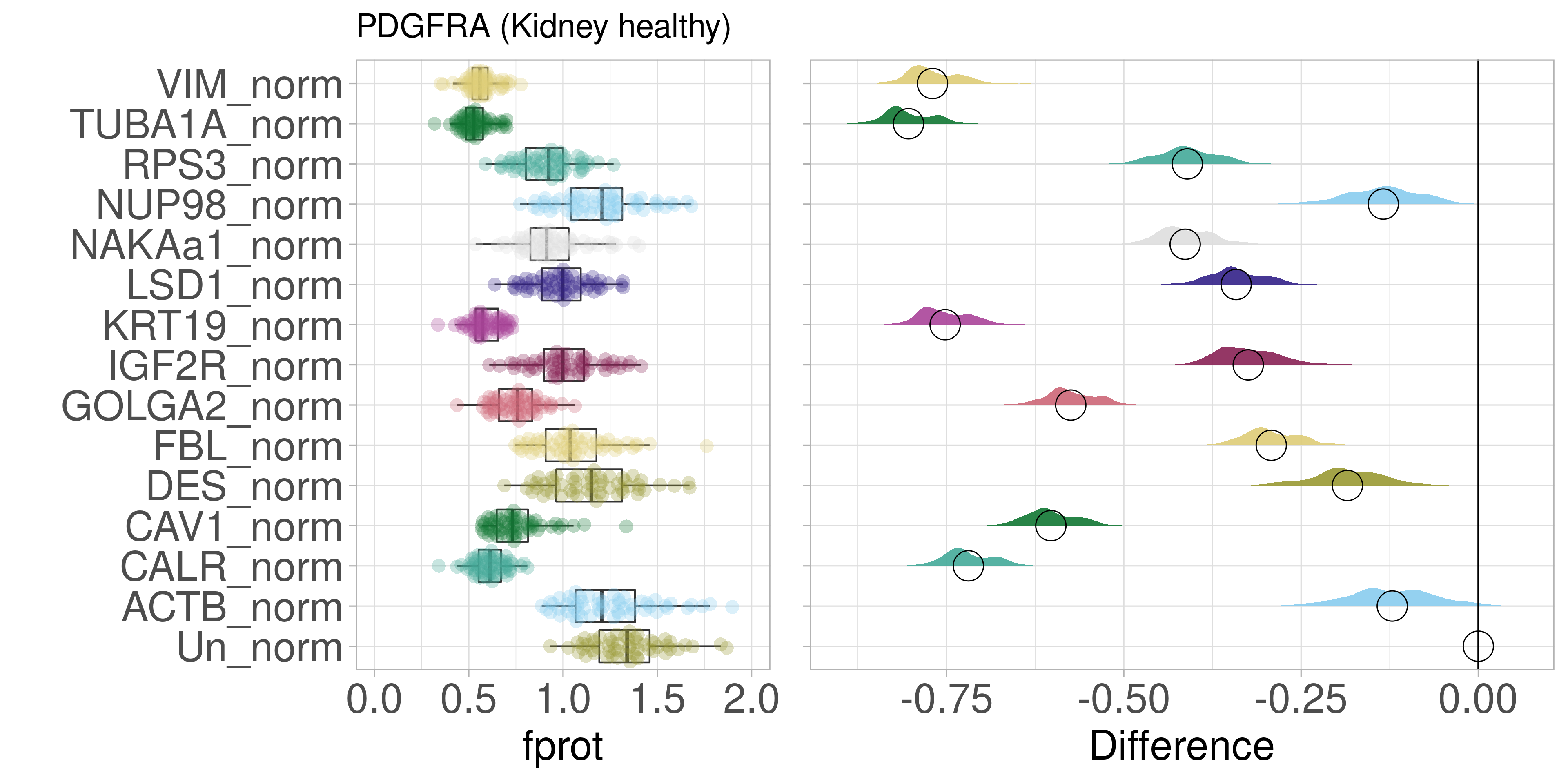

Supplement: Supplementary file 17 — Supplementary Material 17 [file 41598_2026_48754_MOESM17_ESM.zip › RPPA normalizations to cell markers/Kidney_plots/Oncoprotein_Kidney/PDGFRA_Kidney_H.png]

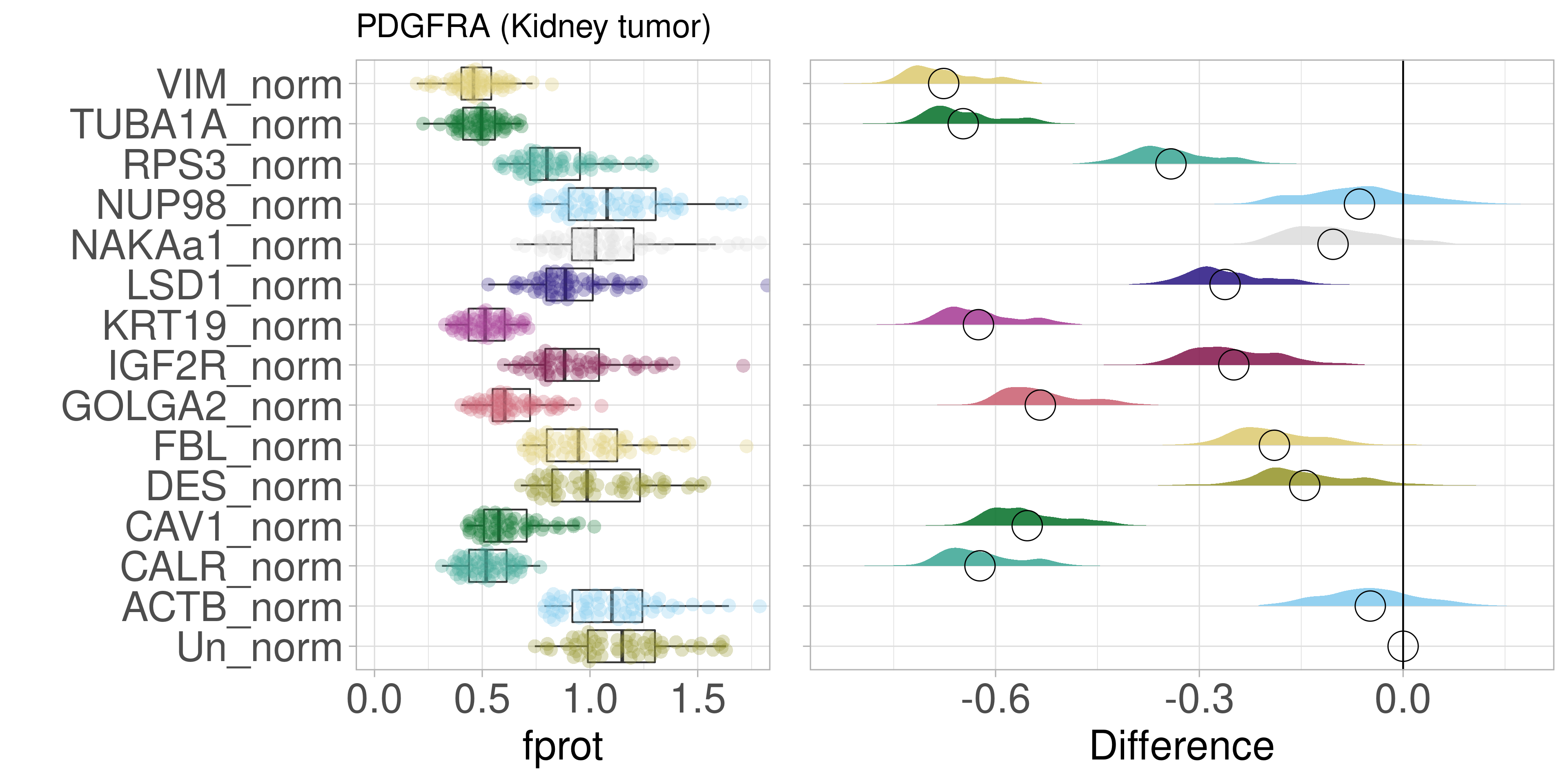

Supplement: Supplementary file 17 — Supplementary Material 17 [file 41598_2026_48754_MOESM17_ESM.zip › RPPA normalizations to cell markers/Kidney_plots/Oncoprotein_Kidney/PDGFRA_Kidney_T.png]

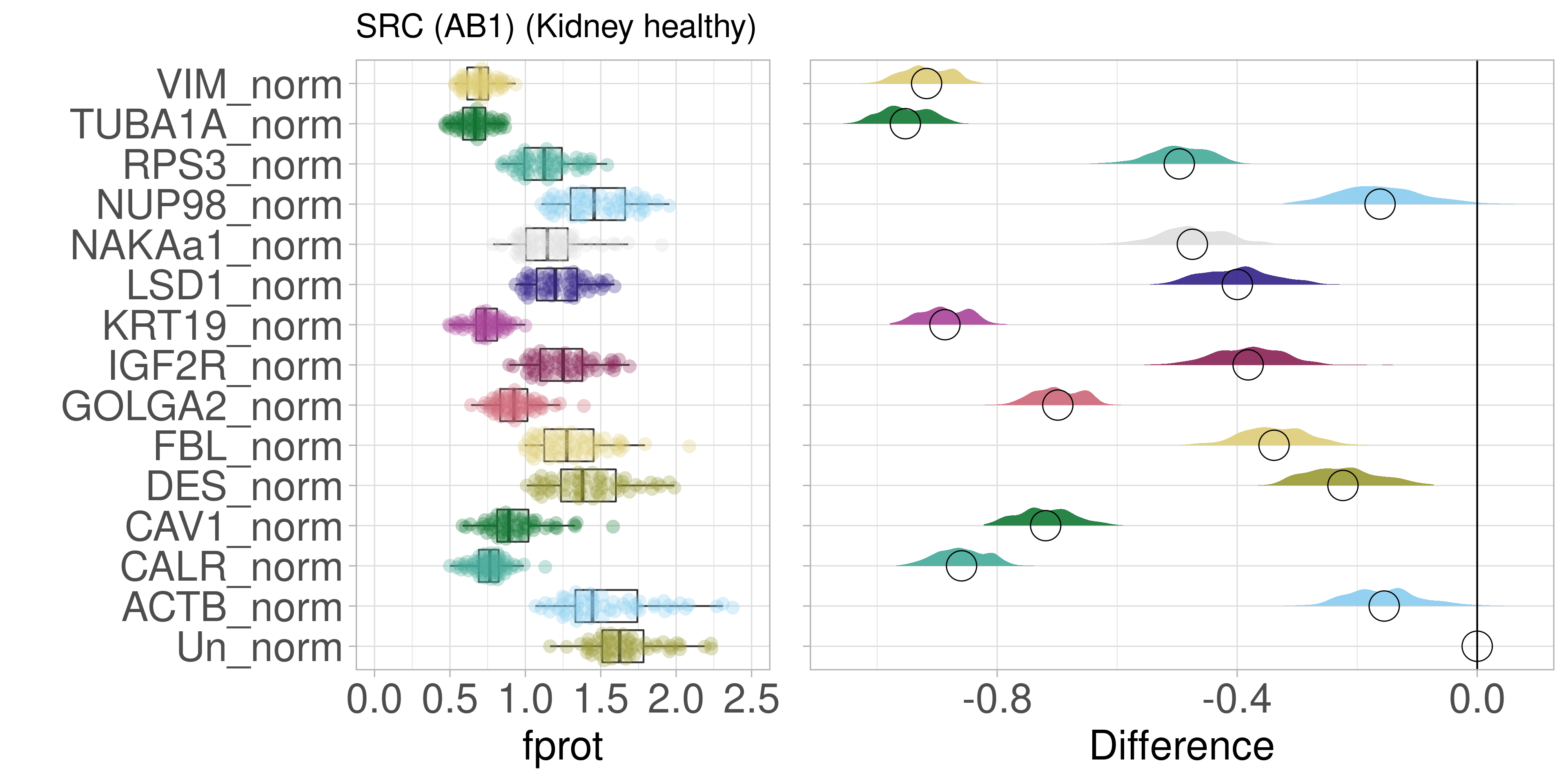

Supplement: Supplementary file 17 — Supplementary Material 17 [file 41598_2026_48754_MOESM17_ESM.zip › RPPA normalizations to cell markers/Kidney_plots/Oncoprotein_Kidney/SRC(AB1)_Kidney_H.png]

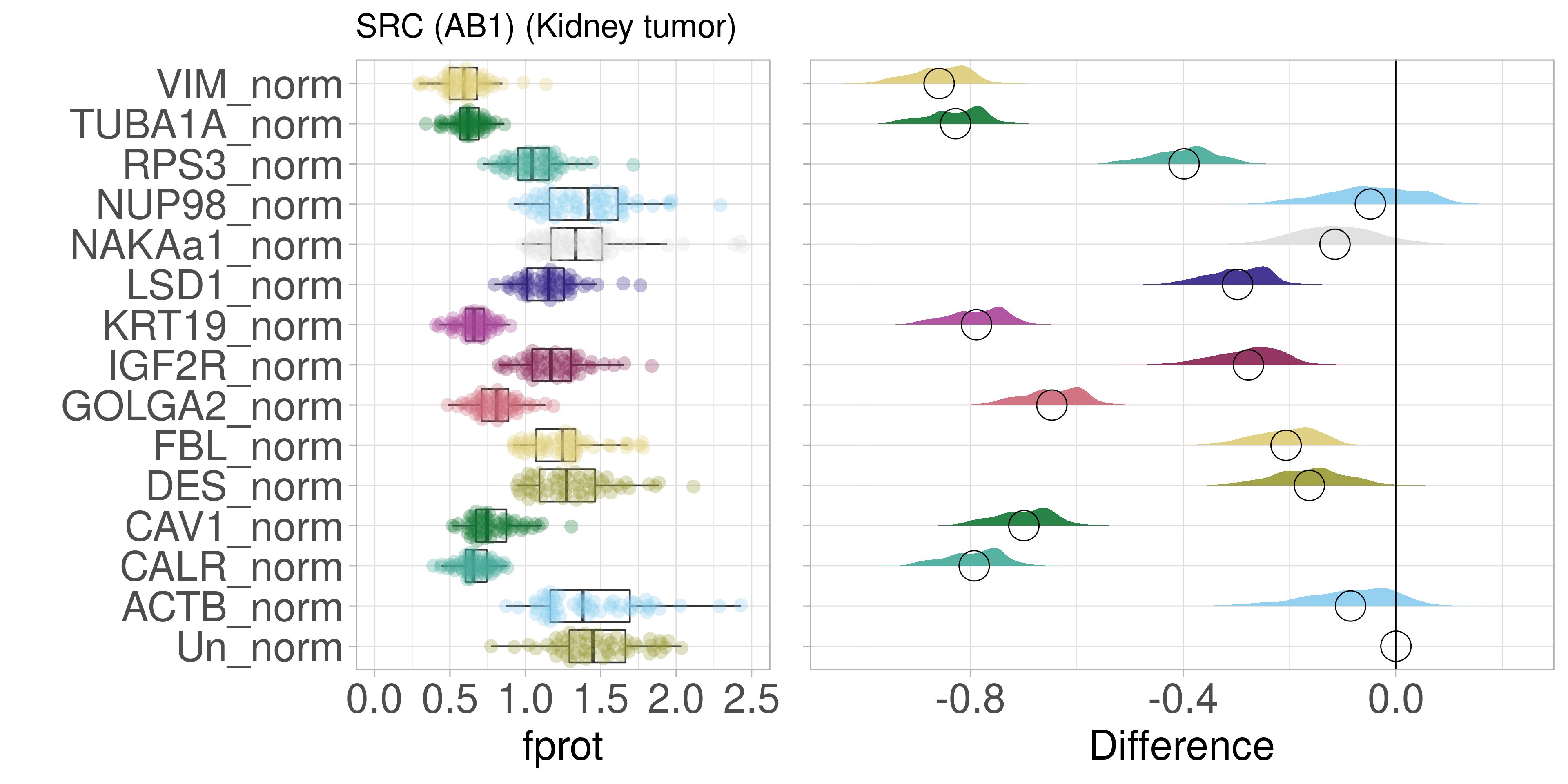

Supplement: Supplementary file 17 — Supplementary Material 17 [file 41598_2026_48754_MOESM17_ESM.zip › RPPA normalizations to cell markers/Kidney_plots/Oncoprotein_Kidney/SRC(AB1)_Kidney_T.png]

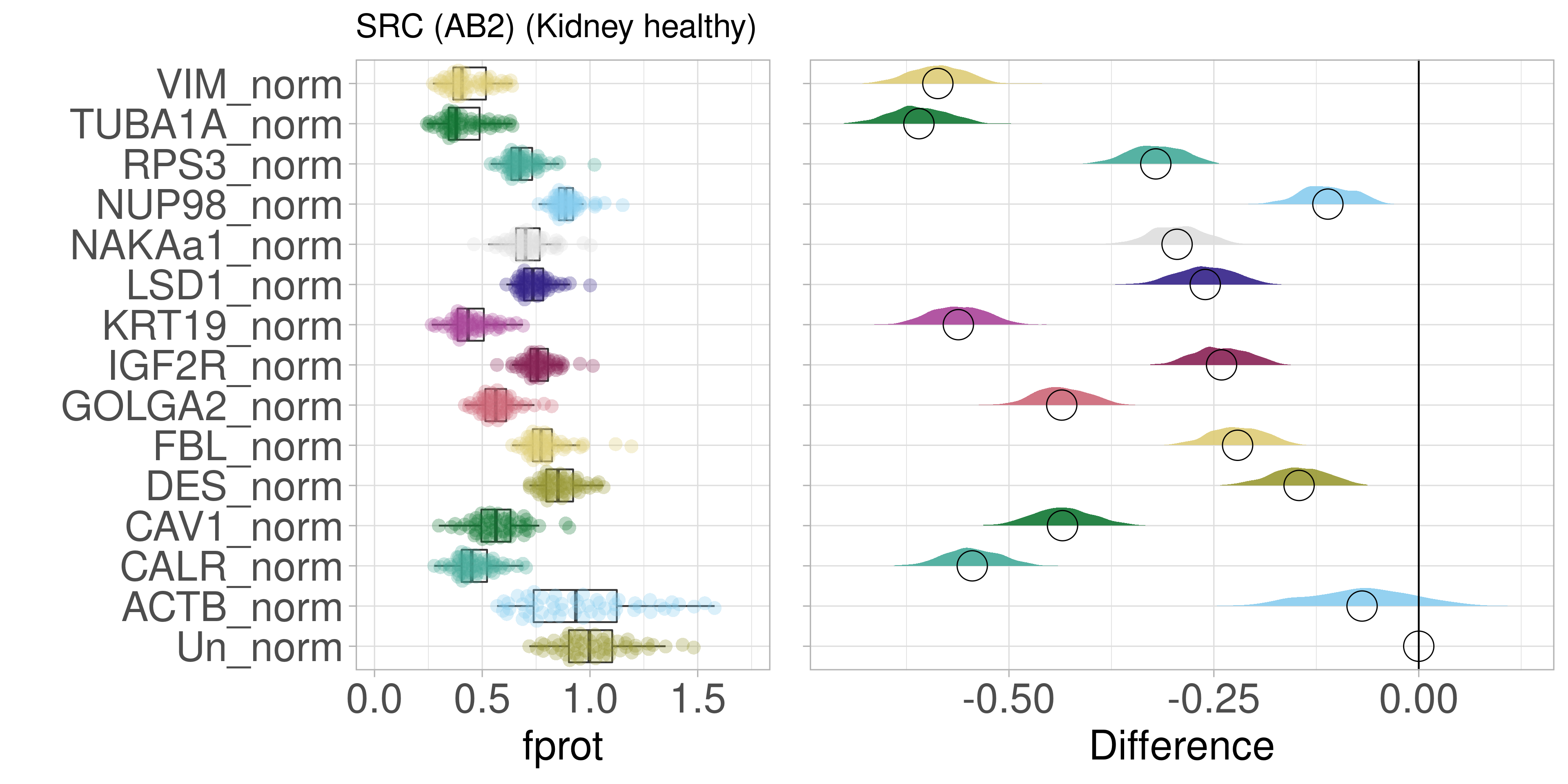

Supplement: Supplementary file 17 — Supplementary Material 17 [file 41598_2026_48754_MOESM17_ESM.zip › RPPA normalizations to cell markers/Kidney_plots/Oncoprotein_Kidney/SRC(AB2)_Kidney_H.png]

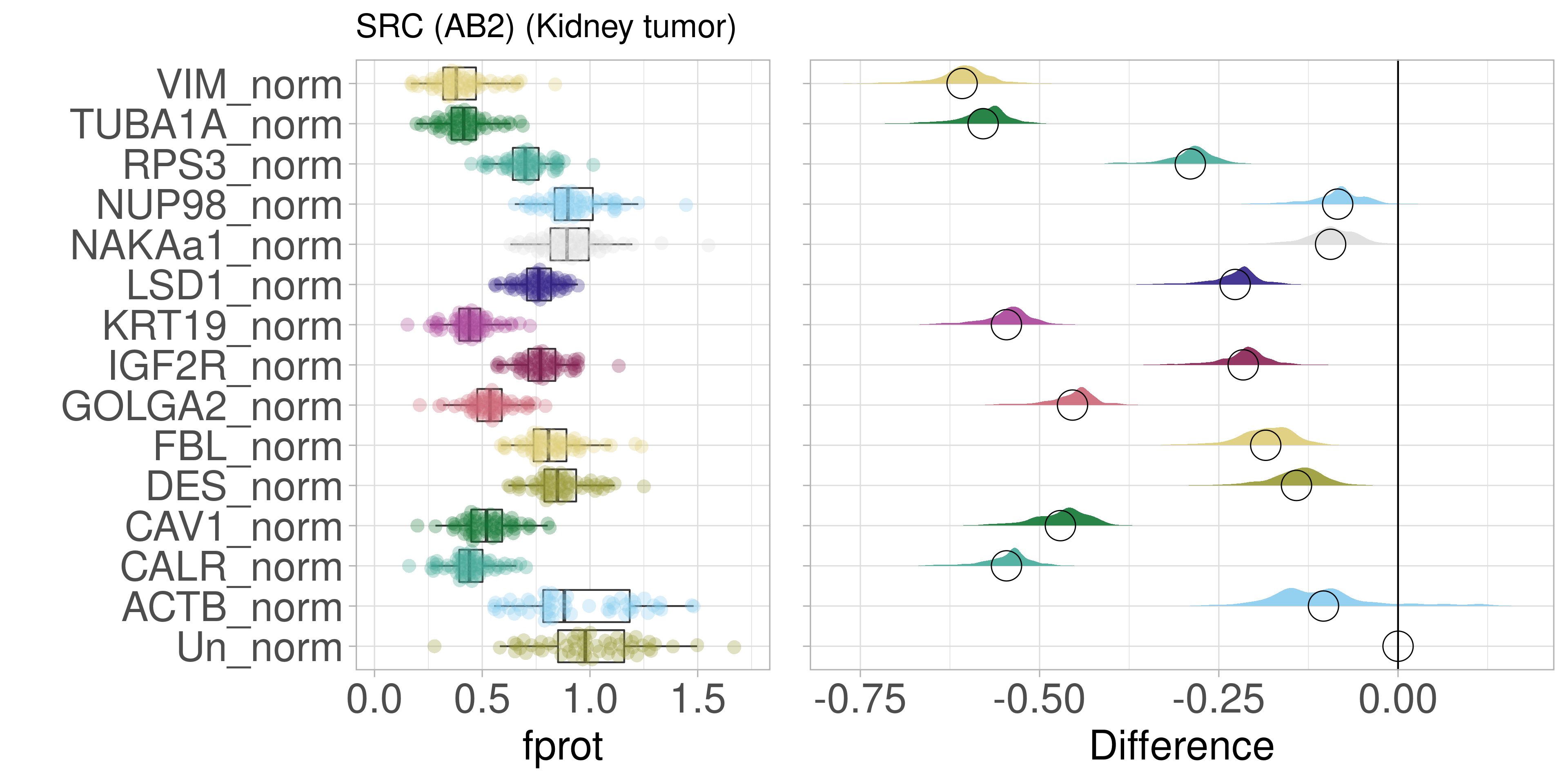

Supplement: Supplementary file 17 — Supplementary Material 17 [file 41598_2026_48754_MOESM17_ESM.zip › RPPA normalizations to cell markers/Kidney_plots/Oncoprotein_Kidney/SRC(AB2)_Kidney_T.png]

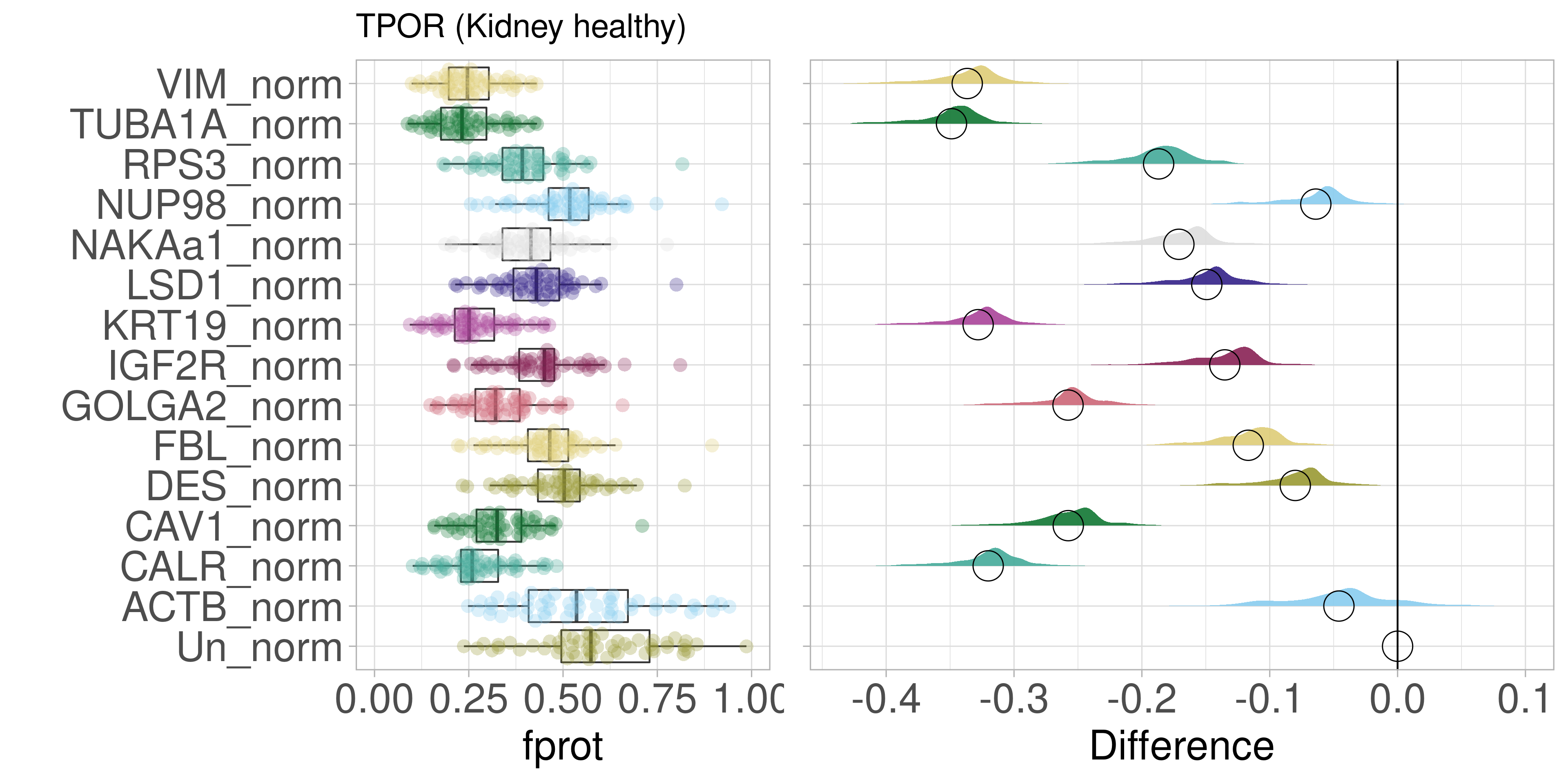

Supplement: Supplementary file 17 — Supplementary Material 17 [file 41598_2026_48754_MOESM17_ESM.zip › RPPA normalizations to cell markers/Kidney_plots/Oncoprotein_Kidney/TPOR_Kidney_H.png]

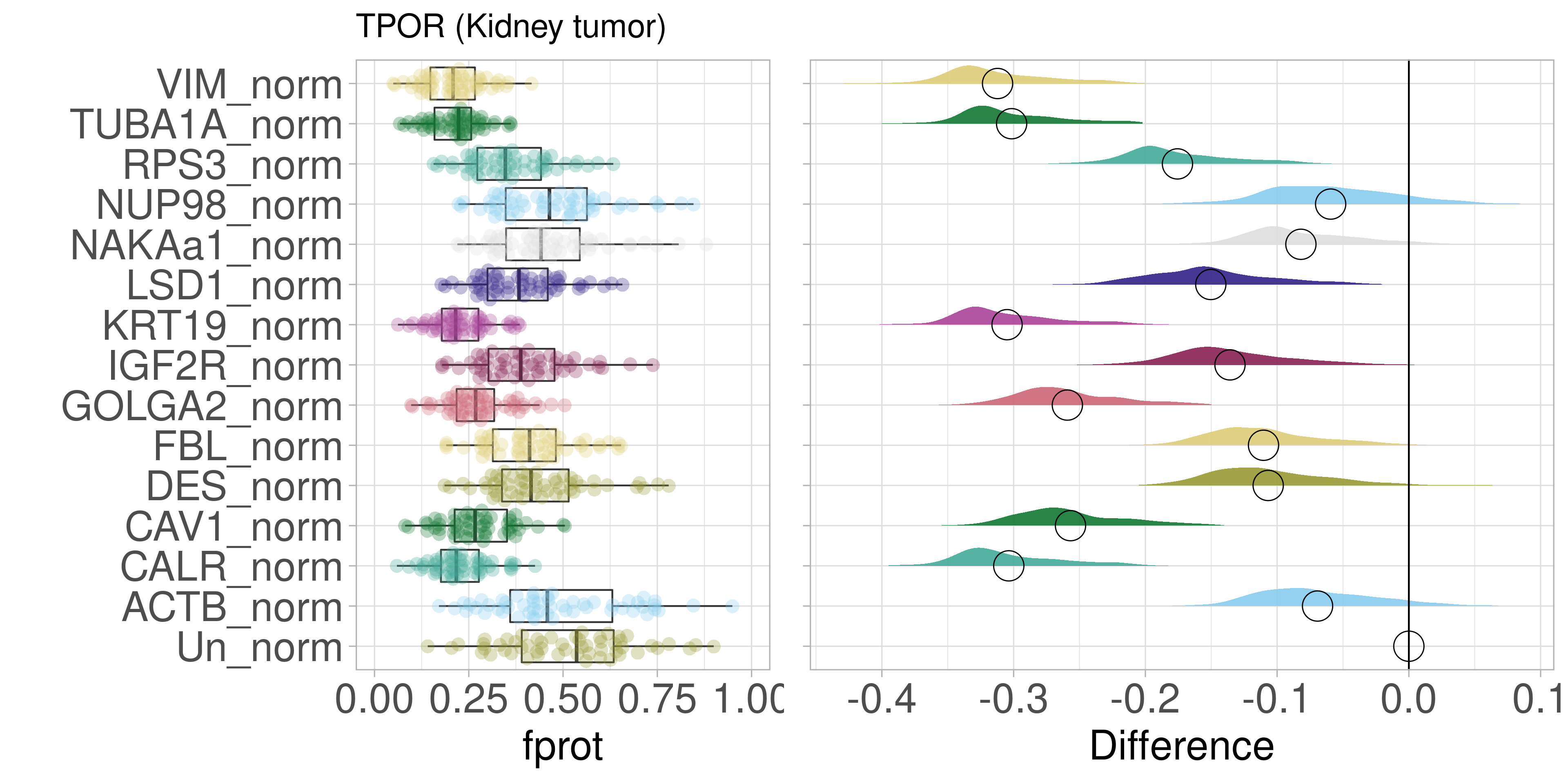

Supplement: Supplementary file 17 — Supplementary Material 17 [file 41598_2026_48754_MOESM17_ESM.zip › RPPA normalizations to cell markers/Kidney_plots/Oncoprotein_Kidney/TPOR_Kidney_T.png]

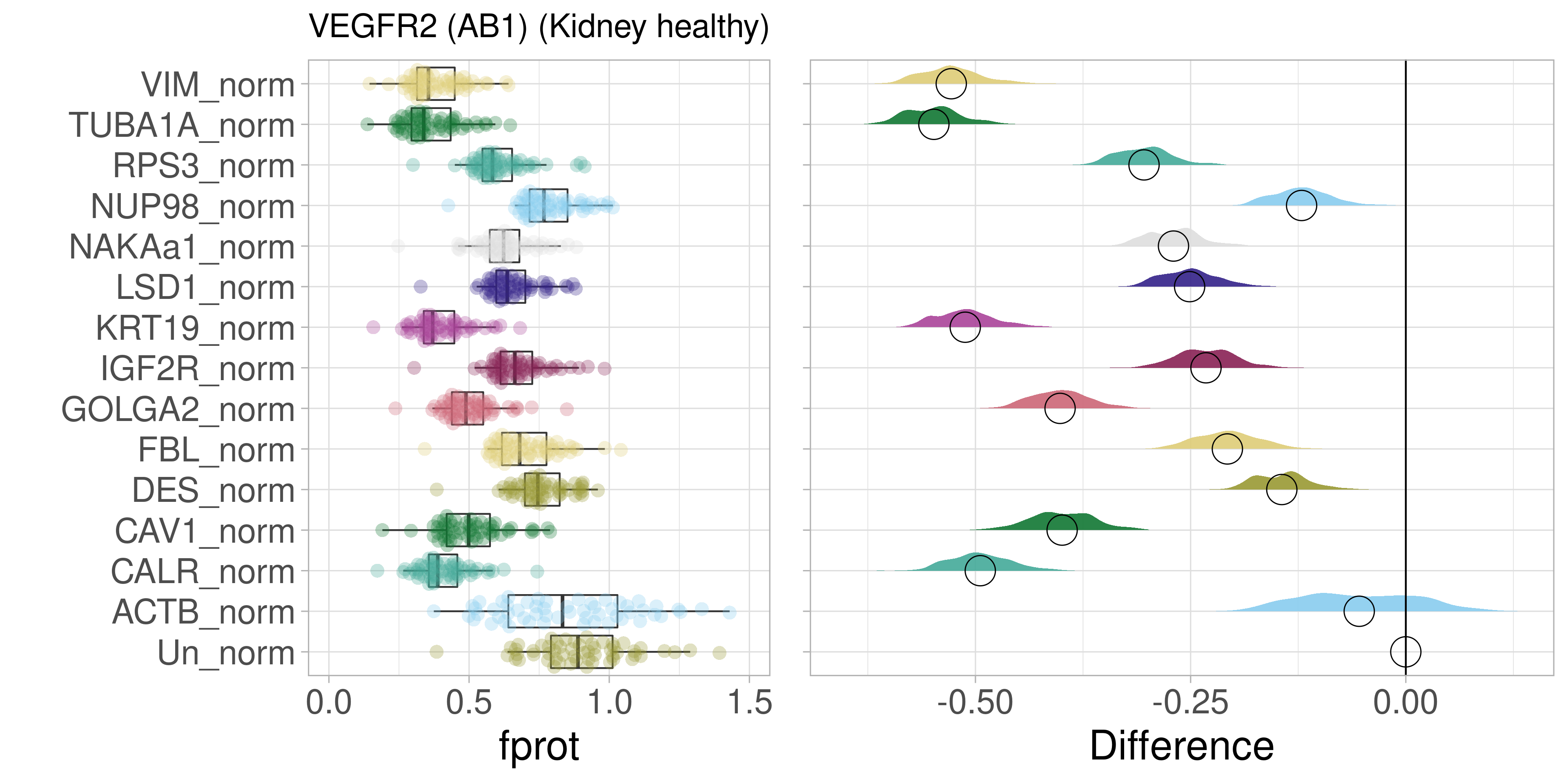

Supplement: Supplementary file 17 — Supplementary Material 17 [file 41598_2026_48754_MOESM17_ESM.zip › RPPA normalizations to cell markers/Kidney_plots/Oncoprotein_Kidney/VEGFR2(AB1)_Kidney_H.png]

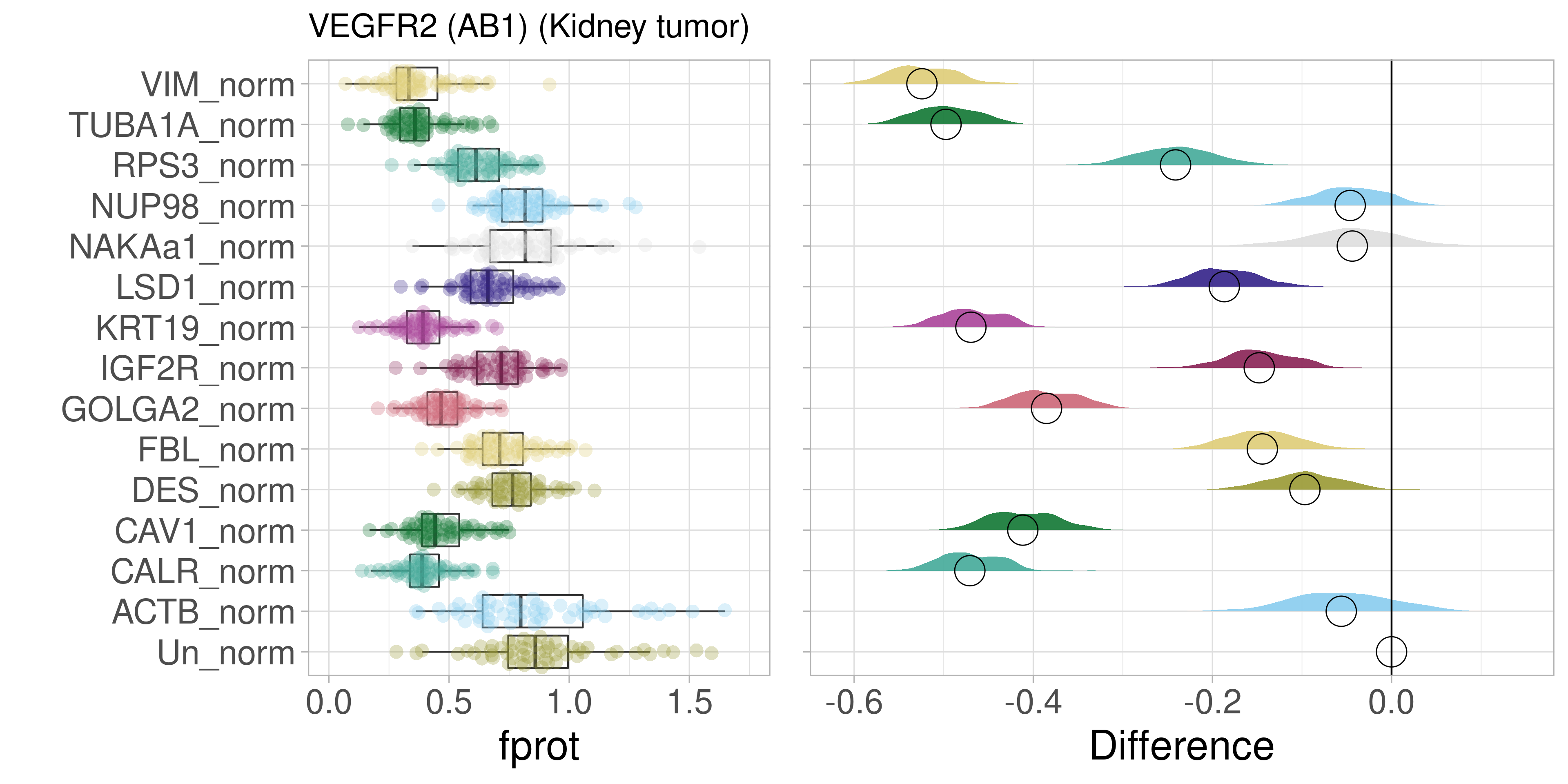

Supplement: Supplementary file 17 — Supplementary Material 17 [file 41598_2026_48754_MOESM17_ESM.zip › RPPA normalizations to cell markers/Kidney_plots/Oncoprotein_Kidney/VEGFR2(AB1)_Kidney_T.png]

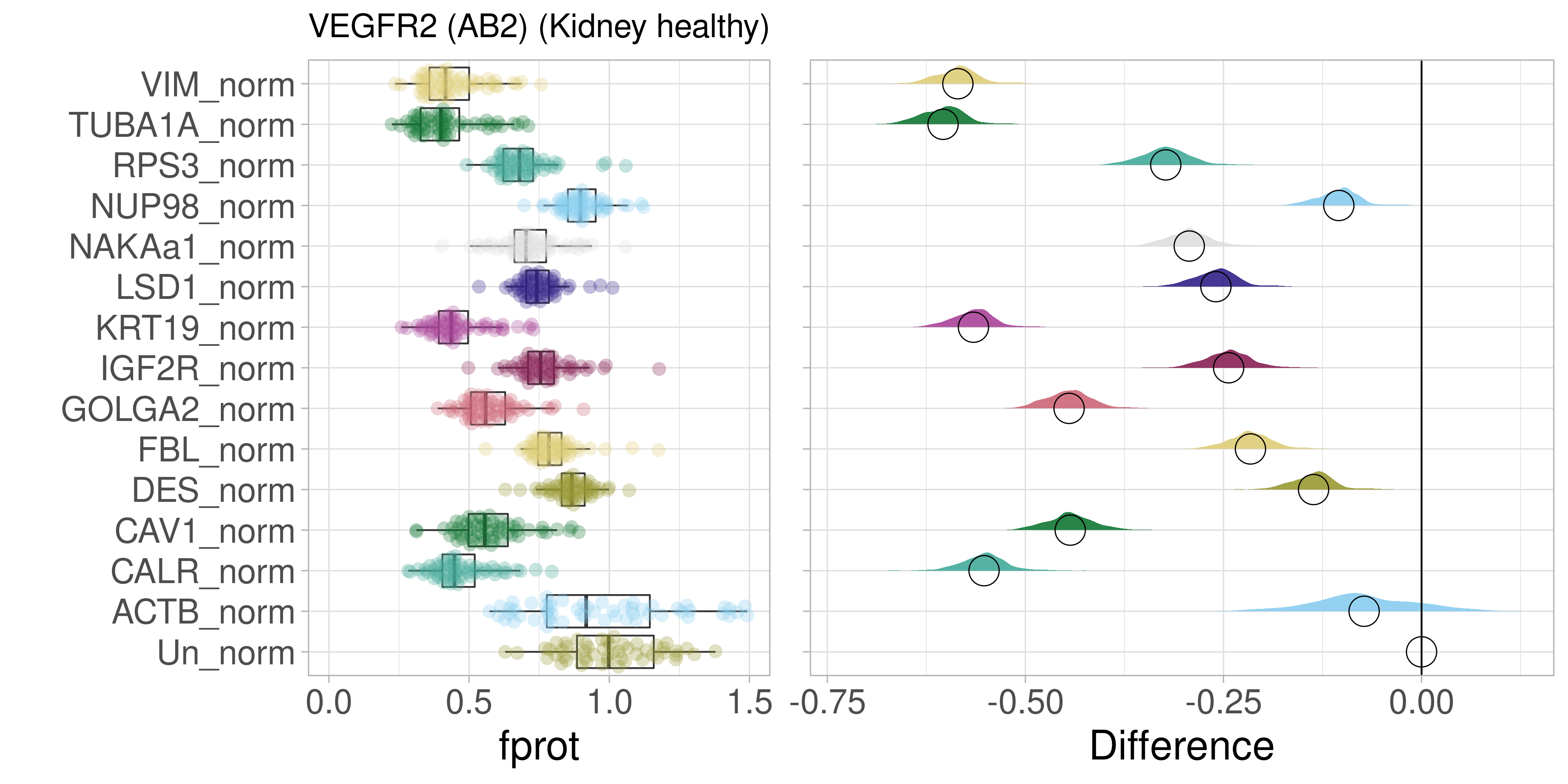

Supplement: Supplementary file 17 — Supplementary Material 17 [file 41598_2026_48754_MOESM17_ESM.zip › RPPA normalizations to cell markers/Kidney_plots/Oncoprotein_Kidney/VEGFR2(AB2)_Kidney_H.png]

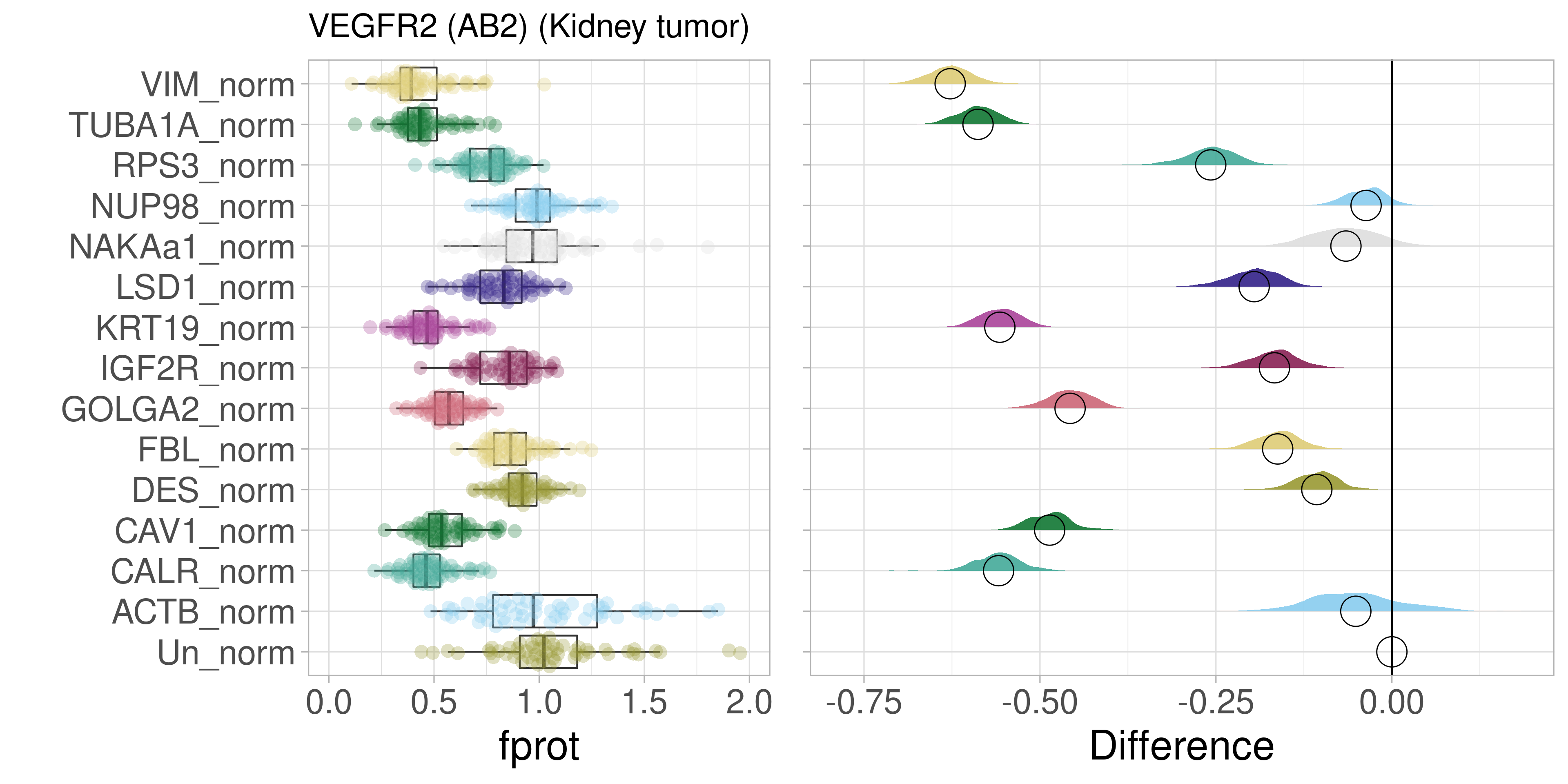

Supplement: Supplementary file 17 — Supplementary Material 17 [file 41598_2026_48754_MOESM17_ESM.zip › RPPA normalizations to cell markers/Kidney_plots/Oncoprotein_Kidney/VEGFR2(AB2)_Kidney_T.png]

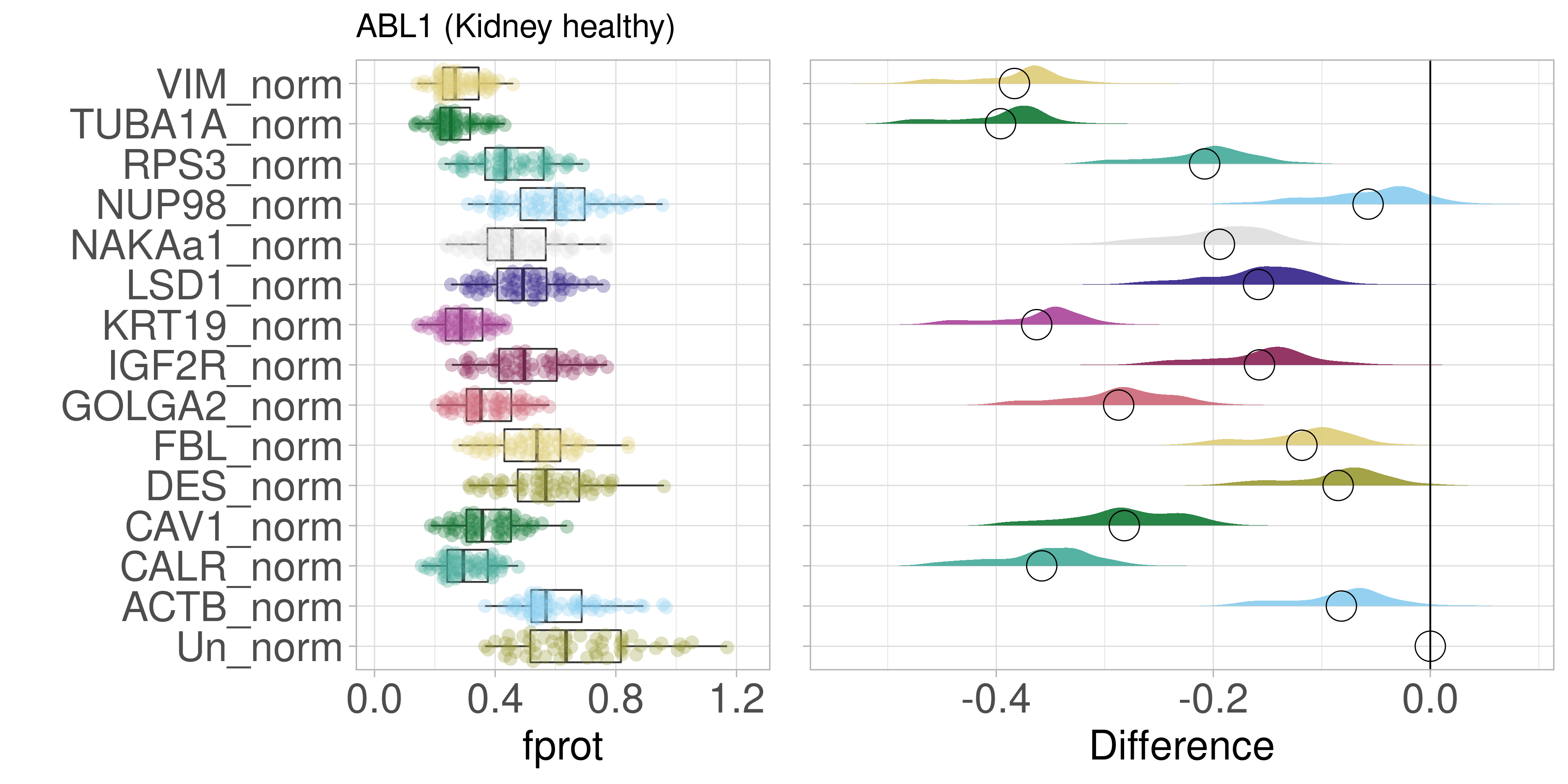

Supplement: Supplementary file 17 — Supplementary Material 17 [file 41598_2026_48754_MOESM17_ESM.zip › RPPA normalizations to cell markers/Kidney_plots/Tumor_suppr_Kidney/ABL1_Kidney_H.png]

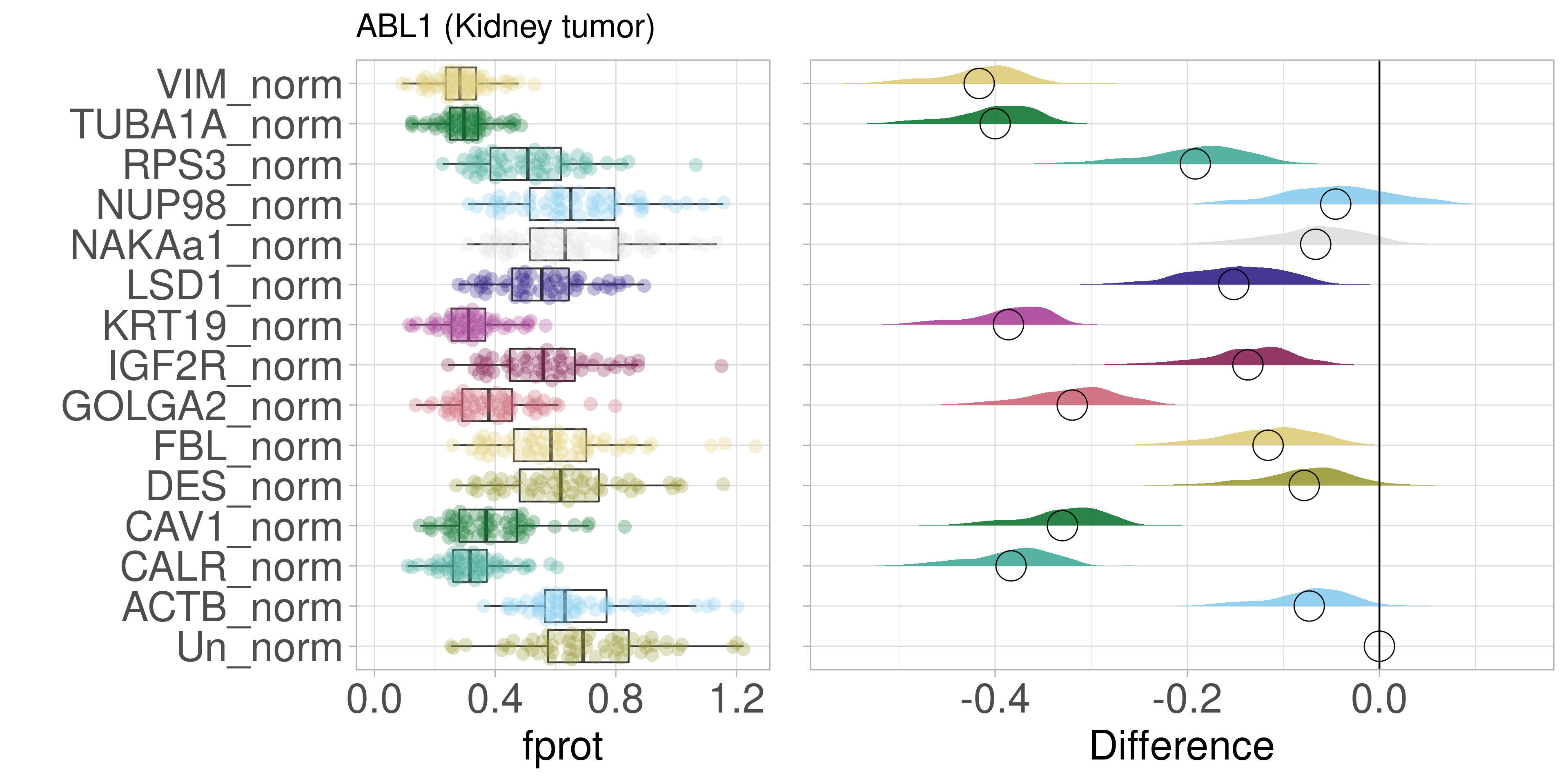

Supplement: Supplementary file 17 — Supplementary Material 17 [file 41598_2026_48754_MOESM17_ESM.zip › RPPA normalizations to cell markers/Kidney_plots/Tumor_suppr_Kidney/ABL1_Kidney_T.png]

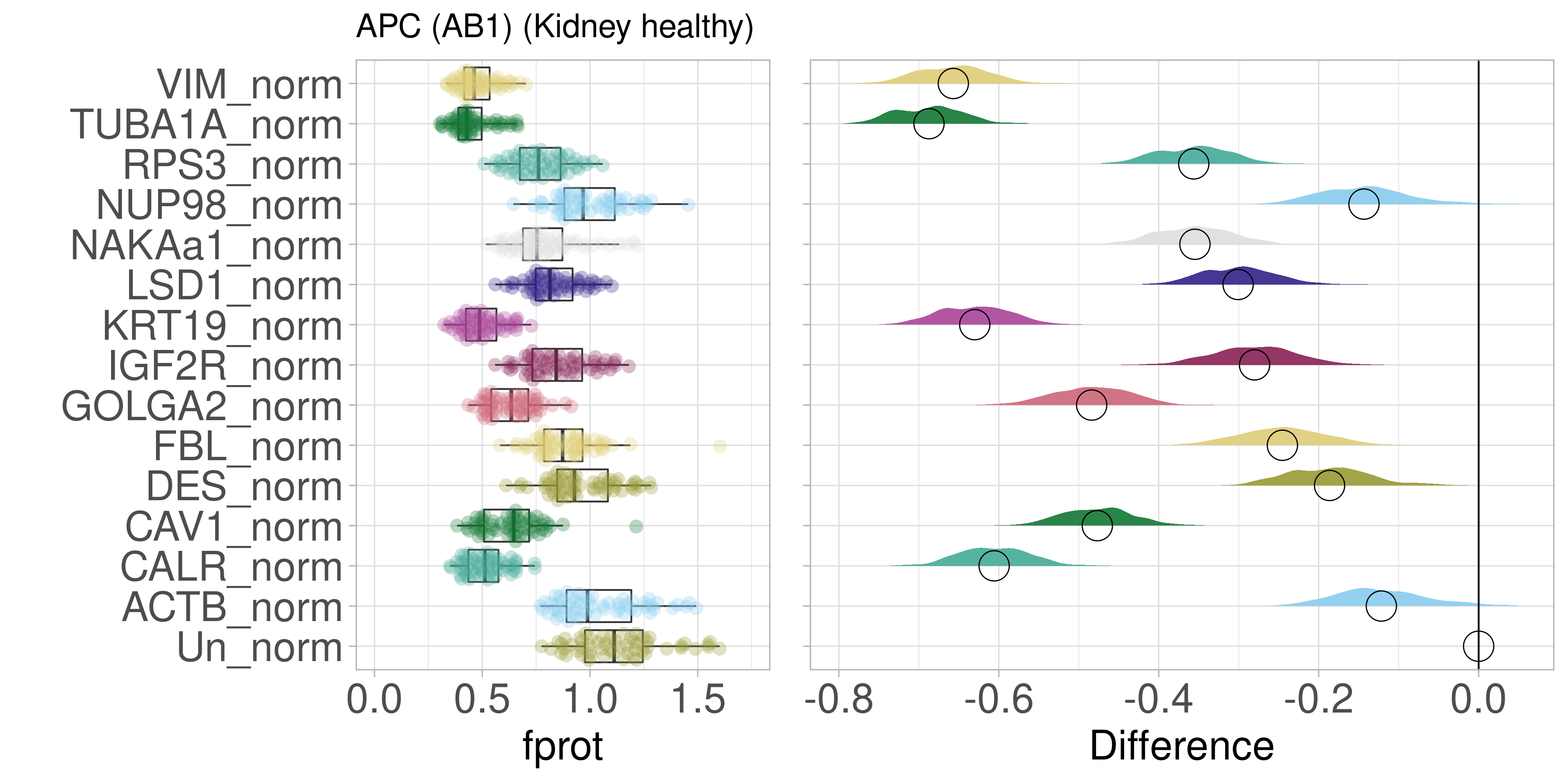

Supplement: Supplementary file 17 — Supplementary Material 17 [file 41598_2026_48754_MOESM17_ESM.zip › RPPA normalizations to cell markers/Kidney_plots/Tumor_suppr_Kidney/APC(AB1)_Kidney_H.png]

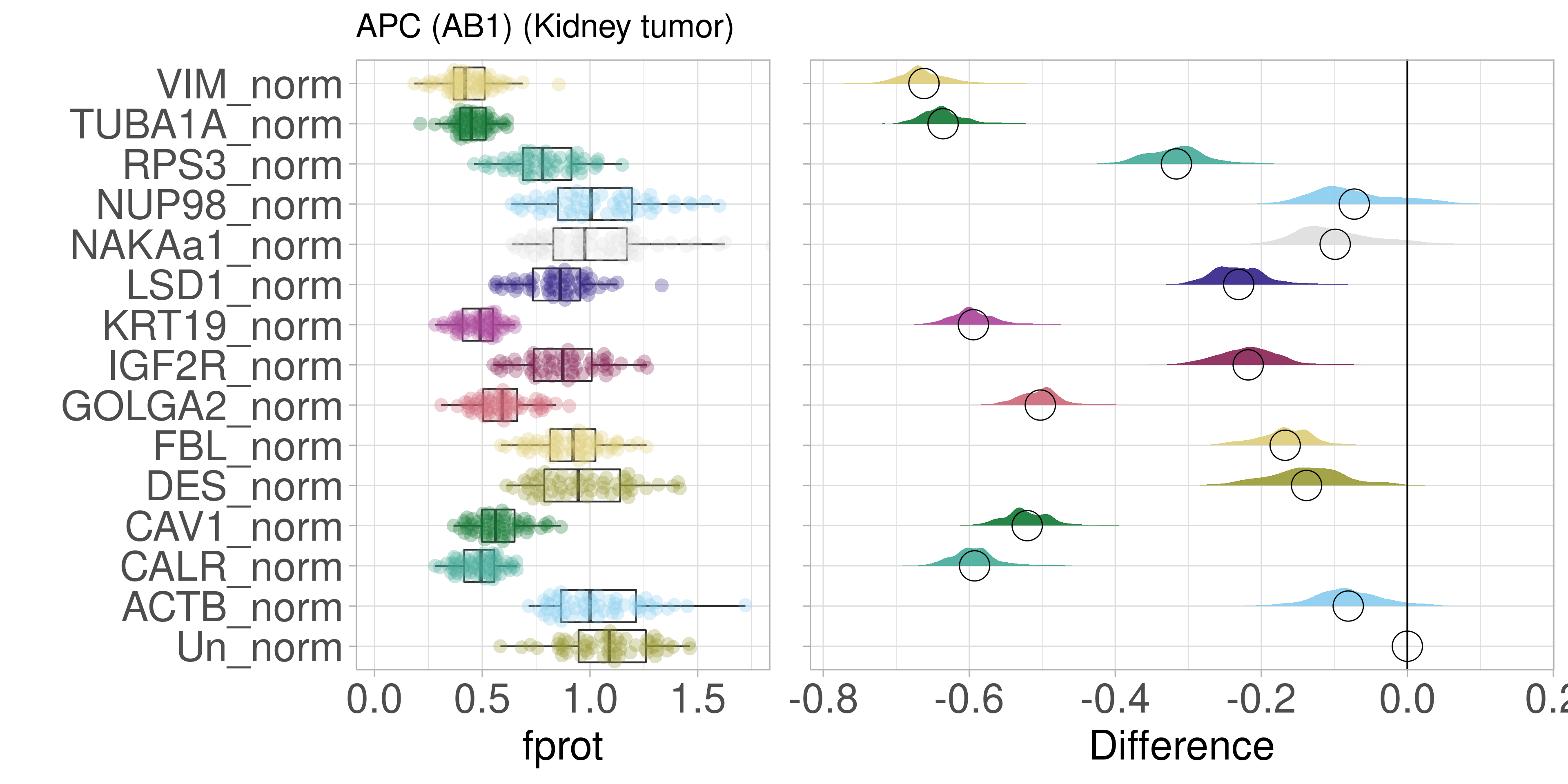

Supplement: Supplementary file 17 — Supplementary Material 17 [file 41598_2026_48754_MOESM17_ESM.zip › RPPA normalizations to cell markers/Kidney_plots/Tumor_suppr_Kidney/APC(AB1)_Kidney_T.png]

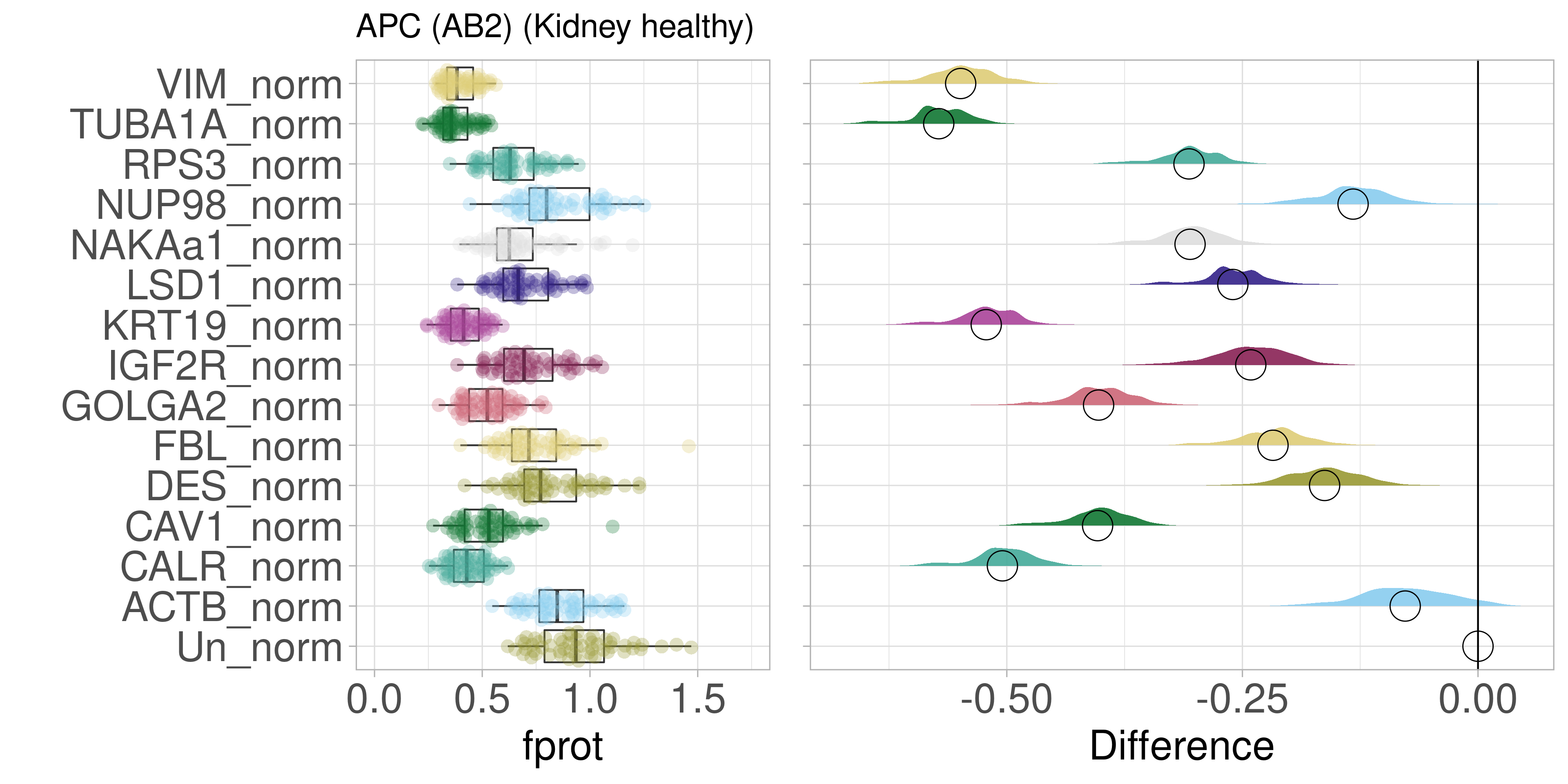

Supplement: Supplementary file 17 — Supplementary Material 17 [file 41598_2026_48754_MOESM17_ESM.zip › RPPA normalizations to cell markers/Kidney_plots/Tumor_suppr_Kidney/APC(AB2)_Kidney_H.png]

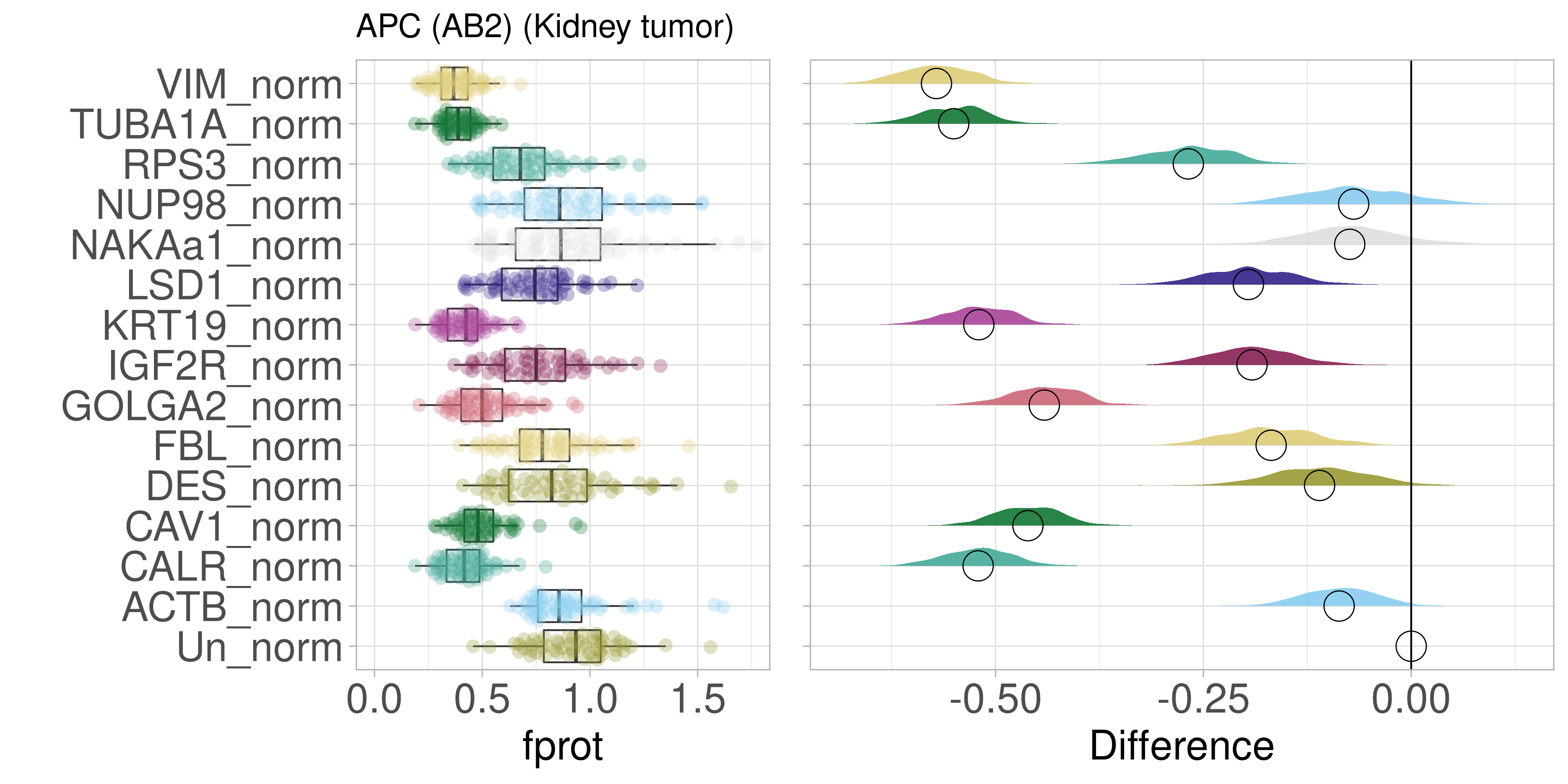

Supplement: Supplementary file 17 — Supplementary Material 17 [file 41598_2026_48754_MOESM17_ESM.zip › RPPA normalizations to cell markers/Kidney_plots/Tumor_suppr_Kidney/APC(AB2)_Kidney_T.png]

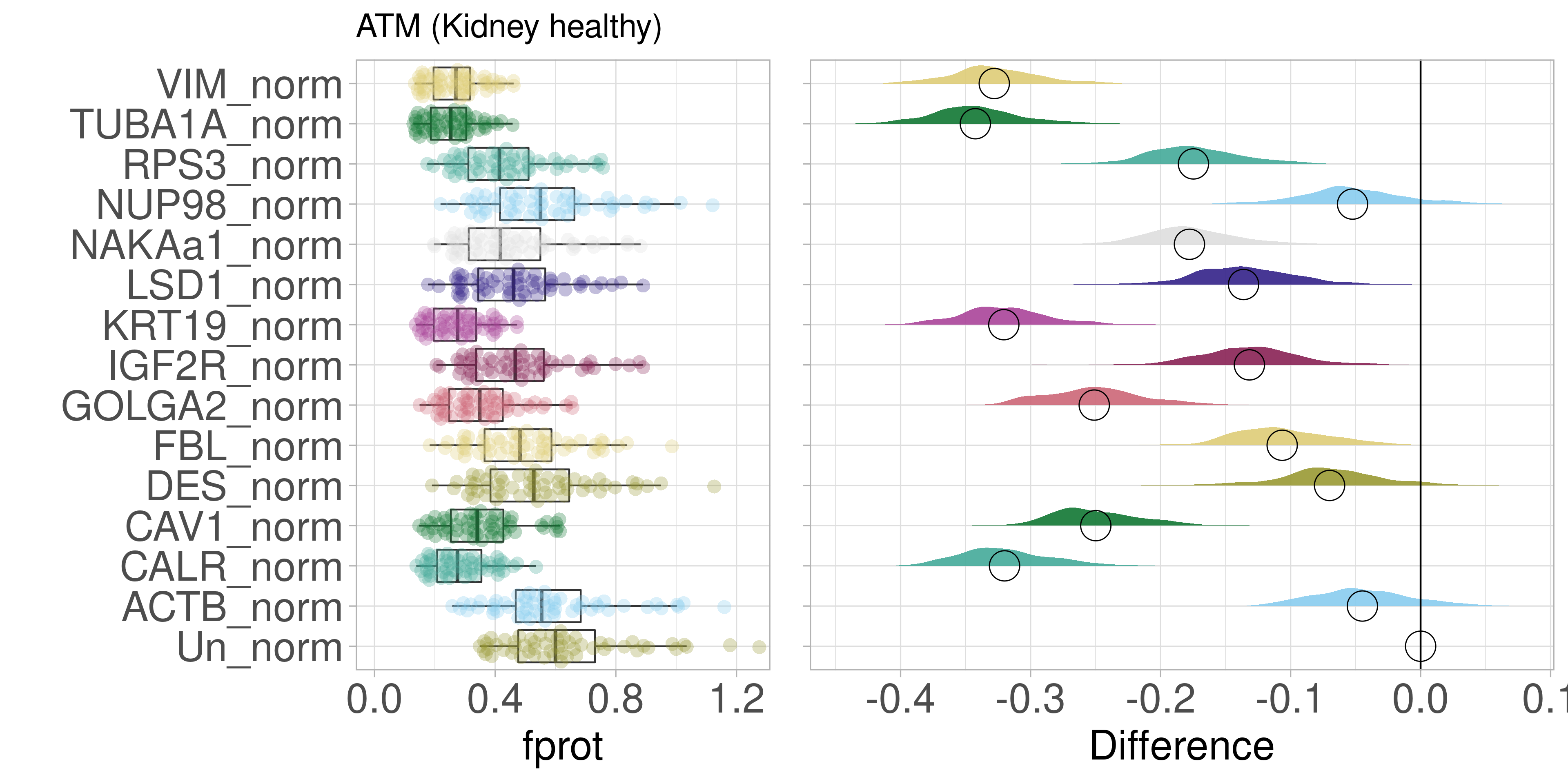

Supplement: Supplementary file 17 — Supplementary Material 17 [file 41598_2026_48754_MOESM17_ESM.zip › RPPA normalizations to cell markers/Kidney_plots/Tumor_suppr_Kidney/ATM_Kidney_H.png]

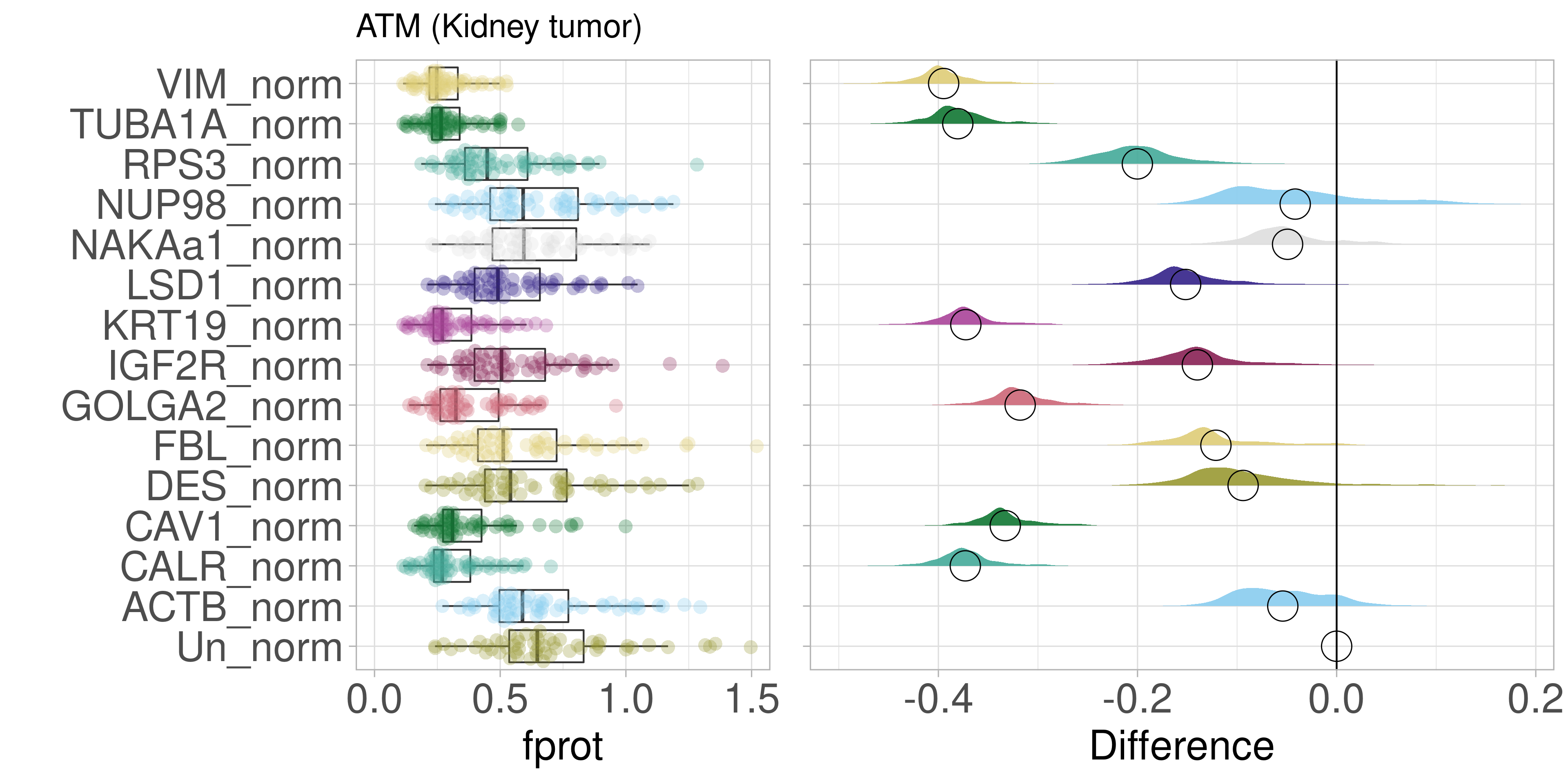

Supplement: Supplementary file 17 — Supplementary Material 17 [file 41598_2026_48754_MOESM17_ESM.zip › RPPA normalizations to cell markers/Kidney_plots/Tumor_suppr_Kidney/ATM_Kidney_T.png]

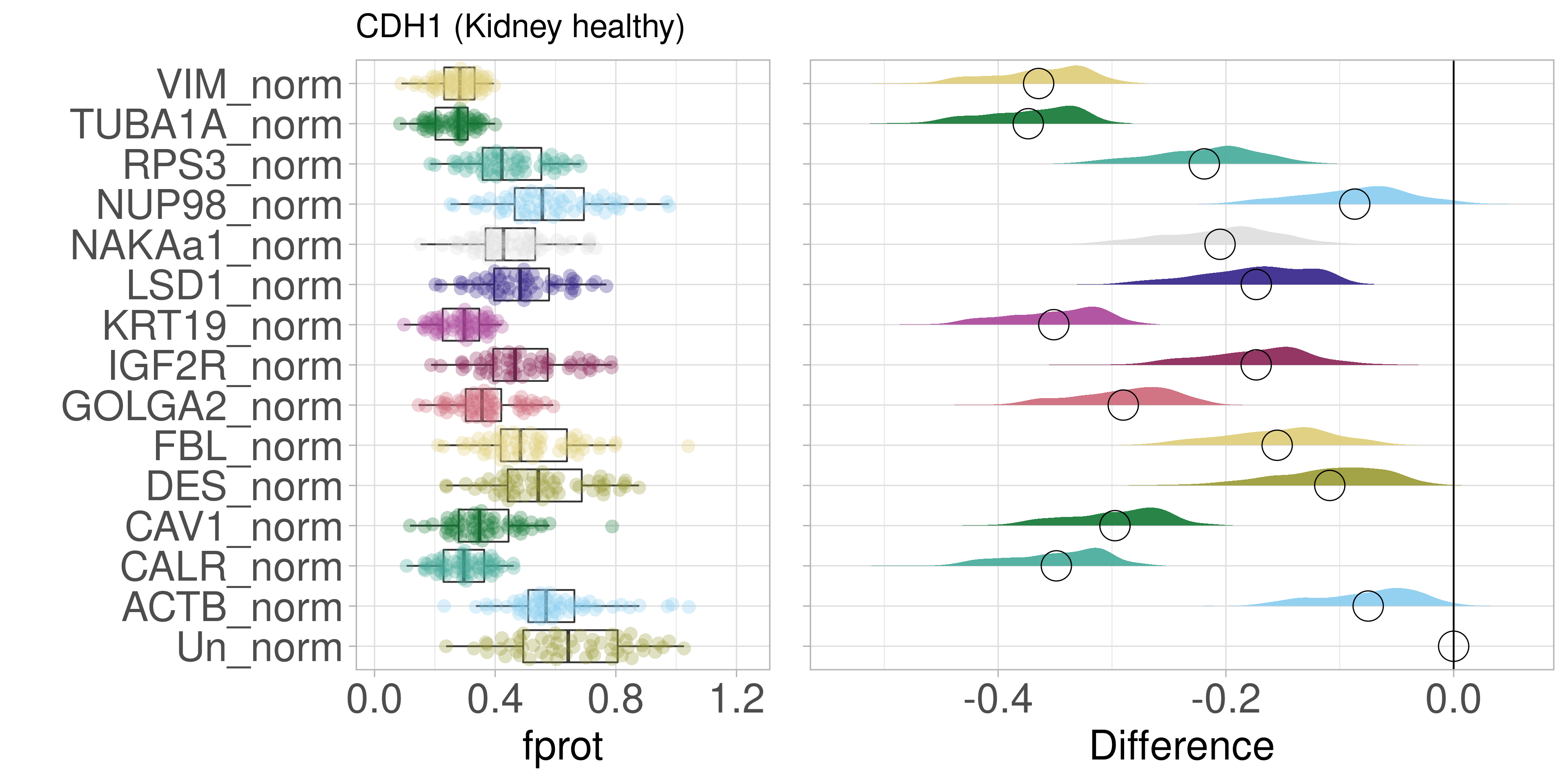

Supplement: Supplementary file 17 — Supplementary Material 17 [file 41598_2026_48754_MOESM17_ESM.zip › RPPA normalizations to cell markers/Kidney_plots/Tumor_suppr_Kidney/CDH1_Kidney_H.png]

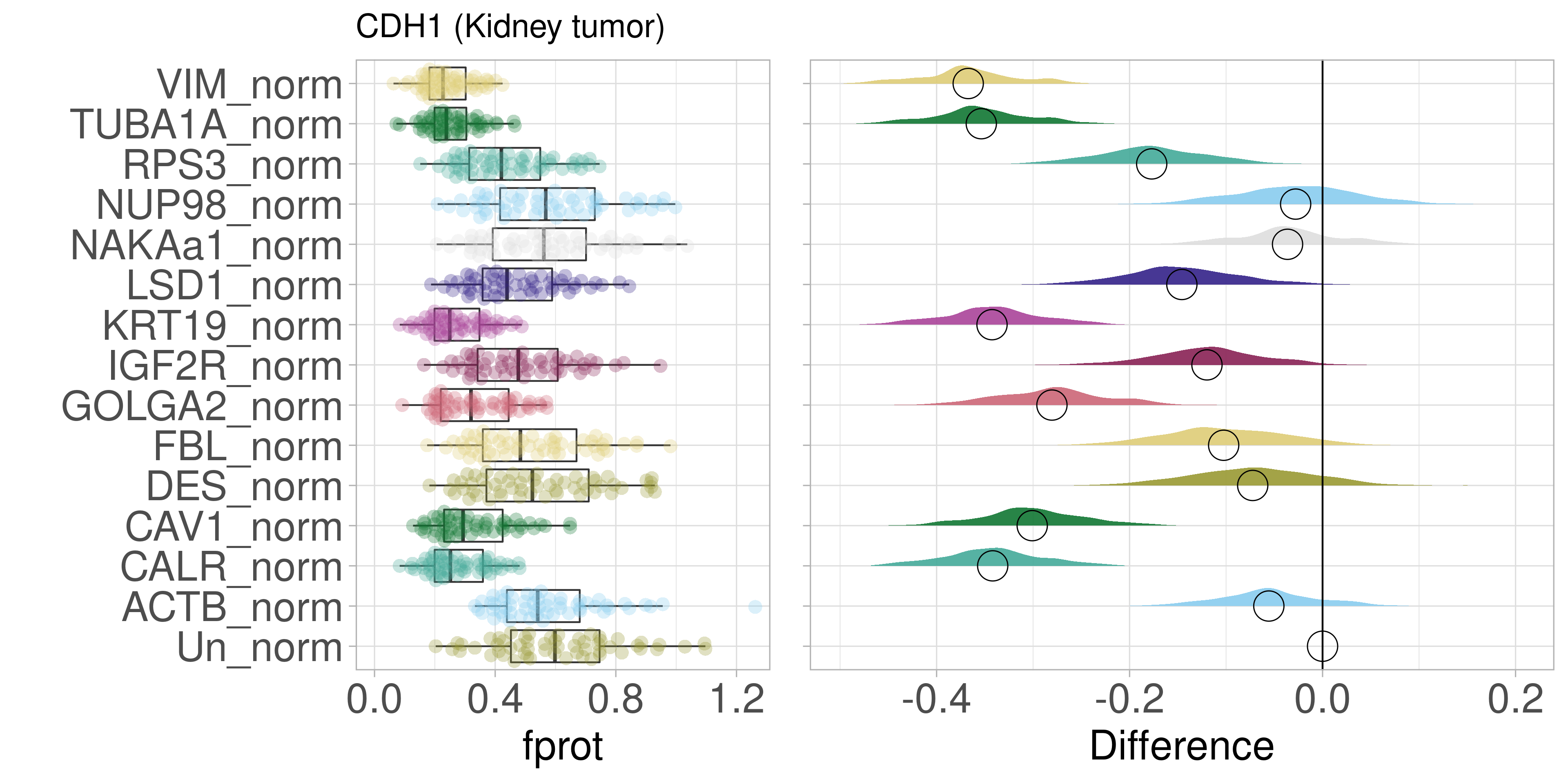

Supplement: Supplementary file 17 — Supplementary Material 17 [file 41598_2026_48754_MOESM17_ESM.zip › RPPA normalizations to cell markers/Kidney_plots/Tumor_suppr_Kidney/CDH1_Kidney_T.png]

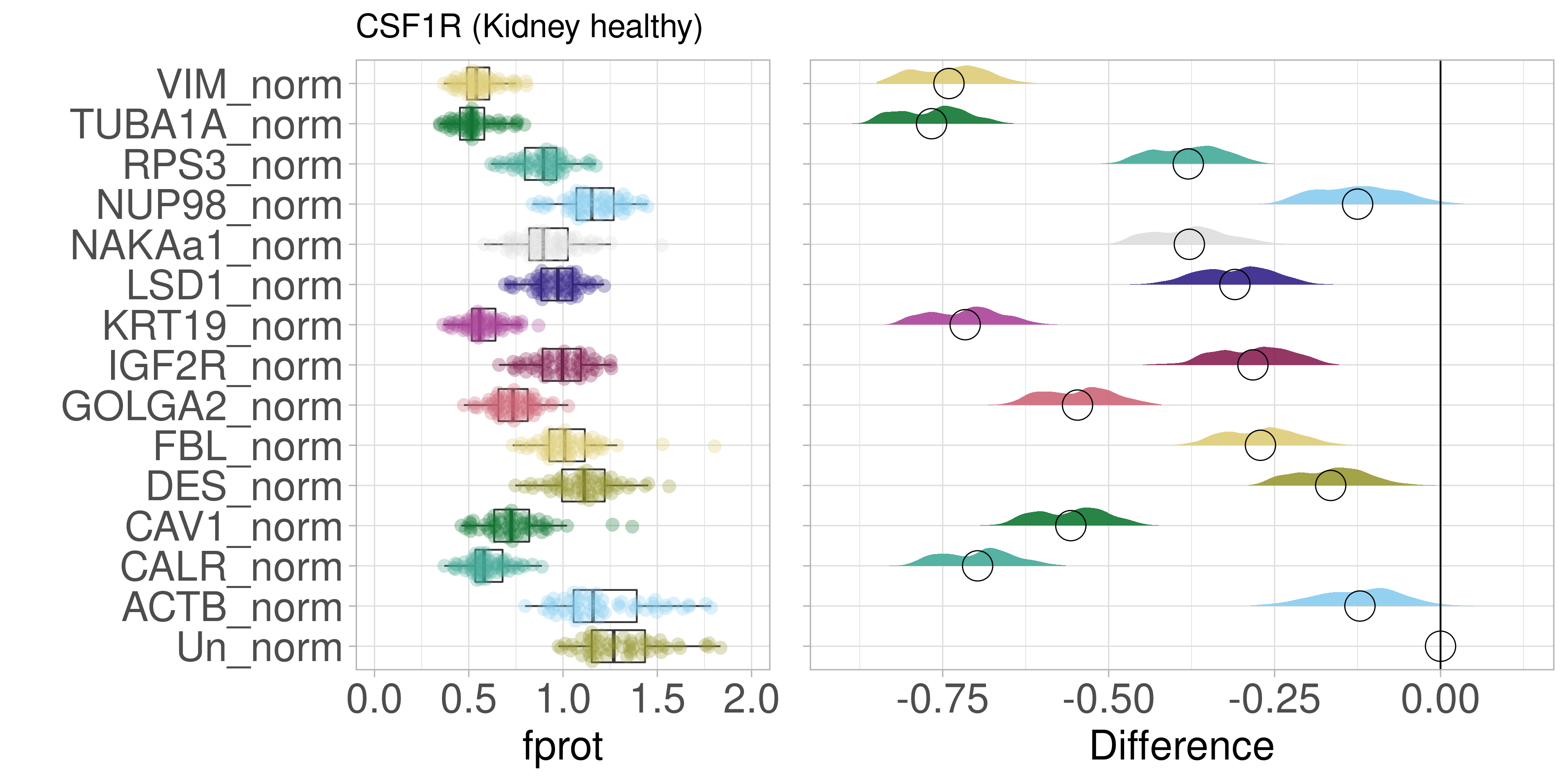

Supplement: Supplementary file 17 — Supplementary Material 17 [file 41598_2026_48754_MOESM17_ESM.zip › RPPA normalizations to cell markers/Kidney_plots/Tumor_suppr_Kidney/CSF1R_Kidney_H.png]

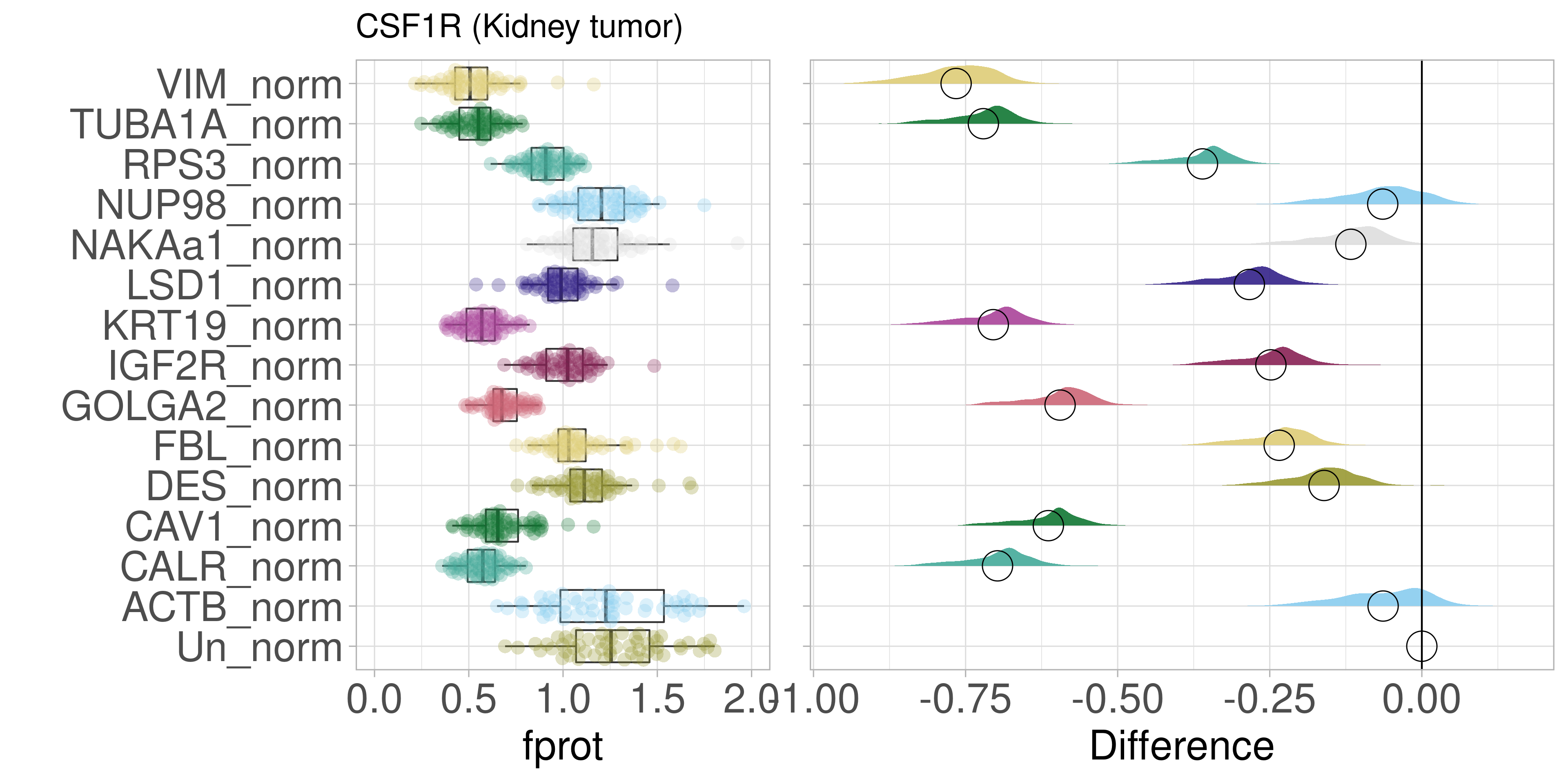

Supplement: Supplementary file 17 — Supplementary Material 17 [file 41598_2026_48754_MOESM17_ESM.zip › RPPA normalizations to cell markers/Kidney_plots/Tumor_suppr_Kidney/CSF1R_Kidney_T.png]

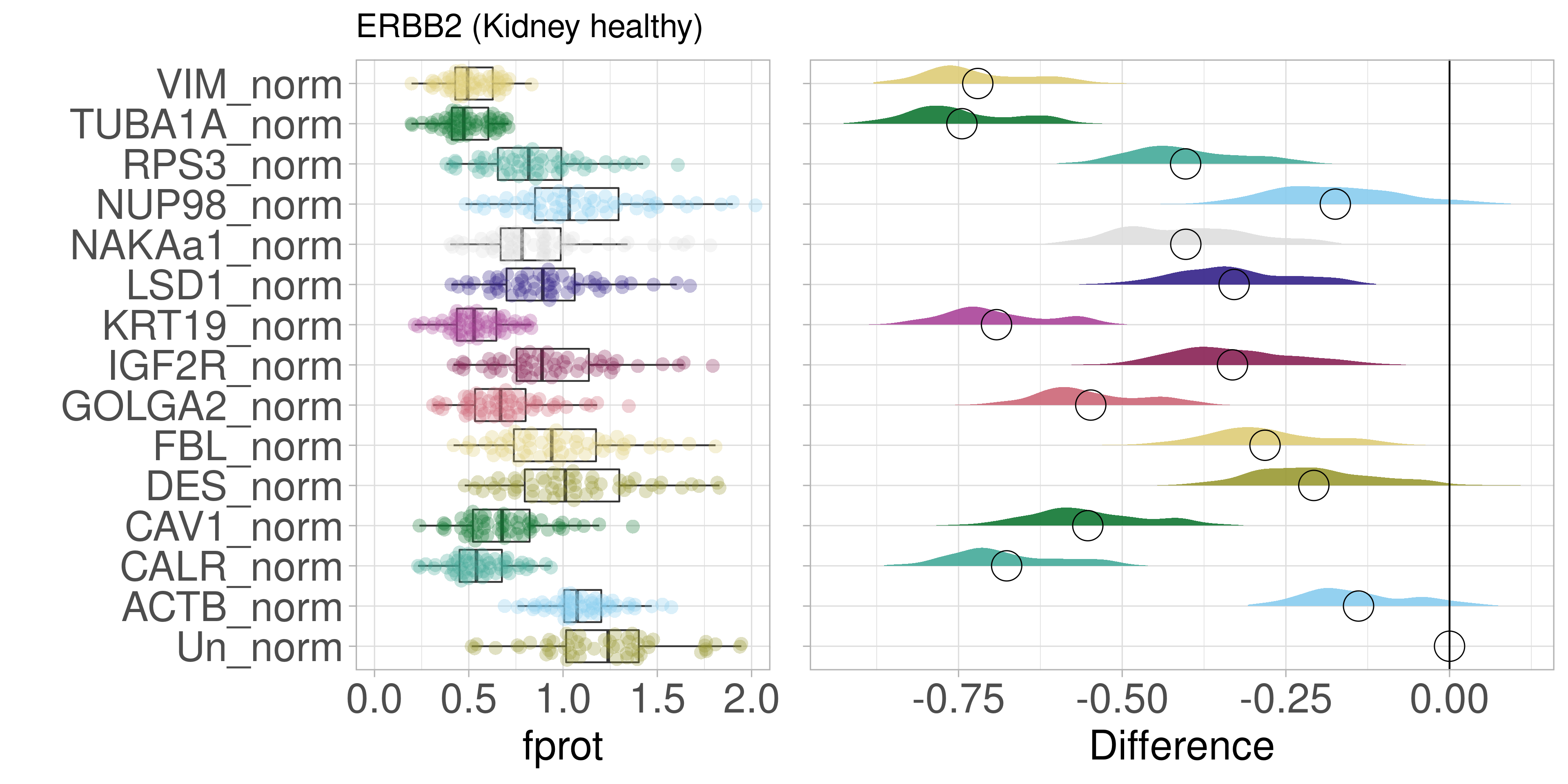

Supplement: Supplementary file 17 — Supplementary Material 17 [file 41598_2026_48754_MOESM17_ESM.zip › RPPA normalizations to cell markers/Kidney_plots/Tumor_suppr_Kidney/ERBB2_Kidney_H.png]

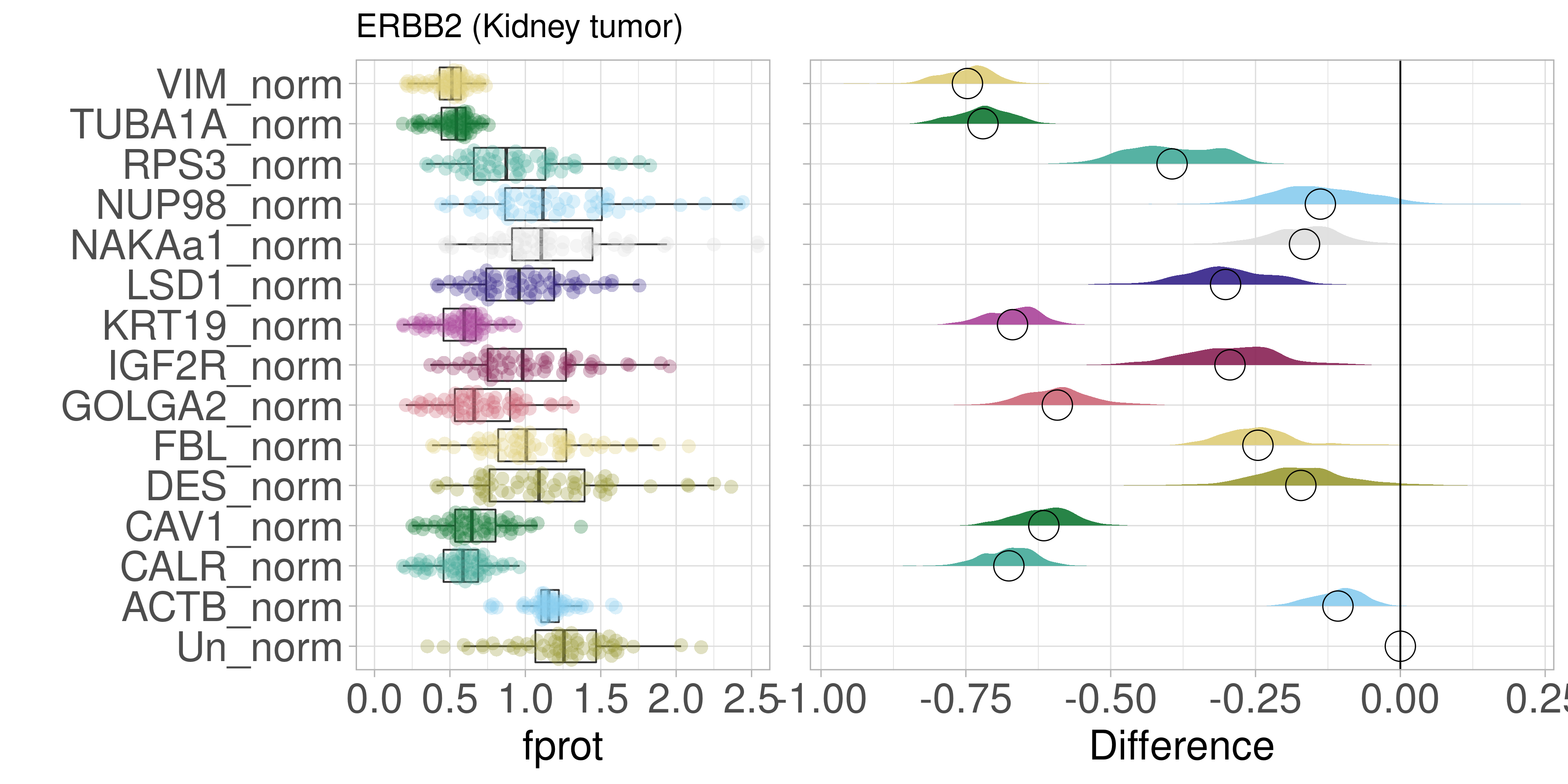

Supplement: Supplementary file 17 — Supplementary Material 17 [file 41598_2026_48754_MOESM17_ESM.zip › RPPA normalizations to cell markers/Kidney_plots/Tumor_suppr_Kidney/ERBB2_Kidney_T.png]

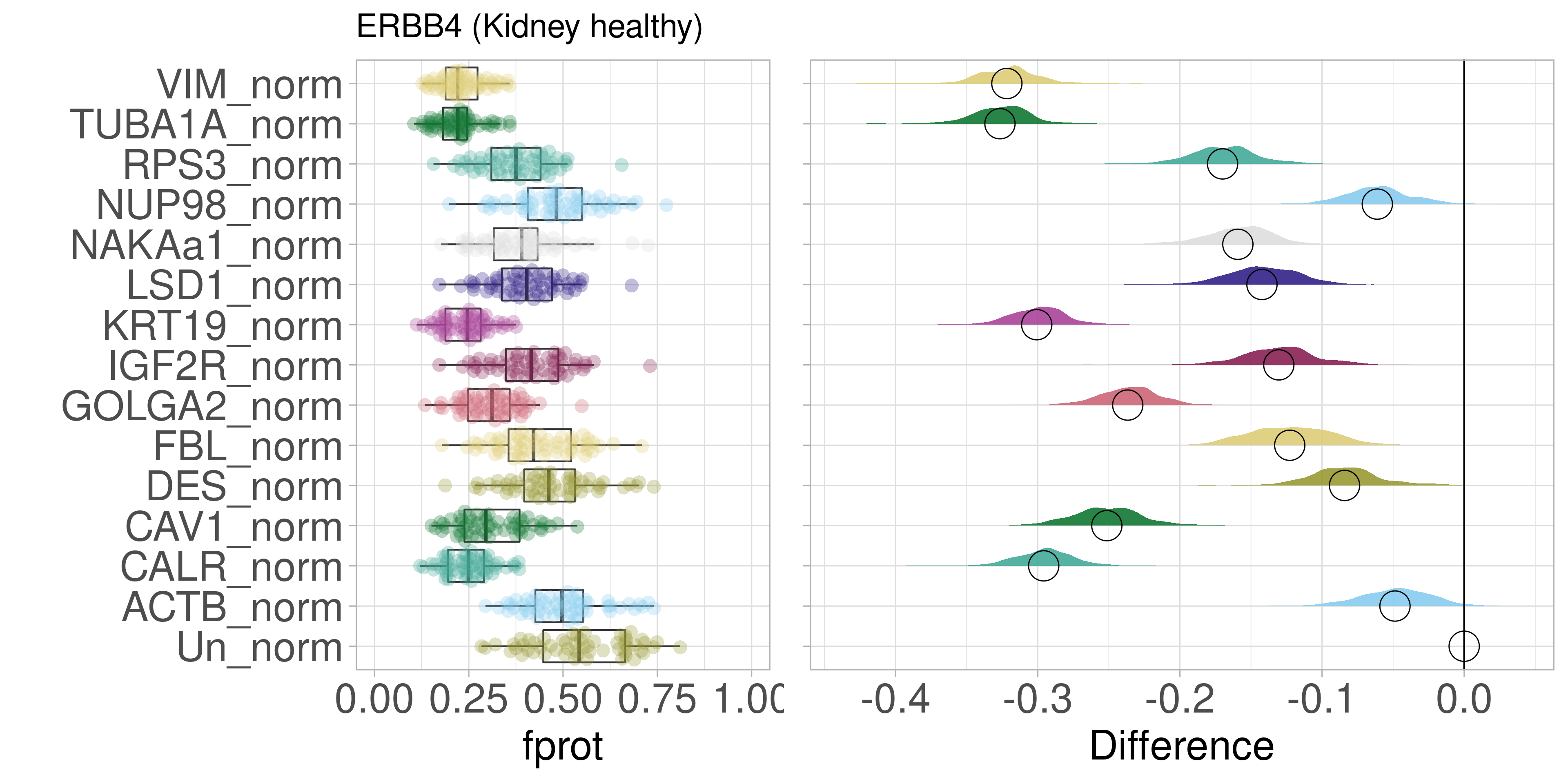

Supplement: Supplementary file 17 — Supplementary Material 17 [file 41598_2026_48754_MOESM17_ESM.zip › RPPA normalizations to cell markers/Kidney_plots/Tumor_suppr_Kidney/ERBB4_Kidney_H.png]

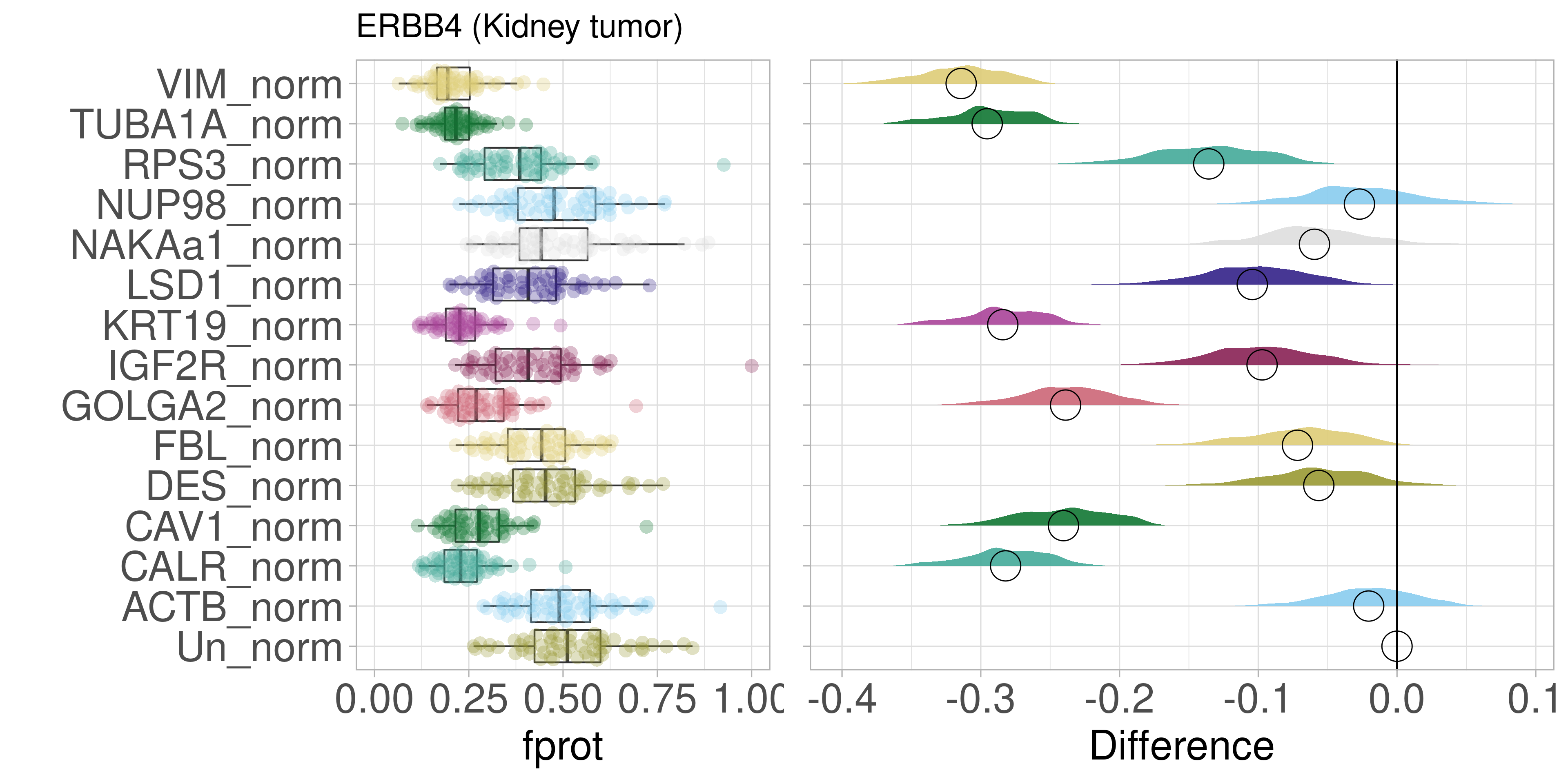

Supplement: Supplementary file 17 — Supplementary Material 17 [file 41598_2026_48754_MOESM17_ESM.zip › RPPA normalizations to cell markers/Kidney_plots/Tumor_suppr_Kidney/ERBB4_Kidney_T.png]

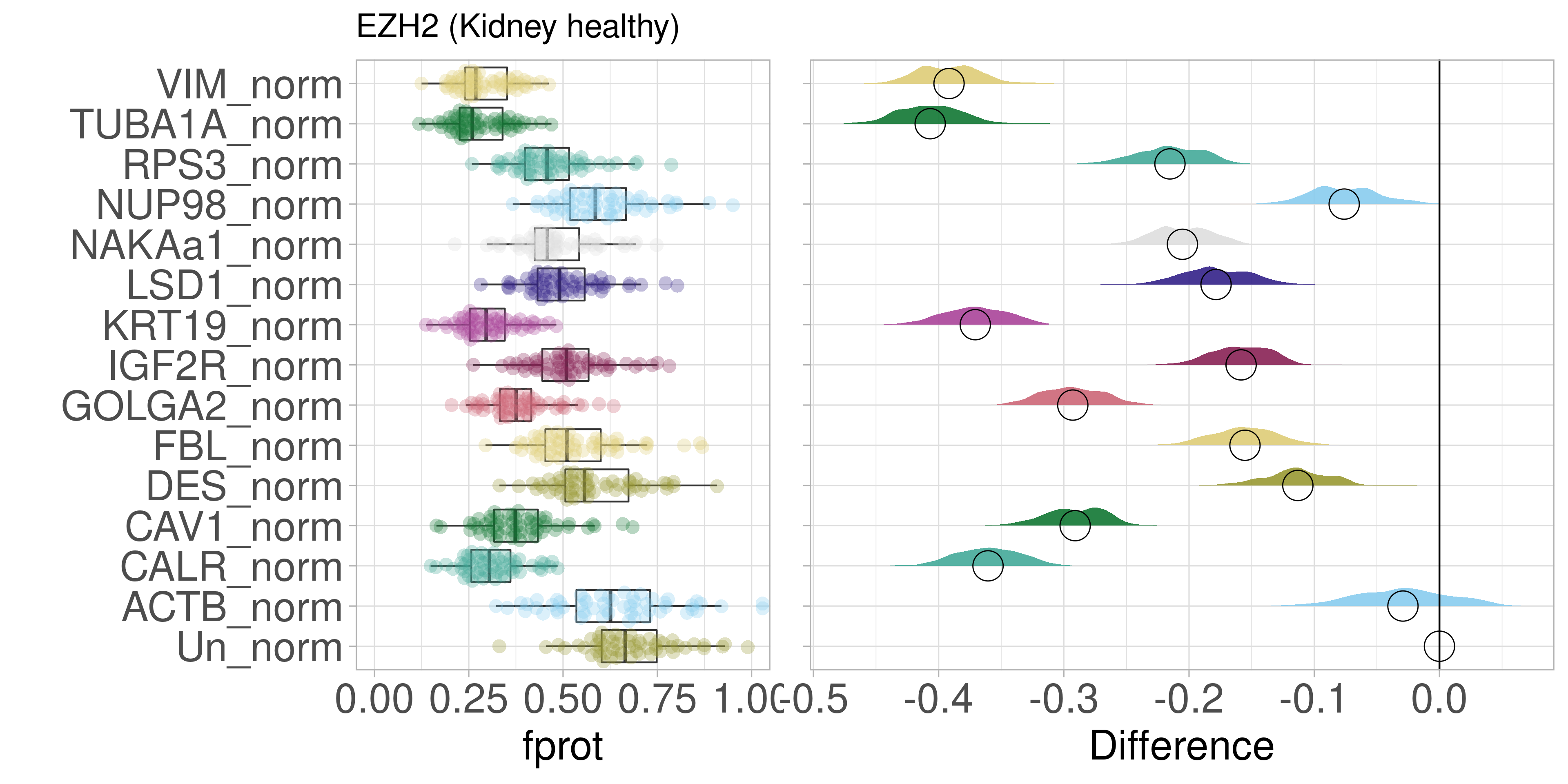

Supplement: Supplementary file 17 — Supplementary Material 17 [file 41598_2026_48754_MOESM17_ESM.zip › RPPA normalizations to cell markers/Kidney_plots/Tumor_suppr_Kidney/EZH2_Kidney_H.png]

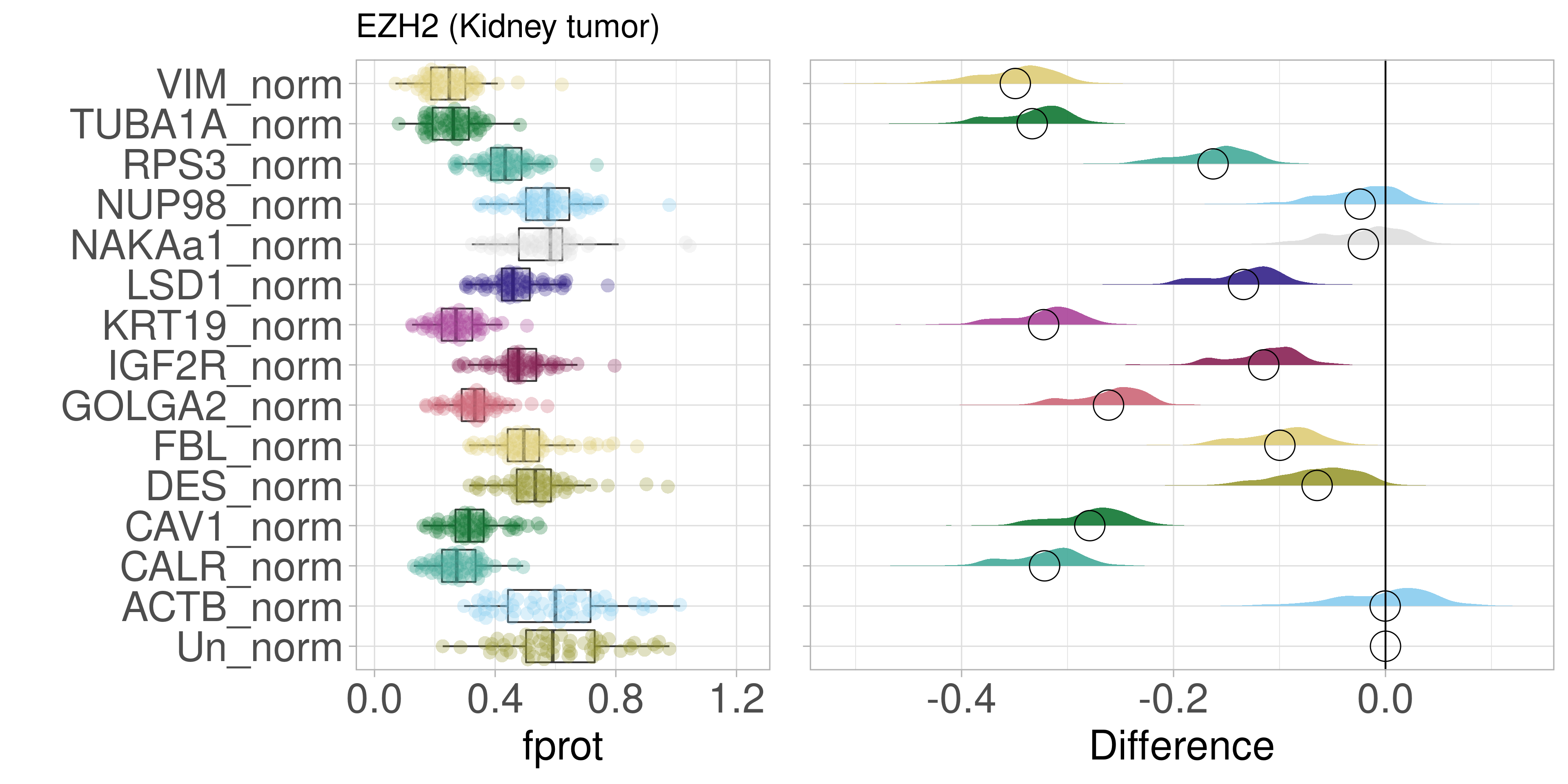

Supplement: Supplementary file 17 — Supplementary Material 17 [file 41598_2026_48754_MOESM17_ESM.zip › RPPA normalizations to cell markers/Kidney_plots/Tumor_suppr_Kidney/EZH2_Kidney_T.png]

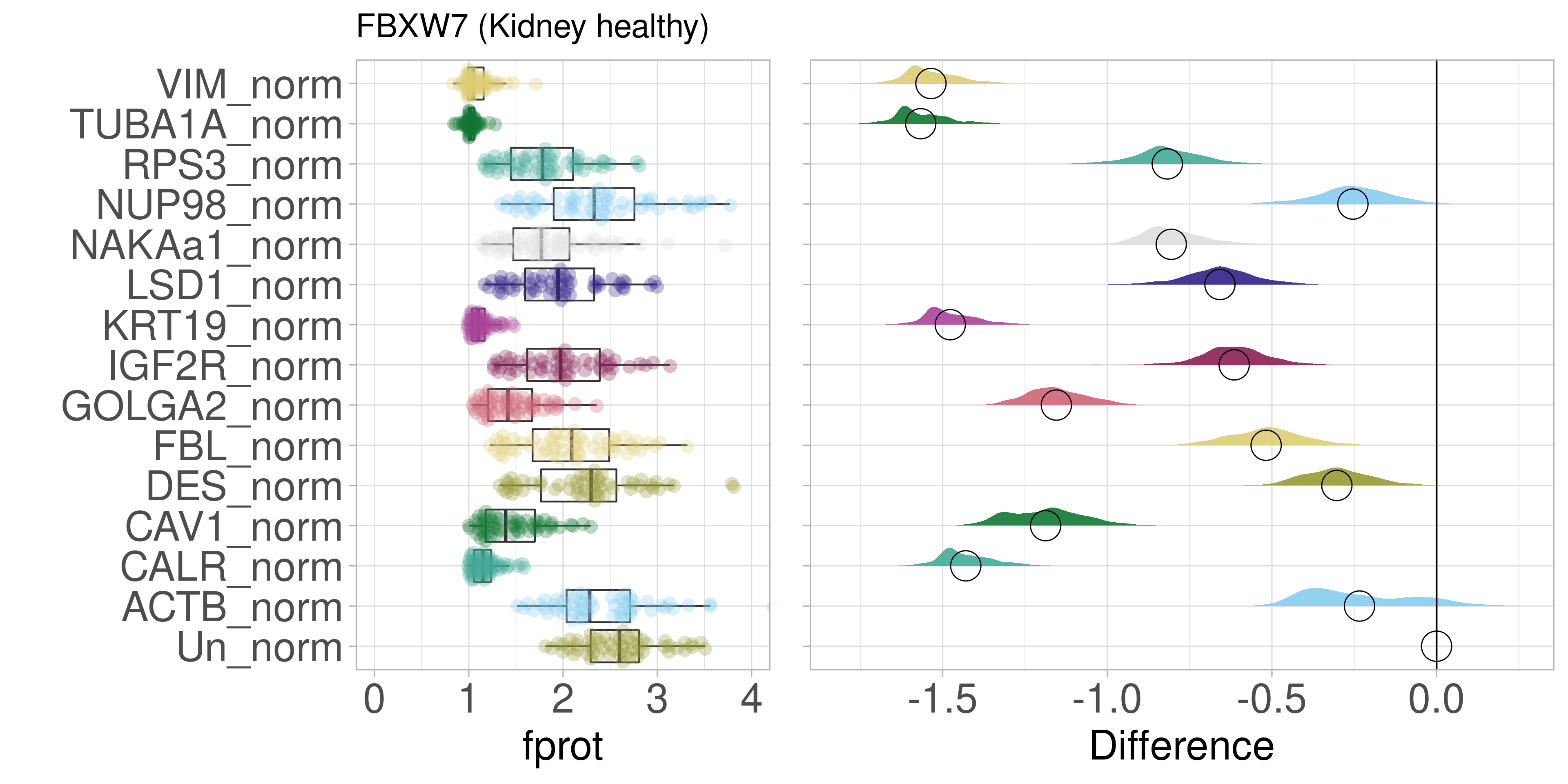

Supplement: Supplementary file 17 — Supplementary Material 17 [file 41598_2026_48754_MOESM17_ESM.zip › RPPA normalizations to cell markers/Kidney_plots/Tumor_suppr_Kidney/FBXW7_Kidney_H.png]

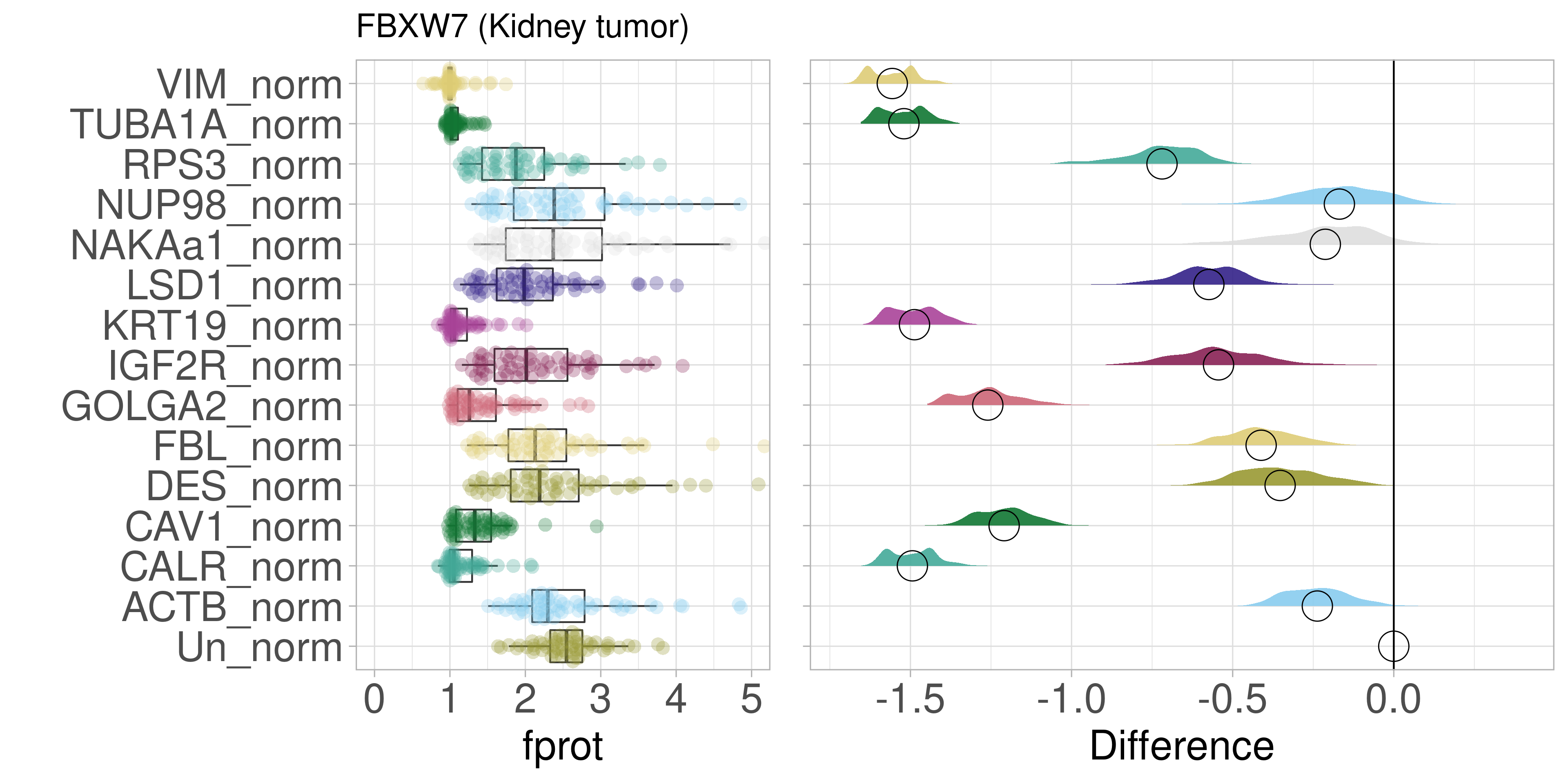

Supplement: Supplementary file 17 — Supplementary Material 17 [file 41598_2026_48754_MOESM17_ESM.zip › RPPA normalizations to cell markers/Kidney_plots/Tumor_suppr_Kidney/FBXW7_Kidney_T.png]

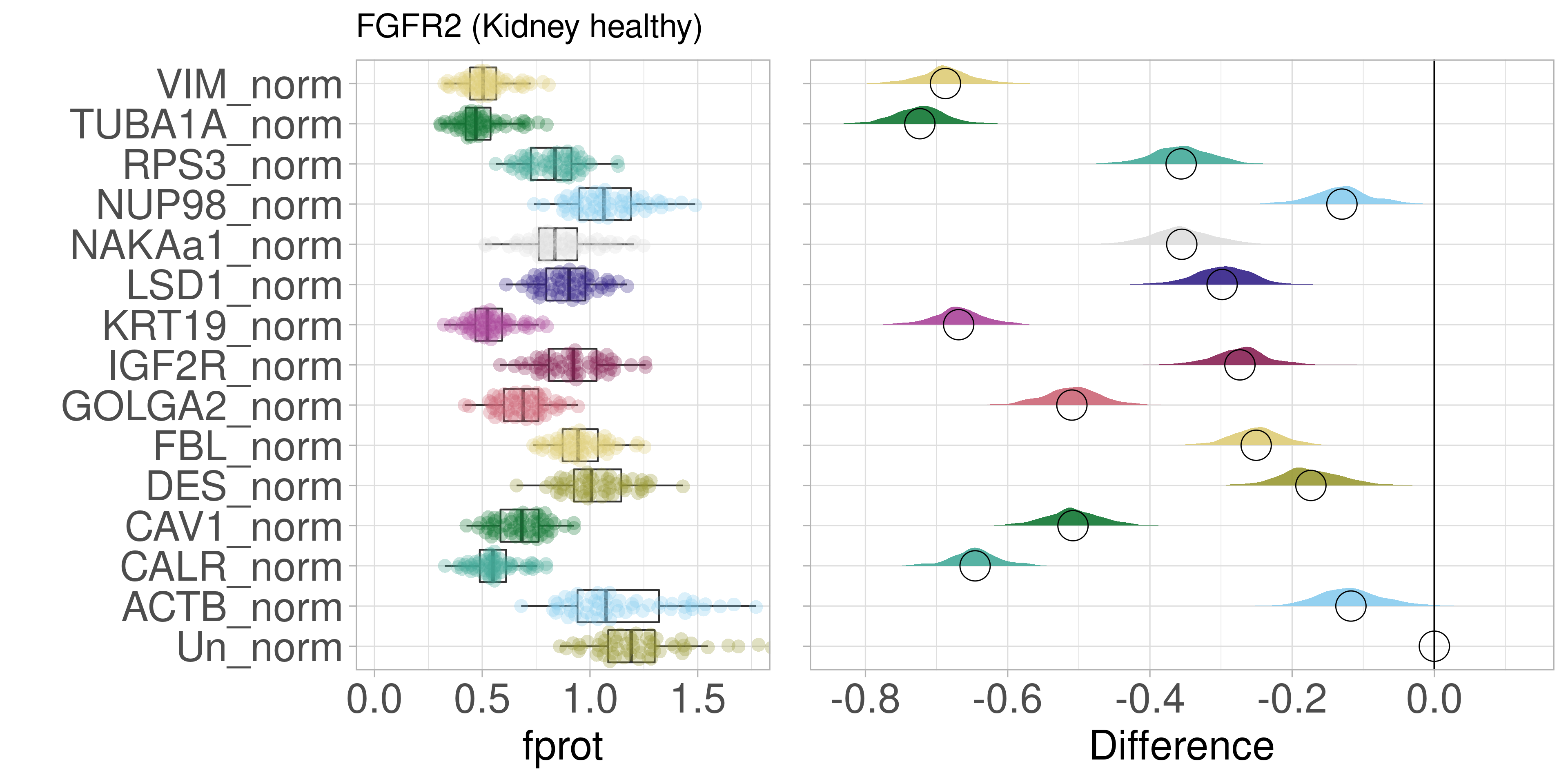

Supplement: Supplementary file 17 — Supplementary Material 17 [file 41598_2026_48754_MOESM17_ESM.zip › RPPA normalizations to cell markers/Kidney_plots/Tumor_suppr_Kidney/FGFR2_Kidney_H.png]

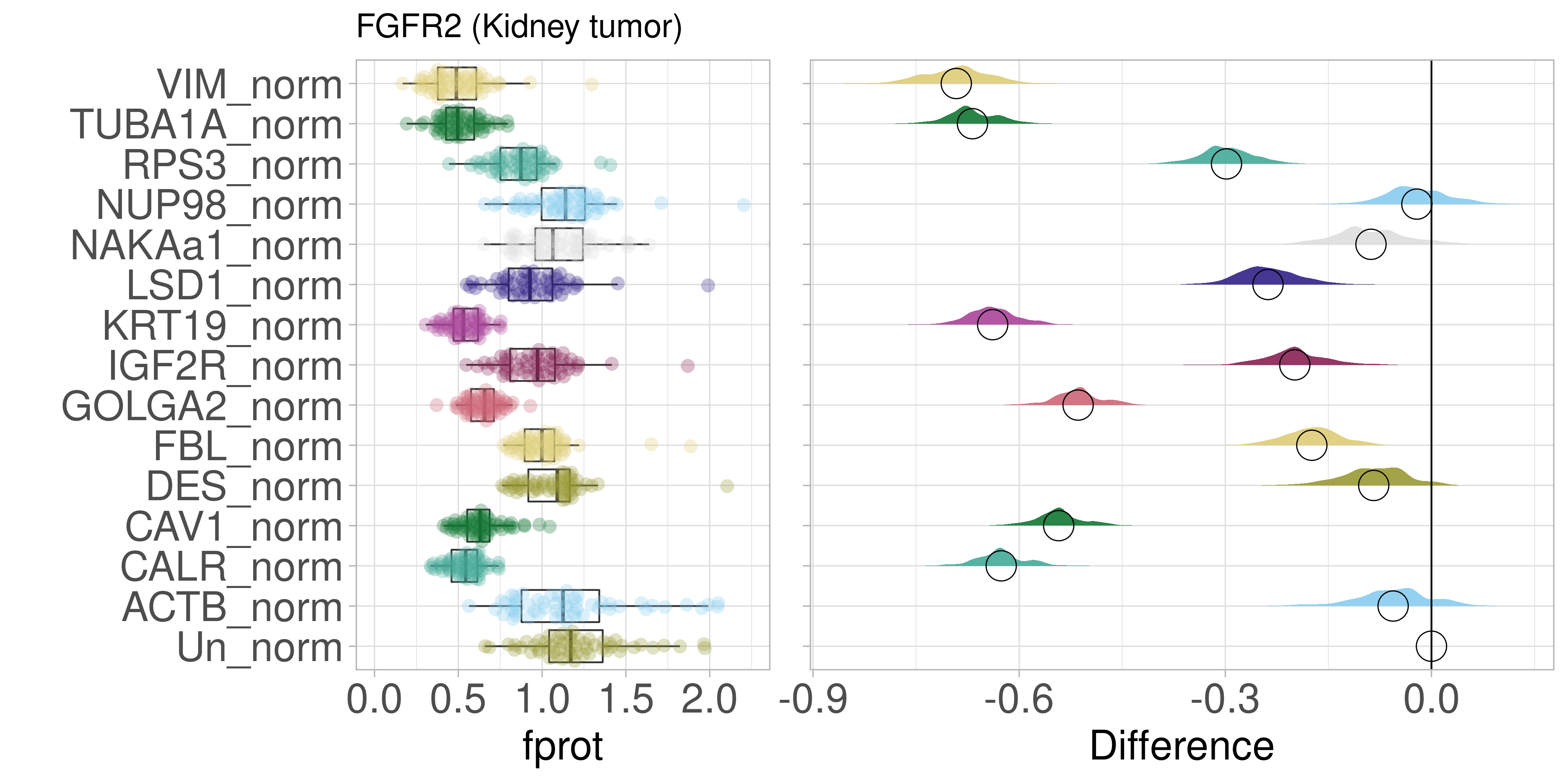

Supplement: Supplementary file 17 — Supplementary Material 17 [file 41598_2026_48754_MOESM17_ESM.zip › RPPA normalizations to cell markers/Kidney_plots/Tumor_suppr_Kidney/FGFR2_Kidney_T.png]
