# Supplementary material for: Comparative analysis and correlation of cancer hotspot proteins and cell markers in tumor-normal adjacent breast and kidney samples using RPPA and LC-MS
Source: Sci Rep. 2026 May 18;16:22442. doi: 10.1038/s41598-026-48754-2 (PMC13377106; doi:10.1038/s41598-026-48754-2)
Supplement: Supplementary file 18 — Supplementary Material 18 [file 41598_2026_48754_MOESM18_ESM.docx]

**Supplementary material**

Comparative Analysis and Correlation of Cancer Hotspot Proteins and Cell Markers in Tumor-Normal Adjacent Breast and Kidney Samples Using RPPA and LC-MS

Krisztina Paal^1†^, Noemi Karnok^1†^, Csaba Konrad^2^, David Bui^1^, Fanni Bugyi^3^, Lilla Turiák^3^, Christos Chinopoulos^1*^

^1^Department of Biochemistry, Semmelweis University, 1094, Budapest, Hungary

^2^Feil Family Brain and Mind Research Institute, Weill Cornell Medicine, New York, NY 10065, USA

^3^MTA-TTK Lendület (Momentum) Glycan Biomarker Research Group, HUN-REN Research Centre for Natural Sciences, 1117, Budapest, Hungary

^†^These authors contributed equally to this work

^*^Correspondence and lead contact: [chinopoulos.christos@semmelweis.hu](mailto:chinopoulos.christos@semmelweis.hu)

Legend to supplementary figure 1: A: Violin plots of the age of patients (in years) from whom the tumor-normal adjacent human breast and kidney samples were obtained. B: Example of dilution ratio vs protein stain data and fitted calibration curve and R^2^ that was used for sample normalization. C: Histogram of all fprot RPPA reads of Vimentin antibody stain, indicating run (grey - Run 1, red – Run 2) D: Histogram of all fprot RPPA reads of Vimentin antibody stain after batch normalization.

Legend to supplementary figure 2: MA plots (mean average A versus log ratio, M) of RPPA tumor-normal comparisons for all breast (top left) and kidney (bottom left) samples, calculated from the dilution-ratio/protein-stain normalization workflow described in supplementary figure 1B. Dots (blue for breast and green for kidney) represent individual protein-expression measurements. The red dashed line marks M = 0. The right panels show an example patient (BR8VM69; breast, top: not normalized, bottom: median normalized). These plots are provided as exploratory supplementary visualizations only. Some panels show what appear to be strong intensity-dependent biases and unusual directional shifts; because the measured features derive from a targeted cancer-hotspot/cell-marker panel rather than an unbiased proteome-wide dataset, these MA plots are not used as stand-alone evidence for global up- or down-regulation or for an underlying systemic technical error.

Legend to supplementary figure 3: Patient-level MA plots of all RPPA breast tumor–adjacent normal paired samples. In these MA plots, the x-axis (A) represents the average log2 RPPA signal intensity of the matched tumor and adjacent normal sample pair, and the y-axis (M) represents the log2 difference between the paired tumor and adjacent normal RPPA signals. These plots are shown for descriptive completeness only. Several panels show what appear to be strong intensity-dependent biases and unusual directional shifts; because they derive from a targeted cancer-hotspot/cell-marker panel, they are not interpreted as stand-alone evidence for global up- or down-regulation or for an underlying systemic technical error.

Legend to supplementary figure 4: Patient-level MA plots of all RPPA kidney tumor–adjacent normal paired samples. In these MA plots, the x-axis (A) represents the average log2 RPPA signal intensity of the matched tumor and adjacent normal sample pair, and the y-axis (M) represents the log2 difference between the paired tumor and adjacent normal RPPA signals. These plots are shown for descriptive completeness only. Several panels show what appear to be strong intensity-dependent biases and unusual directional shifts; because they derive from a targeted cancer-hotspot/cell-marker panel, they are not interpreted as stand-alone evidence for global up- or down-regulation or for an underlying systemic technical error.

Legend to supplementary figure 5: Quasi-random distribution of expression levels (fprot, determined by RPPA) of all oncoproteins in breast tumor-normal adjacent samples. Black lines indicate mean +/- 1 SD. In the right panels, the tumor-normal breast (top right) matching (connected by a line) from each same patient fprot are depicted; blue vs red line indicates a decrease vs an increase in fprot value (healthy as control).

Legend to supplementary figure 6: Quasi-random distribution of expression levels (fprot, determined by RPPA) of all tumor suppressor proteins in breast tumor-normal adjacent samples. Black lines indicate mean +/- 1 SD. In the right panels, the tumor-normal breast (top right) matching (connected by a line) from each same patient fprot are depicted; blue vs red line indicates a decrease *vs* an increase in fprot value (healthy as control).

Legend to supplementary figure 7: Quasi-random distribution of expression levels (fprot, determined by RPPA) of all cell marker proteins in breast tumor-normal adjacent samples. Black lines indicate mean +/- 1 SD. In the right panels, the tumor-normal breast (top right) matching (connected by a line) from each same patient fprot are depicted; blue vs red line indicates a decrease *vs* an increase in fprot value (healthy as control).

Legend to supplementary figure 8: Quasi-random distribution of expression levels (fprot, determined by RPPA) of all oncoproteins in kidney tumor-normal adjacent samples. Black lines indicate mean +/- 1 SD. In the right panels, the tumor-normal kidney (top right) matching (connected by a line) from each same patient fprot are depicted; blue vs red line indicates a decrease *vs* an increase in fprot value (healthy as control).

Legend to supplementary figure 9: Quasi-random distribution of expression levels (fprot, determined by RPPA) of all tumor suppressor proteins in kidney tumor-normal adjacent samples. Black lines indicate mean +/- 1 SD. In the right panels, the tumor-normal kidney (top right) matching (connected by a line) from each same patient fprot are depicted; blue vs red line indicates a decrease *vs* an increase in fprot value (healthy as control).

Legend to supplementary figure 10: Quasi-random distribution of expression levels (fprot, determined by RPPA) of all cell marker proteins in kidney tumor-normal adjacent samples. Black lines indicate mean +/- 1 SD. In the right panels, the tumor-normal kidney (top right) matching (connected by a line) from each same patient fprot are depicted; blue vs red line indicates a decrease *vs* an increase in fprot value (healthy as control).

Legend to supplementary figure 11: Spearman correlation heatmaps for kidney tumor-matched samples. Each panel represents the correlation coefficients between different methods used to measure protein expression levels (tumor/healthy ratios) in kidney tissue samples from four patients (P1-4). The color bar on the bottom right heatmap indicates the range of Spearman correlation coefficients, where 1.0 represents a perfect positive correlation and -1.0 represents a perfect negative correlation.

Legend to supplementary figure 12: Representative Western blots for selected antibodies/proteins using tumor and tumor-adjacent samples from the same lysate aliquots analyzed by RPPA and LC-MS. The primary purpose of these blots is to demonstrate that antibodies used for RPPA yield predominantly single bands at the expected molecular weight under our assay conditions, thereby supporting their suitability for the dot-based RPPA assay. The blots are shown as qualitative antibody-validation and illustrative examples only and were not used as a third quantitative platform for formal correlation analysis with RPPA or LC-MS. Detailed information regarding all antibodies can be found in db.rppa.hu portal (registration required; contact the corresponding author C.C.).

Legend to “supplementary dataset RPPA normalizations to cell markers”: Can be downloaded from <http://rppa.hu/suppl_mcp.html>. Jittered dot graphs showing RPPA data (fprot) of cancer hotspot panel proteins (indicated on the top of each panel) ratioed to the levels of cell markers expression, in breast or kidney healthy or tumor sample-matched manner. Un_norm: un-normalized to a cell marker. The summaries of the data are shown as a boxplot, with the box indicating the interquartile range and vertical line indicating the median. Plots on the right show the effect size, relative to Un_norm, which is the protein indicated on the top of each panel. The bootstrap samples that are used to calculate the 95%CI of the effect size are shown as a distribution.

Legend to “Supplementary dataset LC-MS and Spearman”: Can be downloaded from <http://rppa.hu/suppl_mcp.html>. Comparison of all proteins expression between 8 tumor-normal adjacent breast samples from 4 patients estimated by RPPA or LC-MS normalized by various methods, as indicated on the y-axis of each panel. Spearman correlation coefficients for pairwise comparisons of the above data obtained by RPPA or LC-MS for all proteins expression.
