# Supplementary material for: Comparative analysis and correlation of cancer hotspot proteins and cell markers in tumor-normal adjacent breast and kidney samples using RPPA and LC-MS
Source: Sci Rep. 2026 May 18;16:22442. doi: 10.1038/s41598-026-48754-2 (PMC13377106; doi:10.1038/s41598-026-48754-2)
Supplement: Supplementary file 19 — Supplementary Material 19 [file 41598_2026_48754_MOESM19_ESM.docx]

Supplementary Table 3. Name of cell markers, abbreviations (used in the text and figures), Uniprot identifiers, Research Resource Identifiers (RRIDs) of antibodies used in RPPA and their titers.

| **Name of protein** | **Abbreviation** | **Uniprot** | **RRID** | **Titer** |
| --- | --- | --- | --- | --- |
| α-tubulin | TUBA1A | Q71U36 | AB_2619646 | 1:4,000 |
| β-actin | ACTB | P60709 | AB_2223210 | 1:10,000 |
| Calreticulin | CALR | P27797 | AB_2688013 | 1:4,000 |
| Caveolin-1 | CAV1 | Q03135 | AB_2275453 | 1:4,000 |
| Desmin | DES | P17661 | AB_1903947 | 1:4,000 |
| Fibrillarin | FBL | P22087 | AB_2278087 | 1:4,000 |
| Golgi matrix protein 130 | GOLGA2 | Q08379 | AB_2797933 | 1:4,000 |
| Insulin-like growth factor II | IGF2R | P11717 | AB_2798462 | 1:4,000 |
| Keratin 19 | KRT19 | P08727 | AB_2722626 | 1:4,000 |
| Lysine-specific histone demethylase 1A | LSD1 | O60341 | AB_2070132 | 1:4,000 |
| Na^+^/K^+^ ATPase α1 subunit | NAKAa1 | P05023 | AB_2798866 | 1:4,000 |
| Nuclear pore complex protein Nup98-Nup96 | NUP98 | P52948 | AB_2267700 | 1:4,000 |
| Ribosomal Protein S3 | RPS3 | P23396 | AB_10622028 | 1:4,000 |
| Vimentin | VIM | P08670 | AB_10695459 | 1:4,000 |
