# Supplementary material for: Comparative analysis and correlation of cancer hotspot proteins and cell markers in tumor-normal adjacent breast and kidney samples using RPPA and LC-MS
Source: Sci Rep. 2026 May 18;16:22442. doi: 10.1038/s41598-026-48754-2 (PMC13377106; doi:10.1038/s41598-026-48754-2)
Supplement: Supplementary file 21 — Supplementary Material 21 [file 41598_2026_48754_MOESM21_ESM.docx]

Supplementary Table 1. Cancer hotspot panels offered by vendors. Source: <https://app.dimensions.ai>.

| ***Cancer hotspot panel name*** | ***Number of genes examined*** | ***Vendor*** | ***Number of publications**** |
| --- | --- | --- | --- |
| Ion AmpliSeq Cancer Hotspot Panel v2 | 50 | ThermoFisher | 6,850 |
| TruSight Oncology 500 | 523 | Illumina | 578 |
| CleanPlex OncoZoom Cancer Hotspot Kit | 65 | Paragon Genomics | 230 |
| Tapestri Single-cell DNA Tumor Hotspot Panel | 59 | Mission Bio | 334 |
| NEBNext Direct Cancer HotSpot Panel | 50 | New England Biolabs | 1,045 |
| OncoGxOne Discovery cancer panels | 150-400 | GENEWIZ | 10 |
| Azenta Pan-Cancer Panel | 634 | Azenta Life Sciences | 236 |
| Cancer Hotspot Panel | 65 | CD Genomics | 524 |

* publications: datasets, grants, patents, clinical trials, policy documents (mined out of 139,456,791 entries as of October 2023)
